# Supplementary material for: A biomimetic electrostatic assistance for guiding and promoting N-terminal protein chemical modification
Source: Nat Commun. 2022 Nov 5;13:6667. doi: 10.1038/s41467-022-34392-5 (PMC9637170; doi:10.1038/s41467-022-34392-5)
Supplement: Supplementary file 1 — Supplementary Information [file 41467_2022_34392_MOESM1_ESM.pdf]

Supplementary Information

A biomimetic electrostatic assistance for guiding and promoting N-terminal protein chemical modification

Authors: Nathalie Ollivier,[1] Magalie Sénéchal,[1] Rémi Desmet,[1] Benoît Snella,[1] Vangelis Agouridas,[1,2] Oleg Melnyk[1]*

Affiliations:

[1] Univ. Lille, CNRS, Inserm, CHU Lille, Institut Pasteur de Lille, U1019 - UMR 9017; Center for Infection and Immunity of Lille, F-59000 Lille, France.

[2] Centrale Lille; F-59000 Lille, France.

* Corresponding author

Email: oleg.melnyk@ibl.cnrs.fr

Table of content

Supplementary Information 1
1. Supplementary Methods 4
Reagents and solvents 4
Peptide synthesis 4
Analyses 5
Purifications 5
Synthesis of peptide hydrazides 6
Synthesis of peptide thioesters 18
Synthesis of peptide nucleophiles 33
Synthesis of glycyl peptides **2a-g** 33
Production of Gly(Arg)$_6$-I27 titin protein **5** in E. coli 47
Chemical synthesis of Cys-(Arg)$_6$-K1 protein **9** 54
Synthesis of Cys peptides 61
Factors influencing the electrostatic assistance of peptide thioester aminolysis (Fig. 3) 65
Effect of peptide concentration 65
Importance of the charged modules (Fig. 3b) 77
Influence of the number of Arg residues in the cationic module (Fig. 3c) 97
Effect on the number and position of pSer residues (Fig. 3d) 159
Effect of the pH 180
Effect of phosphate buffer concentration 181
Effect of increasing concentrations of Gn·HCl (Fig. 3e) 184
Effect of increasing concentrations of urea (Fig. 3e) 187
Effect of TCEP 190
Effect of *n*-octylglucoside 193
Synthesis of peptide **3a,a** on preparative scale (Fig. 2a) 195
Comparison with an authentic sample produced by SPPS 198
Peptide thioester aminolysis. Competition experiments 202
Competition experiment 1: **1a** + **1d** + **2a** → 202
Competition experiment 2: **1a** + **2a** + **2g** → 204
Aminolysis rate law and dependence to the ionic strength 206
Aminolysis rate law 206
Aminolysis rate dependence to the ionic strength (Fig. 3f) 209

Enzymatic dephosphorylation of peptide thioester aminolysis product 214
Synthesis of I27 titin conjugate **6** (Fig. 2c-e)... 216
Electrostatic assistance of the native chemical ligation (NCL, Fig. 4)............................... 223
Kinetic model 226
Reproducibility 228
Effect of the pH (kinetic studies).. 229
Control experiment with glycyl peptide **2a** (importance of side-chain thiol) 230
Control experiment with peptide CILKEPVHGV-NH_2 (importance of Arg_6 module)... 232
Replacement of pSer residues by glutamic acid residues (NCL ligation products 8f,g). 233
Influence of intramolecular electrostatic competition 235
Synthesis of conjugate **10** (modified kringle 1 HGF domain, Fig. 4d,e) 237
Ligation of CR_6-K1 protein **9** with peptide thioester **1g** 241
Electrostatic assistance of hydrazone ligation 244
Arg·HCl catalyzed, UV monitoring... 244
2. Supplementary References.. 251

1. Supplementary Methods

Reagents and solvents

2-(1*H*-Benzotriazol-1-yl)-1,1,3,3-tetramethyluronium hexafluorophosphate (HBTU), 2-(1*H*-Benzotriazol-1-yl)-1,1,3,3-tetramethylaminium tetrafluoroborate (TBTU), 1-hydroxybenzotriazole (HOBt) and *N*-Fmoc protected amino acids were obtained from Iris Biotech GmbH. Side-chain protecting groups used for the amino acids were Fmoc-Arg(Pbf)-OH, Fmoc-Asn(Trt)-OH, Fmoc-Asp(O*t*Bu)-OH, Fmoc-Gln(Trt)-OH, Fmoc-Glu(O*t*Bu)-OH, Fmoc-His(Trt)-OH, Fmoc-Lys(Boc)-OH, Fmoc-Ser(*t*Bu)-OH, Fmoc-Thr(*t*Bu)-OH, Fmoc-Trp(Boc)-OH, Fmoc-Tyr(*t*Bu)-OH, Fmoc-Cys(S*t*Bu)-OH or Fmoc-Cys(Trt)-OH and Fmoc-Ser(PO(OBzl)OH)-OH.

2-chlorotrityl chloride 1% divinylbenzene cross-linked polystyrene (2-CTC, 100-200 Mesh, 0.6 mmol g^{-1}) was obtained from Iris Biotech GmbH. Hydrazine hydrate (50-60% w/v), sodium nitrite, 4-mercaptophenylacetic acid (97%, MPAA), *tris*(2-carboxyethyl)phosphine hydrochloride (≥ 98%), TCEP), triisopropylsilane (TIS), guanidine hydrochloride (Gn·HCl, ≥ 99%), sodium phosphate dibasic dihydrate (≥ 99%), urea, hydrochloric acid (reagent grade, 37% w/v) and sodium hydroxide (pellets, 97%) were purchased from Sigma-Aldrich. All other reagents were purchased from Acros Organics or Merck and were of the purest grade available.

Peptide synthesis grade *N*,*N*-dimethylformamide (DMF), dichloromethane (CH_2Cl_2), diethylether (Et_2O), acetonitrile (CH_3CN), heptane, LC–MS-grade acetonitrile (CH_3CN, 0.1% TFA), LC–MS-grade water (H_2O, 0.1% TFA), *N*,*N*-diisopropylethylamine (DIEA), acetic anhydride (Ac_2O) were purchased from Biosolve and Fisher Chemical. Trifluoroacetic acid (TFA) was obtained from Biosolve. Formic acid (FA) reagent grade was obtained from Sigma Aldrich. Water was purified with a Milli-Q Ultra Pure Water Purification System.

Peptide synthesis

Peptides were synthesized using standard Fmoc solid phase peptide synthesis methods. Peptide amides were prepared on a NovaSyn TGR solid support (0.25 mmol g^{-1}). Peptide hydrazides were assembled on hydrazine 2-chlorotrityl 1% divinylbenzene cross-linked polystyrene solid support (~ 0.2 mmol g^{-1}).

Unless otherwise stated, peptide elongation was performed at room temperature (rt) using an automated peptide synthesizer. In brief, amino acids (10 equiv) were activated using HBTU (9.5 equiv)/DIEA (10 equiv) in DMF. The peptidyl solid support was acetylated after each coupling step using Ac_2O/DIEA/DMF 10/5/85 v/v/v. The removal of the Fmoc group was performed by treating the peptidyl solid support with DMF/piperidine 80/20 v/v. After elongation, the peptidyl solid support was washed with DMF (3 × 1 min), DCM (3 × 1 min) and Et_2O (2 × 1 min). The solid support was finally dried in vacuo. The experimental conditions used for the final cleavage and deprotection step are indicated for each peptide. Typically, 10

mL of TFA supplemented with the appropriate scavengers were used for 0.1 mmole of peptidyl solid support.

Analyses

Products were characterized by analytical UPLC–MS using a System Ultimate 3000 UPLC (Thermofisher) equipped with an Acquity peptide BEH300 C18 column (1.7 µm, 2.1 × 100 mm) or an Agilent SB C3 column (1.8 µm, 3.0 × 100 mm), a diode array detector and a mass spectrometer (Ion trap LCQfleet, heat temperature 350 °C, spray voltage 2.8 kV, capillary temperature 350 °C, capillary voltage 10 V, tube lens voltage 75 V). Analyses were performed at 50 °C using a linear gradient of 0-40% of eluent B in eluent A over 15 min at a flow rate of 0.4 mL min^{-1} (eluent A = 0.1% TFA in H_2O; eluent B = 0.1% TFA in CH_3CN).

Products were in some cases characterized by analytical LC–MS (Waters 2695 LC/ZQ 2000 quadripole) on a reverse phase XBridge BEH300 C18 column (3.5 µm, 300 Å, 4.6 × 150 mm) at 30 °C using a linear gradient of 0-100% of eluent B in eluent A over 30 min at a flow rate of 1 mL/min (eluent A = 0.1% TFA in H_2O; eluent B = 0.1% TFA in CH_3CN/H_2O: 4/1 by vol) or on a reverse phase Zorbax 300SB-C3 column (3.5 µm, 300 Å, 4.6 × 150 mm) at 50 °C using a linear gradient of 0-50% of eluent D in eluent C in 30 min at a flow rate of 1 mL/min (eluent C = 0.1% formic acid in H_2O; eluent D = 0.1% formic acid in CH_3CN/H_2O: 4/1 by vol). The column eluate was monitored by UV at 215 nm and by evaporative light scattering (ELS, waters 2424). The peptide masses were measured by on-line LC–MS: Ionization mode: ES+, m/z range 350–2040, capillary voltage 3 kV, cone voltage 30 V, extractor voltage 3 V, RF lens 0.2 V, source temperature 120 °C, dessolvation temperature 350 °C.

MALDI-TOF mass spectra were recorded with a Bruker Autoflex Speed using alpha-cyano-4-hydroxycinnaminic acid, sinapinic acid or 2,5-dihydroxybenzoic acid (DHB) as matrix. The observed m/z corresponded to the monoisotopic ions, unless otherwise stated.

Purifications

Preparative reverse phase HPLC of crude peptides were performed with a preparative HPLC Waters system using a reverse phase XBridge BEH300 Prep C18 column (5 µm, 300 Å, 10 × 250 mm) or C3Zorbax column (300SB-C3, 5 µm, 9.4 × 250 mm) and appropriate linear gradient of increasing concentration of eluent B in eluent A (flow rate of 6 mL min^{-1}, detection at 215 nm). Selected fractions were combined, frozen and lyophilized.

Synthesis of peptide hydrazides

Synthesis of Ac-ALKEPVHGVSGSA-NHNH$_2$

The synthesis was performed on a 0.1 mmol scale. The peptide was cleaved from the solid support and deprotected using a mixture of TFA/water/TIS 92.5/2.5/5 v/v/v for 1 h and then precipitated in 200 mL of ice-cold Et_2O/heptane 1/1 v/v. The peptide was solubilized in water, frozen and lyophilized (188 mg). The RP-HPLC purification was performed using a C3 zorbax column (50 °C, 215 nm, 6 mL min^{-1}, eluent A = water containing 0.1% v/v TFA, eluent B = CH_3CN/water 4/1 v/v containing 0.1% v/v of TFA, 0 to 10% B in 5 min, then 10 to 40% B in 60 min). 34 mg of crude peptide furnished 25 mg (84%) of purified peptide hydrazide.

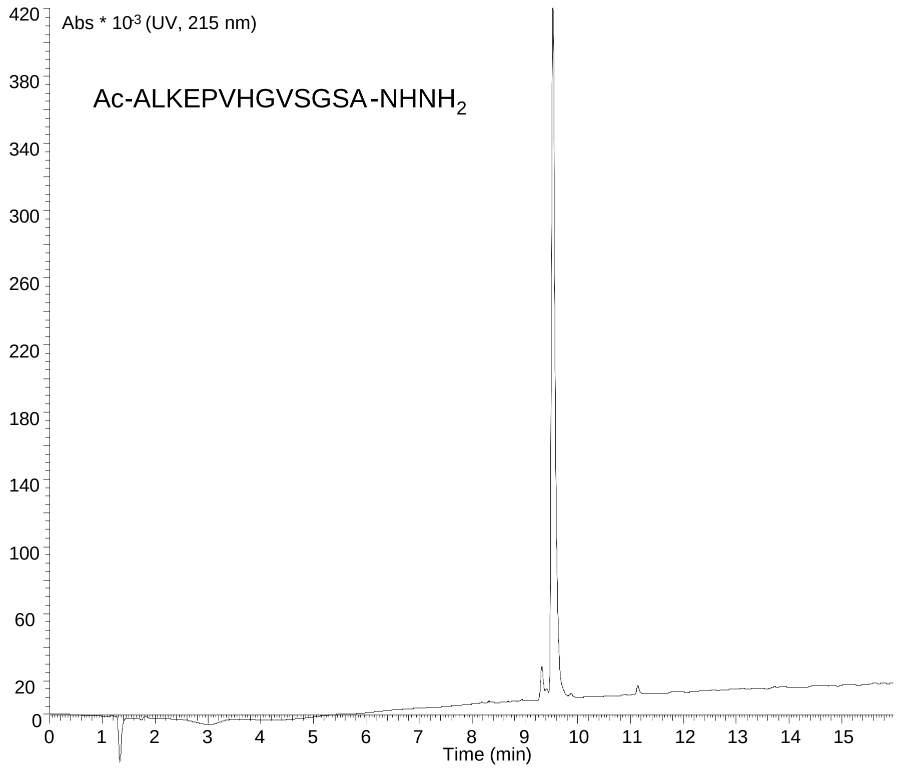

Supplementary Figure 1. UPLC-MS analysis of peptide hydrazide Ac-ALKEPVHGVSGSA-NHNH$_2$. LC trace. Eluent A 0.1% TFA in water, eluent B 0.1% TFA in CH_3CN. SB C3 (1.8 µm, 3.0 × 100 mm) column, gradient 0-40% B in 15 min, 0.4 mL min^{-1}, detection at 215 nm).

A)

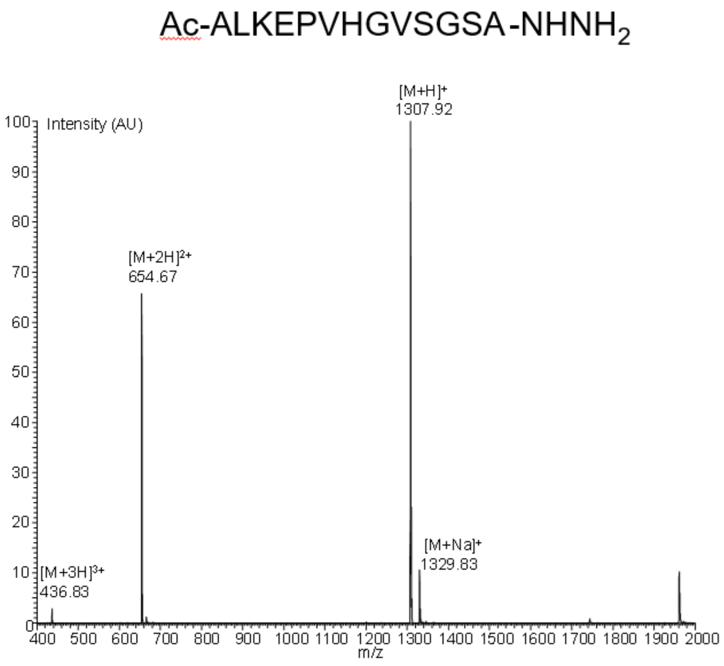

B)

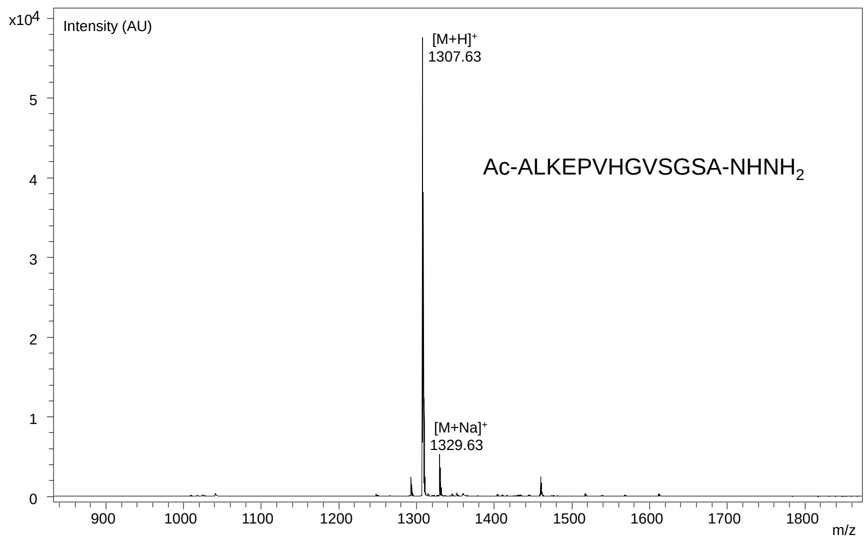

Supplementary Figure 2. Analysis of peptide hydrazide Ac-ALKEPVHGVSGSA-$NHNH_2$ by mass spectrometry. A) MS trace of peptide hydrazide Ac-ALKEPVHGVSGSA-$NHNH_2$ from LC-MS. $[M+H]^+$ m/z calcd. (monoisotopic) 1307.71, obs. 1307.50, $[M+2H]^{2+}$ m/z calcd. (av.) 654.74, obs. 654.42. B) MALDI-TOF analysis of peptide hydrazide Ac-ALKEPVHGVSGSA-$NHNH_2$. Matrix alpha-cyano-4-hydroxycinnaminic acid, positive detection mode, $[M+H]^+$ m/z calcd. (monoisotopic) 1307.71, found 1307.63.

Synthesis of Ac-ALKEPVHGVSGpSA-NHNH$_2$

The synthesis was performed on a 0.1 mmol scale. The peptide was cleaved from the solid support and deprotected using a mixture of TFA/water/TIS 92.5/2.5/5 v/v/v for 1 h 30 and then precipitated in 200 mL of ice-cold Et_2O/heptane 1/1 v/v. The peptide was solubilized in water, frozen and lyophilized (202 mg). The RP-HPLC purification was performed using a C3 zorbax column (50 °C, 215 nm, 6 mL min^{-1}, eluent A = water containing 0.1% v/v TFA, eluent B = CH_3CN/water 4/1 v/v containing 0.1% v/v of TFA, 0 to 20% B in 60 min). 35.3 mg of crude peptide hydrazide furnished 19.6 mg (55%) of purified peptide hydrazide.

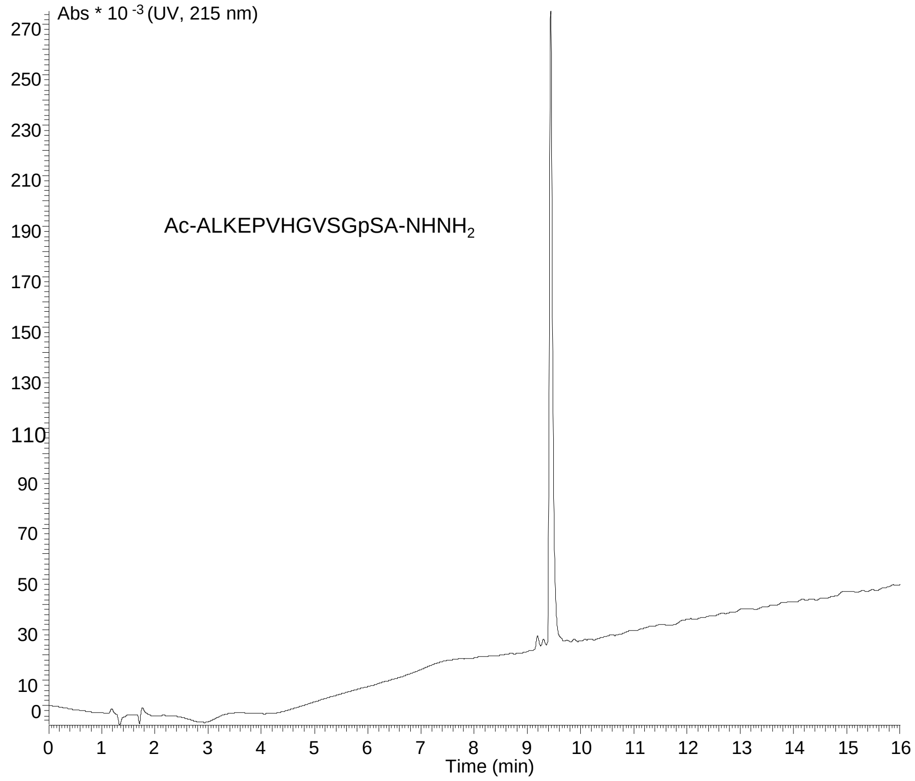

Supplementary Figure 3. UPLC-MS analysis of peptide hydrazide Ac-ALKEPVHGVSGpSA-NHNH$_2$. LC trace: Eluent A 0.1% TFA in water, eluent B 0.1% TFA in CH_3CN. SB C3 (1.8 µm, 3.0 × 100 mm) column, gradient 0-40% B in 15 min, 0.4 mL min^{-1}, detection at 215 nm).

A)

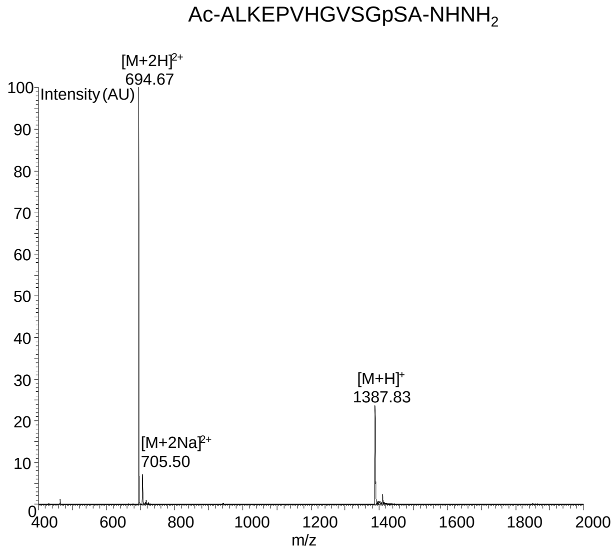

B)

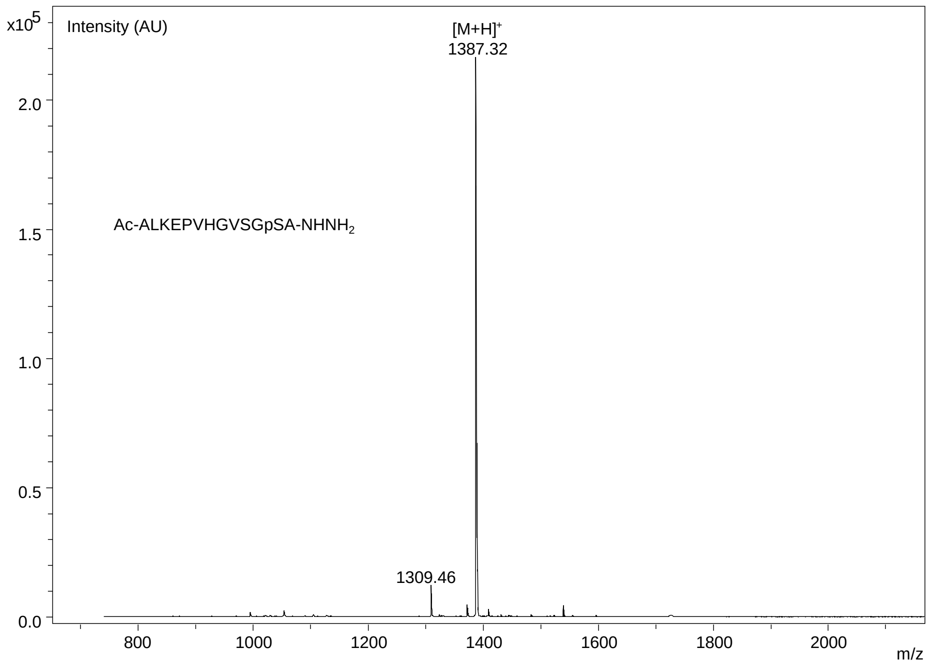

Supplementary Figure 4. Analysis of peptide hydrazide Ac-ALKEPVHGVSGpSA-$NHNH_2$ by mass spectrometry. A) MS trace of peptide hydrazide Ac-ALKEPVHGVSGpSA-$NHNH_2$ from LC-MS. $[M+H]^+$ m/z calcd. (monoisotopic) 1387.67, obs. 1387.83, $[M+2H]^{2+}$ m/z calcd. (av.) 694.72, obs. 694.67. B) MALDI-TOF analysis of peptide hydrazide Ac-ALKEPVHGVSGpSA-$NHNH_2$. Matrix 2,5-dihydroxybenzoic acid, positive detection mode, $[M+H]^+$ m/z calcd. (monoisotopic) 1387.67, found 1387.32.

Synthesis of Ac-ALKEPVHGVpSGSA-NHNH$_2$

The synthesis was performed on a 0.1 mmol scale. The peptide was cleaved from the solid support and deprotected using a mixture of TFA/water/TIS 92.5/2.5/5 v/v/v for 1 h 30 and then precipitated in 200 mL of ice-cold Et_2O/heptane 1/1 v/v. The peptide was solubilized in water, frozen and lyophilized (146 mg, 85%). The peptide was used directly in the next step.

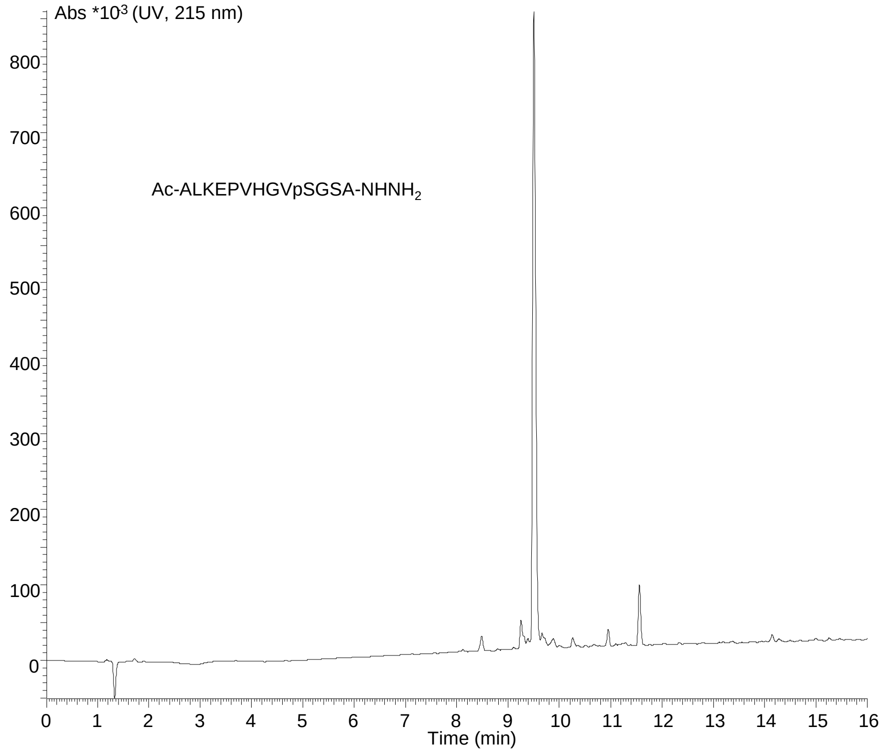

Supplementary Figure 5. UPLC-MS analysis of crude peptide hydrazide Ac-ALKEPVHGVpSGSA-NHNH$_2$. LC trace: Eluent A 0.1% TFA in water, eluent B 0.1% TFA in CH_3CN. SB C3 (1.8 µm, 3.0 × 100 mm) column, gradient 0-40% B in 15 min, 0.4 mL min^{-1}, detection at 215 nm).

A)

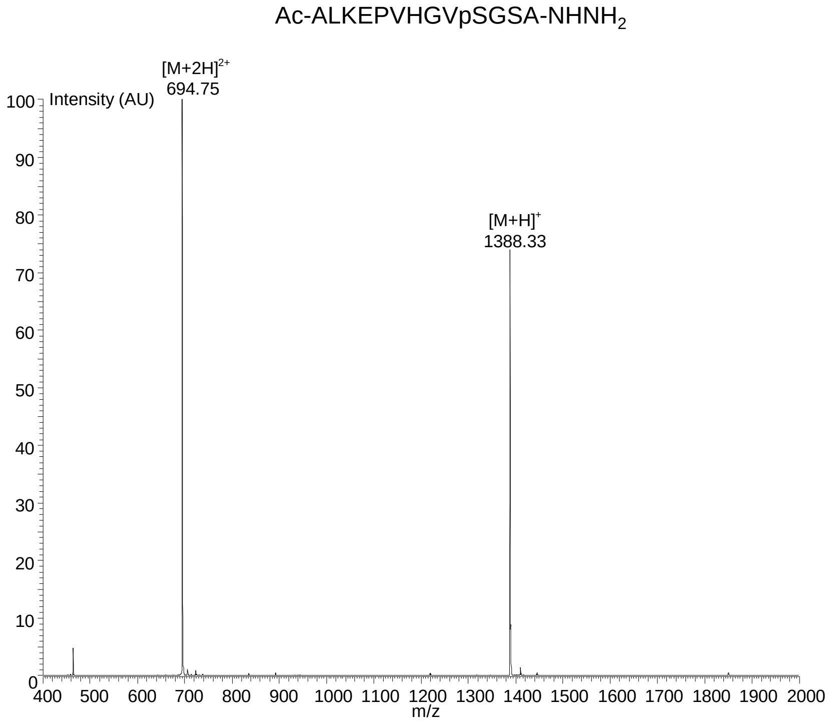

B)

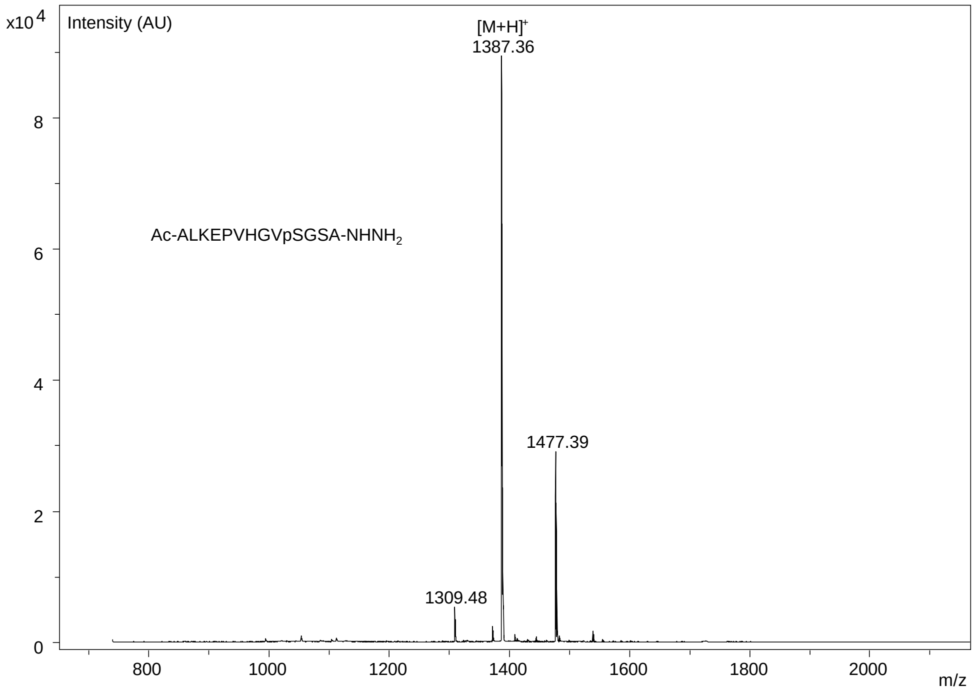

Supplementary Figure 6. MS analysis of peptide hydrazide Ac-ALKEPVHGVpSGSA-$NHNH_2$ A) MS trace of crude peptide hydrazide Ac-ALKEPVHGVpSGSA-$NHNH_2$ from UPLC-MS analysis. $[M+H]^+$ m/z calcd. (av.) 1388.43, obs.1388.33, $[M+2H]^{2+}$ m/z calcd. (av.) 694.72, obs. 694.75. B) MALDI-TOF analysis of peptide hydrazide Ac-ALKEPVHGVpSGSA-$NHNH_2$.

Matrix 2,5-dihydroxybenzoic acid, positive detection mode, $[M+H]^+$ m/z calcd. (monoisotopic) 1387.67, found 1387.36.

Synthesis of Ac-ALKEPVHGVpSGpSA-$NHNH_2$

The synthesis was performed on a 0.1 mmol scale. The peptide was cleaved from the solid support and deprotected using a mixture of TFA/water/TIS 92.5/2.5/5 v/v/v for 2 h and then precipitated in 200 mL of ice-cold Et_2O/heptane 1/1 v/v. The peptide was solubilized in water, frozen and lyophilized (177 mg). The RP-HPLC purification was performed using a C3 zorbax column (50 °C, 215 nm, 6 mL min^{-1}, eluent A = water containing 0.1% v/v TFA, eluent B = CH_3CN/water 4/1 v/v containing 0.1% v/v of TFA, 0 to 20% B in 60 min). 31 mg of crude peptide hydrazide furnished 14.6 mg (47%) of purified peptide hydrazide.

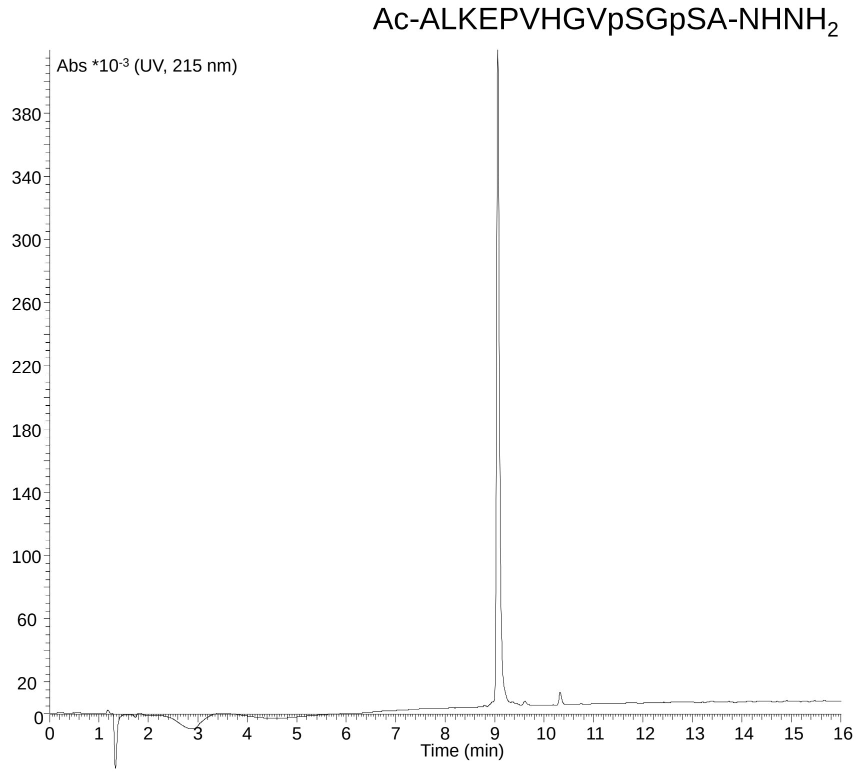

Supplementary Figure 7. UPLC-MS analysis of peptide hydrazide Ac-ALKEPVHGVpSGpSA-$NHNH_2$. LC trace: Eluent A 0.1% TFA in water, eluent B 0.1% TFA in CH_3CN. SB C3 (1.8 µm, 3.0 × 100 mm) column, gradient 0-40% B in 15 min, 0.4 mL min^{-1}, detection at 215 nm).

A)

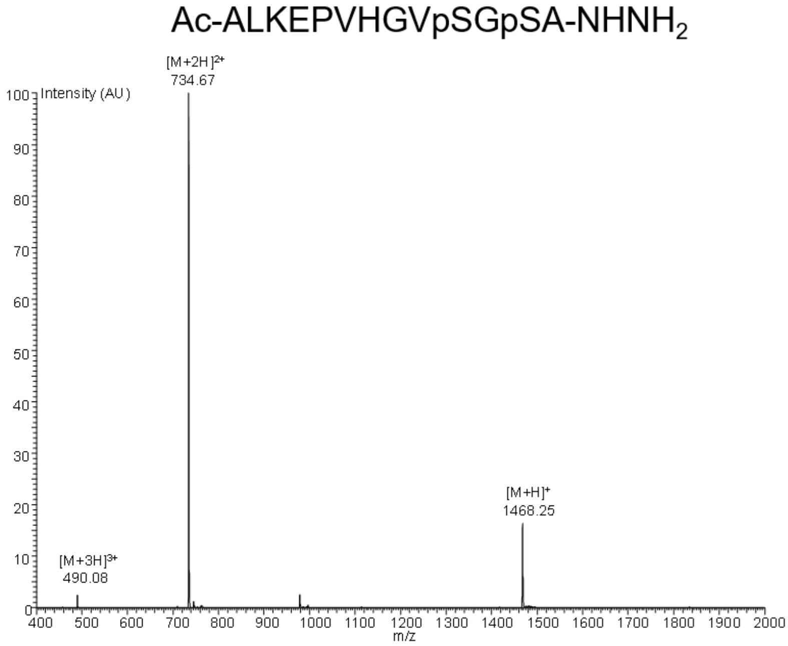

B)

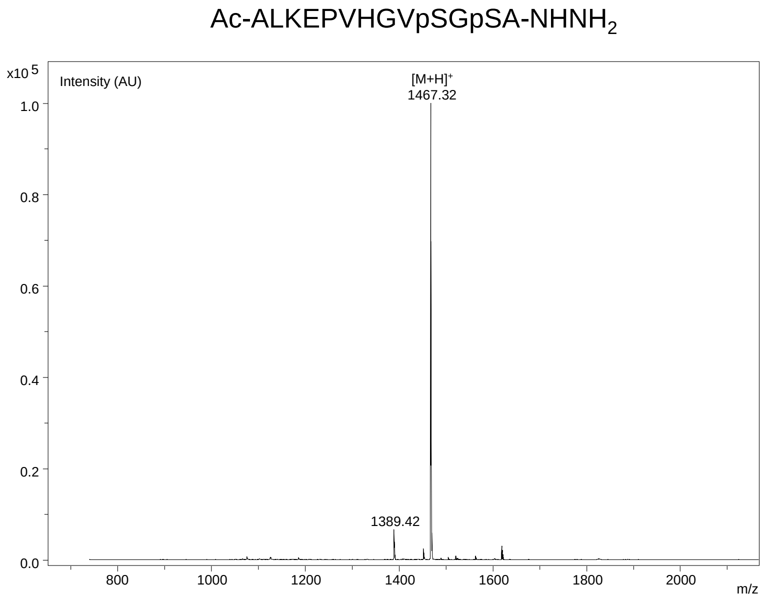

Supplementary Figure 8. Analysis of peptide hydrazide Ac-ALKEPVHGVpSGpSA-$NHNH_2$ by mass spectrometry. A) MS trace of peptide hydrazide Ac-ALKEPVHGVpSGpSA-$NHNH_2$ from UPLC-MS analysis. $[M+H]^+$ m/z calcd. (av.) 1468.43, obs. 1468.25, $[M+2H]^{2+}$ m/z calcd. (av.) 734.72, obs. 734.67, $[M+3H]^{3+}$ m/z calcd. (av.) 490.14, obs. 490.08. B) MALDI-TOF analysis of peptide hydrazide Ac-ALKEPVHGVpSGpSA-$NHNH_2$. Matrix 2,5-dihydroxybenzoic acid, positive detection mode, $[M+H]^+$ m/z calcd. (monoisotopic) 1467.63, found 1467.32.

Synthesis of Ac-ALKEPVHGVpSpSpSA-NHNH₂

The synthesis was performed on a 0.05 mmol scale. The peptide was cleaved from the solid support and deprotected using a mixture of TFA/water/TIS 92.5/2.5/5 v/v/v for 3 h and then precipitated in 100 mL of ice-cold Et_2O/heptane 1/1 v/v. The peptide was solubilized in water containing 0.1% TFA, frozen and lyophilized (37.2 mg).

The RP-HPLC purification was performed using a C18 XBridge column (65 °C, 215 nm, 6 mL min^{-1}, eluent A = water containing 0.1% v/v TFA, eluent B = CH_3CN containing 0.1% v/v of TFA, 0 to 10% B in 5 min, then 10 to 50% B in 80 min). 36.2 mg of crude peptide furnished 2.2 mg (6.1%) of purified peptide hydrazide.

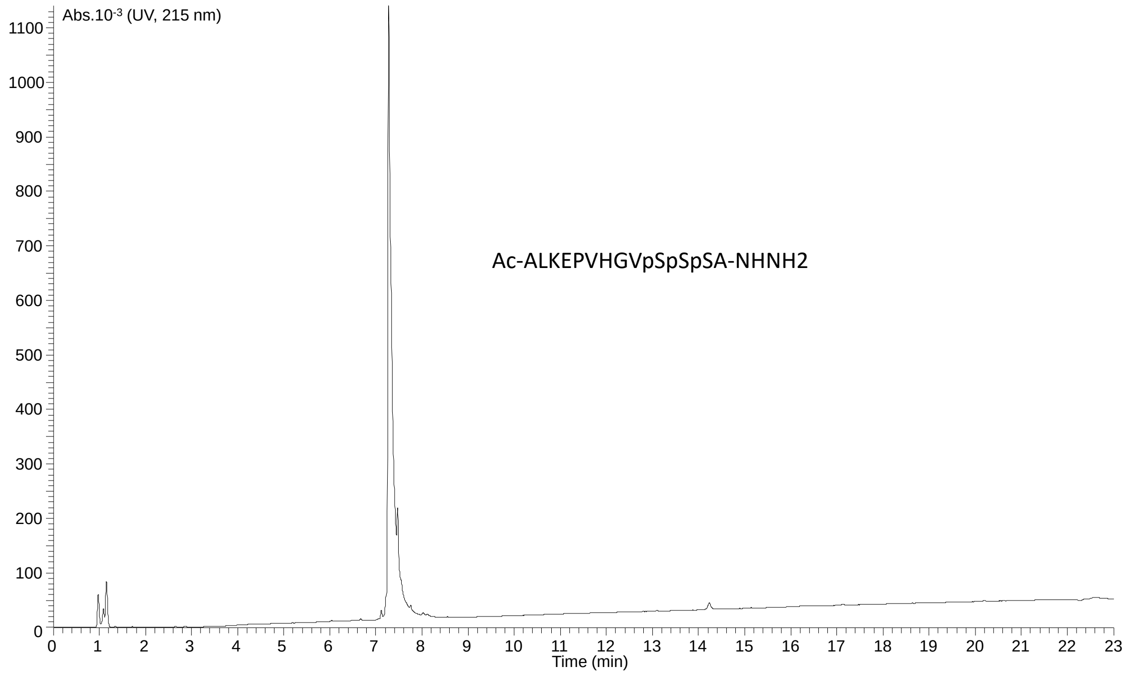

Supplementary Figure 9. LC-MS analysis of purified peptide hydrazide Ac-ALKEPVHGVpSpSpSA-$NHNH_2$. LC trace. Eluent A 0.1% TFA in water, eluent B 0.1% TFA in CH_3CN. C18 BEH 300 Å (1.7 µm, 2.1 × 100 mm) column, gradient 0-70% B in 20 min, 0.4 mL min^{-1}, detection at 215 nm).

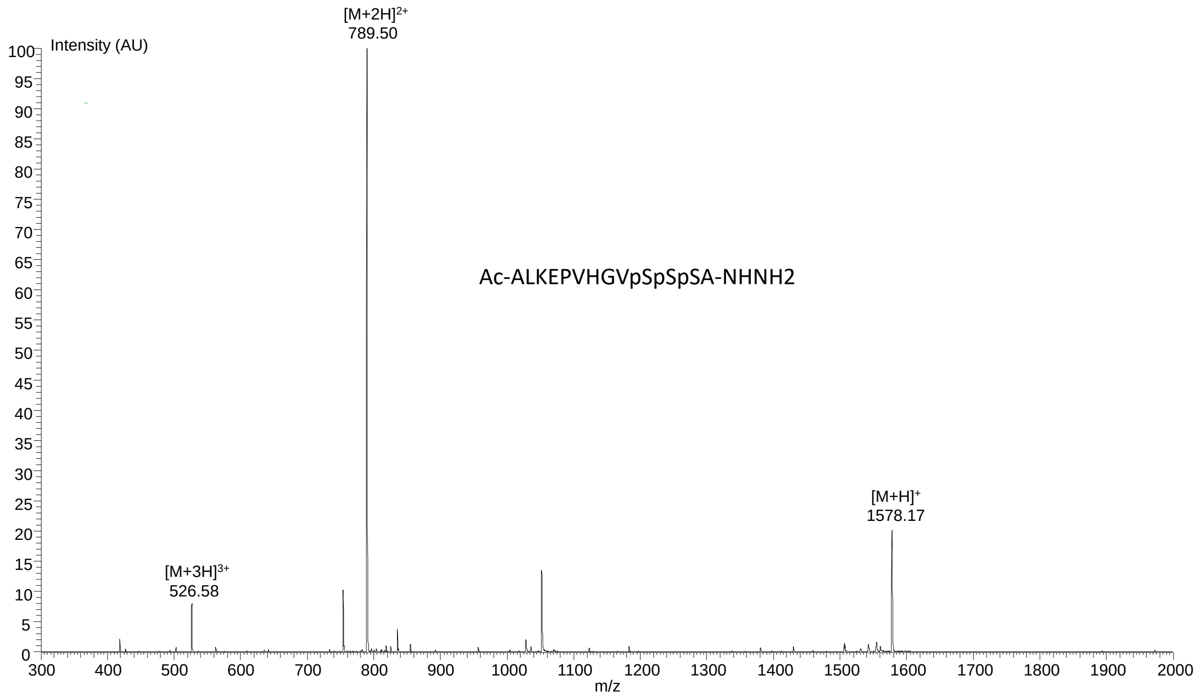

Supplementary Figure 10. MS trace of purified peptide hydrazide Ac-ALKEPVHGVpSpSpSA-$NHNH_2$ from LC-MS analysis. $[M+H]^+$ m/z calcd. (av.) 1578.44, obs. 1578.17, $[M+2H]^{2+}$ m/z calcd. (av.) 789.72, obs. 789.50, $[M+3H]^{3+}$ m/z calcd. (av.) 526.81, obs. 526.58.

Synthesis of Ac-GSGSGSGKPIPNPLLGLDSTASGSGSGpSGpSA-$NHNH_2$

The sequence of this peptide is derived from V5 peptide tag.

The synthesis was performed on a 0.1 mmol scale. Fmoc-Ala-Ser(ψ(Me, Me)Pro)-OH was introduced in the sequence in place of Ala-Ser (GSGSGSGKPIPNPLLGLDST<u>AS</u>GSGSGpSGpSA). The pseudoproline dipeptide (2 equiv, 87.7 mg, 0.2 mmol) was coupled twice manually using HATU (1.9 equiv, 72.2 mg, 0.190 mmol) and DIEA (4 eq, 69.7 µL, 0.400 mmol) dissolved in the minimal volume of DMF during 1 h. After coupling, a capping step was performed with 10% v/v acetic anhydride and 5% v/v DIEA in DMF (5 and 15 min).

The peptide was cleaved from the solid support and deprotected using a mixture of TFA/water/TIS 92.5/2.5/5 v/v/v for 2 h 20 min and then precipitated in 200 mL of ice-cold Et_2O/heptane 1/1 v/v. The peptide was solubilized in water containing 0.1 % TFA and lyophilized (93 mg). The RP-HPLC purification was performed using on a C18 XBridge column (50 °C, 215 nm, 6 mL min^{-1}, eluent A = water containing 0.1% v/v TFA, eluent B = CH_3CN containing 0.1% v/v of TFA, 0 to 35% B in 50 min) and furnished 16.3 mg (5%) of purified peptide hydrazide.

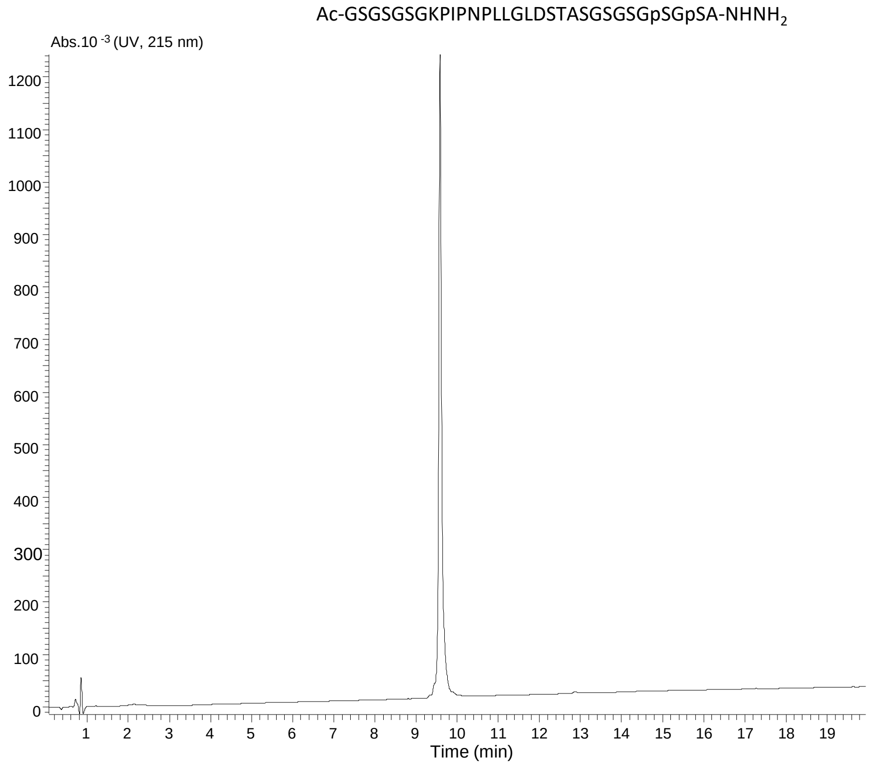

Supplementary Figure 11. UPLC-MS analysis of peptide hydrazide Ac-GSGSGSGKPIPNPLLGLDSTASGSGSGpSGpSA-$NHNH_2$. LC trace: Eluent A 0.1% TFA in water, eluent B 0.1% TFA in CH_3CN. BEH C18 (300 Å, 1.7 µm, 2.1 × 100 mm) column, gradient 0-70% B in 20 min, 0.4 mL min^{-1}, detection at 215 nm).

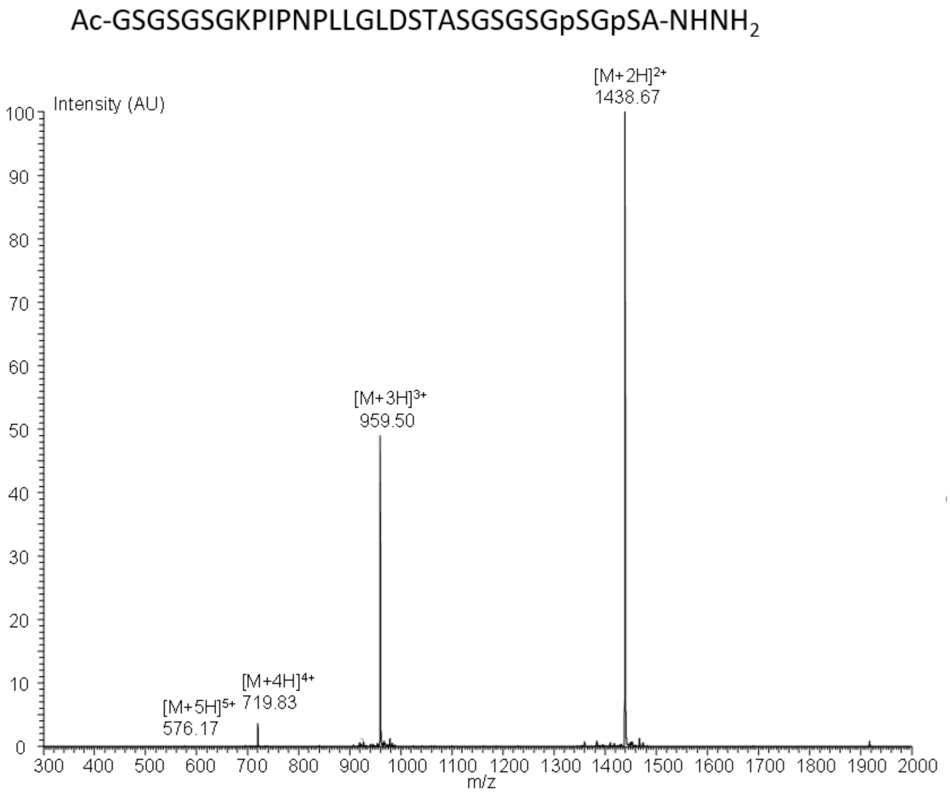

Supplementary Figure 12. MS trace of peptide hydrazide Ac-GSGSGSGKPIPNPLLGLDSTASGSGSGpSGpSA-$NHNH_2$ from UPLC-MS analysis. $[M+2H]^{2+}$ m/z calcd. (av.) 1438.92, obs. 1438.67, $[M+3H]^{3+}$ m/z calcd. (av.) 959.61, obs. 959.50, $[M+4H]^{4+}$ m/z calcd. (av.) 719.96, obs. 719.83, $[M+5H]^{5+}$ m/z calcd. (av.) 576.17, obs. 576.17.

Synthesis of peptide thioesters

The peptide thioesters used in this work and derived from peptide hydrazides and MPAA were prepared using protocols adapted from published procedures.[1, 2]

Synthesis of MPAA thioester peptides from peptide hydrazides

Synthesis of Ac-ALKEPVHGVpSGpSA-MPAA ***1a***

The RP-HPLC purification was performed using a C18 XBridge column (50 °C, 215 nm, 6 mL min^{-1}, eluent A = water containing 0.1% v/v TFA, eluent B = CH_3CN/water 4/1 v/v containing 0.1% v/v of TFA, 0 to 20% B in 5 min, then 20 to 50% B in 90 min). 18.3 mg of purified peptide hydrazide furnished 15.7 mg (75%) of purified peptide thioester **1a**.

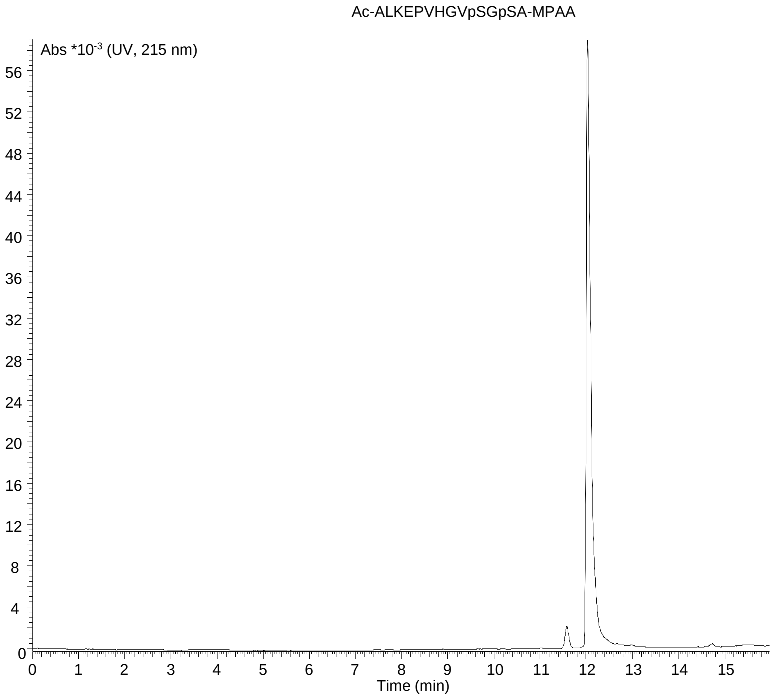

Supplementary Figure 13. UPLC-MS analysis of peptide thioester **1a**. LC trace: Eluent A 0.1% TFA in water, eluent B 0.1% TFA in CH_3CN. SB C3 (1.8 µm, 3.0 × 100 mm) column, gradient 0-40% B in 15 min, 0.4 mL min^{-1}, detection at 215 nm).

A)

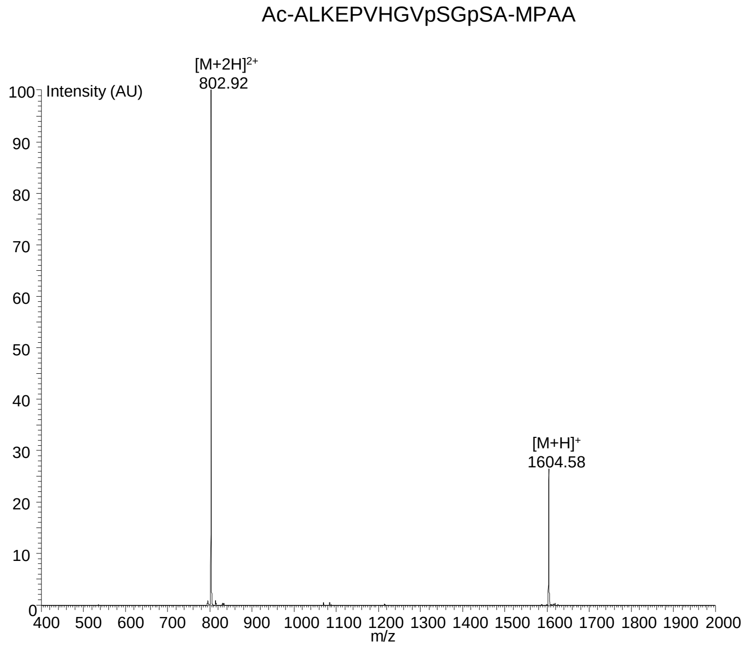

B)

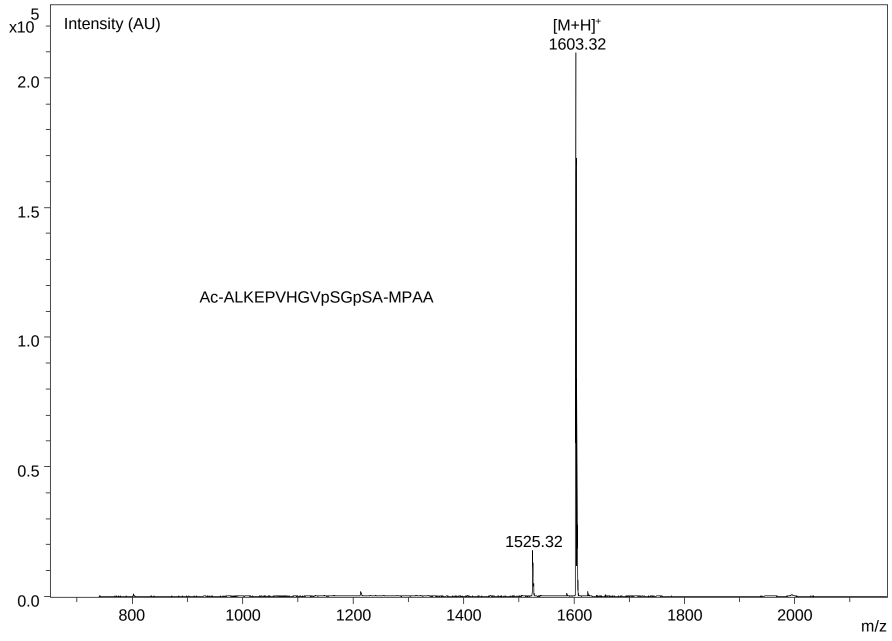

Supplementary Figure 14. Analysis of peptide thioester **1a** by mass spectrometry. A) MS trace of peptide thioester **1a** from LC-MS analysis. $[M+H]^+$ m/z calcd. (av.) 1604.60, obs. 1604.58, $[M+2H]^{2+}$ m/z calcd. (av.) 802.80, obs. 802.92. B) MALDI-TOF analysis of peptide thioester **1a**. Matrix 2,5-dihydroxybenzoic acid, positive detection mode, $[M+H]^+$ m/z calcd. (monoisotopic) 1603.63, found 1603.32.

Synthesis of Ac-ALKEPVHGVSGpSA-MPAA ***1b***

The RP-HPLC purification was performed using a C18 XBridge column (50 °C, 215 nm, 6 mL min^{-1}, eluent A = water containing 0.1% v/v TFA, eluent B = CH_3CN/water 4/1 v/v containing 0.1% v/v of TFA, 0 to 20% B in 5 min, then 20 to 50% B in 90 min). 10.9 mg of peptide hydrazide furnished 5.2 mg (47%) of purified peptide thioester **1b**.

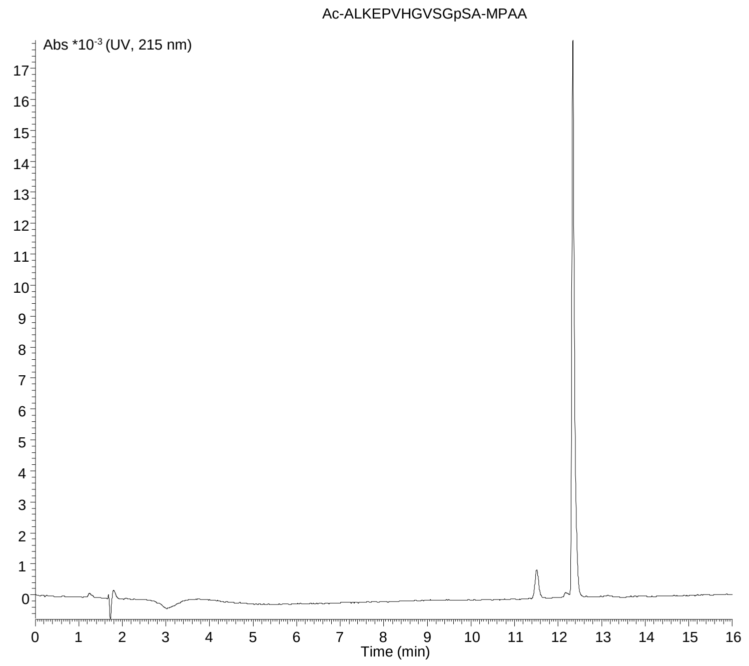

Supplementary Figure 15. UPLC-MS analysis of peptide thioester **1b.** LC trace: Eluent A 0.1% TFA in water, eluent B 0.1% TFA in CH_3CN. SB C3 (1.8 µm, 3.0 × 100 mm) column, gradient 0-40% B in 15 min, 0.4 mL min^{-1}, detection at 215 nm).

A)

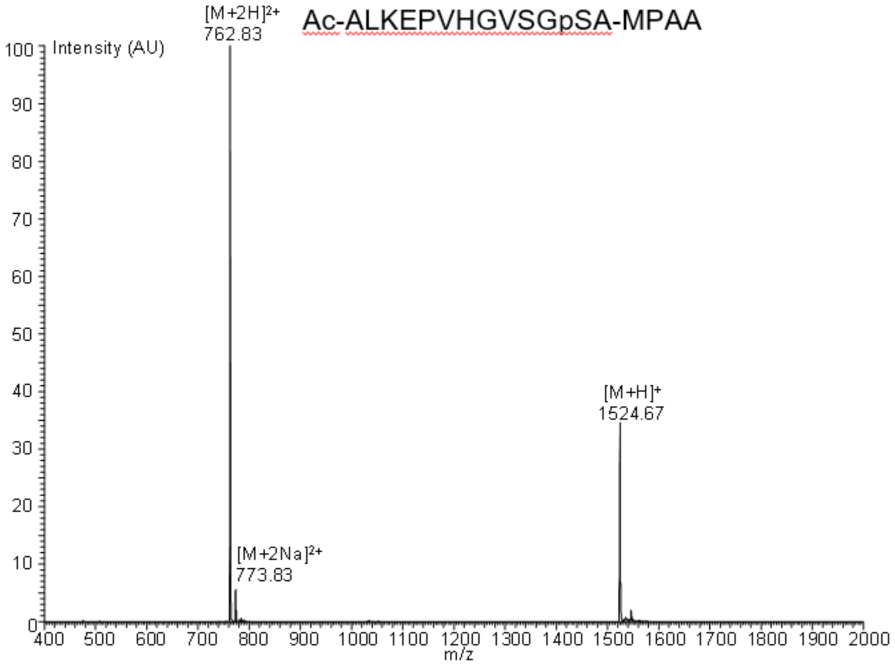

B)

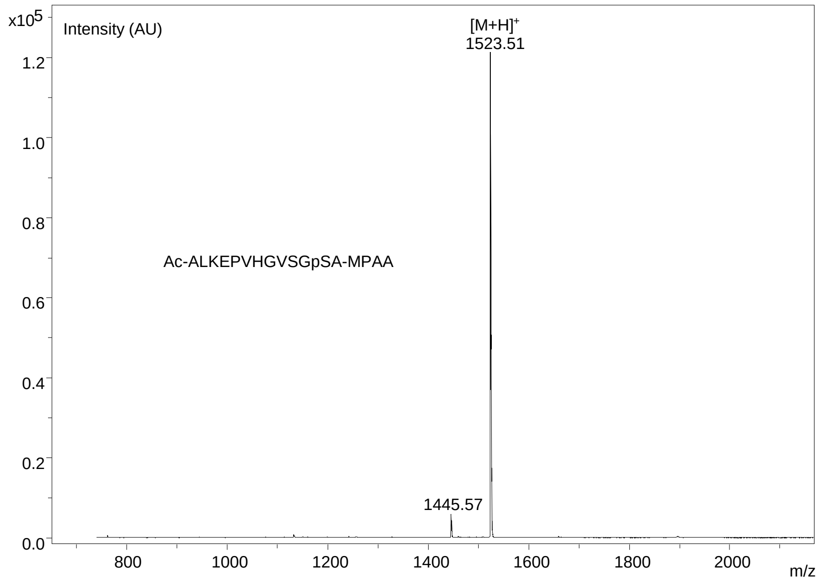

Supplementary Figure 16. Analysis of peptide thioester **1b** by mass spectrometry. A) MS trace of peptide thioester **1b** from LC-MS analysis. $[M+H]^+$ m/z calcd. (av.) 1524.62, obs. 1524.67, $[M+2H]^{2+}$ m/z calcd. (av.) 762.81, obs. 762.92. B) MALDI-TOF analysis of peptide thioester **1b.** Matrix 2,5-dihydroxybenzoic acid, positive detection mode, $[M+H]^+$ m/z calcd. (monoisotopic) 1523.66, found 1523.51.

Synthesis of Ac-ALKEPVHGVpSGSA-MPAA ***1c***

The RP-HPLC purification was performed using a C18 XBridge column (50 °C, 215 nm, 6 mL min^{-1}, eluent A = water containing 0.1% v/v TFA, eluent B = CH_3CN/water 4/1 v/v containing 0.1% v/v of TFA, 0 to 20% B in 5 min, then 20 to 50% B in 90 min). 10.6 mg of crude peptide hydrazide furnished 5 mg (47%) of purified peptide thioester **1c**.

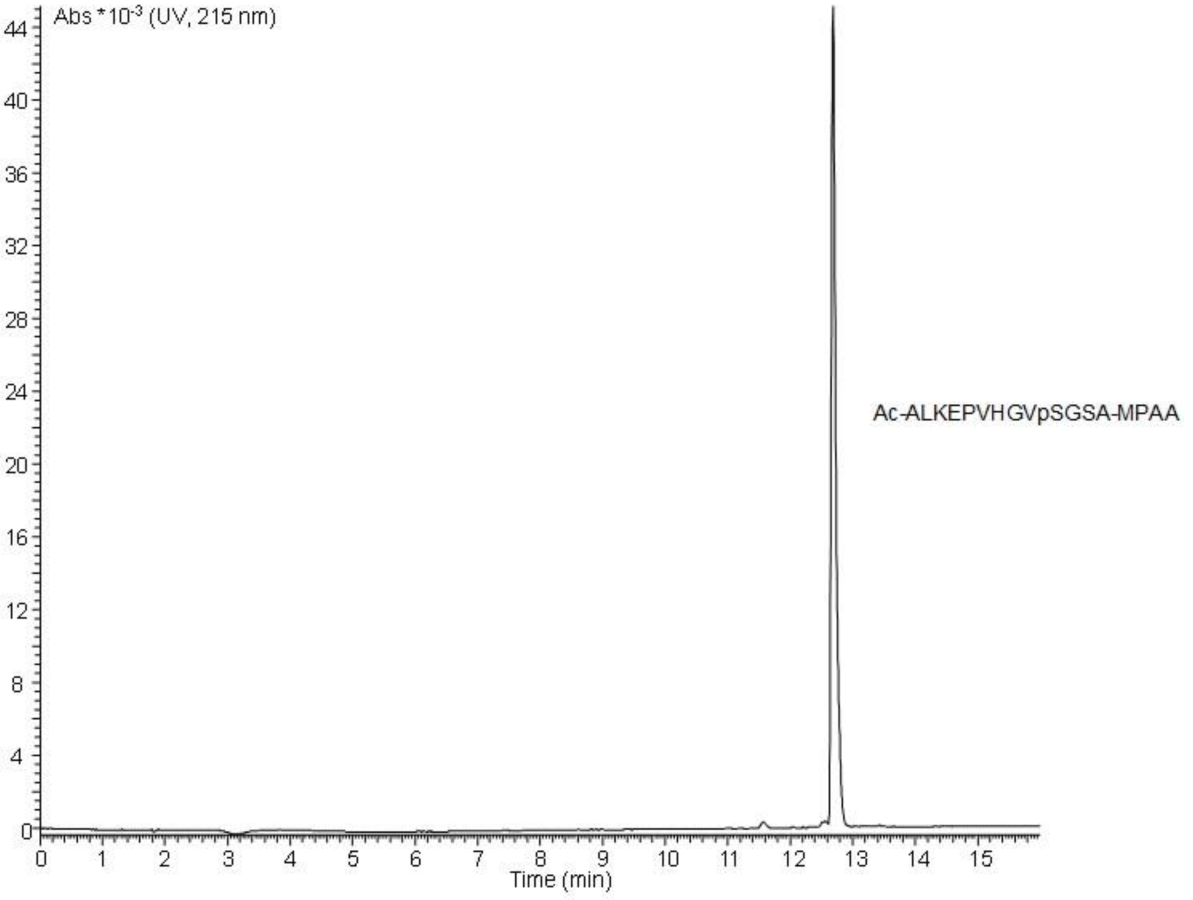

Supplementary Figure 17. UPLC-MS analysis of peptide thioester **1c**. LC trace. Eluent A 0.1% TFA in water, eluent B 0.1% TFA in CH_3CN. SB C3 (1.8 µm, 3.0 × 100 mm) column, gradient 0-40% B in 15 min, 0.4 mL min^{-1}, detection at 215 nm).

A)

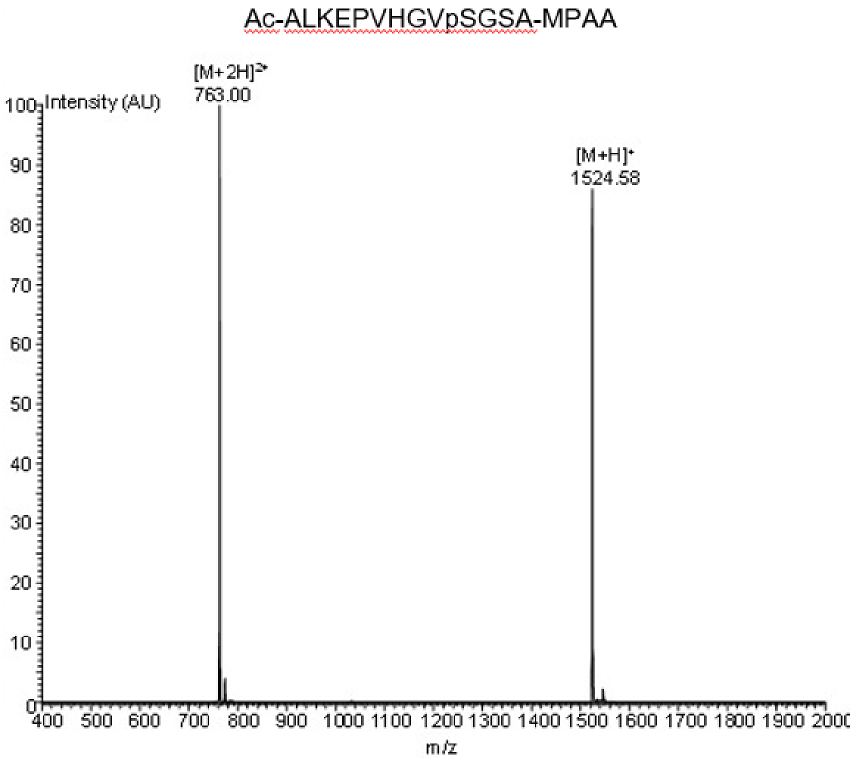

B)

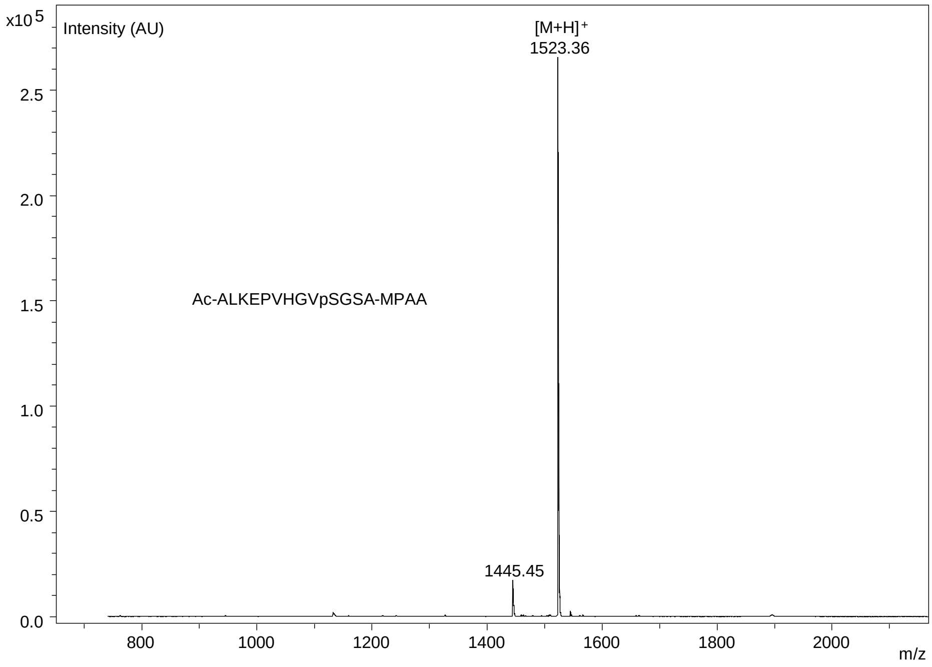

Supplementary Figure 18. Analysis of peptide thioester **1c** by mass spectrometry. A) MS trace of peptide thioester **1c** from LC-MS analysis. $[M+H]^+$ m/z calcd. (av.) 1524.62, obs. 1524.58, $[M+2H]^{2+}$ m/z calcd. (av.) 762.81, obs. 763.00. B) MALDI-TOF analysis of peptide thioester **1c**. Matrix 2,5-dihydroxybenzoic acid, positive detection mode, $[M+H]^+$ m/z calcd. (monoisotopic) 1523.66, found 1523.36.

Synthesis of Ac-ALKEPVHGVSGSA-MPAA ***1d***

The RP-HPLC purification was performed using a C18 XBridge column (50 °C, 215 nm, 6 mL min^{-1}, eluent A = water containing 0.1% v/v TFA, eluent B = CH_3CN/water 4/1 v/v containing 0.1% v/v of TFA, 0 to 20% B in 5 min, then 20 to 50% B in 90 min). 17.1 mg of peptide hydrazide furnished 10.8 mg (62%) of purified peptide thioester **1d**.

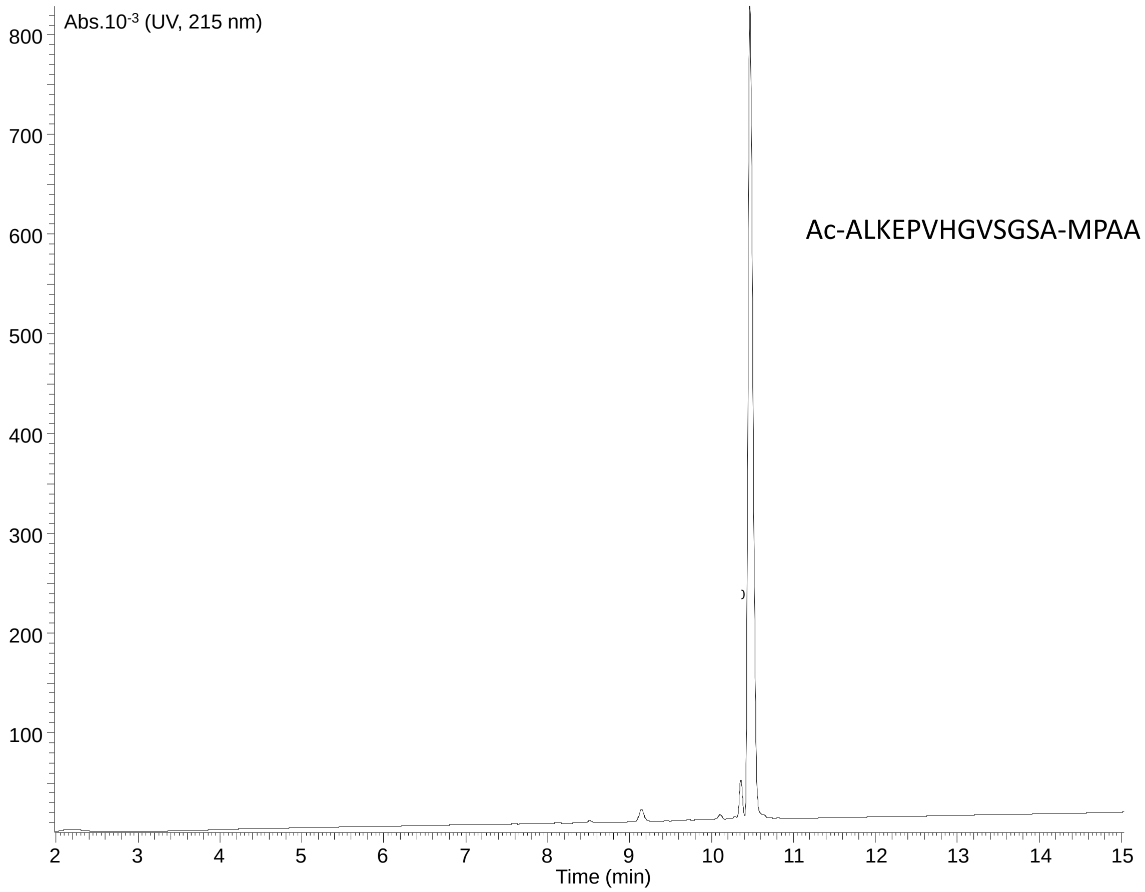

Supplementary Figure 19. UPLC-MS analysis of peptide thioester **1d**. LC trace: Eluent A 0.1% TFA in water, eluent B 0.1% TFA in CH_3CN. SB C3 (1.8 µm, 3.0 × 100 mm) column, gradient 0-40% B in 15 min, 0.4 mL min^{-1}, detection at 215 nm). MS trace: $[M+H]^+$ m/z calcd. (monoisotopic) 1443.69, obs. 1443.67, $[M+2H]^{2+}$ m/z calcd. (av.) 722.82, obs. 722.67.

A)

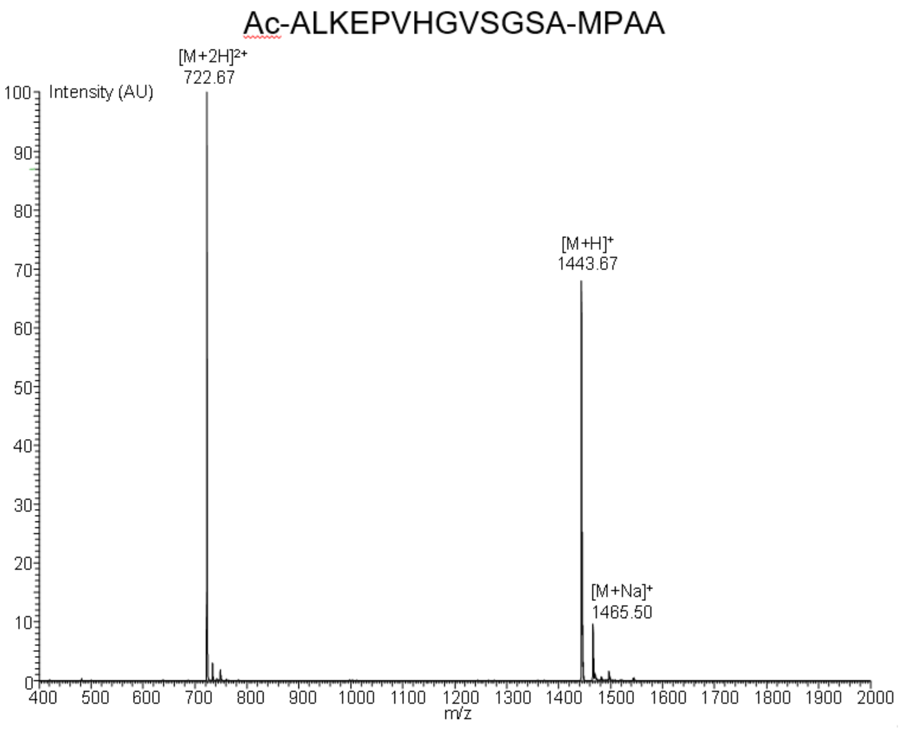

B)

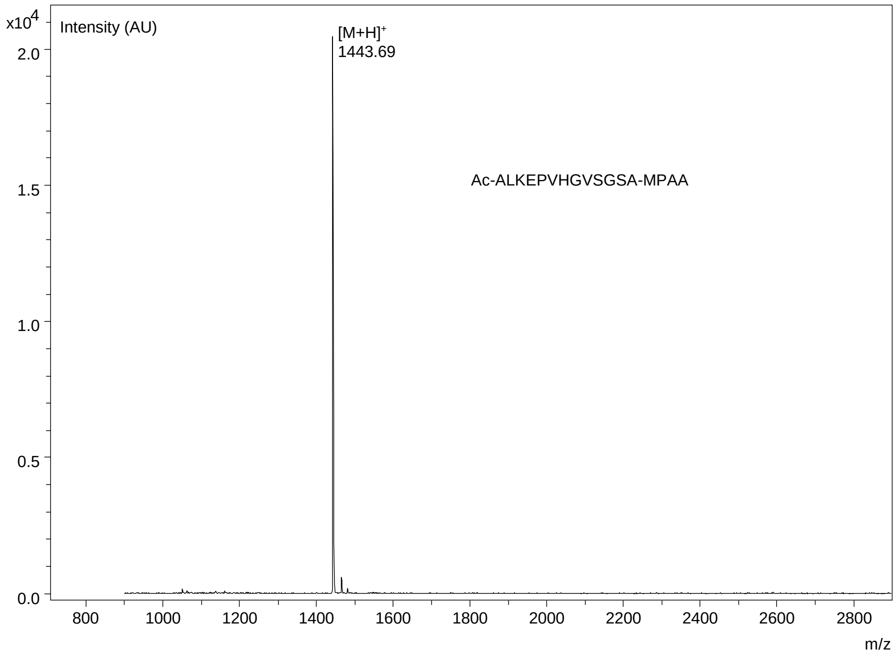

Supplementary Figure 20. Analysis of peptide thioester **1d** by mass spectrometry. A) MS trace of peptide thioester **1d** from LC-MS analysis. $[M+H]^+$ m/z calcd. (monoisotopic) 1443.69, obs. 1443.67, $[M+2H]^{2+}$ m/z calcd. (av.) 722.82, obs. 722.67. B) MALDI-TOF analysis of peptide thioester **1d**. Matrix 2,5-dihydroxybenzoic acid, positive detection mode, $[M+H]^+$ m/z calcd. (monoisotopic) 1443.69, found 1443.69.

Synthesis of Ac-ALKEPVHGVpSpSpSA-MPAA **1e**

The RP-HPLC purification was performed using a C18 XBridge column (50 °C, 215 nm, 6 mL min^{-1}, eluent A = water containing 0.1% v/v TFA, eluent B = CH_3CN containing 0.1% of TFA, 0 to 10% B in 5 min, then 10 to 30% B in 60 min). 2.2 mg of peptide hydrazide furnished 749 µg (34%) of purified peptide thioester **1e**.

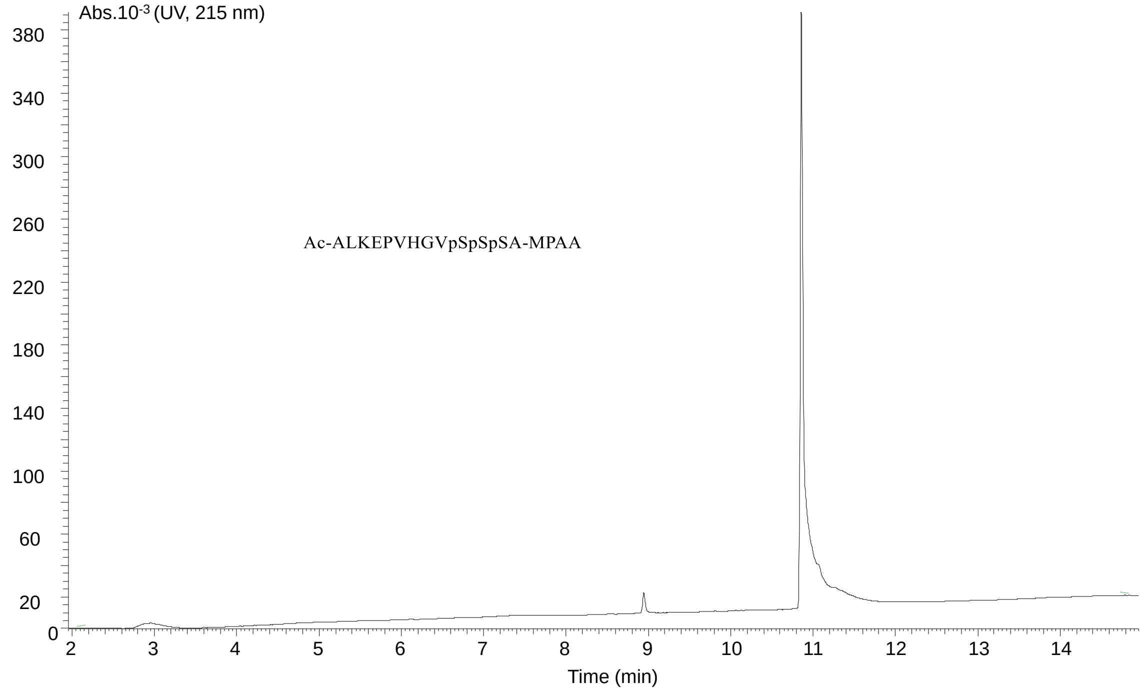

Supplementary Figure 21. LC-MS analysis of peptide thioester **1e**. LC trace. Eluent A 0.1% TFA in water, eluent B 0.1% TFA in CH_3CN. C18 BEH 300 Å (1.7 µm, 2.1 × 100 mm) column, gradient 0-40% B in 15 min, 0.4 mL min^{-1}, detection at 215 nm).

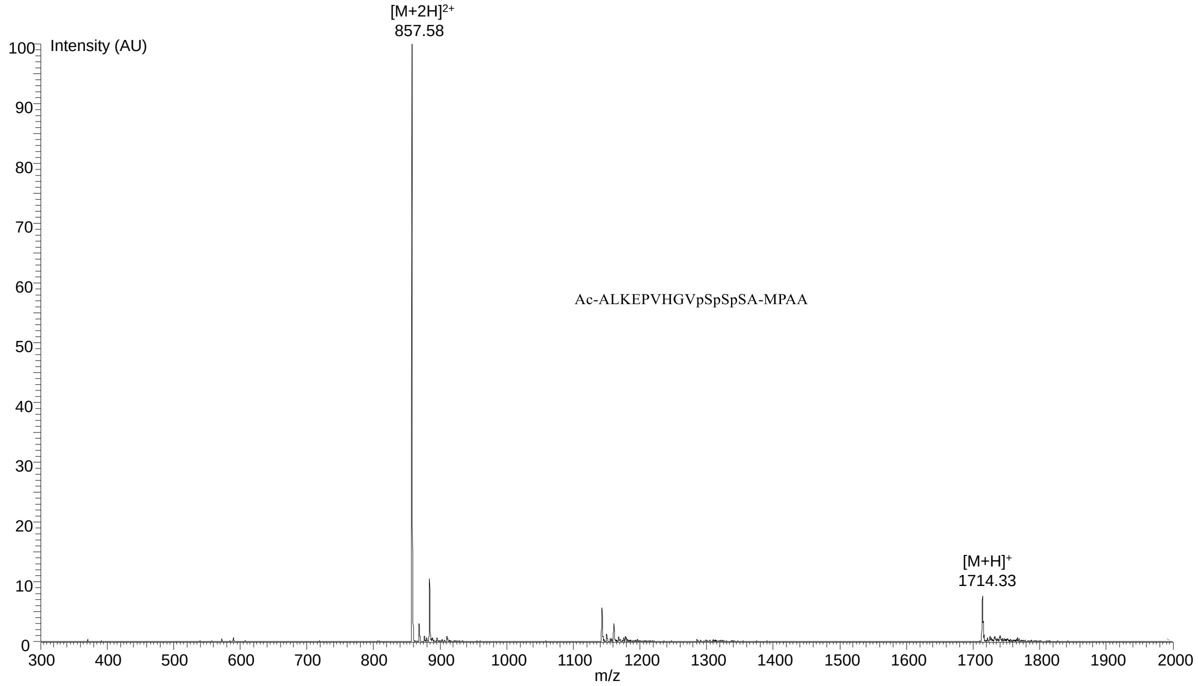

Supplementary Figure 22. MS trace of peptide thioester **1e** from LC-MS analysis. $[M+H]^+$ m/z calcd. (av.) 1714.60, obs. 1714.33, $[M+2H]^2$+ m/z calcd. (av.) 857.8, obs. 857.58.

*Synthesis of Ac-ALKEPVHGVEGEA-MPAA **1f***

Synthesis of Ac-ALKEPVHGVEGEA-$NHNH_2$: The synthesis was performed on a 0.05 mmol scale. The peptide was cleaved from the solid support and deprotected using a mixture of TFA/water/TIS 92.5/2.5/5 v/v/v for 1 h and then precipitated in 100 mL of ice-cold Et_2O/heptane 1/1 v/v. The peptide was solubilized in water containing 0.1% TFA, frozen and lyophilized (71.5 mg). The crude peptide hydrazide was used directly in the next step.

The RP-HPLC purification of the crude peptide thioester **1f** was performed using a C18 XBridge column (50 °C, 215 nm, 6 mL min^{-1}, eluent A = water containing 0.1% v/v TFA, eluent B = CH_3CN containing 0.1% v/v of TFA, 0 to 15% B in 5 min, then 15 to 45% B in 60 min). 19.7 mg of crude peptide hydrazide furnished 7.9 mg (41%) of purified peptide thioester **1f**.

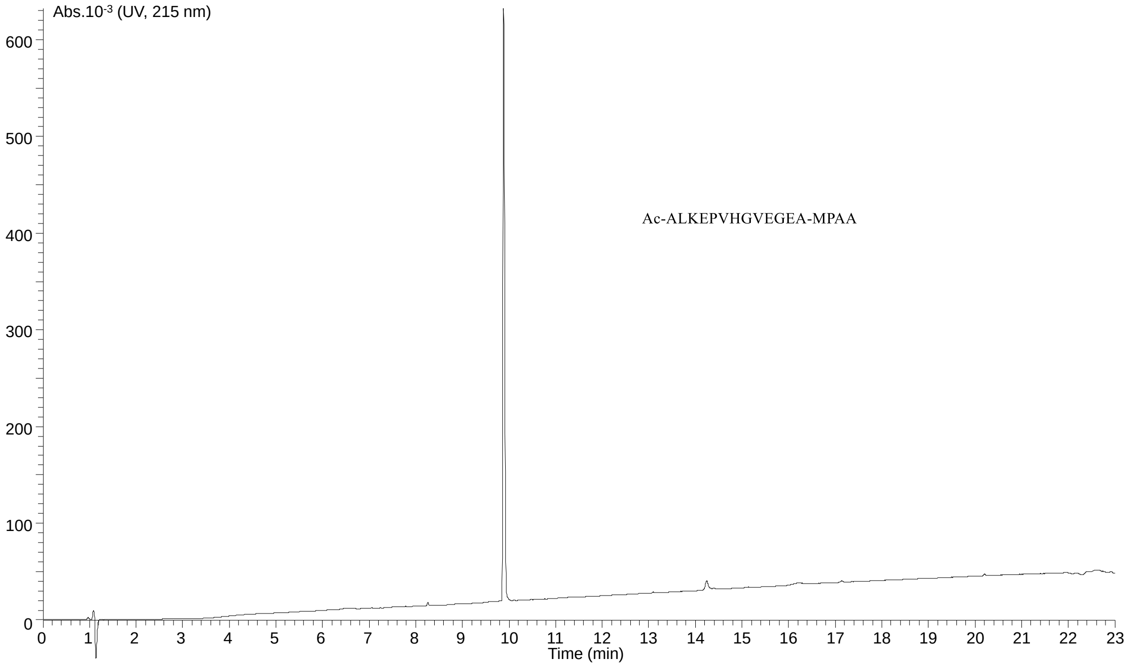

Supplementary Figure 23. UPLC analysis of peptide thioester **1f**. LC trace. Eluent A 0.1% TFA in water, eluent B 0.1% TFA in CH_3CN. C18 BEH 300 Å (1.7 µm, 2.1 × 100 mm) column, gradient 0-70% B in 20 min, 0.4 mL min^{-1}, detection at 215 nm.

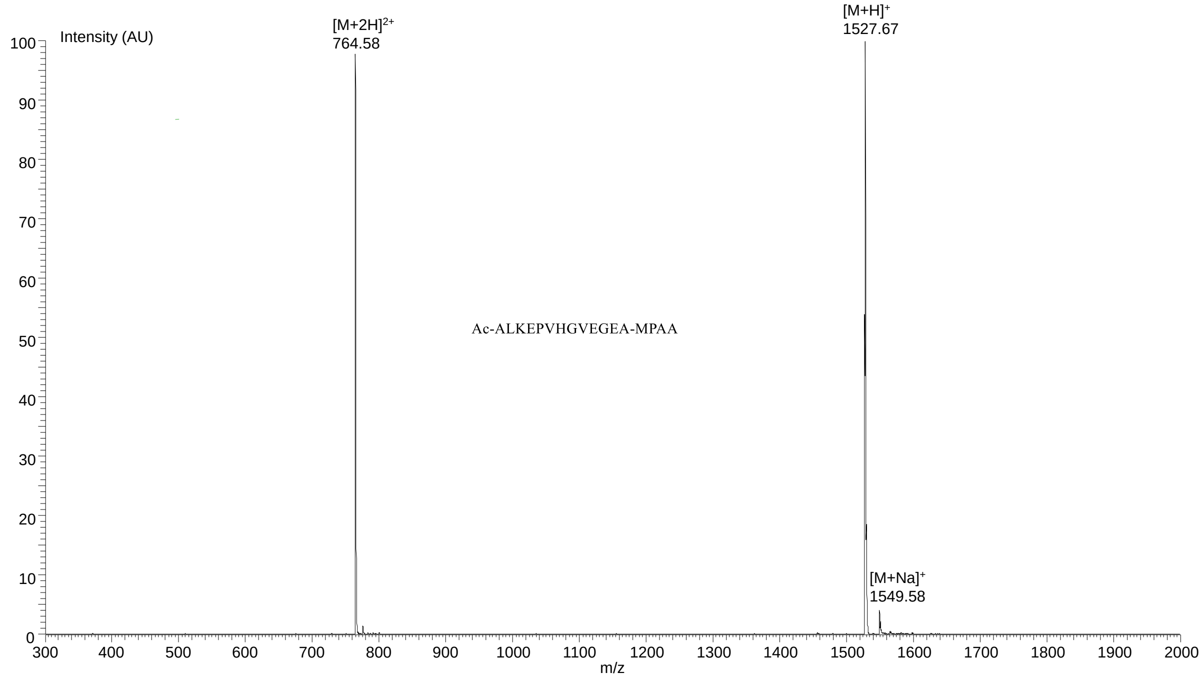

Supplementary Figure 24. MS trace of peptide thioester **1f** from UPLC-MS analysis. $[M+H]^+$ m/z calcd. (monoisotopic) 1527.71, obs. 1527.67, $[M+2H]^{2+}$ m/z calcd. (av.) 764.85, obs. 764.58.

*Synthesis of Ac-ALKEPVHGVEEGEEA-MPAA **1g***

Synthesis of Ac-ALKEPVHGVEEGEEA-$NHNH_2$: The synthesis was performed on a 0.05 mmol scale. The peptide was cleaved from the solid support and deprotected using a mixture of TFA/water/TIS 92.5/2.5/5 v/v/v for 1 h and then precipitated in 100 mL of ice-cold Et_2O/heptane 1/1 v/v. The peptide was solubilized in water containing 0.1% TFA, frozen and lyophilized (97.5 mg). The crude peptide hydrazide was used directly in the next step.

The RP-HPLC purification of the crude peptide thioester **1g** was performed using a C18 XBridge column (50 °C, 215 nm, 6 mL min^{-1}, eluent A = water containing 0.1% v/v TFA, eluent B = CH_3CN containing 0.1% v/v of TFA, 0 to 15% B in 5 min, then 15 to 45% B in 60 min). 19 mg of crude peptide hydrazide furnished 8.4 mg (45%) of purified peptide thioester **1g**.

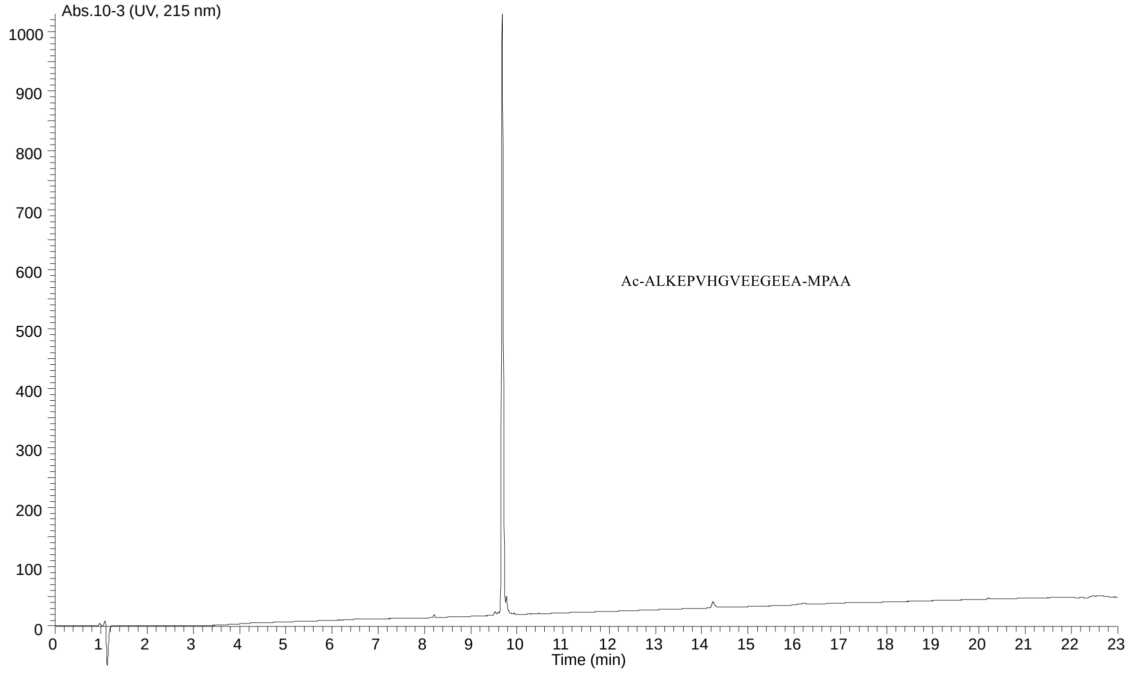

Supplementary Figure 25. UPLC analysis of peptide thioester **1g**. LC trace. Eluent A 0.1% TFA in water, eluent B 0.1% TFA in CH_3CN. C18 BEH 300 Å (1.7 µm, 2.1 × 100 mm) column, gradient 0-70% B in 20 min, 0.4 mL min^{-1}, detection at 215 nm).

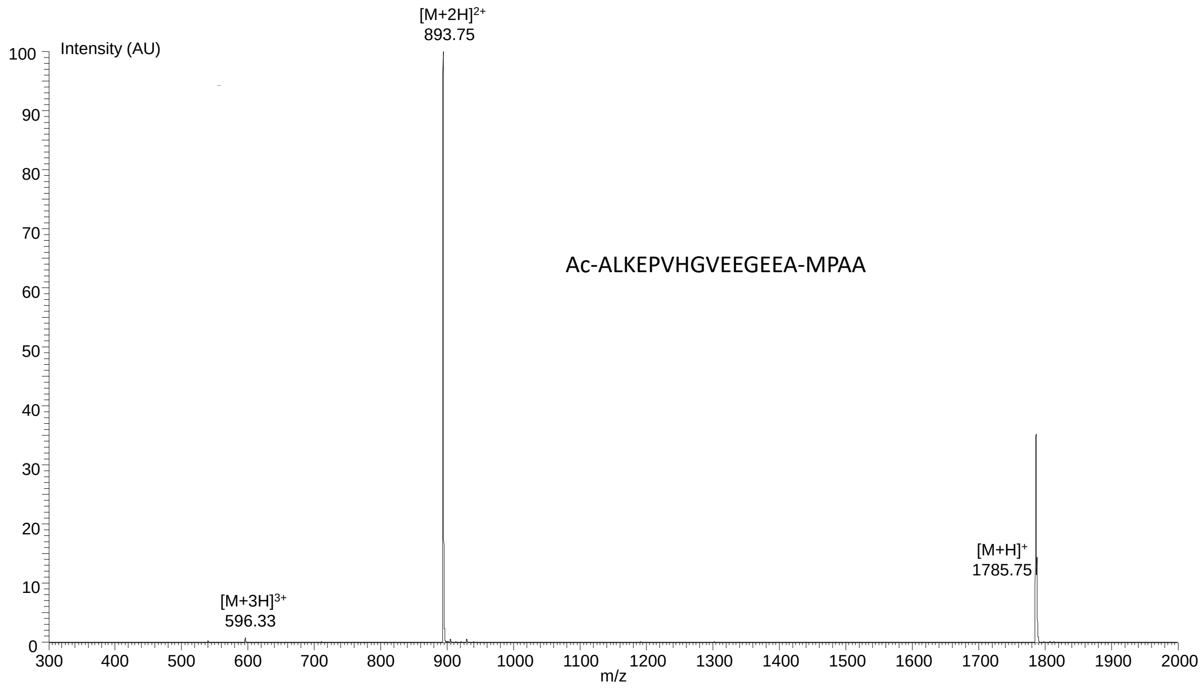

Supplementary Figure 26. MS trace of peptide thioester **1g** from UPLC-MS analysis. $[M+H]^+$ m/z calcd. (monoisotopic) 1785.79, obs. 1785.75, $[M+2H]^{2+}$ m/z calcd. (av.) 893.97, obs. 893.75, $[M+3H]^{3+}$ m/z calcd. (av.) 596.31, obs. 596.33.

Synthesis of Ac-GSGSGSGKPIPNPLLGLDSTASGSGSGpSGpSA-MPAA ***4***

The RP-HPLC purification was performed using a C18 XBridge column (50 °C, 215 nm, 6 mL min^{-1}, eluent A = water containing 0.1% v/v TFA, eluent B = CH_3CN containing 0.1% v/v of TFA, 0 to 50% B in 78 min). 8.0 mg of peptide hydrazide furnished 4.4 mg (55%) of purified peptide thioester **4**.

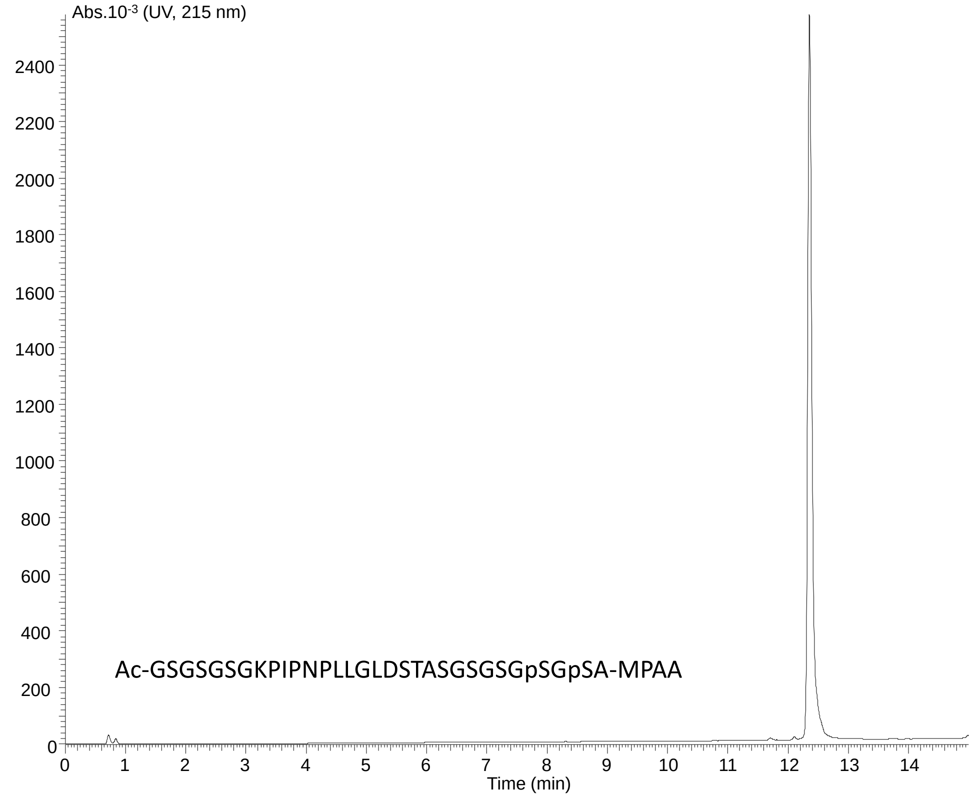

Supplementary Figure 27. UPLC-MS analysis of peptide thioester **4**. LC trace: Eluent A 0.1% TFA in water, eluent B 0.1% TFA in CH_3CN. C18 X bridge BEH 300 Å (5 µm, 4.6 × 250 mm) column, gradient 0-40% B in 15 min, 0.4 mL min^{-1}, detection at 215 nm).

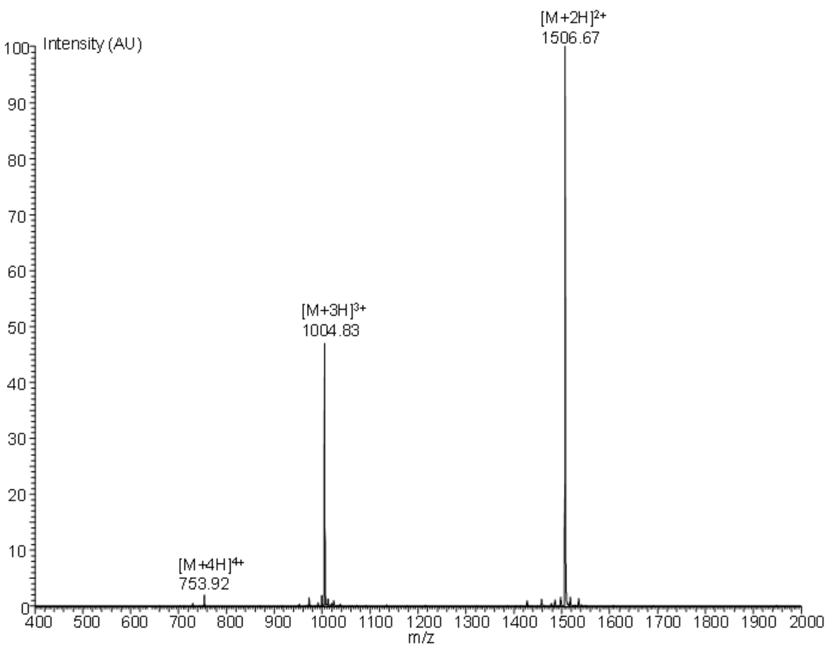

Ac-GSGSGSGKPIPNPLLGLDSTASGSGSGpSGpSA-MPAA

Supplementary Figure 28. MS trace of peptide thioester **4** from UPLC-MS analysis. $[M+2H]^{2+}$ m/z calcd. (av.) 1506.99, obs. 1506.67, $[M+3H]^{3+}$ m/z calcd. (av.) 1004.99, obs. 1004.83, $[M+4H]^{4+}$ m/z calcd. (av.) 753.99, obs. 753.92.

Synthesis of peptide nucleophiles

Synthesis of glycyl peptides 2a-g

Synthesis of peptide GRRRRRRALKEPVHGV-NH_2 ***2a***

The synthesis was performed on a 0.1 mmol scale using Novasyn TGR solid support. The peptide was cleaved from the solid support and deprotected using a mixture of TFA/water/TIS 92.5/2.5/5 v/v/v for 3 h 15 min and then precipitated in 200 mL of ice-cold Et_2O/heptane 1/1 v/v. The crude peptide was solubilized in water, frozen and lyophilized (162 mg). The RP-HPLC purification was performed using a C18 XBridge column (50 °C, 215 nm, 6 mL min^{-1}, eluent A = water containing 0.1% v/v TFA, eluent B = CH_3CN/water 4/1 v/v containing 0.1% v/v of TFA, 0 to 12% B in 5 min, then 12 to 32% B in 60 min). 37 mg of crude glycyl peptide **2a** furnished 23 mg (62%) of purified glycyl peptide **2a**.

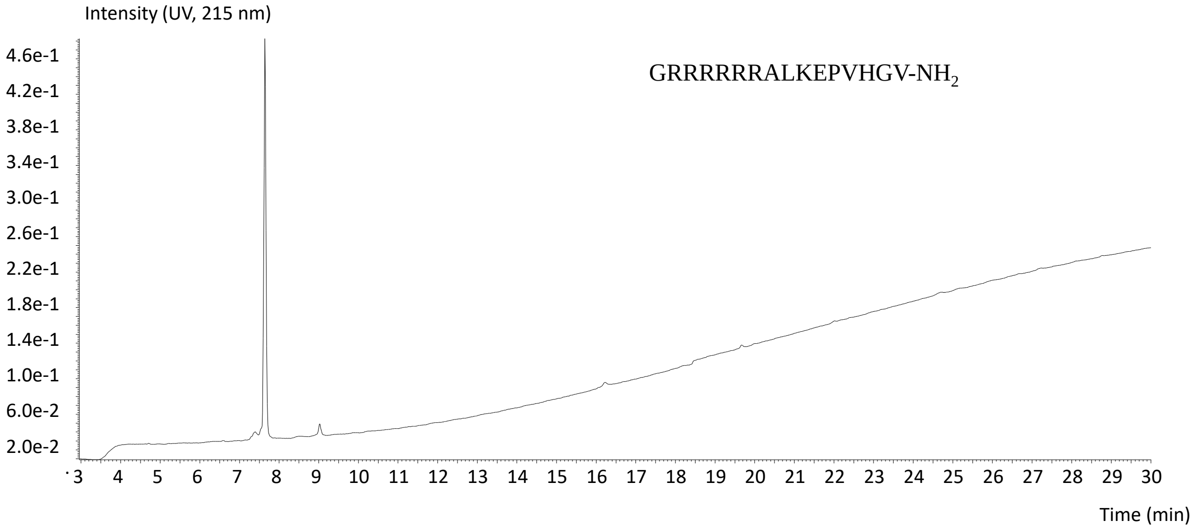

Supplementary Figure 29. LC-MS analysis of glycyl peptide **2a**. LC trace: Eluent A 0.1% TFA in water, eluent B 0.1% TFA in CH_3CN/water 4/1 v/v. C18 X bridge BEH 300 Å (5 µm, 4.6 × 250 mm) column, gradient 0-100% B in 30 min, 1 mL min^{-1}, detection at 215 nm).

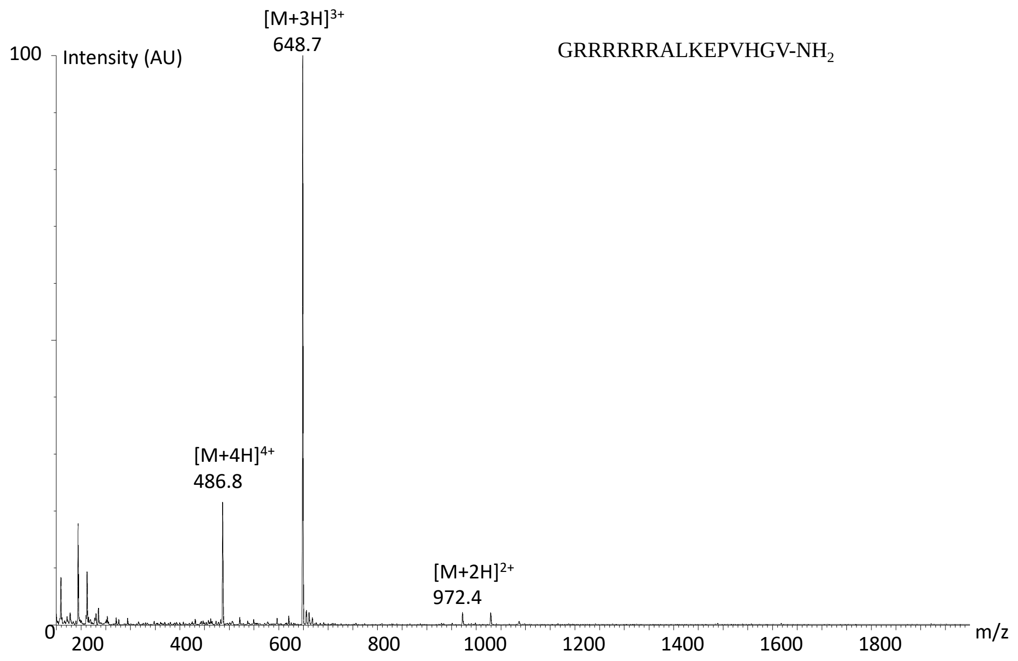

Supplementary Figure 30. MS trace of glycyl peptide **2a** from LC-MS analysis. $[M+2H]^{2+}$ m/z calcd. (av.) 972.16, obs. 972.4, $[M+3H]^{3+}$ m/z calcd. (av.) 648.44, obs. 648.7, $[M+4H]^{4+}$ m/z calcd. (av.) 486.58, obs. 486.8.

Synthesis of GRRRRRALKEPVHGV-NH_2 ***2b***

The synthesis was performed on a 0.1 mmol scale using Novasyn TGR solid support. The peptide was cleaved from the solid support and deprotected using a mixture of TFA/water/TIS 92.5/2.5/5 v/v/v for 3 h and then precipitated in 200 mL of ice-cold Et_2O/heptane 1/1 v/v. The peptide was solubilized in water, frozen and lyophilized (138 mg). The RP-HPLC purification was performed using a C18 XBridge column (50 °C, 215 nm, 6 mL min^{-1}, eluent A = water containing 0.1% v/v TFA, eluent B = CH_3CN/water 4/1 v/v containing 0.1% v/v of TFA, 0 to 10% B in 5 min, then 10 to 30% B in 60 min). 45 mg of crude glycyl peptide **2b** furnished 33 mg (74%) of purified glycyl peptide **2b**.

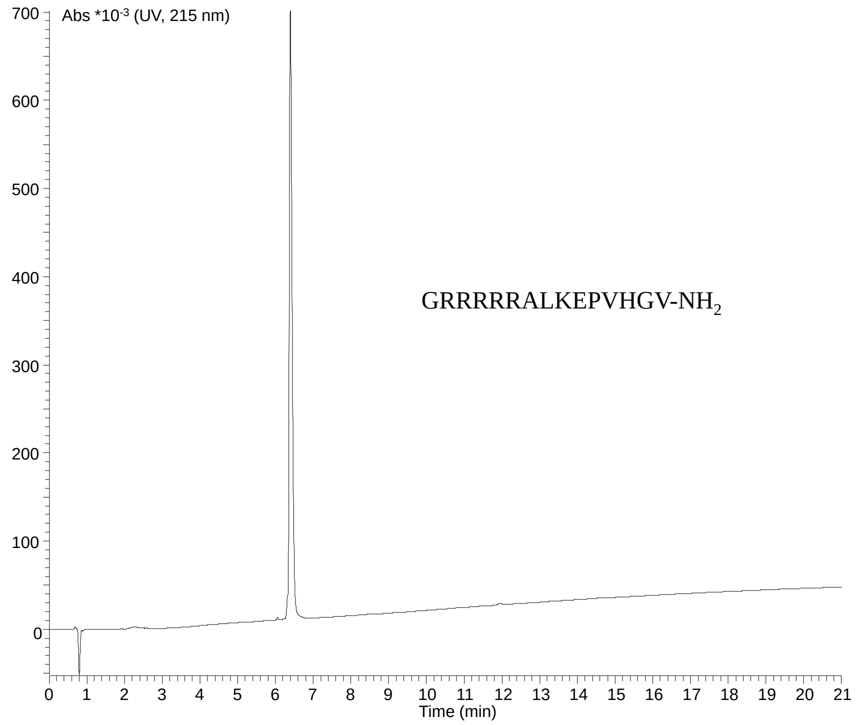

Supplementary Figure 31. UPLC-MS analysis of glycyl peptide **2b.** LC trace: Eluent A 0.1% TFA in water, eluent B 0.1% TFA in CH_3CN. BEH C18 (300 Å, 1.7 µm, 2.1 × 100 mm) column, gradient 0-70% B in 20 min, 0.4 mL min^{-1}, detection at 215 nm).

A)

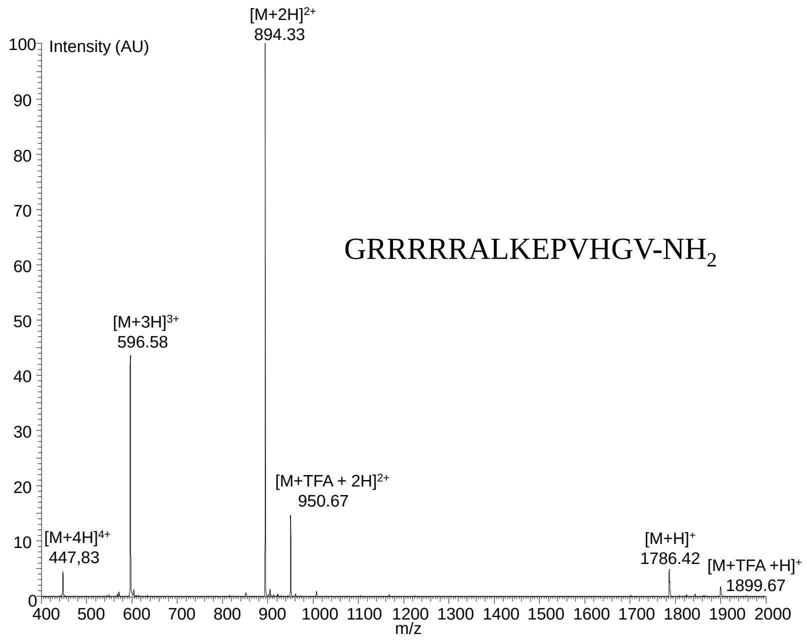

B)

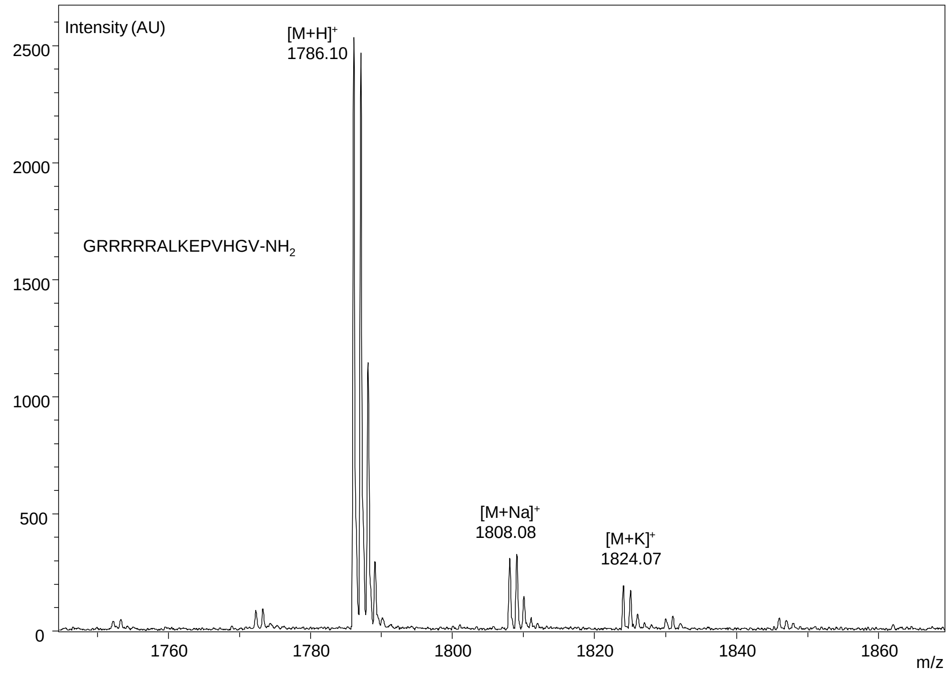

Supplementary Figure 32. Analysis of peptide **2b** by mass spectrometry. A) MS trace of peptide **2b** from LC-MS analysis: $[M+H]^+$ m/z calcd. (monoisotopic) 1786.08, obs. 1786.42, $[M+2H]^{2+}$ m/z calcd. (av.) 894.06, obs. 894.33, $[M+3H]^{3+}$ m/z calcd. (av.) 596.38, obs. 596.58, $[M+4H]^{4+}$ m/z calcd. (av.) 447.53, obs. 447.83. B) MALDI-TOF analysis of glycyl peptide **2b**. Matrix alpha-cyano-4-hydroxycinnaminic acid, positive detection mode, $[M+H]^+$ m/z calcd. (monoisotopic) 1786.09, found 1786.10.

Synthesis of GRRRRALKEPVHGV-NH_2 ***2c***

The synthesis was performed on a 0.1 mmol scale using Novasyn TGR solid support. The peptide was cleaved from the solid support and deprotected using a mixture of TFA/water/TIS 92.5/2.5/5 v/v/v for 2 h 30 min and then precipitated in 200 mL of ice-cold Et_2O/heptane 1/1 v/v. The peptide was solubilized in water, frozen and lyophilized (132 mg). The RP-HPLC purification was performed using a C18 XBridge column (50 °C, 215 nm, 6 mL min^{-1}, eluent A = water containing 0.1% v/v TFA, eluent B = CH_3CN/water 4/1 v/v containing 0.1% v/v of TFA, 0 to 10% B in 5 min, then 10 to 30% B in 60 min). 44 mg of crude glycyl peptide **2c** furnished 33 mg (75%) of purified glycyl peptide **2c**.

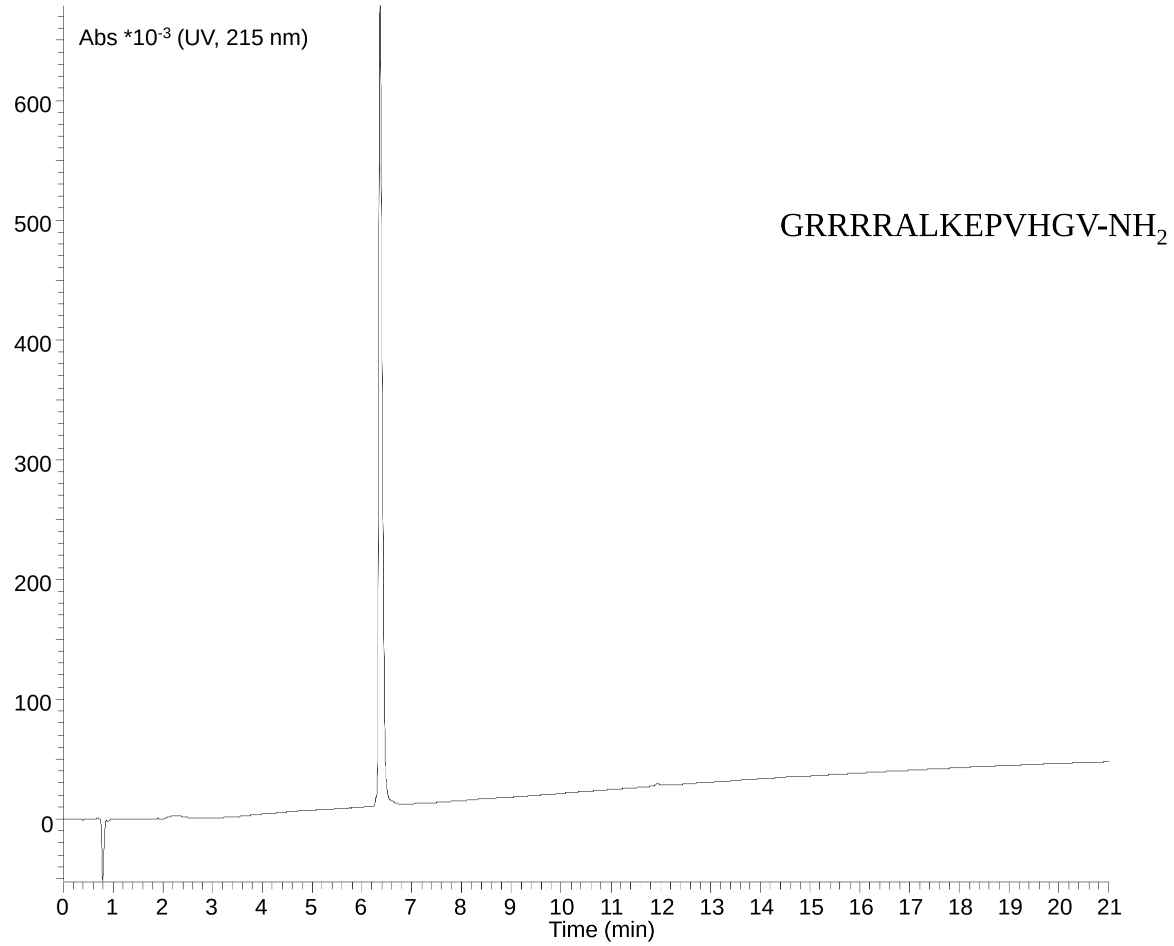

Supplementary Figure 33. UPLC-MS analysis of glycyl peptide **2c.** LC trace: Eluent A 0.1% TFA in water, eluent B 0.1% TFA in CH_3CN. BEH C18 (300 Å, 1.7 µm, 2.1 × 100 mm), gradient 0-70% B in 20 min, 0.4 mL min^{-1}, detection at 215 nm).

A)

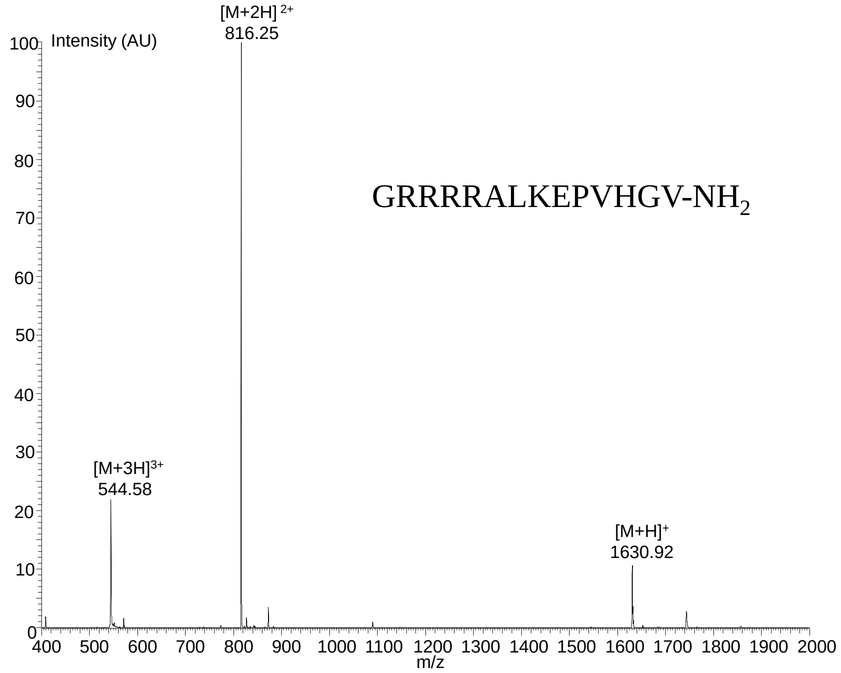

B)

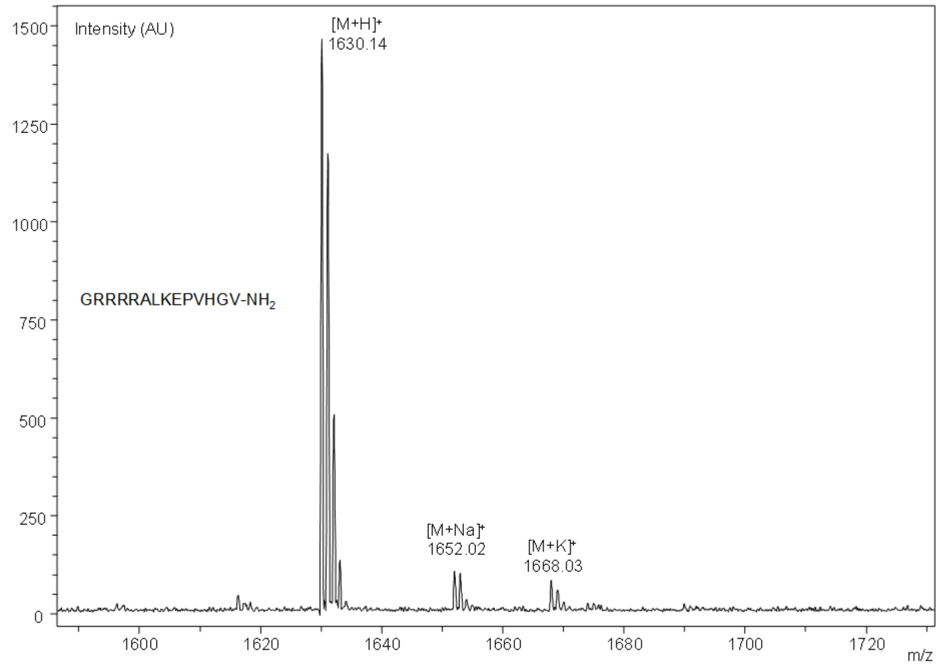

Supplementary Figure 34. Analysis of peptide **2c** by mass spectrometry. A) MS trace of glycyl peptide **2c** from UPLC-MS analysis. $[M+H]^+$ m/z calcd. (av.) 1630.94, obs.1630.92, $[M+2H]^{2+}$ m/z calcd. (av.) 815.97, obs. 816.25, $[M+3H]^{3+}$ m/z calcd. (av.) 544.31, obs. 544.58. B) MALDI-TOF analysis of glycyl peptide **2c**. Matrix alpha-cyano-4-hydroxycinnaminic acid, positive detection mode, $[M+H]^+$ m/z calcd. (monoisotopic) 1629.99, found 1630.14.

Synthesis of GRRRALKEPVHGV-NH$_2$ ***2d***

The synthesis was performed on a 0.1 mmol scale using Novasyn TGR solid support. The peptide was cleaved from the solid support and deprotected using a mixture of TFA/water/TIS 92.5/2.5/5 v/v/v for 2 h 30 min and then precipitated in 200 mL of ice-cold Et_2O/heptane 1/1 v/v. The peptide was solubilized in water, frozen and lyophilized (121 mg). The RP-HPLC purification was performed using a C18 XBridge column (50 °C, 215 nm, 6 mL min^{-1}, eluent A = water containing 0.1% v/v TFA, eluent B = CH_3CN/water 4/1 v/v containing 0.1% v/v of TFA, 0 to 10% B in 5 min, then 10 to 30% B in 60 min). 43 mg of crude glycyl peptide **2d** furnished 34 mg (79%) of purified glycyl peptide **2d**.

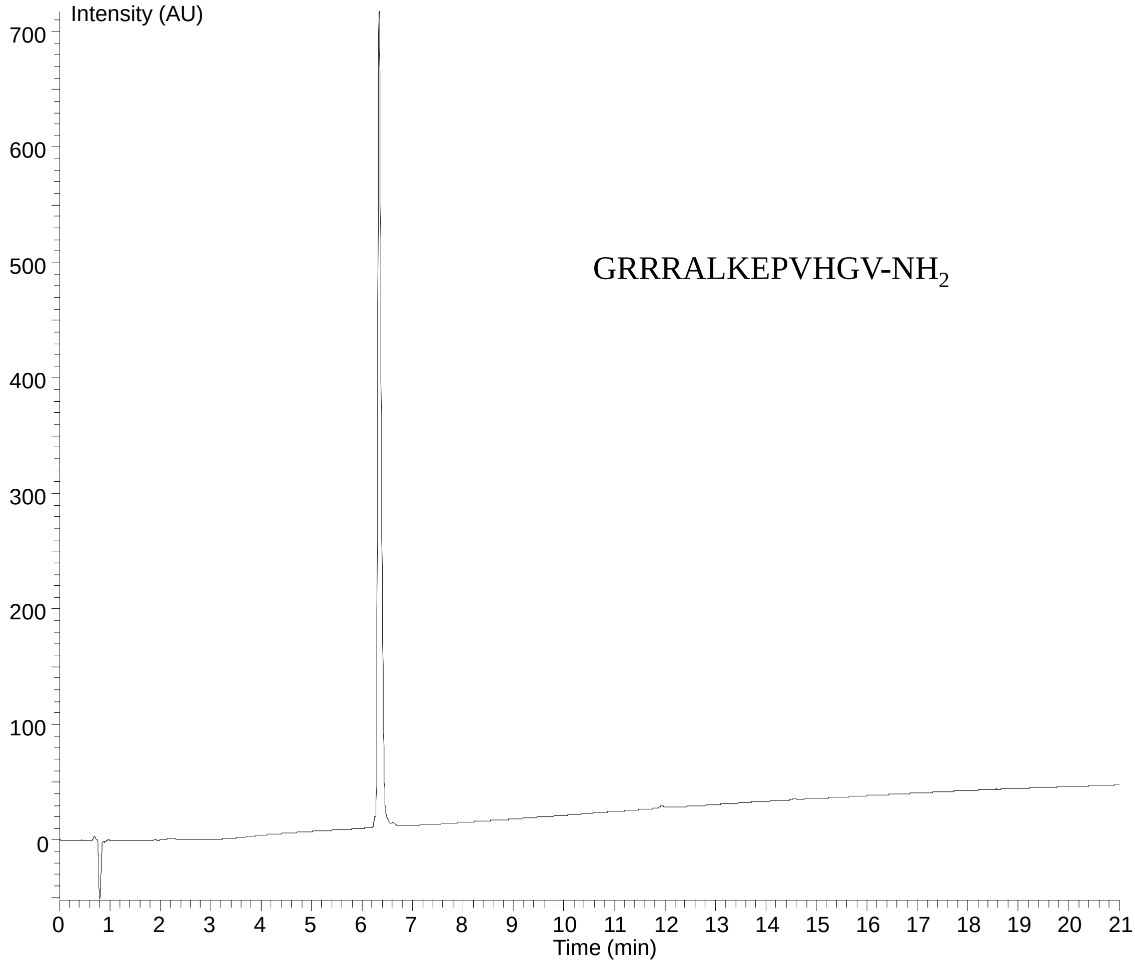

Supplementary Figure 35. UPLC-MS analysis of glycyl peptide **2d**. LC trace: Eluent A 0.1% TFA in water, eluent B 0.1% TFA in CH_3CN. BEH C18 (300 Å, 1.7 µm, 2.1 × 100 mm) column, gradient 0-70% B in 20 min, 0.4 mL min^{-1}, detection at 215 nm).

A)

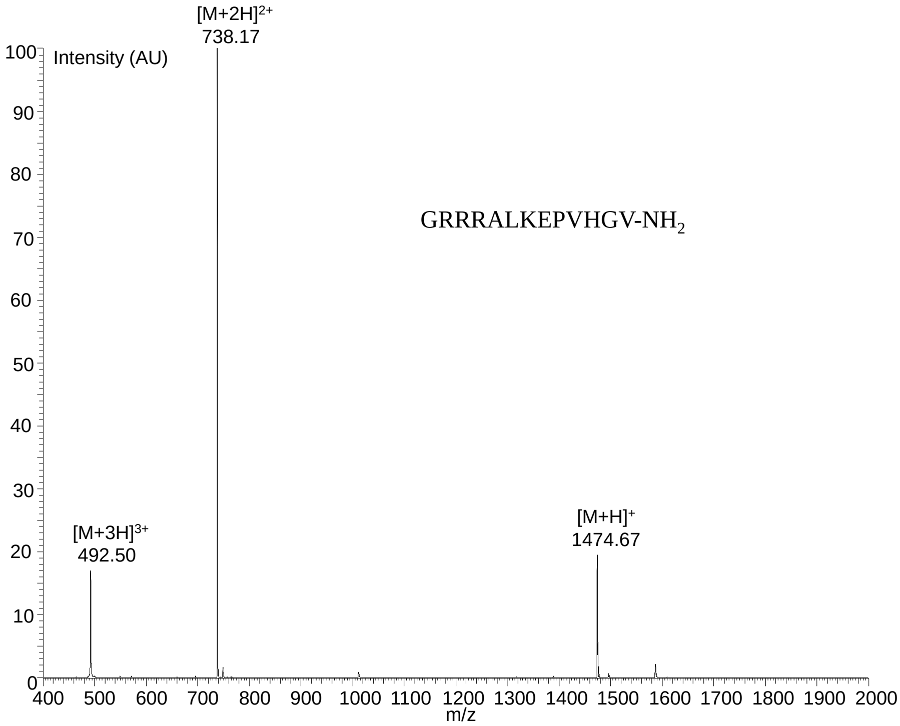

B)

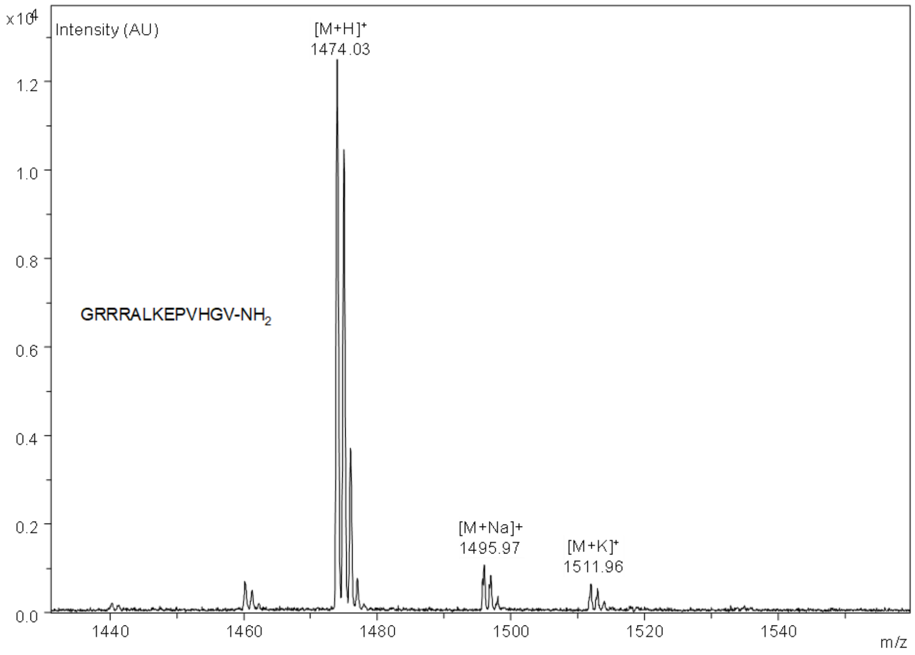

Supplementary Figure 36. Analysis of peptide **2d** by mass spectrometry. A) MS trace of glycyl peptide **2d** from UPLC-MS analysis. $[M+H]^+$ m/z calcd. (av.) 1474.76, obs. 1474.67, $[M+2H]^{2+}$ m/z calcd. (av.) 737.88, obs. 738.17, $[M+3H]^{3+}$ m/z calcd. (av.) 492.25, obs. 492.50. B) MALDI-TOF analysis of glycyl peptide **2d**. Matrix alpha-cyano-4-hydroxycinnaminic acid, positive detection mode, $[M+H]^+$ m/z calcd. (monoisotopic) 1473.89, found 1474.03.

Synthesis of GRRALKEPVHGV-NH₂ ***2e***

The synthesis was performed on a 0.1 mmol scale using Novasyn TGR solid support. The peptide was cleaved from the solid support and deprotected using a mixture of TFA/water/TIS 92.5/2.5/5 v/v/v for 1 h 30 min and then precipitated in 200 mL of ice-cold Et_2O/heptane 1/1 v/v. The peptide was solubilized in water, frozen and lyophilized (109 mg). The RP-HPLC purification was performed using a C18 XBridge column (50 °C, 215 nm, 6 mL min^{-1}, eluent A = water containing 0.1% v/v TFA, eluent B = CH_3CN/water 4/1 v/v containing 0.1% v/v of TFA, 0 to 10% B in 5 min, then 10 to 30% B in 60 min). 42 mg of crude glycyl peptide **2e** furnished 33 mg (79%) of purified glycyl peptide **2e**.

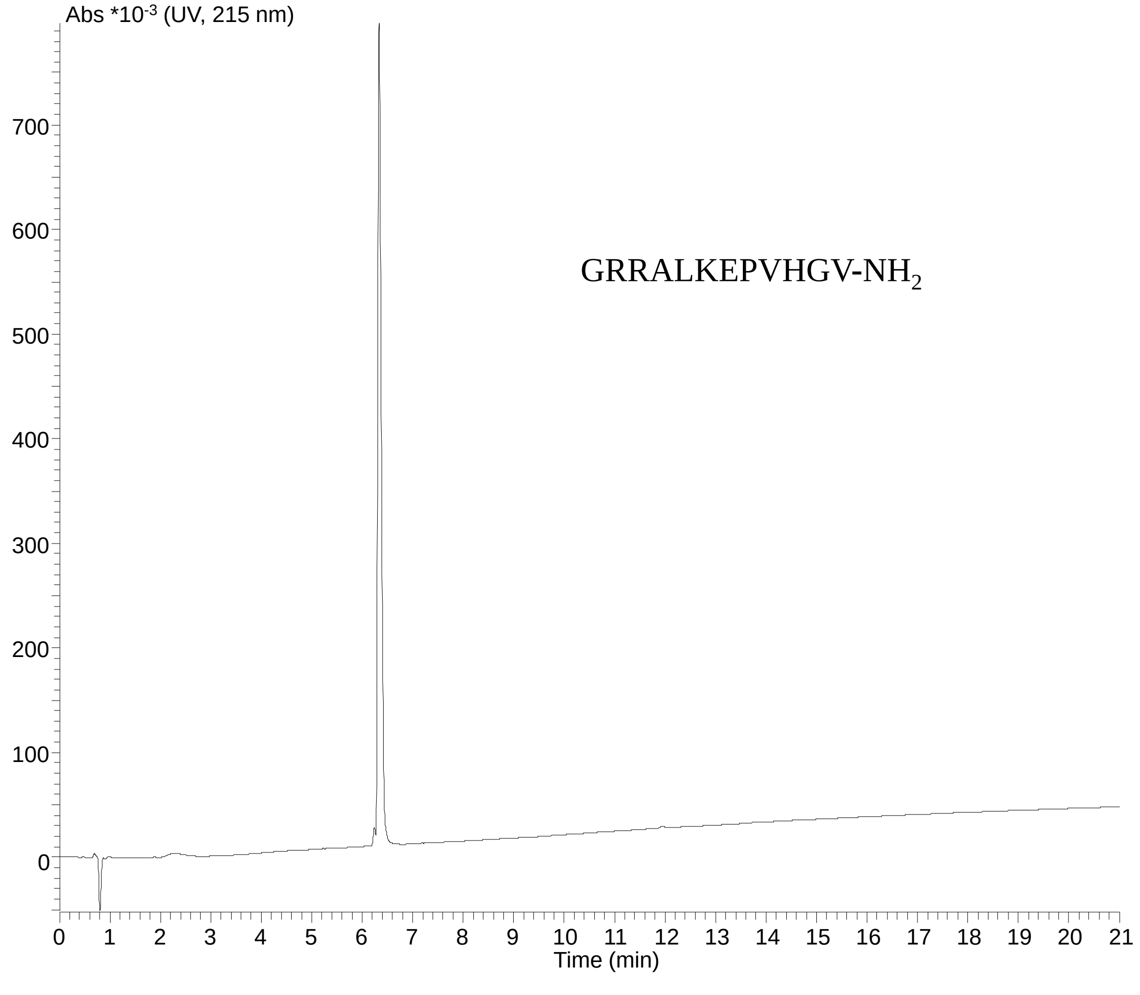

Supplementary Figure 37. UPLC-MS analysis of glycyl peptide **2e**. LC trace: Eluent A 0.1% TFA in water, eluent B 0.1% TFA in CH_3CN. BEH C18 (300 Å, 1.7 µm, 2.1 × 100 mm) column, gradient 0-70% B in 20 min, 0.4 mL min^{-1}, detection at 215 nm).

A)

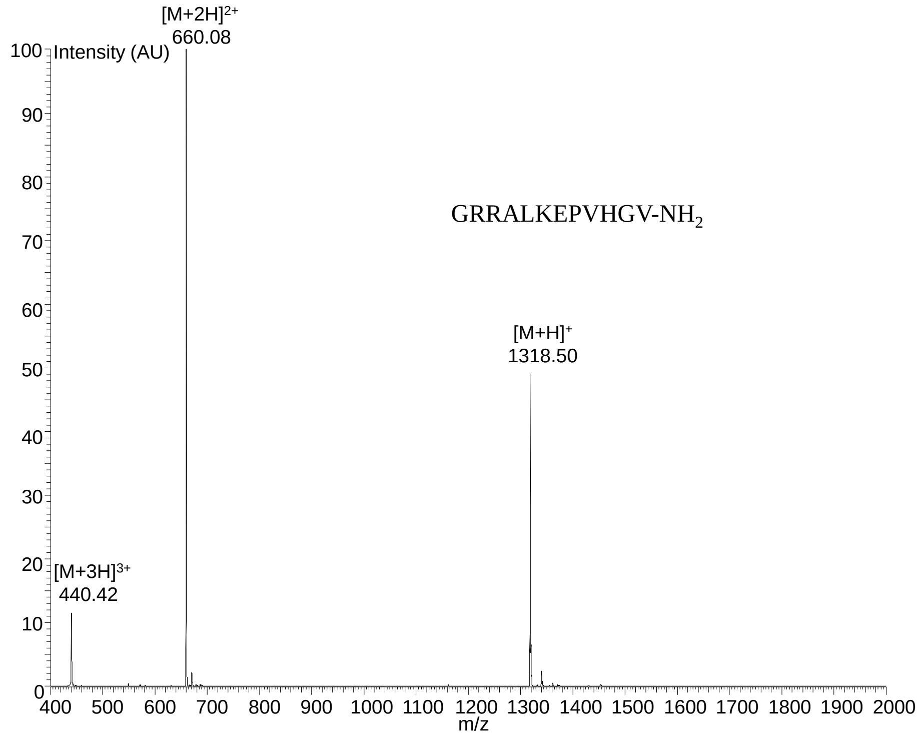

B)

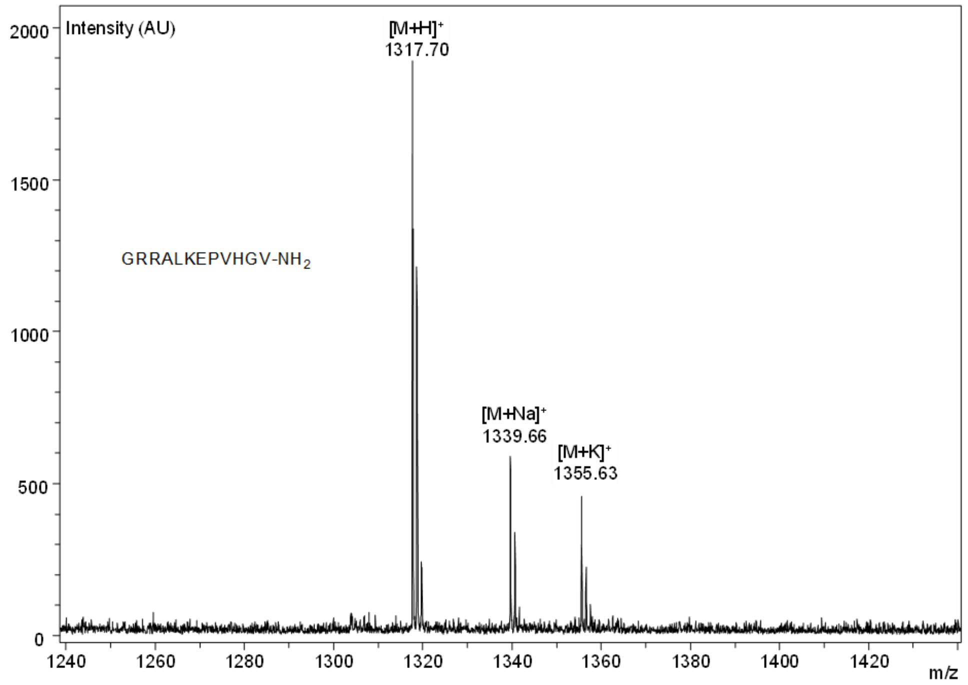

Supplementary Figure 38. Analysis of peptide **2e** by mass spectrometry. A) MS trace of glycyl peptide **2e** from UPLC-MS analysis. $[M+H]^+$ m/z calcd. (av.) 1318.57, obs. 1318.50, $[M+2H]^{2+}$ m/z calcd. (av.) 659.78, obs. 660.08, $[M+3H]^{3+}$ m/z calcd. (av.) 440.19, obs. 440.42. B) MALDI-TOF analysis of glycyl peptide **2e**. Matrix alpha-cyano-4-hydroxycinnaminic acid, positive detection mode, $[M+H]^+$ m/z calcd. (monoisotopic) 1317.79, found 1317.70.

Synthesis of GRALKEPVHGV-NH$_2$ ***2f***

The synthesis was performed on a 0.1 mmol scale using Novasyn TGR solid support. The peptide was cleaved from the solid support and deprotected using a mixture of TFA/water/TIS 92.5/2.5/5 v/v/v for 1 h 30 min and then precipitated in 200 mL of ice-cold Et_2O/heptane 1/1 v/v. The peptide was solubilized in water, frozen and lyophilized (94 mg). The RP-HPLC purification was performed using on a C18 XBridge column (50 °C, 215 nm, 6 mL min^{-1}, eluent A = water containing 0.1% v/v TFA, eluent B = CH_3CN/water 4/1 v/v containing 0.1% v/v of TFA, 0 to 10% B in 5 min, then 10 to 30% B in 60 min). 43 mg of crude glycyl peptide **2f** furnished 35 mg (80%) of purified glycyl peptide **2f**.

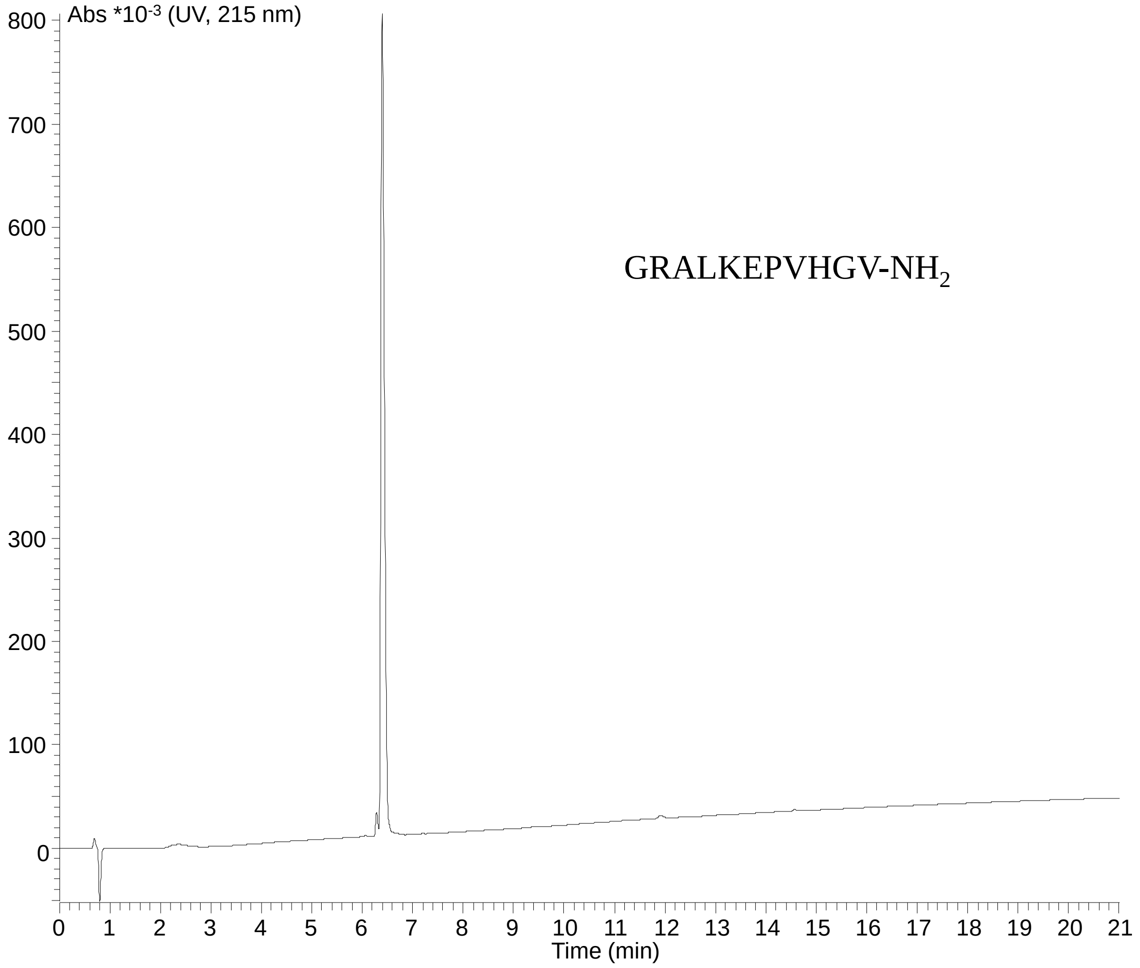

Supplementary Figure 39. UPLC-MS analysis of glycyl peptide **2f**. LC trace: Eluent A 0.1% TFA in water, eluent B 0.1% TFA in CH_3CN. BEH C18 (300 Å, 1.7 µm, 2.1 × 100 mm) column, gradient 0-70% B in 20 min, 0.4 mL min^{-1}, detection at 215 nm).

A)

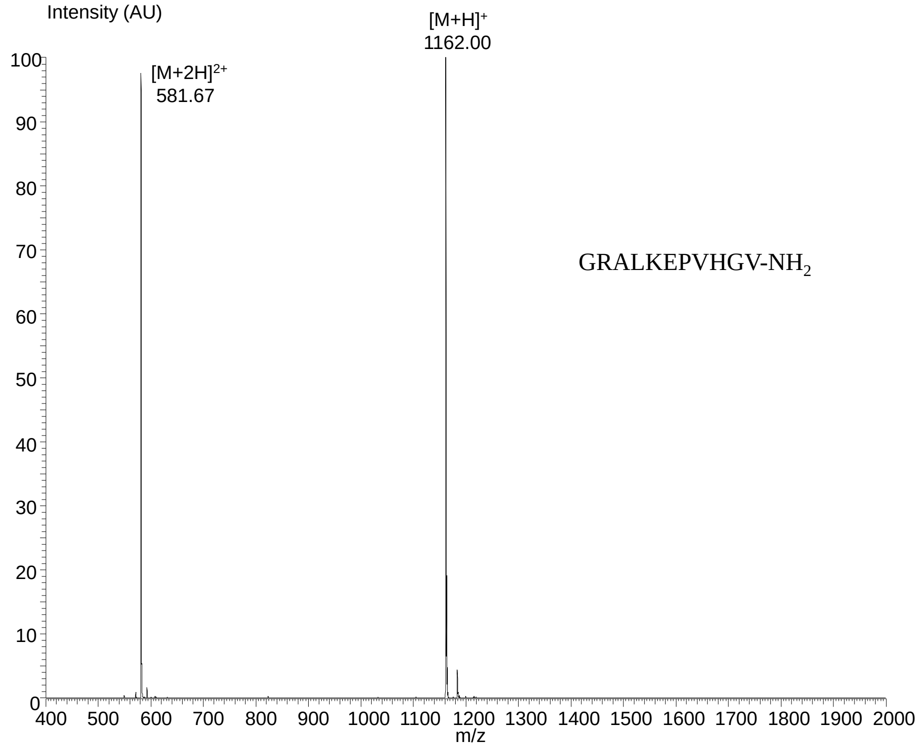

B)

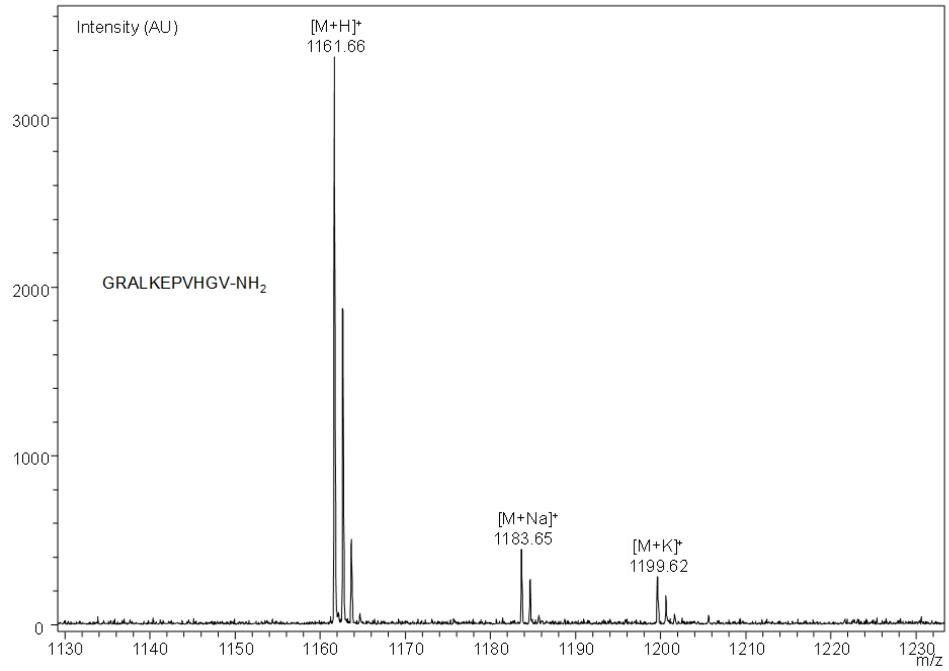

Supplementary Figure 40. Analysis of peptide **2f** by mass spectrometry. A) MS trace of glycyl peptide **2f** from UPLC-MS analysis. $[M+H]^+$ m/z calcd. (monoisotopic) 1161.68, obs. 1162.00, $[M+2H]^{2+}$ m/z calcd. (av.) 581.69, obs. 581.67. B) MALDI-TOF analysis of glycyl peptide **2f**. Matrix alpha-cyano-4-hydroxycinnaminic acid, positive detection mode, $[M+H]^+$ m/z calcd. (monoisotopic) 1161.68, found 1161.66.

Synthesis of GALKEPVHGV-NH_2 ***2g***

The synthesis was performed on a 0.1 mmol scale using Novasyn TGR solid support. The peptide was cleaved from the solid support and deprotected using a mixture of TFA/water/TIS 92.5/2.5/5 v/v/v for 1 h 30 min and then precipitated in 200 mL of ice-cold Et_2O/heptane 1/1 v/v. The peptide was solubilized in water, frozen and lyophilized (80 mg). The RP-HPLC purification was performed using on a C18 XBridge column (50 °C, 215 nm, 6 mL min^{-1}, eluent A = water containing 0.1% v/v TFA, eluent B = CH_3CN/water 4/1 v/v containing 0.1% v/v of TFA, 0 to 10% B in 5 min, then 10 to 40% B in 90 min). 40 mg of crude glycyl peptide **2g** furnished 31 mg (77%) of purified glycyl peptide **2g**.

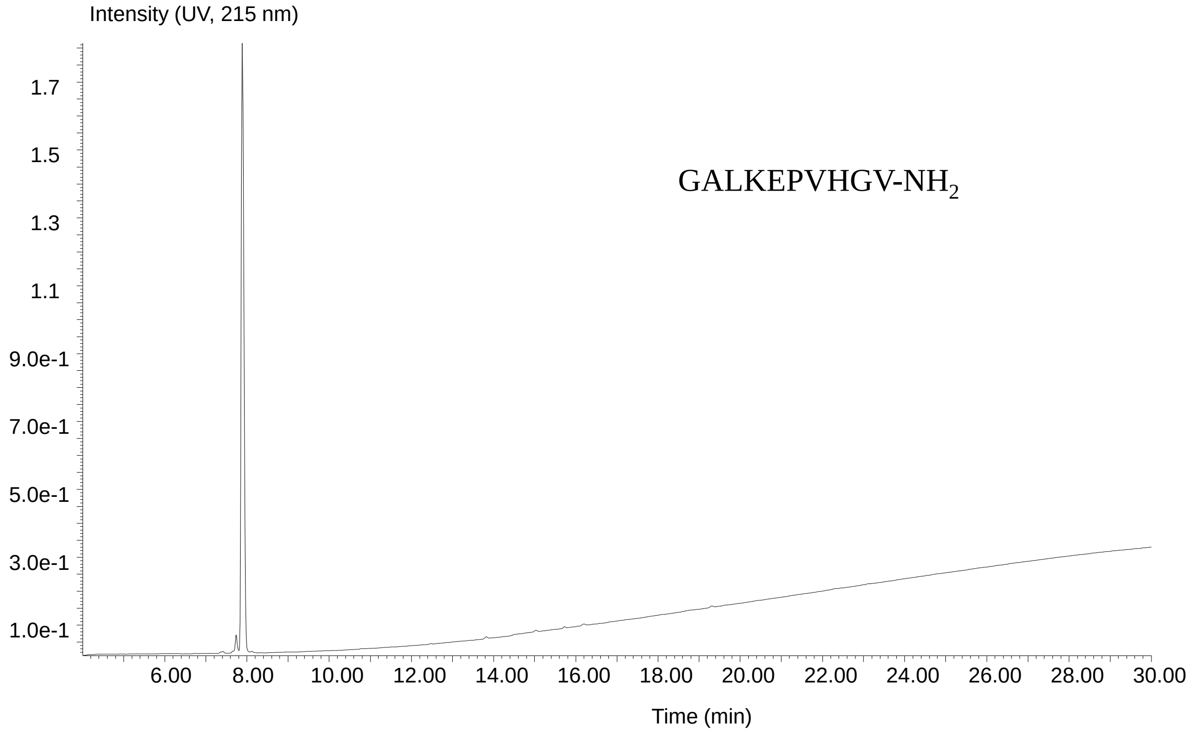

Supplementary Figure 41**.** LC-MS analysis of glycyl peptide **2g**. LC trace: Eluent A 0.1% TFA in water, eluent B 0.1% TFA in CH_3CN/water 4/1 v/v. C18 X bridge BEH 300 Å (5 µm, 4.6 × 250 mm) column, gradient 0-100% B in 30 min, 1 mL min^{-1}, detection at 215 nm).

A)

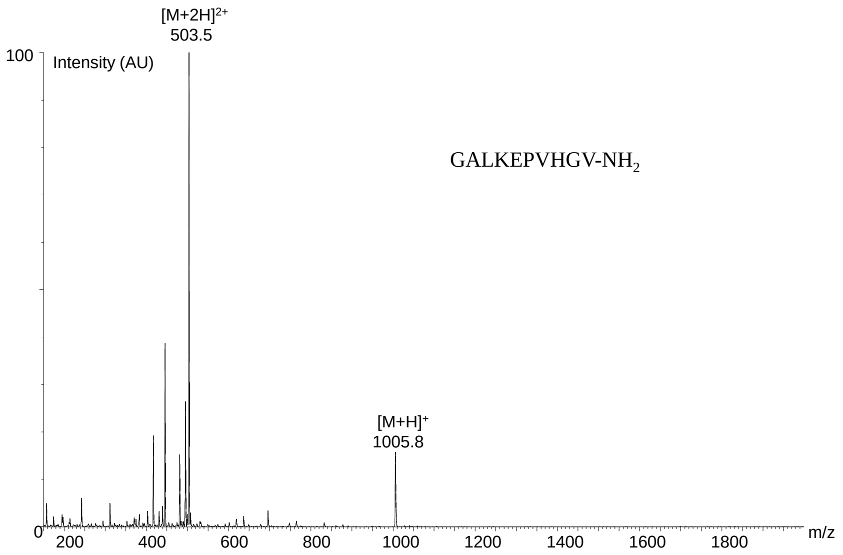

B)

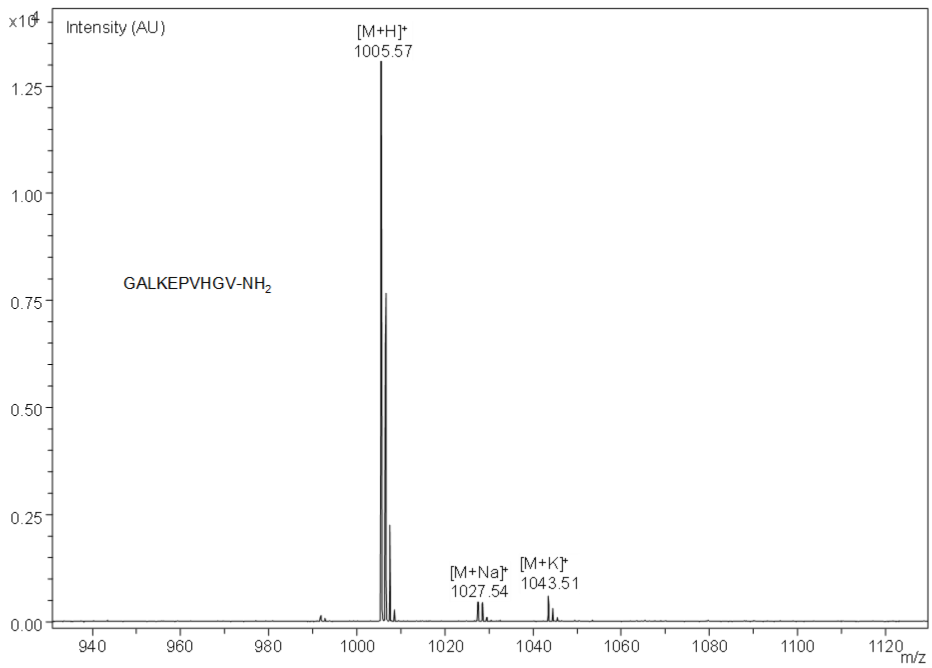

Supplementary Figure 42. Analysis of peptide **2g** by mass spectrometry. A) MS trace of glycyl peptide **2g** from LC-MS analysis. MS trace: $[M+H]^+$ m/z calcd. (monoisotopic) 1005.58, obs. 1005.8, $[M+2H]^{2+}$ m/z calcd. (av.) 503.59, obs. 503.5. B) MALDI-TOF analysis of glycyl peptide **2g**. Matrix alpha-cyano-4-hydroxycinnaminic acid, positive detection mode, $[M+H]^+$ m/z calcd. (monoisotopic) 1005.58, found 1005.57.

Production of Gly(Arg)$_6$-I27 titin protein 5 in *E. coli*

Materials

Bioruptor Sonication System, Diagenode

NGC chromatography systems, Biorad

HiTrap Talon Crude Prepacked column, ref: 28953766, Cytiva

NuPAGE 4 to 12%, Bis-Tris, 1.5 mm Midi Protein Gel, ref: WG1402A, Invitrogen

Slide-A-Lyzer Dialysis Cassettes, 3.5K MWCO, 12 mL, ref: 66110, Thermo Fisher Scientific

ZORBAX 300SB-C3 5 µm 250 × 9.4 mm HPLC column, ref: 880995-209, Interchim

AcTEV Protease, ref: 10216572, Thermo Fisher, Scientific

SOC Medium, ref: 15544034, Invitrogen

BL21(DE3) competent *E. coli* cells, ref: C2527H**,** New England Biolabs

Luria Broth base, ref: 12795027, Invitrogen

Complete EDTA-free Protease Inhibitor Cocktail, ref: 11873580001, Roche

Halt Protease Inhibitor Cocktail, EDTA-Free (100X), ref: 7843, Pierce

Imidazole, ref: I5513-100G, Sigma

Ampicillin, ref: A9393-25G**,** Sigma

Isopropyl β-D-1-thiogalactopyranoside, I5502-10G**,** Sigma

DNase I from bovine pancreas, ref: DN25-100MG, Sigma

Trizma hydrochloride, ref: T3253-500G, Sigma

Coomassie Brilliant Blue R, ref: B-0149, Sigma

LDS NuPage sample buffer 4X, ref: NP0007, Invitrogen

MES-SDS running buffer, NP0002, Invitrogen

The sequence of the target Gly(Arg)$_6$-I27 titin protein **5** is:

GRRRRRRLIEVEKPLYGVEVFVGETAHFEIELSEPDVHGQWKLKGQPLTASPDCEIIED GKKHILILHNCQLGMTGEVSFQAANAKSAANLKVKEL-OH

To access this protein, the following construct was expressed in *E. coli* and cleaved (⇓) with TEV protease:

His$_6$-TEV-⇓-GRRRRRRLIEVEKPLYGVEVFVGETAHFEIELSEPDVHGQWKLKGQPLTASPDCEIIED GKKHILILHNCQLGMTGEVSFQAANAKSAANLKVKEL-OH

Where TEV stands for: ENLYFQ

Design of expression vector

The expression vector was designed as shown in **Supplementary Figure 43**.

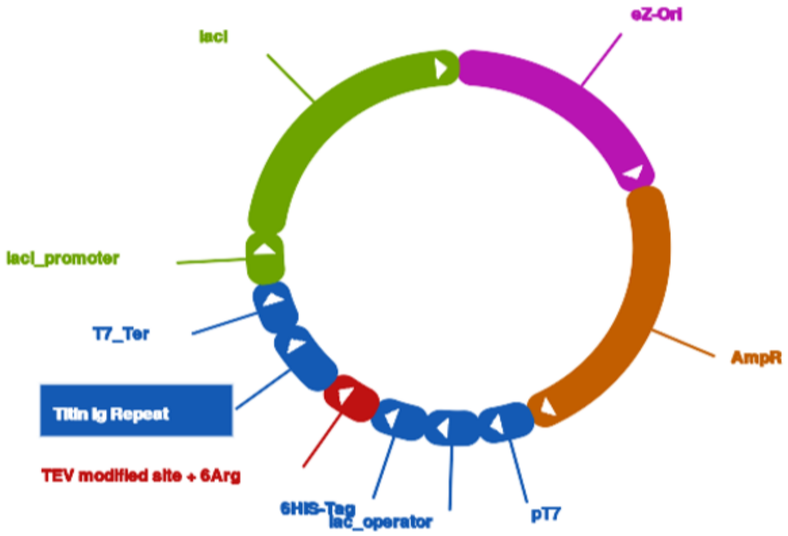

Supplementary Figure 43**.** Expression vector encoding recombinant His tag-TEV-Gly(Arg)$_6$-I27 titin protein, as designed by e-Zyvec company. eZ-Ori: High copy number origin of replication optimized for optimal assembly and plasmid amplification yield, derived from pMB1 (Origin compatibility group: A); AmpR: antibiotic resistance cassette for ampicillin selection of bacterial clones; pT7: T7 promoter; Lac-operator: Lac-operator; 6HIS-Tag: 6-histidine tag; T7-Ter: T7 terminator; LacI-promoter: The lactose repressor gene (lacI) promoter is used to drive a weak constitutive gene expression in *Escherichia coli*; lacI: lac repressor.

Transformation protocol for BL21(DE3) competent E. coli *cells*

BL21(DE3) competent *E. coli* cells were placed on ice for 10 min. Plasmid DNA (1 µL, 100 ng) was added to the cells (3 10^7 CFU), which were incubated on ice for further 30 min. The cells were placed at 42 °C for 40 s, and then cooled down on ice for 5 min. SOC medium (950 µL) equilibrated at room temperature was added to the cells and the cell suspension was placed at 37 °C for 1 h 30 min under vigorous shaking (250 rpm).

Luria Broth base (LB) supplemented with ampicillin (100 µg/mL, 5 mL) was added to the cell suspension, which was placed in an incubator at 37 °C and shaken overnight (180 rpm).

Production of His tag-TEV-Gly(Arg)$_6$-I27 titin protein

The cell suspension obtained above (5 mL) was diluted with LB supplemented with ampicillin (100 µg/mL, 500 mL) and placed in a baffle flask. The optical density (OD) of the cell suspension was measured at 600 nm and the culture was incubated until it reached 0.5–0.6 OD_{600}. Then, the protein expression was induced by adding isopropyl β-D-1-thiogalactopyranoside (IPTG) at a final concentration of 0.5 mM. The flask was kept in the incubator shaker for further 4 h at 37 °C. After this step, the culture was pelleted by centrifugation at 4000 × *g* for 30 min and stored at −20°C.

The pellet obtained from 1 L of culture was resuspended in 50 mL of bacterial lysis buffer consisting in 20 mM Tris·HCl pH 7, 200 mM NaCl supplemented with a cocktail of protease inhibitors (complete EDTA-free Protease Inhibitor Cocktail, a tablet for 500 mL), Halt Protease Inhibitor Cocktail, final concentration 1X) and DNase I from bovine pancreas (20 mg/L final concentration). The suspension was sonicated at 4 °C (Bioruptor, 60 on/off cycles of 35 s at 100% amplitude) and centrifuged at 7000 × *g* for 45 min at 4 °C to remove non-lysed cells. The supernatant was collected.

Purification of expressed His tag-TEV-Gly(Arg)$_6$-I27 titin protein

Imidazole (10 mM) was added to the supernatant, which was filtered on a 0.22 µm membrane. A HiTrap Talon prepacked column was equilibrated with the washing buffer made of 20 mM Trizma hydrochloride (Tris·HCl) pH 7, 200 mM NaCl, 10 mM imidazole supplemented with a cocktail of protease inhibitors (Complete EDTA-free Protease Inhibitor Cocktail, a tablet for 500 mL) and Halt Protease Inhibitor Cocktail, EDTA-Free (1X final concentration). The column was washed with at least 5 times the bed volume at a flow rate of 1 mL/min.

The column was loaded with the cell lysate (50 mL, 0.8 mL/min) and washed with the washing buffer (10 × the bed volume, 1 mL/min). Then, the protein was eluted with elution buffer (10 mM to 300 mM imidazole over 30 column volumes at a flow rate of 1 mL/min).

The purity of the different fractions was checked by SDS-PAGE electrophoresis followed by Coomassie blue staining (**Supplementary Figure 44**). For that, samples (32 µL) were mixed with 12.5 µl LDS NuPage sample buffer 4X supplemented with 5.5 µL of reducing solution (DTT 2.45 M, β-mercaptoethanol 2.35 M in water). Samples were heated at 70 °C for 10 min, cooled down and loaded (40 µL) on NuPAGE 4 to 12%, Bis-Tris, 1.5 mm Midi Protein Gel. The separation was performed using a MES-SDS running buffer at 150 V for 1 h 30 min.

The gel was fixed and stained in Coomassie solution (40% MeOH, 8% acetic acid, 0.01% Coomassie Brilliant Blue R) for 1 h and decolorized (20% MeOH, 8% acetic acid) until obtaining a clear background.

The purified fractions of Titin protein were pooled and concentrated twice under reduced pressure. Imidazole was removed by dialyzing overnight at 4 °C against a buffer containing 40 mM Tris·HCl pH 7 and 10 mM EDTA (4 L) using a Slide-A-Lyzer Dialysis Cassette, 12 mL (3.5 kDa cut-off).

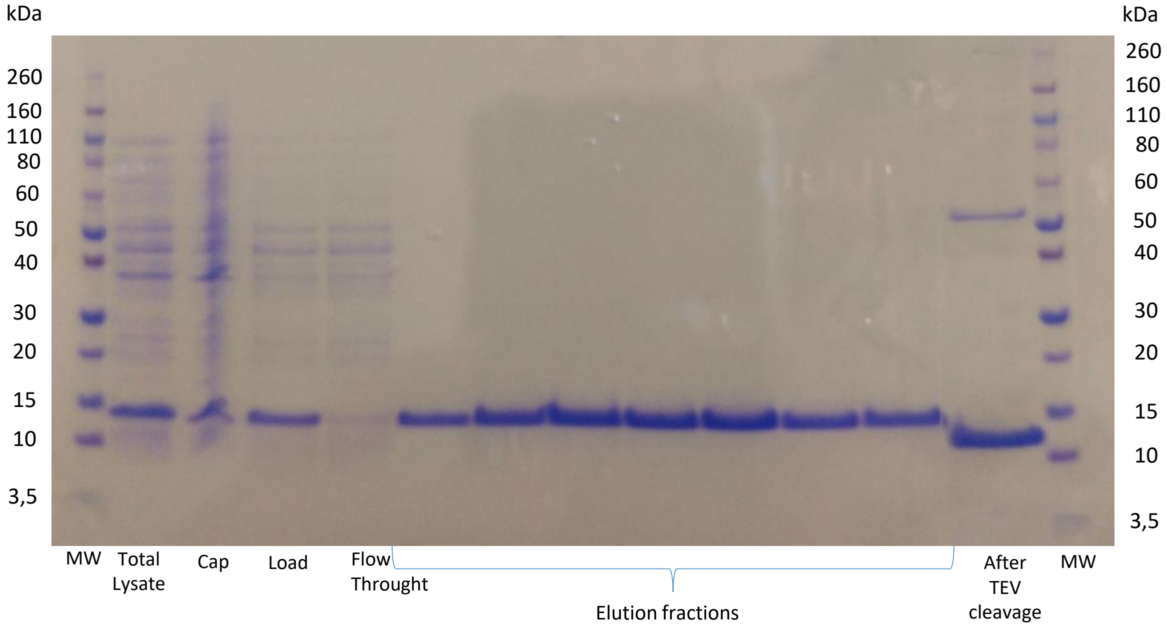

Supplementary Figure 44. SDS-Page analysis of the different solutions obtained during the production of His tag-TEV-Gly(Arg)$_6$-I27 titin protein. MW: molecular weight markers.

TEV cleavage

DTT was added to the dialyzed protein obtained above (0.1 M, 41 µL for 4.1 mL of protein solution).

A TEV protease/target protein (w/w) ratio of 1:20 was used (205 µL of the commercial enzyme solution, 10000 units/mL) to cleave the protein for 1 h at 30 °C under agitation. The cleavage

was monitored by UPLC-MS (**Supplementary Figure 45**). The entire sample was cleaved in about one hour as shown in **Supplementary Figure 44** and **Supplementary Figure 45**.

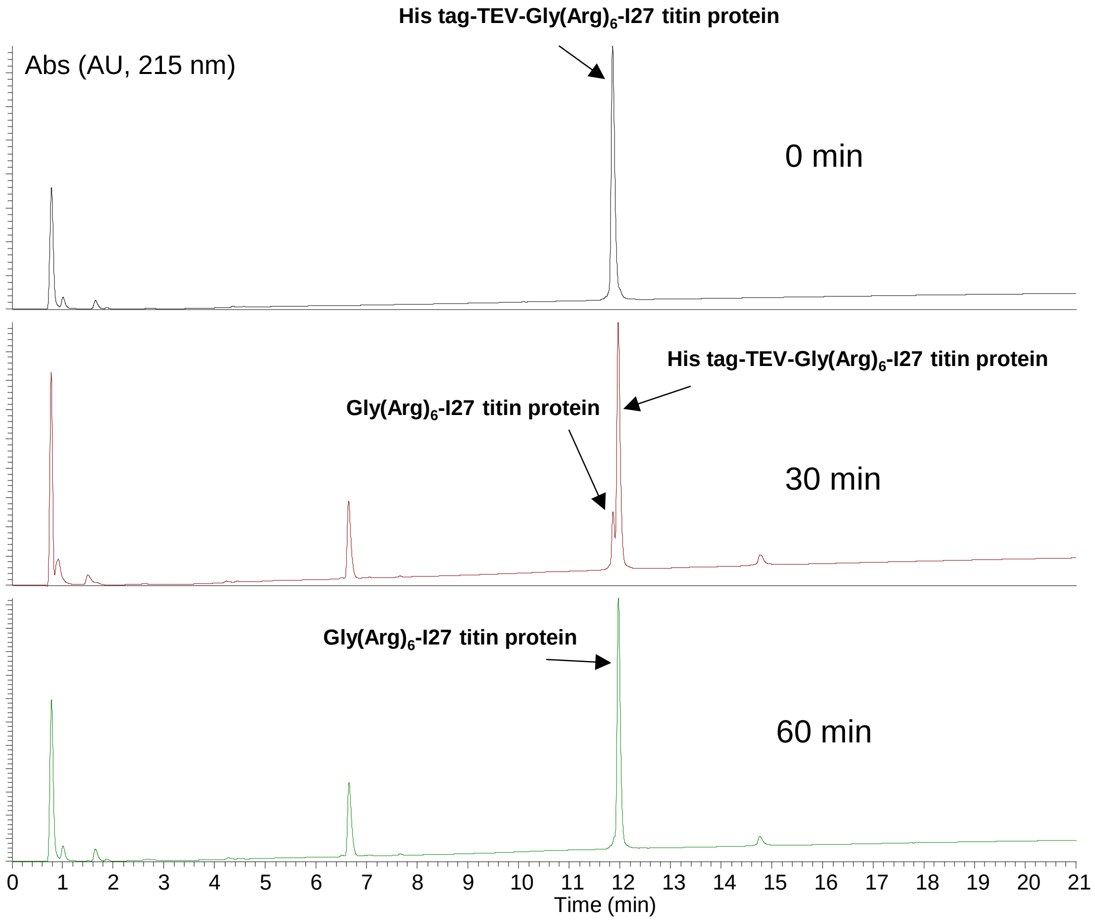

Supplementary Figure 45. The cleavage of His tag-TEV tag by TEV protease was monitored by UPLC-MS.

Purification of Gly(Arg)$_6$-I27 titin protein ***5***

TCEP (1 mg/mL, 500 µL) was added to the above crude protein solution before purification by RP-HPLC. The purification was performed using a ZORBAX 300SB-C3 (5 µm, 250 × 9.4 mm) HPLC column at 70 °C, eluent A = water containing 0.1% v/v TFA, eluent B = CH_3CN containing 0.1% v/v of TFA, 0-20% B in 5 min then 20-40% B in 35 min.

The purified fractions were collected and lyophilized to give 4.0 mg of Gly(Arg)$_6$-I27 titin protein **5**.

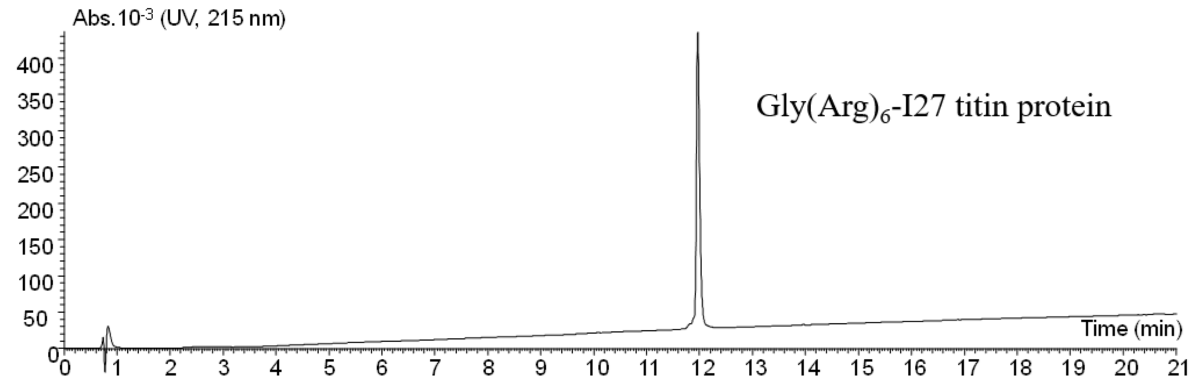

Supplementary Figure 46. UPLC-MS analysis of purified Gly(Arg)$_6$-I27 titin protein **5**. LC trace. Eluent A 0.1% TFA in water, eluent B 0.1% TFA in CH_3CN. Agilent SB C3 column (1.8 µm, 3.0 × 100 mm), gradient 0-70% B in 20 min, 70 °C, 0.4 mL min^{-1}, detection at 215 nm).

A)

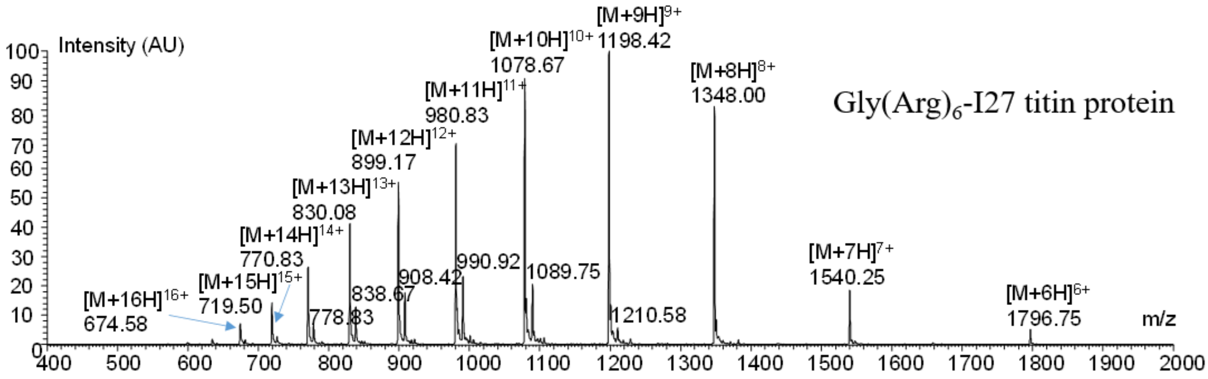

B)

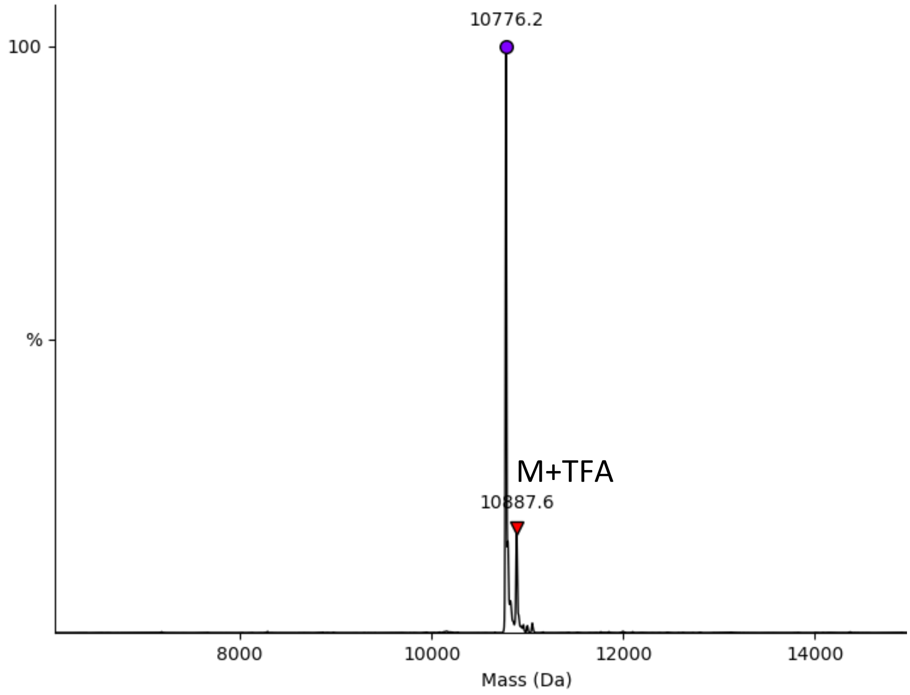

Supplementary Figure 47. Analysis of Gly(Arg)$_6$-I27 titin protein **5** by mass spectrometry. A) MS trace of purified Gly(Arg)$_6$-I27 titin protein **5** from UPLC-MS analysis. The presence of the Arg$_6$ motif favours the formation of TFA adducts in the MS spectrum of the protein. MS trace $[M+6H]^{6+}$ m/z calcd. (av.) 1797.07, obs. 1796.75, $[M+7H]^{7+}$ m/z calcd. (av.) 1540.49, obs. 1540.25, $[M+8H]^{8+}$ m/z calcd. (av.) 1348.05, obs. 1348.00, $[M+9H]^{9+}$ m/z calcd. (av.) 1198.38, obs. 1198.42, $[M+10H]^{10+}$ m/z calcd. (av.) 1078.64, obs. 1078.67, $[M+11H]^{11+}$ m/z calcd. (av.) 980.67, obs. 980.83, $[M+12H]^{12+}$ m/z calcd. (av.) 899.04, obs. 899.17, $[M+13H]^{13+}$ m/z calcd. (av.) 829.96, obs. 830.08, $[M+14H]^{14+}$ m/z calcd. (av.) 770.74, obs. 770.83, $[M+15H]^{15+}$ m/z calcd. (av.) 719.43, obs. 719.50, $[M+16H]^{16+}$ m/z calcd. (av.) 674.53, obs. 674.58. B) Deconvoluted MS spectrum. M av. calcd. 10776.43, obs. 10776.2.

Chemical synthesis of Cys-$(Arg)_6$-K1 protein 9

The sequence of kringle 1 (K1) domain of hepatocyte growth factor/scatter factor (HGF/SF) is as retrieved from Uniprot:

>sp|P14210|128-209

CIIGKGRSYKGTVSITKSGIKCQPWSSMIPHEHSFLPSSYRGKDLQENYCRNPRGEEGG PWCFTSNPEVRYEVCDIPQCSEV

In this work, K1 domain refers to the following sequence, which has a K(Biot)-NH_2 extension at the C-terminus, where Biot stands for a biotin group attached to the side-chain of lysine, and an N-terminal alanine residue used as a spacer between K1 domain and Arg_6 module:

ACIIGKGRSYKGTVSITKSGIKCQPWSSMIPHEHSFLPSSYRGKDLQENYCRNPRGEEG GPWCFTSNPEVRYEVCDIPQCSEVK(Biot)-NH_2

The biotin enabled the characterization of the ligation product by SDS-PAGE.

K1 polypeptide **9** has additionally a Cys$(Arg)_6$ extension at the N-terminus, which constitutes the NCL ligation site:

Cys$(Arg)_6$-ACIIGKGRSYKGTVSITKSGIKCQPWSSMIPHEHSFLPSSYRGKDLQE

NYCRNPRGEEGGPWCFTSNPEVRYEVCDIPQCSEVK(Biot)-NH_2

Assembly strategy

K1 polypeptide **9** was assembled by ligating three peptide segments in one-pot as described in **Supplementary Figure 48**. The procedure is similar to the one published by our group in these papers [3, 4]. It utilizes the latent thioester properties of the *bis*(2-sulfanylethyl)amido group (SEA^{off}) to elongate the polypeptide in the N-to-C direction.

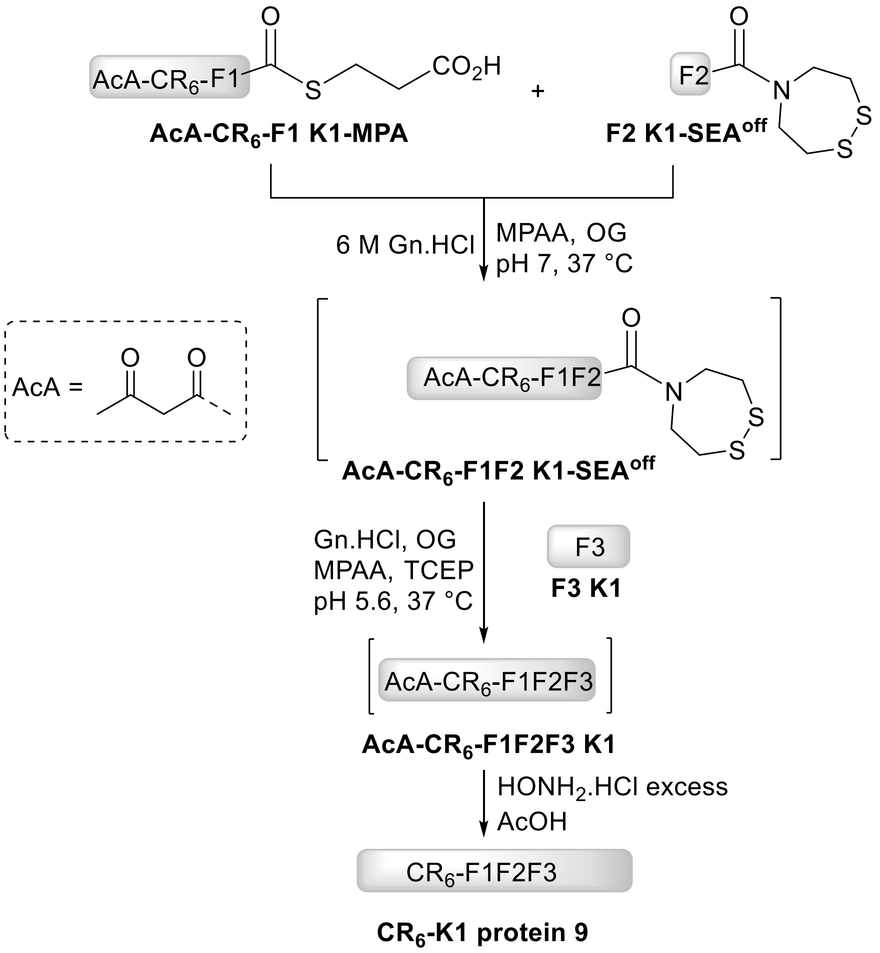

CR_6-F1 = Cys-$(Arg)_6$-ACIIGKGRSYKGTVSITKSGIK-
F2 = CQPWSSMIPHEHSFLPSSYRGKDLQENY-
F3 = CRNPRGEEGGPWCFTSNPEVRYEVCDIPQCSEVK(Biot)-NH_2

Supplementary Figure 48. Assembly of K1 polypeptide **9** in one-pot.

Preparation of the starting peptide segments

The synthesis of F2 K1-SEA^{off} and F3 K1 was already described elsewhere.[5]

Peptide thioester AcA-CR$_6$-F1 K1-MPA has been prepared according to known methods as described in **Supplementary Figure 49**.[3]

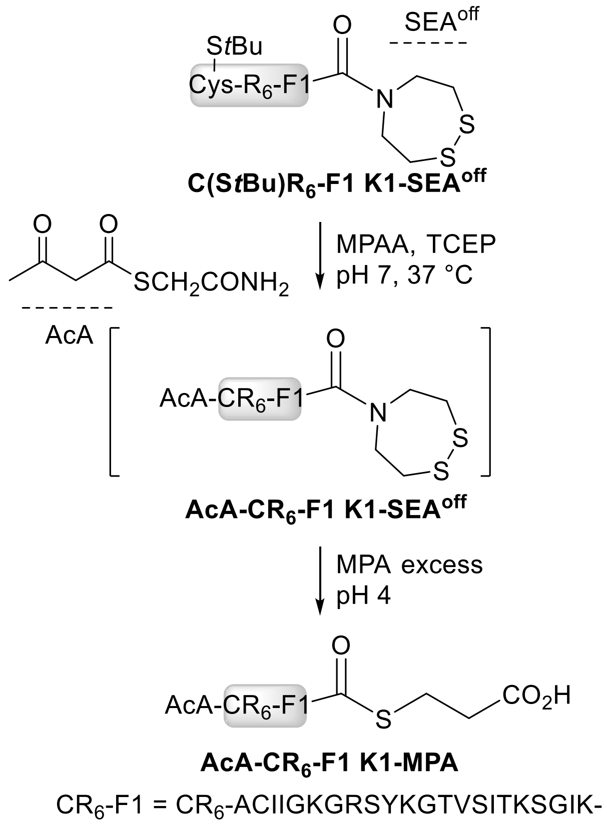

Supplementary Figure 49. Preparation of AcA-CR$_6$-F1 K1-MPA.

In brief, N-terminal segment C(S*t*Bu)R_6-F1 K1-SEA^{off} was produced by Fmoc-SPPS on SEA solid support as described elsewhere.[6] The N-terminal cysteine was protected in situ at pH 7 by adding AcA-MTG thioester according to the native chemical ligation (NCL) reaction. Temporary S*t*Bu groups present on Cys residues are removed during this step due to the reducing conditions. Then, an excess of 3-mercaptopropionic acid (MPA) was added (5% by vol) together with TCEP (100 mM). The pH of the reaction mixture was adjusted to pH 4.0 to convert the SEA peptide into a thioester peptide in one-pot. Starting from C(StBu)R_6-F1 K1-SEA^{off} (10 mg, 2.01 µmol), the purification of the crude mixture by RP-HPLC (C18 XBridge column, 50 °C, 215 nm, 6 mL min^{-1}, eluent A = water containing 0.1% v/v TFA, eluent B = CH_3CN containing 0.1% v/v of TFA, 0 to 32% B in 50 min) furnished 4.1 mg of purified AcA-CR_6-F1 K1-MPA (0.87 µmol, 43.3%).

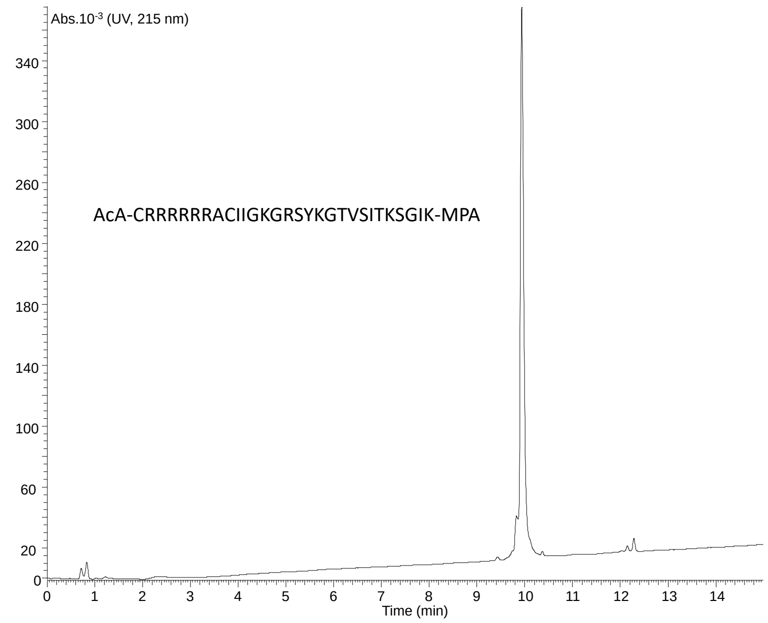

Supplementary Figure 50. UPLC-MS analysis of purified AcA-CR_6-F1 K1-MPA. LC trace. Eluent A 0.1% TFA in water, eluent B 0.1% TFA in CH_3CN. BEH C18 (300 Å, 1.7 µm, 2.1 × 100 mm) column, gradient 0-40% B in 15 min, 0.4 mL min^{-1}, 50 °C, detection at 215 nm).

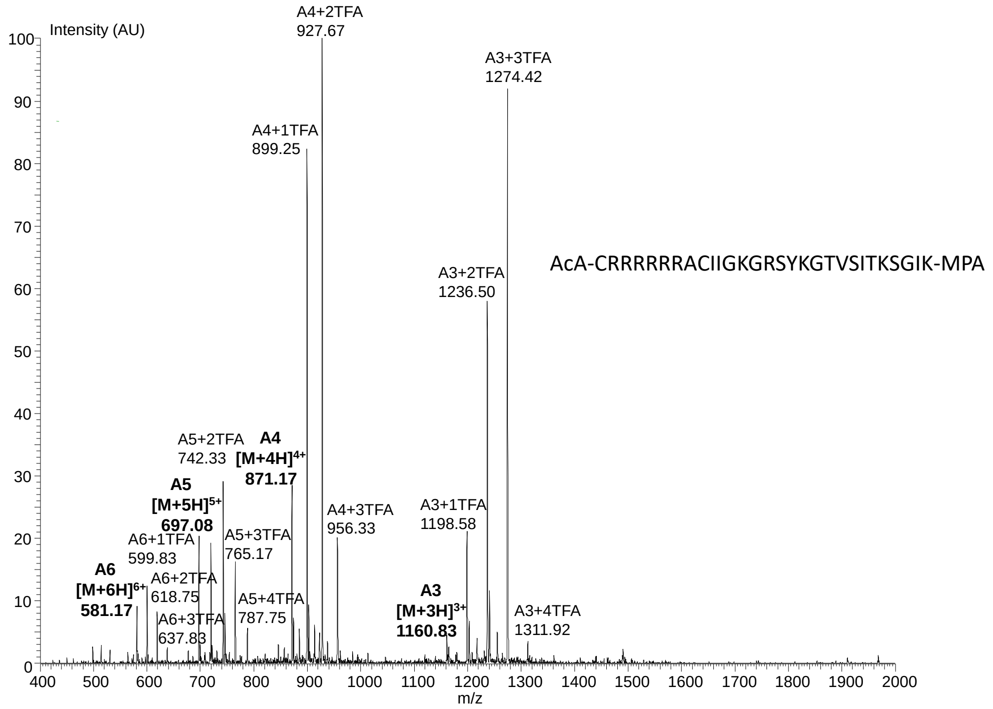

Supplementary Figure 51. MS trace of purified AcA-CR$_6$-F1 K1-MPA from UPLC-MS analysis. $[M+3H]^{3+}$ m/z calcd. (av.) 1161.06, obs. 1160.83, $[M+4H]^{4+}$ m/z calcd. (av.) 871.05, obs. 871.17, $[M+5H]^{5+}$ m/z calcd. (av.) 697.04, obs. 697.08, $[M+6H]^{6+}$ m/z calcd. (av.) 581.03, obs. 581.17. The MS spectrum is complicated by the TFA adducts formed during analysis due to the presence of basic arginine residues.

*One-pot assembly of K1 polypeptide **9***

The one-pot assembly protocol to produce the target protein was already described elsewhere.[4] The crude mixture was purified by RP-HPLC (C18 XBridge column, 50 °C, 215 nm, 6 mL min^{-1}, eluent A = water containing 0.1% v/v TFA, eluent B = CH_3CN containing 0.1% v/v of TFA, 0 to 50% B in 78 min). Starting from AcA-CR_6-F1 K1-MPA (3.73 mg, 0.79 µmoles), the assembly of the 3 segments and the removal of the AcA group in one-pot furnished 3.1 mg (0.24 µmol, 30.5%) of purified protein CR_6-K1 **9**.

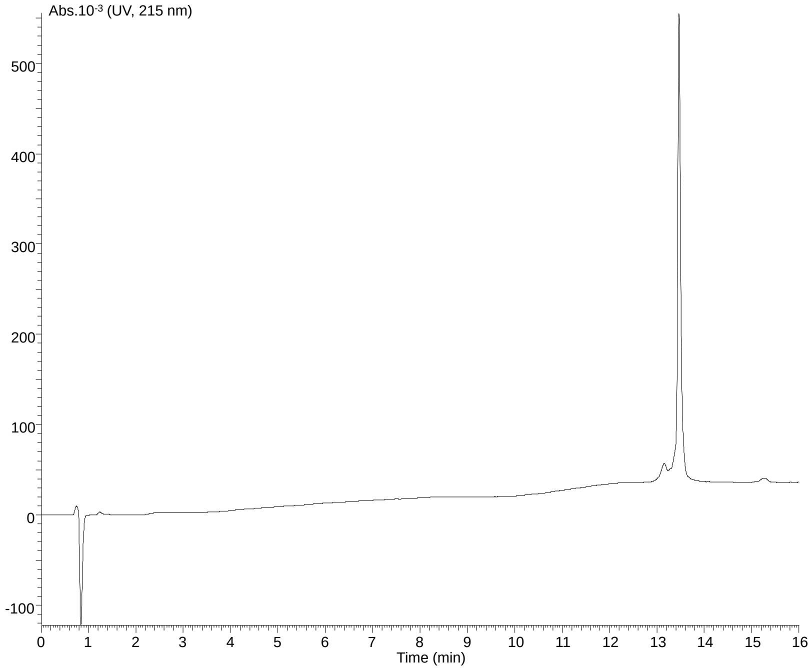

Supplementary Figure 52. UPLC-MS analysis of purified protein **9**. LC trace. Eluent A 0.1% TFA in water, eluent B 0.1% TFA in CH_3CN. BEH C18 (300 Å, 1.7 µm, 2.1 × 100 mm) column, gradient 0-70% B in 20 min, 0.4 mL min^{-1}, 50 °C, detection at 215 nm).

A)

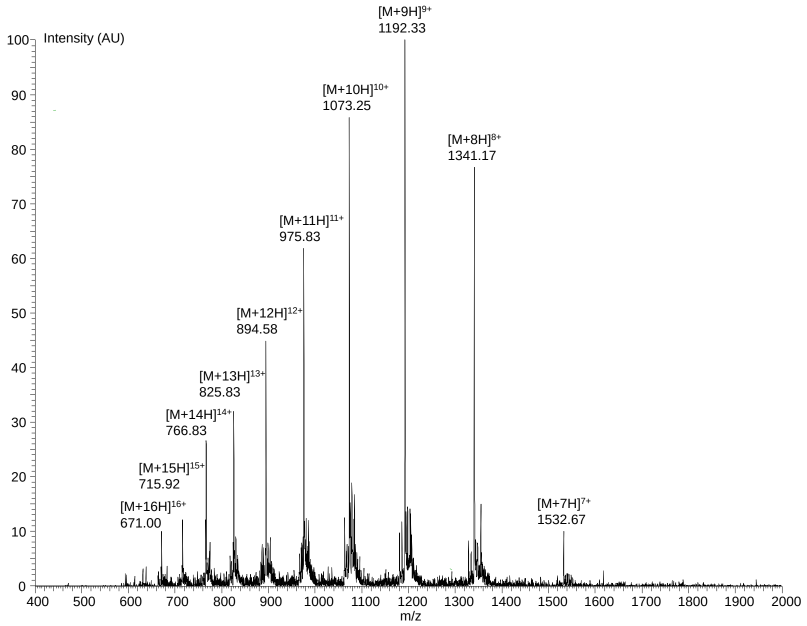

B)

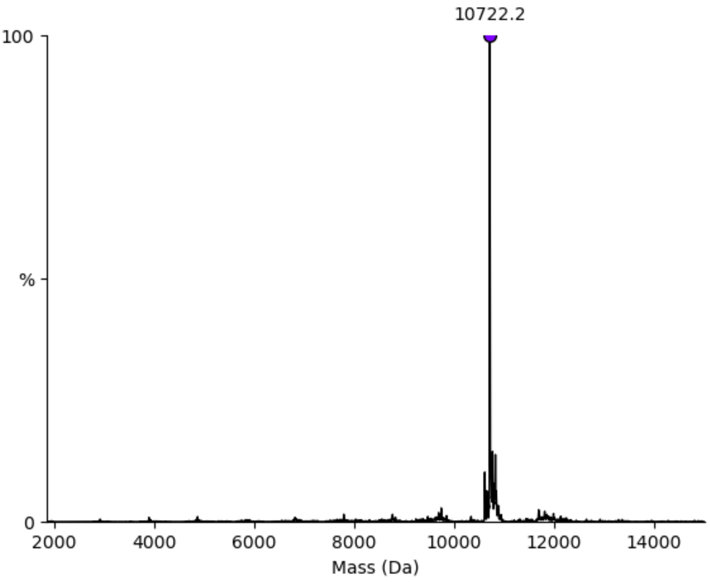

Supplementary Figure 53. Mass spectrometry analysis of purified protein **9**. A) MS trace obtained using a deionization temperature of 70 °C. $[M+7H]^{7+}$ m/z calcd. (av.) 1532.61, obs. 1532.67, $[M+8H]^{8+}$ m/z calcd. (av.) 1341.16, obs. 1341.17, $[M+9H]^{9+}$ m/z calcd. (av.) 1192.25, obs. 1192.33, $[M+10H]^{10+}$ m/z calcd. (av.) 1073.13, obs. 1073.25, $[M+11H]^{11+}$ m/z calcd. (av.) 975.66, obs. 975.83, $[M+12H]^{12+}$ m/z calcd. (av.) 894.44, obs. 894.58, $[M+13H]^{13+}$ m/z calcd. (av.) 825.71, obs. 825.83, $[M+14H]^{14+}$ m/z calcd. (av.) 766.81, obs. 766.83, $[M+15H]^{15+}$ m/z calcd. (av.) 715.75, obs. 715.92, $[M+16H]^{16+}$ m/z calcd. (av.) 671.08, obs. 671.00. B) Deconvoluted MS spectrum. M av. calcd. 10721.30, obs. 10722.2.

Synthesis of Cys peptides

Synthesis of peptide CRRRRRRALKEPVHGVpSGpS-NH$_2$

The synthesis was performed on a 0.05 mmol scale using Novasyn TGR solid support. The peptide was cleaved from the solid support and deprotected using a mixture of TFA/water/TIS 92.5/2.5/5 v/v/v for 3 h and then precipitated in 100 mL of ice-cold Et_2O/heptane 1/1 v/v. The peptide was solubilized in water, frozen and lyophilized (15.3 mg).

The RP-HPLC purification was performed using a C18 XBridge column (50 °C, 215 nm, 6 mL min^{-1}, eluent A = water containing 0.25% v/v TFA, eluent B = CH_3CN containing 0.25% v/v of TFA, 0 to 30% B in 90 min). 14.8 mg of crude peptide furnished 0.9 mg (6.1%) of purified peptide.

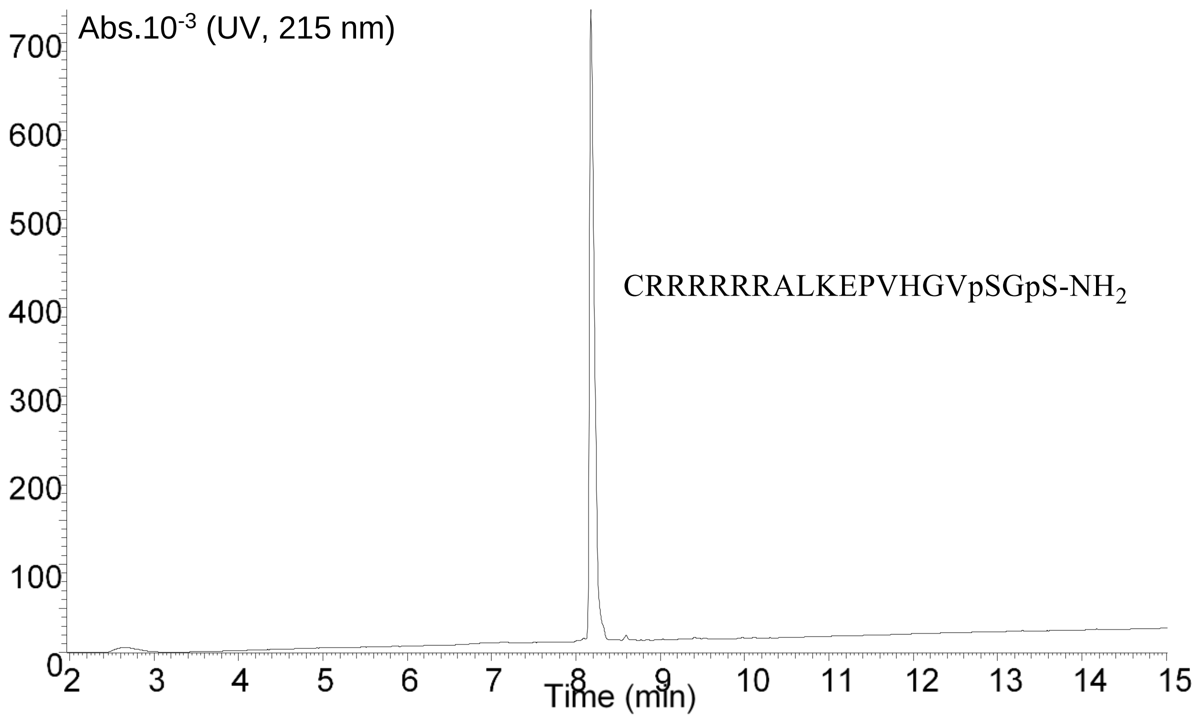

Supplementary Figure 54. UPLC-MS analysis of CRRRRRRALKEPVHGVpSGpS-NH$_2$. LC trace. Eluent A 0.1% TFA in water, eluent B 0.1% TFA in CH_3CN. C18 BEH 300 Å (1.7 µm, 2.1 × 100 mm) column, gradient 0-40% B in 15 min, 0.4 mL min^{-1}, detection at 215 nm).

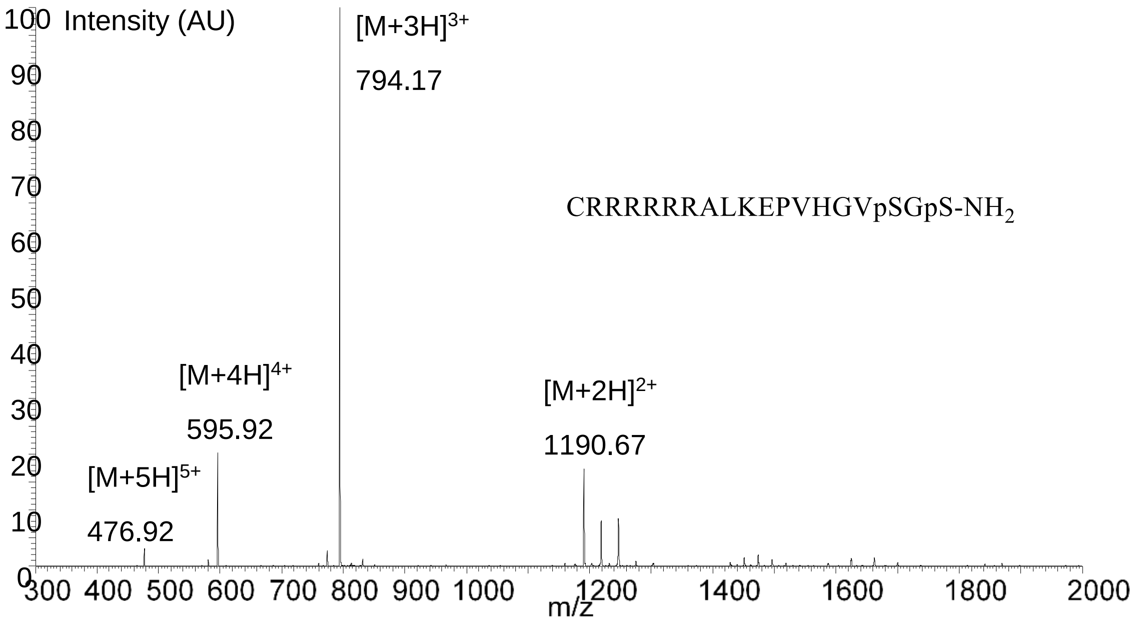

Supplementary Figure 55. MS trace of CRRRRRRALKEPVHGVpSGpS-NH_2 from UPLC-MS analysis. $[M+2H]^{2+}$ m/z calcd. (av.) 1190.79, obs. 1190.67, $[M+3H]^{3+}$ m/z calcd. (av.) 794.19, obs. 794.17, $[M+4H]^{4+}$ m/z calcd. (av.) 595.89, obs. 595.92, $[M+5H]^{5+}$ m/z calcd. (av.) 476.92, obs. 476.92.

Synthesis of peptide CRRRRRRALKEPVHGVEEGEE-NH2

The synthesis was performed on a 0.05 mmol scale using Novasyn TGR solid support. The peptide was cleaved from the solid support and deprotected using a mixture of TFA/water/TIS 92.5/2.5/5 v/v/v for 3 h and then precipitated in 100 mL of ice-cold Et_2O/heptane 1/1 v/v. The peptide was solubilized in water and directly purified.

The RP-HPLC purification was performed using a C18 XBridge column (65 °C, 215 nm, 6 mL min^{-1}, eluent A = water containing 0.1% v/v TFA, eluent B = CH_3CN containing 0.1% v/v of TFA, 0 to 30% B in 80 min) and furnished 5.1 mg of purified peptide.

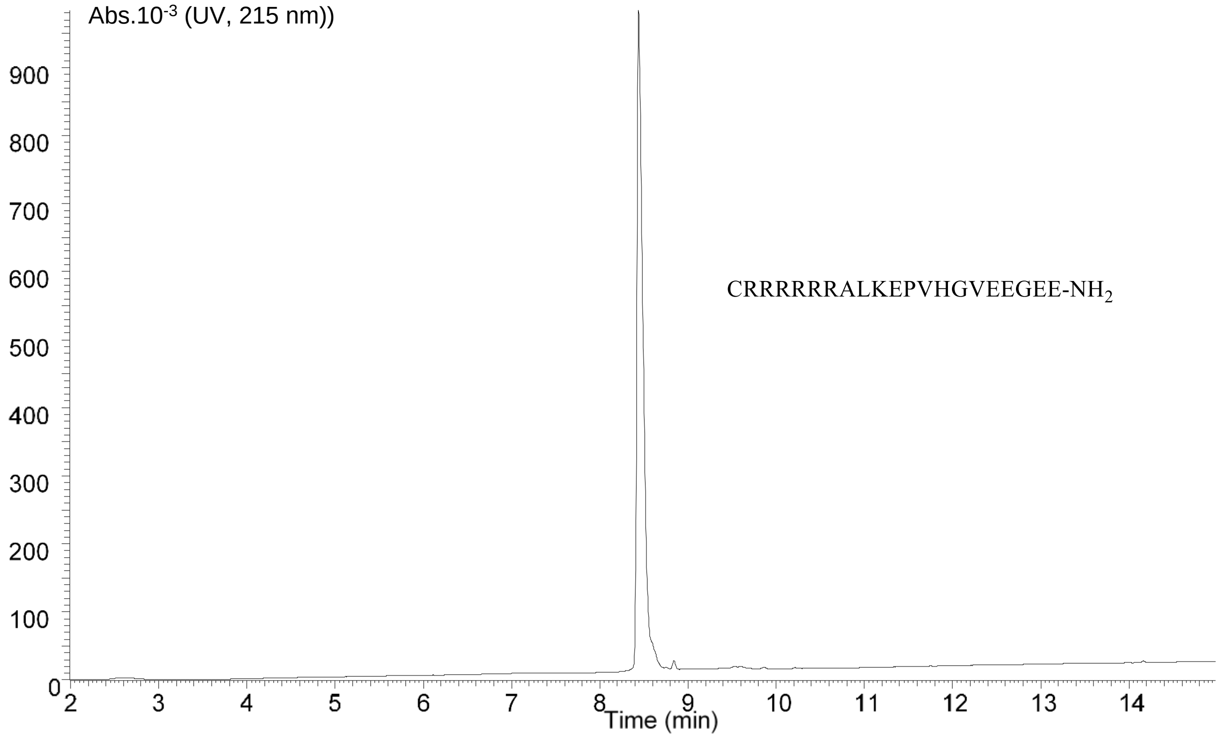

Supplementary Figure 56. UPLC-MS analysis of CRRRRRRALKEPVHGVEEGEE-NH_2. LC trace. Eluent A 0.1% TFA in water, eluent B 0.1% TFA in CH_3CN. C18 BEH 300 Å (1.7 µm, 2.1 × 100 mm) column, gradient 0-40% B in 15 min, 0.4 mL min^{-1}, detection at 215 nm).

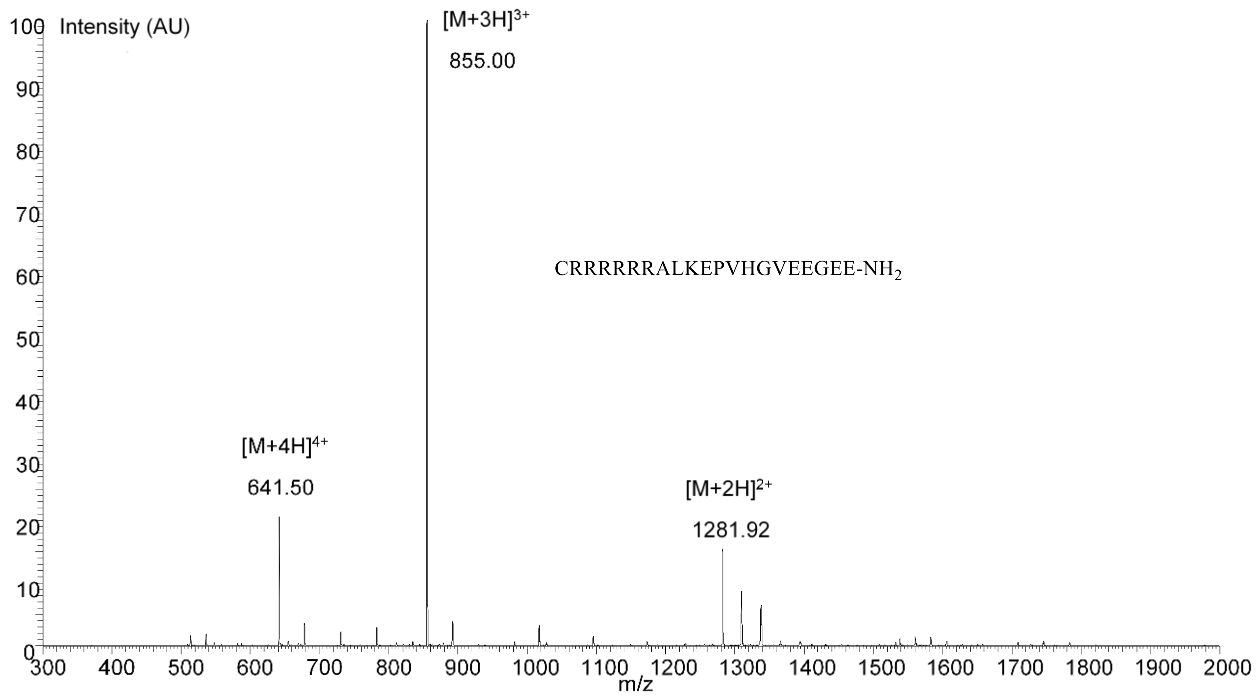

Supplementary Figure 57. MS trace of CRRRRRRALKEPVHGVEEGEE-NH_2 from UPLC-MS analysis. $[M+2H]^{2+}$ m/z calcd. (av.) 1282.45, obs. 1281.92, $[M+3H]^{3+}$ m/z calcd. (av.) 855.30, obs. 855.00, $[M+4H]^{4+}$ m/z calcd. (av.) 641.73, obs. 641.50.

Factors influencing the electrostatic assistance of peptide thioester aminolysis (Fig. 3)

Effect of peptide concentration

The reaction of peptide thioester **1a** with glycyl peptide **2a** was studied at 5 mM, 1 mM or 0.1 mM (1 equiv of each peptide).

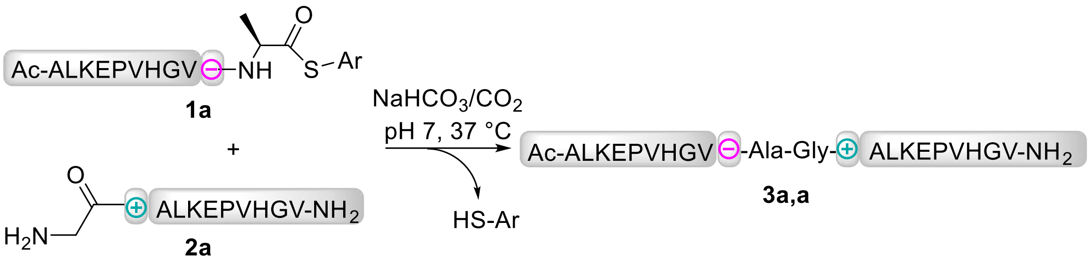

The results are presented in **Supplementary Figure 58.**

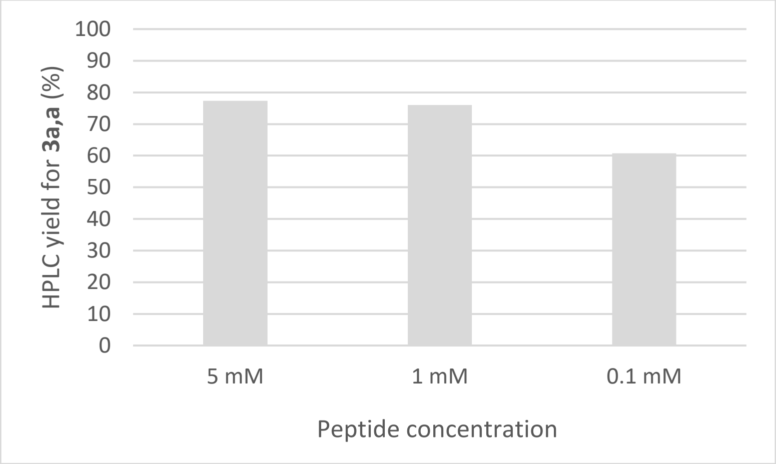

Supplementary Figure 58. Influence of peptide concentration on the HPLC yield of target ligation product **3a,a**.

Peptide reactants 5 mM

A)

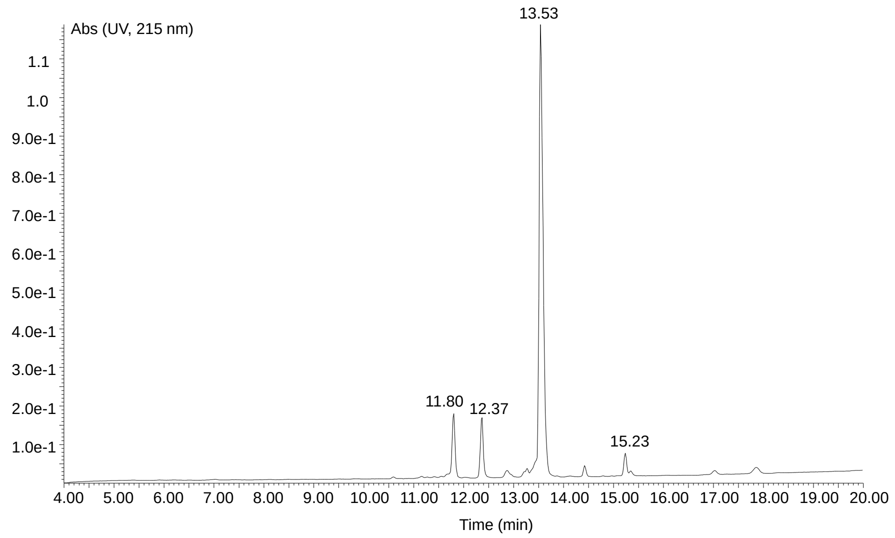

B)

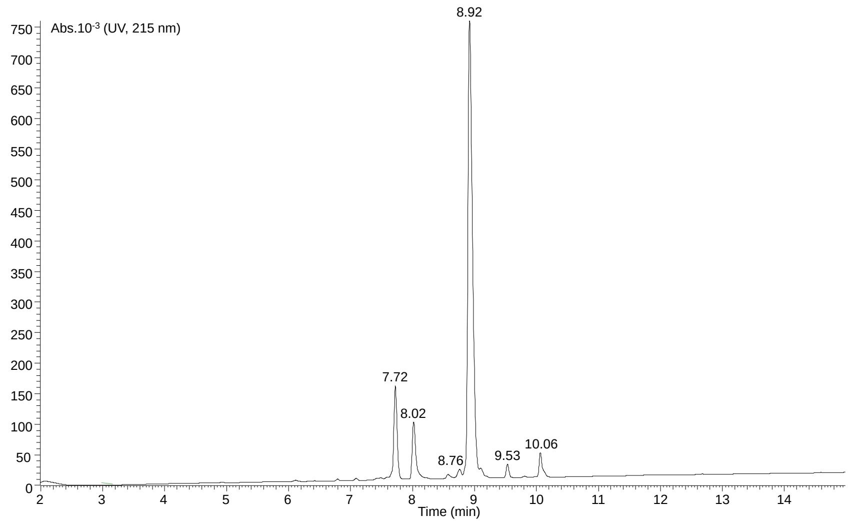

Supplementary Figure 59. Analysis of the reaction of Ac-ALKEPVHGVpSGpSA-MPAA **1a** with GRRRRRRALKEPVHGV-NH2 **2a** after 20 h (5 mM for each peptide) by LC or UPLC-MS. A) LC trace. Eluent A 0.1% TFA in water, eluent B 0.1% TFA in CH_3CN/water 4/1 v/v. C18 X bridge BEH 300 Å (5 µm, 4.6 × 250 mm) column, gradient 0-50% B in 30 min, 1 mL min^{-1}, detection at 215 nm). B) UPLC trace. Eluent A 0.1% TFA in water, eluent B 0.1% TFA in CH_3CN. C18 BEH 300 Å (1.7 µm, 2.1 × 100 mm) column, gradient 0-40% B in 15 min, 0.4 mL min^{-1}, detection at 215 nm).

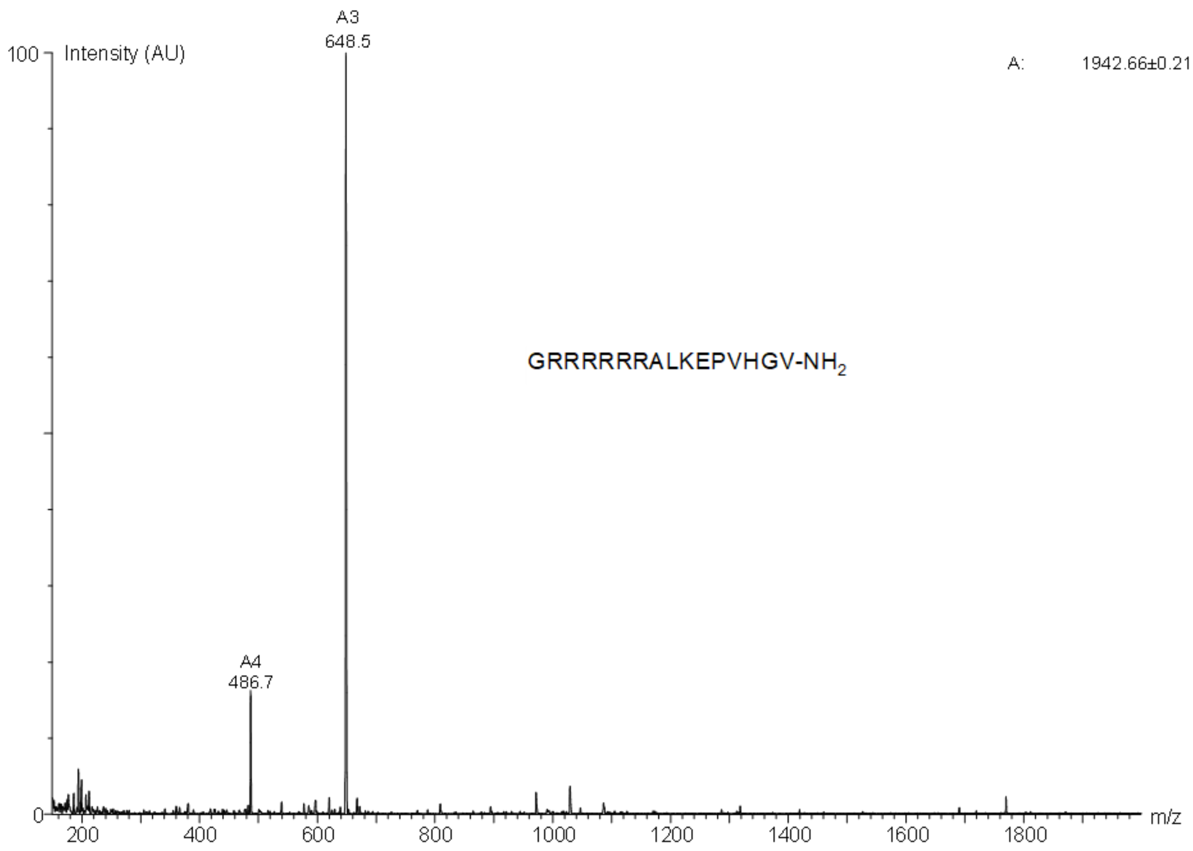

Supplementary Figure 60. MS trace of peak at Rt = 11.80 min from LC-MS analysis of the reaction of Ac-ALKEPVHGVpSGpSA-MPAA **1a** with GRRRRRRALKEPVHGV-NH_2 **2a** after 20 h (5 mM for each peptide). GRRRRRRALKEPVHGV-NH_2 **2a**: $[M+3H]^{3+}$ m/z calcd. (av.) 648.44, obs. 648.5, $[M+4H]^{4+}$ m/z calcd. (av.) 486.58, obs. 486.7.

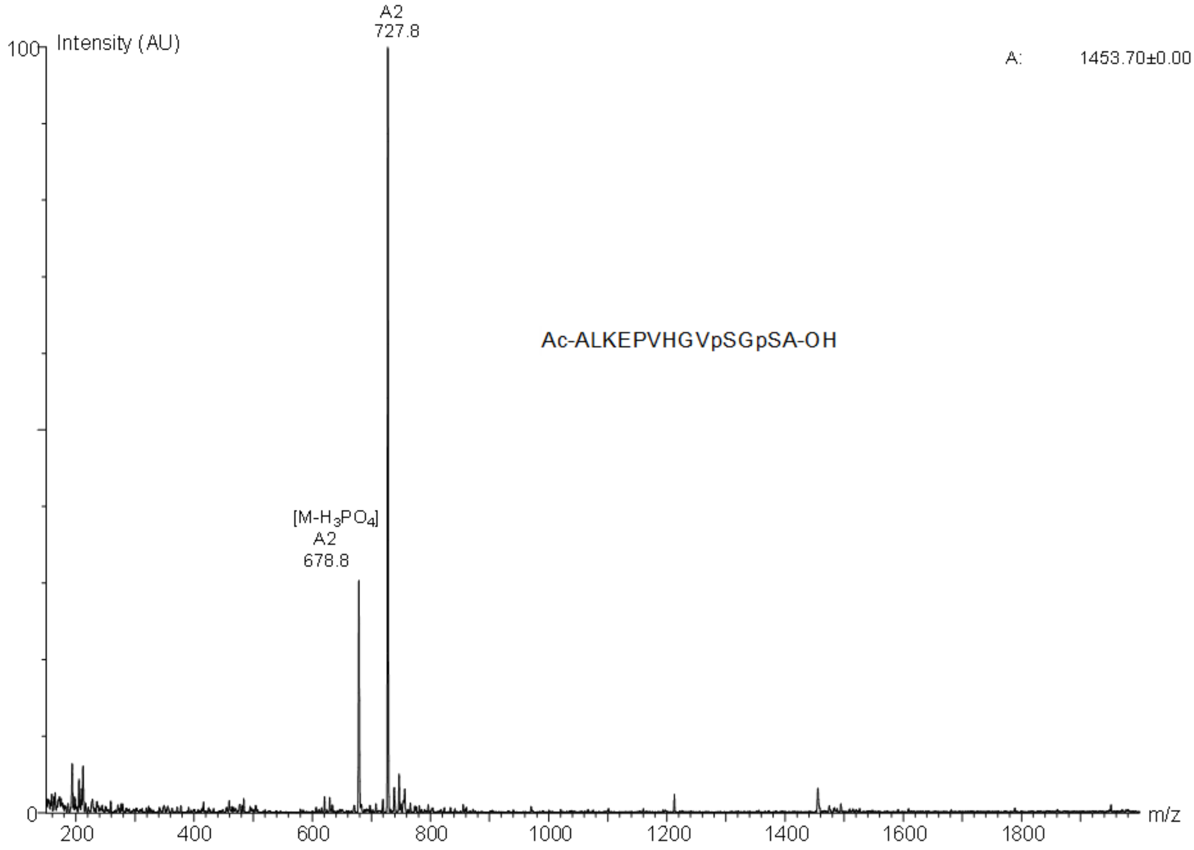

Supplementary Figure 61. MS trace of peak at Rt = 12.37 min from LC-MS analysis of the reaction of peptide thioester Ac-ALKEPVHGVpSGpSA-MPAA **1a** with GRRRRRRALKEPVHGV-NH_2 **2a** after 20 h (5 mM for each peptide). Peptide thioester hydrolysis byproduct Ac-ALKEPVHGVpSGpSA-OH: $[M+2H]^{2+}$ m/z calcd. (av.) 727.70, obs.727.8.

A)

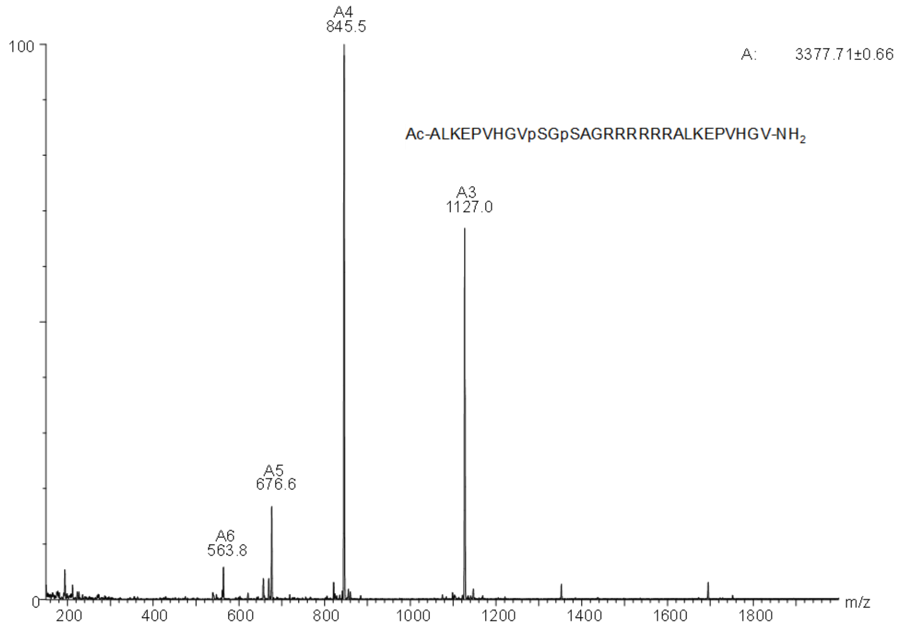

B)

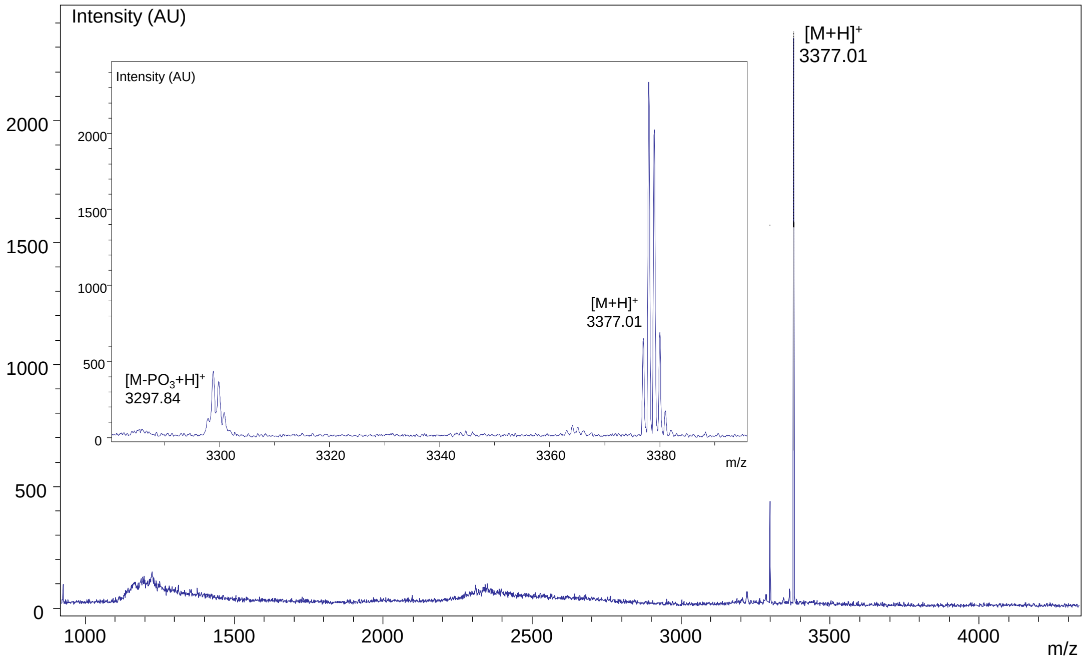

Supplementary Figure 62. Analysis of the peak at Rt = 13.53 min, target ligated peptide Ac-ALKEPVHGVpSGpSAGRRRRRRALKEPVHGV-NH_2 **3a,a** by mass spectrometry. A) MS trace of peak at Rt = 13.53 min from LC-MS analysis of target ligated peptide Ac-ALKEPVHGVpSGpSAGRRRRRRALKEPVHGV-NH_2 **3a,a**. $[M+3H]^{3+}$ m/z calcd. (av.) 1126.90, obs. 1127.0, $[M+4H]^{4+}$ m/z calcd. (av.) 845.43, obs. 845.5, $[M+5H]^{5+}$ m/z calcd. (av.) 676.54, obs. 676.6, $[M+6H]^{6+}$ m/z calcd. (av.) 563.95, obs. 563.8. B) MALDI-TOF analysis of target ligation peptide **3a,a**. Matrix alpha-cyano-4-hydroxycinnaminic acid, positive detection mode, $[M+H]^{+}$ m/z calcd. (monoisotopic) 3376.78, found 3377.01.

A)

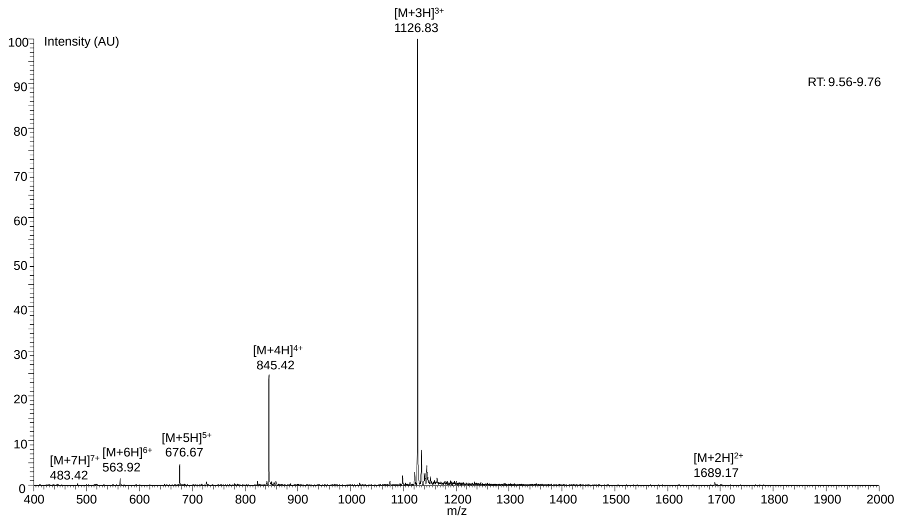

B)

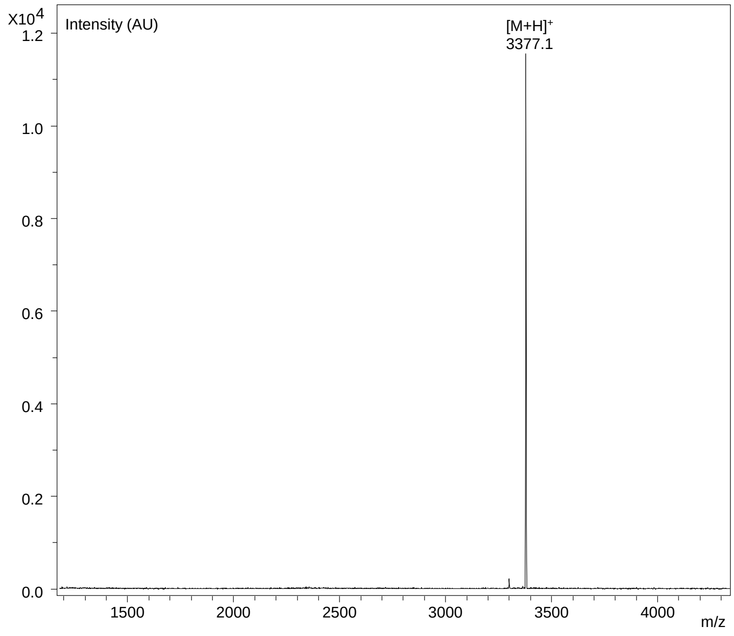

Supplementary Figure 63. Analysis of branched peptide byproduct my mass spectrometry. A) MS trace of peak at Rt = 9.53 min from LC-MS analysis of the reaction of peptide thioester Ac-ALKEPVHGVpSGpSA-MPAA **1a** and GRRRRRRALKEPVHGV-NH_2 **2a** at 5 mM. Branched product GRRRRRRALK(Ac-ALKEPVHGVpSGpSA)EPVHGV-NH_2: $[M+2H]^{2+}$ m/z calcd. (av.) 1689.85, obs. 1689.17, $[M+3H]^{3+}$ m/z calcd. (av.) 1126.90, obs. 1126.83, $[M+4H]^{4+}$ m/z calcd. (av.) 845.43, obs. 845.42, $[M+5H]^{5+}$ m/z calcd. (av.) 676.54, obs. 676.67, $[M+6H]^{6+}$ m/z calcd. (av.) 563.95, obs. 563.92, $[M+7H]^{7+}$ m/z calcd. (av.) 483.53, obs. 483.42. B)

MALDI-TOF analysis of branched peptide byproduct Ac-ALKEPVHGVpSGpSA | GRRRRRRALKEPVHGV-NH_2 .

Matrix 2,5-dihydroxybenzoic acid, positive detection mode, $[M+H]^+$ m/z calcd. (monoisotopic) 3376.78, found 3377.1.

Peptide reactants 1 mM

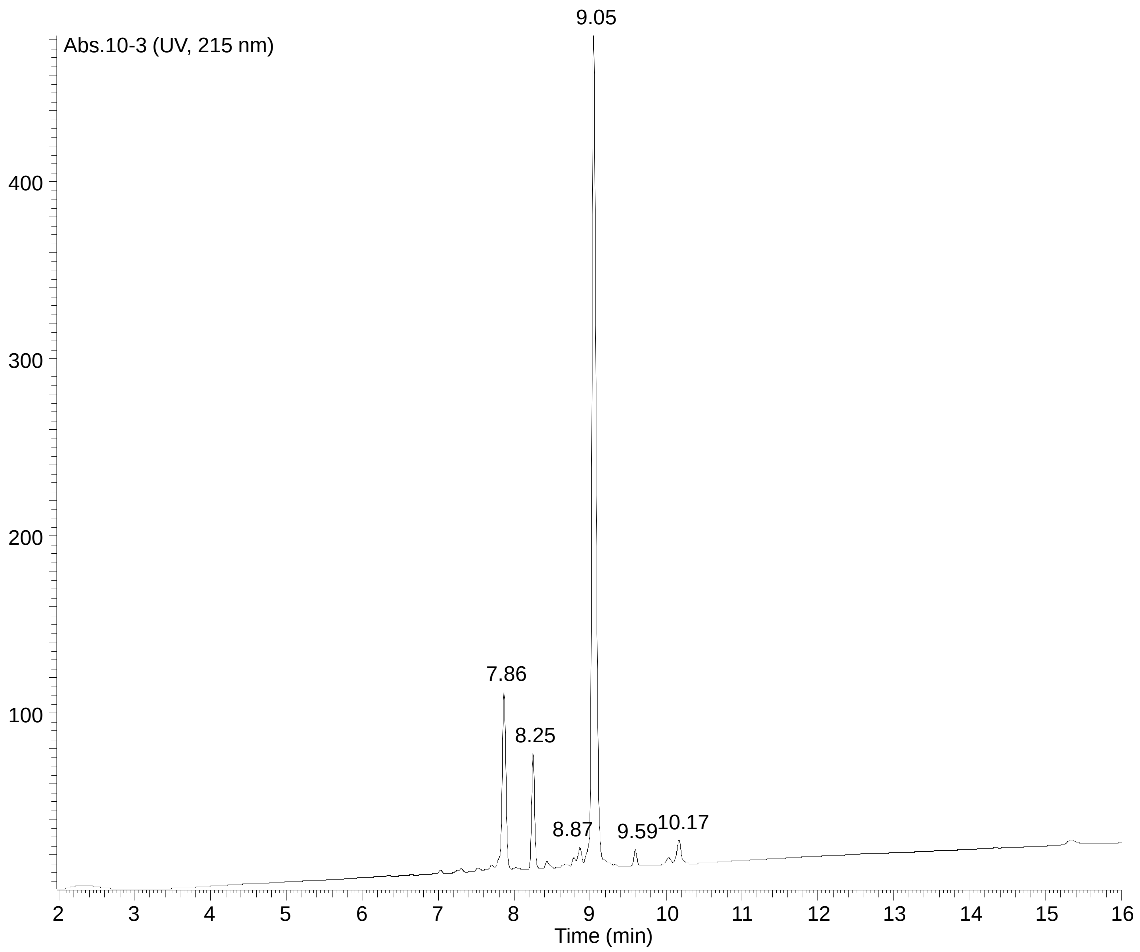

Supplementary Figure 64**.** UPLC-MS analysis of the reaction of Ac-ALKEPVHGVpSGpSA-MPAA **1a** with GRRRRRRALKEPVHGV-NH_2 **2a** (at 1 mM for each peptide) in 10 mM sodium bicarbonate/CO_2 buffer. LC trace. Eluent A 0.1% TFA in water, eluent B 0.1% TFA in CH_3CN. C18 BEH 300 Å (1.7 µm, 2.1 × 100 mm) column, gradient 0-40% B in 15 min, 0.4 mL min^{-1}, detection at 215 nm).

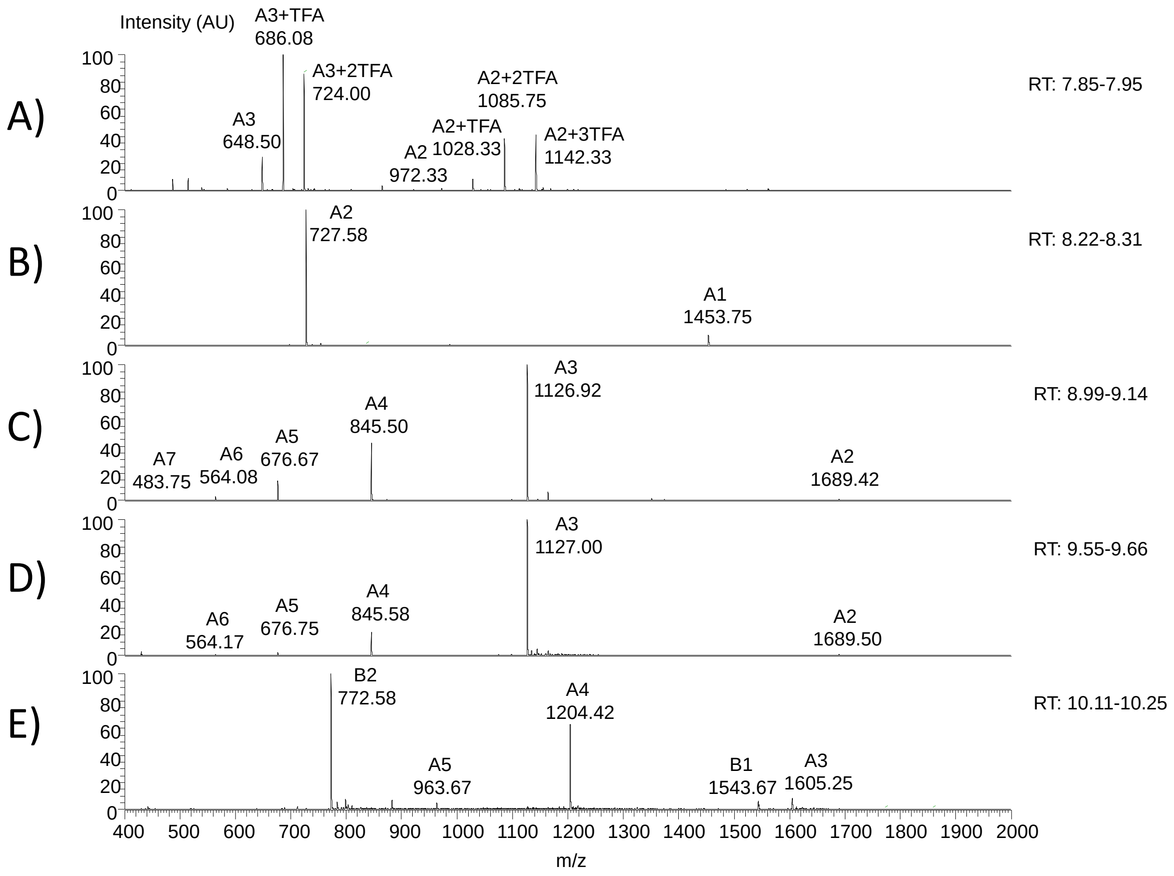

Supplementary Figure 65. MS traces from the UPLC-MS analysis of the reaction mixture between Ac-ALKEPVHGVpSGpSA-MPAA **1a** and GRRRRRRALKEPVHGV-NH_2 **2a** at 1 mM. A) Rt = 7.86 min, GRRRRRRALKEPVHGV-NH_2 **1a**: $[M+2H]^{2+}$ m/z calcd. (av.) 972.16, obs. 972.33, $[M+3H]^{3+}$ m/z calcd. (av.) 648.44, obs. 648.50. B) Rt = 8.25 min, Ac-ALKEPVHGVpSGpSA-OH: $[M+H]^{+}$ m/z calcd. (monoisotopic) 1453.61, obs. 1453.75, $[M+2H]^{2+}$ m/z calcd. (av.) 727.70, obs. 727.58. C) Rt = 9.05 min, Ac-ALKEPVHGVpSGpSAGRRRRRRALKEPVHGV-NH_2: $[M+2H]^{2+}$ m/z calcd. (av.) 1689.85, obs. 1689.42, $[M+3H]^{3+}$ m/z calcd. (av.) 1126.90, obs. 1126.92, $[M+4H]^{4+}$ m/z calcd. (av.) 845.43, obs. 845.5, $[M+5H]^{5+}$ m/z calcd. (av.) 676.54, obs. 676.67, $[M+6H]^{6+}$ m/z calcd. (av.) 563.95, obs. 564.08, $[M+7H]^{7+}$ m/z calcd. (av.) 483.53, obs. 483.75. D) Rt = 9.59 min, branched product: $[M+2H]^{2+}$ m/z calcd. (av.) 1689.85, obs. 1689.42, $[M+3H]^{3+}$ m/z calcd. (av.) 1126.90, obs. 1127.00, $[M+4H]^{4+}$ m/z calcd. (av.) 845.43, obs. 845.58, $[M+5H]^{5+}$ m/z calcd. (av.) 676.54, obs. 676.75, $[M+6H]^{6+}$ m/z calcd. (av.) 563.95, obs. 564.17. E) Rt = 10.17 min,

Ac-ALKEPVHGVpSGpSA
|
Ac-ALKEPVHGVpSGpSA-GRRRRRRALKEPVHGV-NH_2

$[M+3H]^{3+}$ m/z calcd. (av.) 1605.37, obs. 1605.25, $[M+4H]^{4+}$ m/z calcd. (av.) 1204.27, obs. 1204.42, $[M+5H]^{5+}$ m/z calcd. (av.) 963.62, obs. 963.67.

Peptide reactants 0.1 mM

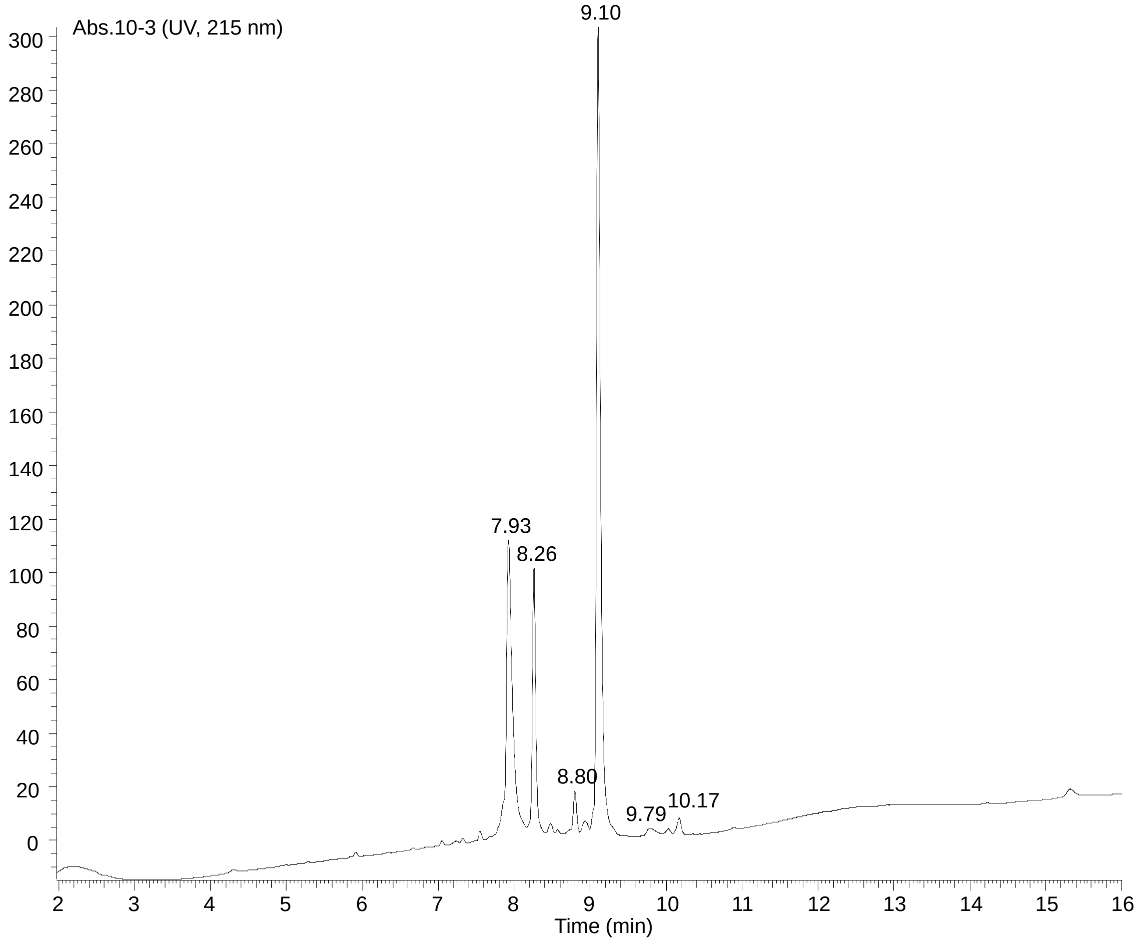

Supplementary Figure 66. UPLC analysis of the reaction of Ac-ALKEPVHGVpSGpSA-MPAA **1a** with GRRRRRRALKEPVHGV-NH_2 **2a** (at 0.1 mM for each peptide) in 10 mM sodium bicarbonate/CO_2 buffer. LC trace. Eluent A 0.1% TFA in water, eluent B 0.1% TFA in CH_3CN. C18 BEH 300 Å (1.7 µm, 2.1 × 100 mm) column, gradient 0-40% B in 15 min, 0.4 mL min^{-1}, detection at 215 nm).

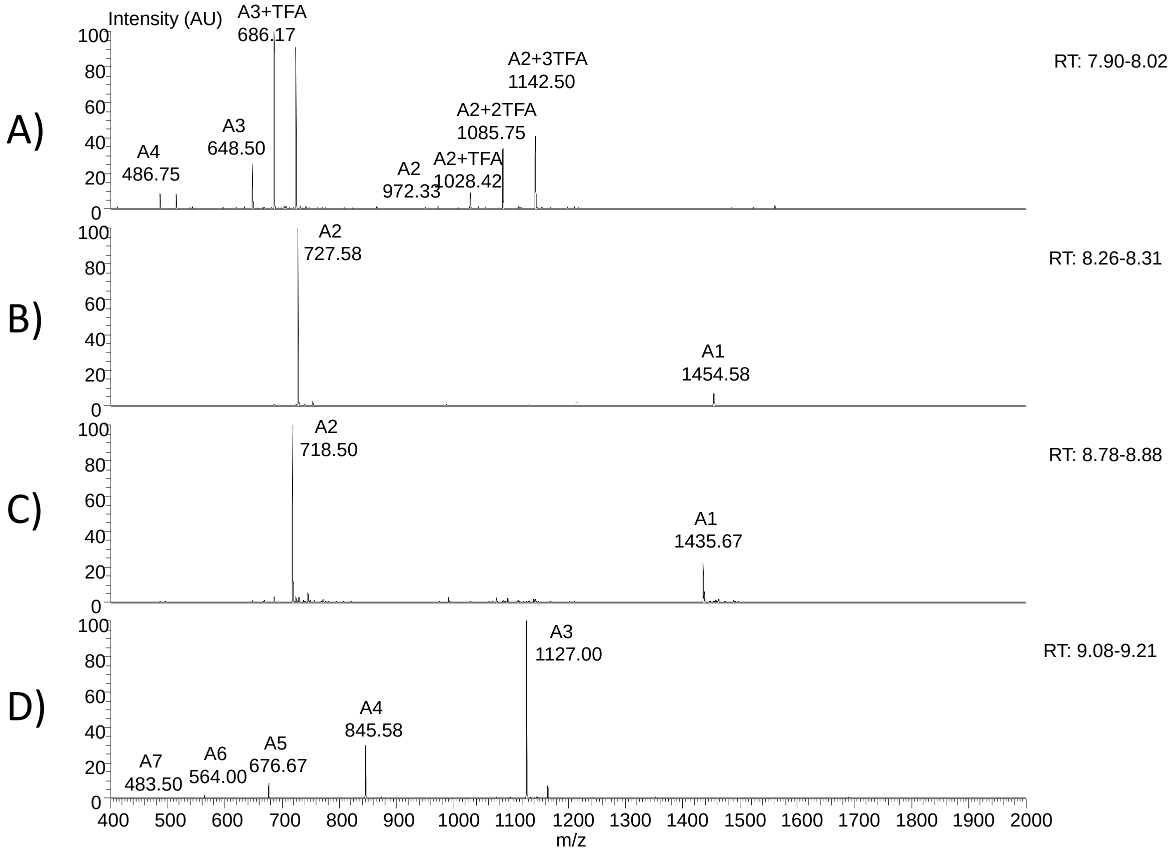

Supplementary Figure 67. MS traces from the UPLC-MS analysis of the reaction mixture between Ac-ALKEPVHGVpSGpSA-MPAA **1a** and GRRRRRRALKEPVHGV-NH_2 **2a** at 0.1 mM. MS traces. A) Rt = 7.93 min, GRRRRRRALKEPVHGV-NH_2 **2a**. $[M+2H]^{2+}$ m/z calcd. (av.) 972.16, obs. 972.33, $[M+3H]^{3+}$ m/z calcd. (av.) 648.44, obs. 648.50, $[M+4H]^{4+}$ m/z calcd. (av.) 486.58, obs. 486.75. B) Rt = 8.26 min, hydrolyzed peptide thioester Ac-ALKEPVHGVpSGpSA-OH. $[M+H]^{+}$ m/z calcd. (av.) 1454.40, obs. 1454.58, $[M+2H]^{2+}$ m/z calcd. (av.) 727.70, obs. 727.58. C) Rt = 8.80 min, cyclized peptide thioester Ac-ALKEPVHGVpSGpSA. $[M+H]^{+}$ m/z calcd. (monoisotopic) 1435.60, obs. 1435.67, $[M+2H]^{2+}$ m/z calcd. (av.) 718.69, obs. 718.50. D) Rt = 9.10 min, Ac-ALKEPVHGVpSGpSAGRRRRRRALKEPVHGV-NH_2 **3a,a**. $[M+3H]^{3+}$ m/z calcd. (av.) 1126.90, obs. 1127.00, $[M+4H]^{4+}$ m/z calcd. (av.) 845.43, obs. 845.58, $[M+5H]^{5+}$ m/z calcd. (av.) 676.54, obs. 676.67, $[M+6H]^{6+}$ m/z calcd. (av.) 563.95, obs. 564.00, $[M+7H]^{7+}$ m/z calcd. (av.) 483.53, obs. 483.50.

Proteomic analyses of the reaction products

The identity of the target ligation product **3a,a** and branched peptide byproduct formed at 5 mM was confirmed by proteomic analysis of peaks collected during LC-MS analysis.

The peaks eluting at 13.53 min (target ligation peptide **3a,a**) and 14.40 min (branched peptide byproduct) were collected and the eluent was evaporated under reduced pressure. The residue was dissolved in water (100 µL). The solution was directly spotted on a MALDI plate, mixed with the matrix and dried before analysis.

Digestion of the peak at Rt 13.53 min (target ligation peptide **3a,a**)

The residue was dissolved in water (10 µL) and 0.2 M aqueous solution of ammonium bicarbonate (2 µL) was added. Then, an aqueous solution of trypsin (0.5 µL, 0.25 µg of trypsin) was added to the sample. The mixture was incubated 30 min at room temperature and then spotted on the MALDI plate as described above.

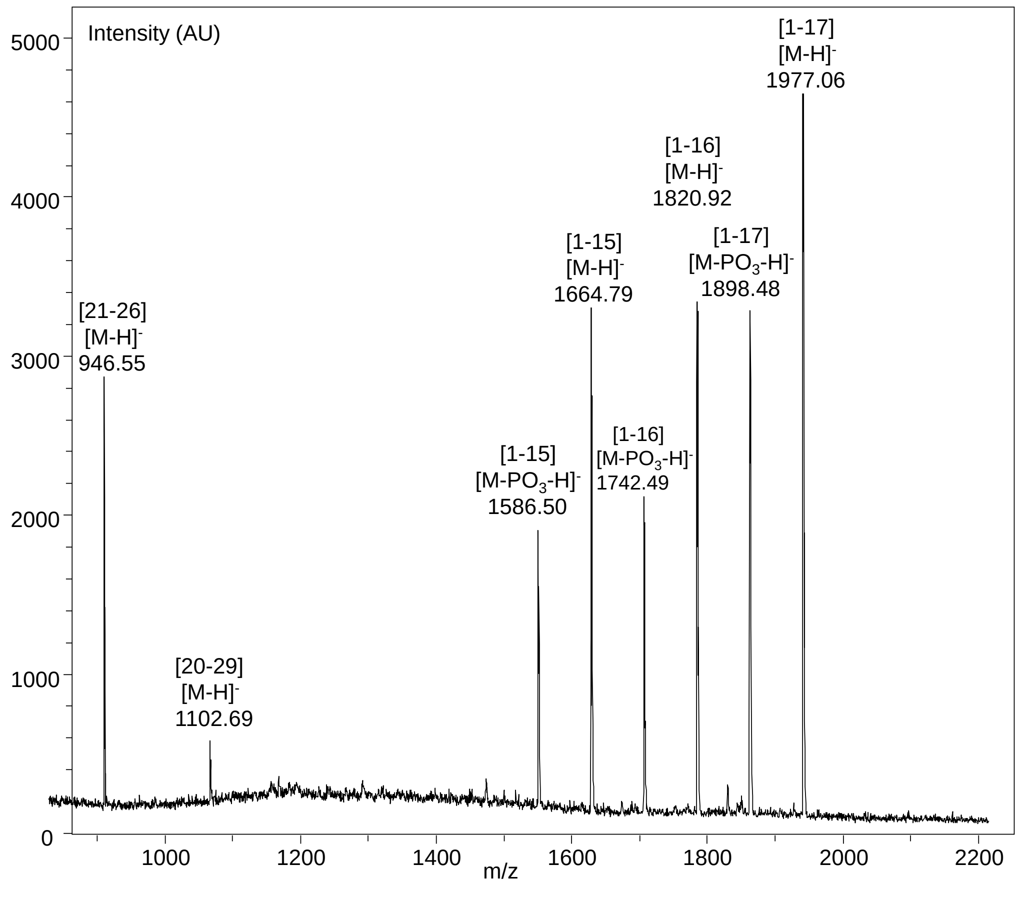

Supplementary Figure 68. MALDI-TOF analysis of the trypsic digest of target ligation product **3a,a**. Negative detection mode. Matrix alpha-cyano-4-hydroxycinnaminic acid. A series of peptide fragments coming from the digestion of **3a,a** were easily identified (amino acid residues are indicated for each peak).

Digestion of the peak at Rt 14.40 min (branched peptide byproduct)

The residue was dissolved in water (10 µL) and 0.2 M aqueous solution of ammonium bicarbonate (2 µL) was added. Then, an aqueous solution of trypsin (0.5 µL, 0.25 µg of trypsin) was added to the sample. The mixture was incubated 1 h 30 min at room temperature and then spotted on the MALDI plate as described above (**Supplementary Figure 69**).

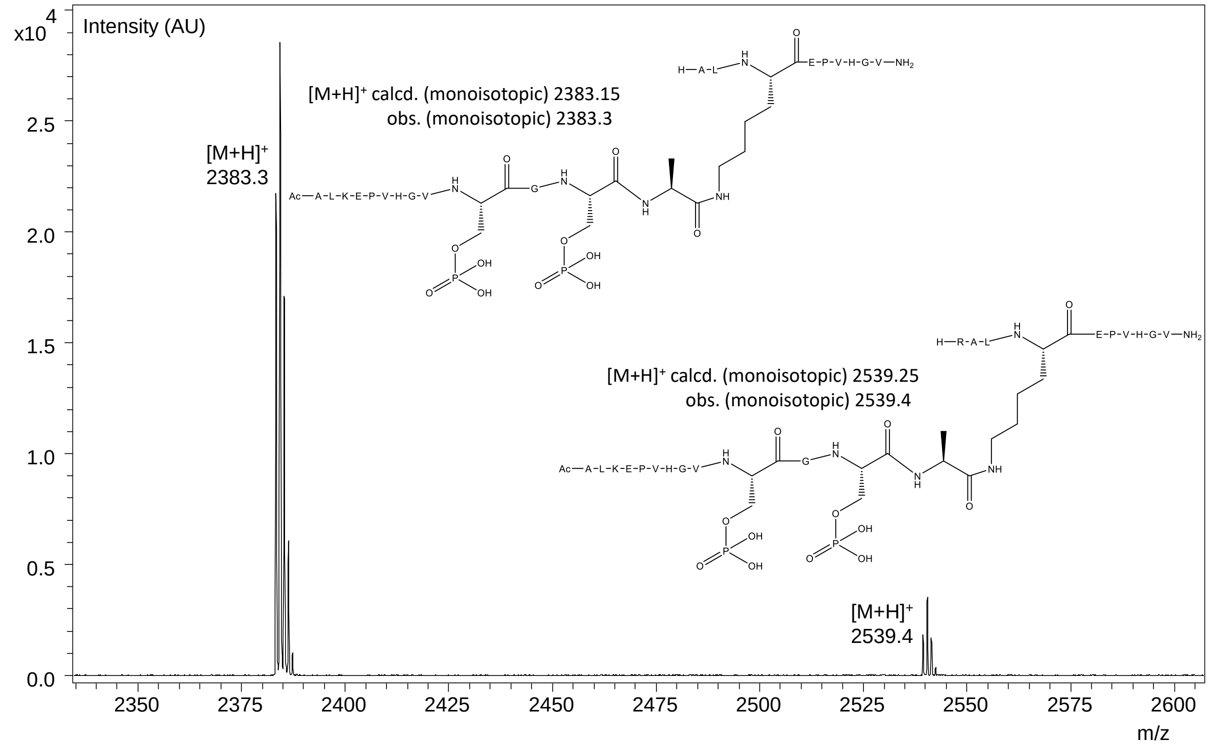

Supplementary Figure 69. MALDI-TOF analysis of the trypsic digest of branched byproduct

Ac-ALKEPVHGVpSGpSA
|
GRRRRRRALKEPVHGV-NH_2

. Positive detection mode. Matrix alpha-cyano-4-hydroxycinnaminic acid.

1d + 2a → 3d,a:

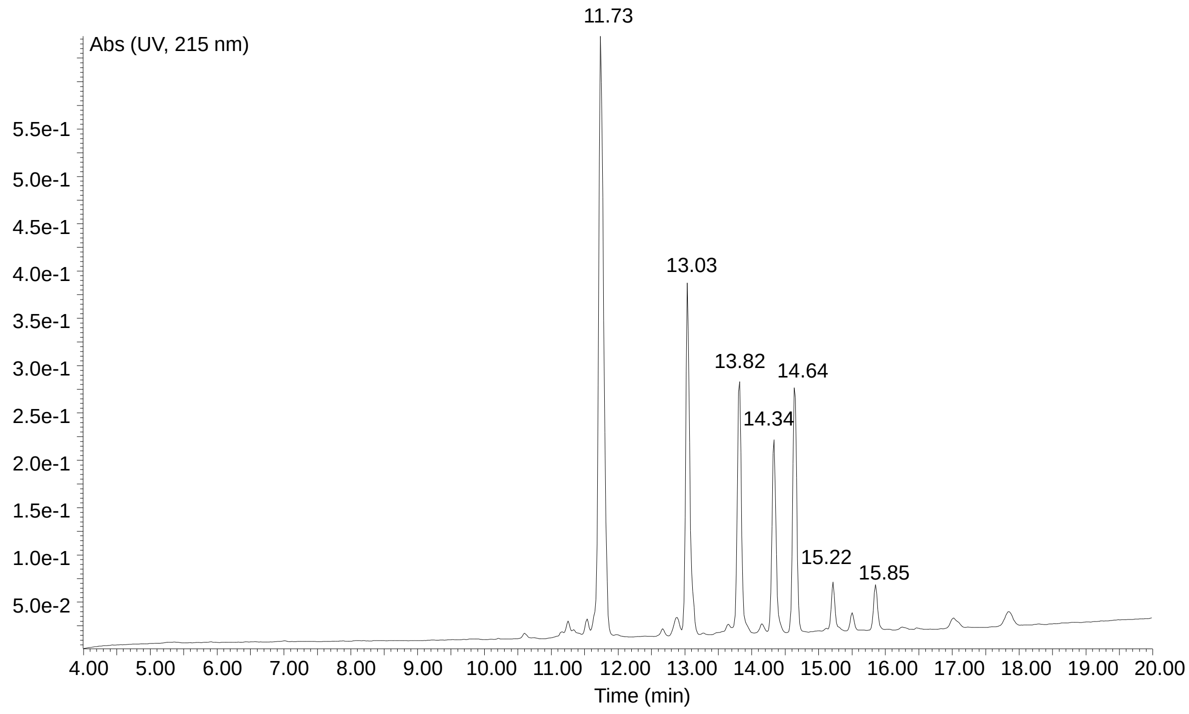

Supplementary Figure 70. LC-MS analysis of the reaction of peptide thioester Ac-ALKEPVHGVSGSA-MPAA **1d** with peptide GRRRRRRALKEPVHGV-NH_2 **2a** after 20 h (5 mM peptide concentration). LC trace. Eluent A 0.1% TFA in water, eluent B 0.1% TFA in CH_3CN. C18 X bridge BEH 300 Å (5 µm, 4.6 × 250 mm) column, gradient 0-50% B in 30 min, 1 mL min^{-1}, detection at 215 nm).

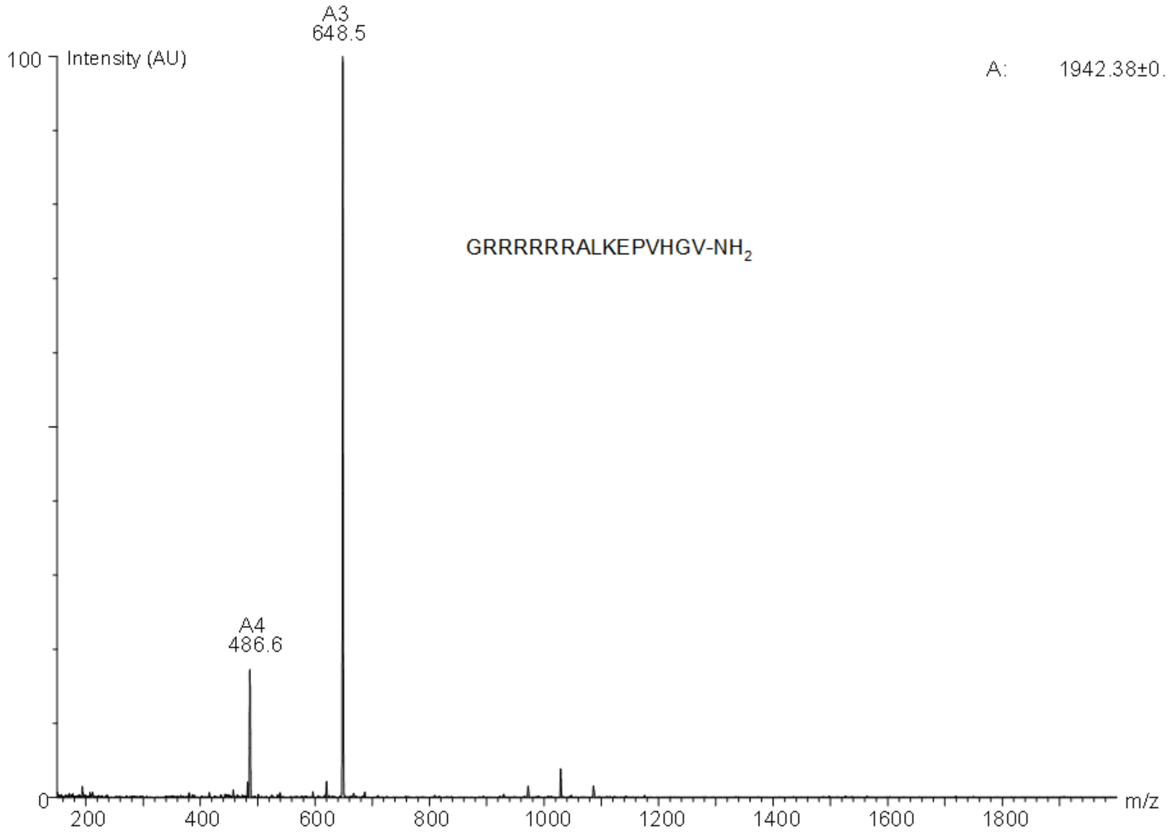

Supplementary Figure 71. MS trace of peak at Rt = 11.73 min from LC-MS analysis of the reaction of peptide thioester Ac-ALKEPVHGVSGSA-MPAA **1d** with peptide GRRRRRRALKEPVHGV-NH_2 **2a** after 20 h (5 mM peptide concentration). GRRRRRRALKEPVHGV-NH_2 **2a**: $[M+3H]^{3+}$ m/z calcd. (av.) 648.44, obs. 648.5, $[M+4H]^{4+}$ m/z calcd. (av.) 486.58, obs. 486.6.

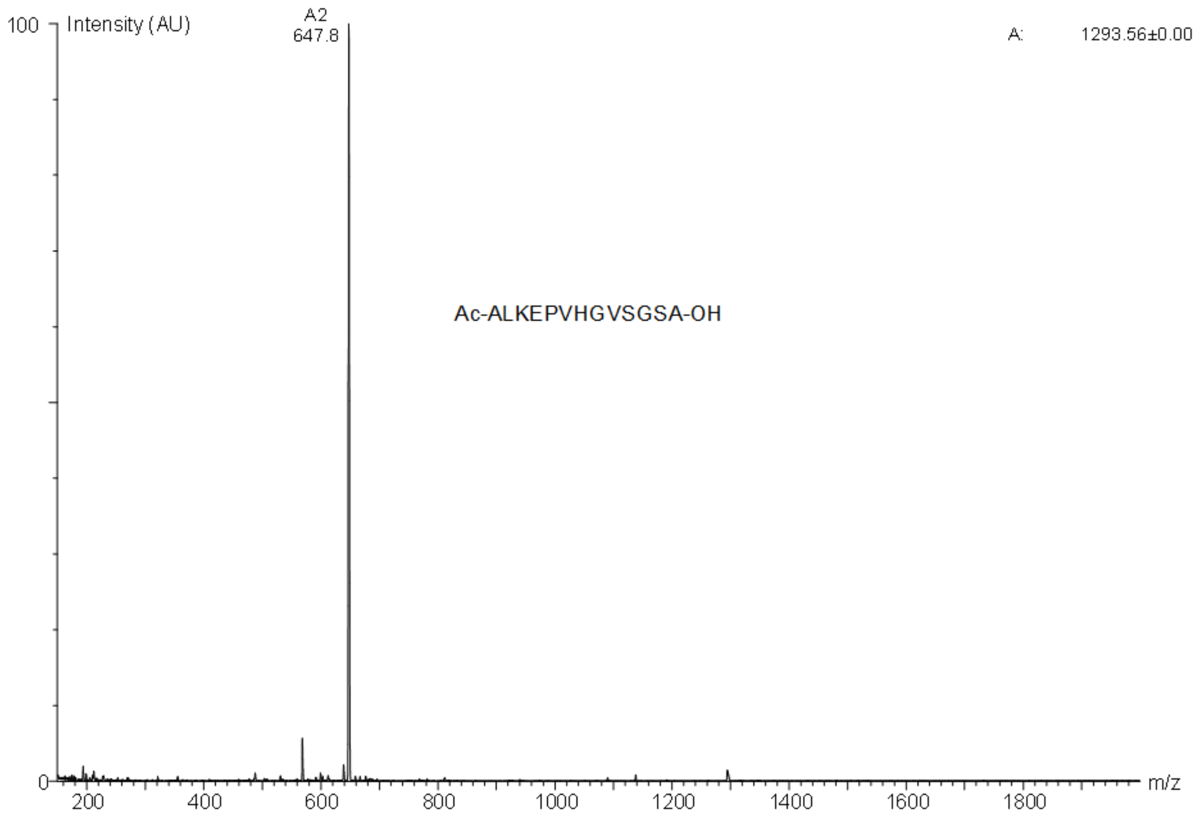

Supplementary Figure 72. MS trace of peak at Rt = 13.03 min from LC-MS analysis of the reaction of peptide thioester Ac-ALKEPVHGVSGSA-MPAA **1d** with peptide GRRRRRRALKEPVHGV-NH_2 **2a** after 20 h (5 mM peptide concentration). Peptide thioester hydrolysis byproduct Ac-ALKEPVHGVSGSA-OH: $[M+2H]^{2+}$ m/z calcd. (av.) 647.72, obs. 647.8.

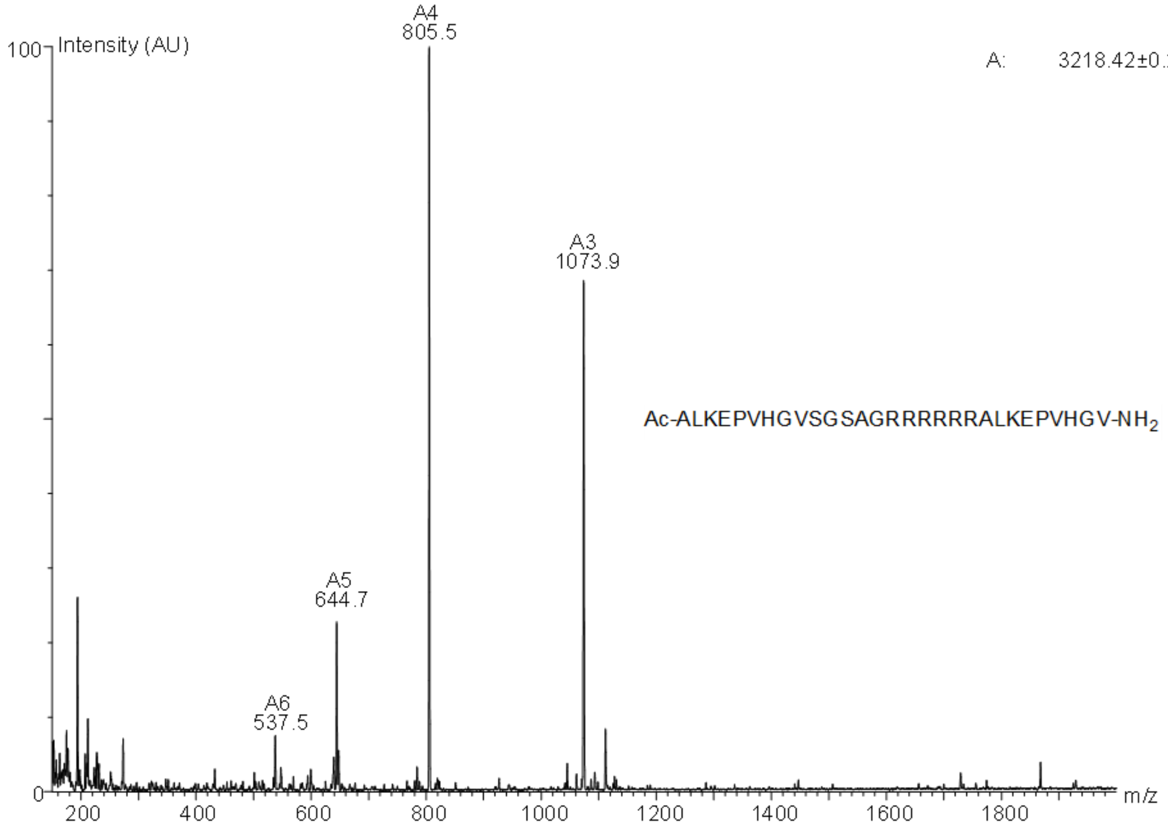

Supplementary Figure 73. MS trace of peak at Rt = 13.82 min from LC-MS analysis of the reaction of peptide thioester Ac-ALKEPVHGVSGSA-MPAA **1d** with glycyl peptide GRRRRRRALKEPVHGV-NH_2 **2a** after 20 h (5 mM peptide concentration). Target ligated peptide Ac-ALKEPVHGVSGSAGRRRRRRALKEPVHGV-NH_2 **3d,a**: $[M+3H]^{3+}$ m/z calcd. (av.) 1073.58, obs. 1073.9, $[M+4H]^{4+}$ m/z calcd. (av.) 805.44, obs. 805.5, $[M+5H]^{5+}$ m/z calcd. (av.) 644.55, obs. 644.7, $[M+6H]^{6+}$ m/z calcd. (av.) 537.29, obs. 537.5.

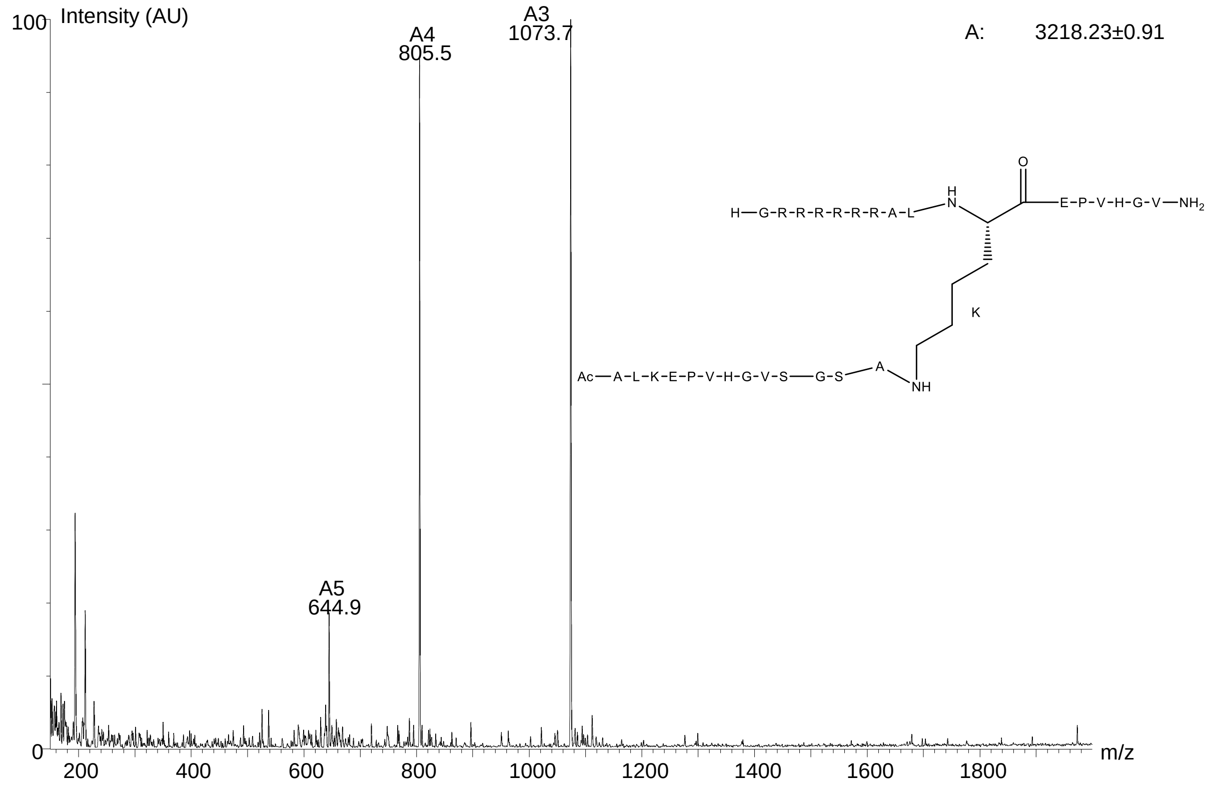

Supplementary Figure 74. MS trace of peak at Rt = 14.34 min from LC-MS analysis of the reaction of peptide thioester Ac-ALKEPVHGVSGSA-MPAA **1d** with peptide GRRRRRRALKEPVHGV-NH_2 **2a** after 20 h (5 mM peptide concentration). Branched byproduct GRRRRRRALK(Ac-ALKEPVHGVSGSA)EPVHGV-NH_2: $[M+3H]^{3+}$ m/z calcd. (av.) 1073.58, obs. 1073.7, $[M+4H]^{4+}$ m/z calcd. (av.) 805.44, obs. 805.5, $[M+5H]^{5+}$ m/z calcd. (av.) 644.55, obs. 644.9.

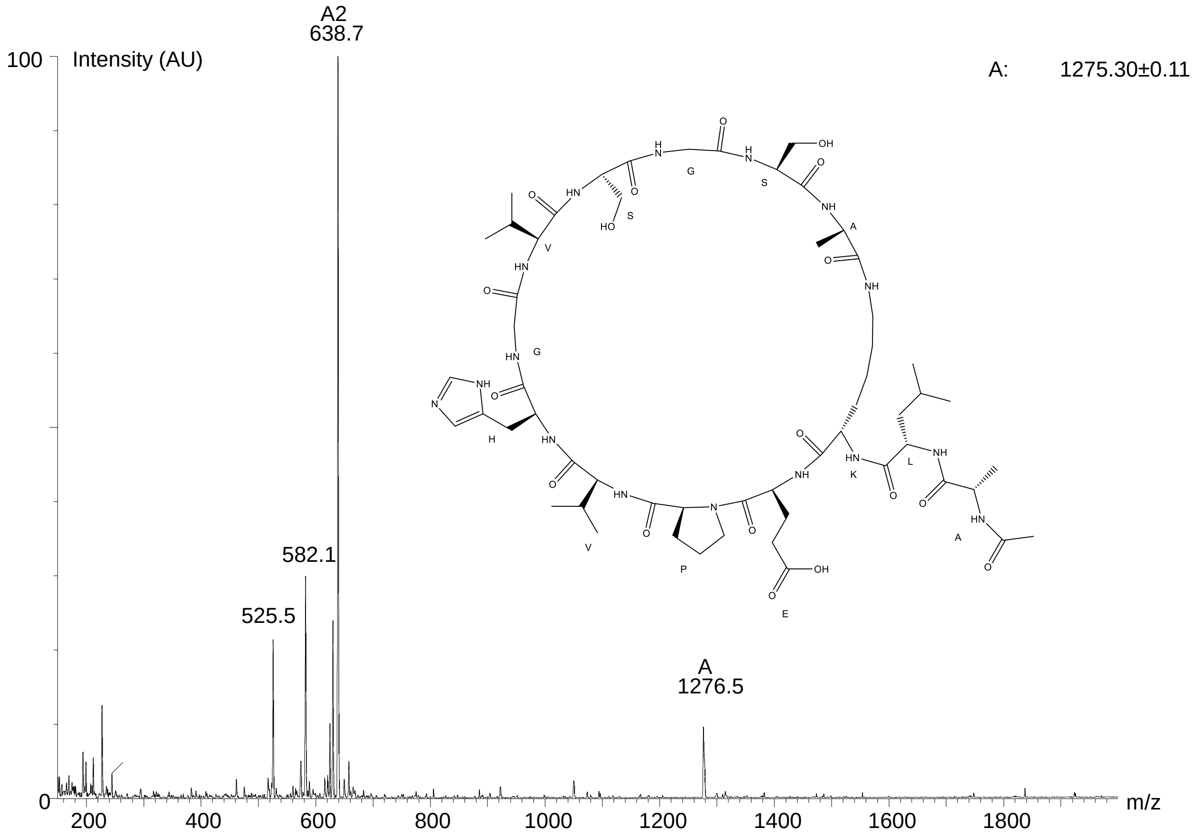

Supplementary Figure 75. MS trace of peak at Rt = 14.64 min from LC-MS analysis of the reaction of peptide thioester Ac-ALKEPVHGVSGSA-MPAA **1d** with peptide GRRRRRRALKEPVHGV-NH_2 **2a** after 20 h (5 mM peptide concentration). Peptide thioester cyclized byproduct: $[M+H]^+$ m/z calcd. (av.) 1276.43, obs. 1276.5, $[M+2H]^{2+}$ m/z calcd. (av.) 638.71, obs. 638.7.

Analyses of the reaction products

The identity of the cyclized product and ligation product was confirmed by proteomic analysis or MALDI-TOF in source fragmentation of peaks collected during LC-MS analysis.

The peaks eluting at 13.82 min (target ligation peptide **3d,a**), at 14.34 min (branched byproduct GRRRRRRALK(Ac-ALKEPVHGVSGSA)EPVHGV-NH_2) and at 14.64 min (thioester cyclization byproduct) were collected and the eluent was evaporated under reduced pressure. The residue was dissolved in water (100 µL). The solution was directly spotted on a MALDI plate, mixed with the matrix and dried before analysis.

Peak at Rt 13.82 min (target ligation peptide **3d,a**),

The residue was dissolved in water (10 µL) and 0.2 M aqueous solution of ammonium bicarbonate (2 µL) was added. Then, an aqueous solution of trypsin (0.5 µL, 0.25 µg of trypsin)

was added. The mixture was incubated at room temperature and then spotted on the MALDI plate as described above before analysis.

A)

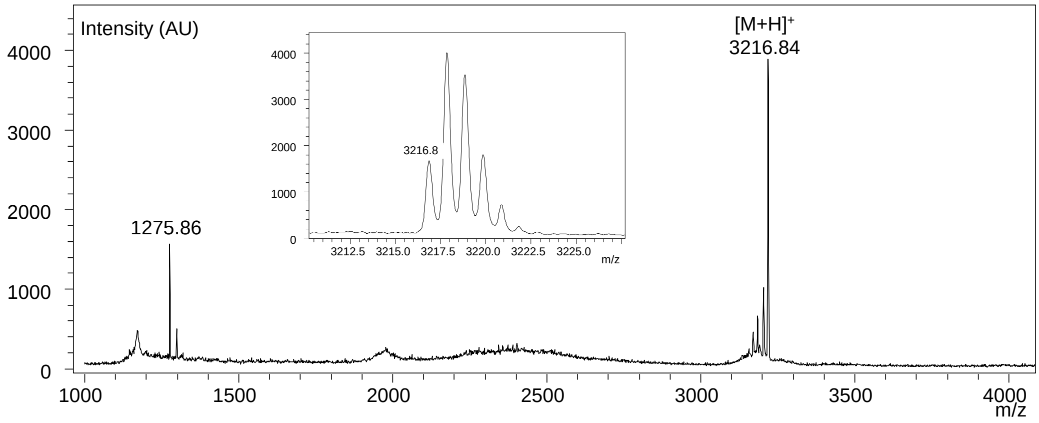

B)

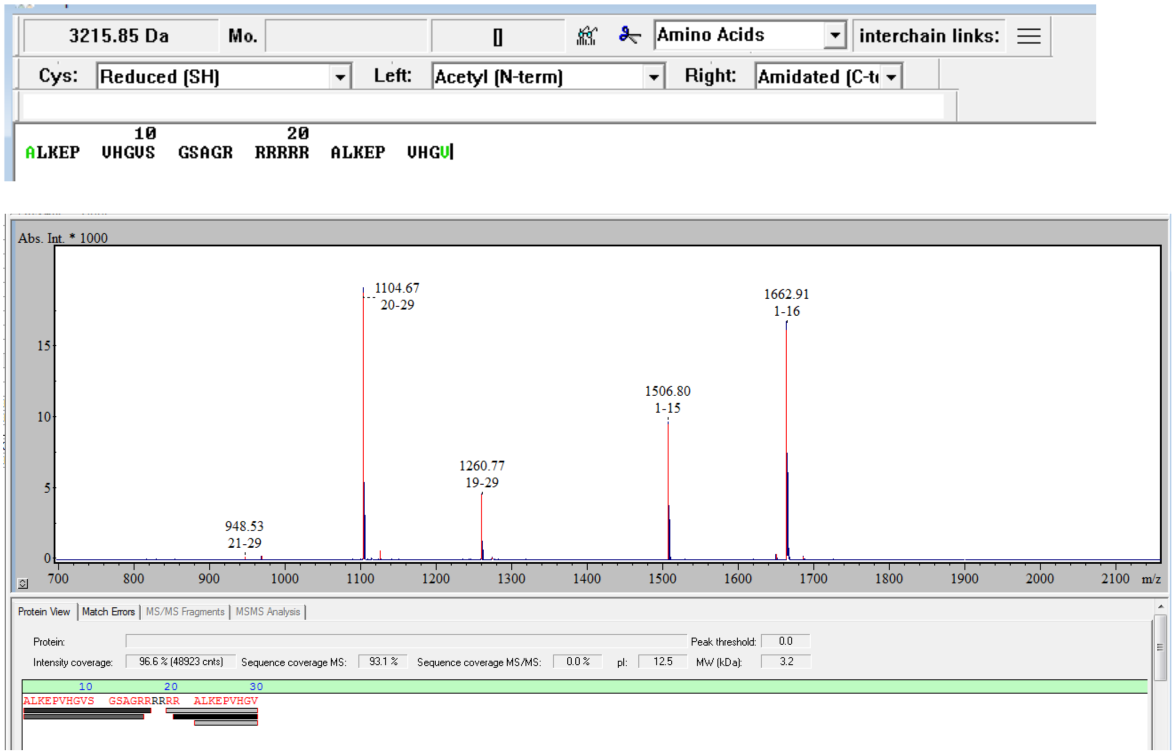

Supplementary Figure 76. Analysis of ligation product **3d,a** by mass spectrometry. A) MALDI-TOF analysis of ligation product Ac-ALKEPVHGVSGSAGRRRRRRALKEPVHGV-NH_2 **3d,a**. B) MALDI-TOF analysis of the ligation product Ac-ALKEPVHGVSGSAGRRRRRRALKEPVHGV-NH_2 **3d,a** following digestion by trypsin. A series of peptide fragments coming from the digestion of **3d,a** were easily identified (amino acid residues are indicated below the m/z values for each peak).

Peak at Rt 14.34 min (branched byproduct GRRRRRRALK(Ac-ALKEPVHGVSGSA)EPVHGV-NH_2)

The sample was treated with trypsin and analyzed as described above.

A)

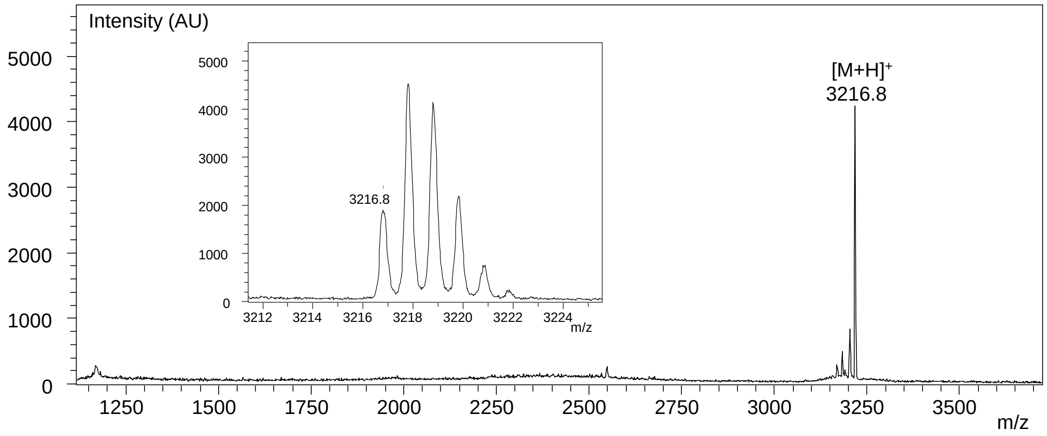

B)

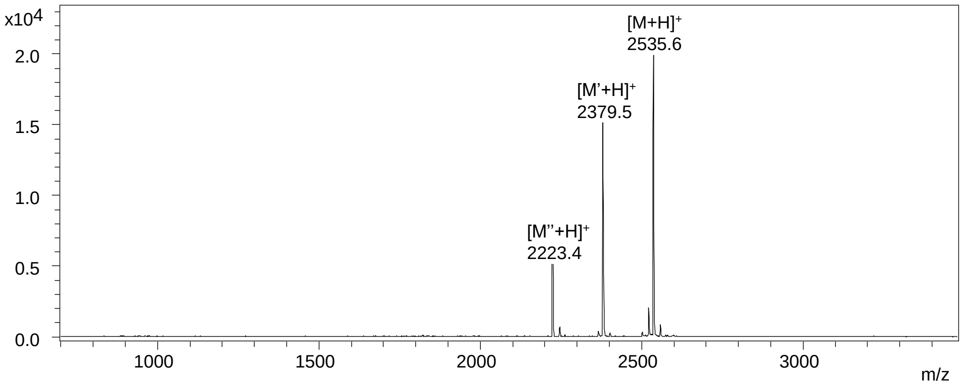

C)

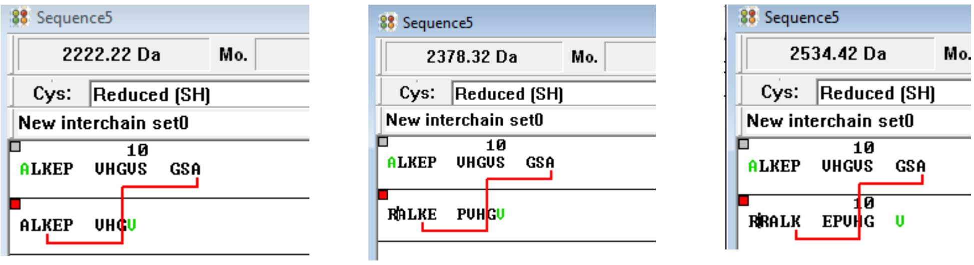

Supplementary Figure 77. Analysis of branched byproduct by mass spectrometry. A) MALDI-TOF analysis of the peak collected at Rt 14.34 min (branched byproduct). B) MALDI-TOF analysis of the trypsin digest. C) Peptide fragments generated by branched byproduct GRRRRRRALK(Ac-ALKEPVHGVSGSA)EPVHGV-NH_2.

Peak at Rt 14.64 min (peptide thioester cyclization byproduct)

A)

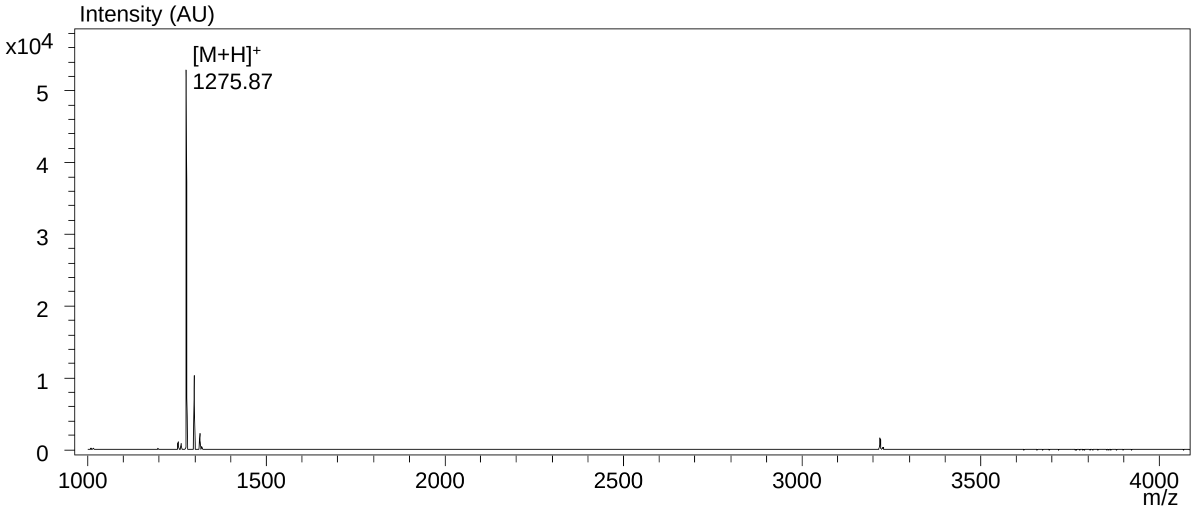

B)

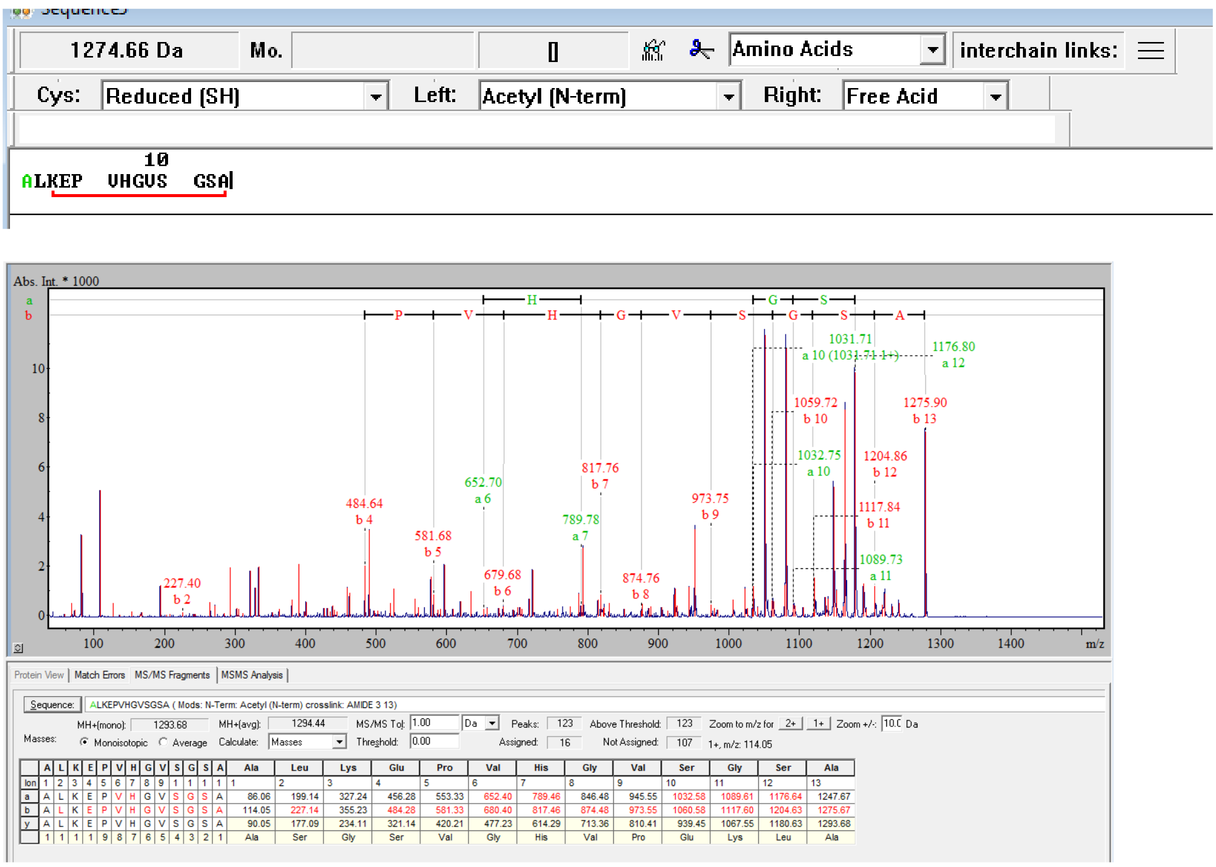

Supplementary Figure 78. MALDI-TOF analysis of the peak collected at Rt 14.64 min and corresponding to the thioester cyclization byproduct. A) MALDI-TOF spectrum. B) The in-source fragmentation of the ion at m/z 1275.87 is in accord with the proposed structure.

1a + 2g → 3a,g:

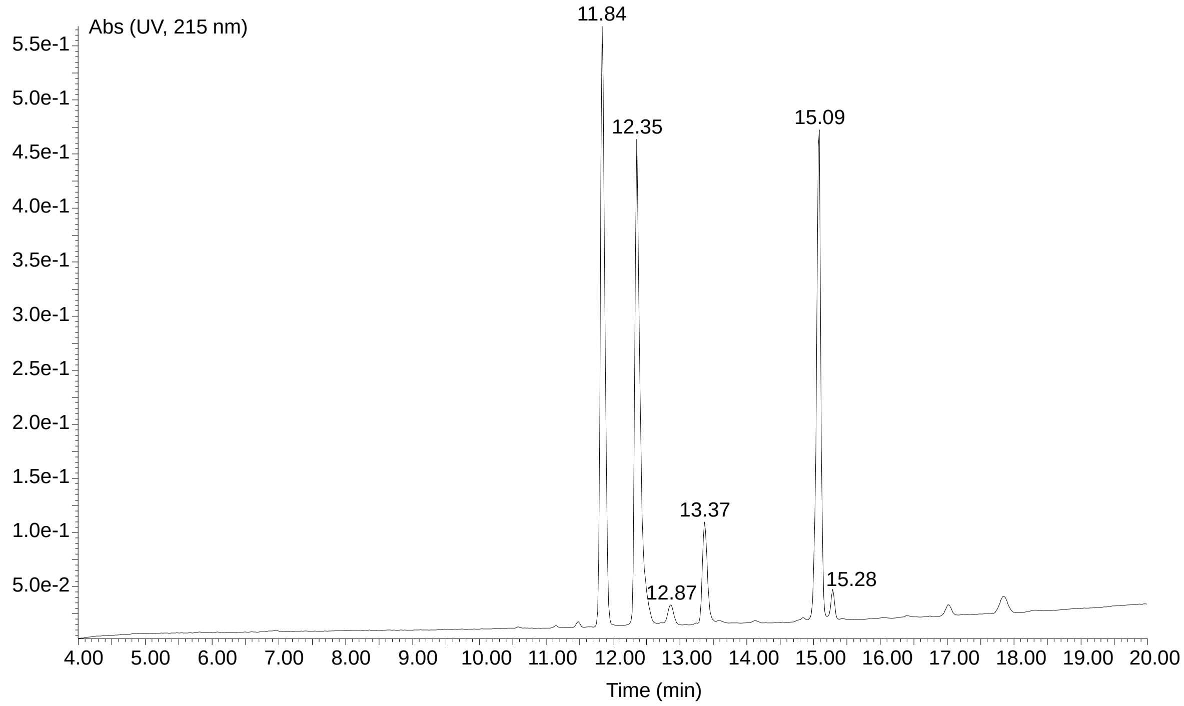

Supplementary Figure 79. LC-MS analysis of the reaction of peptide thioester Ac-ALKEPVHGVpSGpSA-MPAA **1a** with peptide GALKEPVHGV-NH_2 **2g** after 20 h (5 mM peptide concentration). LC trace. Eluent A 0.1% TFA in water, eluent B 0.1% TFA in CH_3CN. C18 X bridge BEH 300 Å (5 µm, 4.6 × 250 mm) column, gradient 0-50% B in 30 min, 1 mL min^{-1}, detection at 215 nm).

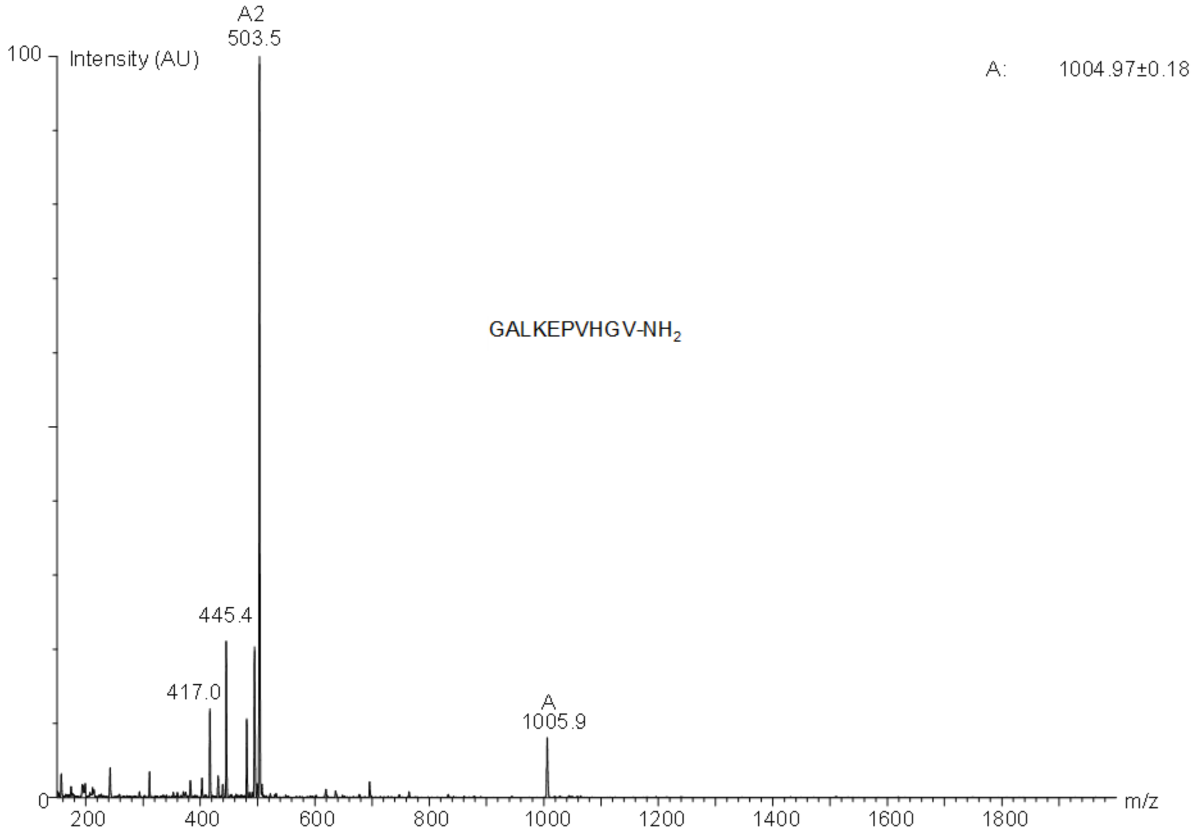

Supplementary Figure 80. MS trace of peak at Rt = 11.84 min from LC-MS analysis of the reaction of peptide thioester Ac-ALKEPVHGVpSGpSA-MPAA **1a** with peptide GALKEPVHGV-NH_2 **2g** after 20 h (5 mM peptide concentration). GALKEPVHGV-NH_2 **2g**. MS trace $[M+H]^+$ m/z calcd. (monoisotopic) 1005.58, obs. 1005.9, $[M+2H]^{2+}$ m/z calcd. (av.) 503.59, obs. 503.5.

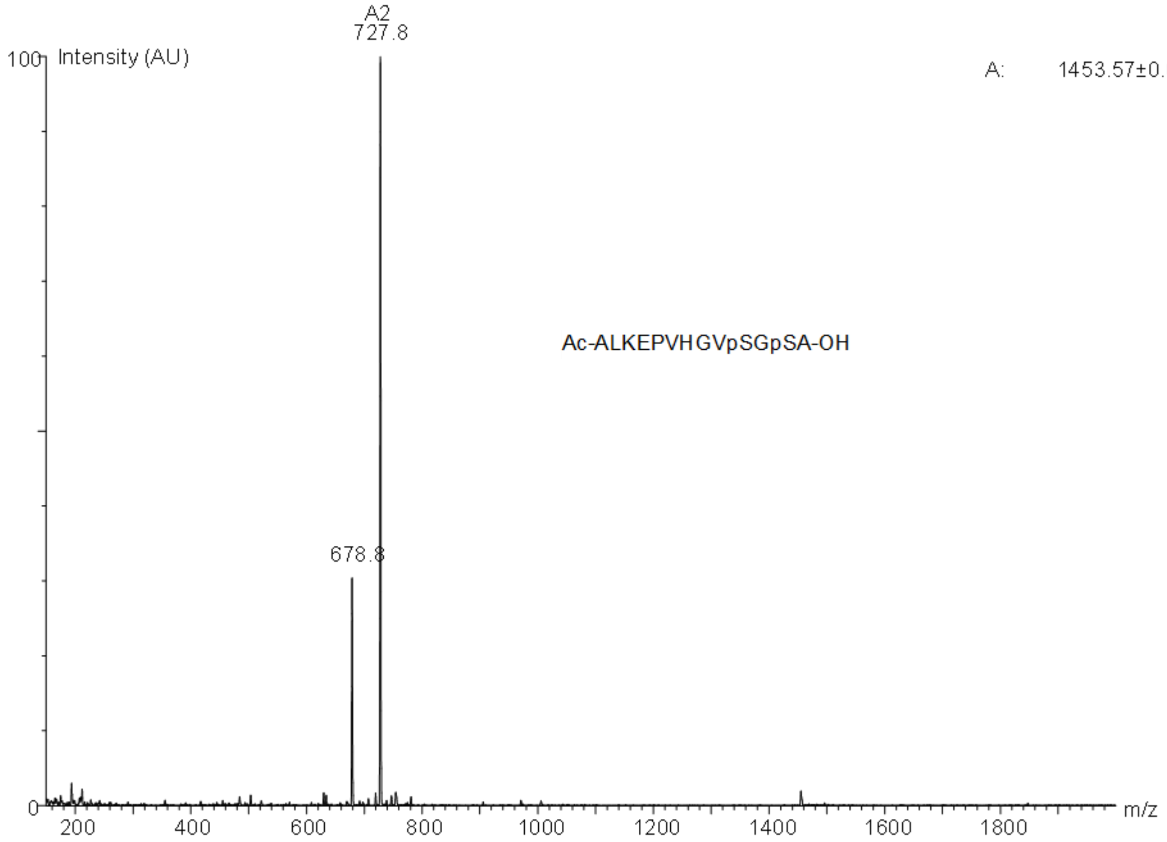

Supplementary Figure 81. MS trace of peak at Rt = 12.35 min from LC-MS analysis of the reaction of peptide thioester Ac-ALKEPVHGVpSGpSA-MPAA **1a** with peptide GALKEPVHGV-NH_2 **2g** after 20 h (5 mM peptide concentration). Peptide thioester hydrolysis byproduct Ac-ALKEPVHGVpSGpSA-OH. MS trace, $[M+2H]^{2+}$ m/z calcd. (av.) 727.70, obs. 727.8.

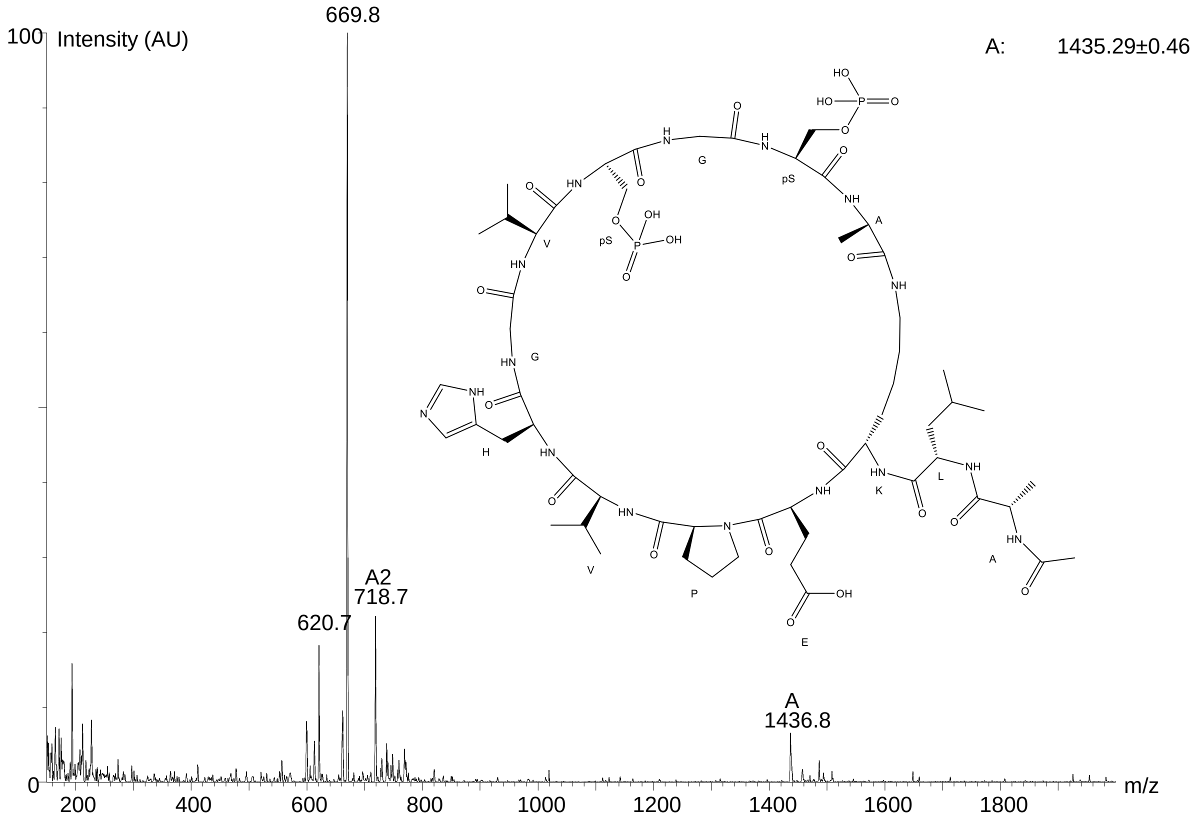

Supplementary Figure 82. MS trace of peak at Rt = 13.37 min from LC-MS analysis of the reaction of peptide thioester Ac-ALKEPVHGVpSGpSA-MPAA **1a** with peptide GALKEPVHGV-NH_2 **2g** after 20 h (5 mM peptide concentration). Peptide thioester cyclized byproduct. MS trace, $[M+H]^+$ m/z calcd. (av.) 1436.40, obs. 1436.8, $[M+2H]^{2+}$ m/z calcd. (av.) 718.69, obs. 718.7.

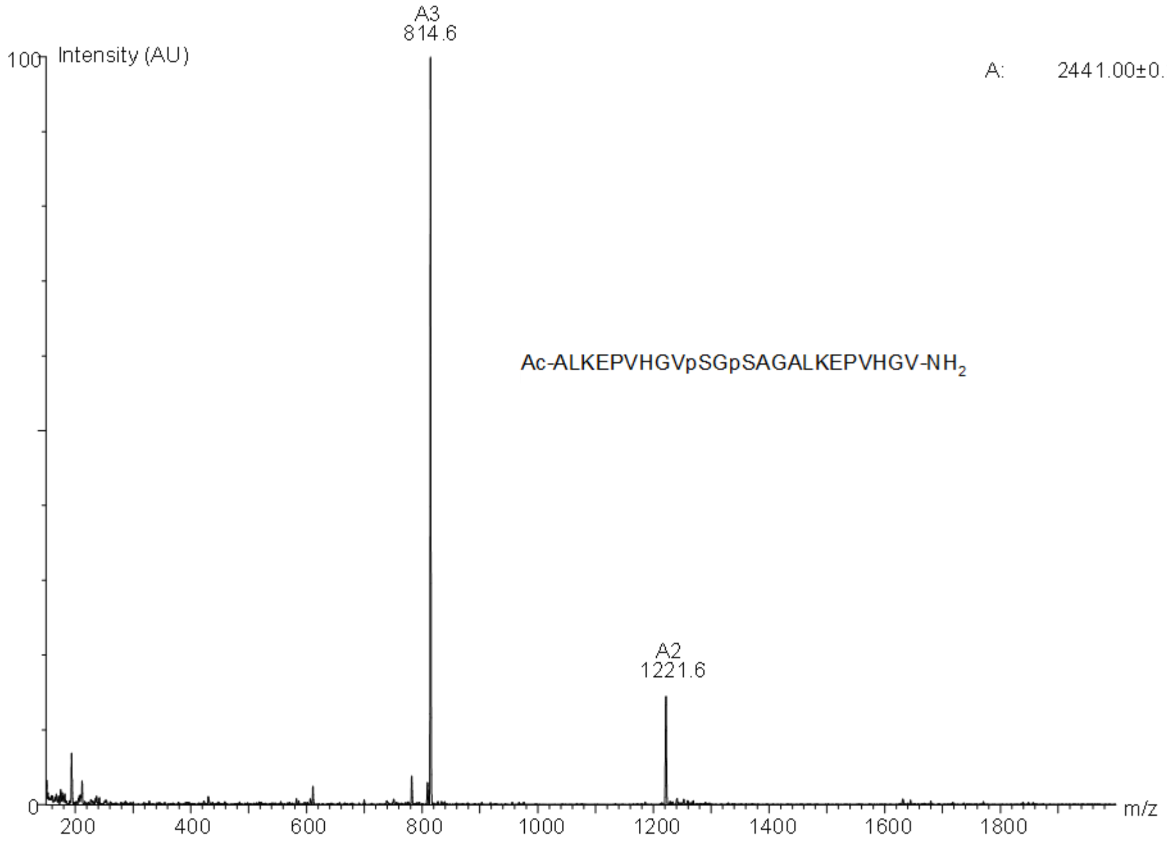

Supplementary Figure 83. MS trace of peak at Rt = 15.09 min from LC-MS analysis of the reaction of Ac-ALKEPVHGVpSGpSA-MPAA **1a** with peptide GALKEPVHGV-NH_2 **2g** after 20 h (5 mM peptide concentration). Ac-ALKEPVHGVpSGpSAGALKEPVHGV-NH_2 **3a,g**. $[M+2H]^{2+}$ m/z calcd. (av.) 1221.78, obs. 1221.6, $[M+3H]^{3+}$ m/z calcd. (av.) 814.85, obs. 814.6.

1d + 2g → 3d,g:

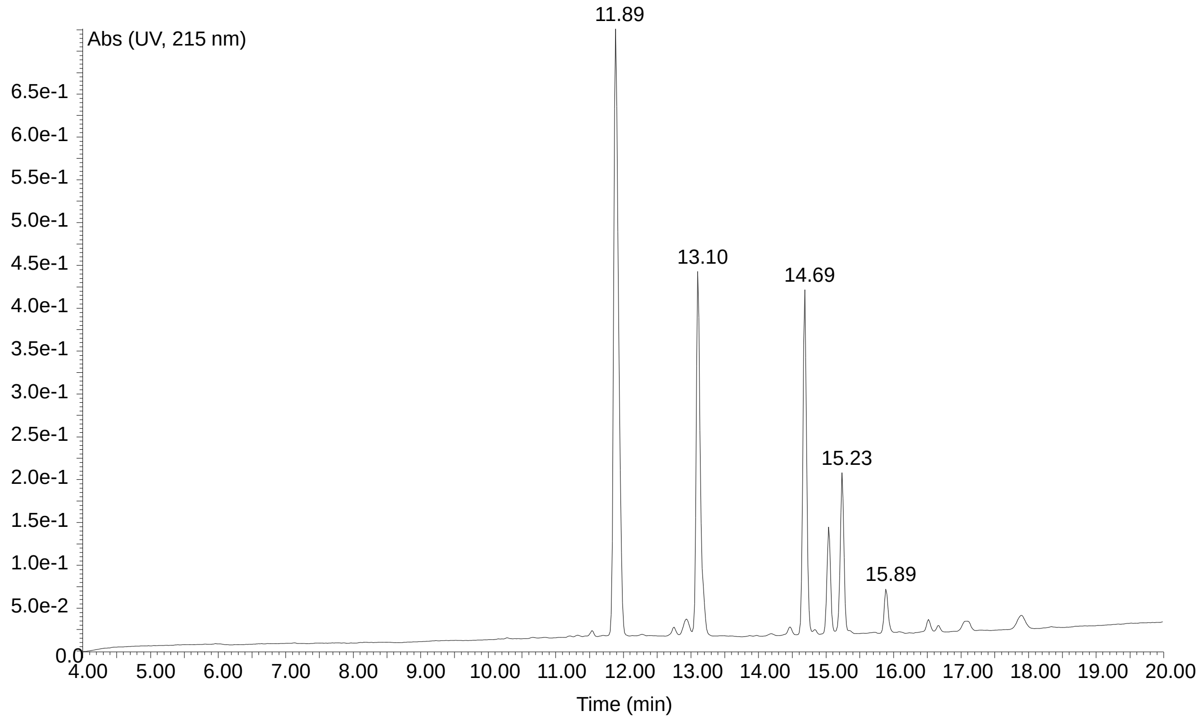

Supplementary Figure 84**.** LC-MS analysis of the reaction of peptide thioester Ac-ALKEPVHGVSGSA-MPAA **1d** with peptide GALKEPVHGV-NH_2 **2g** after 20 h (5 mM peptide concentration). LC trace. Eluent A 0.1% TFA in water, eluent B 0.1% TFA in CH_3CN. C18 X bridge BEH 300 Å (5 µm, 4.6 × 250 mm) column, gradient 0-50% B in 30 min, 1 mL min^{-1}, detection at 215 nm).

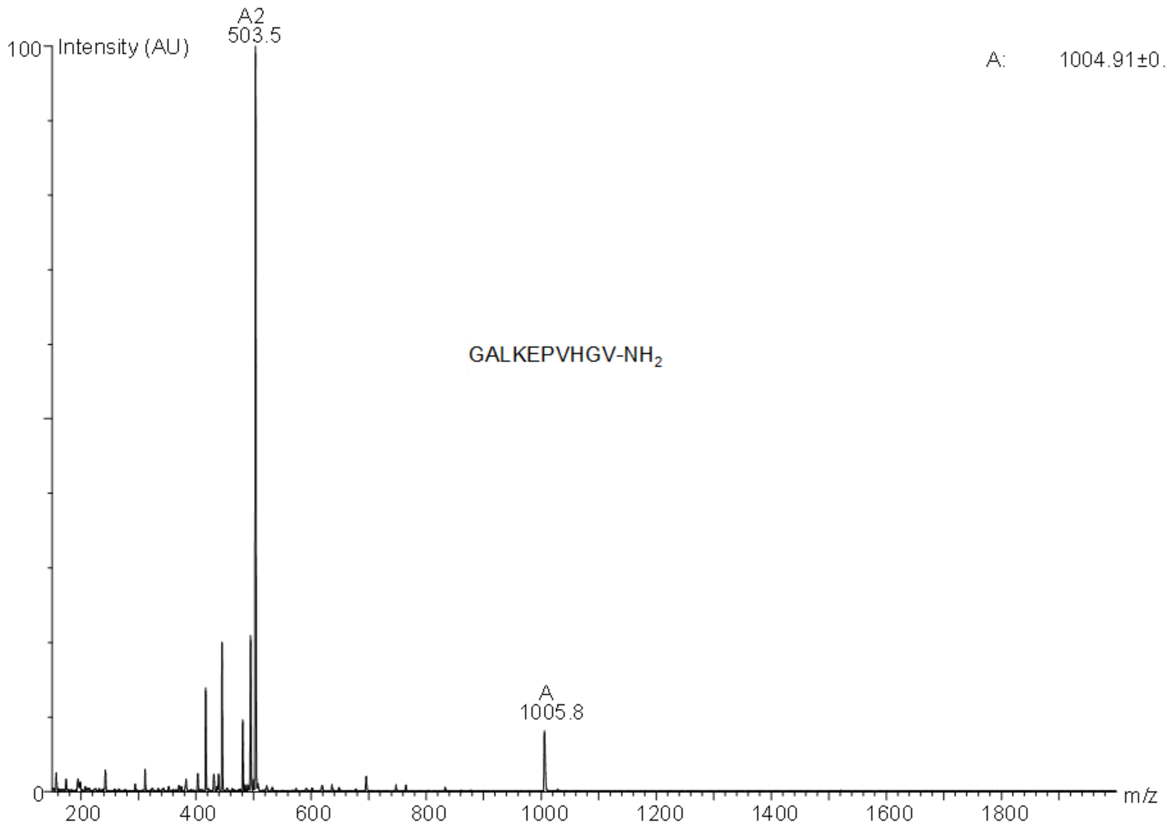

Supplementary Figure 85. MS trace of peak at Rt = 11.89 min from LC-MS analysis of the reaction of Ac-ALKEPVHGVSGSA-MPAA **1d** with peptide GALKEPVHGV-NH_2 **2g** after 20 h (5 mM peptide concentration). GALKEPVHGV-NH_2 **2g**. $[M+H]^+$ m/z calcd. (monoisotopic) 1005.58, obs. 1005.8, $[M+2H]^{2+}$ m/z calcd. (av.) 503.59, obs. 503.5.

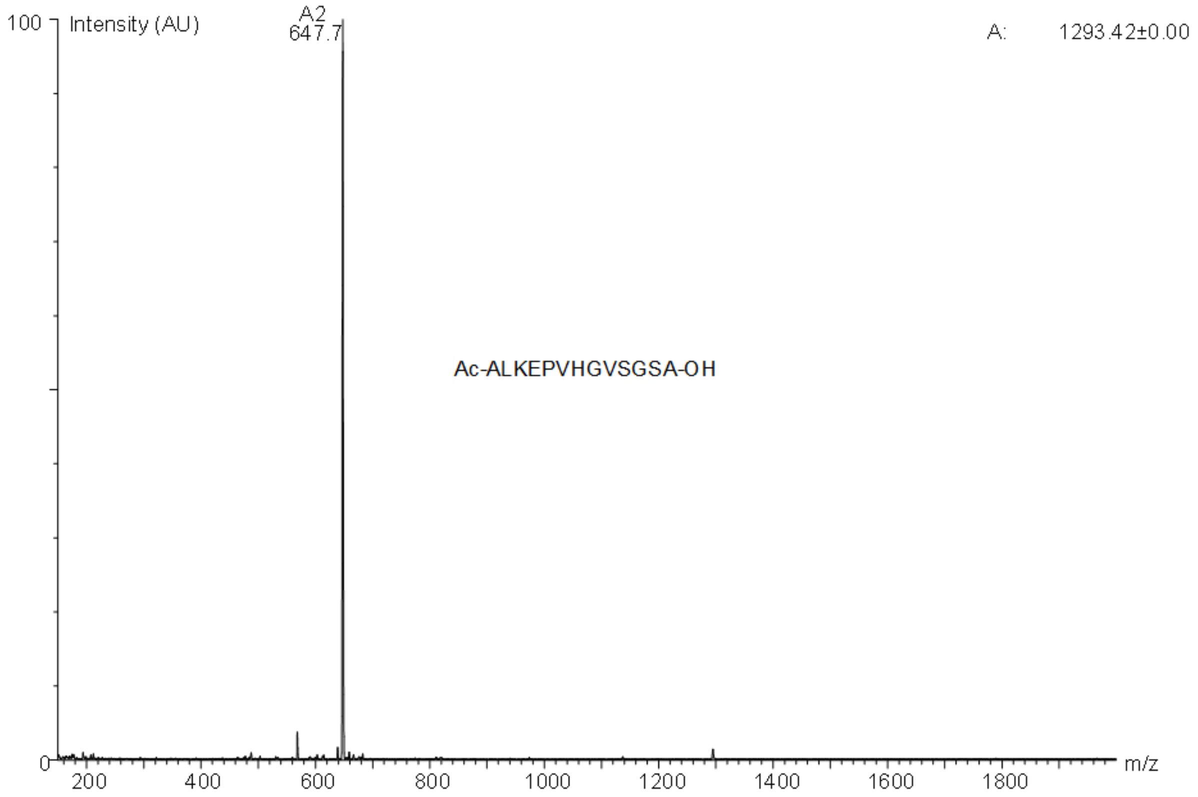

Supplementary Figure 86. MS trace of peak at Rt = 13.10 min from LC-MS analysis of the reaction of Ac-ALKEPVHGVSGSA-MPAA **1d** with peptide GALKEPVHGV-NH_2 **2g** after 20 h (5 mM peptide concentration). Peptide thioester hydrolysis byproduct Ac-ALKEPVHGVSGSA-OH. $[M+2H]^{2+}$ m/z calcd. (av.) 647.72, obs. 647.7.

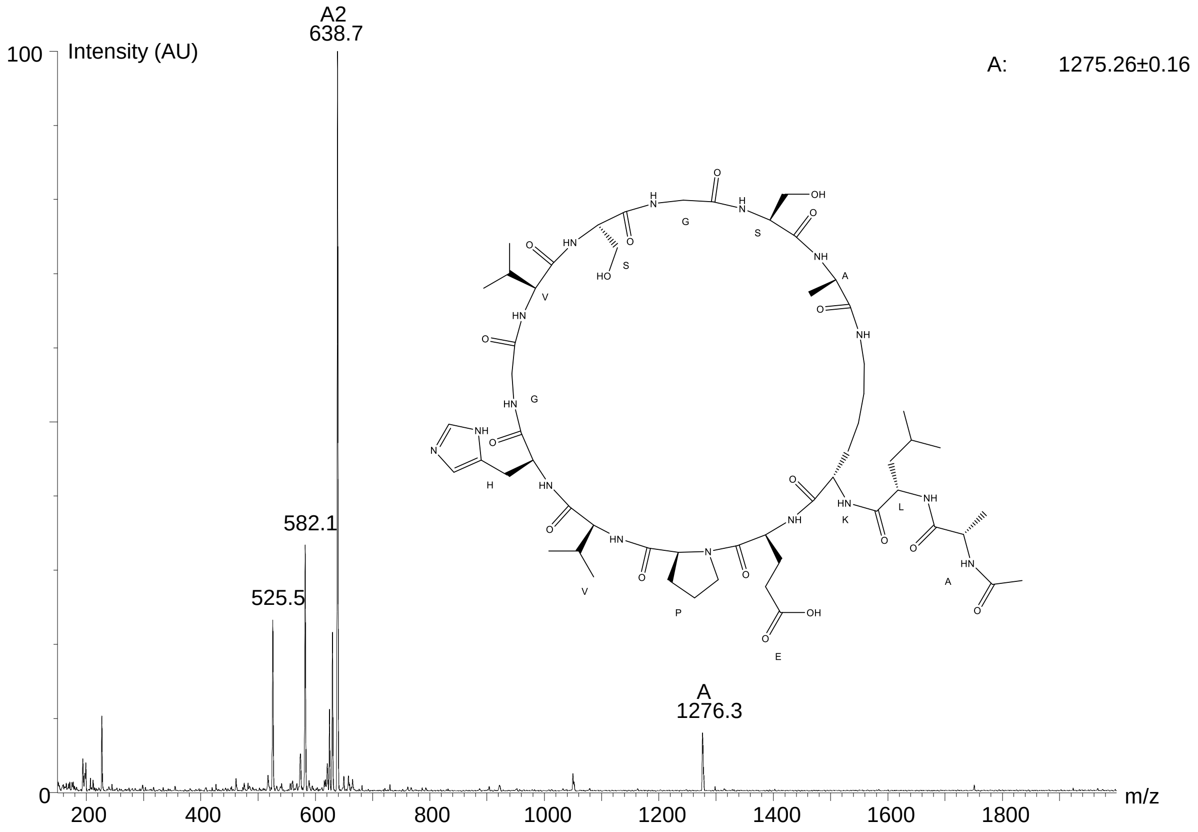

Supplementary Figure 87. MS trace of peak at Rt = 14.69 min from LC-MS analysis of the reaction of Ac-ALKEPVHGVSGSA-MPAA **1d** with peptide GALKEPVHGV-NH_2 **2g** after 20 h (5 mM peptide concentration). Peptide thioester cyclized byproduct. $[M+H]^+$ m/z calcd. (av.) 1276.43, obs. 1276.3, $[M+2H]^{2+}$ m/z calcd. (av.) 638.71, obs. 638.7.

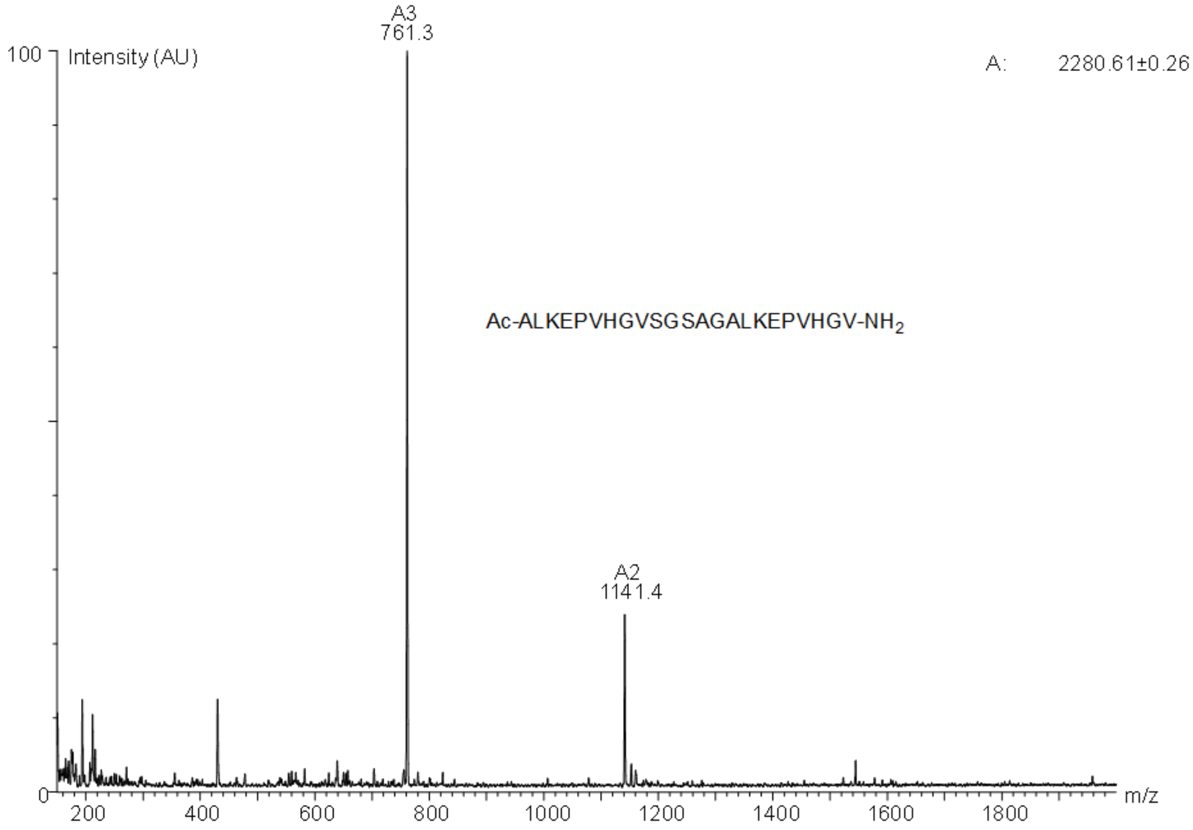

Supplementary Figure 88. MS trace of peak at Rt = 15.05 min from LC-MS analysis of the reaction of Ac-ALKEPVHGVSGSA-MPAA **1d** with peptide GALKEPVHGV-NH_2 **2g** after 20 h (5 mM peptide concentration). Ac-ALKEPVHGVSGSAGALKEPVHGV-NH_2 **3d,g**: $[M+2H]^{2+}$ m/z calcd. (av.) 1141.31, obs. 1141.4, $[M+3H]^{3+}$ m/z calcd. (av.) 761.21, obs. 761.3.

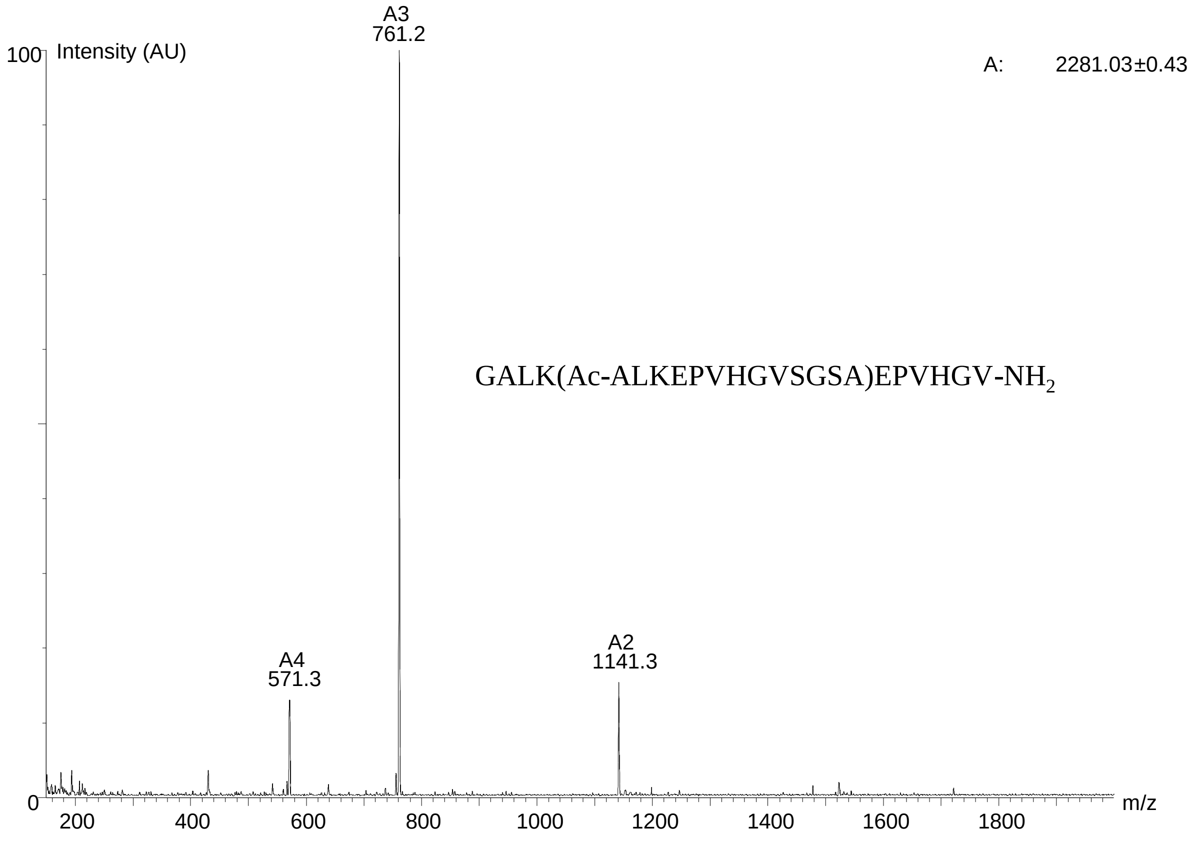

Supplementary Figure 89. MS trace of peak at Rt = 15.23 min from LC-MS analysis of the reaction of Ac-ALKEPVHGVSGSA-MPAA **1d** with peptide GALKEPVHGV-NH_2 **2g** after 20 h (5 mM peptide concentration). Branched byproduct GALK(Ac-ALKEPVHGVSGSA)EPVHGV-NH_2). $[M+2H]^{2+}$ m/z calcd. (av.) 1141.31, obs. 1141.3, $[M+3H]^{3+}$ m/z calcd. (av.) 761.21, obs. 761.2, $[M+4H]^{4+}$ m/z calcd. (av.) 571.15, obs. 571.3.

Influence of the number of Arg residues in the cationic module (Fig. 3c)

*Reaction of peptide thioester **1a** (two pSer residues) with peptides **2a-g** (Fig. 3c)*

Ac-ALKEPVHGVpSGpSA-MPAA peptide **1a** (~0.34 µmol, 5 mM final concentration) was dissolved in 50 mM sodium bicarbonate/CO_2 buffer (474 µL total, 67.6 µL for each glycyl peptide) and added to a series of glycyl peptides varying by the number of Arg present in the positively charged module (glycyl peptides **2a-g**, powders placed in a plastic tube, 1 equiv, 5 mM final concentration). Once dissolved, the reaction mixtures (pH=7.15-7.43) were placed in a CO_2 incubator for cell biology (5% partial CO_2 pressure, 37 °C, water saturated atmosphere) with a needle through the cap to enable CO_2 to diffuse into the plastic tube and the reaction mixture.

After 23 h, the reaction mixtures (1 µL) were quenched with aqueous AcOH (10 % AcOH in water, 100 µL) and analyzed by LC-MS.

1a + 2a → 3a,a:

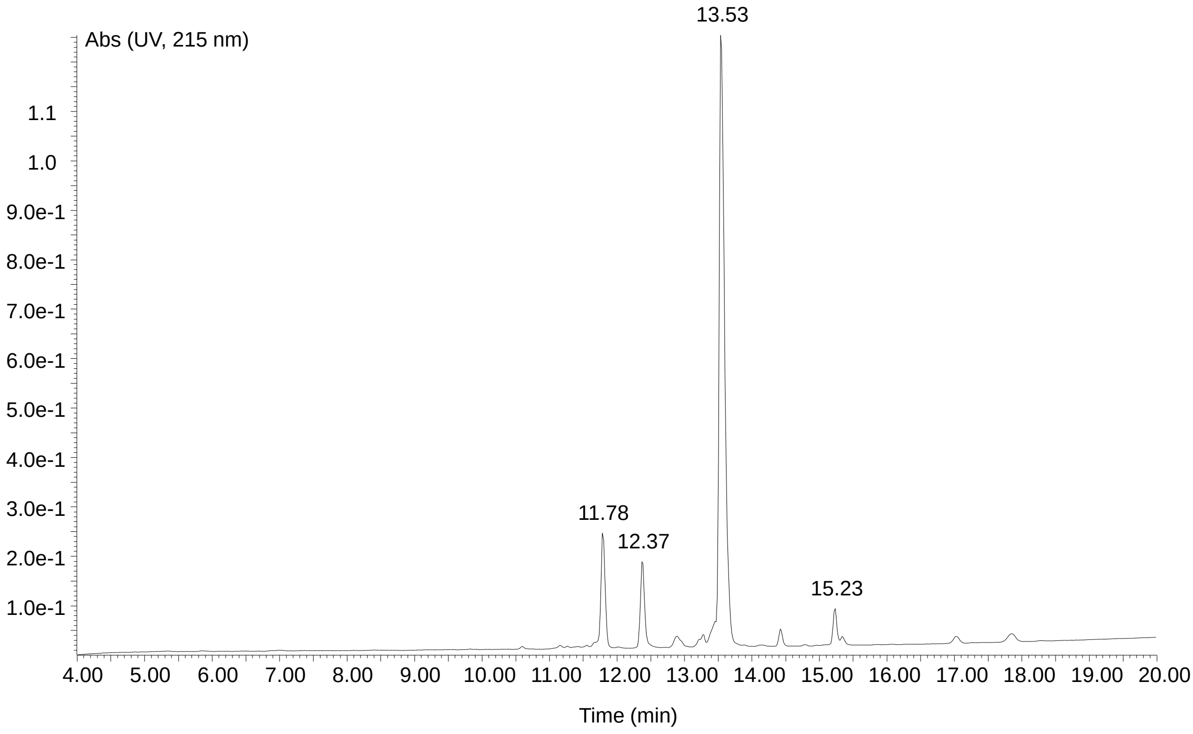

Supplementary Figure 90. LC-MS analysis of the reaction of peptide thioester Ac-ALKEPVHGVpSGpSA-MPAA **1a** with GRRRRRRALKEPVHGV-NH_2 **2a** after 23 h. LC trace. Eluent A 0.1% TFA in water, eluent B 0.1% TFA in CH_3CN. C18 X bridge BEH 300 Å (5 µm, 4.6 × 250 mm) column, gradient 0-50% B in 30 min, 1 mL min^{-1}, detection at 215 nm).

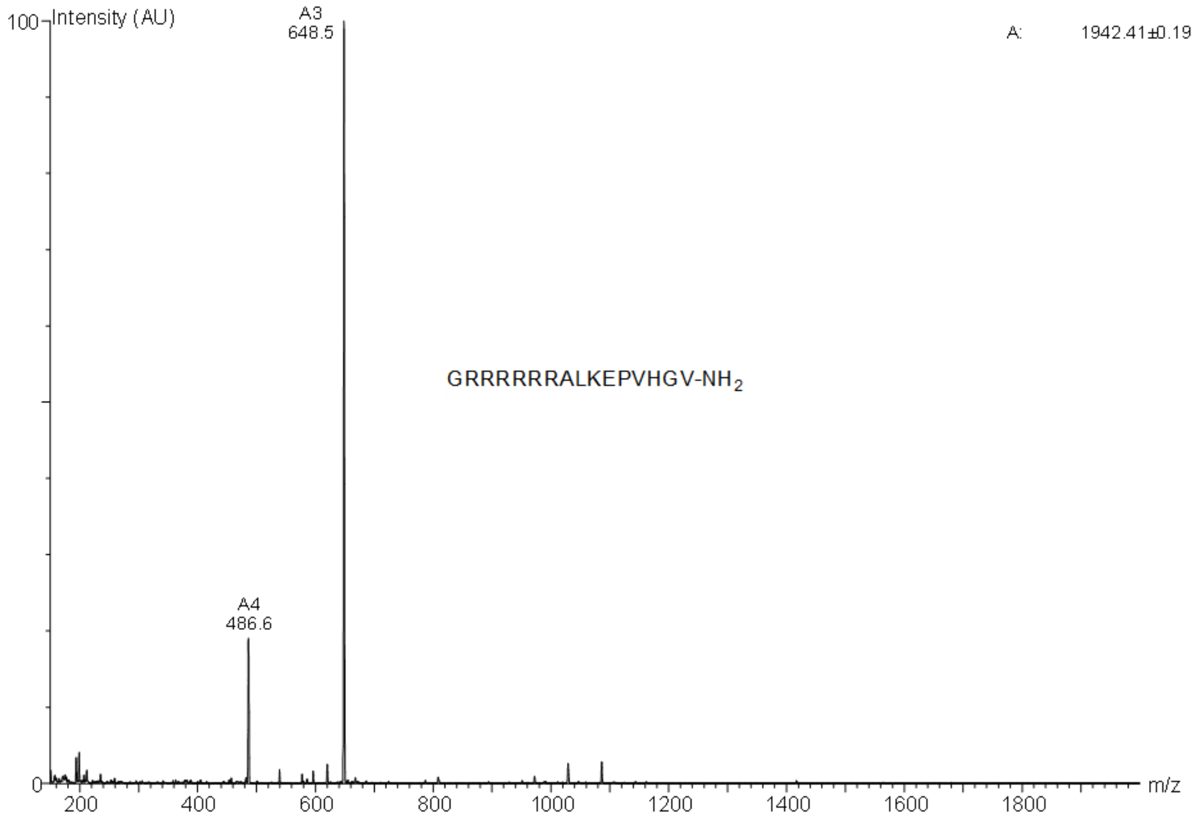

Supplementary Figure 91. MS trace of peak at Rt = 11.78 min from LC-MS analysis of the reaction of Ac-ALKEPVHGVpSGpSA-MPAA **1a** with GRRRRRRALKEPVHGV-NH_2 **2a** after 23 h. GRRRRRRALKEPVHGV-NH_2 **2a**. $[M+3H]^{3+}$ m/z calcd. (av.) 648.44, obs. 648.5, $[M+4H]^{4+}$ m/z calcd. (av.) 486.58, obs. 486.6.

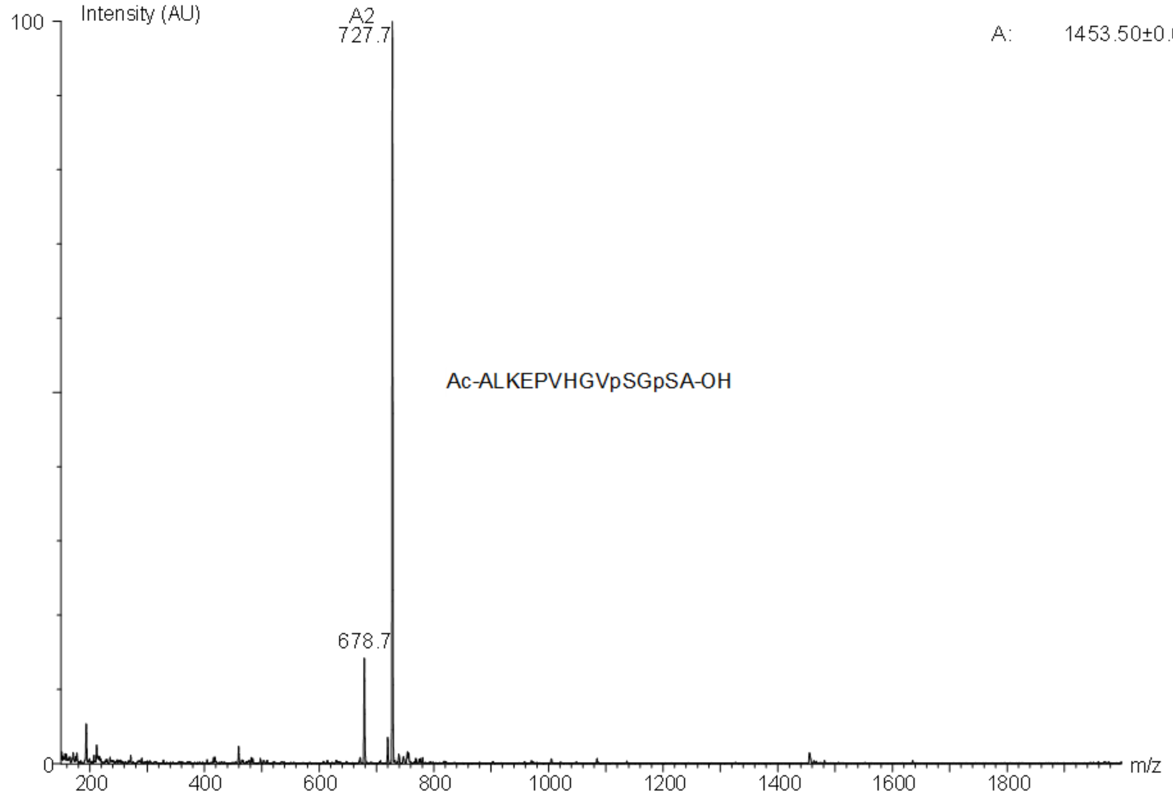

Supplementary Figure 92. MS trace of peak at Rt = 12.37 min from LC-MS analysis of the reaction of Ac-ALKEPVHGVpSGpSA-MPAA **1a** with GRRRRRRALKEPVHGV-NH_2 **2a** after 23 h. Thioester hydrolysis byproduct Ac-ALKEPVHGVpSGpSA-OH. $[M+2H]^{2+}$ m/z calcd. (av.) 727.70, obs. 727.7.

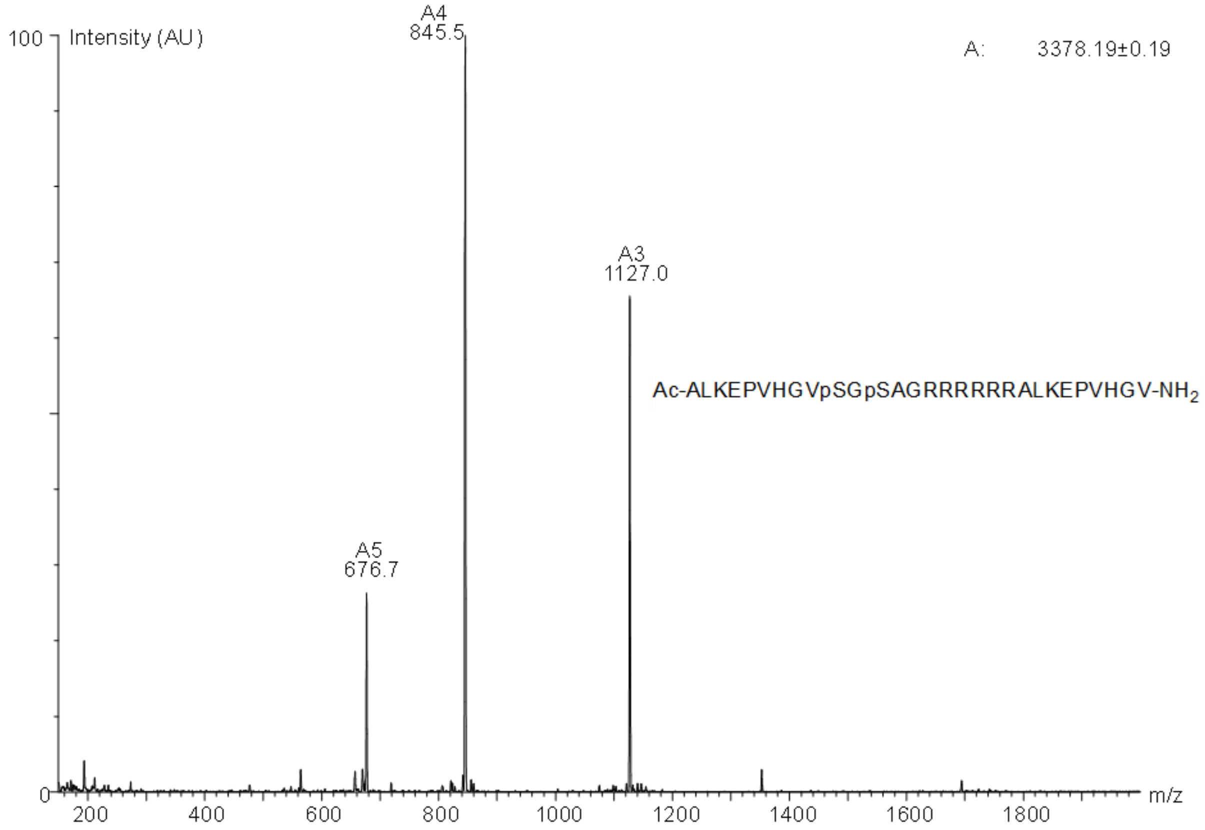

Supplementary Figure 93. MS trace of peak at Rt = 13.53 min from LC-MS analysis of the reaction of Ac-ALKEPVHGVpSGpSA-MPAA **1a** with GRRRRRRALKEPVHGV-NH_2 **2a** after 23 h. Target ligation product Ac-ALKEPVHGVpSGpSAGRRRRRRALKEPVHGV-NH_2 **3a,a**. $[M+3H]^{3+}$ m/z calcd. (av.) 1126.90, obs. 1127.0, $[M+4H]^{4+}$ m/z calcd. (av.) 845.43, obs. 845.5, $[M+5H]^{5+}$ m/z calcd. (av.) 676.54, obs. 676.7.

1a + 2b → 3a,b:

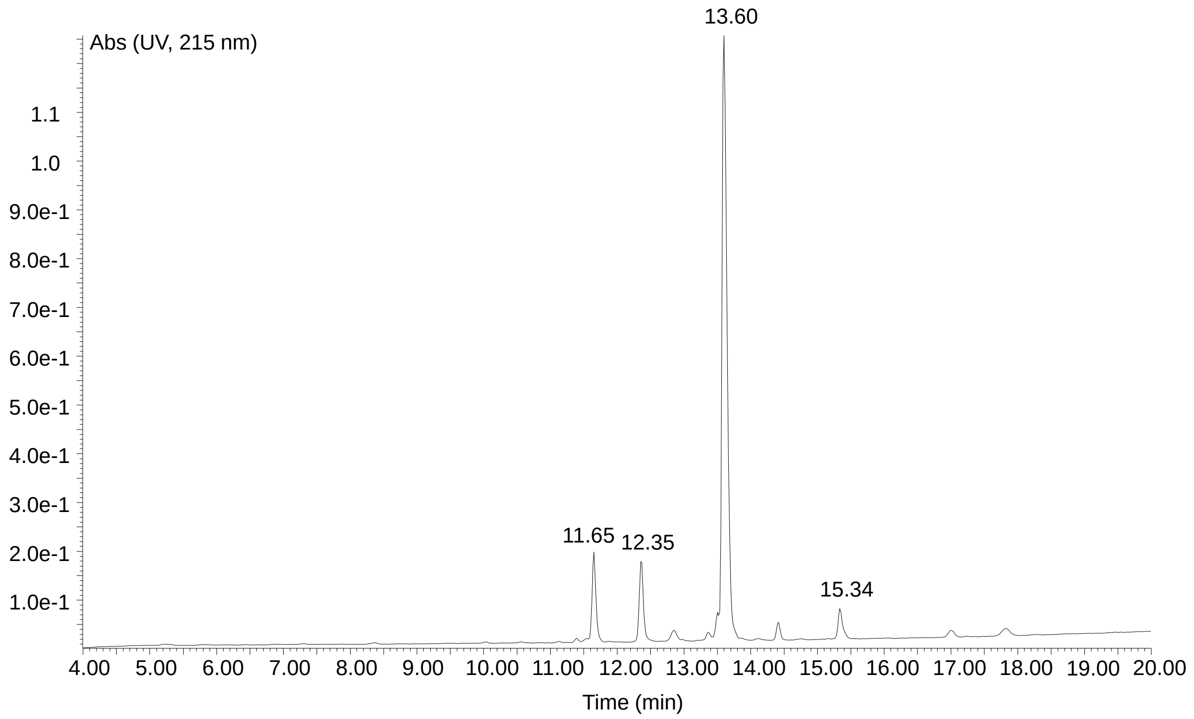

Supplementary Figure 94**.** LC-MS analysis of the reaction of peptide thioester Ac-ALKEPVHGVpSGpSA-MPAA **1a** with GRRRRRALKEPVHGV-NH_2 **2b** after 23 h. LC trace. Eluent A 0.1% TFA in water, eluent B 0.1% TFA in CH_3CN. C18 X bridge BEH 300 Å (5 µm, 4.6 × 250 mm) column, gradient 0-50% B in 30 min, 1 mL min^{-1}, detection at 215 nm).

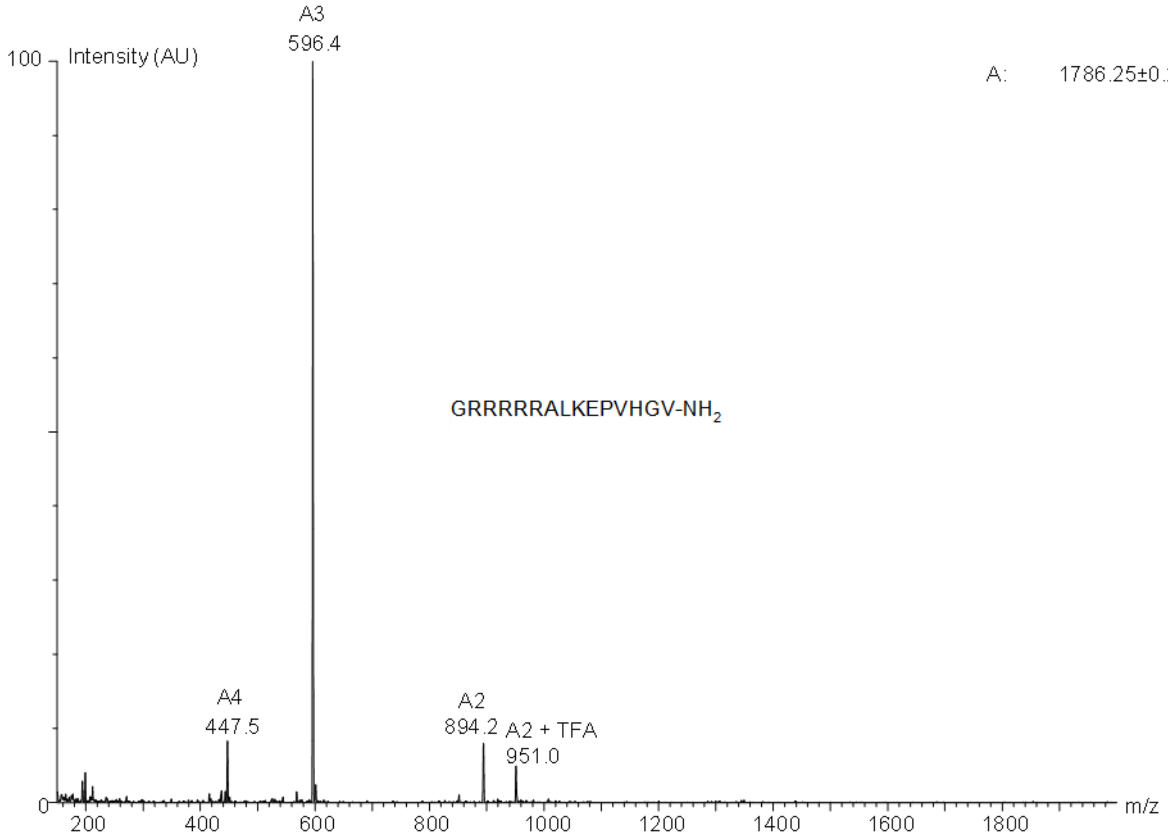

Supplementary Figure 95. MS trace of peak at Rt = 11.65 min from LC-MS analysis of the reaction of Ac-ALKEPVHGVpSGpSA-MPAA **1a** with GRRRRRALKEPVHGV-NH_2 **2b** after 23 h. GRRRRRALKEPVHGV-NH_2 **2b**. $[M+2H]^{2+}$ m/z calcd. (av.) 894.07, obs. 894.2, $[M+3H]^{3+}$ m/z calcd. (av.) 596.38, obs. 596.4, $[M+4H]^{4+}$ m/z calcd. (av.) 447.53, obs. 447.5.

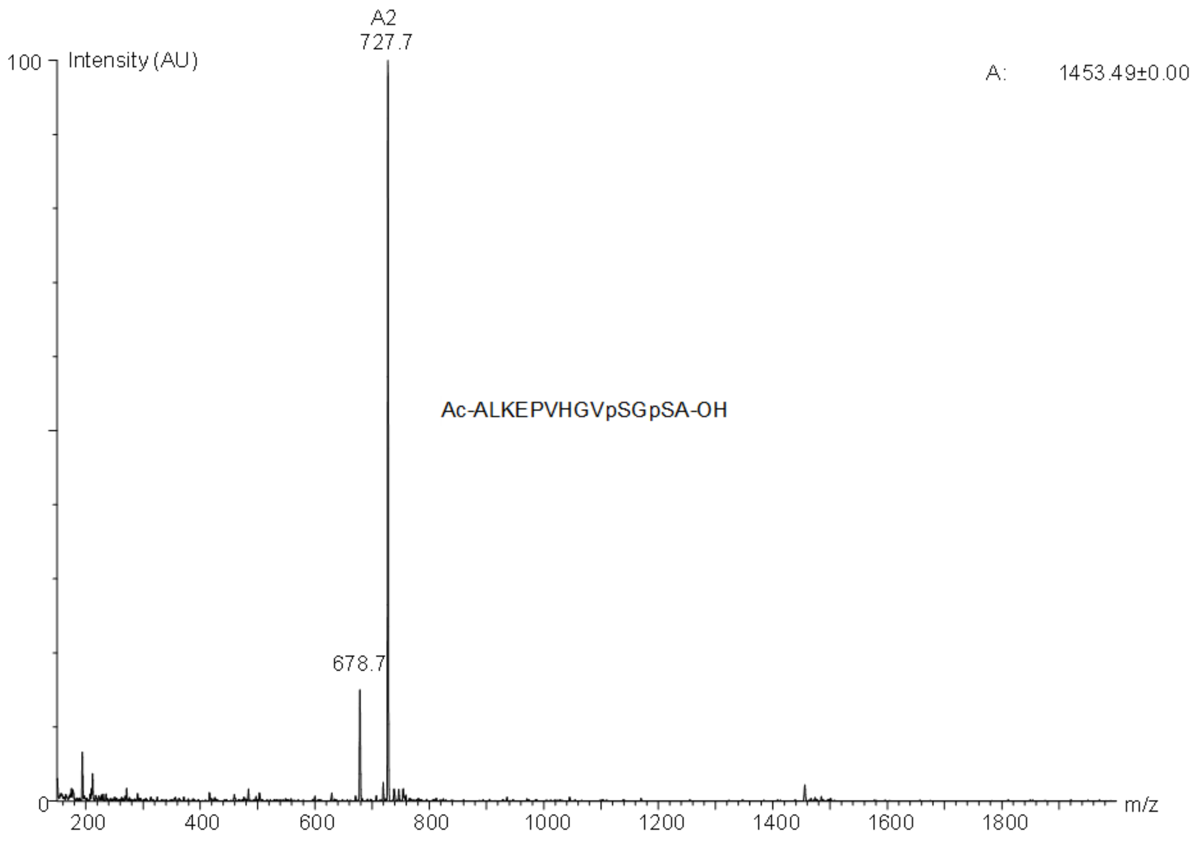

Supplementary Figure 96. MS trace of peak at Rt = 12.35 min from LC-MS analysis of the reaction of Ac-ALKEPVHGVpSGpSA-MPAA **1a** with GRRRRRALKEPVHGV-NH_2 **2b** after 23 h. Peptide thioester byproduct Ac-ALKEPVHGVpSGpSA-OH. $[M+2H]^{2+}$ m/z calcd. (av.) 727.70, obs. 727.7.

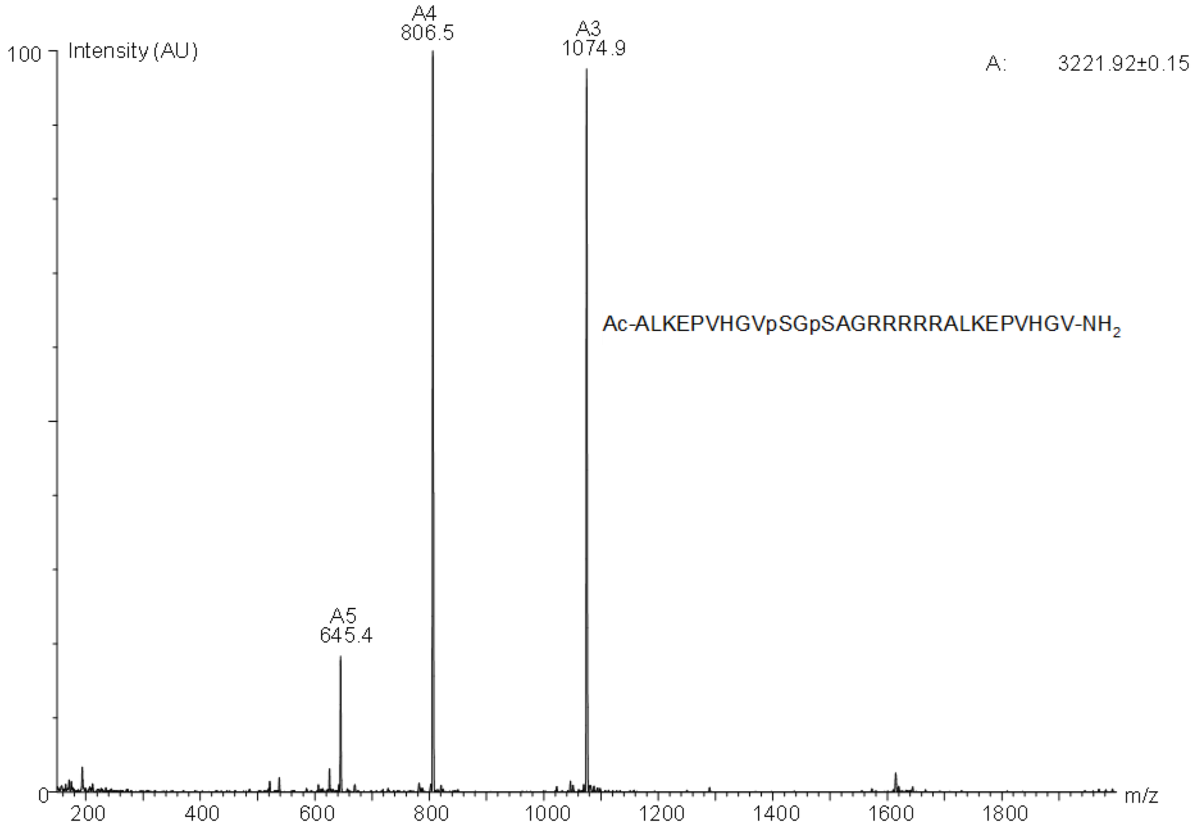

Supplementary Figure 97. MS trace of peak at Rt = 13.60 min from LC-MS analysis of the reaction of peptide thioester Ac-ALKEPVHGVpSGpSA-MPAA **1a** with GRRRRRALKEPVHGV-NH_2 **2b** after 23 h. Target ligation product Ac-ALKEPVHGVpSGpSAGRRRRRALKEPVHGV-NH_2 **3a,b**. $[M+3H]^{3+}$ m/z calcd. (av.) 1074.84, obs. 1074.9, $[M+4H]^{4+}$ m/z calcd. (av.) 806.38, obs. 806.5, $[M+5H]^{5+}$ m/z calcd. (av.) 645.30, obs. 645.4.

1a + 2c → 3a,c:

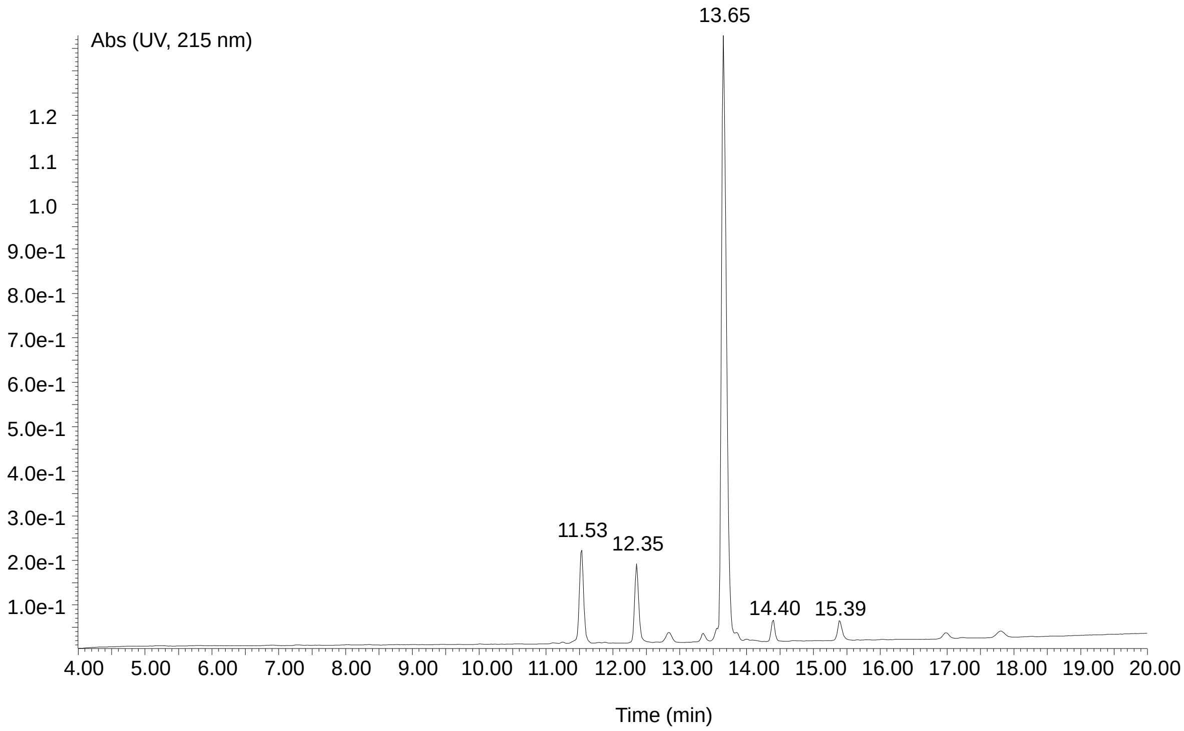

Supplementary Figure 98. LC-MS analysis of the reaction of peptide thioester Ac-ALKEPVHGVpSGpSA-MPAA **1a** with GRRRRALKEPVHGV-NH_2 **2c** after 23 h. LC trace. Eluent A 0.1% TFA in water, eluent B 0.1% TFA in CH_3CN. C18 X bridge BEH 300 Å (5 µm, 4.6 × 250 mm) column, gradient 0-50% B in 30 min, 1 mL min^{-1}, detection at 215 nm).

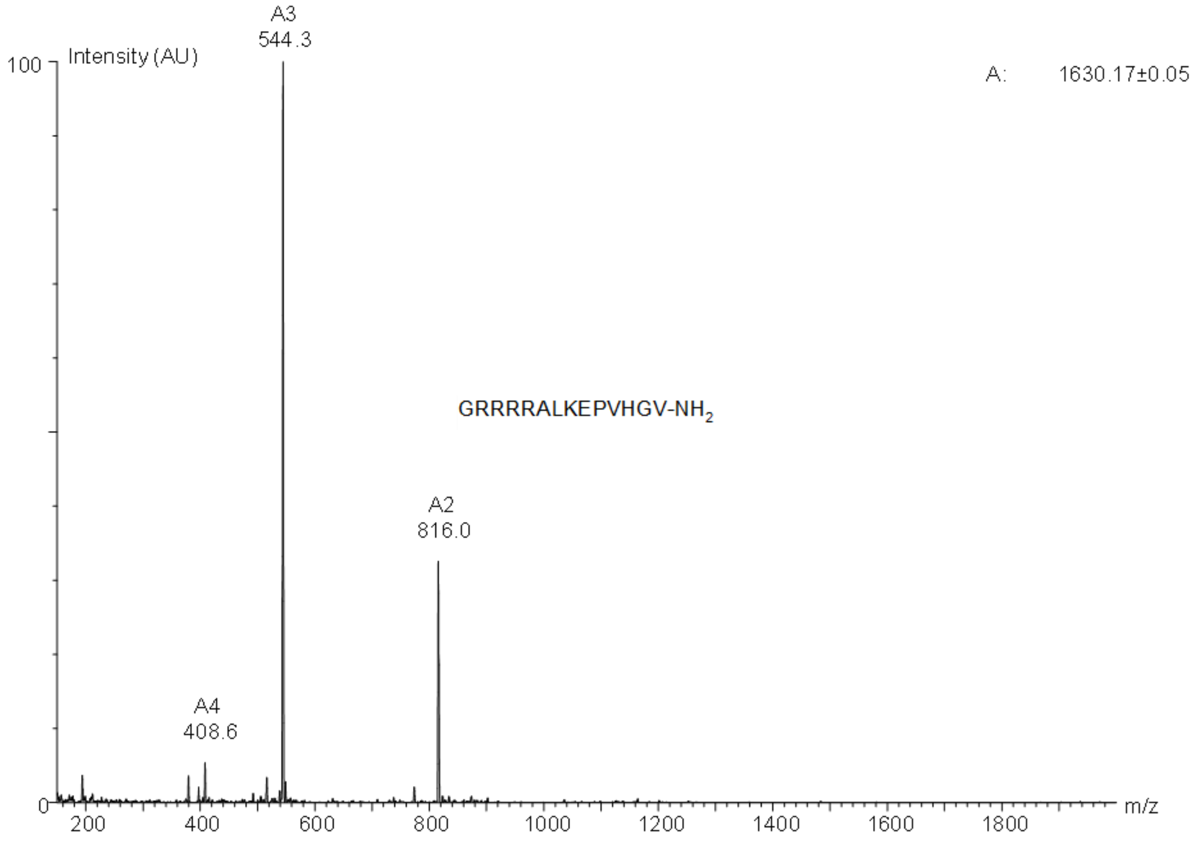

Supplementary Figure 99. MS trace of peak at Rt = 11.53 min from LC-MS analysis of the reaction of peptide thioester Ac-ALKEPVHGVpSGpSA-MPAA **1a** with GRRRRALKEPVHGV-NH_2 **2c** after 23 h. GRRRRALKEPVHGV-NH_2 **2c**. $[M+2H]^{2+}$ m/z calcd. (av.) 815.97, obs. 816.0, $[M+3H]^{3+}$ m/z calcd. (av.) 544.31, obs. 544.3, $[M+4H]^{4+}$ m/z calcd. (av.) 408.49, obs. 408.6.

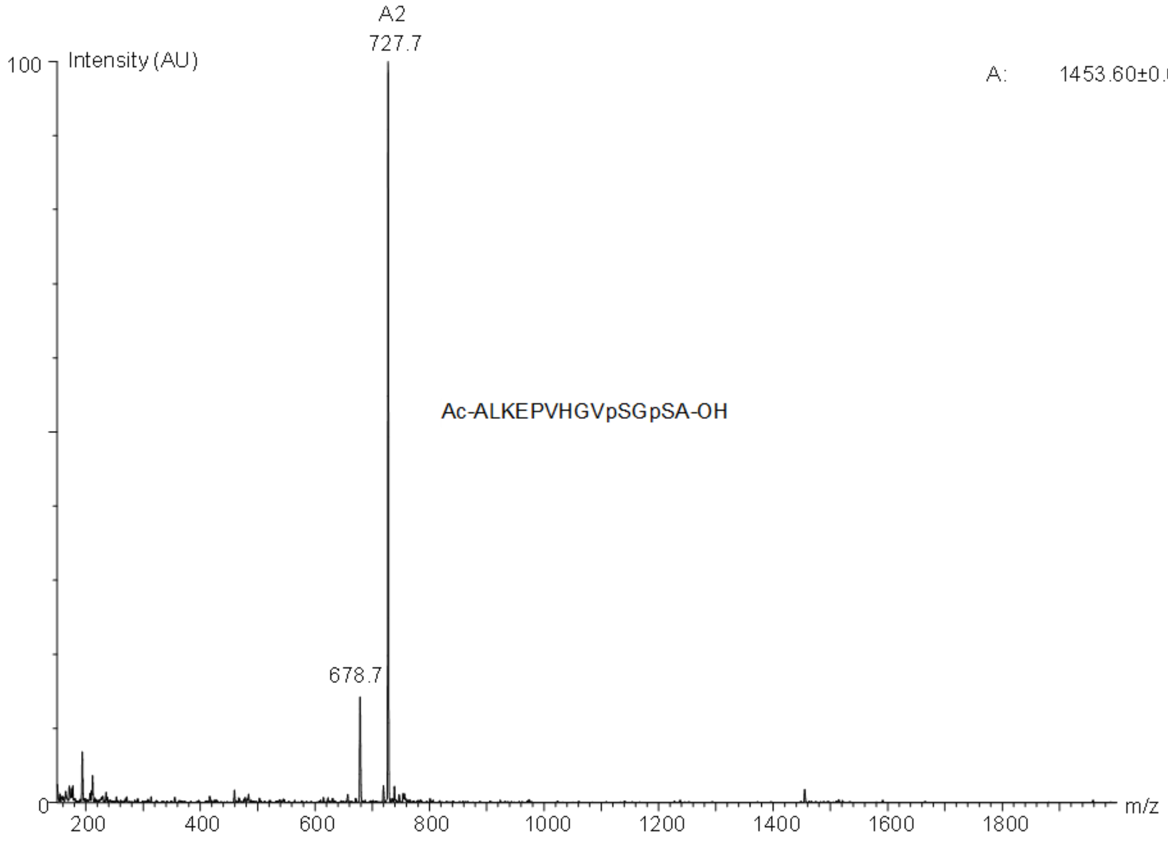

Supplementary Figure 100. MS trace of peak at Rt = 12.35 min from LC-MS analysis of the reaction of peptide thioester Ac-ALKEPVHGVpSGpSA-MPAA **1a** with GRRRRALKEPVHGV-NH_2 **2c** after 23 h. Peptide thioester hydrolysis byproduct Ac-ALKEPVHGVpSGpSA-OH. $[M+2H]^{2+}$ m/z calcd. (av.) 727.70, obs. 727.7, $[M-H_3PO_4+2H]^{2+}$ m/z calcd. (av.) 678.7, obs. 678.7.

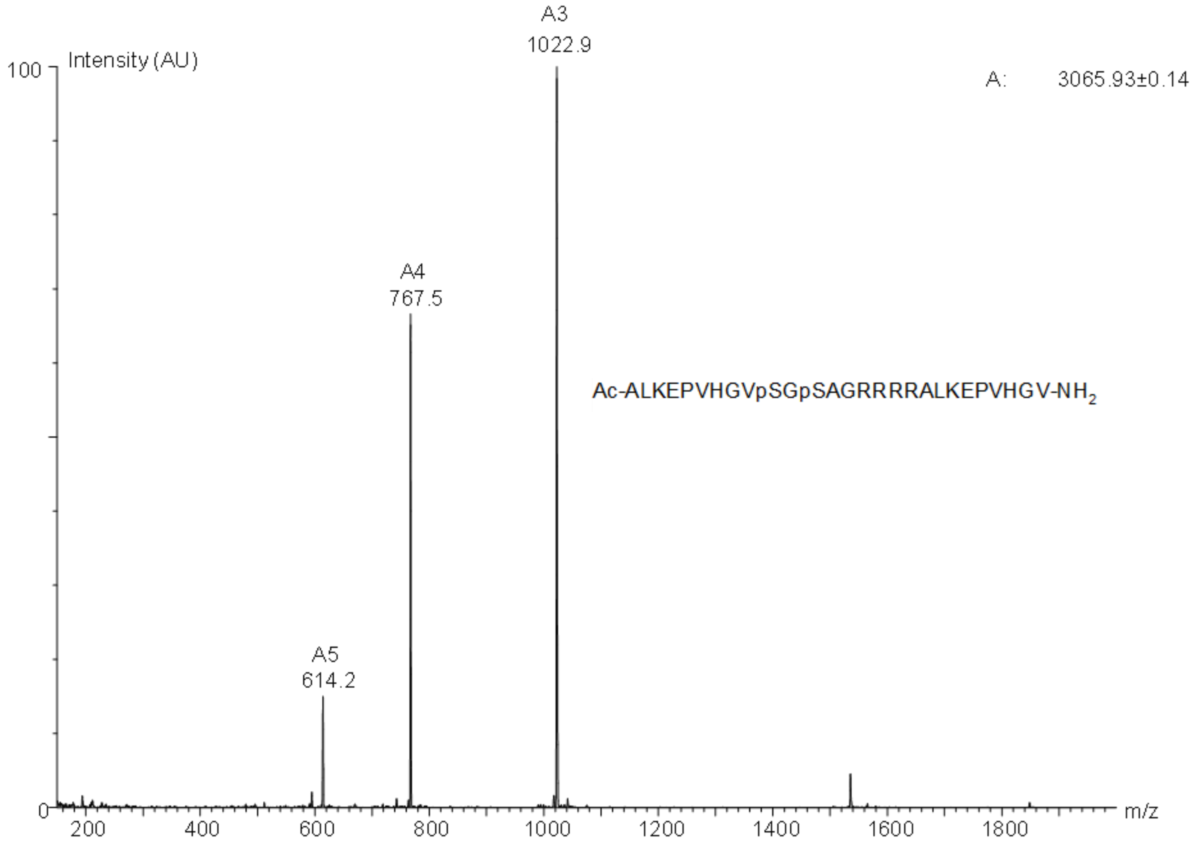

Supplementary Figure 101. MS trace of peak at Rt = 13.65 min from LC-MS analysis of the reaction of peptide thioester Ac-ALKEPVHGVpSGpSA-MPAA **1a** with GRRRRALKEPVHGV-NH_2 **2c** after 23 h. Target ligation product Ac-ALKEPVHGVpSGpSAGRRRRALKEPVHGV-NH_2 **3a,c**. $[M+3H]^{3+}$ m/z calcd. (av.) 1022.78, obs. 1022.9, $[M+4H]^{4+}$ m/z calcd. (av.) 767.33, obs. 767.5, $[M+5H]^{5+}$ m/z calcd. (av.) 614.07, obs. 614.2.

1a + 2d → 3a,d:

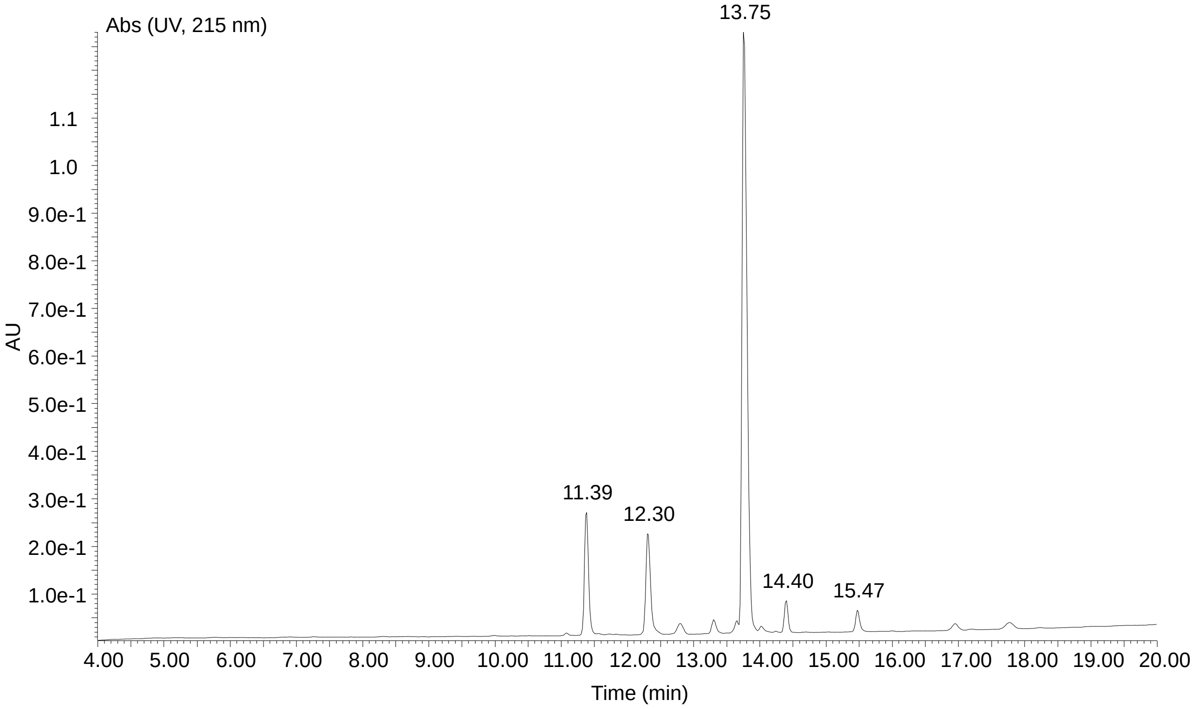

Supplementary Figure 102**.** LC-MS analysis of the reaction of peptide thioester Ac-ALKEPVHGVpSGpSA-MPAA **1a** with GRRRALKEPVHGV-NH_2 **2d** after 23 h. LC trace. Eluent A 0.1% TFA in water, eluent B 0.1% TFA in CH_3CN. C18 X bridge BEH 300 Å (5 µm, 4.6 × 250 mm) column, gradient 0-50% B in 30 min, 1 mL min^{-1}, detection at 215 nm).

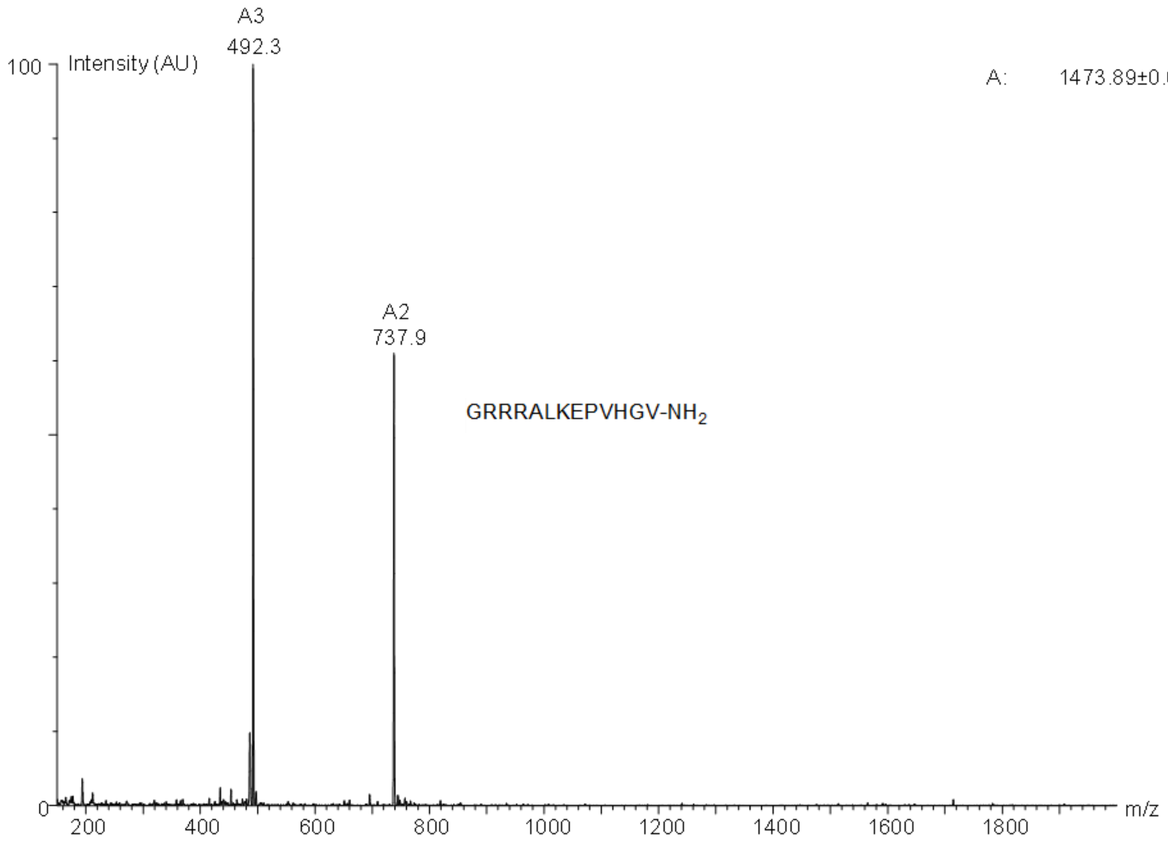

Supplementary Figure 103. MS trace of peak at Rt = 11.39 min from LC-MS analysis of the reaction of peptide thioester Ac-ALKEPVHGVpSGpSA-MPAA **1a** with GRRRALKEPVHGV-NH_2 **2d** after 23 h. GRRRALKEPVHGV-NH_2 **2d**. $[M+2H]^{2+}$ m/z calcd. (av.) 737.88, obs. 737.9, $[M+3H]^{3+}$ m/z calcd. (av.) 492.25, obs. 492.3.

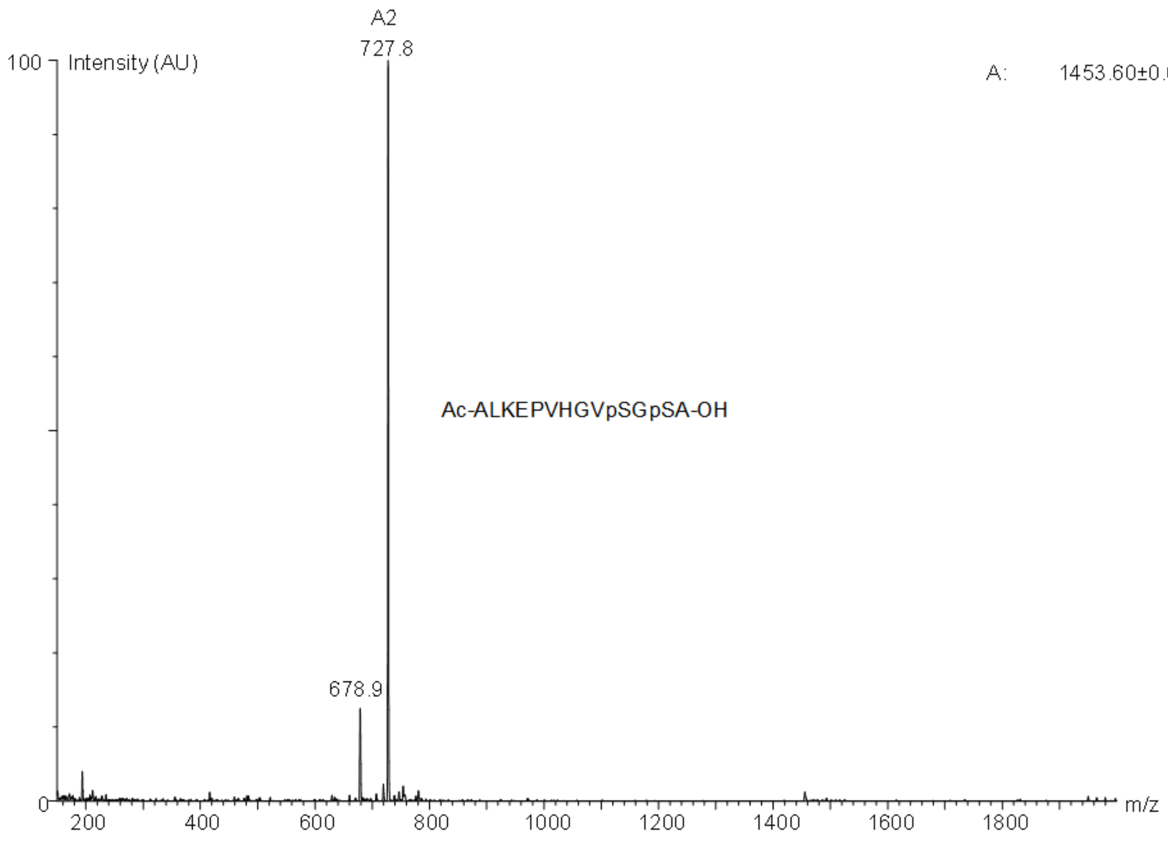

Supplementary Figure 104. MS trace of peak at Rt = 12.30 min from LC-MS analysis of the reaction of peptide thioester Ac-ALKEPVHGVpSGpSA-MPAA **1a** with GRRRALKEPVHGV-NH_2 **2d** after 23 h. Peptide thioester hydrolysis byproduct Ac-ALKEPVHGVpSGpSA-OH. $[M+2H]^{2+}$ m/z calcd. (av.) 727.70, obs. 727.8.

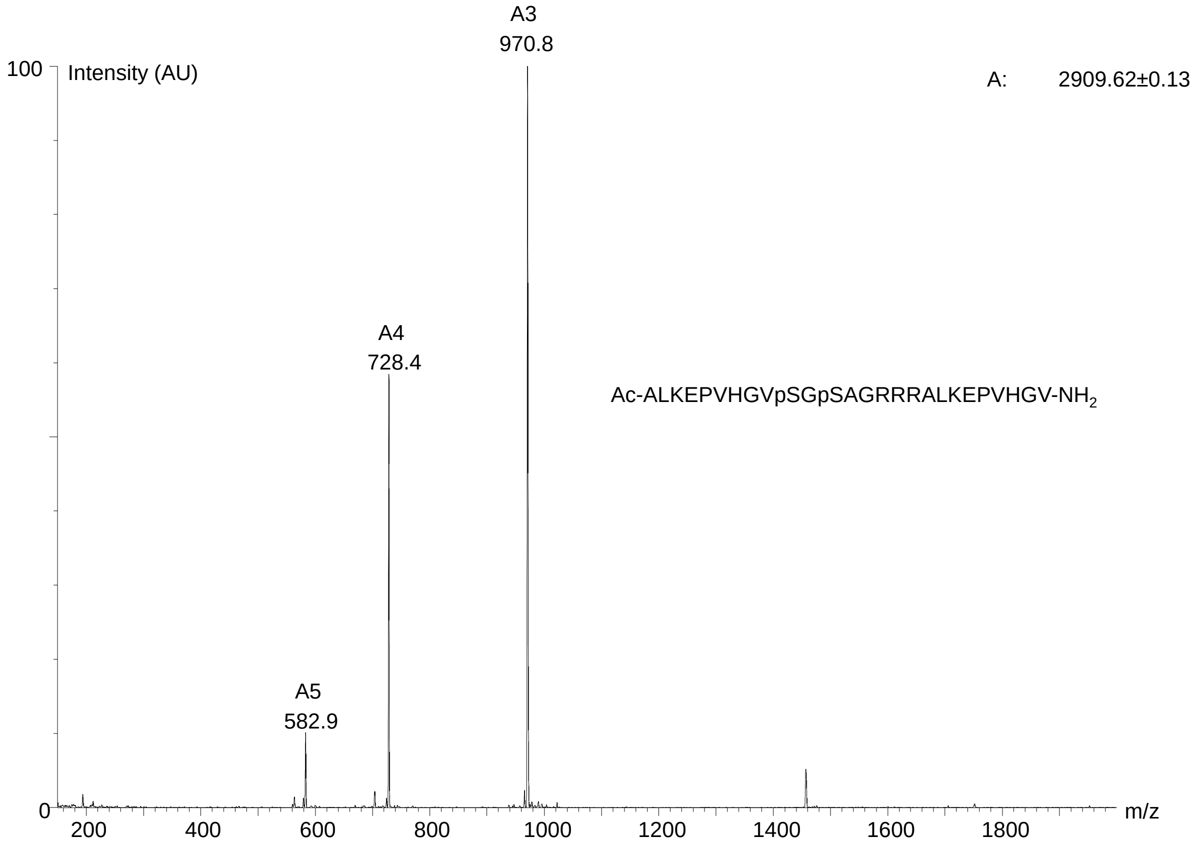

Supplementary Figure 105. MS trace of peak at Rt = 13.65 min from LC-MS analysis of the reaction of peptide thioester Ac-ALKEPVHGVpSGpSA-MPAA **1a** with GRRRALKEPVHGV-NH_2 **2d** after 23 h. Target ligation product Ac-ALKEPVHGVpSGpSAGRRRALKEPVHGV-NH_2 **3a,d**. $[M+3H]^{3+}$ m/z calcd. (av.) 970.71, obs. 970.8, $[M+4H]^{4+}$ m/z calcd. (av.) 728.29, obs. 728.4, $[M+5H]^{5+}$ m/z calcd. (av.) 582.83, obs. 582.9.

1a + 2e → 3a,e:

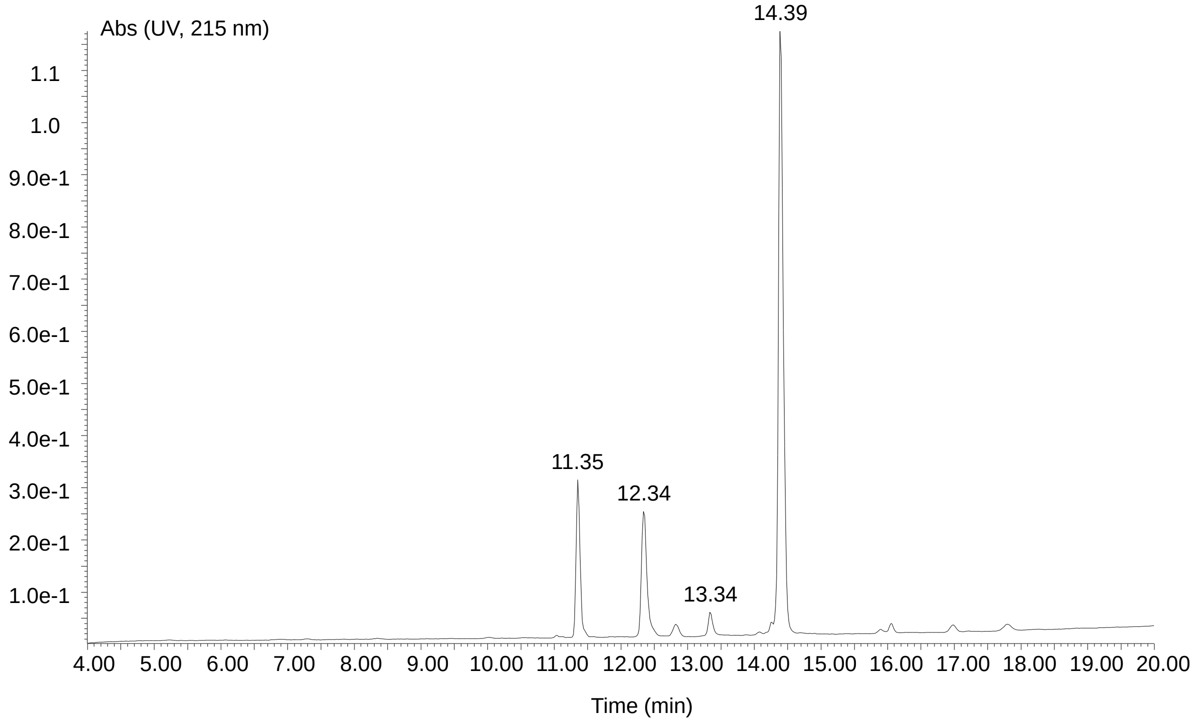

Supplementary Figure 106**.** LC-MS analysis of the reaction of peptide thioester Ac-ALKEPVHGVpSGpSA-MPAA **1a** with GRRALKEPVHGV-NH_2 **2e** after 23 h. LC trace. Eluent A 0.1% TFA in water, eluent B 0.1% TFA in CH_3CN. C18 X bridge BEH 300 Å (5 µm, 4.6 × 250 mm) column, gradient 0-50% B in 30 min, 1 mL min^{-1}, detection at 215 nm).

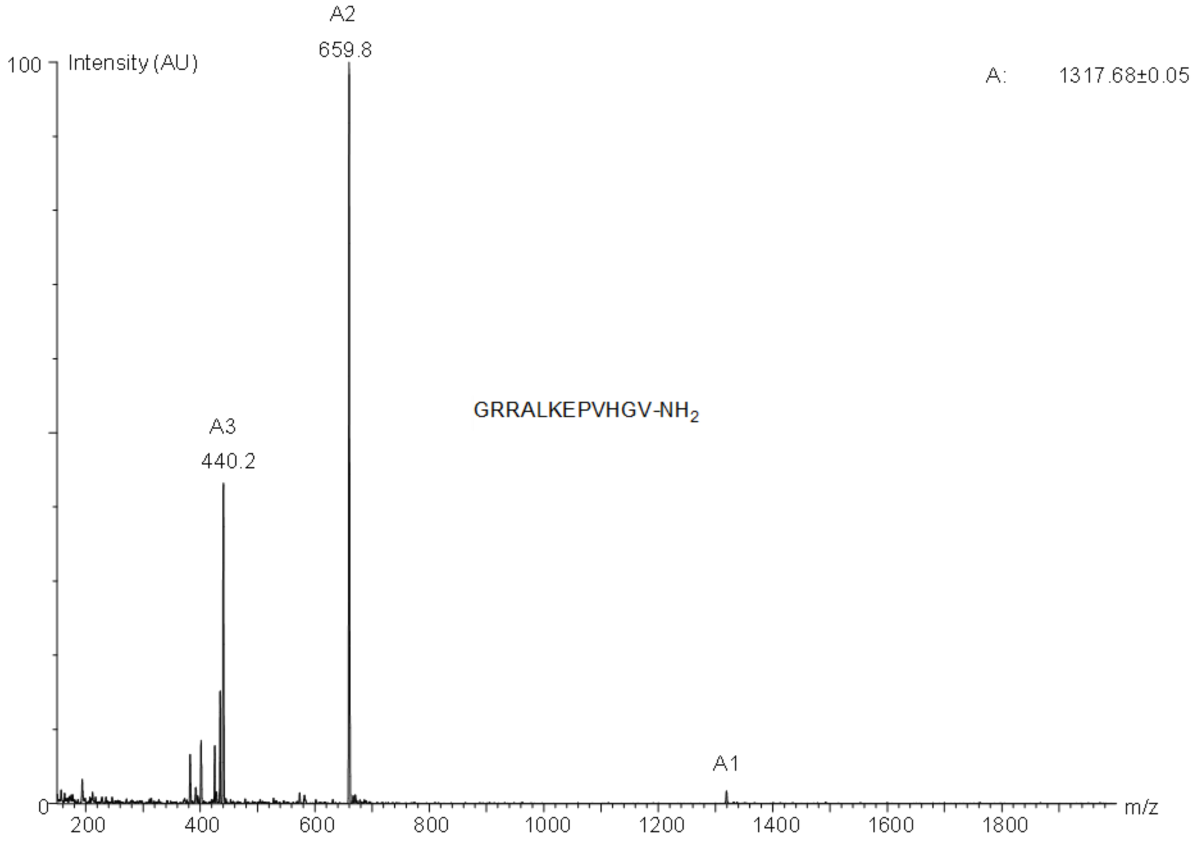

Supplementary Figure 107. MS trace of peak at Rt = 11.35 min from LC-MS analysis of the reaction of peptide thioester Ac-ALKEPVHGVpSGpSA-MPAA **1a** with GRRALKEPVHGV-NH_2 **2e** after 23 h. GRRALKEPVHGV-NH_2 **2e**. $[M+2H]^{2+}$ m/z calcd. (av.) 659.78, obs. 659.8, $[M+3H]^{3+}$ m/z calcd. (av.) 440.19, obs. 440.2.

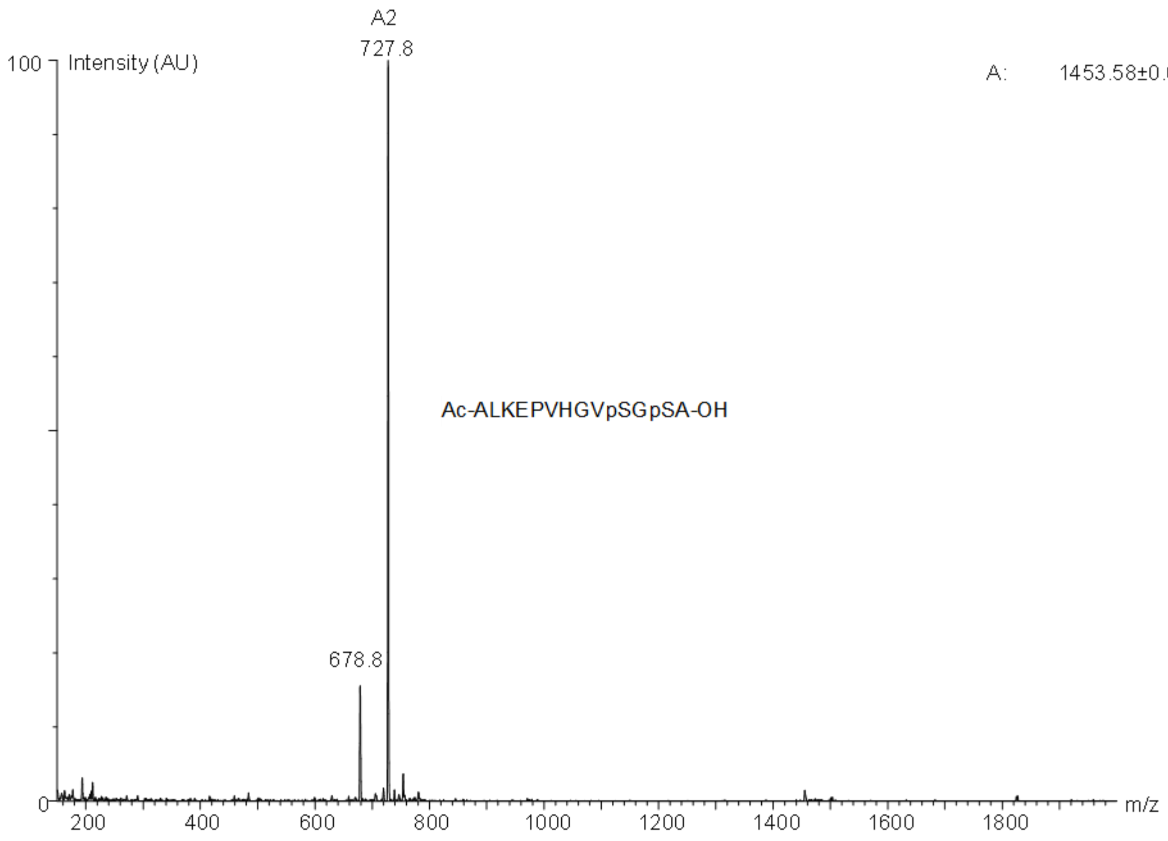

Supplementary Figure 108. MS trace of peak at Rt = 12.34 min from LC-MS analysis of the reaction of peptide thioester Ac-ALKEPVHGVpSGpSA-MPAA **1a** with GRRALKEPVHGV-NH_2 **2e** after 23 h. Peptide thioester hydrolysis byproduct Ac-ALKEPVHGVpSGpSA-OH. $[M+2H]^{2+}$ m/z calcd. (av.) 727.70, obs. 727.8.

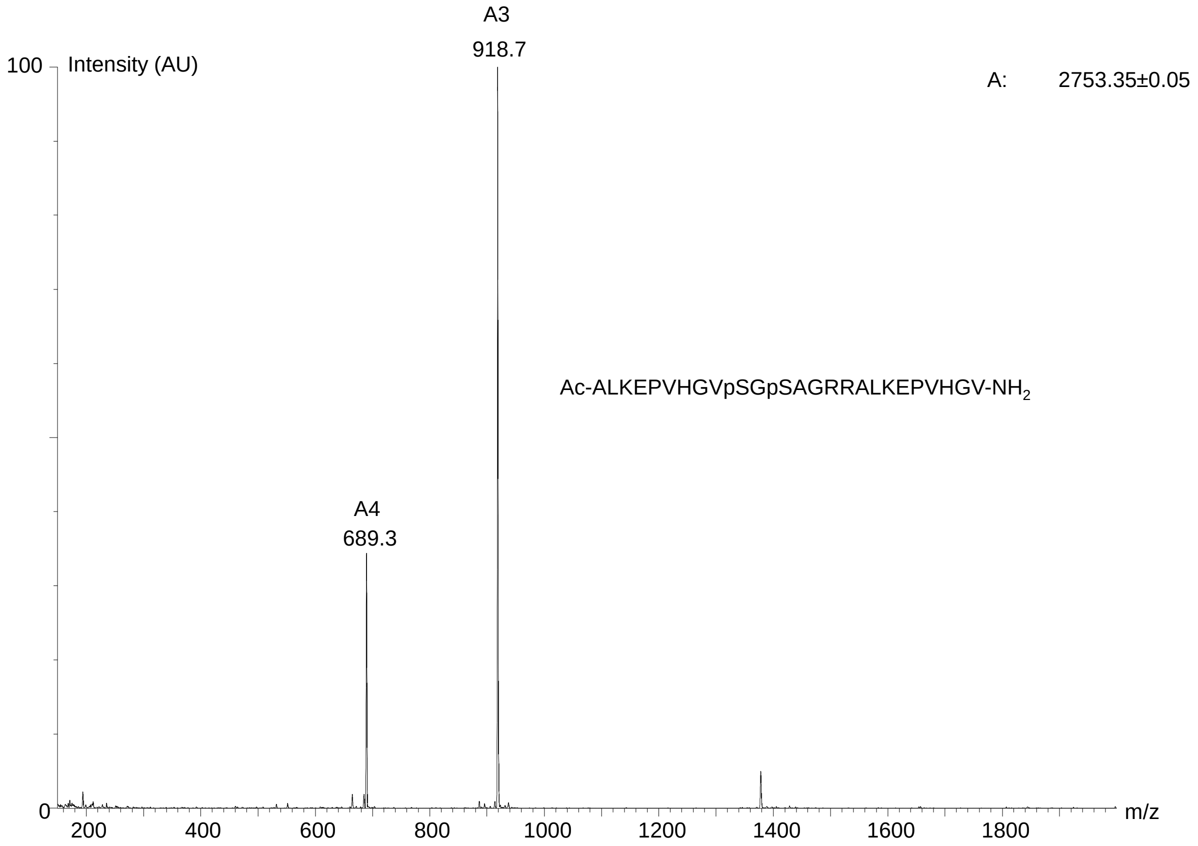

Supplementary Figure 109. MS trace of peak at Rt = 14.39 min from LC-MS analysis of the reaction of peptide thioester Ac-ALKEPVHGVpSGpSA-MPAA **1a** with GRRALKEPVHGV-NH_2 **2e** after 23 h. Target ligation product Ac-ALKEPVHGVpSGpSAGRRALKEPVHGV-NH_2 **3a,e**. $[M+3H]^{3+}$ m/z calcd. (av.) 918.55, obs. 918.7, $[M+4H]^{4+}$ m/z calcd. (av.) 689.24, obs. 689.3.

1a + 2f → 3a,f:

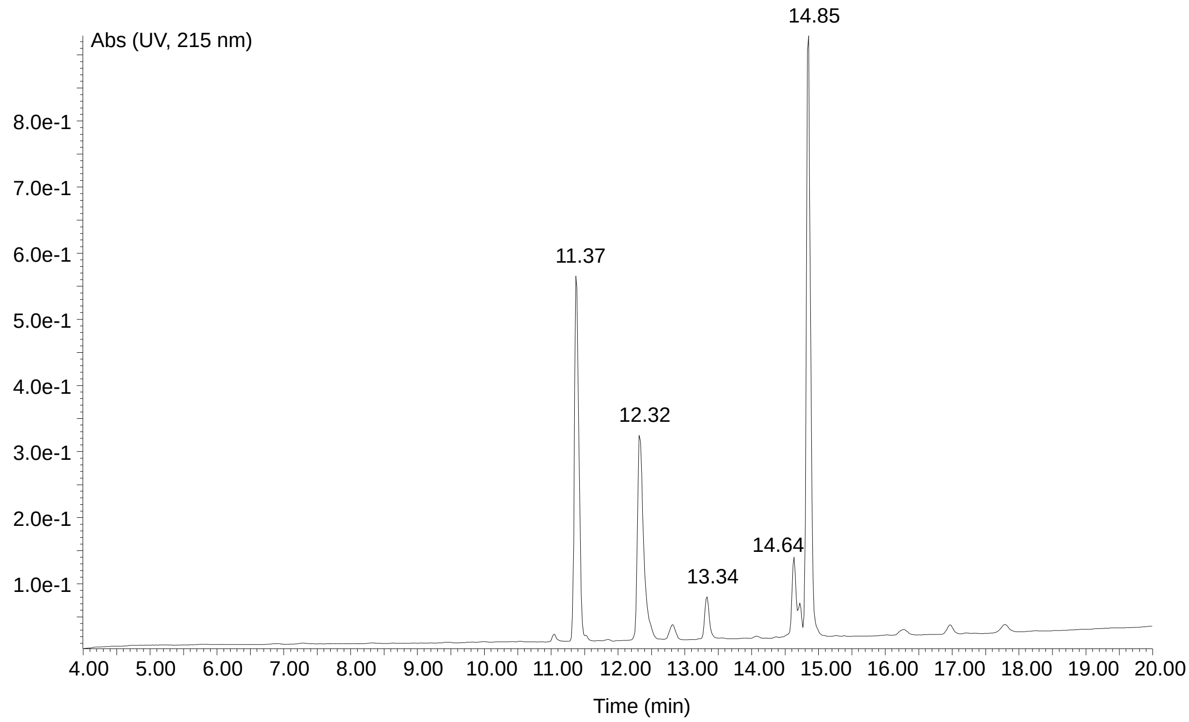

Supplementary Figure 110. LC-MS analysis of the reaction of peptide thioester Ac-ALKEPVHGVpSGpSA-MPAA **1a** with peptide GRALKEPVHGV-NH_2 **2f** after 23 h. LC trace. Eluent A 0.1% TFA in water, eluent B 0.1% TFA in CH_3CN. C18 X bridge BEH 300 Å (5 µm, 4.6 × 250 mm) column, gradient 0-50% B in 30 min, 1 mL min^{-1}, detection at 215 nm).

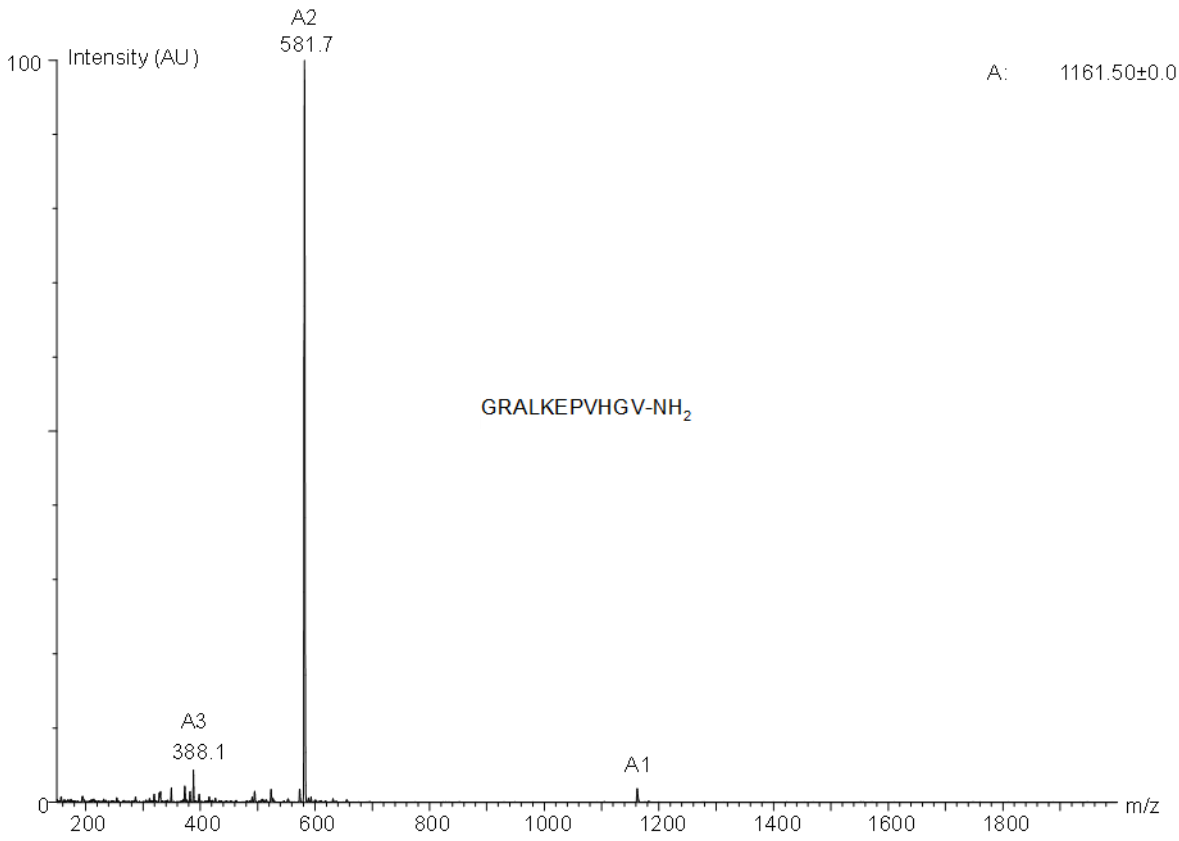

Supplementary Figure 111. MS trace of peak at Rt = 11.37 min from LC-MS analysis of the reaction of peptide thioester Ac-ALKEPVHGVpSGpSA-MPAA **1a** with peptide GRALKEPVHGV-NH_2 **2f** after 23 h. GRALKEPVHGV-NH_2 **2f**. $[M+2H]^{2+}$ m/z calcd. (av.) 581.69, obs. 581.7, $[M+3H]^{3+}$ m/z calcd. (av.) 388.13, obs. 388.1.

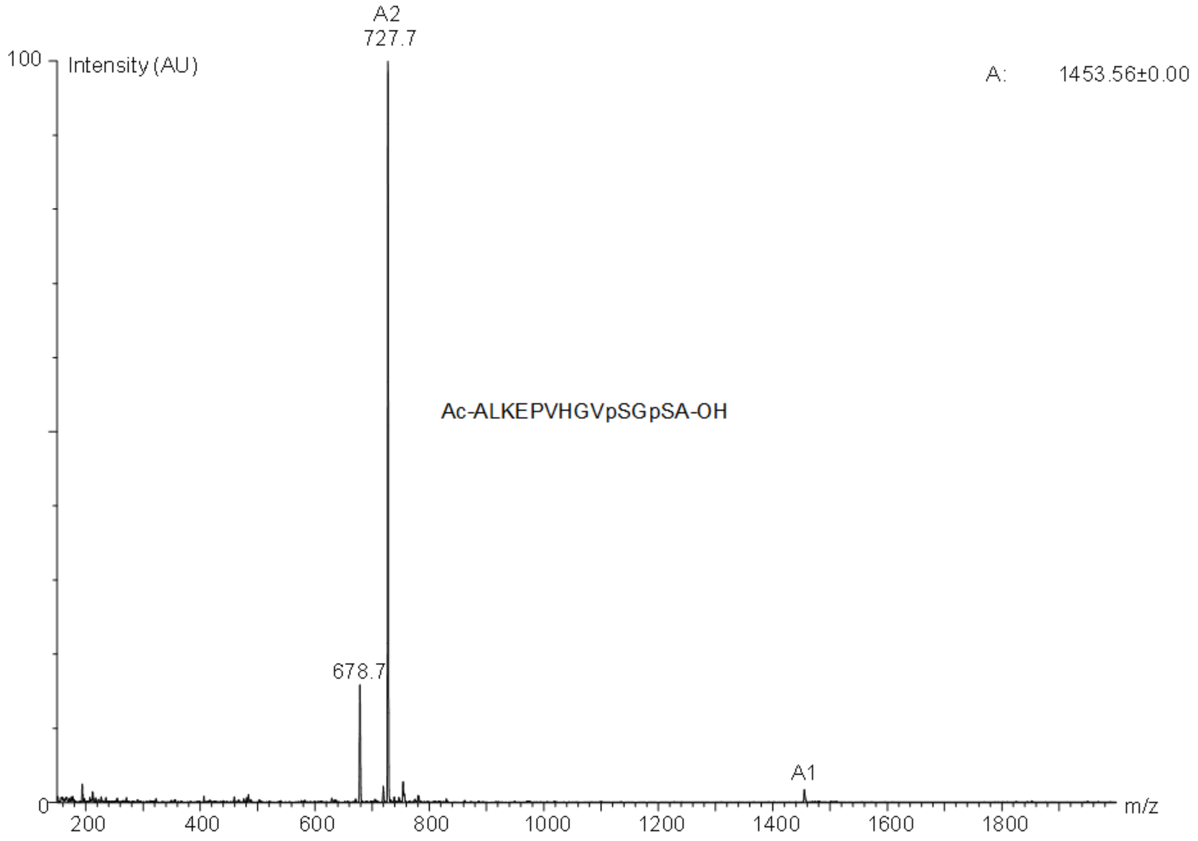

Supplementary Figure 112. MS trace of peak at Rt = 12.32 min from LC-MS analysis of the reaction of peptide thioester Ac-ALKEPVHGVpSGpSA-MPAA **1a** with peptide GRALKEPVHGV-NH_2 **2f** after 23 h. Peptide thioester hydrolysis byproduct Ac-ALKEPVHGVpSGpSA-OH. $[M+2H]^{2+}$ m/z calcd. (av.) 727.7, obs. 727.7.

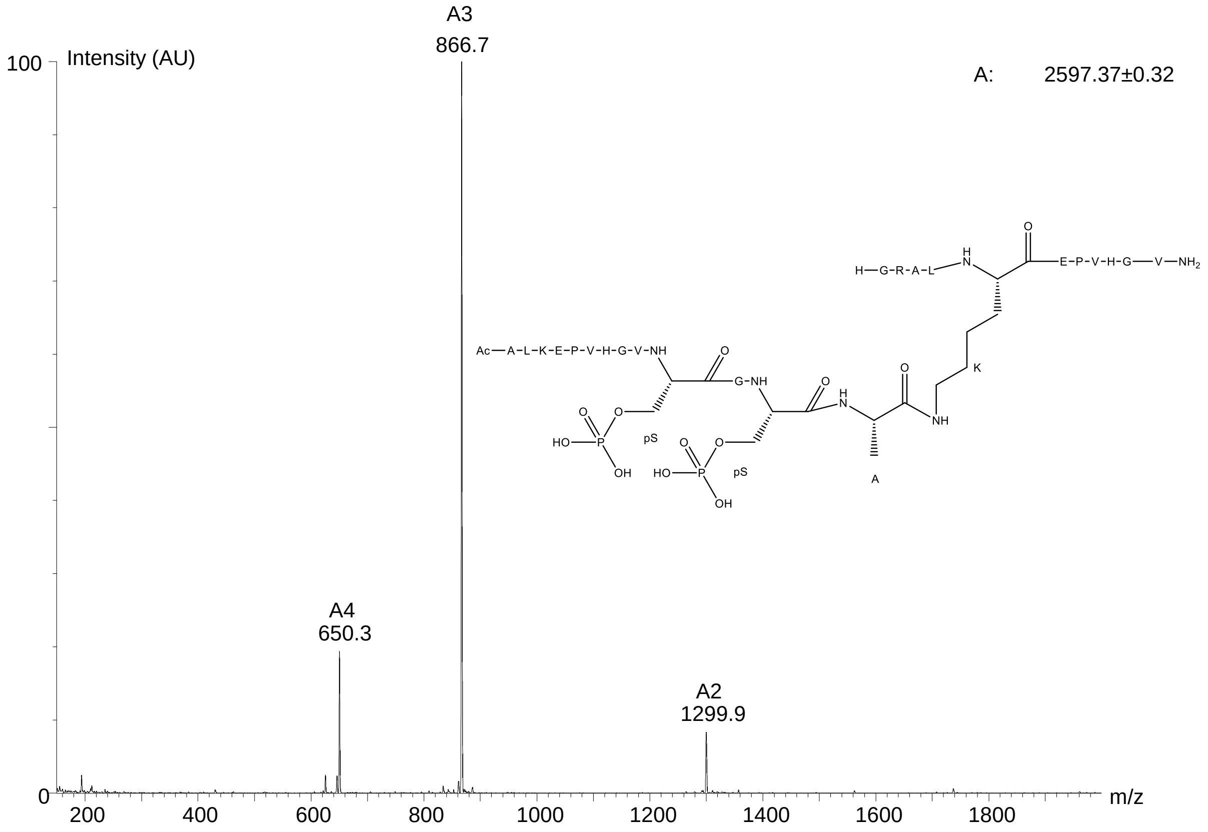

Supplementary Figure 113. MS trace of peak at Rt = 14.64 min from LC-MS analysis of the reaction of peptide thioester Ac-ALKEPVHGVpSGpSA-MPAA **1a** with peptide GRALKEPVHGV-NH_2 **2f** after 23 h. Branched byproduct GRALK(Ac-ALKEPVHGVpSGpSA)EPVHGV-NH_2. $[M+2H]^{2+}$ m/z calcd. (av.) 1299.38, obs. 1299.9, $[M+3H]^{3+}$ m/z calcd. (av.) 866.59, obs. 866.7, $[M+4H]^{4+}$ m/z calcd. (av.) 650.19, obs. 650.3.

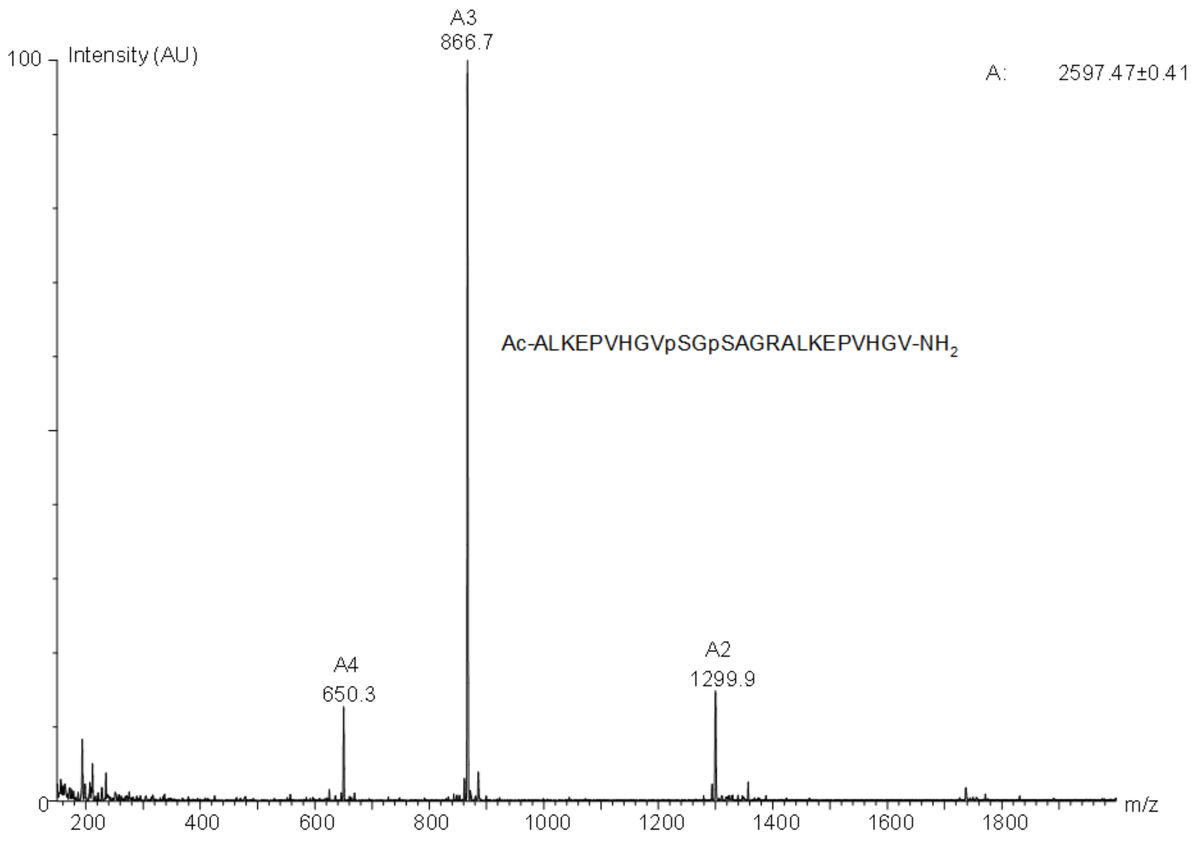

Supplementary Figure 114. MS trace of peak at Rt = 14.85 min from LC-MS analysis of the reaction of peptide thioester Ac-ALKEPVHGVpSGpSA-MPAA **1a** with peptide GRALKEPVHGV-NH_2 **2f** after 23 h. Target ligation product Ac-ALKEPVHGVpSGpSAGRALKEPVHGV-NH_2 **3a,f**. $[M+2H]^{2+}$ m/z calcd. (av.) 1299.38, obs. 1299.9, $[M+3H]^{3+}$ m/z calcd. (av.) 866.59, obs. 866.7, $[M+4H]^{4+}$ m/z calcd. (av.) 650.19, obs. 650.3.

1a + 2g → 3a,g:

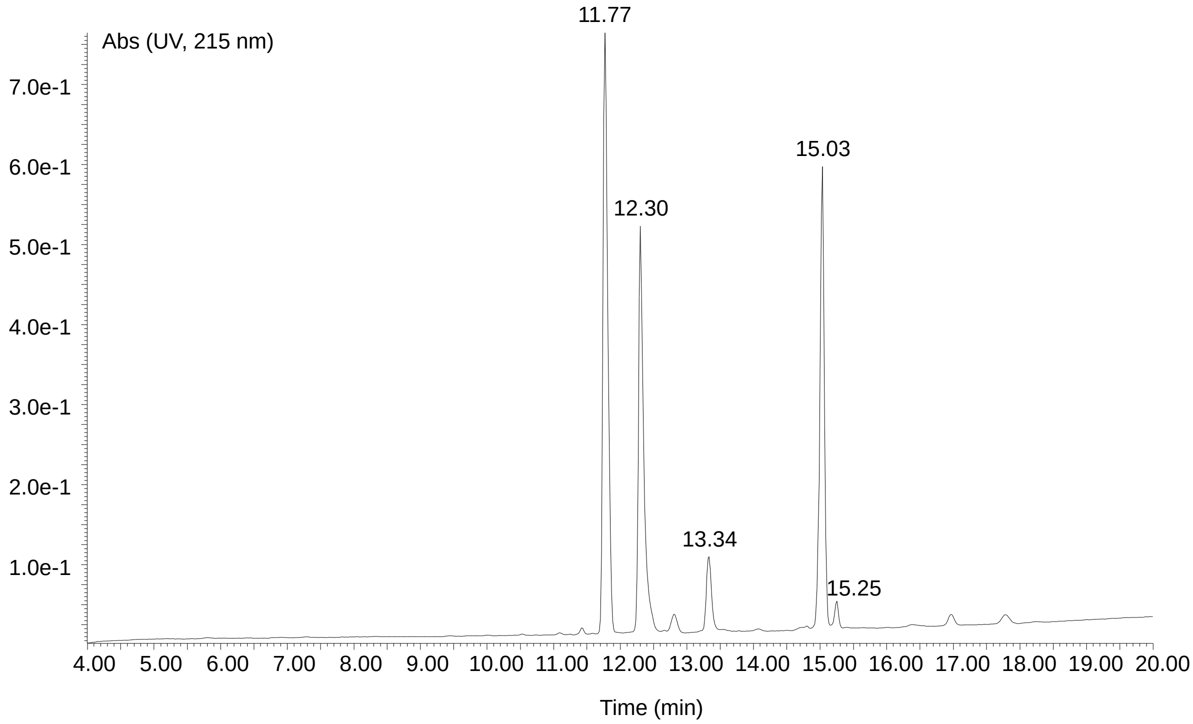

Supplementary Figure 115. LC-MS analysis of the reaction of peptide thioester Ac-ALKEPVHGVpSGpSA-MPAA **1a** with peptide GALKEPVHGV-NH_2 **2g** after 23 h. LC trace. Eluent A 0.1% TFA in water, eluent B 0.1% TFA in CH_3CN. C18 X bridge BEH 300 Å (5 µm, 4.6 × 250 mm) column, gradient 0-50% B in 30 min, 1 mL min^{-1}, detection at 215 nm).

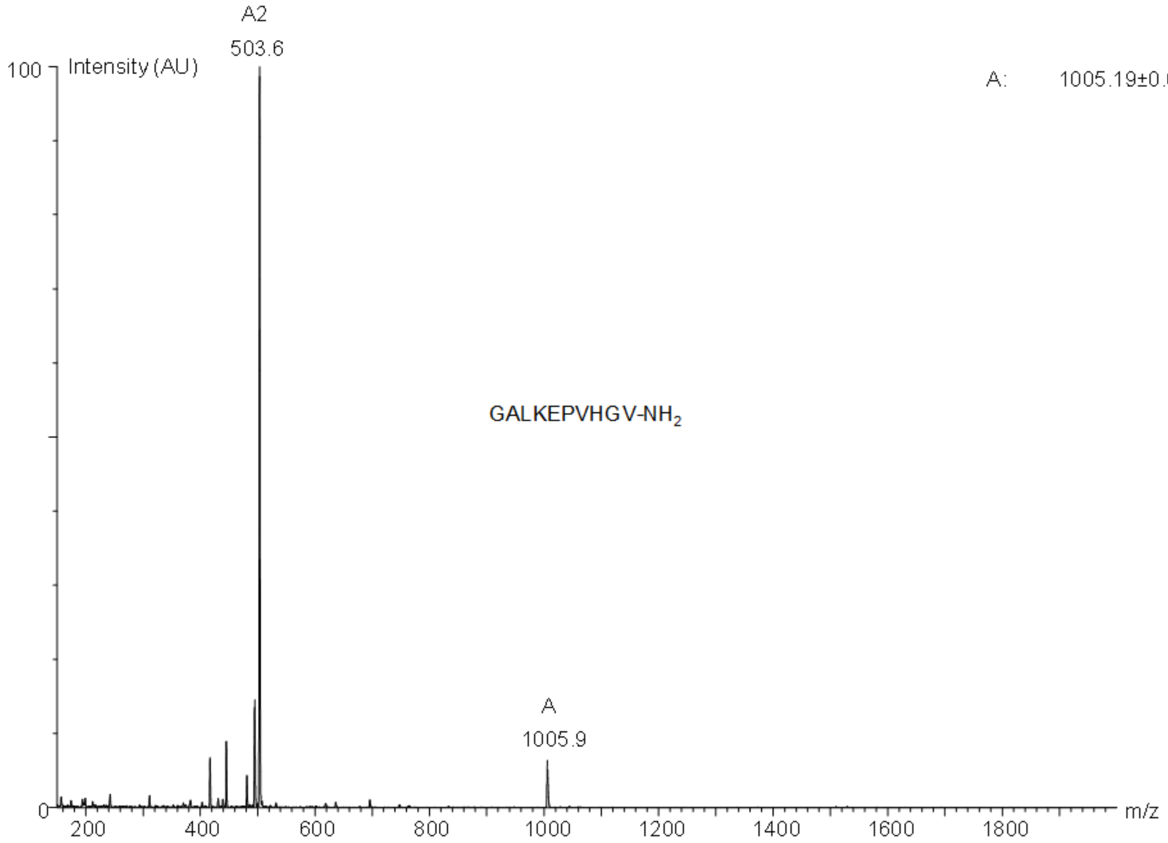

Supplementary Figure 116. MS trace of peak at Rt = 11.77 min from LC-MS analysis of the reaction of peptide thioester Ac-ALKEPVHGVpSGpSA-MPAA **1a** with peptide GALKEPVHGV-NH_2 **2g** after 23 h. GALKEPVHGV-NH_2 **2g**. $[M+H]^+$ m/z calcd. (monoisotopic) 1005.58, obs. 1005.9, $[M+2H]^{2+}$ m/z calcd. (av.) 503.59, obs. 503.6.

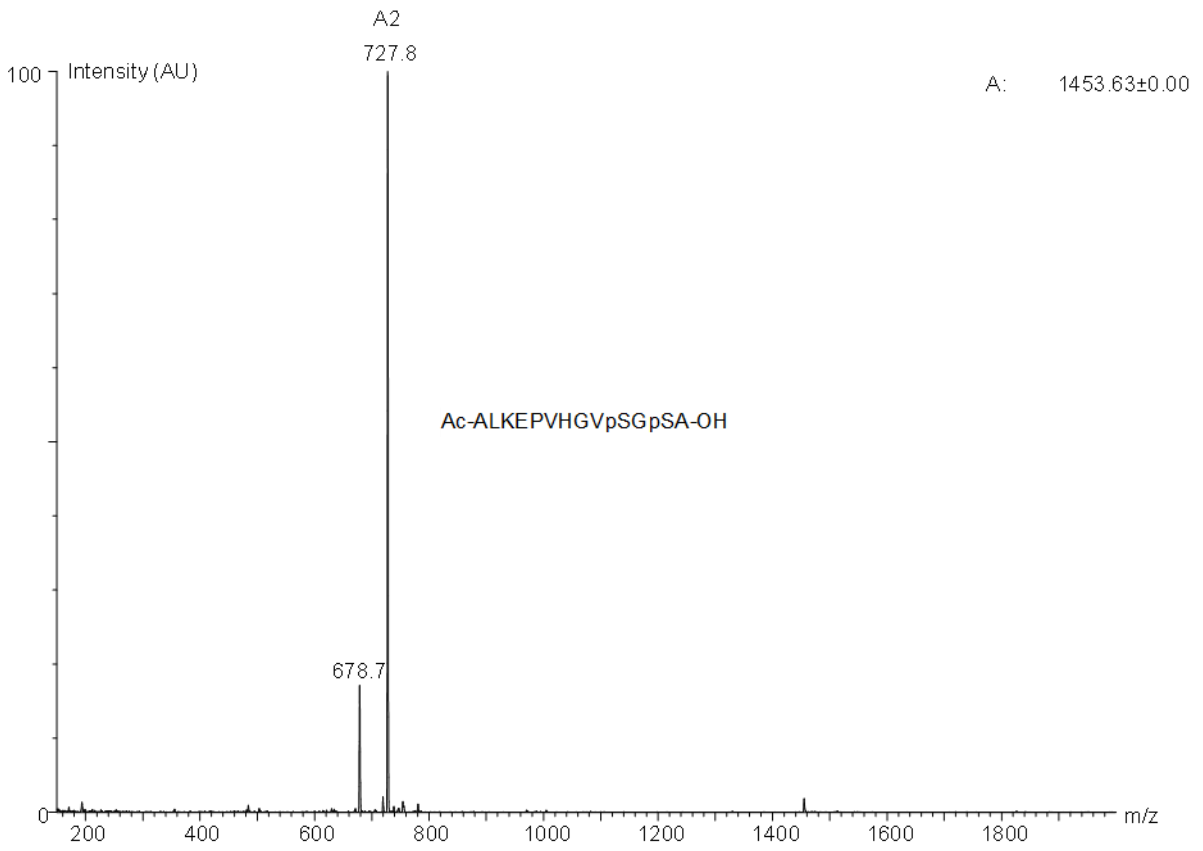

Supplementary Figure 117. MS trace of peak at Rt = 12.30 min from LC-MS analysis of the reaction of peptide thioester Ac-ALKEPVHGVpSGpSA-MPAA **1a** with peptide GALKEPVHGV-NH_2 **2g** after 23 h. Peptide thioester hydrolysis byproduct Ac-ALKEPVHGVpSGpSA-OH. $[M+2H]^{2+}$ m/z calcd. (av.) 727.7, obs. 727.8.

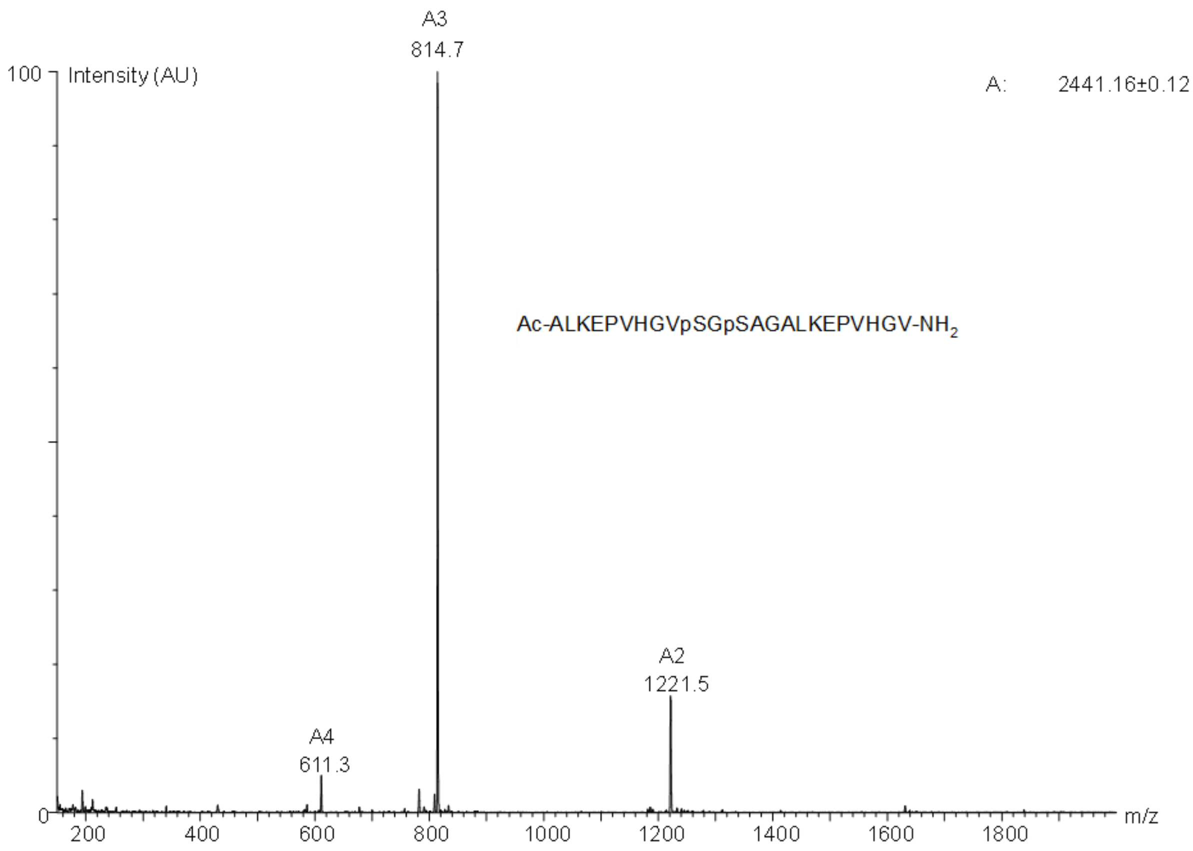

Supplementary Figure 118. MS trace of peak at Rt = 15.03 min from LC-MS analysis of the reaction of peptide thioester Ac-ALKEPVHGVpSGpSA-MPAA **1a** with peptide GALKEPVHGV-NH_2 **2g** after 23 h. Target ligation product Ac-ALKEPVHGVpSGpSAGALKEPVHGV-NH_2 **3a,g**. $[M+2H]^{2+}$ m/z calcd. (av.) 1221.29, obs. 1221.5, $[M+3H]^{3+}$ m/z calcd. (av.) 814.52, obs. 814.7, $[M+4H]^{4+}$ m/z calcd. (av.) 611.14, obs. 611.3.

*Reaction of peptide thioester **1b** (one proximal pSer residue) with peptides **2a-g** (Fig. 3c)*

Ac-ALKEPVHGVSGpSA-MPAA peptide **1b** (~0.28 µmol, 5 mM final concentration) was dissolved in 50 mM sodium bicarbonate/CO_2 buffer (495 µL total) and added (55 µL each) to a series of peptides varying by the number of Arg from GRRRRRRALKEPVHGV-NH_2 **2a** to GALKEPVHGV-NH_2 **2g** (1 equiv, 5 mM final concentration, pH=7.18-7.38). Once dissolved, the plastic tubes containing the reaction mixtures were placed in a CO_2 incubator for cell biology (5% partial CO_2 pressure, 37 °C, water saturated) with a needle inserted through the cap to enable gas exchange.

After 20 h, the reaction mixtures (1 µL) were quenched with aqueous AcOH (10 % AcOH in water, 100 µL) and analyzed by LC-MS.

1b + 2a → 3b,a:

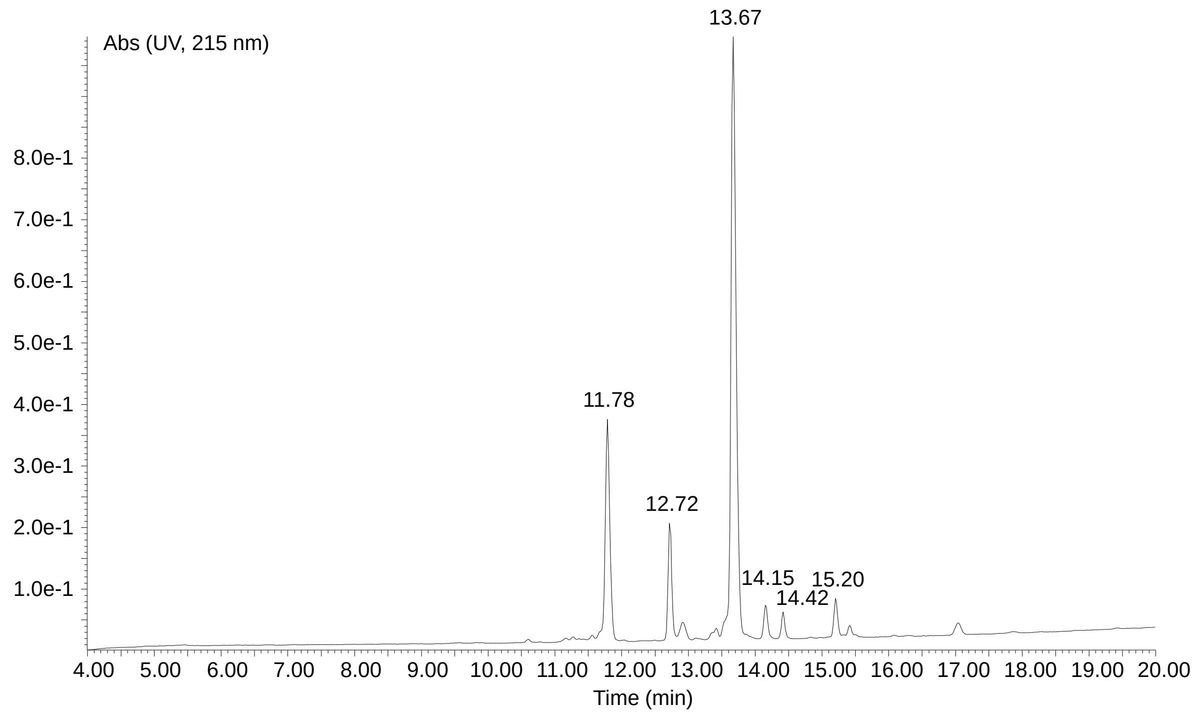

Supplementary Figure 119. LC-MS analysis of the reaction of peptide thioester Ac-ALKEPVHGVSGpSA-MPAA **1b** with peptide GRRRRRRALKEPVHGV-NH_2 **2a** after 20 h. LC trace. Eluent A 0.1% TFA in water, eluent B 0.1% TFA in CH_3CN. C18 X bridge BEH 300 Å (5 µm, 4.6 × 250 mm) column, gradient 0-50% B in 30 min, 1 mL min^{-1}, detection at 215 nm).

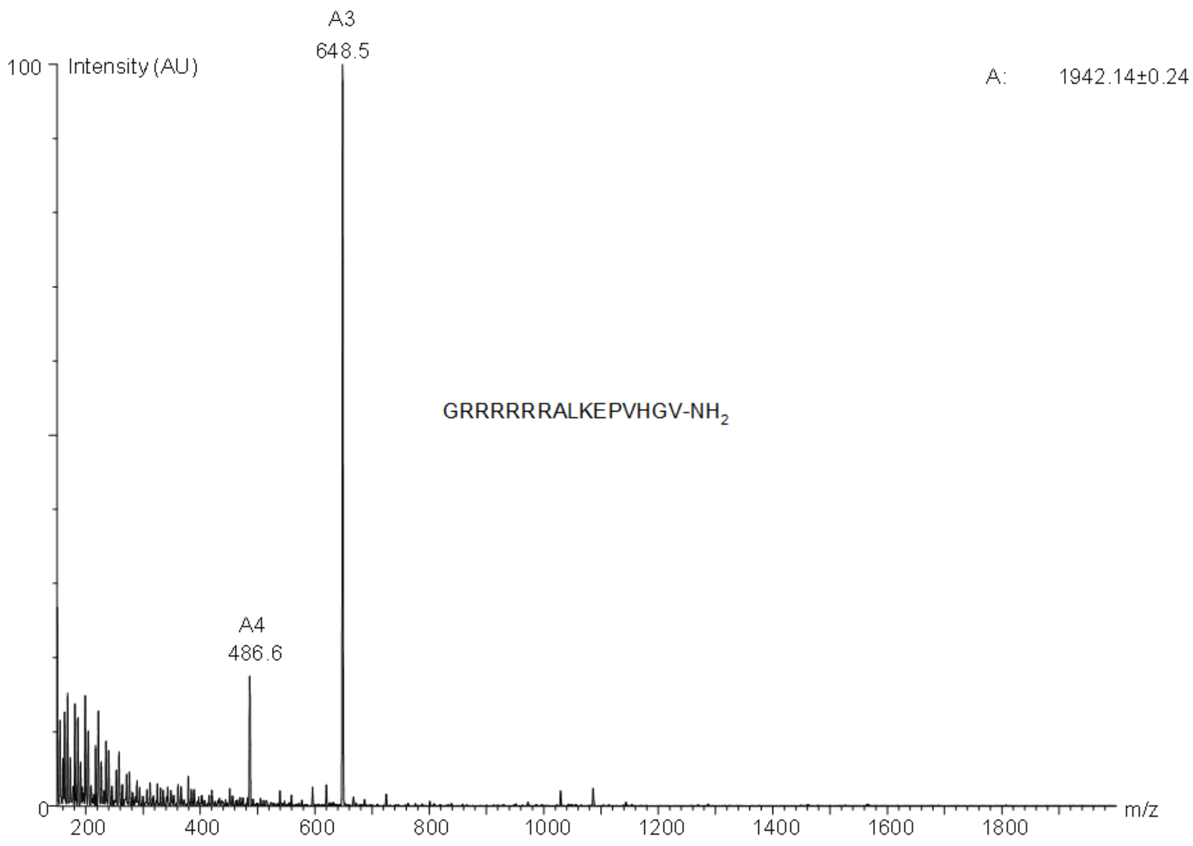

Supplementary Figure 120. MS trace of peak at Rt = 11.78 min from LC-MS analysis of the reaction of peptide thioester Ac-ALKEPVHGVSGpSA-MPAA **1b** with peptide GRRRRRRALKEPVHGV-NH_2 **2a** after 20 h. GRRRRRRALKEPVHGV-NH_2 **2a**. $[M+3H]^{3+}$ m/z calcd. (av.) 648.44, obs. 648.5, $[M+4H]^{4+}$ m/z calcd. (av.) 486.58, obs. 486.6.

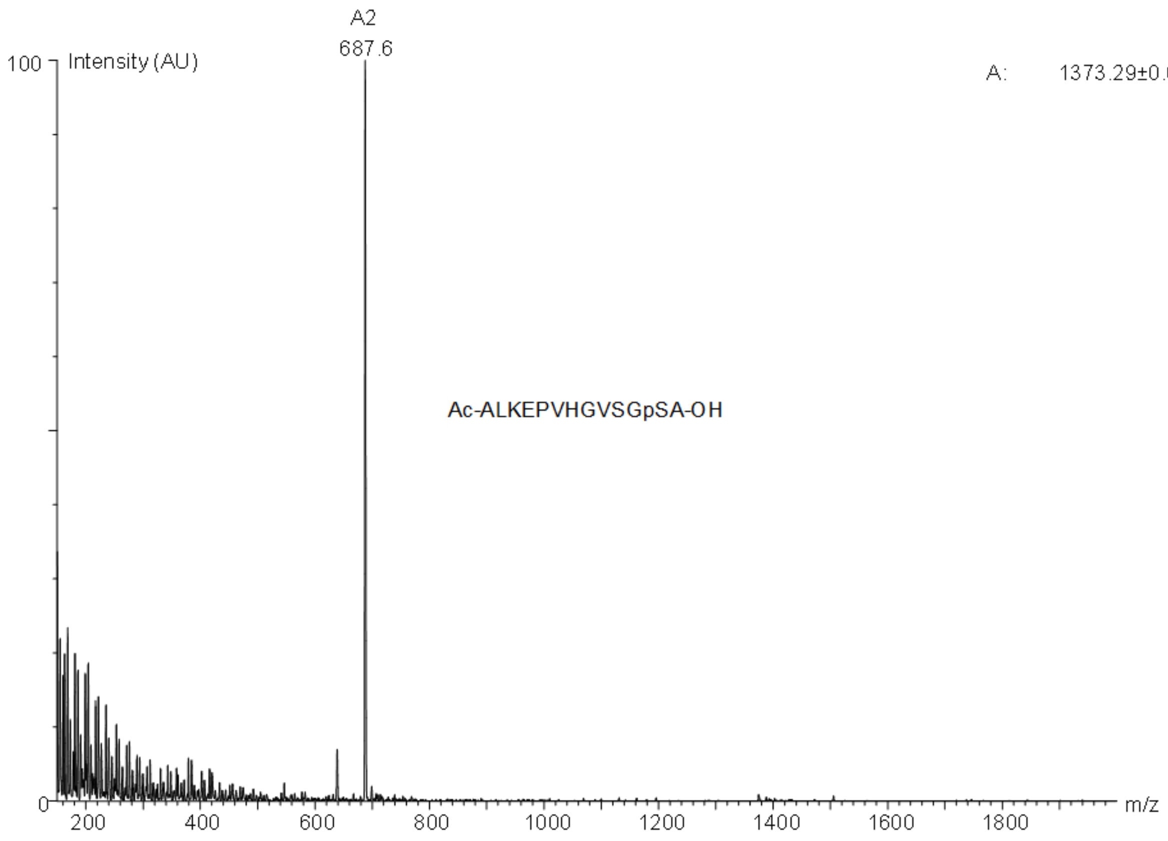

Supplementary Figure 121. MS trace of peak at Rt = 12.72 min from LC-MS analysis of the reaction of peptide thioester Ac-ALKEPVHGVSGpSA-MPAA **1b** with peptide GRRRRRRALKEPVHGV-NH_2 **2a** after 20 h. Peptide thioester hydrolysis byproduct Ac-ALKEPVHGVSGpSA-OH. $[M+2H]^{2+}$ m/z calcd. (av.) 687.71, obs. 687.6.

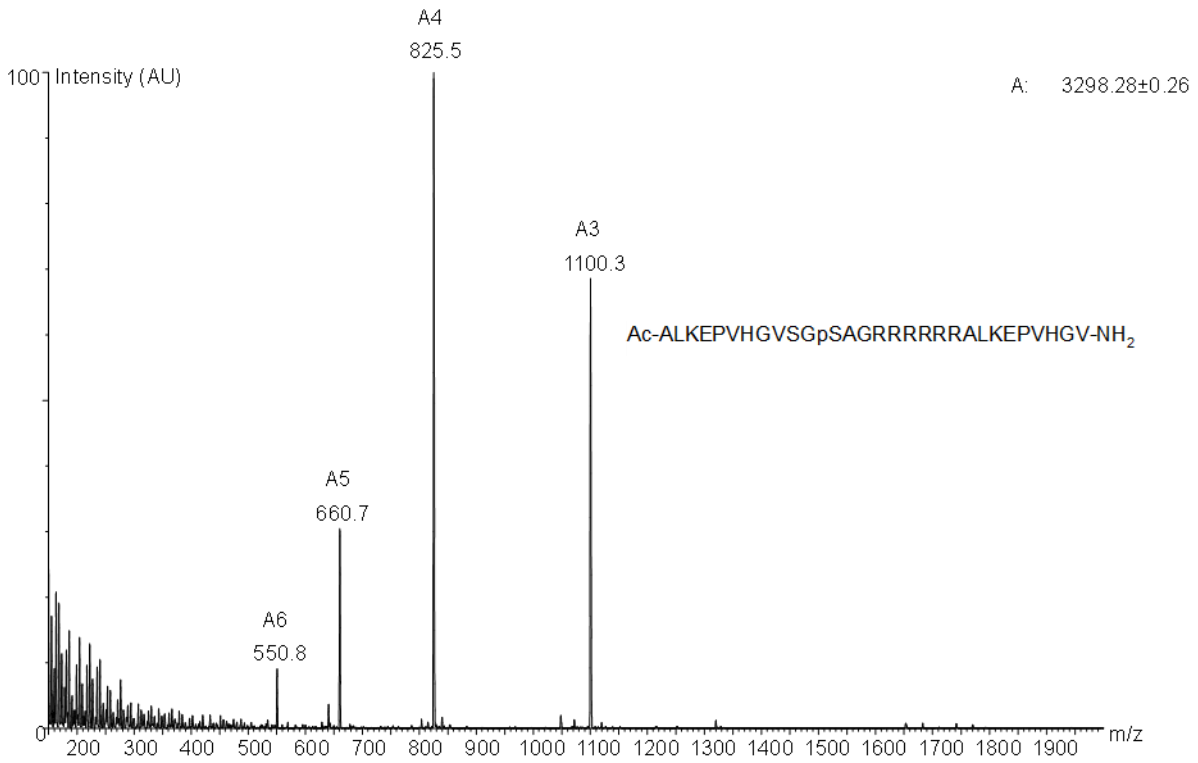

Supplementary Figure 122. MS trace of peak at Rt = 13.67 min from LC-MS analysis of the reaction of peptide thioester Ac-ALKEPVHGVSGpSA-MPAA **1b** with peptide GRRRRRRALKEPVHGV-NH_2 **2a** after 20 h. Target ligation product Ac-ALKEPVHGVSGpSAGRRRRRRALKEPVHGV-NH_2 **3b,a**. $[M+3H]^{3+}$ m/z calcd. (av.) 1100.24, obs. 1100.3, $[M+4H]^{4+}$ m/z calcd. (av.) 825.43, obs. 825.5, $[M+5H]^{5+}$ m/z calcd. (av.) 660.55, obs. 660.7, $[M+6H]^{6+}$ m/z calcd. (av.) 550.62, obs. 550.8.

1b + 2b → 3b,b:

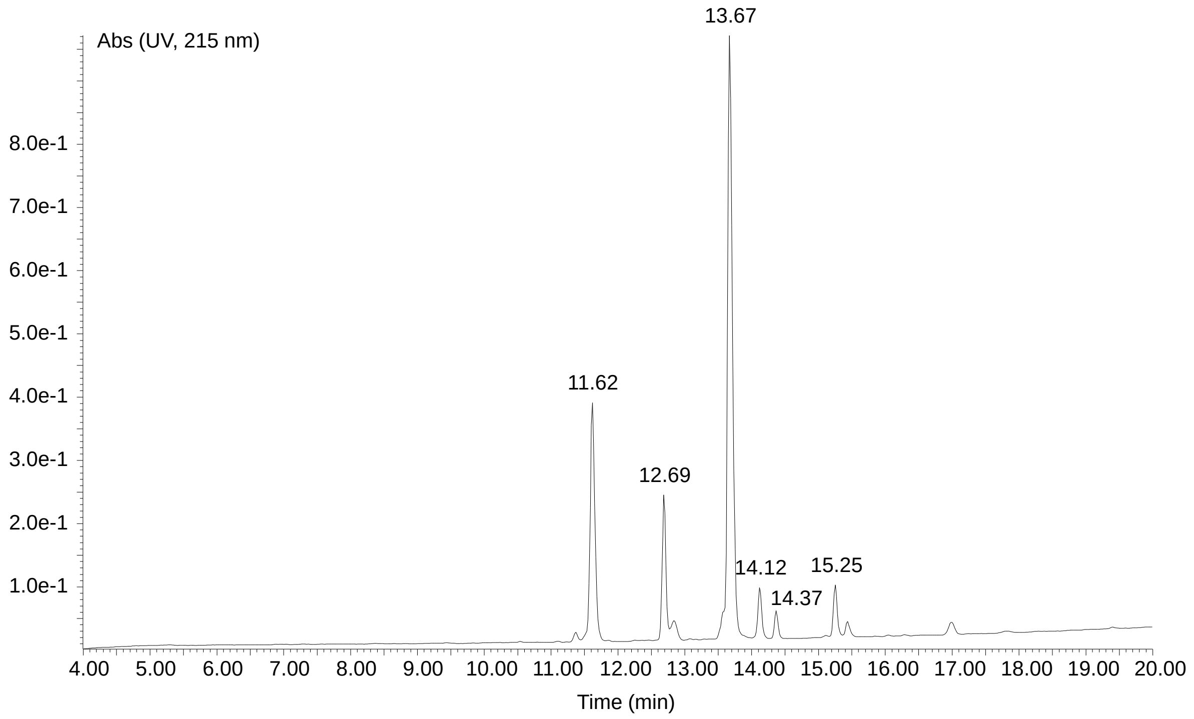

Supplementary Figure 123**.** LC-MS analysis of the reaction of peptide thioester Ac-ALKEPVHGVSGpSA-MPAA **1b** with peptide GRRRRRALKEPVHGV-NH_2 **2b** after 20 h. LC trace. Eluent A 0.1% TFA in water, eluent B 0.1% TFA in CH_3CN. C18 X bridge BEH 300 Å (5 µm, 4.6 × 250 mm) column, gradient 0-50% B in 30 min, 1 mL min^{-1}, detection at 215 nm).

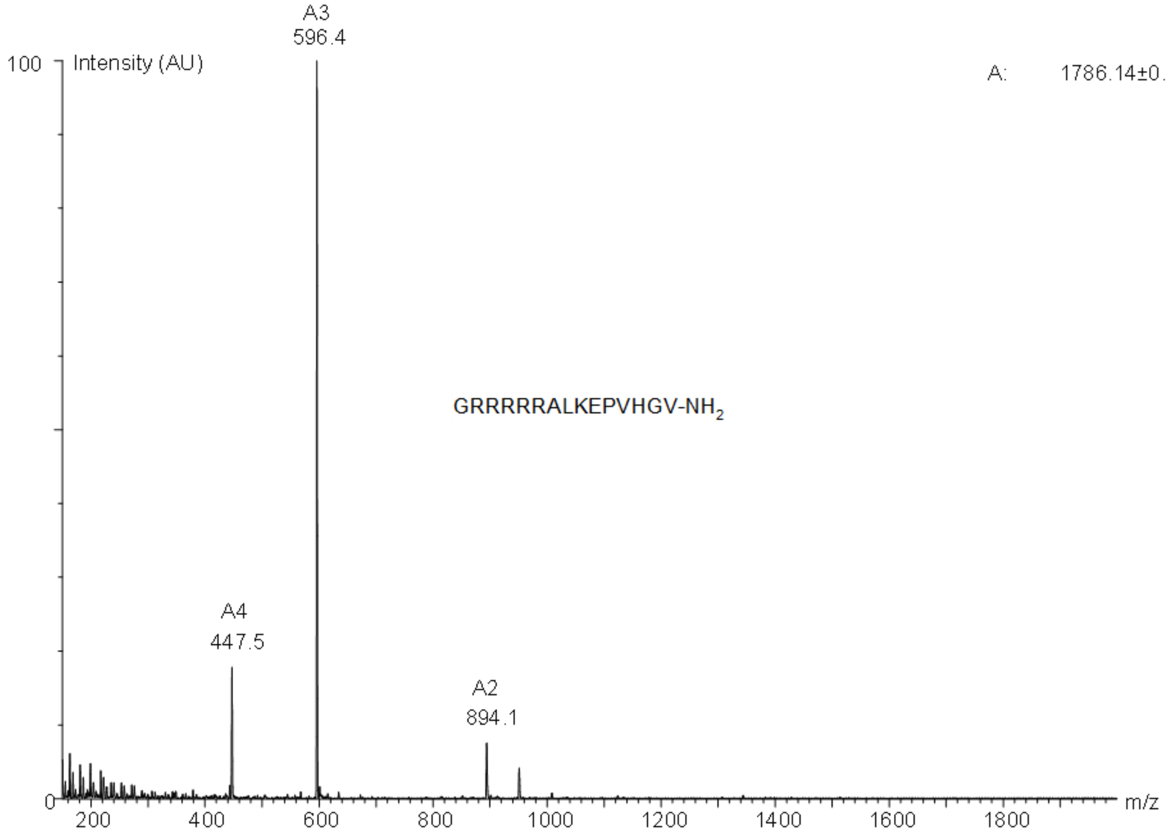

Supplementary Figure 124. MS trace of peak at Rt = 11.62 min from LC-MS analysis of the reaction of peptide thioester Ac-ALKEPVHGVSGpSA-MPAA **1b** with peptide GRRRRRALKEPVHGV-NH_2 **2b** after 20 h. GRRRRRALKEPVHGV-NH_2 **2b**. $[M+2H]^{2+}$ m/z calcd. (av.) 894.07, obs. 894.1, $[M+3H]^{3+}$ m/z calcd. (av.) 596.38, obs. 596.4, $[M+4H]^{4+}$ m/z calcd. (av.) 447.53, obs. 447.5.

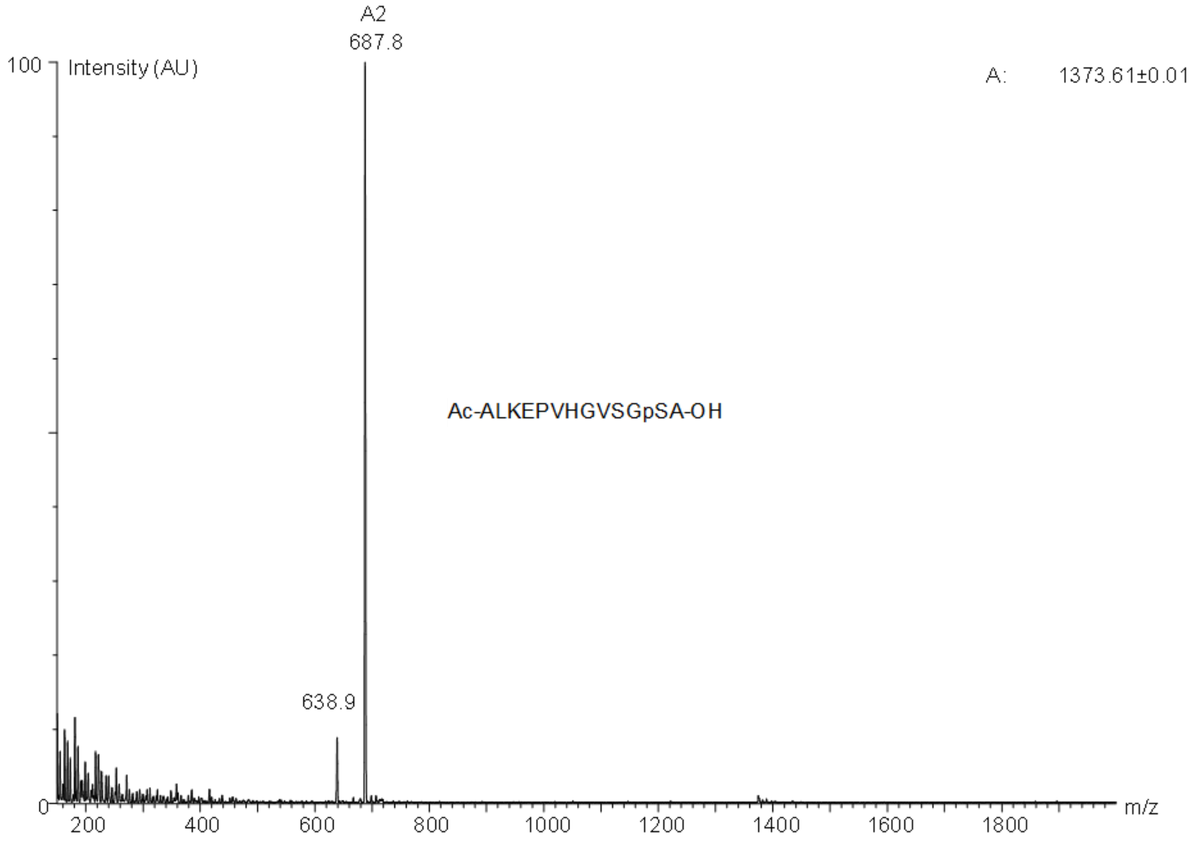

Supplementary Figure 125. MS trace of peak at Rt = 12.69 min from LC-MS analysis of the reaction of peptide thioester Ac-ALKEPVHGVSGpSA-MPAA **1b** with peptide GRRRRRALKEPVHGV-NH_2 **2b** after 20 h. Peptide thioester hydrolysis byproduct Ac-ALKEPVHGVSGpSA-OH. $[M+2H]^{2+}$ m/z calcd. (av.) 687.71, obs. 687.8.

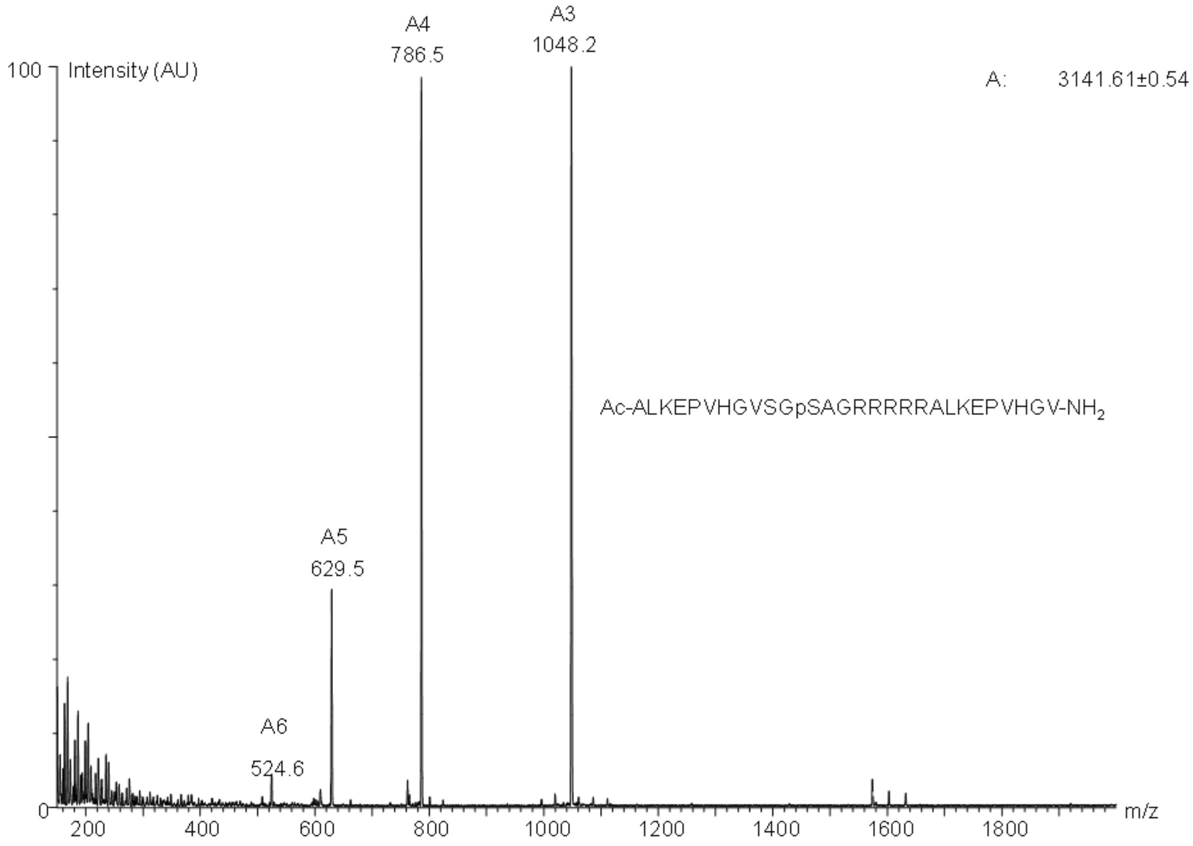

Supplementary Figure 126. MS trace of peak at Rt = 13.67 min from LC-MS analysis of the reaction of peptide thioester Ac-ALKEPVHGVSGpSA-MPAA **1b** with peptide GRRRRRALKEPVHGV-NH_2 **2b** after 20 h. Target ligation product Ac-ALKEPVHGVSGpSAGRRRRRALKEPVHGV-NH_2 **3b,b**. $[M+3H]^{3+}$ m/z calcd. (av.) 1048.18, obs. 1048.2, $[M+4H]^{4+}$ m/z calcd. (av.) 786.38, obs. 786.5, $[M+5H]^{5+}$ m/z calcd. (av.) 629.31, obs. 629.5, $[M+6H]^{6+}$ m/z calcd. (av.) 524.59, obs. 524.6

1b + 2c → 3b,c:

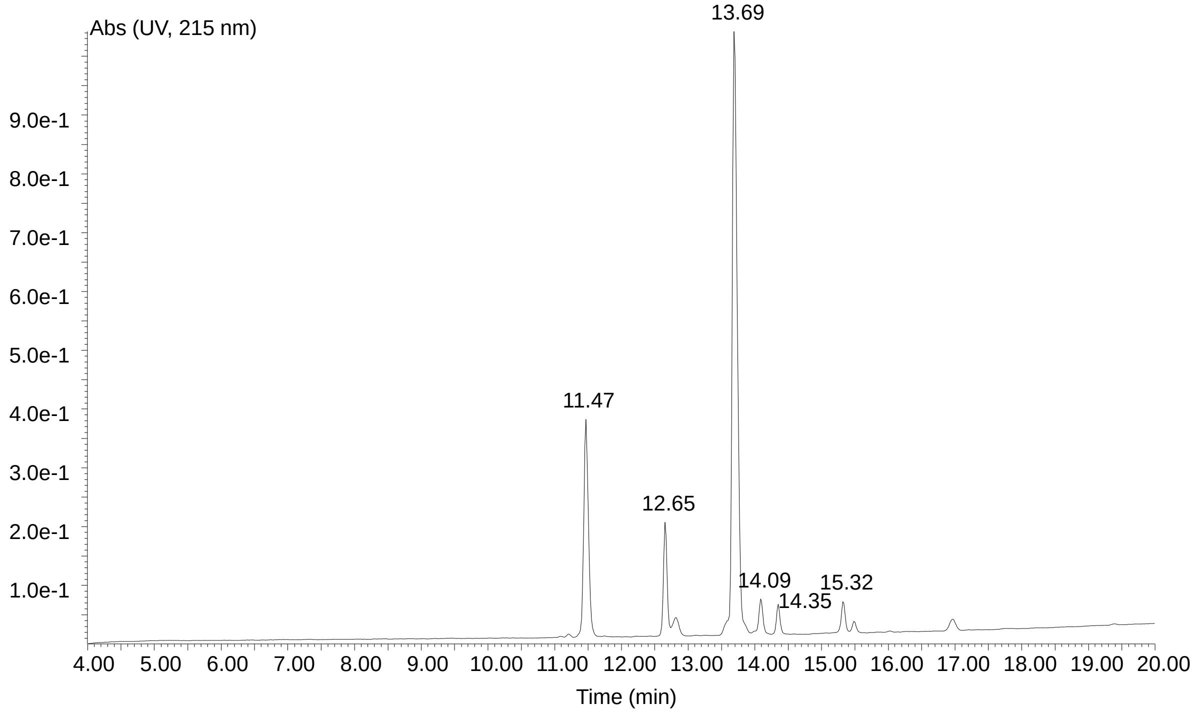

Supplementary Figure 127**.** LC-MS analysis of the reaction of peptide thioester Ac-ALKEPVHGVSGpSA-MPAA **1b** with peptide GRRRRALKEPVHGV-NH_2 **2c** after 20 h. LC trace. Eluent A 0.1% TFA in water, eluent B 0.1% TFA in CH_3CN. C18 X bridge BEH 300 Å (5 µm, 4.6 × 250 mm) column, gradient 0-50% B in 30 min, 1 mL min^{-1}, detection at 215 nm).

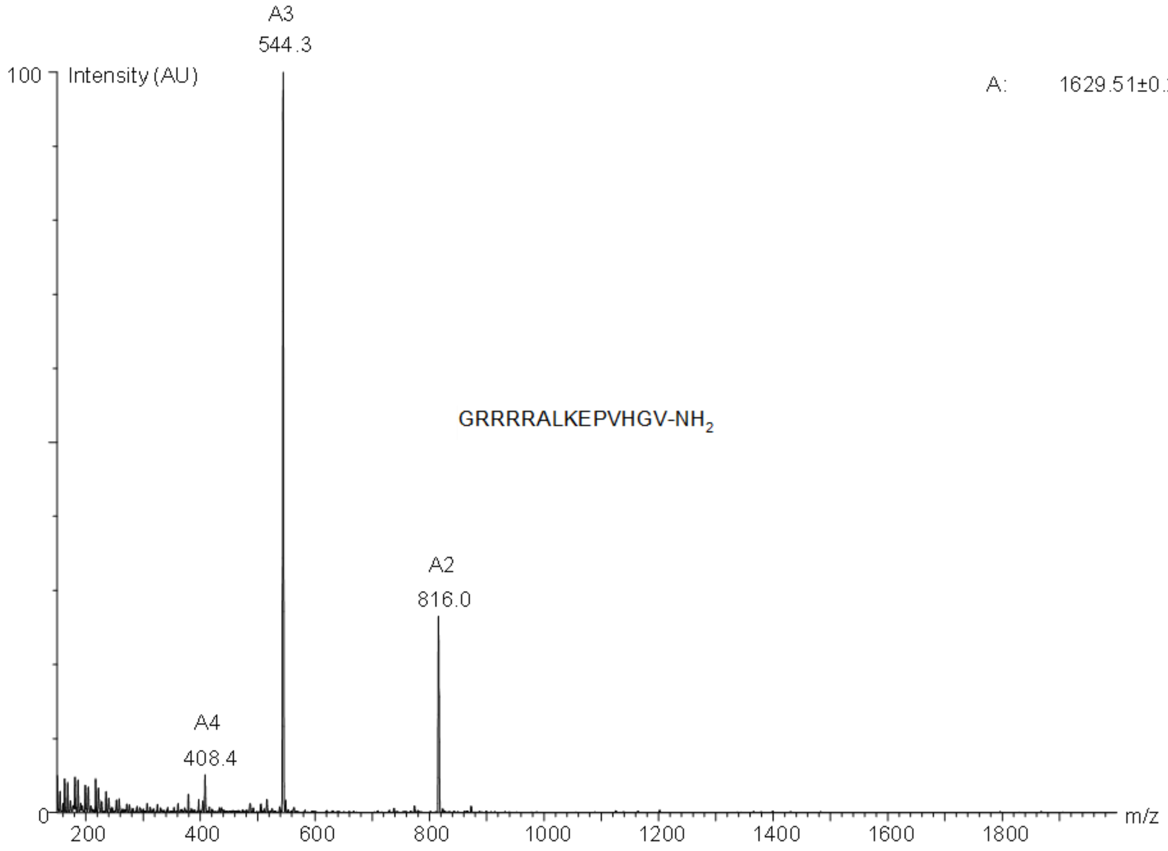

Supplementary Figure 128. MS trace of peak at Rt = 11.47 min from LC-MS analysis of the reaction of peptide thioester Ac-ALKEPVHGVSGpSA-MPAA **1b** with peptide GRRRRALKEPVHGV-NH_2 **2c** after 20 h. GRRRRALKEPVHGV-NH_2 **2c**. $[M+2H]^{2+}$ m/z calcd. (av.) 815.97, obs. 816.0, $[M+3H]^{3+}$ m/z calcd. (av.) 544.31, obs. 544.3, $[M+4H]^{4+}$ m/z calcd. (av.) 408.49, obs. 408.4.

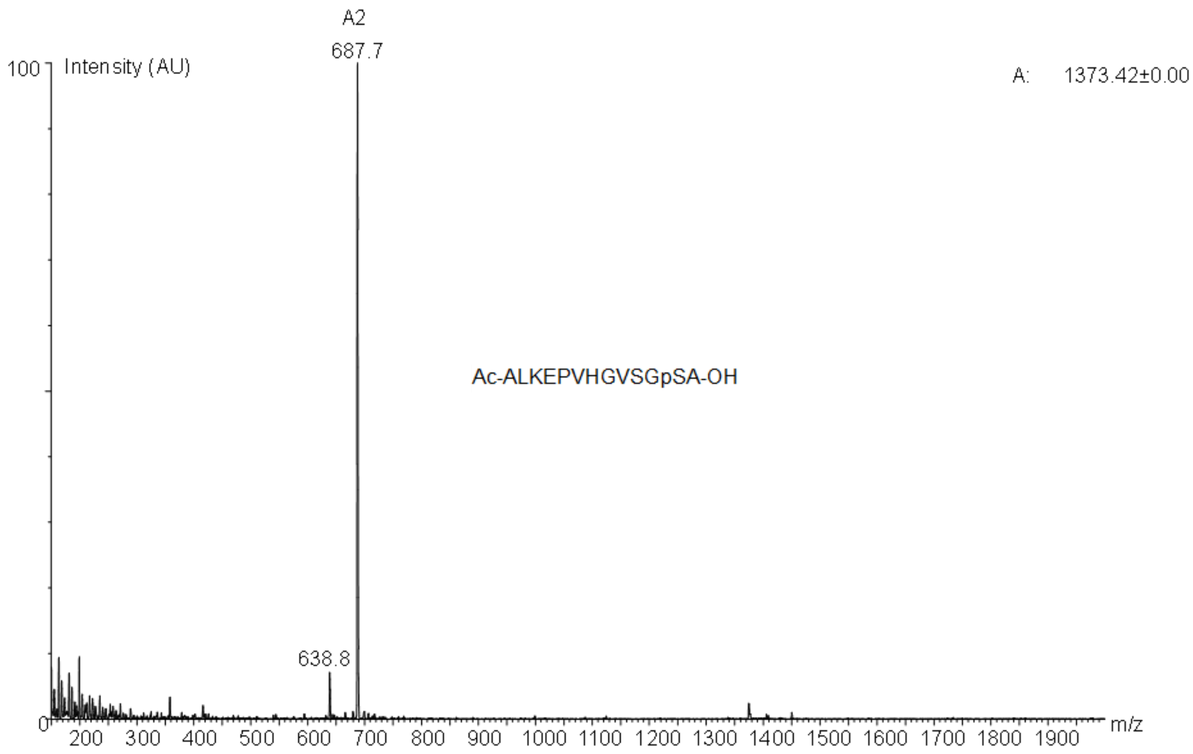

Supplementary Figure 129. MS trace of peak at Rt = 12.65 min from LC-MS analysis of the reaction of peptide thioester Ac-ALKEPVHGVSGpSA-MPAA **1b** with peptide GRRRRALKEPVHGV-NH_2 **2c** after 20 h. Peptide thioester hydrolysis byproduct Ac-ALKEPVHGVSGpSA-OH. $[M+2H]^{2+}$ m/z calcd. (av.) 687.71, obs. 687.7.

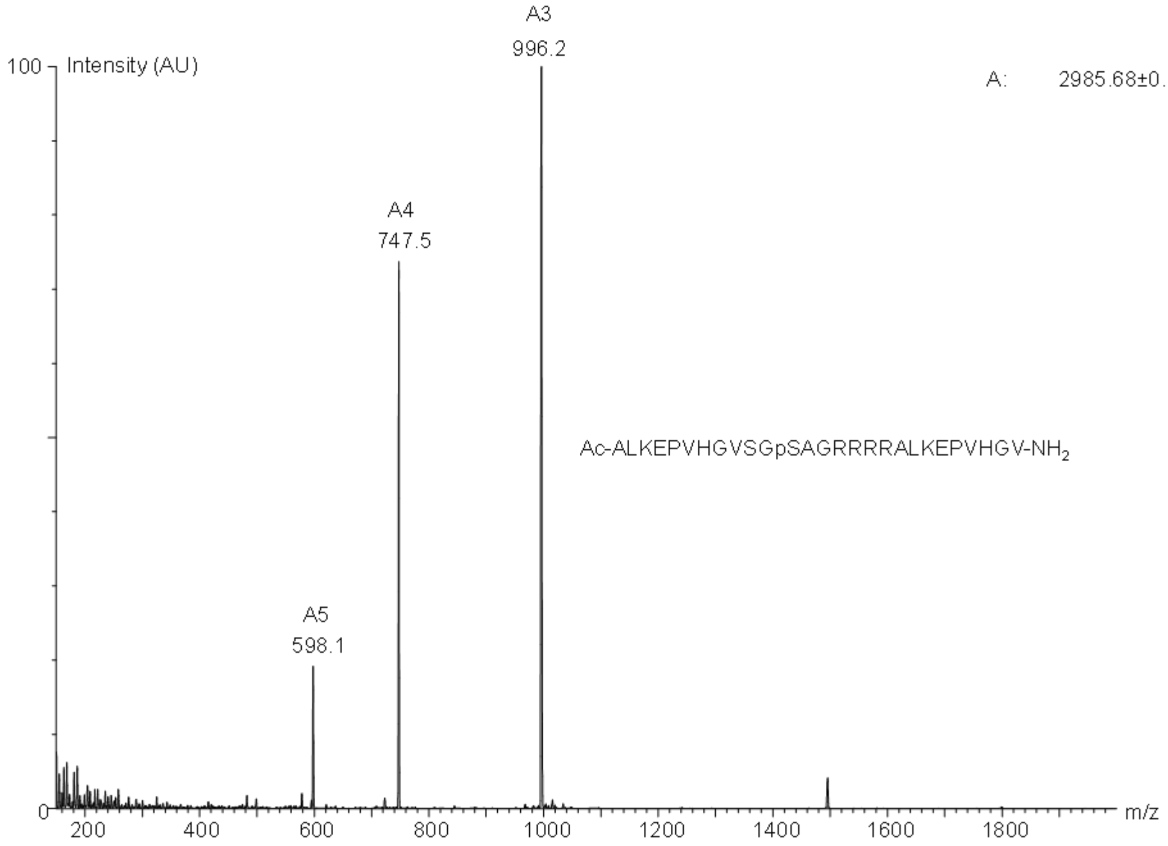

Supplementary Figure 130. MS trace of peak at Rt = 13.69 min from LC-MS analysis of the reaction of peptide thioester Ac-ALKEPVHGVSGpSA-MPAA **1b** with peptide GRRRRALKEPVHGV-NH_2 **2c** after 20 h. Target ligation product Ac-ALKEPVHGVSGpSAGRRRRALKEPVHGV-NH_2 **3b,c**. $[M+3H]^{3+}$ m/z calcd. (av.) 996.12, obs. 996.2, $[M+4H]^{4+}$ m/z calcd. (av.) 747.34, obs. 747.5, $[M+5H]^{5+}$ m/z calcd. (av.) 598.07, obs. 598.1.

1b + 2d → 3b,d:

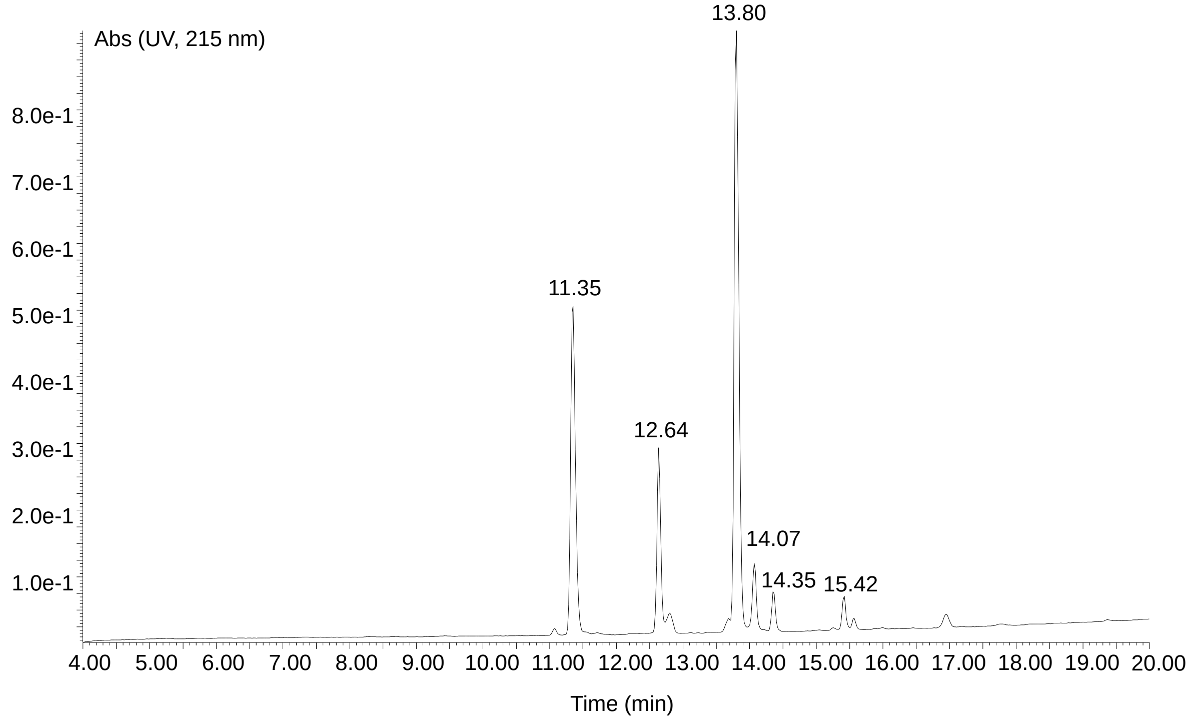

Supplementary Figure 131. LC-MS analysis of the reaction of peptide thioester Ac-ALKEPVHGVSGpSA-MPAA **1b** with peptide GRRRALKEPVHGV-NH_2 **2d** after 20 h. LC trace. Eluent A 0.1% TFA in water, eluent B 0.1% TFA in CH_3CN. C18 X bridge BEH 300 Å (5 µm, 4.6 × 250 mm) column, gradient 0-50% B in 30 min, 1 mL min^{-1}, detection at 215 nm).

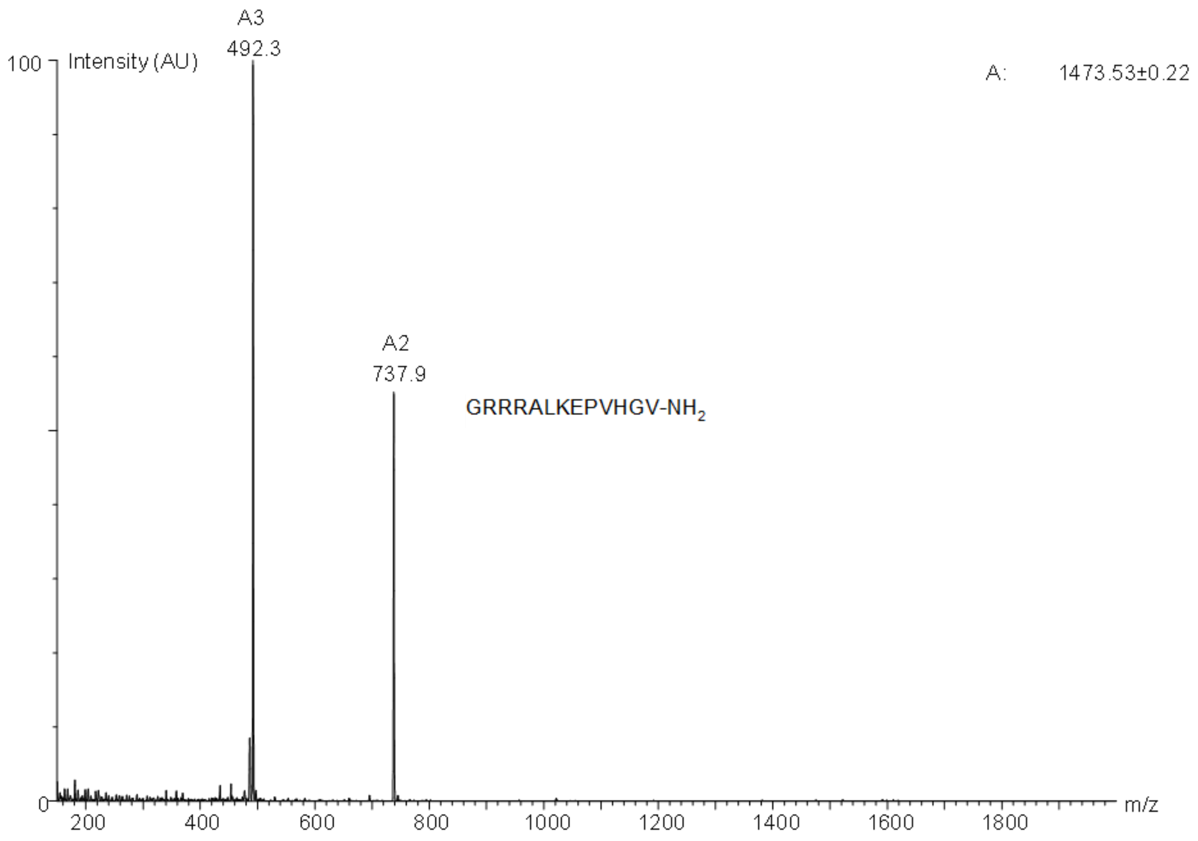

Supplementary Figure 132. MS trace of peak at Rt = 11.35 min from LC-MS analysis of the reaction of peptide thioester Ac-ALKEPVHGVSGpSA-MPAA **1b** with peptide GRRRALKEPVHGV-NH_2 **2d** after 20 h. GRRRALKEPVHGV-NH_2 **2d**. $[M+2H]^{2+}$ m/z calcd. (av.) 737.88, obs. 737.9, $[M+3H]^{3+}$ m/z calcd. (av.) 492.25, obs. 492.3.

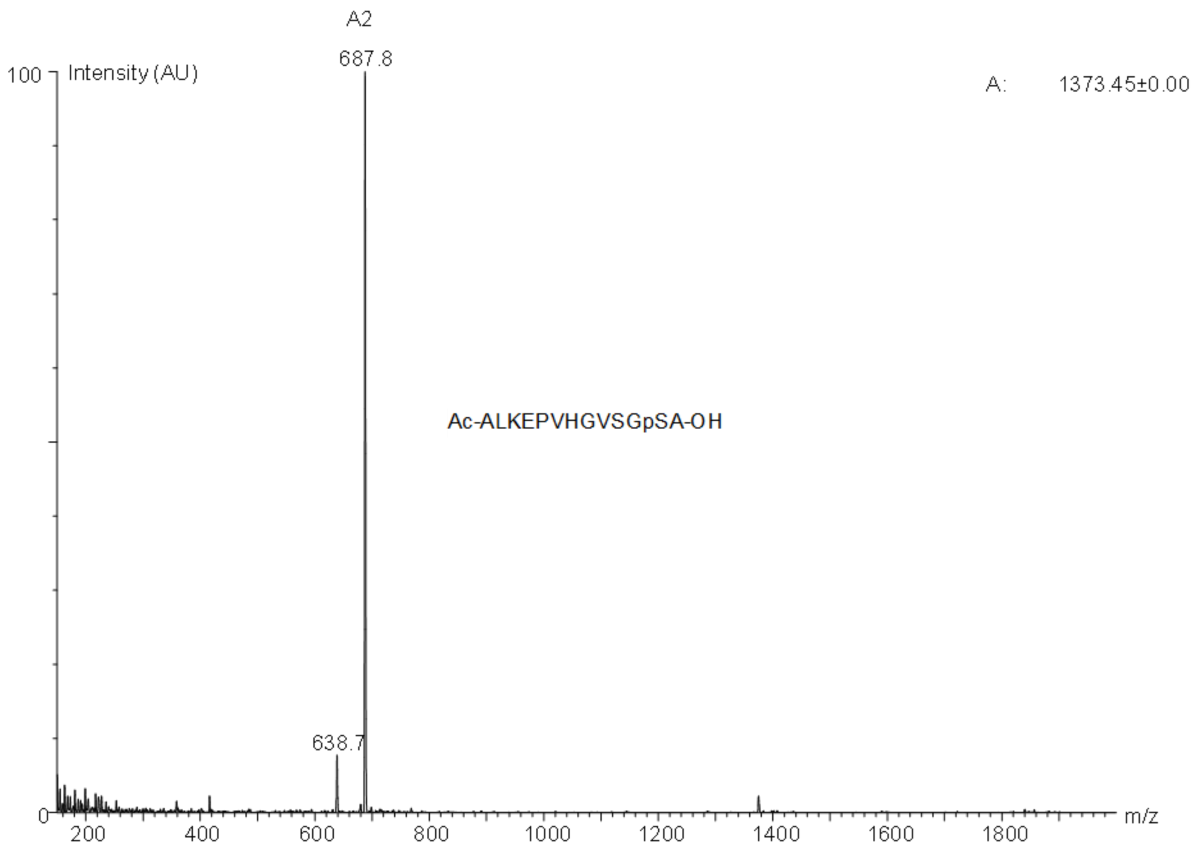

Supplementary Figure 133. MS trace of peak at Rt = 12.64 min from LC-MS analysis of the reaction of peptide thioester Ac-ALKEPVHGVSGpSA-MPAA **1b** with peptide GRRRALKEPVHGV-NH_2 **2d** after 20 h. Peptide thioester hydrolysis byproduct Ac-ALKEPVHGVSGpSA-OH. $[M+2H]^{2+}$ m/z calcd. (av.) 687.71, obs. 687.8.

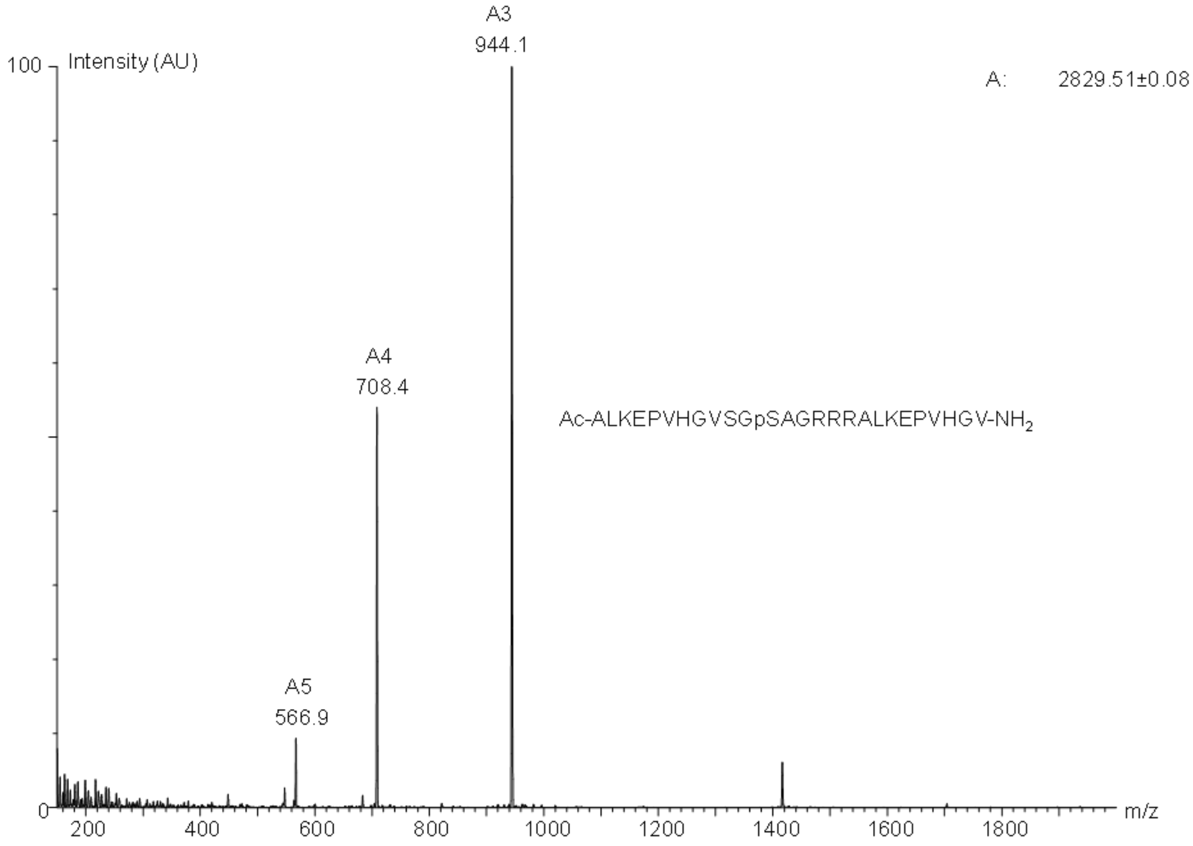

Supplementary Figure 134. MS trace of peak at Rt = 13.80 min from LC-MS analysis of the reaction of peptide thioester Ac-ALKEPVHGVSGpSA-MPAA **1b** with peptide GRRRALKEPVHGV-NH_2 **2d** after 20 h. Target ligation product Ac-ALKEPVHGVSGpSAGRRRALKEPVHGV-NH_2 **3b,d**. $[M+3H]^{3+}$ m/z calcd. (av.) 944.05, obs. 944.1, $[M+4H]^{4+}$ m/z calcd. (av.) 708.29, obs. 708.4, $[M+5H]^{5+}$ m/z calcd. (av.) 566.83, obs. 566.9.

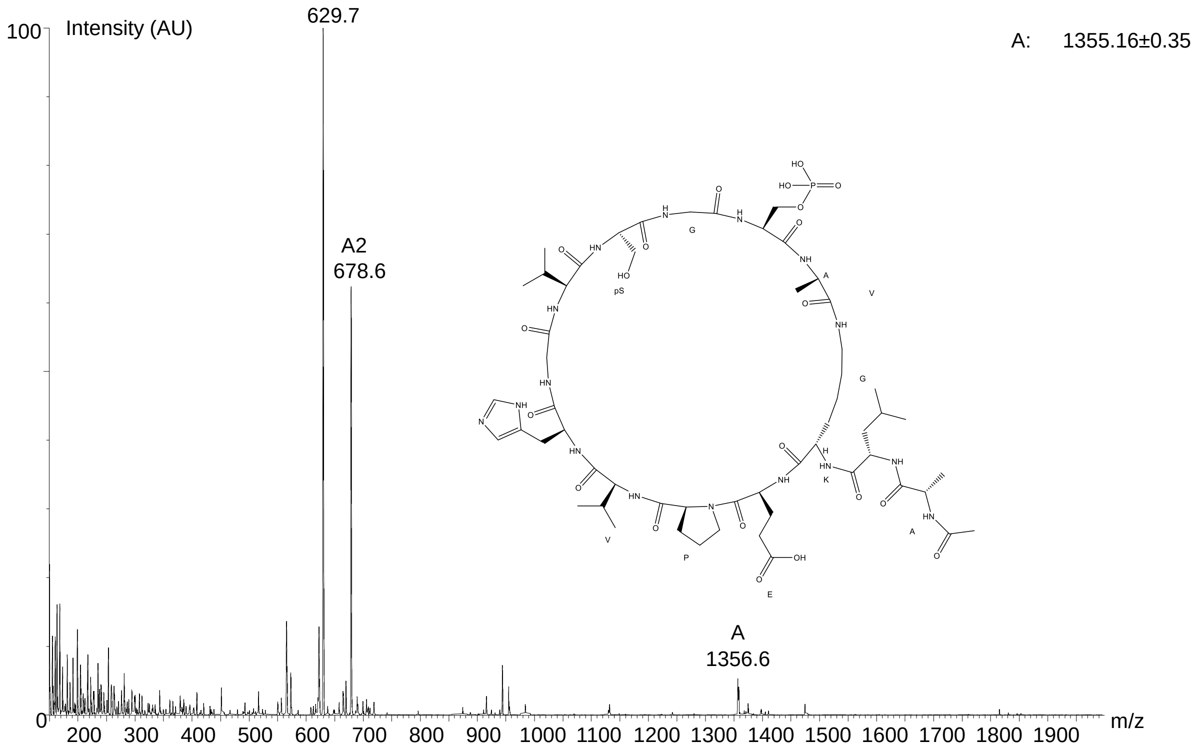

Supplementary Figure 135. MS trace of peak at Rt = 14.07 min from LC-MS analysis of the reaction of peptide thioester Ac-ALKEPVHGVSGpSA-MPAA **1b** with peptide GRRRALKEPVHGV-NH_2 **2d** after 20 h. Peptide thioester cyclized byproduct. $[M+H]^+$ m/z calcd. (av.) 1356.41, obs. 1356.6, $[M+2H]^{2+}$ m/z calcd. (av.) 678.70, obs. 678.6.

1b + 2e → 3b,e:

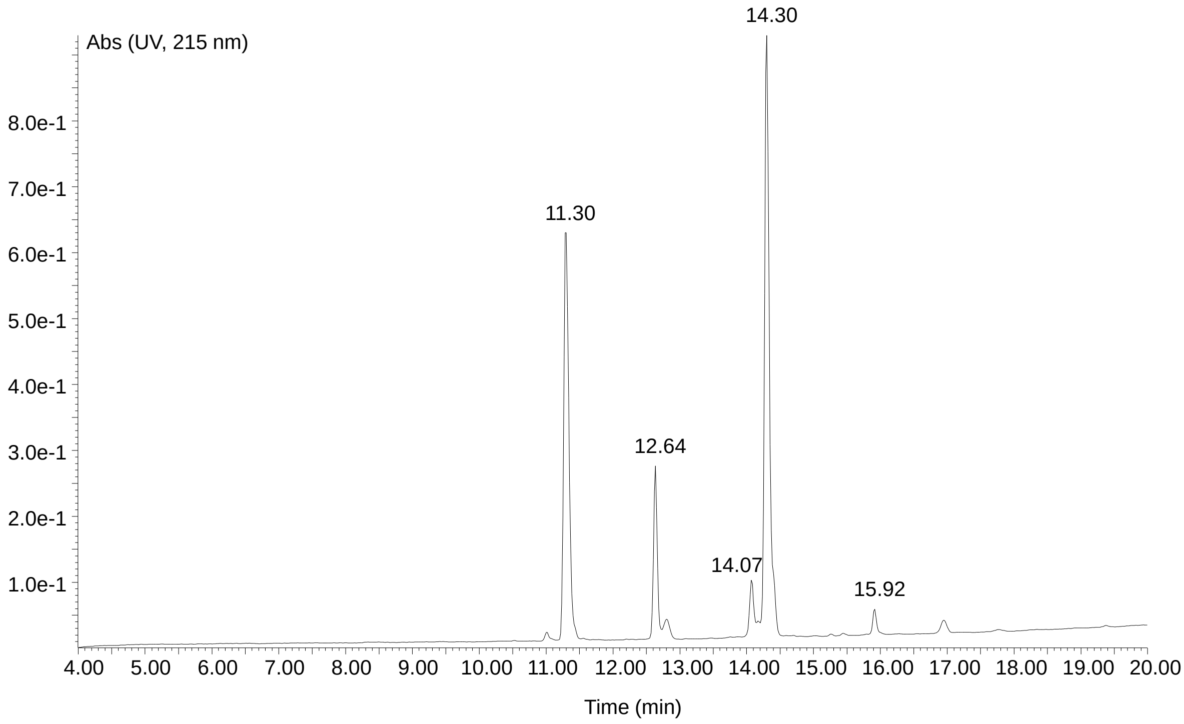

Supplementary Figure 136. LC-MS analysis of the reaction of peptide thioester Ac-ALKEPVHGVSGpSA-MPAA **1b** with peptide GRRALKEPVHGV-NH_2 **2e** after 20 h. LC trace. Eluent A 0.1% TFA in water, eluent B 0.1% TFA in CH_3CN. C18 X bridge BEH 300 Å (5 µm, 4.6 × 250 mm) column, gradient 0-50% B in 30 min, 1 mL min^{-1}, detection at 215 nm).

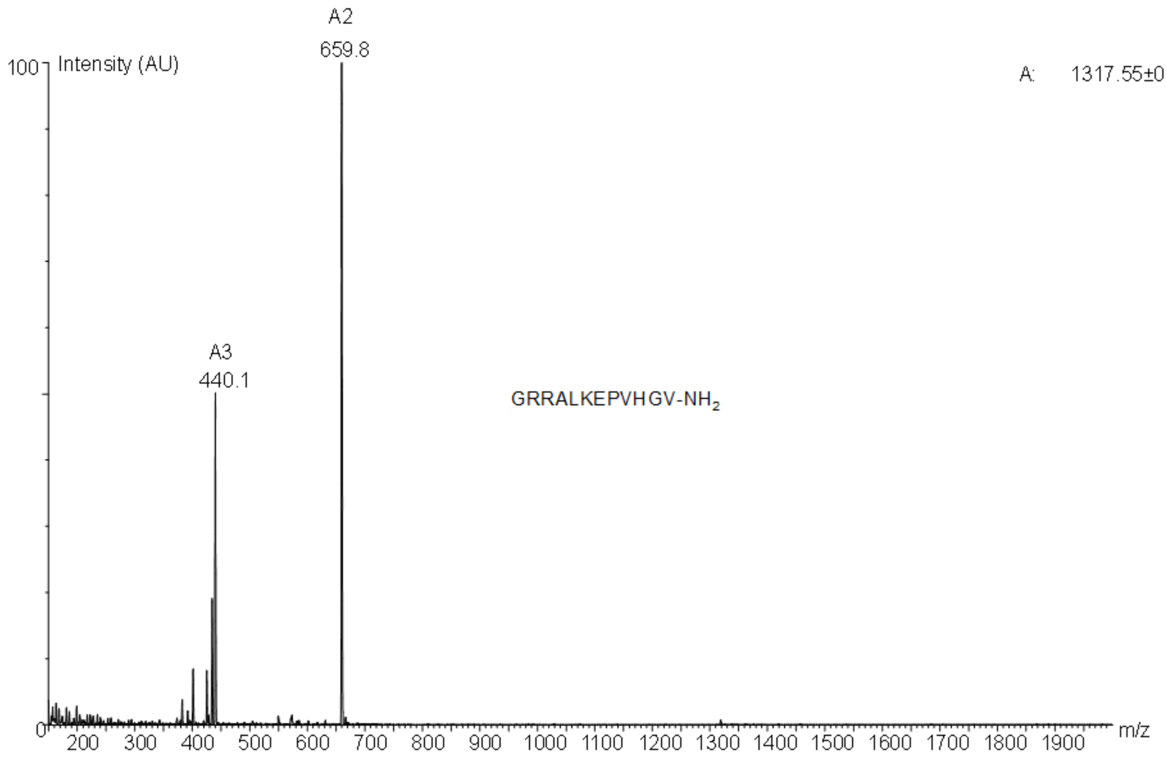

Supplementary Figure 137. MS trace of peak at Rt = 11.30 min from LC-MS analysis of the reaction of peptide thioester Ac-ALKEPVHGVSGpSA-MPAA **1b** with peptide GRRALKEPVHGV-NH_2 **2e** after 20 h. GRRALKEPVHGV-NH_2 **2e**. $[M+2H]^{2+}$ m/z calcd. (av.) 659.78, obs. 659.8, $[M+3H]^{3+}$ m/z calcd. (av.) 440.19, obs. 440.1.

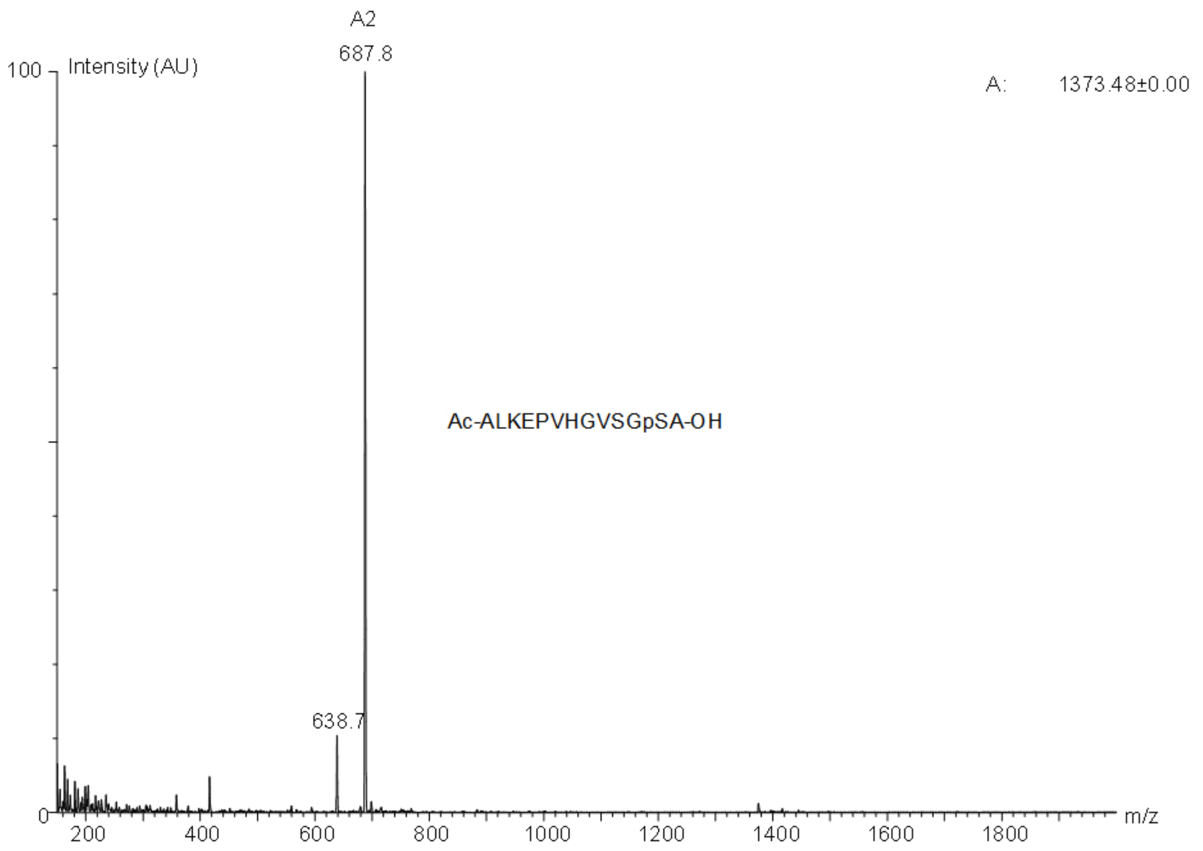

Supplementary Figure 138. MS trace of peak at Rt = 12.64 min from LC-MS analysis of the reaction of peptide thioester Ac-ALKEPVHGVSGpSA-MPAA **1b** with peptide GRRALKEPVHGV-NH_2 **2e** after 20 h. Peptide thioester hydrolysis byproduct Ac-ALKEPVHGVSGpSA-OH. MS trace, $[M+2H]^{2+}$ m/z calcd. (av.) 687.71, obs. 687.8.

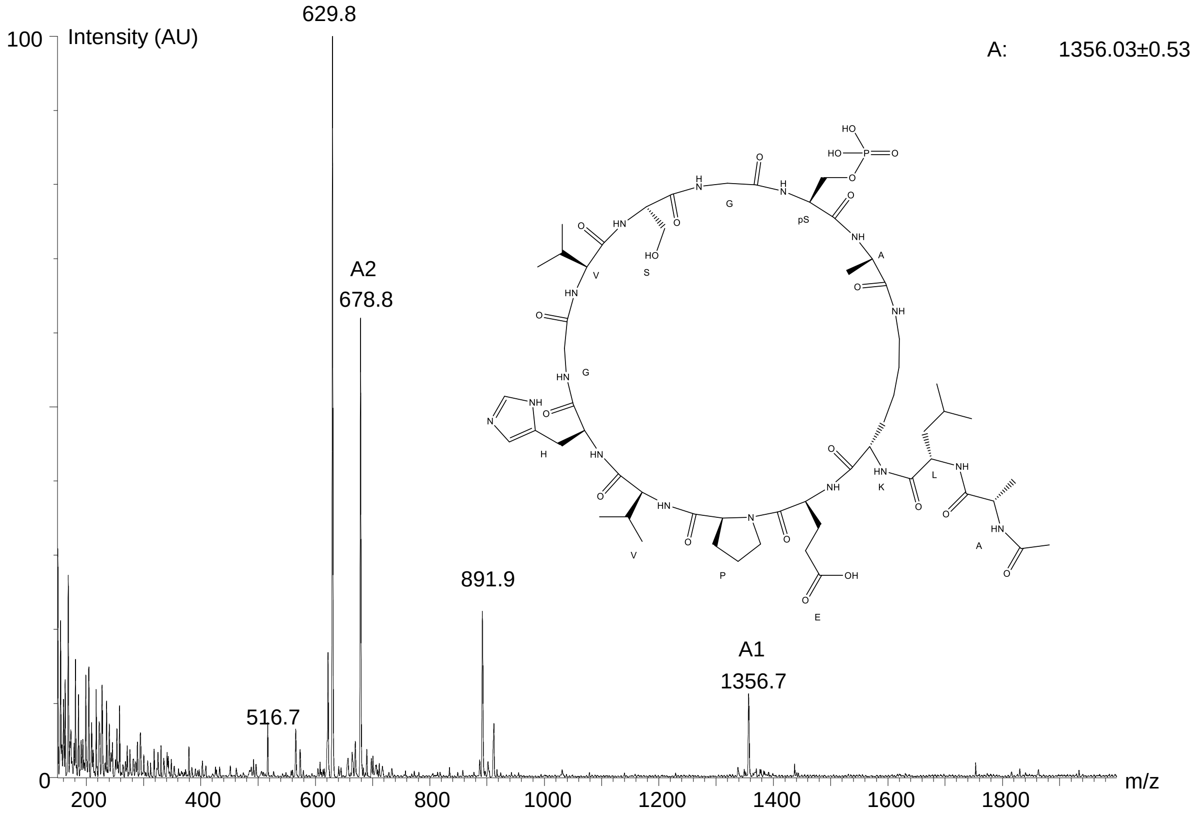

Supplementary Figure 139. MS trace of peak at Rt = 14.07 min from LC-MS analysis of the reaction of peptide thioester Ac-ALKEPVHGVSGpSA-MPAA **1b** with peptide GRRALKEPVHGV-NH_2 **2e** after 20 h. Peptide thioester cyclized byproduct. $[M+H]^+$ m/z calcd. (av.) 1356.41, obs. 1356.7, $[M+2H]^{2+}$ m/z calcd. (av.) 678.70, obs. 678.8

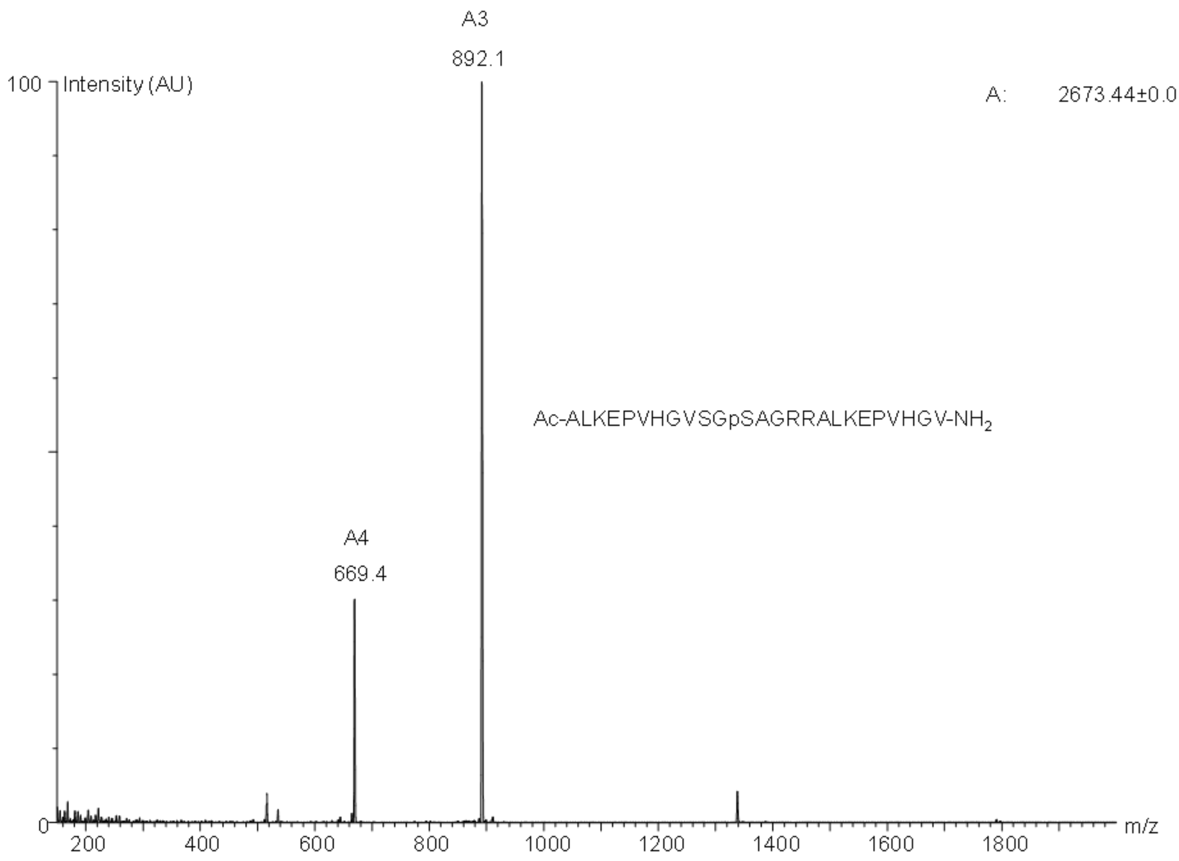

Supplementary Figure 140. MS trace of peak at Rt = 14.30 min from LC-MS analysis of the reaction of peptide thioester Ac-ALKEPVHGVSGpSA-MPAA **1b** with peptide GRRALKEPVHGV-NH_2 **2e** after 20 h. Target ligation product Ac-ALKEPVHGVSGpSAGRRALKEPVHGV-NH_2 **3b,e**. $[M+3H]^{3+}$ m/z calcd. (av.) 891.99, obs. 892.1, $[M+4H]^{4+}$ m/z calcd. (av.) 669.24, obs. 669.4.

1b + 2f → 3b,f:

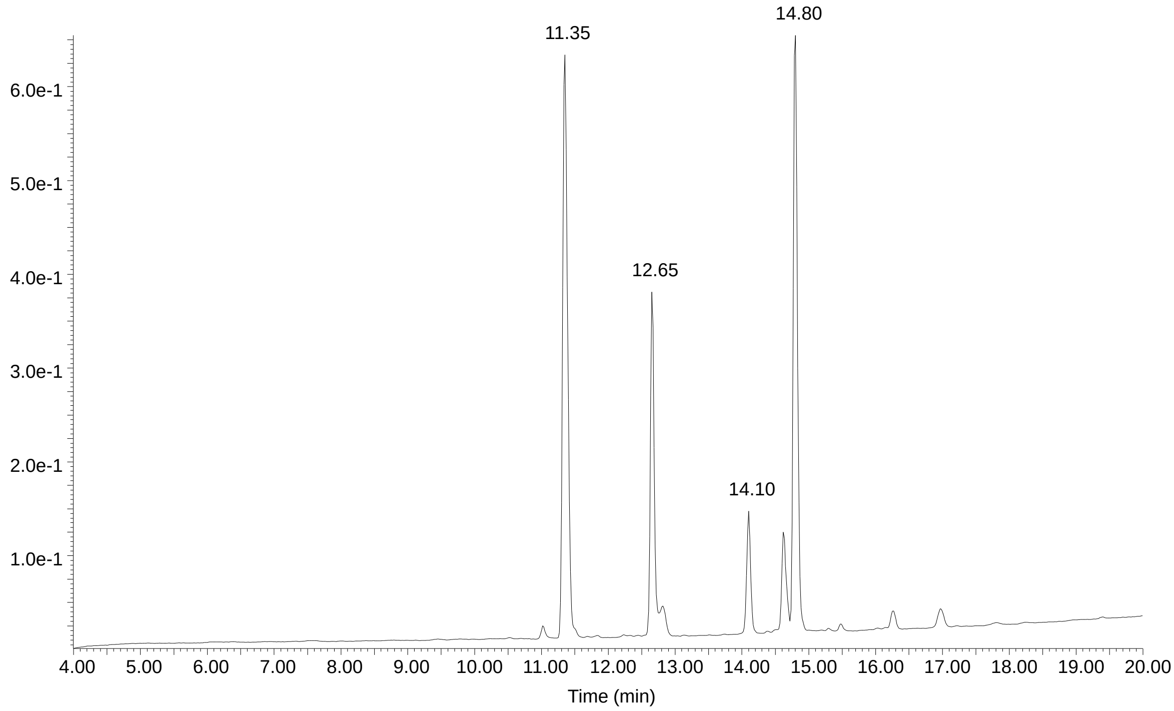

Supplementary Figure 141. LC-MS analysis of the reaction of peptide thioester Ac-ALKEPVHGVSGpSA-MPAA **1b** with peptide GRALKEPVHGV-NH_2 **2f** after 20 h. LC trace. Eluent A 0.1% TFA in water, eluent B 0.1% TFA in CH_3CN. C18 X bridge BEH 300 Å (5 µm, 4.6 × 250 mm) column, gradient 0-50% B in 30 min, 1 mL min^{-1}, detection at 215 nm).

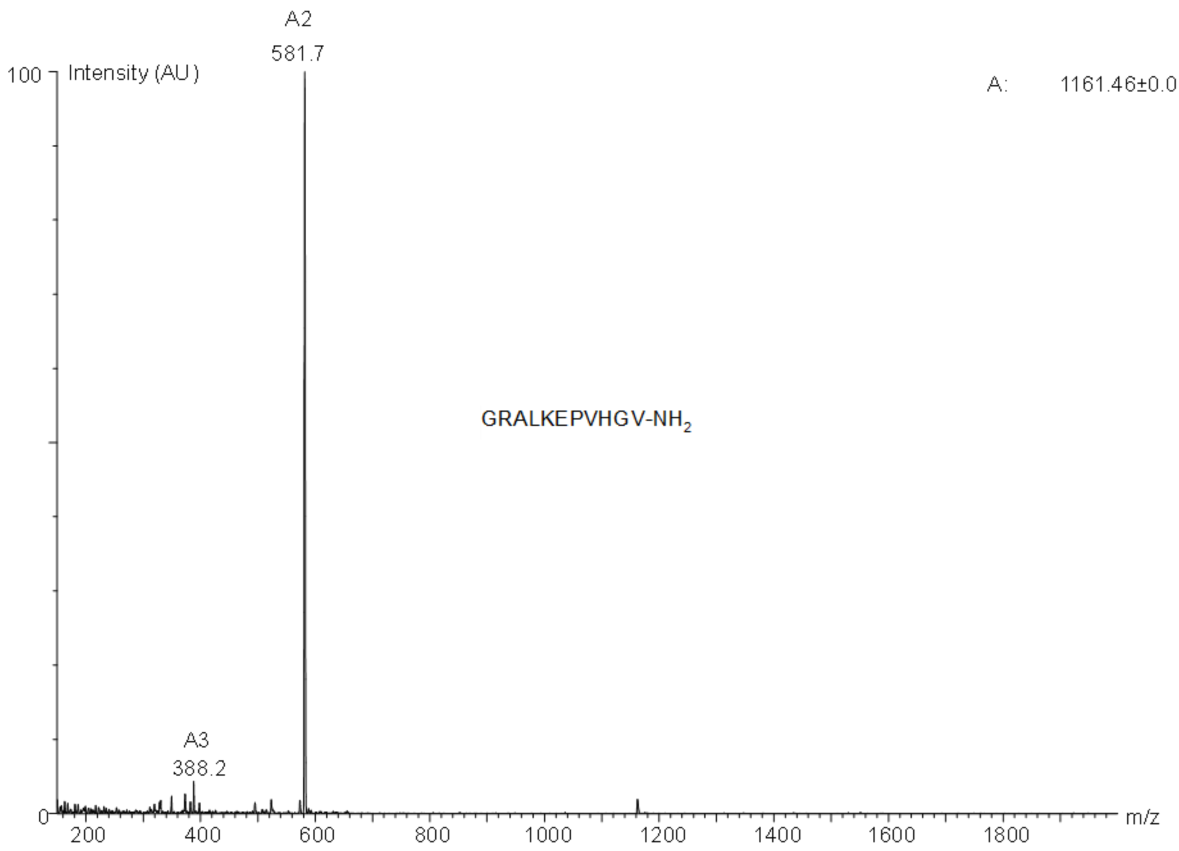

Supplementary Figure 142. MS trace of peak at Rt = 11.35 min from LC-MS analysis of the reaction of peptide thioester Ac-ALKEPVHGVSGpSA-MPAA **1b** with peptide GRALKEPVHGV-NH_2 **2f** after 20 h. GRALKEPVHGV-NH_2 **2f**. $[M+2H]^{2+}$ m/z calcd. (av.) 581.69, obs. 581.7, $[M+3H]^{3+}$ m/z calcd. (av.) 388.13, obs. 388.2.

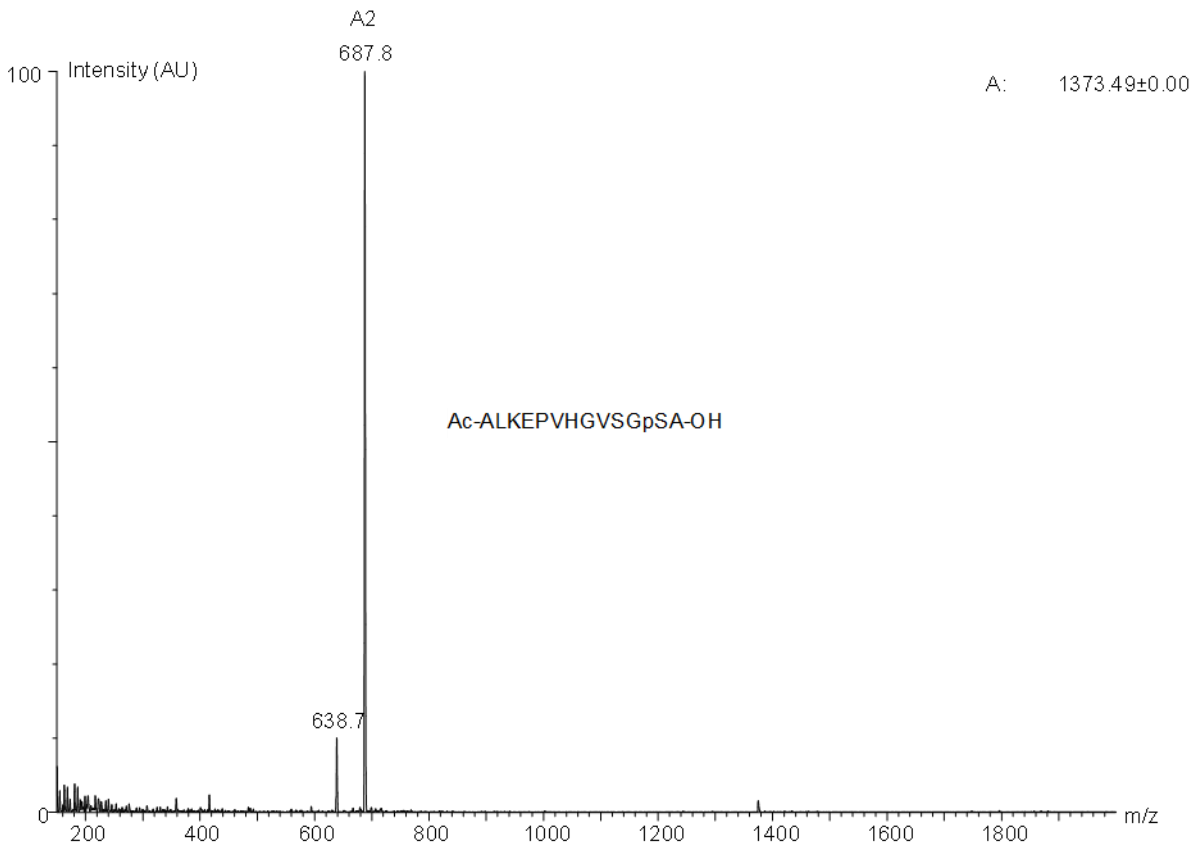

Supplementary Figure 143. MS trace of peak at Rt = 12.65 min from LC-MS analysis of the reaction of peptide thioester Ac-ALKEPVHGVSGpSA-MPAA **1b** with peptide GRALKEPVHGV-NH_2 **2f** after 20 h. Peptide thioester hydrolysis byproduct Ac-ALKEPVHGVSGpSA-OH. $[M+2H]^{2+}$ m/z calcd. (av.) 687.71, obs. 687.8.

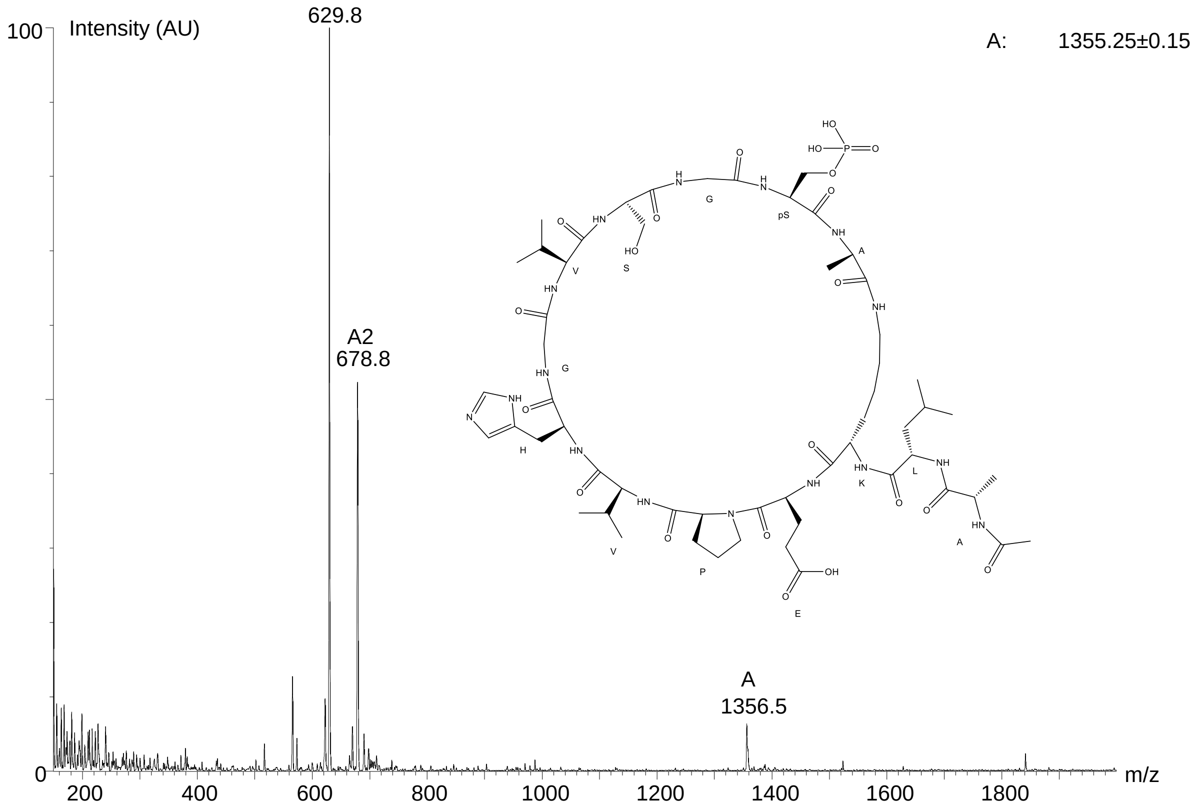

Supplementary Figure 144. MS trace of peak at Rt = 14.10 min from LC-MS analysis of the reaction of peptide thioester Ac-ALKEPVHGVSGpSA-MPAA **1b** with peptide GRALKEPVHGV-NH_2 **2f** after 20 h. Peptide thioester cyclized byproduct. $[M+H]^+$ m/z calcd. (av.) 1356.41, obs. 1356.5, $[M+2H]^{2+}$ m/z calcd. (av.) 678.70, obs. 678.8.

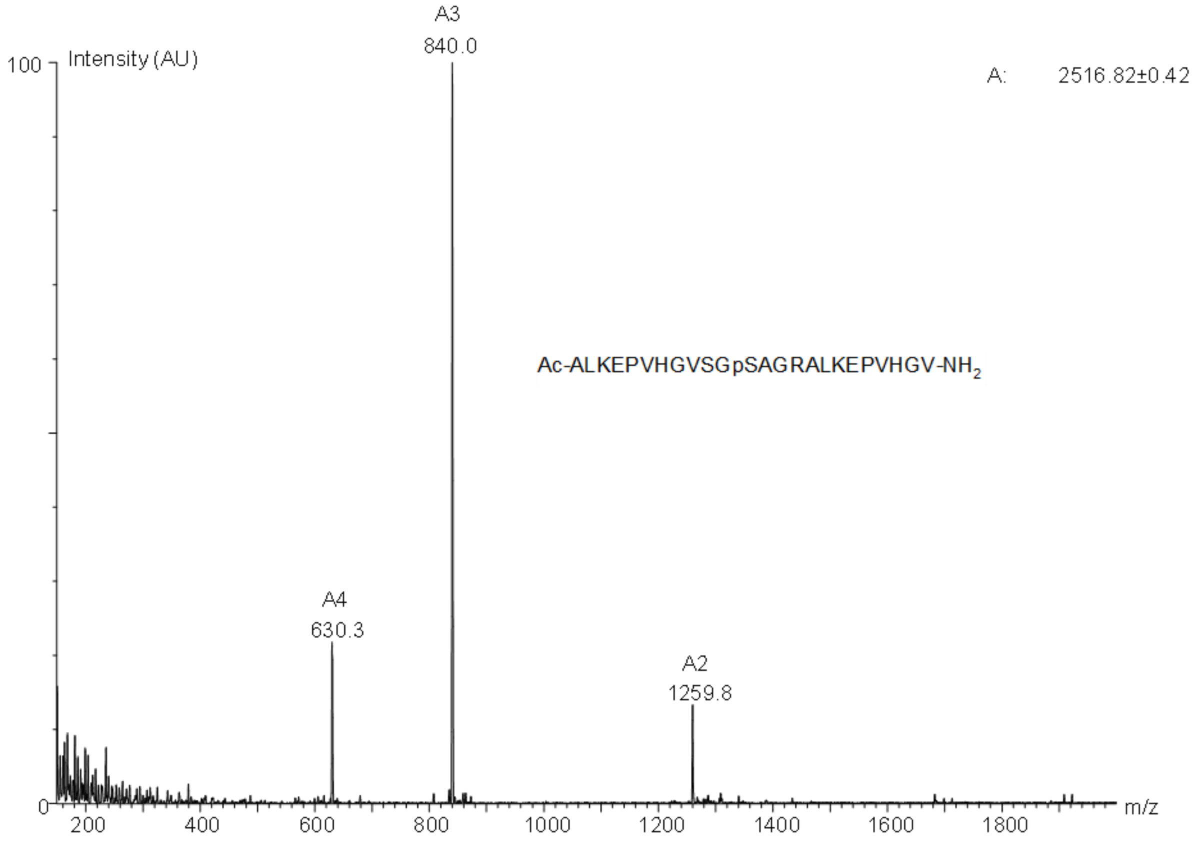

Supplementary Figure 145. MS trace of peak at Rt = 14.60 min from LC-MS analysis of the reaction of peptide thioester Ac-ALKEPVHGVSGpSA-MPAA **1b** with peptide GRALKEPVHGV-NH_2 **2f** after 20 h. Target ligation product Ac-ALKEPVHGVSGpSAGRALKEPVHGV-NH_2 **3b,f**. $[M+2H]^{2+}$ m/z calcd. (av.) 1259.39, obs. 1259.8, $[M+3H]^{3+}$ m/z calcd. (av.) 839.93, obs. 840.0, $[M+4H]^{4+}$ m/z calcd. (av.) 630.20, obs. 630.3.

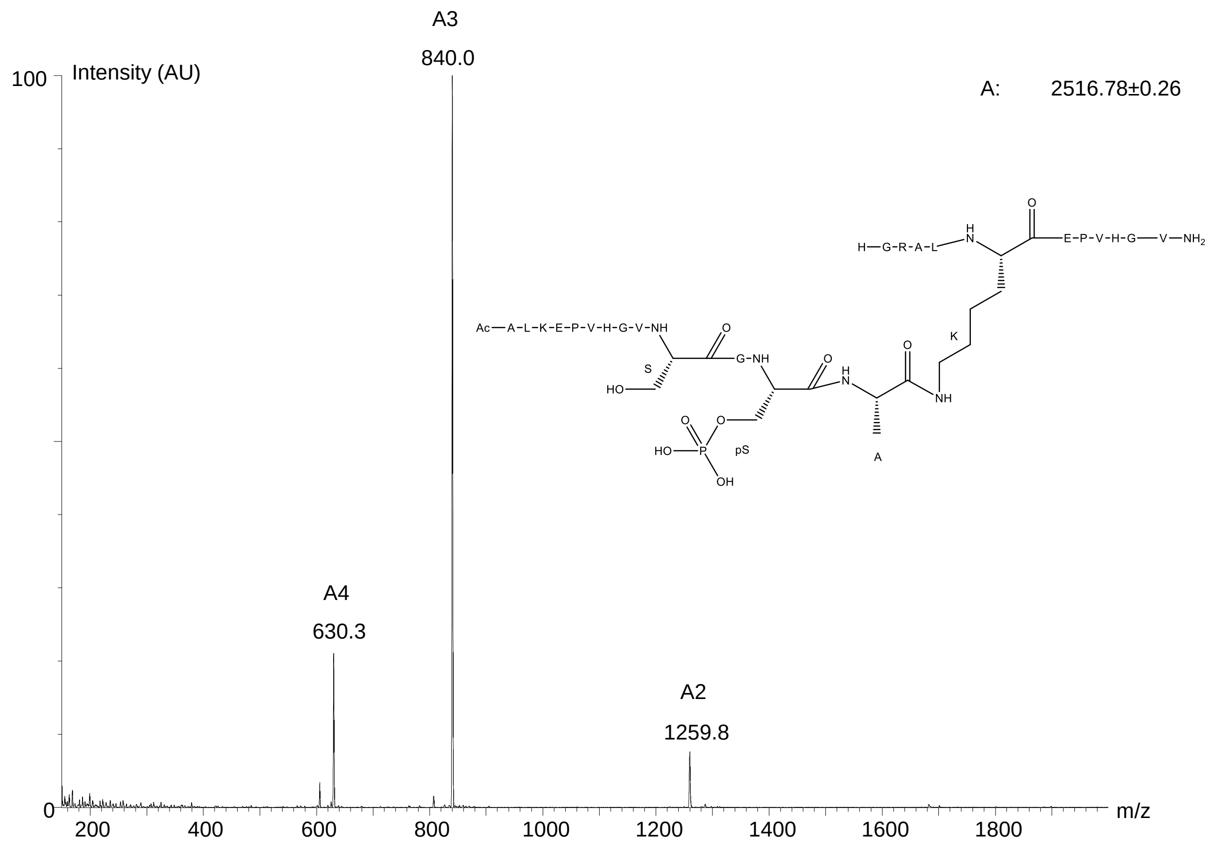

Supplementary Figure 146. MS trace of peak at Rt = 14.80 min from LC-MS analysis of the reaction of peptide thioester Ac-ALKEPVHGVSGpSA-MPAA **1b** with peptide GRALKEPVHGV-NH_2 **2f** after 20 h. Branched byproduct GRALK(Ac-ALKEPVHGVSGpSA)EPVHGV-NH_2. $[M+2H]^{2+}$ m/z calcd. (av.) 1259.39, obs. 1259.8, $[M+3H]^{3+}$ m/z calcd. (av.) 839.93, obs. 840.0, $[M+4H]^{4+}$ m/z calcd. (av.) 630.20, obs. 630.3.

1b + 2g → 3b,g:

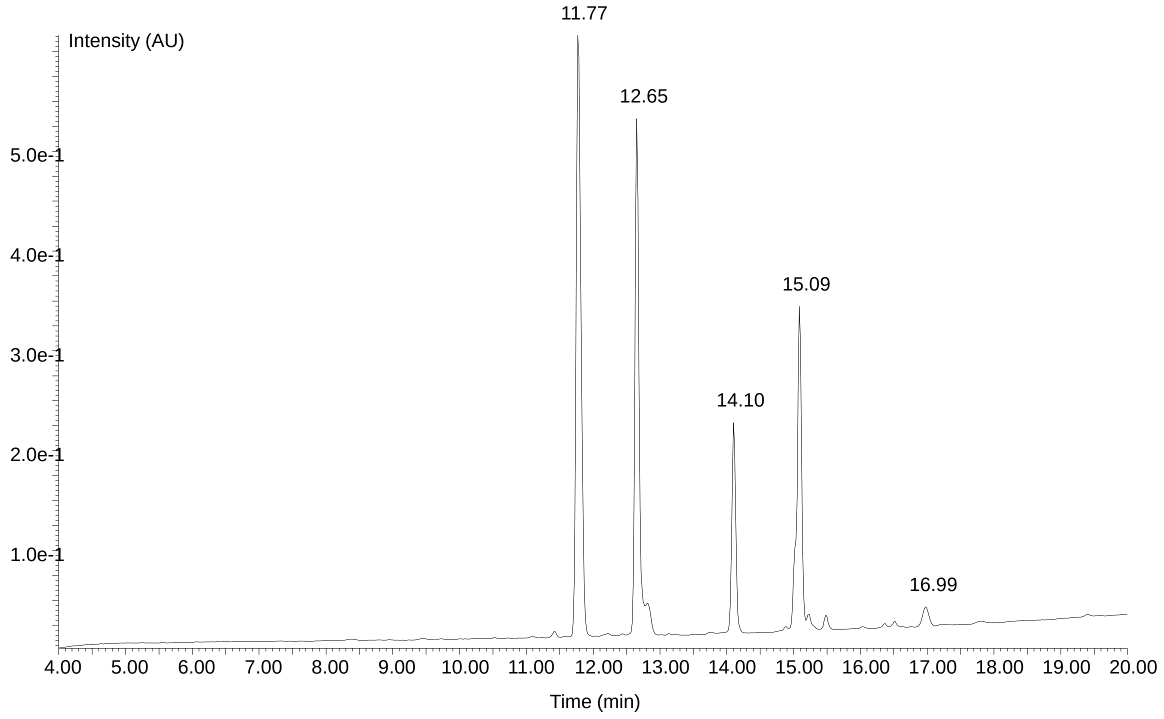

Supplementary Figure 147. LC-MS analysis of the reaction of peptide thioester Ac-ALKEPVHGVSGpSA-MPAA **1b** with peptide GALKEPVHGV-NH_2 **2g** after 20 h. Eluent A 0.1% TFA in water, eluent B 0.1% TFA in CH_3CN. C18 X bridge BEH 300 Å (5 µm, 4.6 × 250 mm) column, gradient 0-50% B in 30 min, 1 mL min^{-1}, detection at 215 nm).

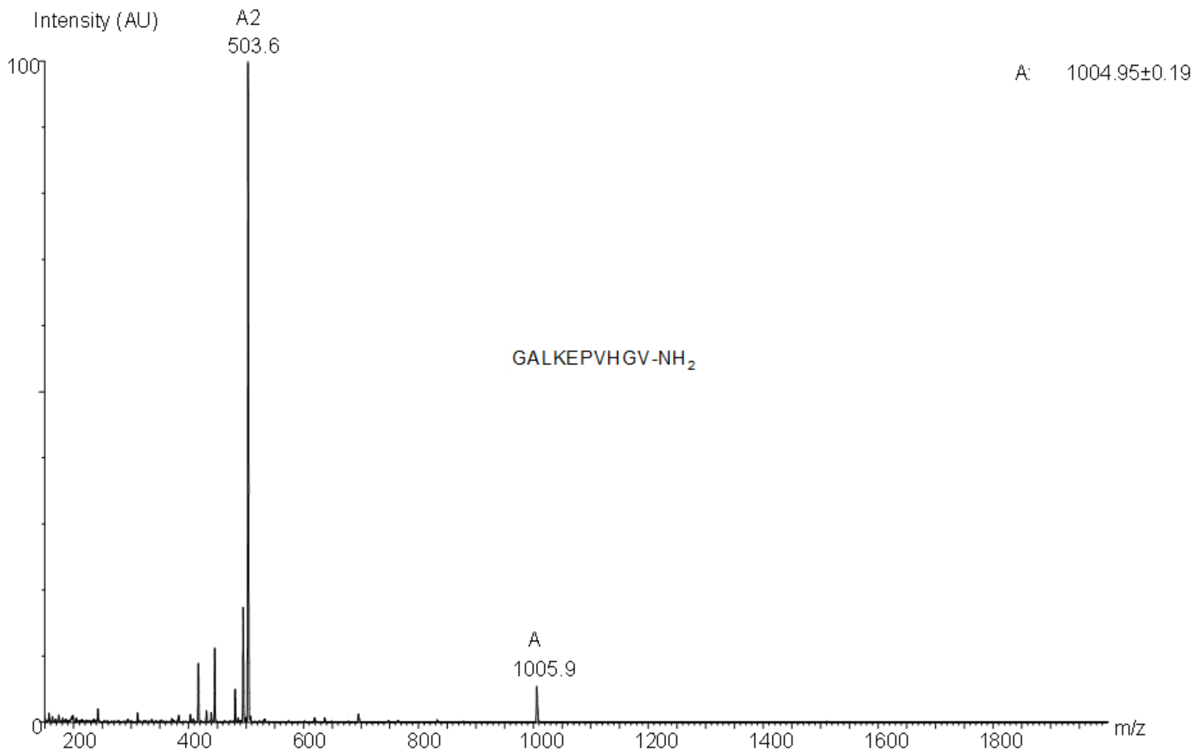

Supplementary Figure 148. MS trace of peak at Rt = 11.77 min from LC-MS analysis of the reaction of peptide thioester Ac-ALKEPVHGVSGpSA-MPAA **1b** with peptide GALKEPVHGV-NH_2 **2g** after 20 h. GALKEPVHGV-NH_2 **2g**. $[M+H]^+$ m/z calcd. (monoisotopic) 1005.58, obs. 1005.9, $[M+2H]^{2+}$ m/z calcd. (av.) 503.59, obs. 503.6.

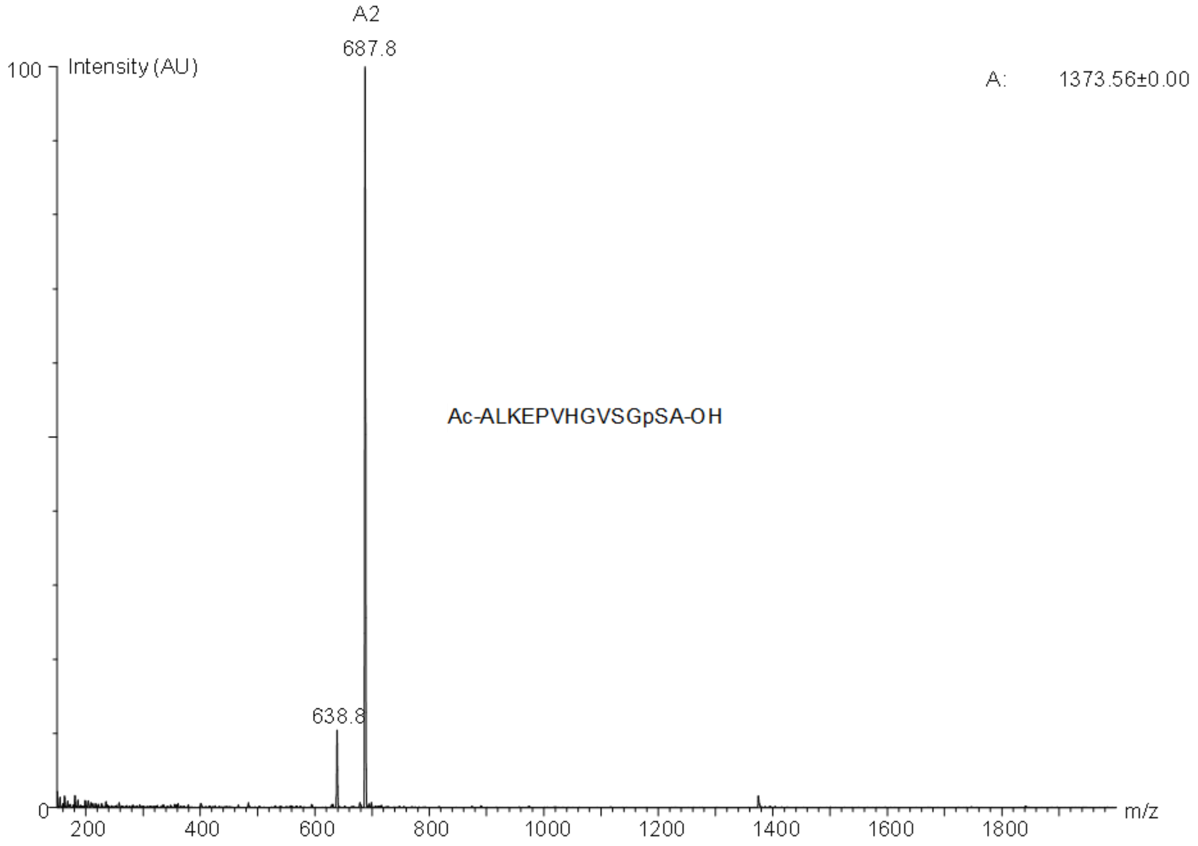

Supplementary Figure 149. MS trace of peak at Rt = 12.65 min from LC-MS analysis of the reaction of peptide thioester Ac-ALKEPVHGVSGpSA-MPAA **1b** with peptide GALKEPVHGV-NH_2 **2g** after 20 h. Peptide hydrolysis byproduct Ac-ALKEPVHGVSGpSA-OH. $[M+2H]^{2+}$ m/z calcd. (av.) 687.71, obs. 687.8.

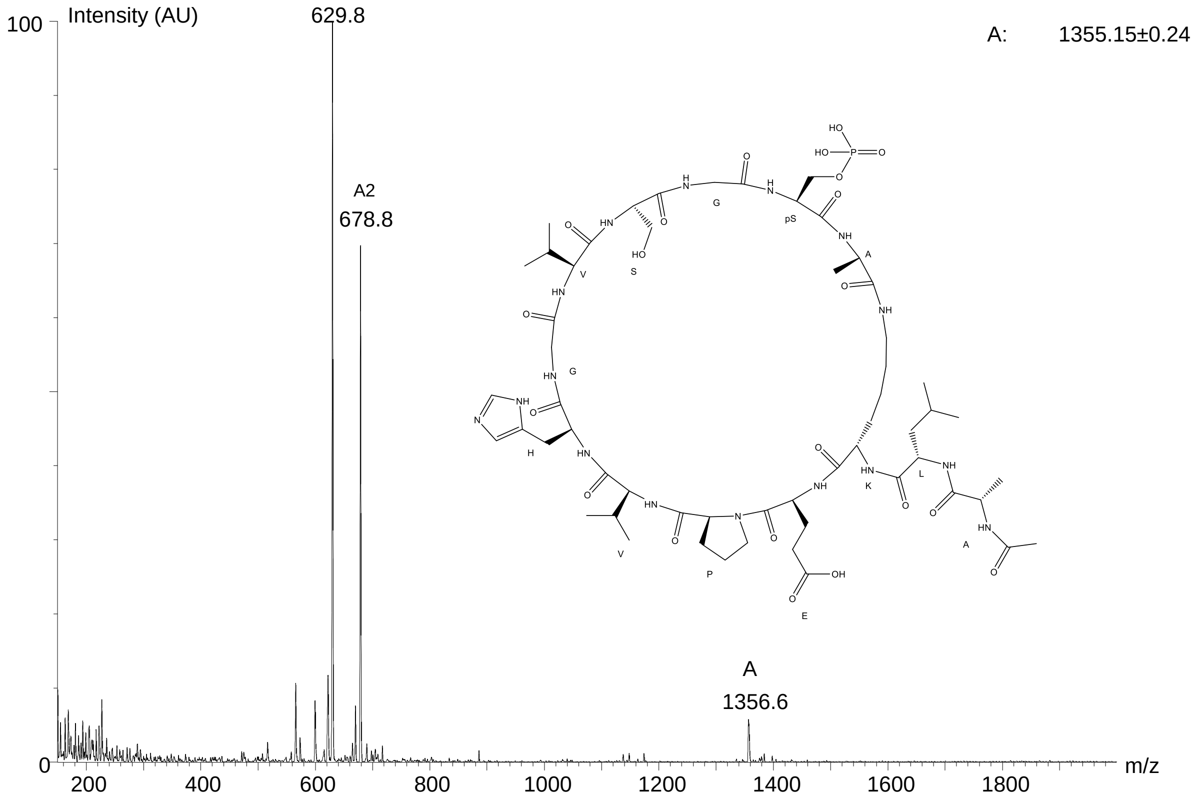

Supplementary Figure 150. MS trace of peak at Rt = 14.10 min from LC-MS analysis of the reaction of peptide thioester Ac-ALKEPVHGVSGpSA-MPAA **1b** with peptide GALKEPVHGV-NH_2 **2g** after 20 h. Peptide thioester cyclized byproduct. $[M+H]^+$ m/z calcd. (av.) 1356.41, obs. 1356.6, $[M+2H]^{2+}$ m/z calcd. (av.) 678.70, obs. 678.8.

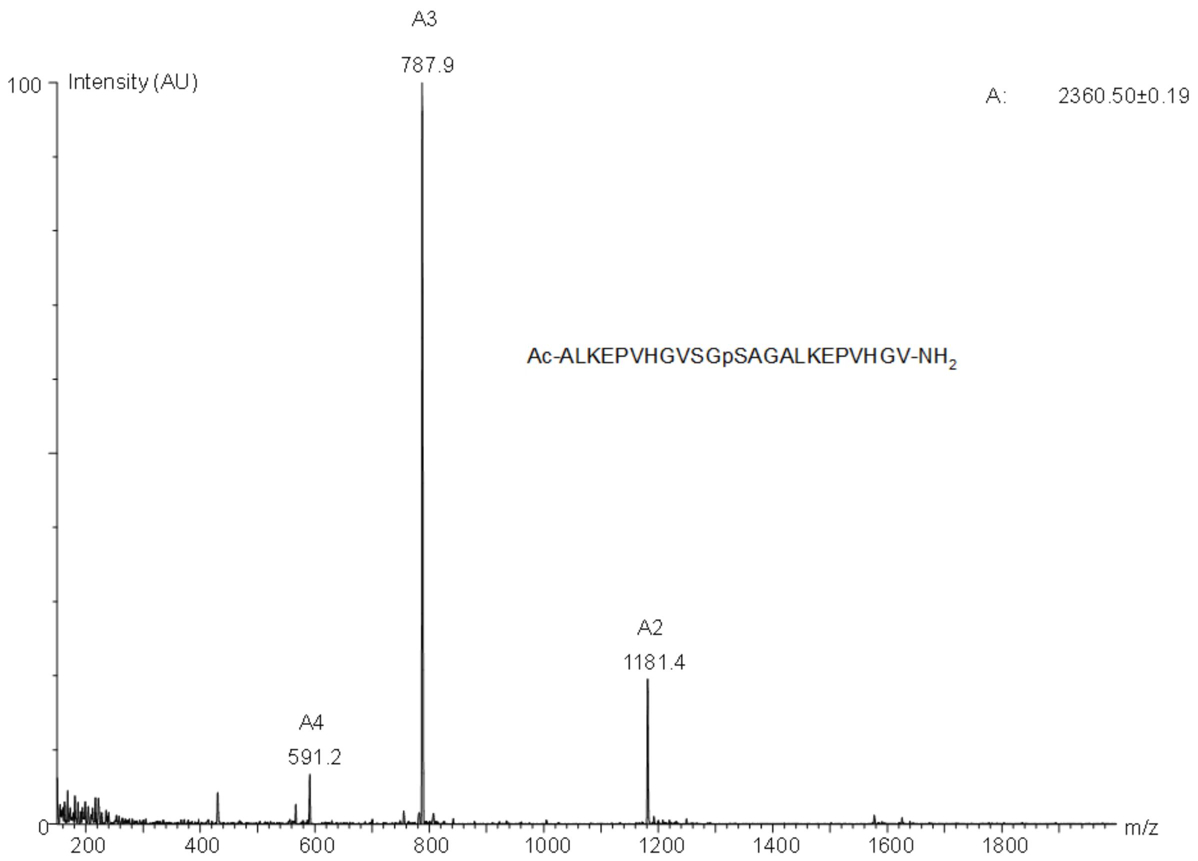

Supplementary Figure 151. MS trace of peak at Rt = 15.09 min from LC-MS analysis of the reaction of peptide thioester Ac-ALKEPVHGVSGpSA-MPAA **1b** with peptide GALKEPVHGV-NH_2 **2g** after 20 h. Target ligation product Ac-ALKEPVHGVSGpSAGALKEPVHGV-NH_2 **3b,g**. $[M+2H]^{2+}$ m/z calcd. (av.) 1181.30, obs. 1181.4, $[M+3H]^{3+}$ m/z calcd. (av.) 787.87, obs. 787.9, $[M+4H]^{4+}$ m/z calcd. (av.) 591.15, obs. 591.2.

Effect on the number and position of pSer residues (Fig. 3d)

GRRRRRRALKEPVHGV-NH_2 peptide **2a** (~1.82 µmol, 5 mM final concentration) was dissolved in sodium bicarbonate/CO_2 buffer (50 mM, 364 µL). The peptide thioesters **1a-d** were weighted in 0.6 mL plastic tubes (1 equiv). Peptide **2a** solution was added to the peptide thioesters **1a-d** (~80 µL, final pH=6.84-7.38) and the plastic tubes were vortexed immediately. The plastic tubes containing the reaction mixtures were then placed in a CO_2 incubator for cell biology (5% partial CO_2 pressure, 37 °C, water saturated) with a needle inserted through the cap to enable gas exchange between the interior of the plastic tube and the incubator.

After 20 h, the reaction mixtures (1 µL) were quenched with aqueous AcOH (10 % AcOH in water, 100 µL) and analyzed by LC-MS.

1a + 2a → 3a,a:

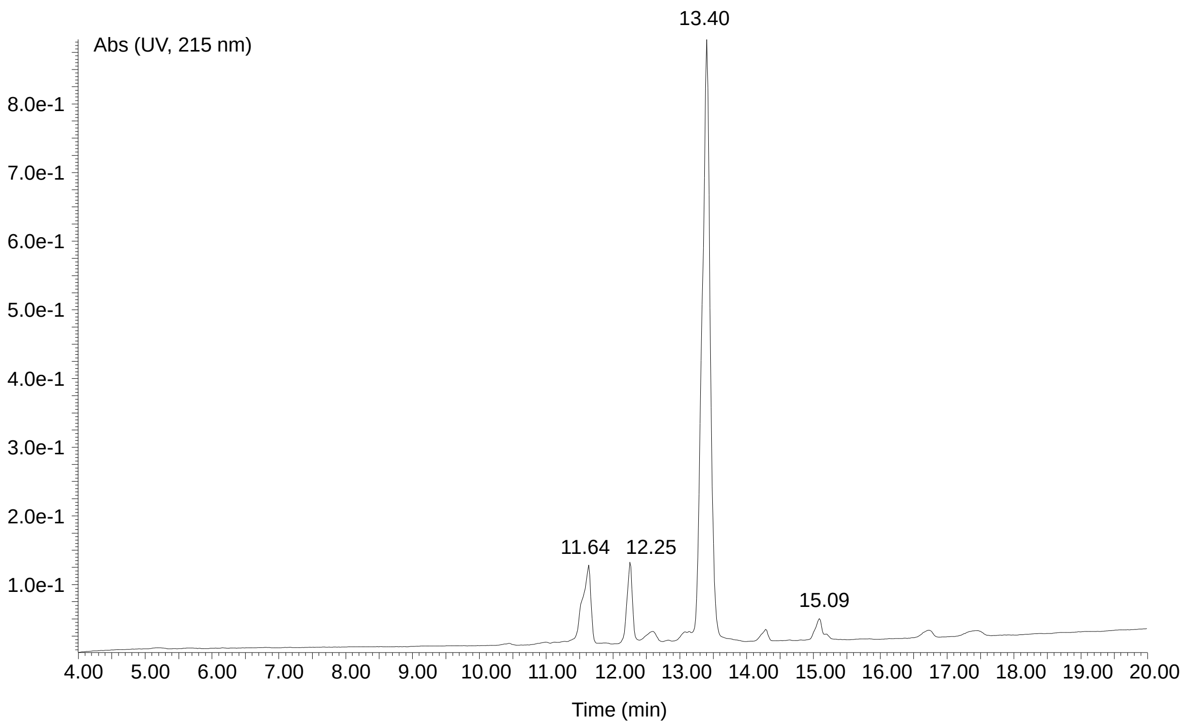

Supplementary Figure 152**.** LC-MS analysis of the reaction of peptide thioester Ac-ALKEPVHGVpSGpSA-MPAA **1a** with peptide GRRRRRRALKEPVHGV-NH_2 **2a** after 20 h. LC trace. Eluent A 0.1% TFA in water, eluent B 0.1% TFA in CH_3CN. C18 X bridge BEH 300 Å (5 µm, 4.6 × 250 mm) column, gradient 0-50% B in 30 min, 1 mL min^{-1}, detection at 215 nm).

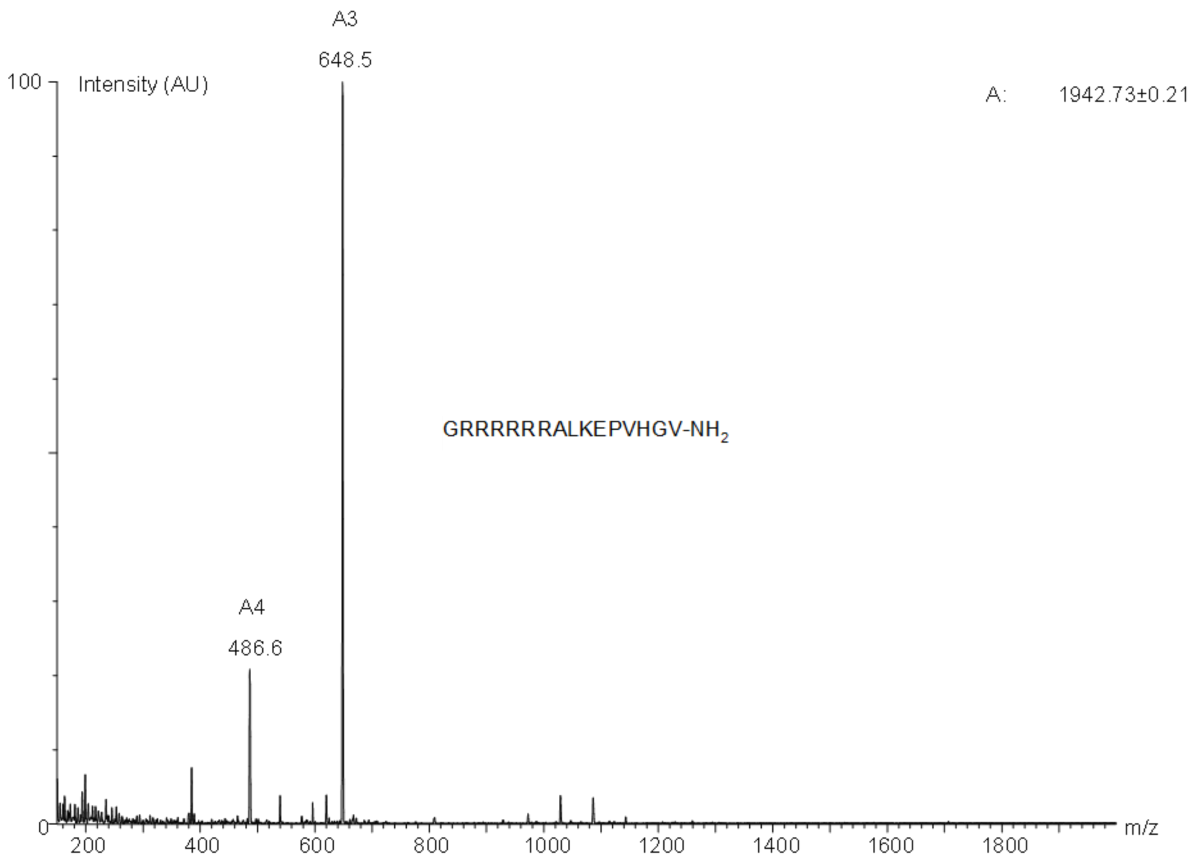

Supplementary Figure 153. MS trace of peak at Rt = 11.64 min from LC-MS analysis of the reaction of peptide thioester Ac-ALKEPVHGVpSGpSA-MPAA **1a** with peptide GRRRRRRALKEPVHGV-NH_2 **2a** after 20 h. GRRRRRRALKEPVHGV-NH_2 **2a**. $[M+3H]^{3+}$ m/z calcd. (av.) 648.44, obs. 648.5, $[M+4H]^{4+}$ m/z calcd. (av.) 486.58, obs. 486.6.

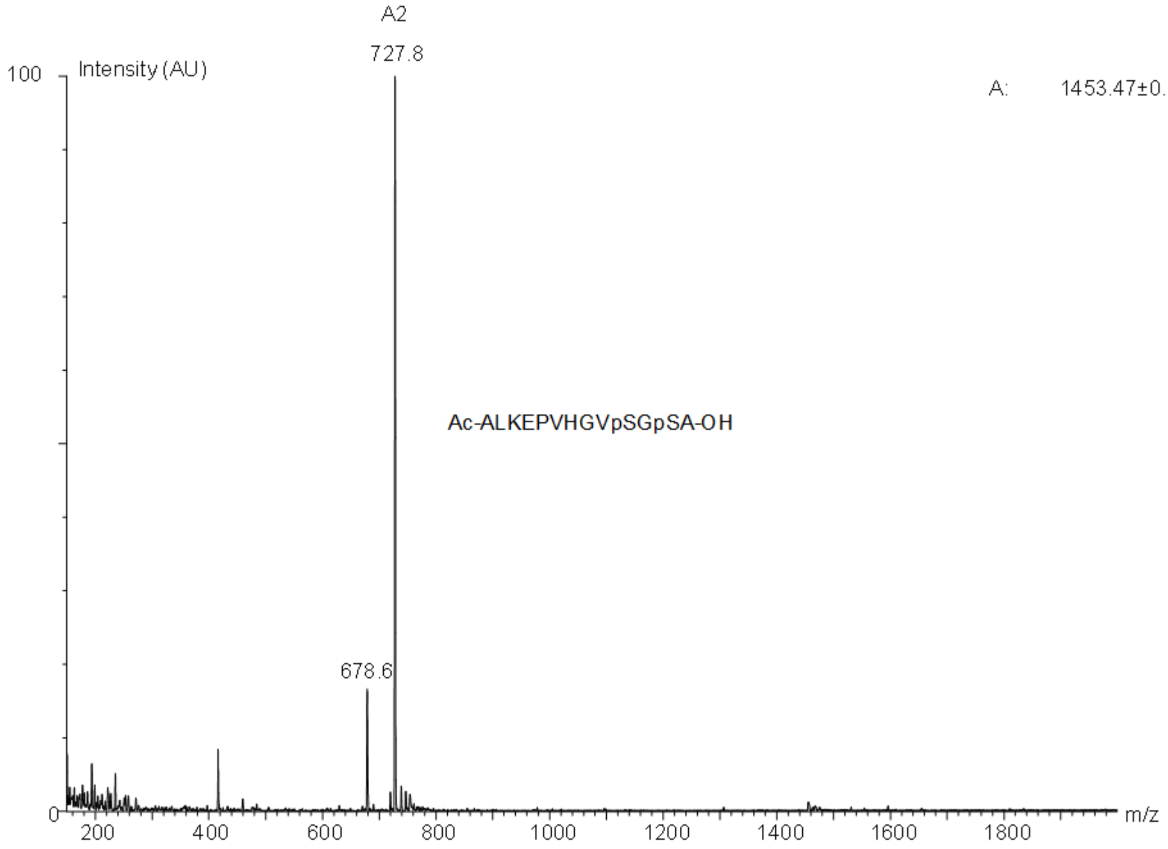

Supplementary Figure 154. MS trace of peak at Rt = 12.25 min from LC-MS analysis of the reaction of peptide thioester Ac-ALKEPVHGVpSGpSA-MPAA **1a** with peptide GRRRRRRALKEPVHGV-NH_2 **2a** after 20 h. Peptide thioester hydrolysis byproduct Ac-ALKEPVHGVpSGpSA-OH. $[M+2H]^{2+}$ m/z calcd. (av.) 727.70, obs. 727.8.

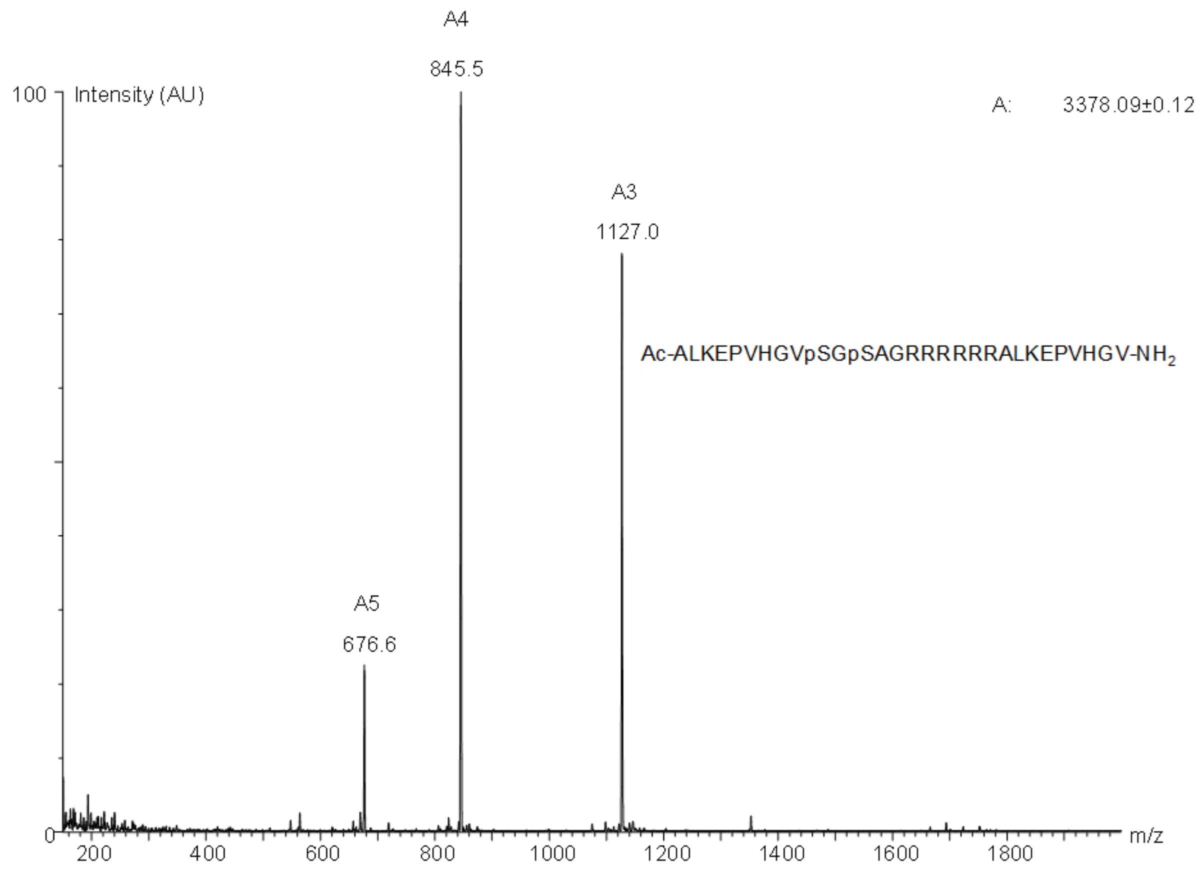

Supplementary Figure 155. MS trace of peak at Rt = 13.40 min from LC-MS analysis of the reaction of peptide thioester Ac-ALKEPVHGVpSGpSA-MPAA **1a** with peptide GRRRRRRALKEPVHGV-NH_2 **2a** after 20 h. Target ligation product Ac-ALKEPVHGVpSGpSAGRRRRRRALKEPVHGV-NH_2 **3a,a**. $[M+3H]^{3+}$ m/z calcd. (av.) 1126.90, obs. 1127.0, $[M+4H]^{4+}$ m/z calcd. (av.) 845.43, obs. 845.5, $[M+5H]^{5+}$ m/z calcd. (av.) 676.54, obs. 676.6.

1b + 2a → 3b,a:

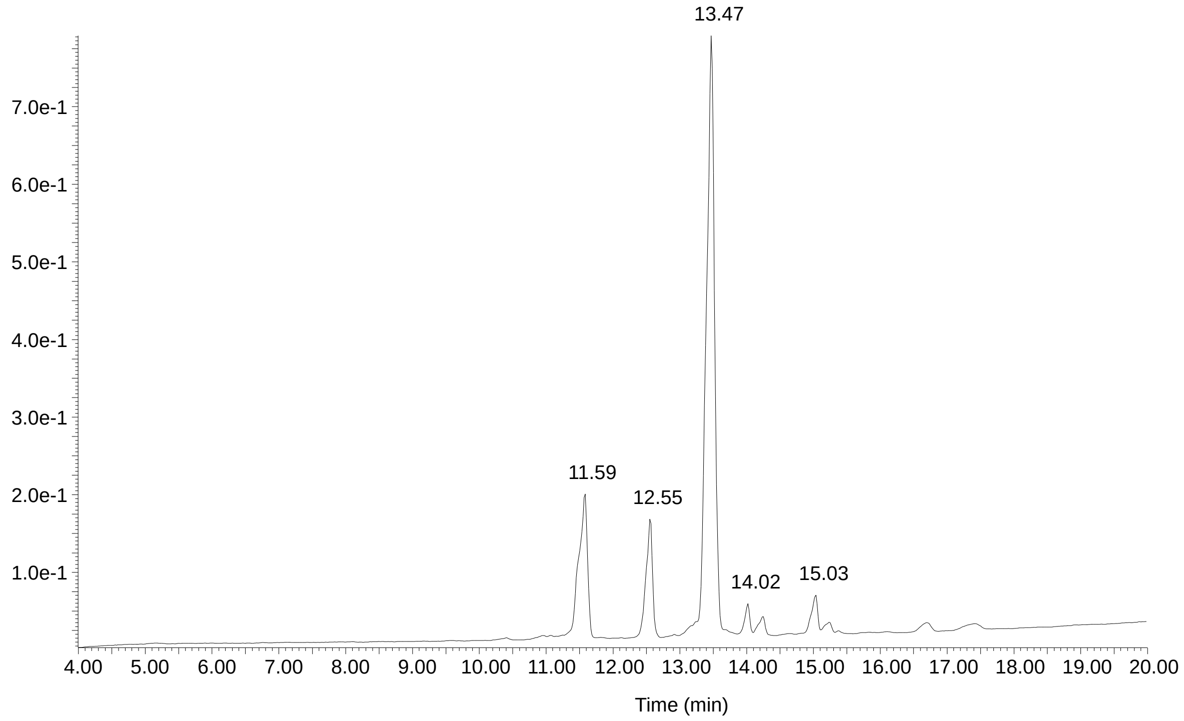

Supplementary Figure 156. LC-MS analysis of the reaction of peptide thioester Ac-ALKEPVHGVSGpSA-MPAA **1b** (one proximal pSer) with peptide GRRRRRRALKEPVHGV-NH_2 **2a** after 20 h. LC trace. Eluent A 0.1% TFA in water, eluent B 0.1% TFA in CH_3CN. C18 X bridge BEH 300 Å (5 µm, 4.6 × 250 mm) column, gradient 0-50% B in 30 min, 1 mL min^{-1}, detection at 215 nm).

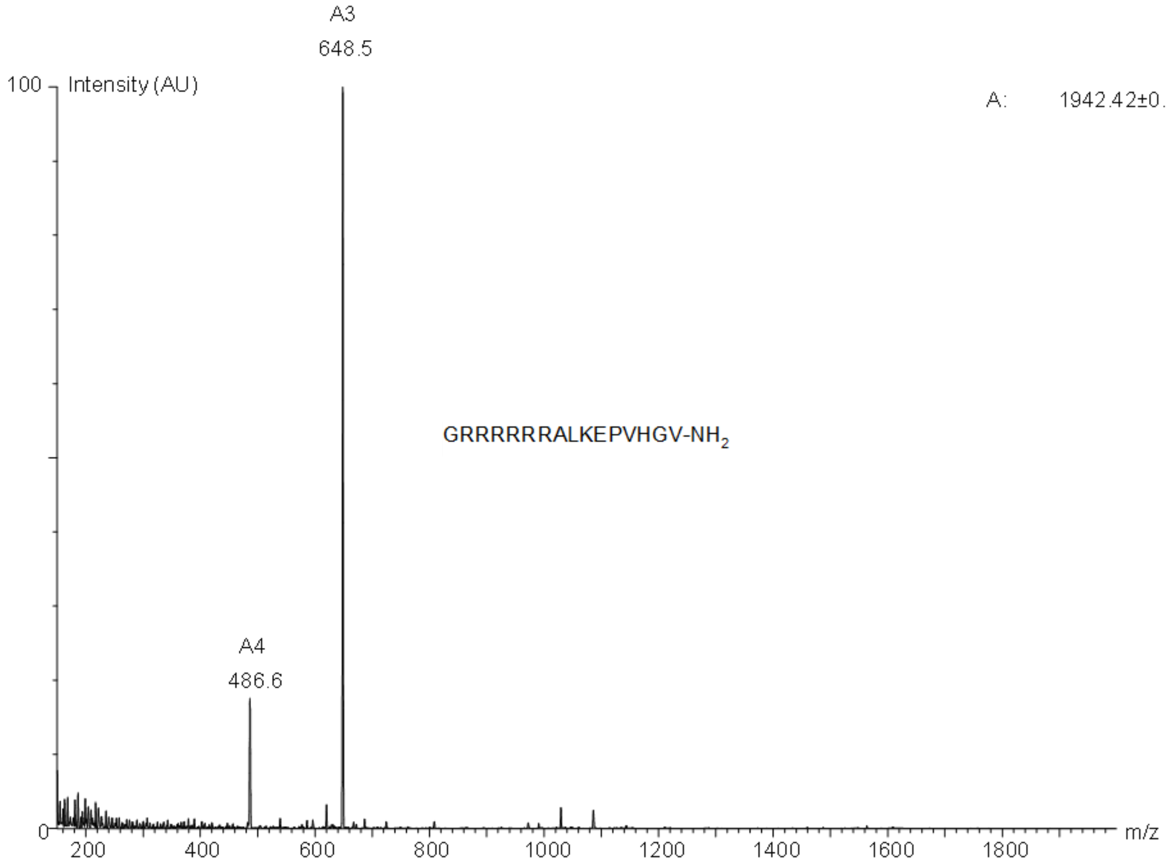

Supplementary Figure 157. MS trace of peak at Rt = 11.59 min from LC-MS analysis of the reaction of peptide thioester Ac-ALKEPVHGVSGpSA-MPAA **1b** (one proximal pSer) with peptide GRRRRRRALKEPVHGV-NH_2 **2a** after 20 h. GRRRRRRALKEPVHGV-NH_2 **2a**. $[M+3H]^{3+}$ m/z calcd. (av.) 648.44, obs. 648.5, $[M+4H]^{4+}$ m/z calcd. (av.) 486.58, obs. 486.6.

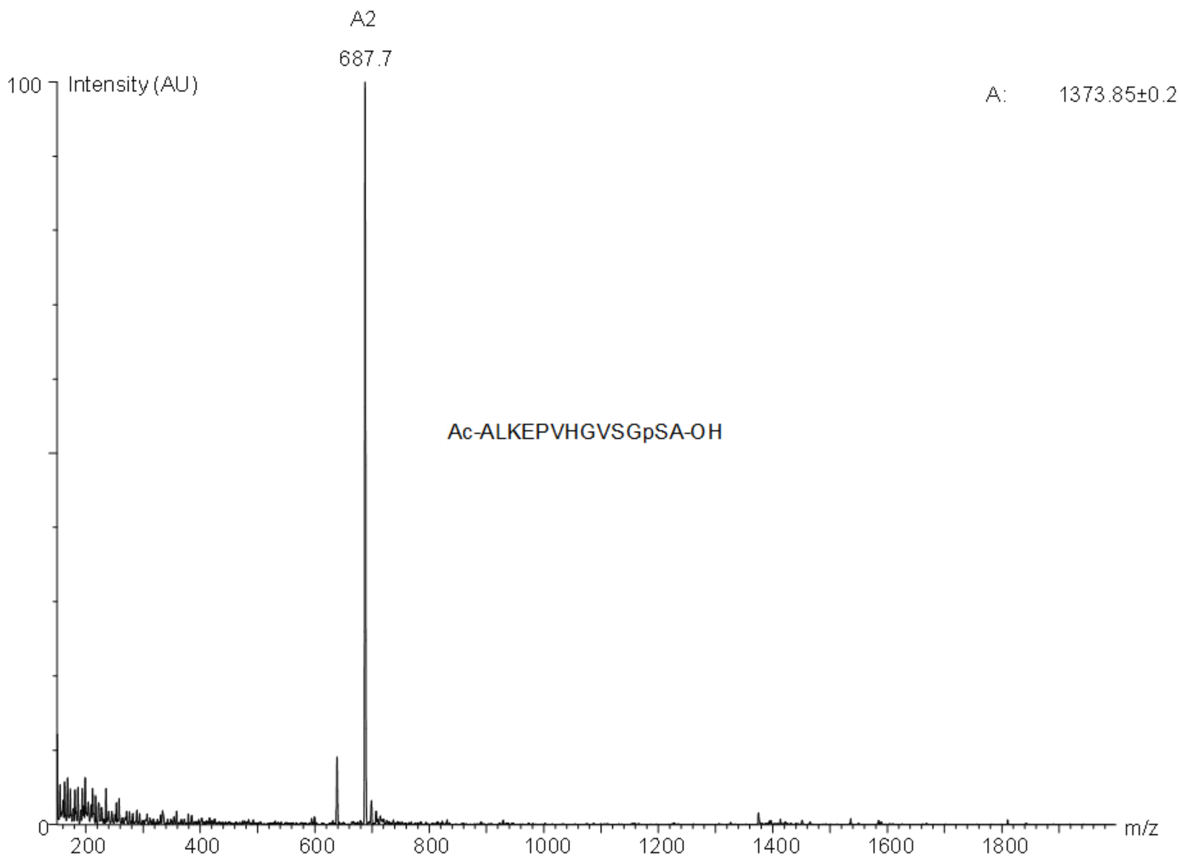

Supplementary Figure 158. MS trace of peak at Rt = 12.55 min from LC-MS analysis of the reaction of peptide thioester Ac-ALKEPVHGVSGpSA-MPAA **1b** (one proximal pSer) with peptide GRRRRRRALKEPVHGV-NH_2 **2a** after 20 h. Peptide thioester hydrolysis byproduct Ac-ALKEPVHGVSGpSA-OH. $[M+2H]^{2+}$ m/z calcd. (av.) 687.71, obs. 687.7.

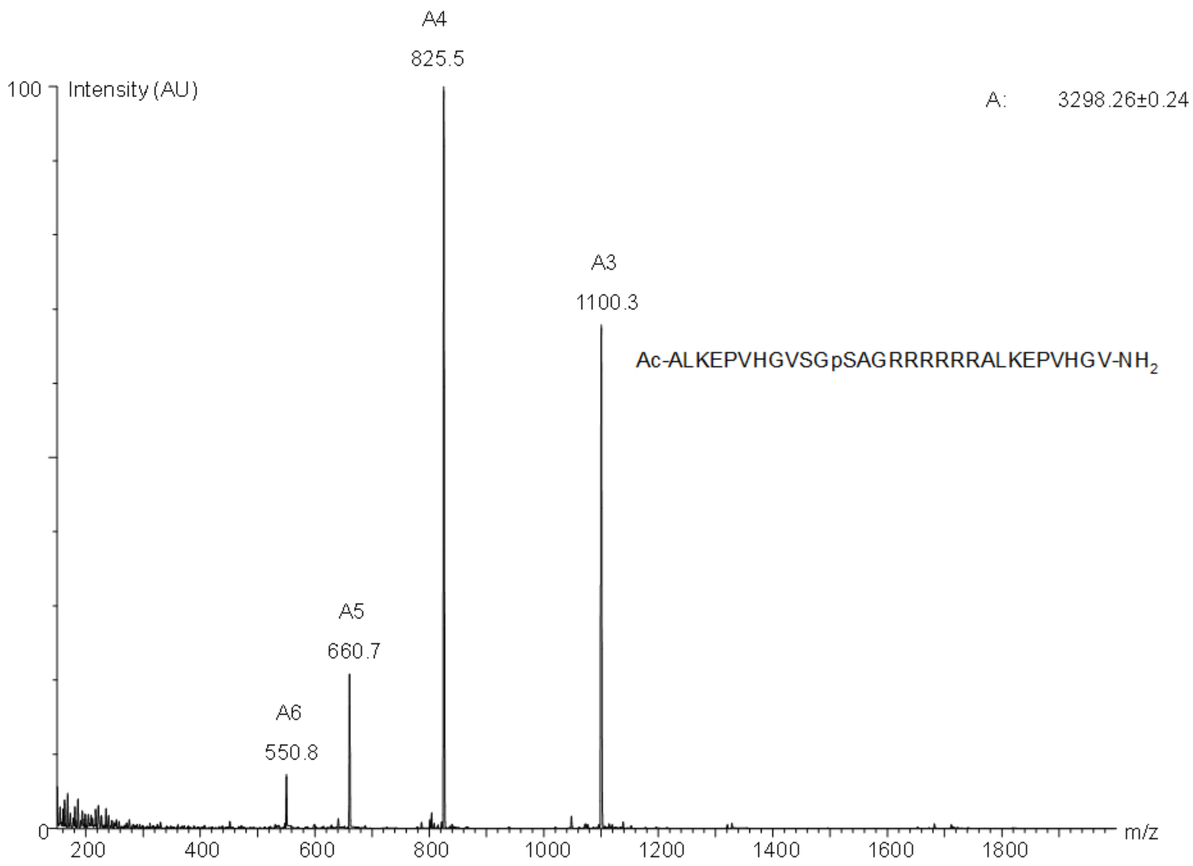

Supplementary Figure 159. MS trace of peak at Rt = 13.47 min from LC-MS analysis of the reaction of peptide thioester Ac-ALKEPVHGVSGpSA-MPAA **1b** (one proximal pSer) with peptide GRRRRRRALKEPVHGV-NH_2 **2a** after 20 h. Target ligation product Ac-ALKEPVHGVSGpSAGRRRRRRALKEPVHGV-NH_2 **3b,a**. $[M+3H]^{3+}$ m/z calcd. (av.) 1100.24, obs. 1100.3, $[M+4H]^{4+}$ m/z calcd. (av.) 825.43, obs. 825.5, $[M+5H]^{5+}$ m/z calcd. (av.) 660.55, obs. 660.7, $[M+6H]^{6+}$ m/z calcd. (av.) 550.62, obs. 550.8.

1c + 2a → 3c,a:

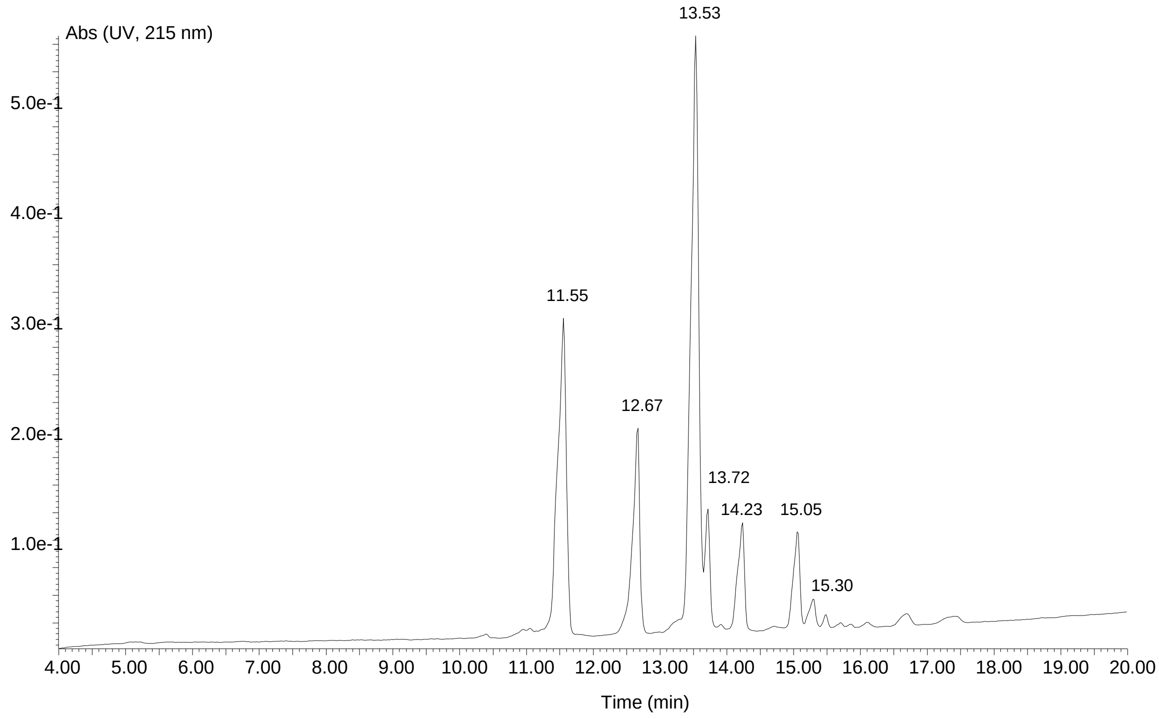

Supplementary Figure 160. LC-MS analysis of the reaction of peptide thioester Ac-ALKEPVHGVpSGSA-MPAA **1c** (one distant pSer) with peptide GRRRRRRALKEPVHGV-NH_2 **2a** after 20 h. LC trace. Eluent A 0.1% TFA in water, eluent B 0.1% TFA in CH_3CN. C18 X bridge BEH 300 Å (5 µm, 4.6 × 250 mm) column, gradient 0-50% B in 30 min, 1 mL min^{-1}, detection at 215 nm).

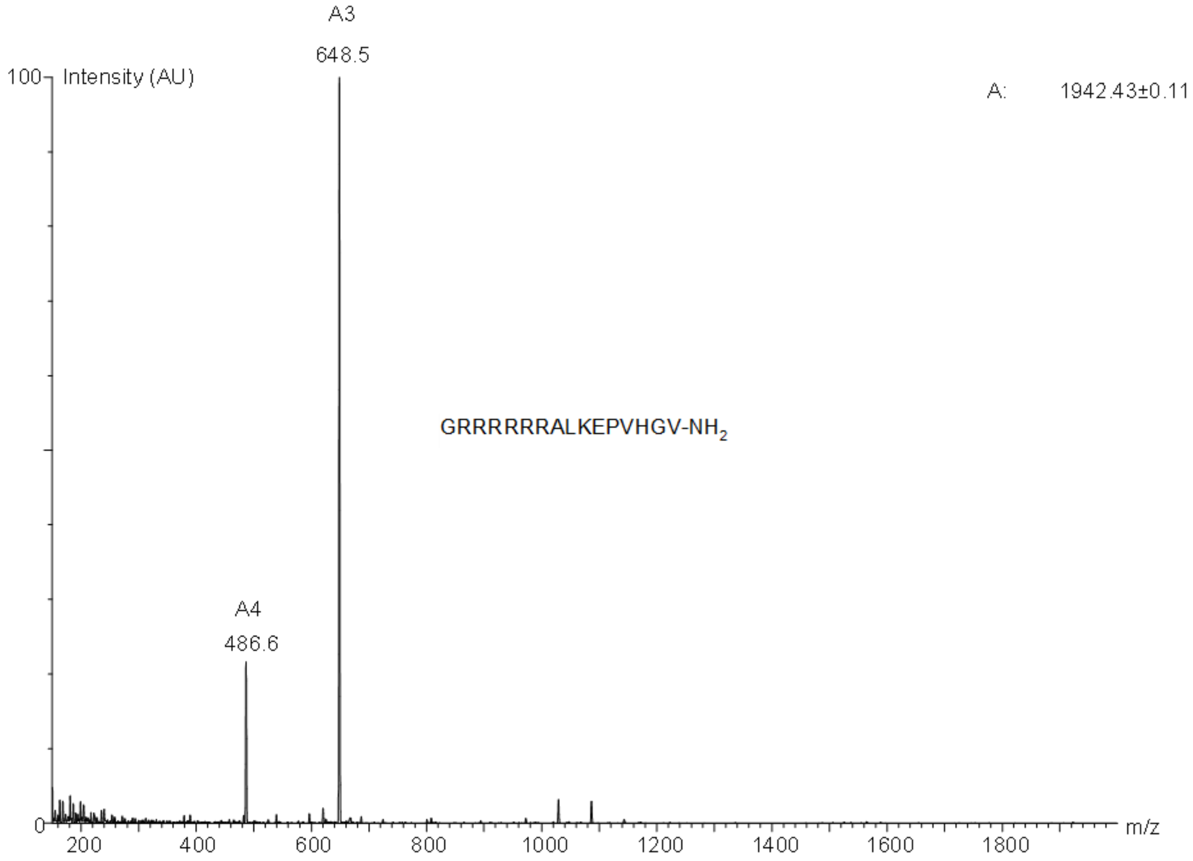

Supplementary Figure 161. MS trace of peak at Rt = 11.55 min from LC-MS analysis of the reaction of peptide thioester Ac-ALKEPVHGVpSGSA-MPAA **1c** (one distant pSer) with peptide GRRRRRRALKEPVHGV-NH_2 **2a** after 20 h. GRRRRRRALKEPVHGV-NH_2 **2a**. $[M+3H]^{3+}$ m/z calcd. (av.) 648.44, obs. 648.5, $[M+4H]^{4+}$ m/z calcd. (av.) 486.58, obs. 486.6.

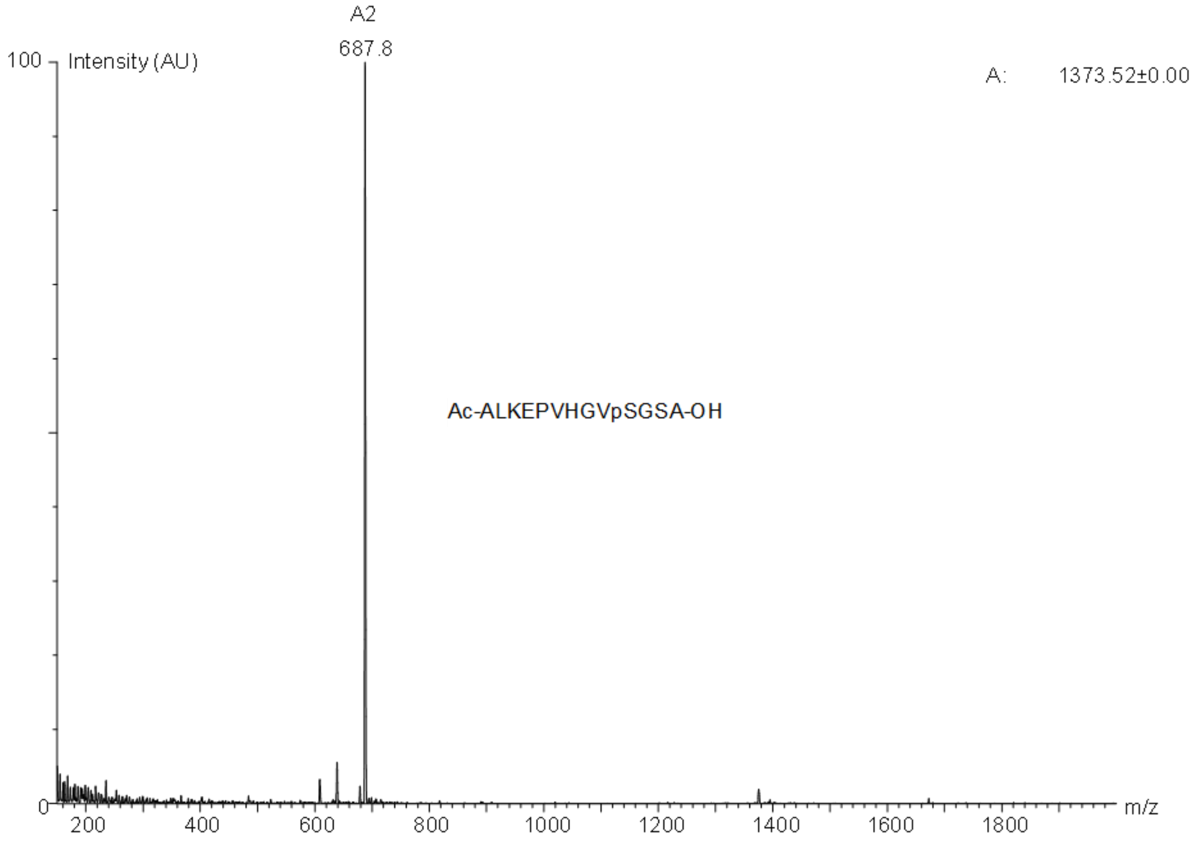

Supplementary Figure 162. MS trace of peak at Rt = 12.67 min from LC-MS analysis of the reaction of peptide thioester Ac-ALKEPVHGVpSGSA-MPAA **1c** (one distant pSer) with peptide GRRRRRRALKEPVHGV-NH_2 **2a** after 20 h. Peptide thioester hydrolysis byproduct Ac-ALKEPVHGVpSGSA-OH. $[M+2H]^{2+}$ m/z calcd. (av.) 687.71, obs. 687.8.

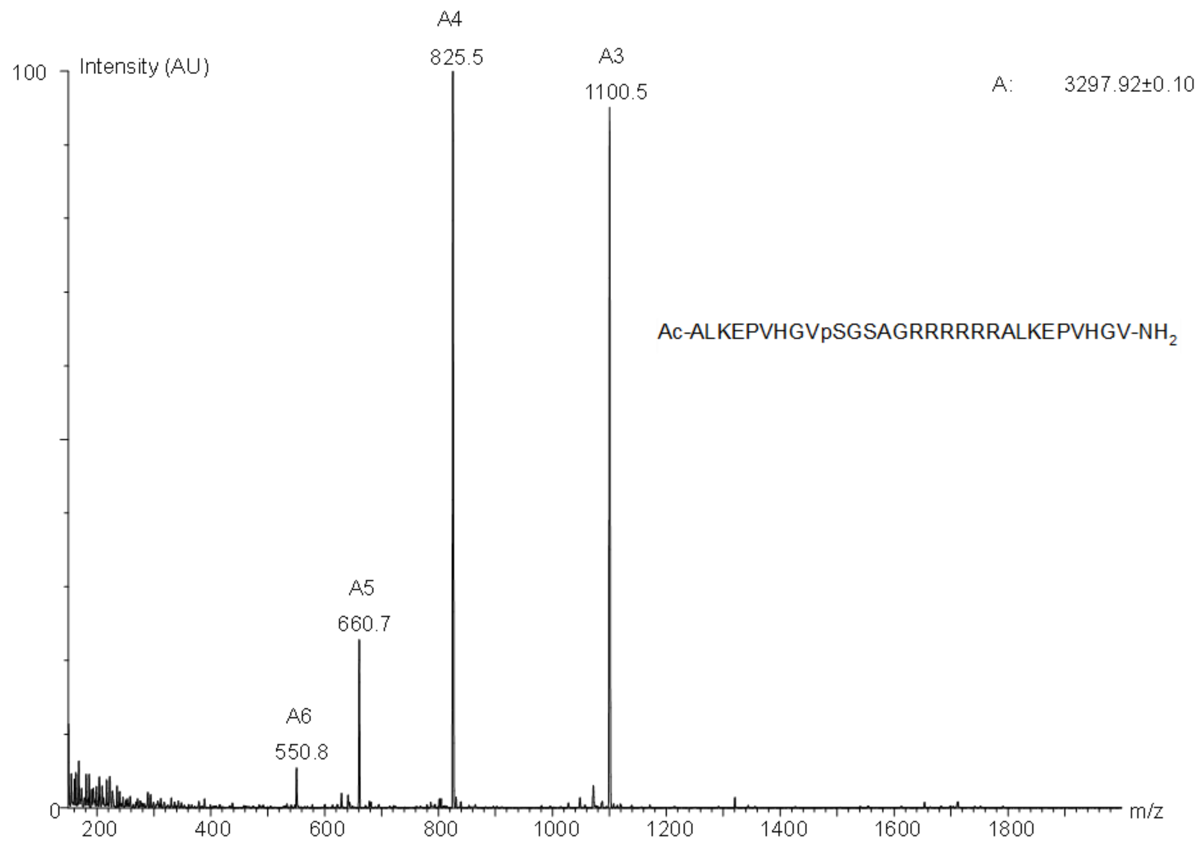

Supplementary Figure 163. MS trace of peak at Rt = 13.53 min from LC-MS analysis of the reaction of peptide thioester Ac-ALKEPVHGVpSGSA-MPAA **1c** (one distant pSer) with peptide GRRRRRRALKEPVHGV-NH_2 **2a** after 20 h. Target ligation product Ac-ALKEPVHGVpSGSAGRRRRRRALKEPVHGV-NH_2 **3c,a**. $[M+3H]^{3+}$ m/z calcd. (av.) 1100.24, obs. 1100.5, $[M+4H]^{4+}$ m/z calcd. (av.) 825.43, obs. 825.5, $[M+5H]^{5+}$ m/z calcd. (av.) 660.55, obs. 660.7, $[M+6H]^{6+}$ m/z calcd. (av.) 550.62, obs. 550.8.

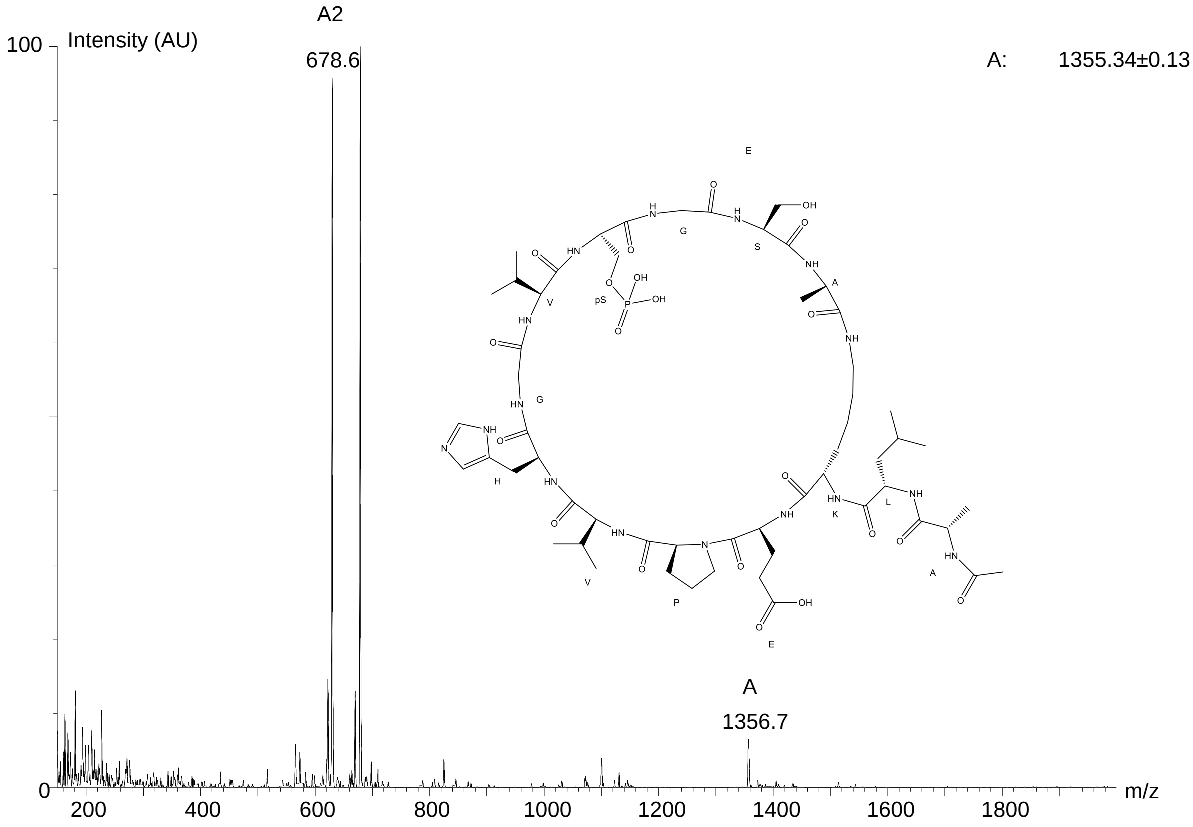

Supplementary Figure 164. MS trace of peak at Rt = 13.72 min from LC-MS analysis of the reaction of peptide thioester Ac-ALKEPVHGVpSGSA-MPAA **1c** (one distant pSer) with peptide GRRRRRRALKEPVHGV-NH_2 **2a** after 20 h. Peptide thioester cyclized byproduct. $[M+H]^{+}$ m/z calcd. (av.) 1356.41, obs. 1356.7, $[M+2H]^{2+}$ m/z calcd. (av.) 678.70, obs. 678.6.

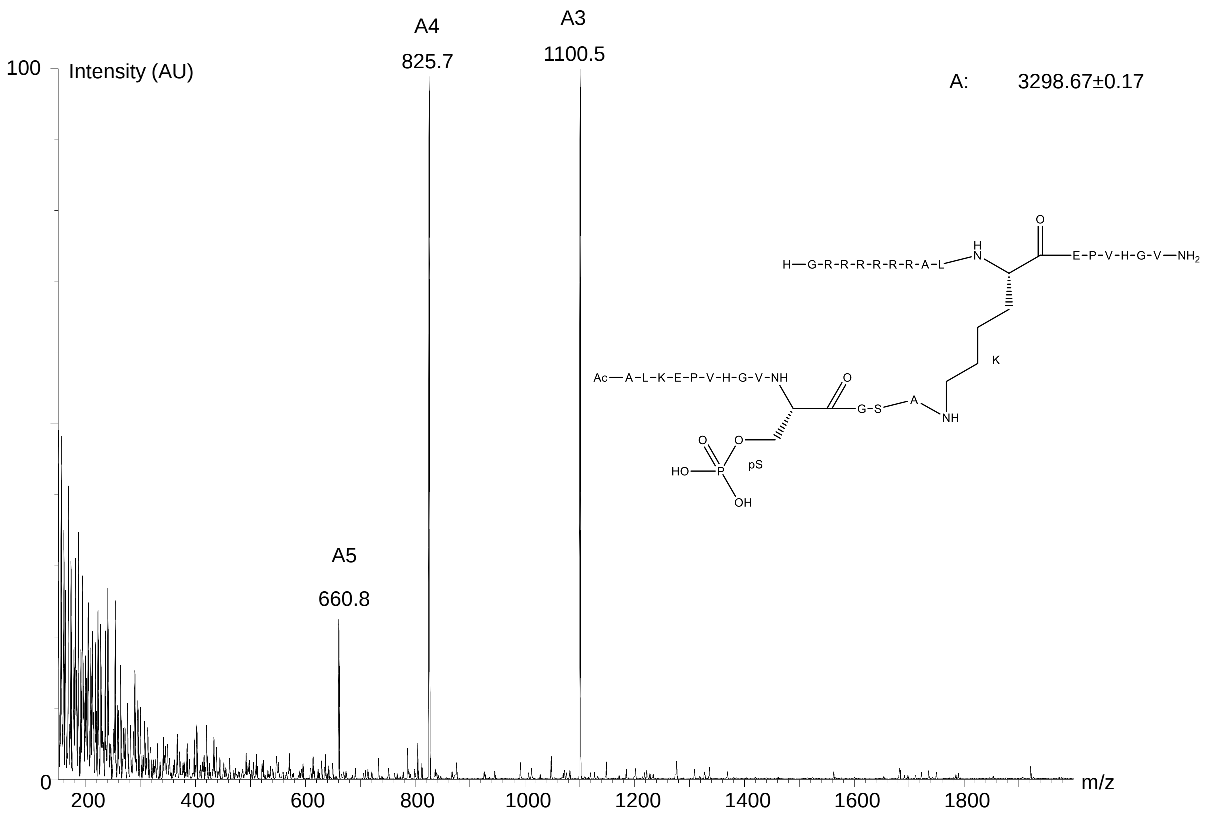

Supplementary Figure 165. MS trace of peak at Rt = 14.23 min from LC-MS analysis of the reaction of peptide thioester Ac-ALKEPVHGVpSGSA-MPAA **1c** (one distant pSer) with peptide GRRRRRRALKEPVHGV-NH_2 **2a** after 20 h. Branched byproduct GRRRRRRALK(Ac-ALKEPVHGVpSGSA)EPVHGV-NH_2. $[M+3H]^{3+}$ m/z calcd. (av.) 1100.24, obs. 1100.5, $[M+4H]^{4+}$ m/z calcd. (av.) 825.43, obs. 825.7, $[M+5H]^{5+}$ m/z calcd. (av.) 660.55, obs. 660.8.

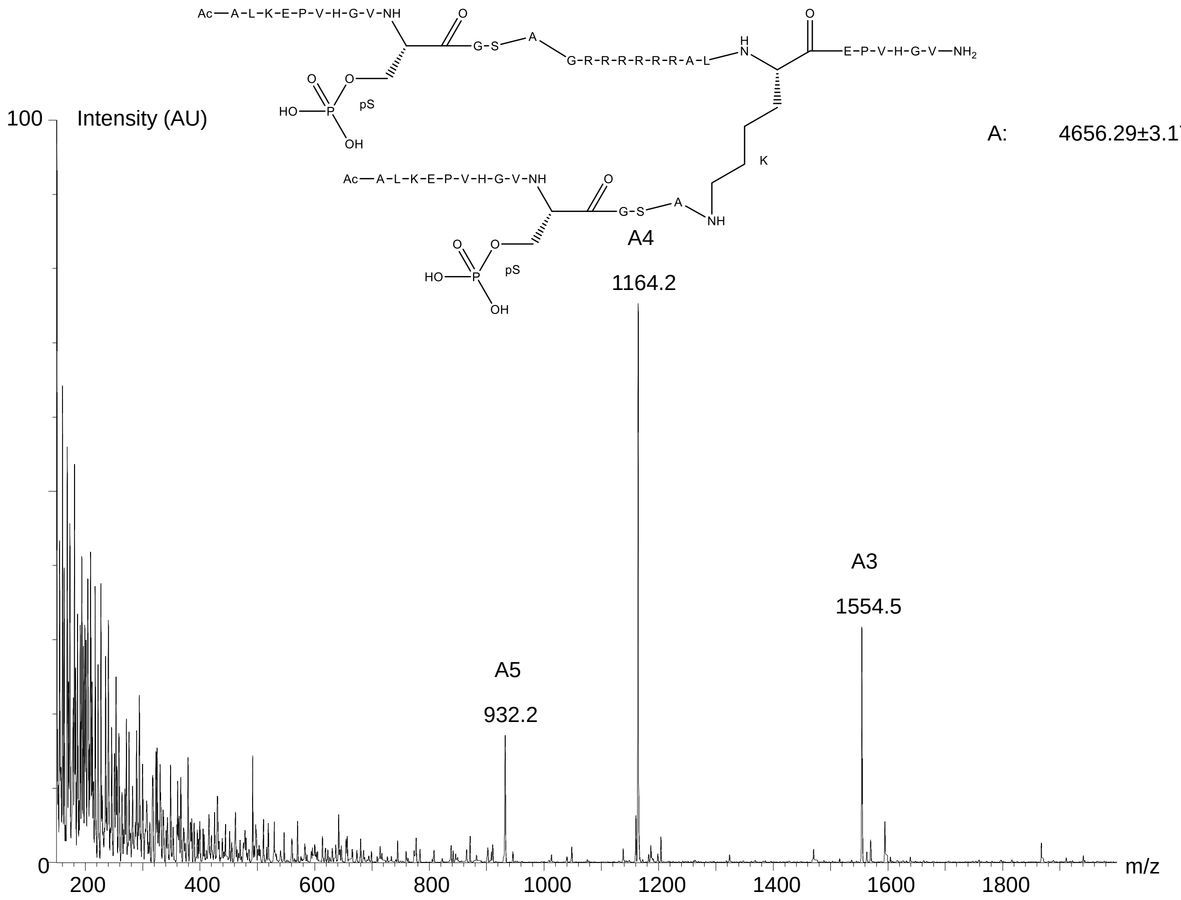

Supplementary Figure 166. MS trace of peak at Rt = 15.05 min from LC-MS analysis of the reaction of peptide thioester Ac-ALKEPVHGVpSGSA-MPAA **1c** (one distant pSer) with peptide GRRRRRRALKEPVHGV-NH_2 **2a** after 20 h. Branched byproduct Ac-ALKEPVHGVpSGSAGRRRRRRALK(Ac-ALKEPVHGVpSGSA)EPVHGV-NH_2. $[M+3H]^{3+}$ m/z calcd. (av.) 1552.05, obs. 1554.5, $[M+4H]^{4+}$ m/z calcd. (av.) 1164.28, obs. 1164.2, $[M+5H]^{5+}$ m/z calcd. (av.) 931.63, obs. 932.2.

1d + 2a → 3d,a:

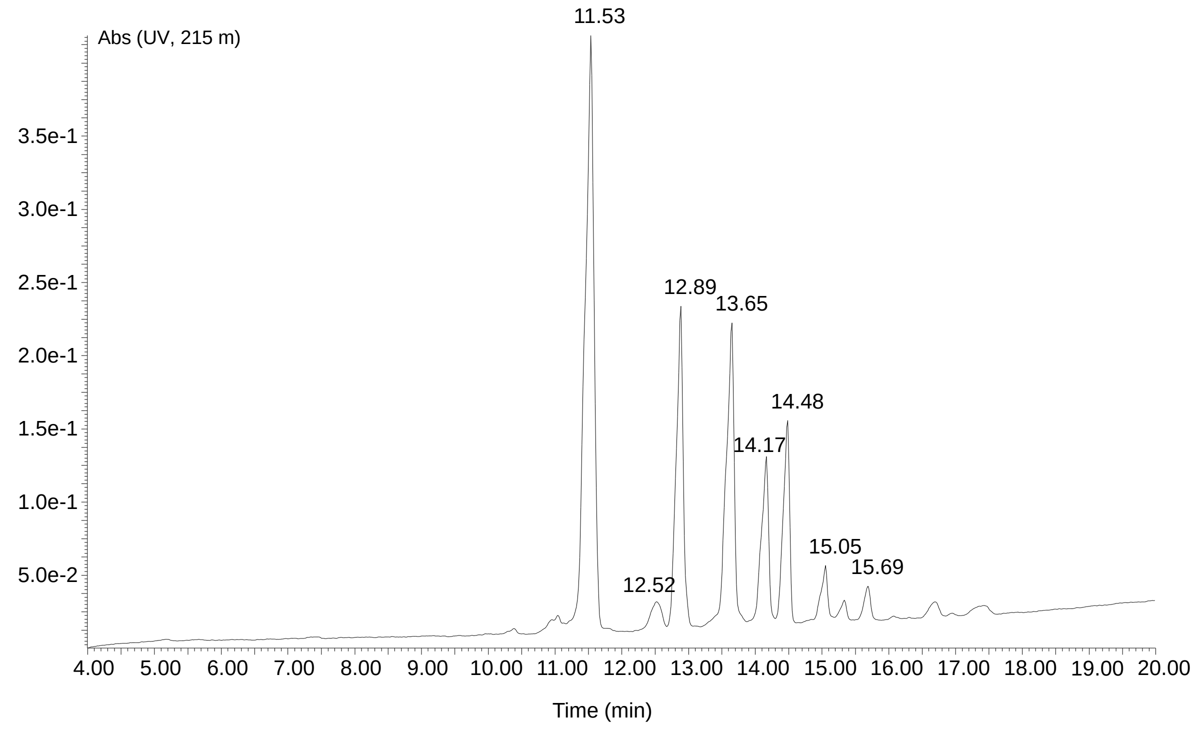

Supplementary Figure 167. LC-MS analysis of the reaction of peptide thioester Ac-ALKEPVHGVSGSA-MPAA **1d** (no pSer) with peptide GRRRRRRALKEPVHGV-NH_2 **2a** after 20 h. LC trace. Eluent A 0.1% TFA in water, eluent B 0.1% TFA in CH_3CN. C18 X bridge BEH 300 Å (5 µm, 4.6 × 250 mm) column, gradient 0-50% B in 30 min, 1 mL min^{-1}, detection at 215 nm).

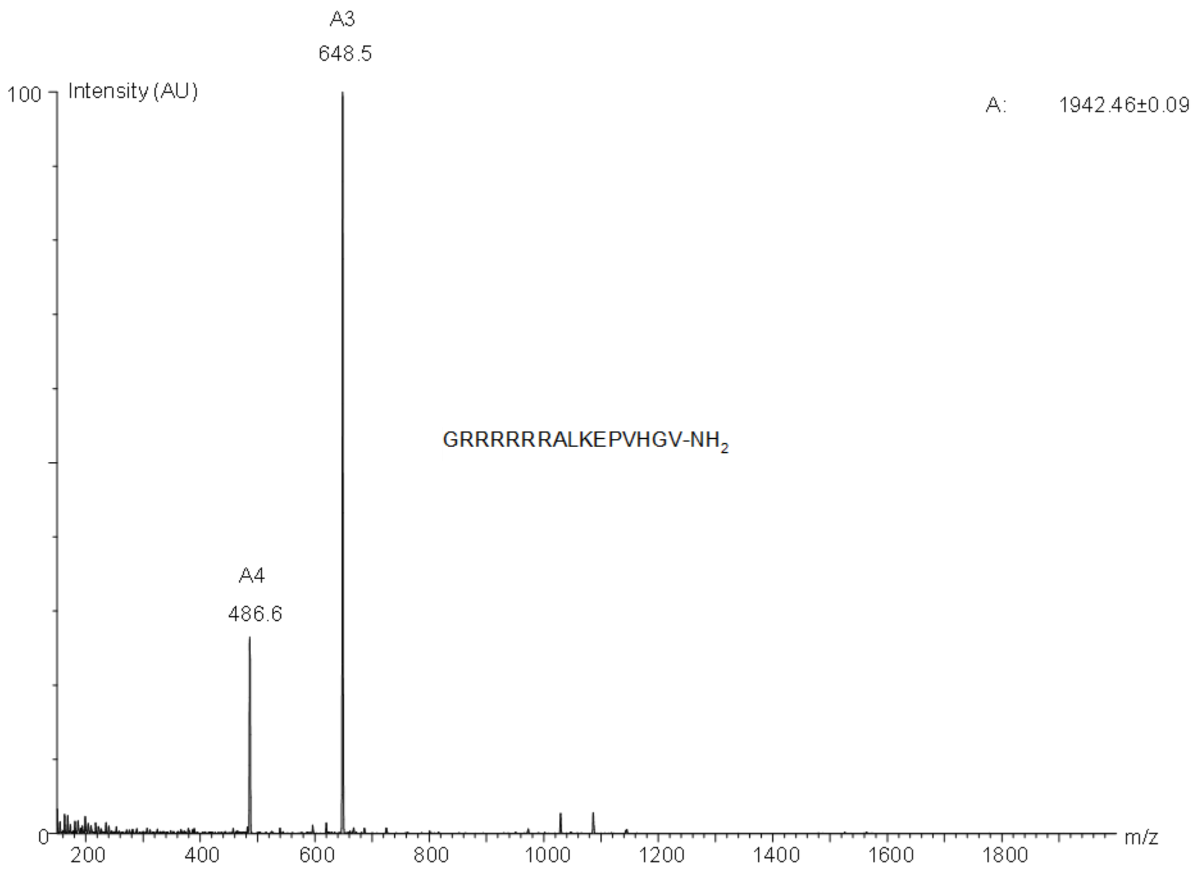

Supplementary Figure 168. MS trace of peak at Rt = 11.53 min from LC-MS analysis of the reaction of peptide thioester Ac-ALKEPVHGVSGSA-MPAA **1d** (no pSer) with peptide GRRRRRRALKEPVHGV-NH_2 **2a** after 20 h. GRRRRRRALKEPVHGV-NH_2 **2a**. $[M+3H]^{3+}$ m/z calcd. (av.) 648.44, obs. 648.5, $[M+4H]^{4+}$ m/z calcd. (av.) 486.58, obs. 486.6.

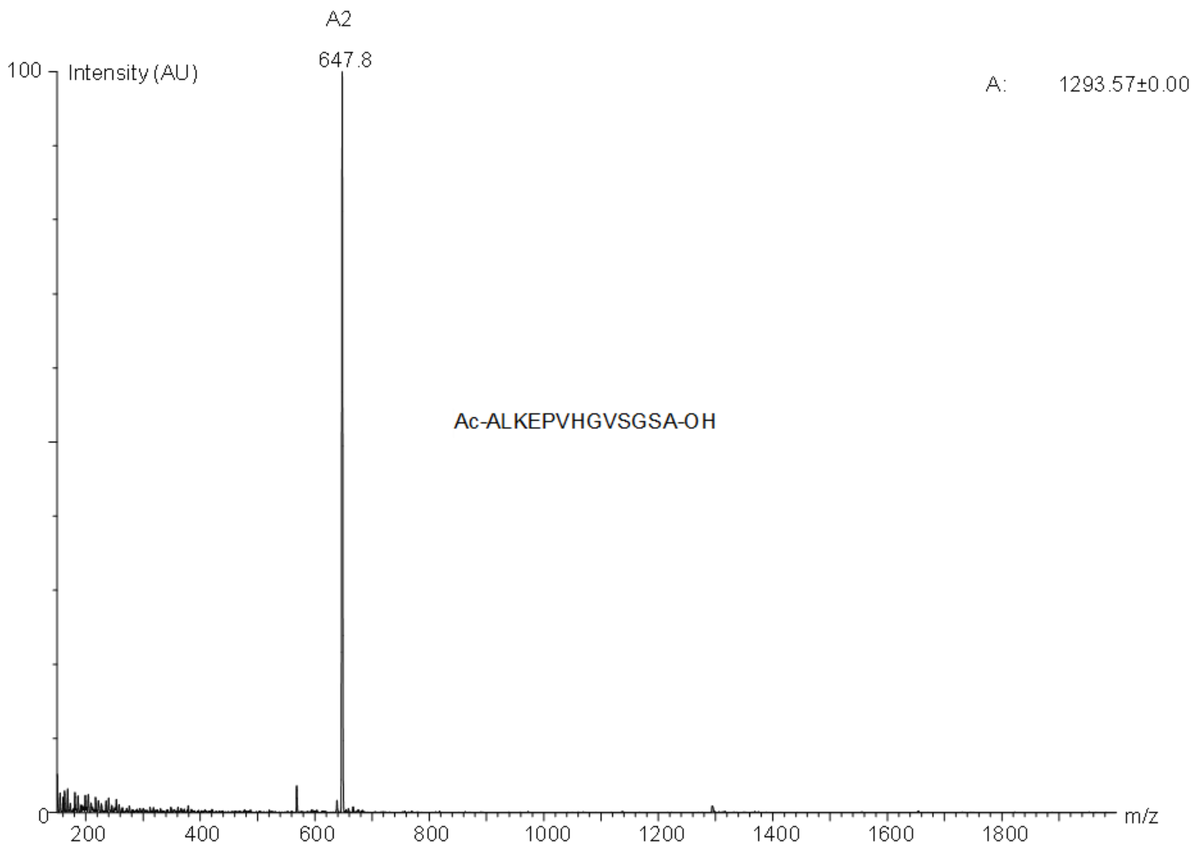

Supplementary Figure 169. MS trace of peak at Rt = 12.89 min from LC-MS analysis of the reaction of peptide thioester Ac-ALKEPVHGVSGSA-MPAA **1d** (no pSer) with peptide GRRRRRRALKEPVHGV-NH_2 **2a** after 20 h. Peptide thioester hydrolysis byproduct Ac-ALKEPVHGVSGSA-OH. $[M+2H]^{2+}$ m/z calcd. (av.) 647.72, obs. 647.8.

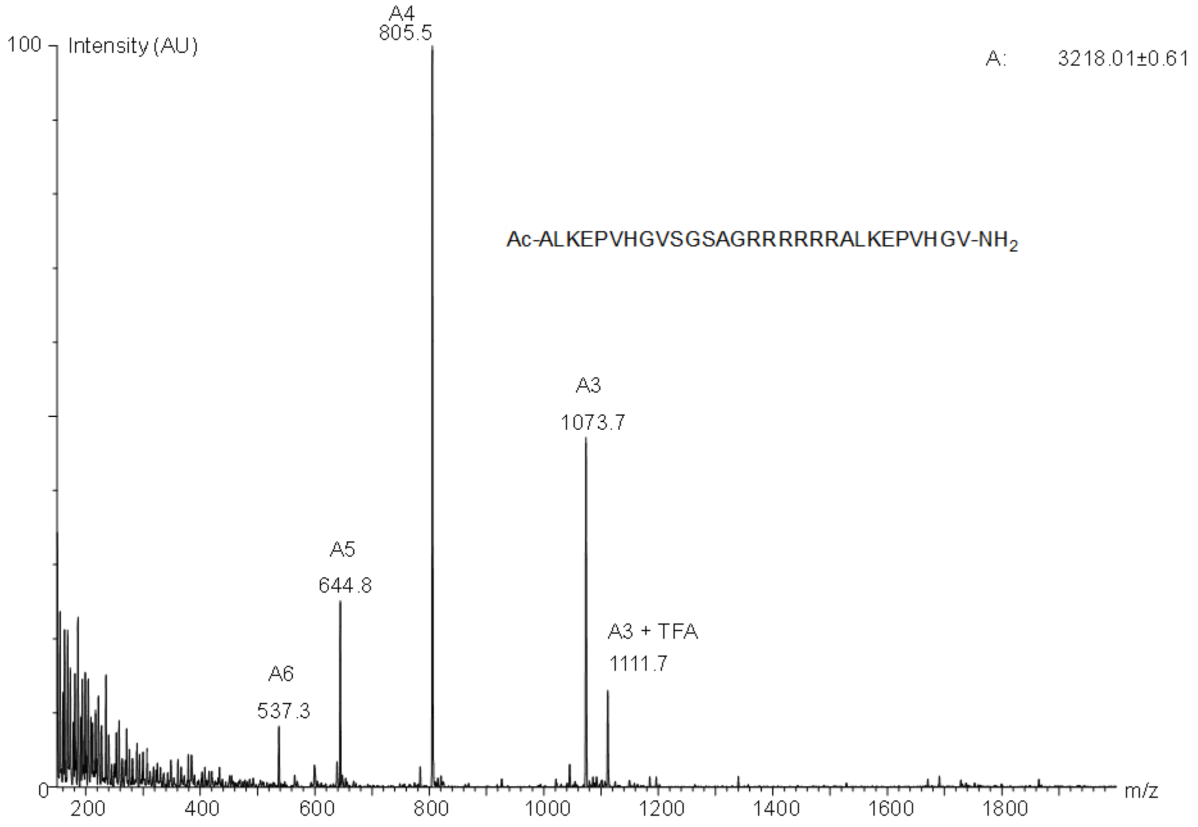

Supplementary Figure 170. MS trace of peak at Rt = 13.65 min from LC-MS analysis of the reaction of peptide thioester Ac-ALKEPVHGVSGSA-MPAA **1d** (no pSer) with peptide GRRRRRRALKEPVHGV-NH_2 **2a** after 20 h. Target ligation product Ac-ALKEPVHGVSGSAGRRRRRRALKEPVHGV-NH_2 **3d,a**. $[M+3H]^{3+}$ m/z calcd. (av.) 1073.58, obs. 1073.7, $[M+4H]^{4+}$ m/z calcd. (av.) 805.44, obs. 805.5, $[M+5H]^{5+}$ m/z calcd. (av.) 644.55, obs. 644.8, $[M+6H]^{6+}$ m/z calcd. (av.) 537.29, obs. 537.3.

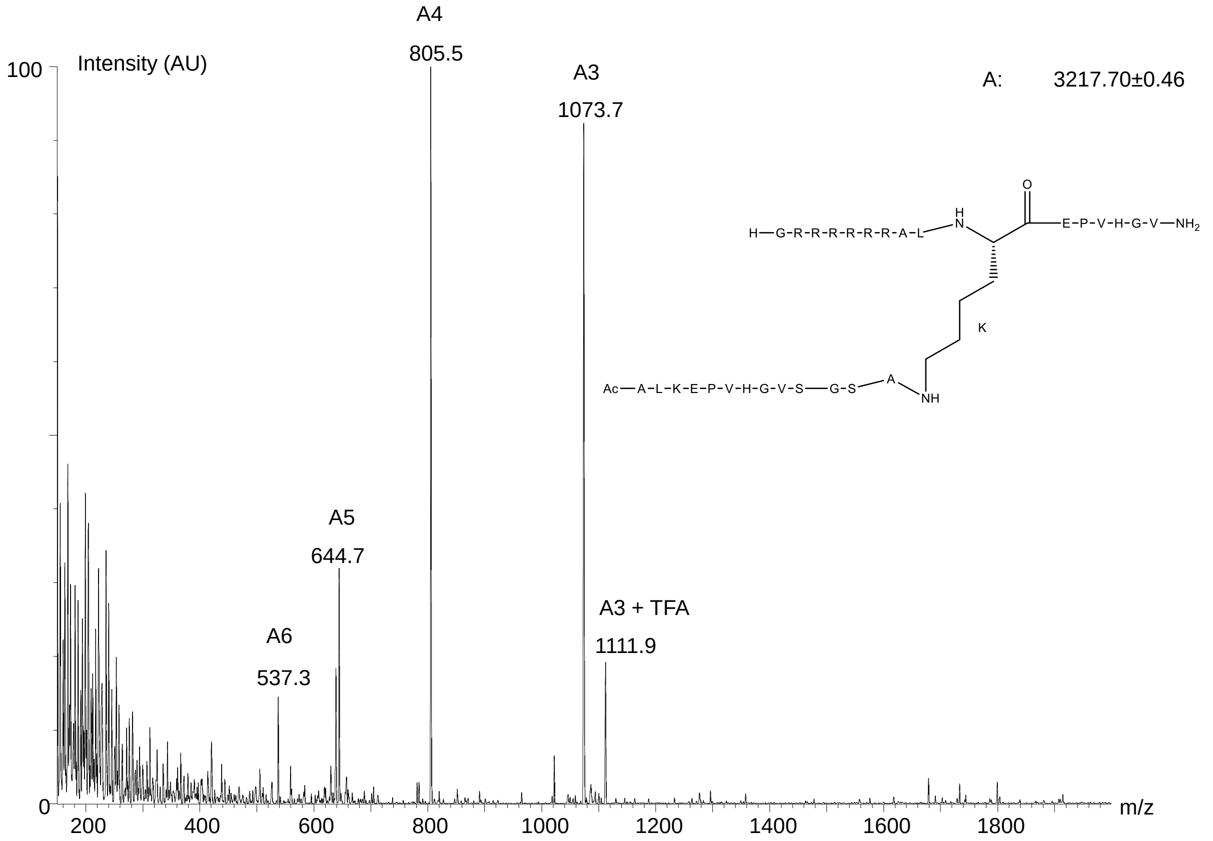

Supplementary Figure 171. MS trace of peak at Rt = 14.17 min from LC-MS analysis of the reaction of the reaction of peptide thioester Ac-ALKEPVHGVSGSA-MPAA **1d** (no pSer) with peptide GRRRRRRALKEPVHGV-NH_2 **2a** after 20 h. Branched byproduct GRRRRRRALK(Ac-ALKEPVHGVSGSA)EPVHGV-NH_2. $[M+3H]^{3+}$ m/z calcd. (av.) 1073.58, obs. 1073.7, $[M+4H]^{4+}$ m/z calcd. (av.) 805.44, obs. 805.5, $[M+5H]^{5+}$ m/z calcd. (av.) 644.55, obs. 644.7, $[M+6H]^{6+}$ m/z calcd. (av.) 537.29, obs. 537.3.

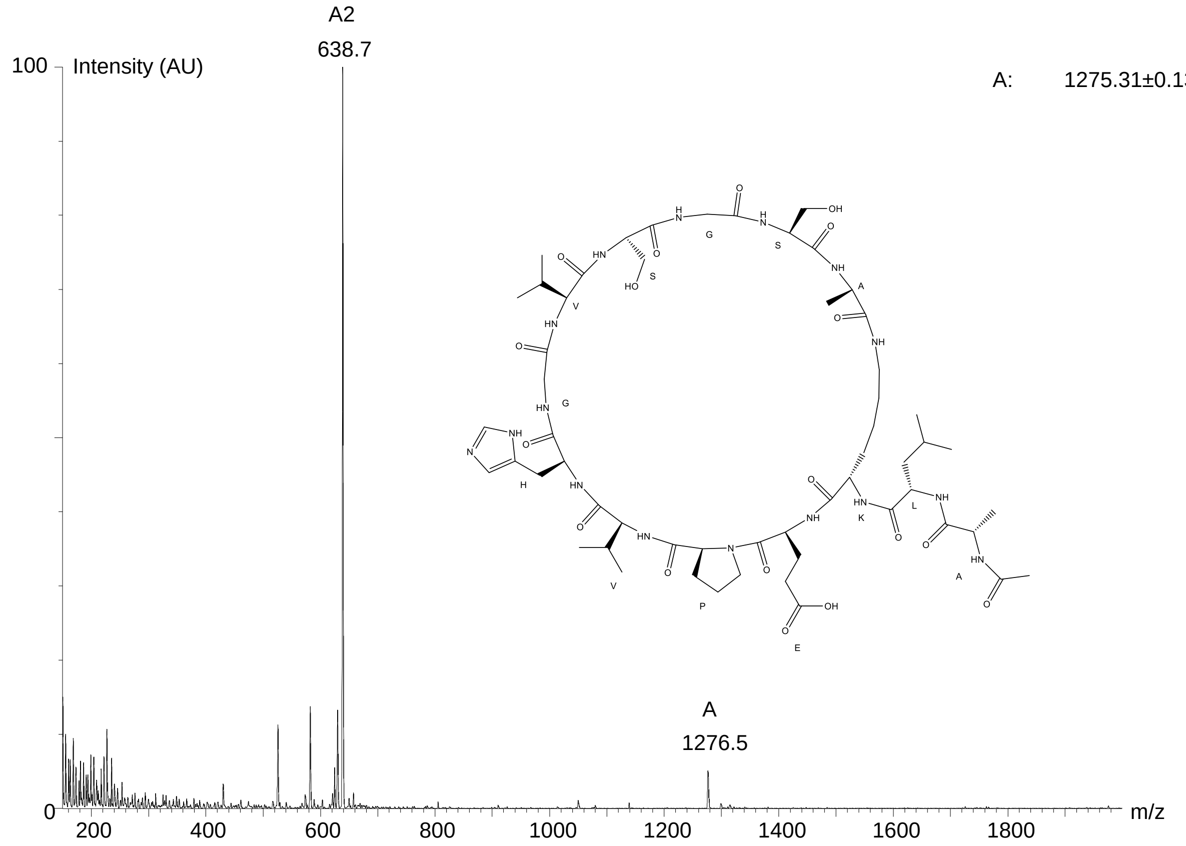

Supplementary Figure 172. MS trace of peak at Rt = 14.48 min from LC-MS analysis of the reaction of peptide thioester Ac-ALKEPVHGVSGSA-MPAA **1d** (no pSer) with peptide GRRRRRRALKEPVHGV-NH_2 **2a** after 20 h. Peptide thioester cyclization byproduct. $[M+H]^+$ m/z calcd. (av.) 1276.43, obs. 1276.5, $[M+2H]^{2+}$ m/z calcd. (av.) 638.71, obs.638.7.

Effect of the pH

The effect of the pH on the outcome of the aminolysis reaction was studied using peptides **1a** and **2a**. The pH was varied by adapting the concentration of sodium bicarbonate/CO_2 buffer (15-100 mM).

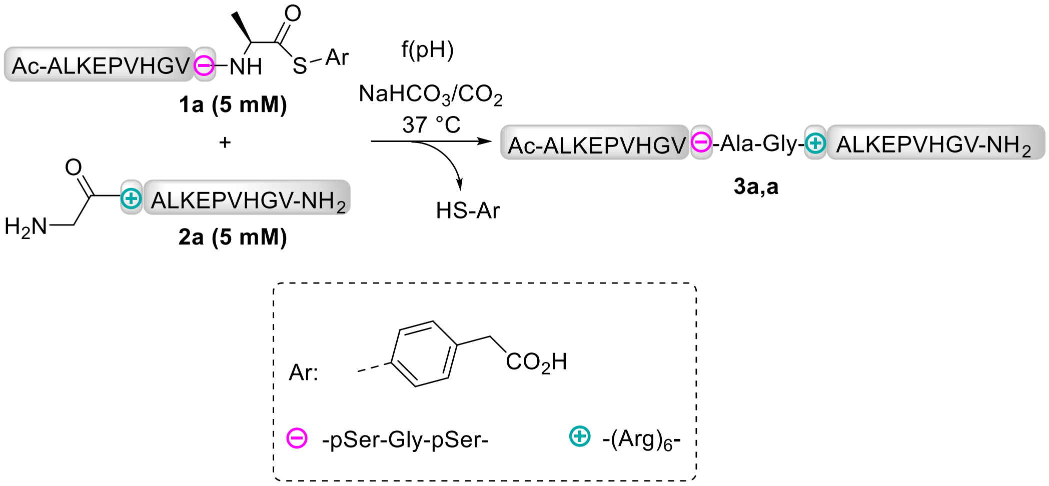

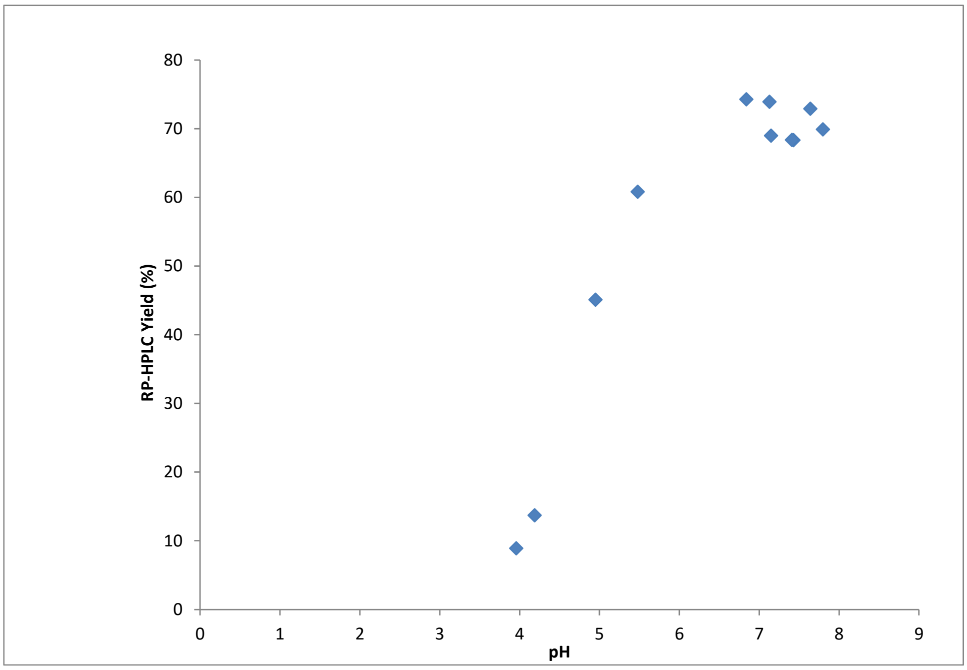

Supplementary Figure 173. Relationship between pH and the HPLC yield of peptide **3a,a** produced by the aminolysis of peptide thioester Ac-ALKEPVHGVpSGpSA-MPAA 1a by glycyl peptide GRRRRRRALKEPVHGV-NH_2 **2a** (5 mM final peptide concentrations). The pH was varied by adapting the concentration of sodium bicarbonate (15-100 mM).

Effect of phosphate buffer concentration

The effect of sodium phosphate concentration on the outcome of the aminolysis reaction was studied using peptides **1a** and **2a** (pH=7.0-7.1).

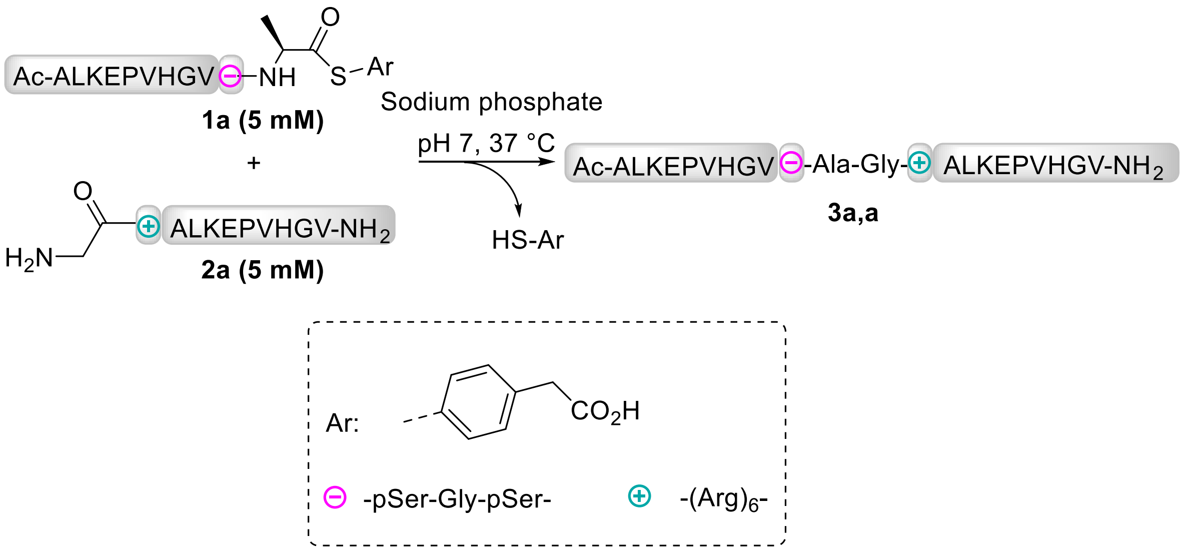

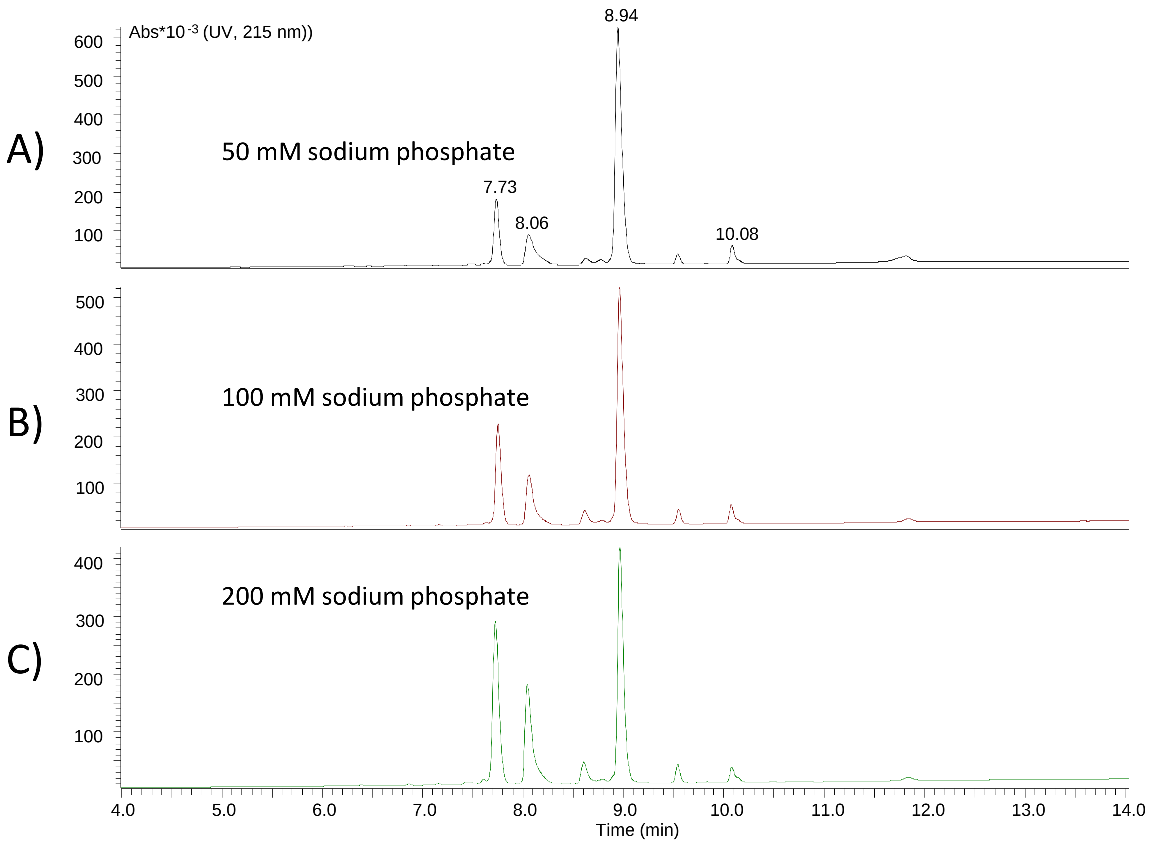

Supplementary Figure 174. UPLC-MS analysis of the reaction of peptide thioester Ac-ALKEPVHGVpSGpSA-MPAA **1a** with glycyl peptide GRRRRRRALKEPVHGV-NH_2 **2a** after 40 h 30 min in presence of increasing concentrations of sodium phosphate buffer at pH=7.0-7.1. LC trace. Eluent A 0.1% TFA in water, eluent B 0.1% TFA in CH_3CN. C18 BEH 300 Å (1.7 µm, 2.1 × 100 mm) column, gradient 0-40% B in 15 min, 0.4 mL min^{-1}, detection at 215 nm). A) 50 mM sodium phosphate. B) 100 mM sodium phosphate. C) 200 mM sodium phosphate.

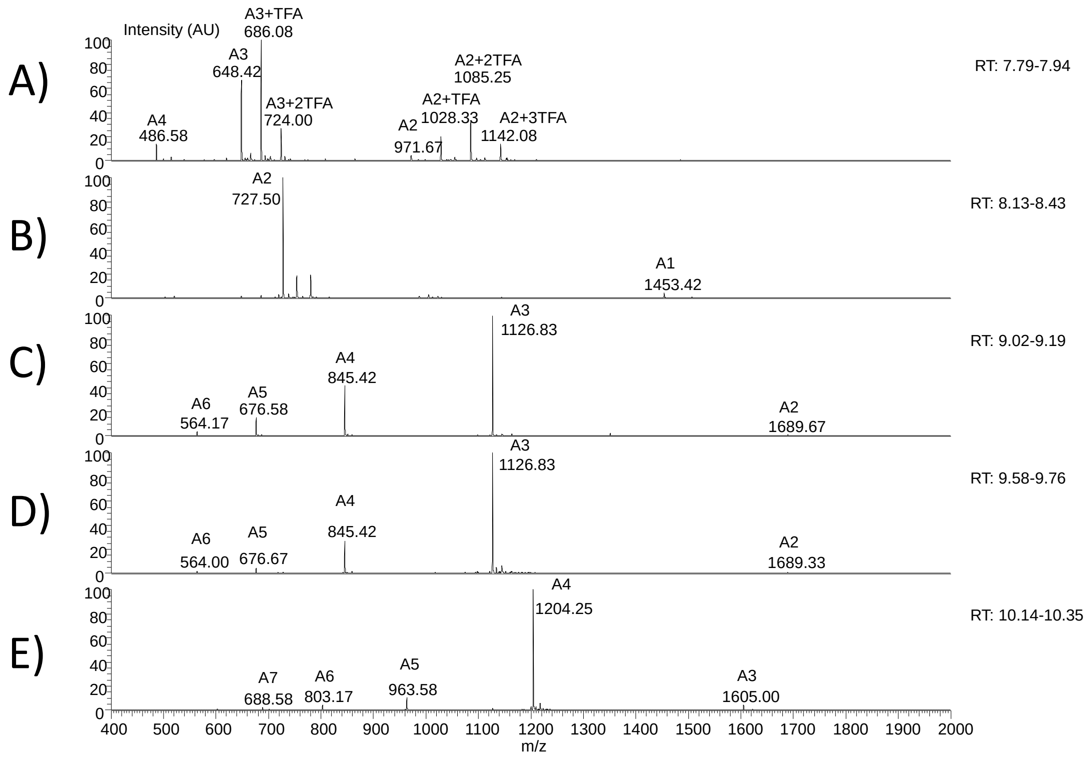

Supplementary Figure 175. MS traces from UPLC-MS analysis of the reaction of peptide thioester Ac-ALKEPVHGVpSGpSA-MPAA **1a** with glycyl peptide GRRRRRRALKEPVHGV-NH2 **2a** after 40 h 30 min in the presence of sodium phosphate buffer (50 mM). A) Rt = 7.73 min, GRRRRRRALKEPVHGV-NH_2 **2a**. MS trace, $[M+2H]^{2+}$ m/z calcd. (av.) 972.16, obs. 971.67, $[M+3H]^{3+}$ m/z calcd. (av.) 648.44, obs. 648.42, $[M+4H]^{4+}$ m/z calcd. (av.) 486.58, obs. 486.58. B) Rt = 8.06 min, peptide thioester hydrolysis byproduct Ac-ALKEPVHGVpSGpSA-OH. $[M+H]^{+}$ m/z calcd. (monoisotopic) 1453.61, obs. 1453.42, $[M+2H]^{2+}$ m/z calcd. (av.) 727.70, obs. 727.50. C) Rt = 8.94 min, Ac-ALKEPVHGVpSGpSAGRRRRRRALKEPVHGV-NH_2 **3a,a**. $[M+2H]^{2+}$ m/z calcd. (av.) 1689.85, obs.1689.67, $[M+3H]^{3+}$ m/z calcd. (av.) 1126.90, obs. 1126.83, $[M+4H]^{4+}$ m/z calcd. (av.) 845.43, obs. 845.42, $[M+5H]^{5+}$ m/z calcd. (av.) 676.54, obs. 676.58, $[M+6H]^{6+}$ m/z calcd. (av.) 563.95, obs. 564.17. D) Rt = 9.53 min, branched byproduct GRRRRRRALK(Ac-ALKEPVHGVpSGpSA)EPVHGV-NH_2. $[M+2H]^{2+}$ m/z calcd. (av.) 1689.85, obs.1689.33, $[M+3H]^{3+}$ m/z calcd. (av.) 1126.90, obs. 1126.83, $[M+4H]^{4+}$ m/z calcd. (av.) 845.43, obs. 845.42, $[M+5H]^{5+}$ m/z calcd. (av.) 676.54, obs. 676.67, $[M+6H]^{6+}$ m/z calcd. (av.) 563.95, obs. 564.00. E) Rt = 10.08 min, double acylation byproduct

Ac-ALKEPVHGVpSGpSA
|
Ac-ALKEPVHGVpSGpSA-GRRRRRRALKEPVHGV-NH_2

. $[M+3H]^{3+}$ m/z calcd. (av.) 1605.37, obs.1605.00, $[M+4H]^{4+}$ m/z calcd. (av.) 1204.27, obs. 1204.25, $[M+5H]^{5+}$ m/z calcd. (av.) 963.62, obs. 963.58, $[M+6H]^{6+}$ m/z calcd. (av.) 803.18, obs. 803.17, $[M+7H]^{7+}$ m/z calcd. (av.) 688.58, obs. 688.58.

Effect of increasing concentrations of Gn·HCl (Fig. 3e)

The effect of Gn·HCl additive concentration on the outcome of the aminolysis reaction was studied using peptides **1a** and **2a**.

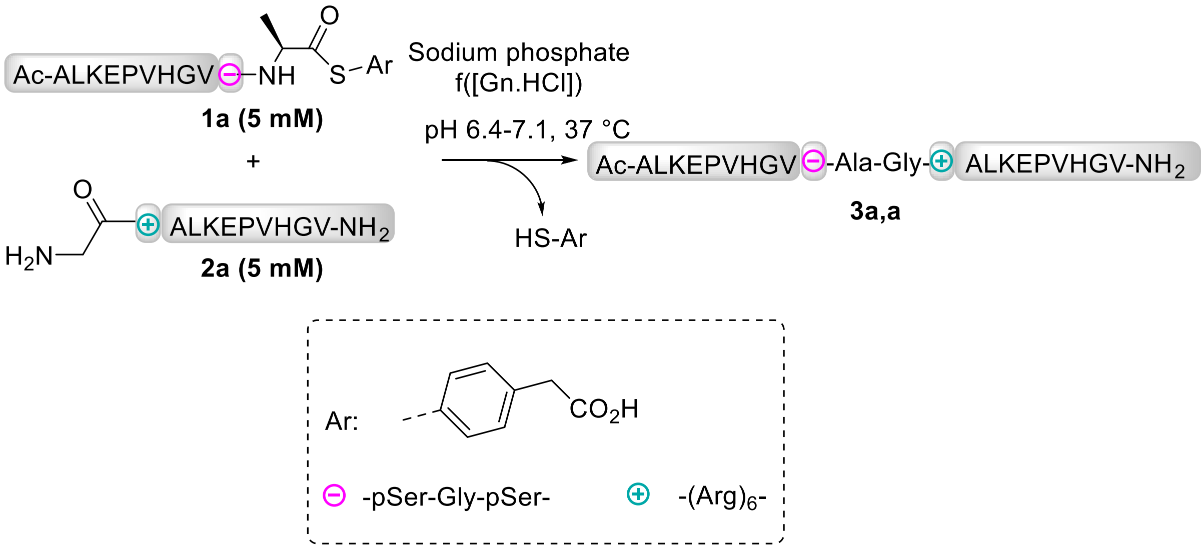

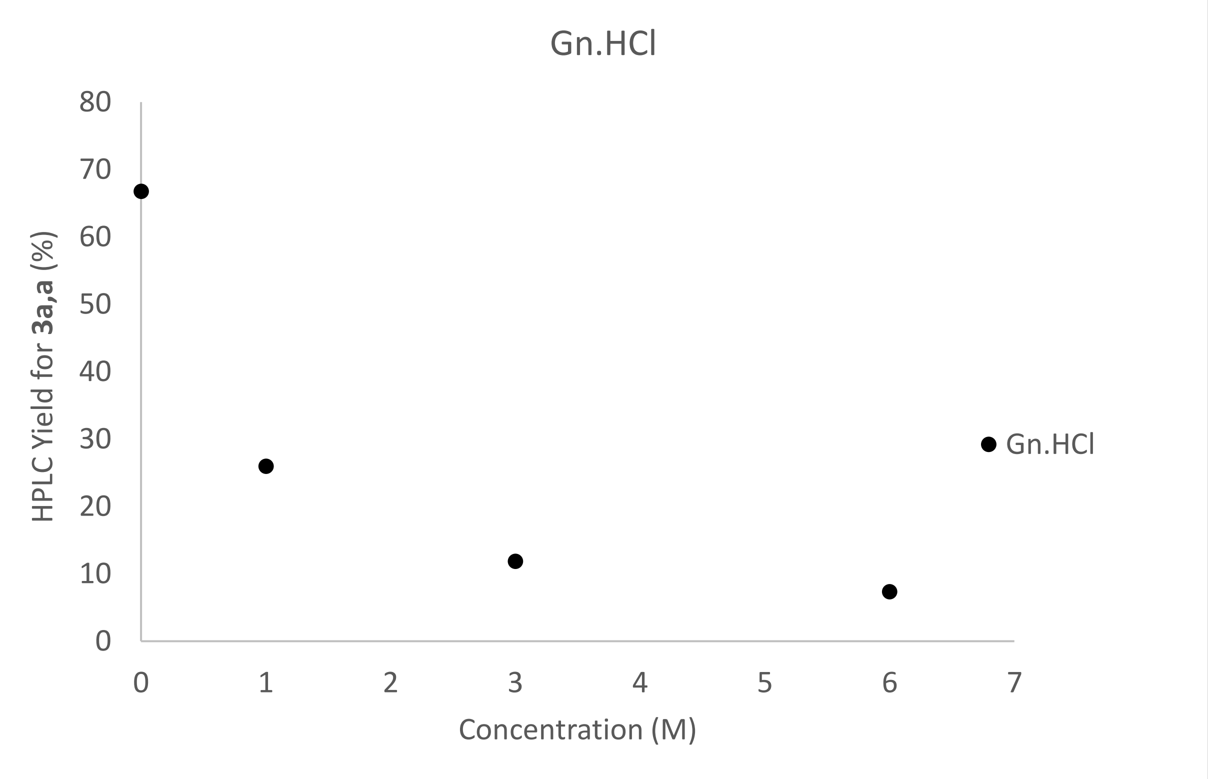

Supplementary Figure 176. Yield of ligation product **3a,a** as a function of increasing concentrations of Gn.HCl.

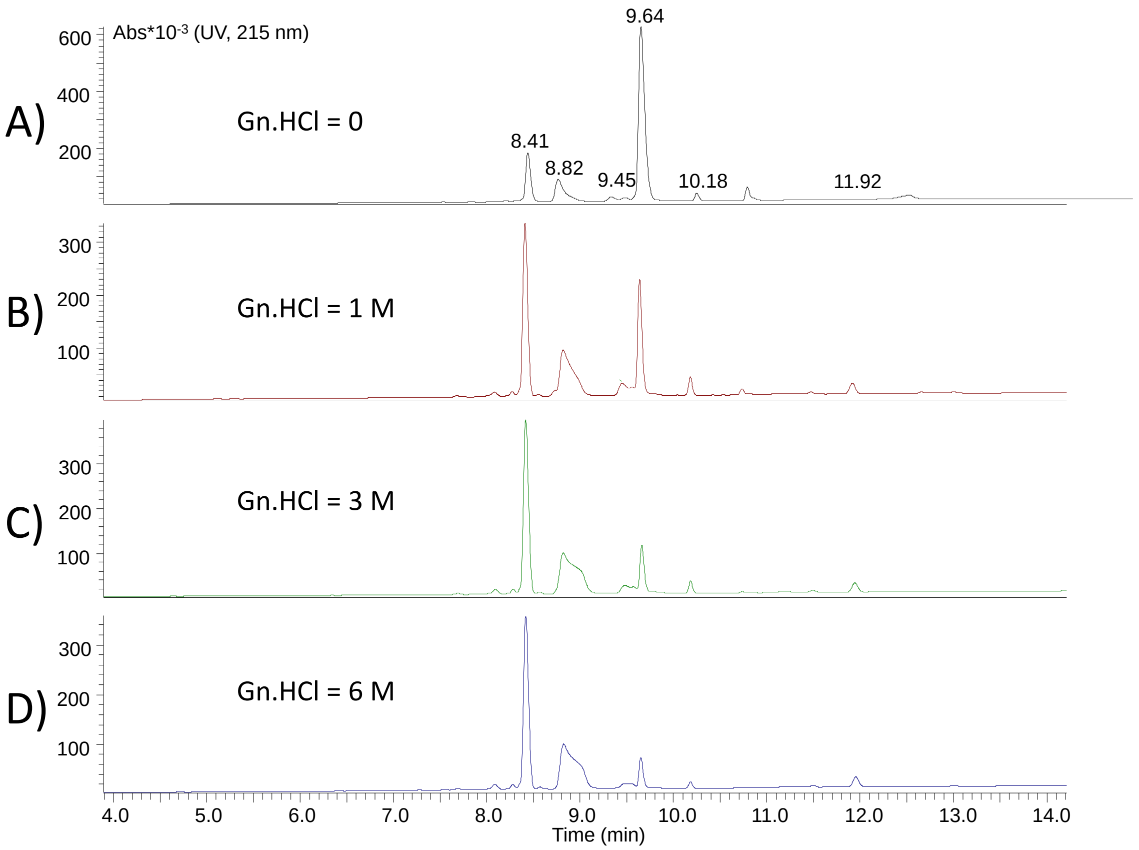

Supplementary Figure 177. UPLC-MS analysis of the reaction of peptide thioester Ac-ALKEPVHGVpSGpSA-MPAA **1a** with glycyl peptide GRRRRRRALKEPVHGV-NH_2 **2a** after 17 h in presence of increasing concentrations of Gn·HCl. LC trace. Eluent A 0.1% TFA in water, eluent B 0.1% TFA in CH_3CN. C18 BEH 300 Å (1.7 µm, 2.1 × 100 mm) column, gradient 0-40% B in 15 min, 0.4 mL min^{-1}, detection at 215 nm). A) w/o Gn·HCl. B) 1 M Gn·HCl. C) 3 M Gn·HCl. D) 6 M Gn·HCl.

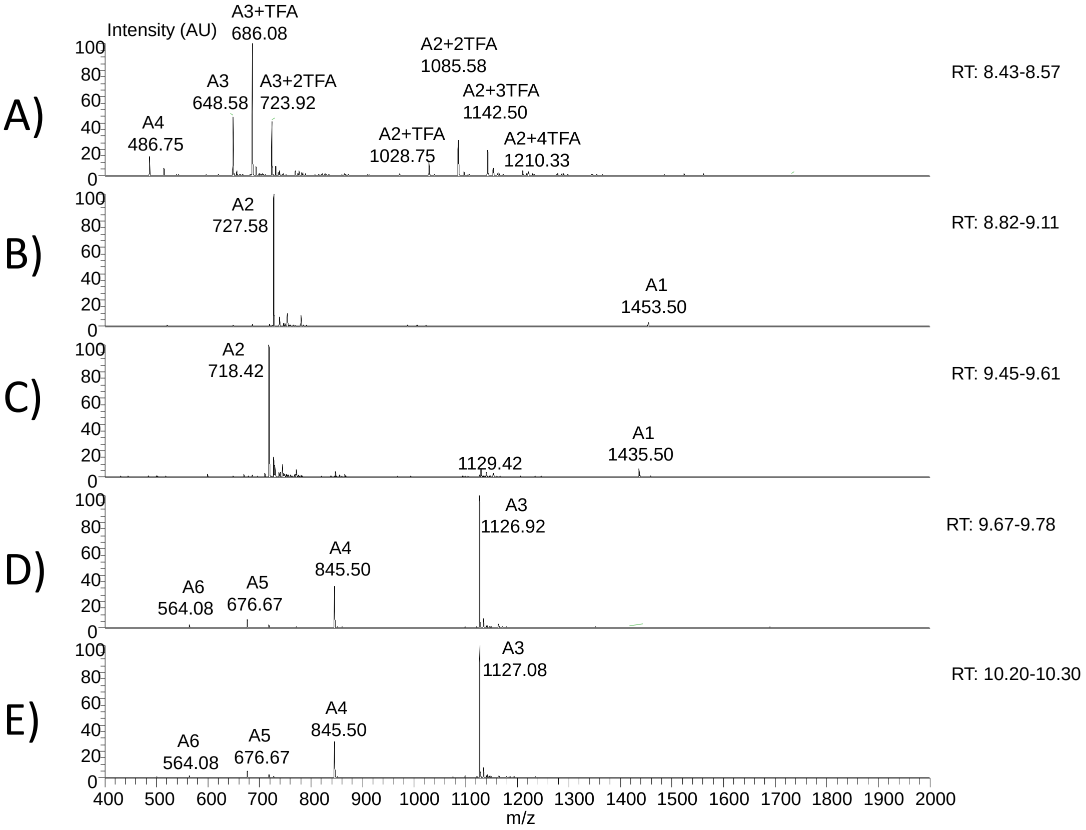

Supplementary Figure 178. MS traces from UPLC-MS analysis of the reaction of peptide thioester Ac-ALKEPVHGVpSGpSA-MPAA **1a** with glycyl peptide GRRRRRRALKEPVHGV-NH2 **2a** after 17 h in presence of 1 M Gn·HCl. A) Rt = 8.41 min, GRRRRRRALKEPVHGV-NH_2 **2a**. $[M+2H+TFA]^{2+}$ m/z calcd. (av.) 1029.16, obs. 1028.75, $[M+3H]^{3+}$ m/z calcd. (av.) 648.44, obs. 648.58, $[M+4H]^{4+}$ m/z calcd. (av.) 486.58, obs. 486.75. B) Rt = 8.82 min, peptide thioester hydrolysis byproduct Ac-ALKEPVHGVpSGpSA-OH. $[M+H]^{+}$ m/z calcd. (monoisotopic) 1453.60, obs. 1453.50, $[M+2H]^{2+}$ m/z calcd. (av.) 727.70, obs. 727.58. C) Rt = 9.45 min, peptide thioester cyclized byproduct. MS trace, $[M+H]^{+}$ m/z calcd. (monoisotopic) 1435.59, obs. 1435.50, $[M+2H]^{2+}$ m/z calcd. (av.) 718.69, obs. 718.42. D) Rt = 9.64 min, target ligation product Ac-ALKEPVHGVpSGpSAGRRRRRRALKEPVHGV-NH_2 **3a,a**. $[M+3H]^{3+}$ m/z calcd. (av.) 1126.90, obs. 1126.92, $[M+4H]^{4+}$ m/z calcd. (av.) 845.43, obs. 845.5, $[M+5H]^{5+}$ m/z calcd. (av.) 676.54, obs. 676.67, $[M+6H]^{6+}$ m/z calcd. (av.) 563.95, obs. 564.08. E) Rt = 10.18 min, branched byproduct GRRRRRRALK(Ac-ALKEPVHGVpSGpSA)EPVHGV-NH_2. $[M+3H]^{3+}$ m/z calcd. (av.) 1126.90, obs. 1127.08, $[M+4H]^{4+}$ m/z calcd. (av.) 845.43, obs. 845.5, $[M+5H]^{5+}$ m/z calcd. (av.) 676.54, obs. 676.67, $[M+6H]^{6+}$ m/z calcd. (av.) 563.95, obs. 564.08.

Effect of increasing concentrations of urea (Fig. 3e)

The effect of urea additive concentration on the outcome of the aminolysis reaction was studied using peptides **1a** and **2a**.

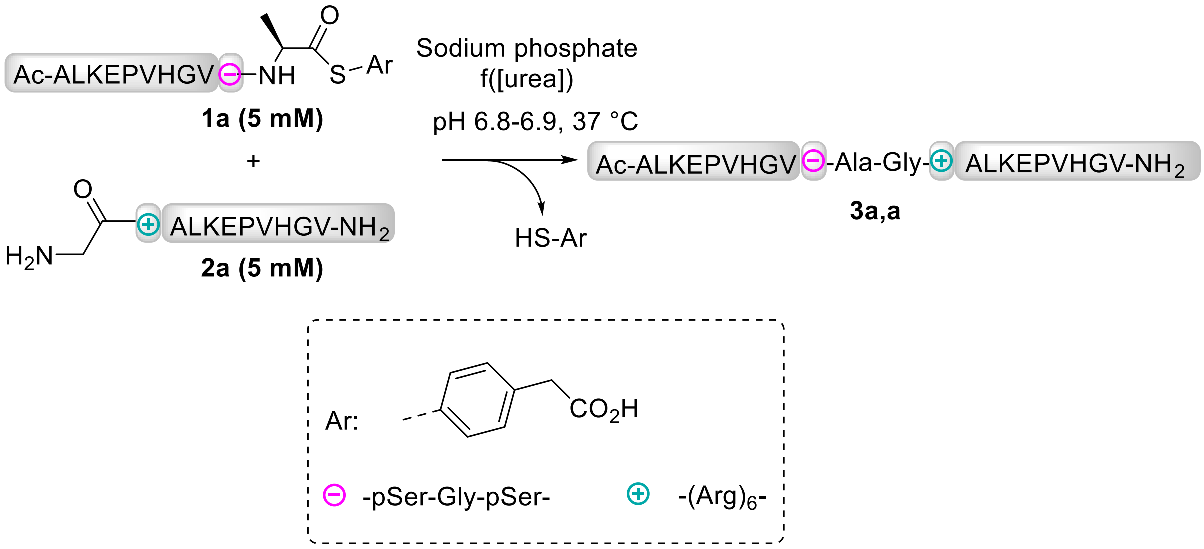

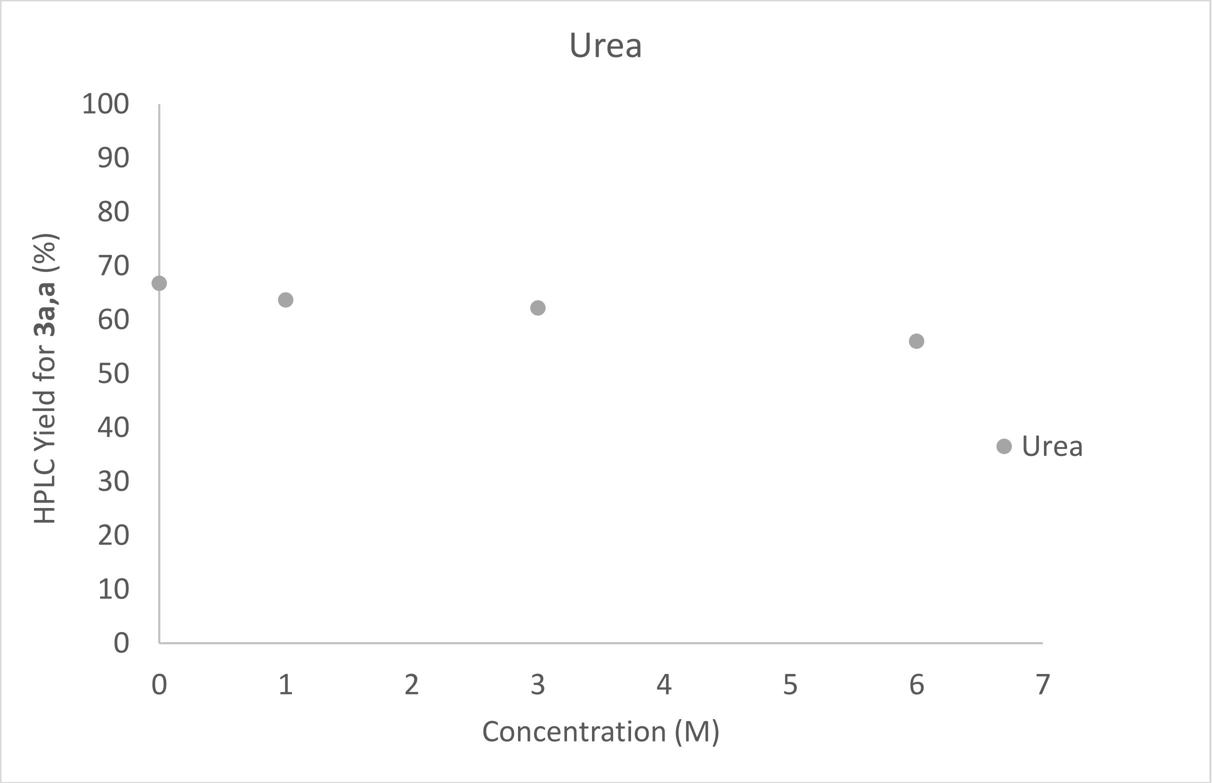

Supplementary Figure 179. Yield of ligation product **3a,a** as a function of increasing concentrations of urea.

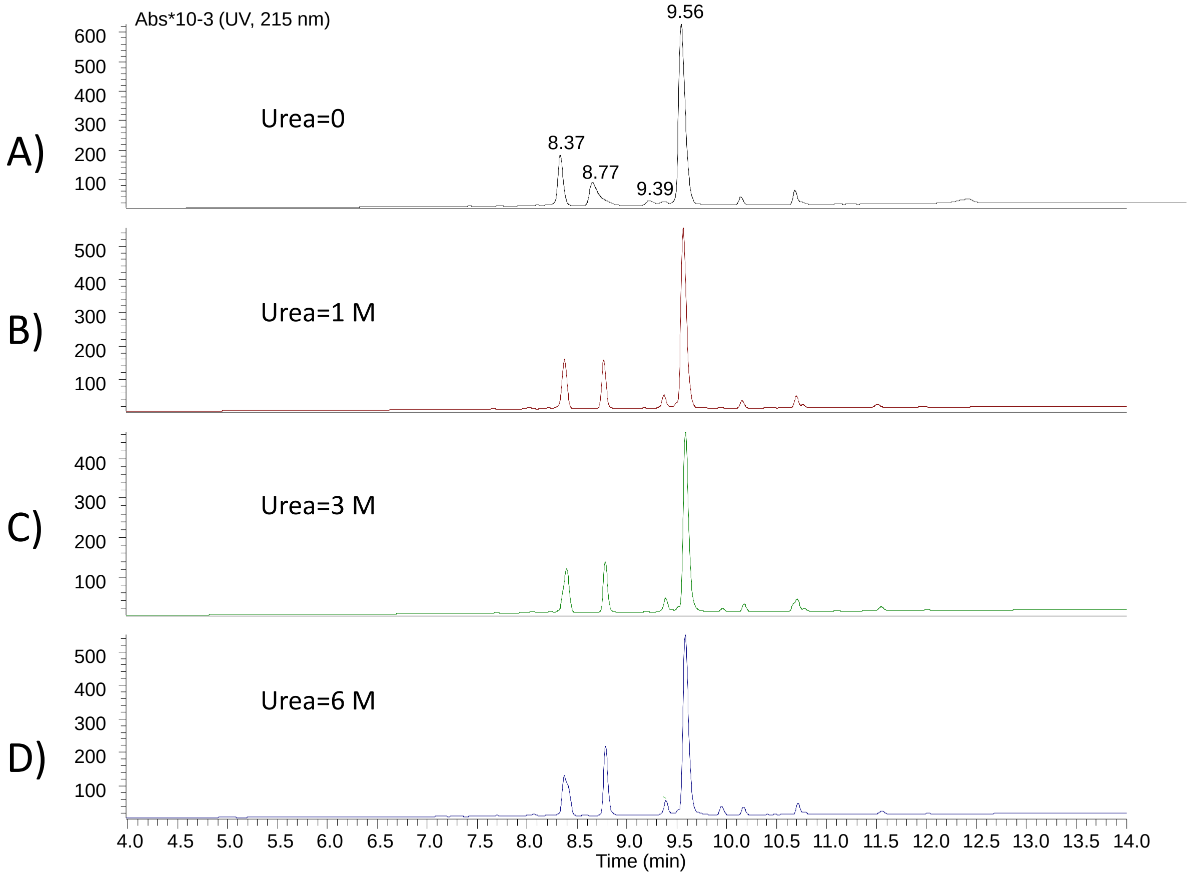

Supplementary Figure 180. UPLC-MS analysis of the reaction of peptide thioester Ac-ALKEPVHGVpSGpSA-MPAA **1a** with glycyl peptide GRRRRRRALKEPVHGV-NH_2 **2a** after 17 h 50 in presence of increasing concentrations of urea. LC trace. Eluent A 0.1% TFA in water, eluent B 0.1% TFA in CH_3CN. C18 BEH 300 Å (1.7 µm, 2.1 × 100 mm) column, gradient 0-40% B in 15 min, 0.4 mL min^{-1}, detection at 215 nm). A) w/o urea. B) 1 M urea. C) 3 M urea. D) 6 M urea.

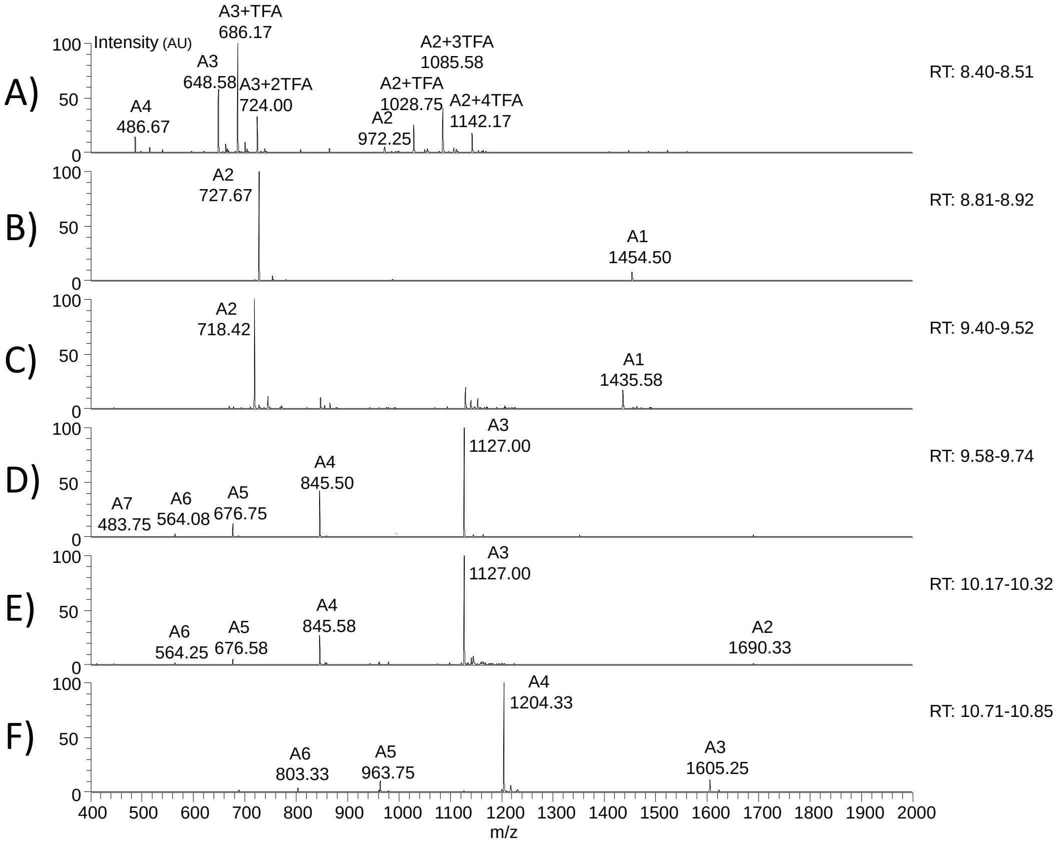

Supplementary Figure 181. MS traces from UPLC-MS analysis of the reaction of peptide thioester Ac-ALKEPVHGVpSGpSA-MPAA **1a** with glycyl peptide GRRRRRRALKEPVHGV-NH2 **2a** after 17 h 50 in presence of 1 M urea. A) Rt = 8.37 min, GRRRRRRALKEPVHGV-NH_2 **2a**. $[M+2H]^{2+}$ m/z calcd. (av.) 972.16, obs. 972.25, $[M+3H]^{3+}$ m/z calcd. (av.) 648.44, obs. 648.58, $[M+4H]^{4+}$ m/z calcd. (av.) 486.58, obs. 486.67. B) Rt = 8.77 min, peptide thioester hydrolysis byproduct Ac-ALKEPVHGVpSGpSA-OH. $[M+H]^{+}$ m/z calcd. (av.) 1454.40, obs. 1454.50, $[M+2H]^{2+}$ m/z calcd. (av.) 727.70, obs. 727.67. C) Rt = 9.39 min, peptide thioester cyclized byproduct. $[M+H]^{+}$ m/z calcd. (monoisotopic) 1435.60, obs. 1435.58, $[M+2H]^{2+}$ m/z calcd. (av.) 718.69, obs. 718.42. D) Rt = 9.56 min, target ligation product Ac-ALKEPVHGVpSGpSAGRRRRRRALKEPVHGV-NH_2 **3a,a**. $[M+3H]^{3+}$ m/z calcd. (av.) 1126.90, obs. 1127.00, $[M+4H]^{4+}$ m/z calcd. (av.) 845.43, obs. 845.5, $[M+5H]^{5+}$ m/z calcd. (av.) 676.54, obs. 676.75, $[M+6H]^{6+}$ m/z calcd. (av.) 563.95, obs. 564.08, $[M+7H]^{7+}$ m/z calcd. (av.) 483.53, obs. 483.75. E) Rt = 10.17 min, branched byproduct GRRRRRRALK(Ac-ALKEPVHGVpSGpSA)EPVHGV-NH_2. $[M+2H]^{2+}$ m/z calcd. (av.) 1689.85, obs. 1690.33, $[M+3H]^{3+}$ m/z calcd. (av.) 1126.90, obs. 1127.00, $[M+4H]^{4+}$ m/z calcd. (av.) 845.43, obs. 845.58, $[M+5H]^{5+}$ m/z calcd. (av.) 676.54, obs. 676.58, $[M+6H]^{6+}$ m/z calcd. (av.) 563.95, obs. 564.25. F) Rt = 10.71 min, double acylated byproduct

Ac-ALKEPVHGVpSGpSA
|
Ac-ALKEPVHGVpSGpSA-GRRRRRRALKEPVHGV-NH_2

. $[M+3H]^{3+}$ m/z calcd. (av.) 1605.37, obs. 1605.25, $[M+4H]^{4+}$ m/z calcd. (av.) 1204.27, obs. 1204.33, $[M+5H]^{5+}$ m/z calcd. (av.) 963.62, obs. 963.75, $[M+6H]^{6+}$ m/z calcd. (av.) 803.18, obs. 803.33.

Effect of TCEP

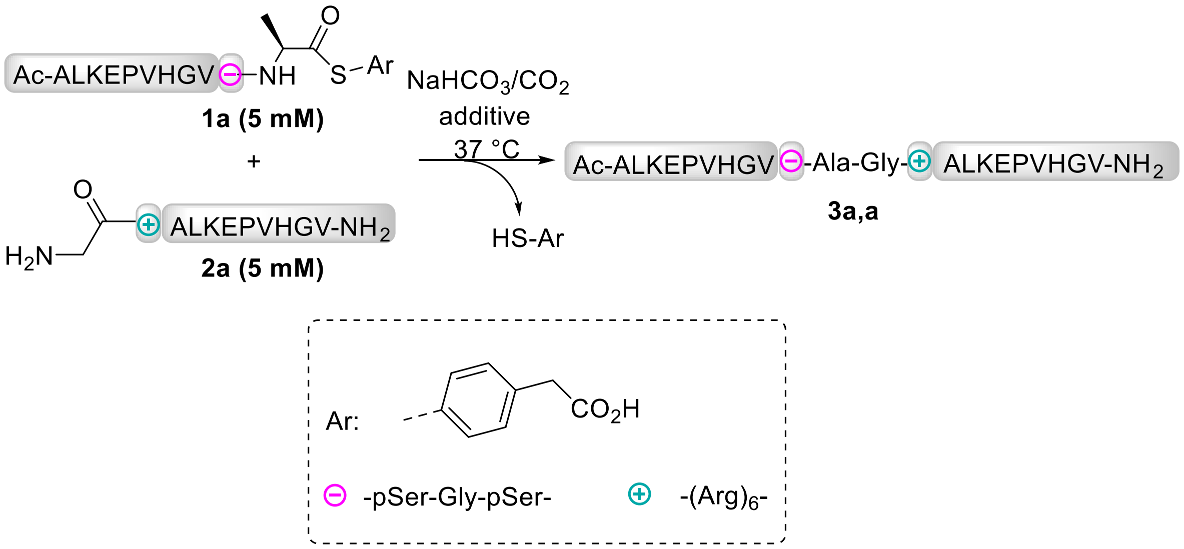

Effect of adding TCEP: TCEP·HCl (273 µg) in water (952 µL, 1 mM final concentration) was first neutralized with NaOH 6 M until pH=5.44 and then lyophilized. The powder was then dissolved in 50 mM sodium bicarbonate/CO_2 buffer (952 µL, 1 mM TCEP concentration). This solution was used to perform the aminolysis reaction as described before.

The data show that TCEP (1 mM) has no significant effect on the aminolysis reaction.

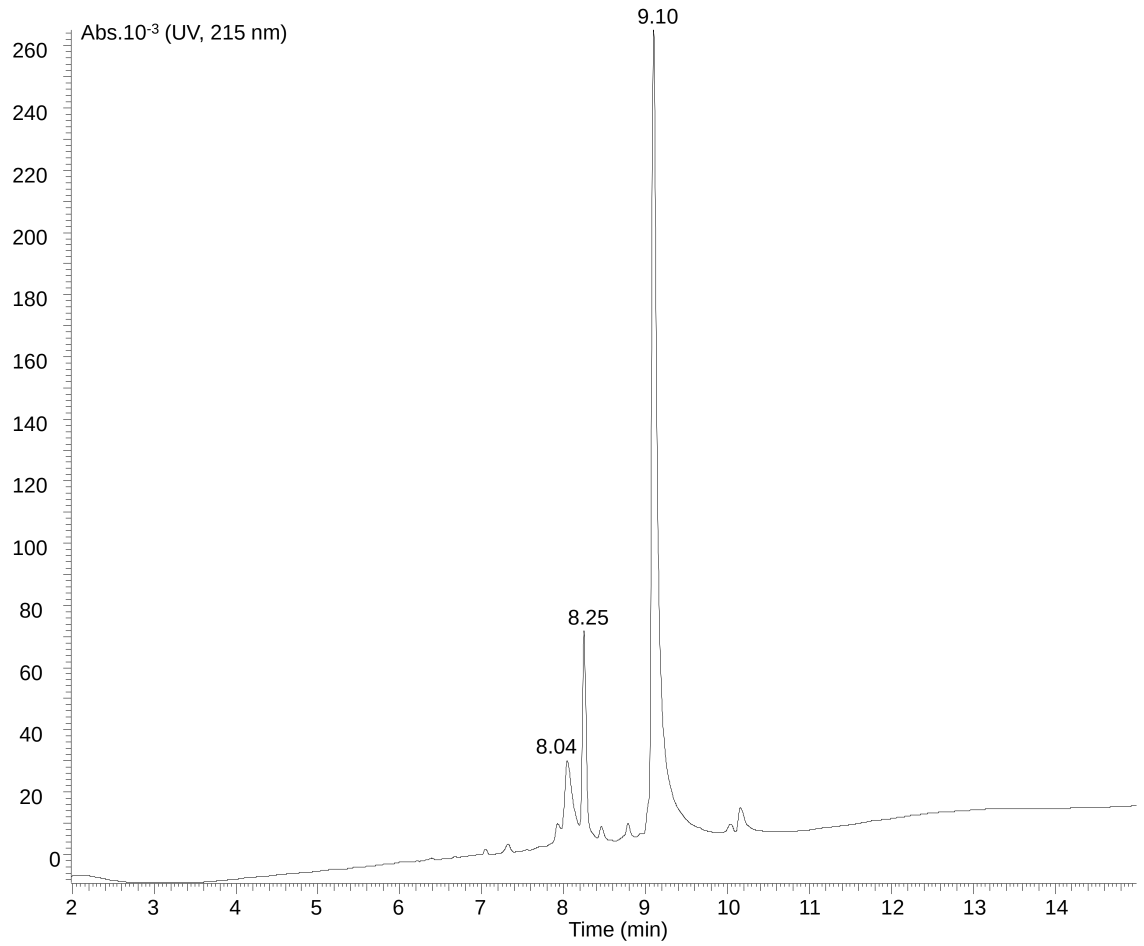

Supplementary Figure 182. UPLC-MS analysis of the reaction of peptide thioester **1a** with glycyl peptide **2a** in the presence of 1 mM TCEP after 22 h. LC trace. Eluent A 0.1% TFA in water, eluent B 0.1% TFA in CH_3CN. C18 BEH 300 Å (1.7 µm, 2.1 × 100 mm) column, gradient 0-40% B in 15 min, 0.4 mL min^{-1}, detection at 215 nm).

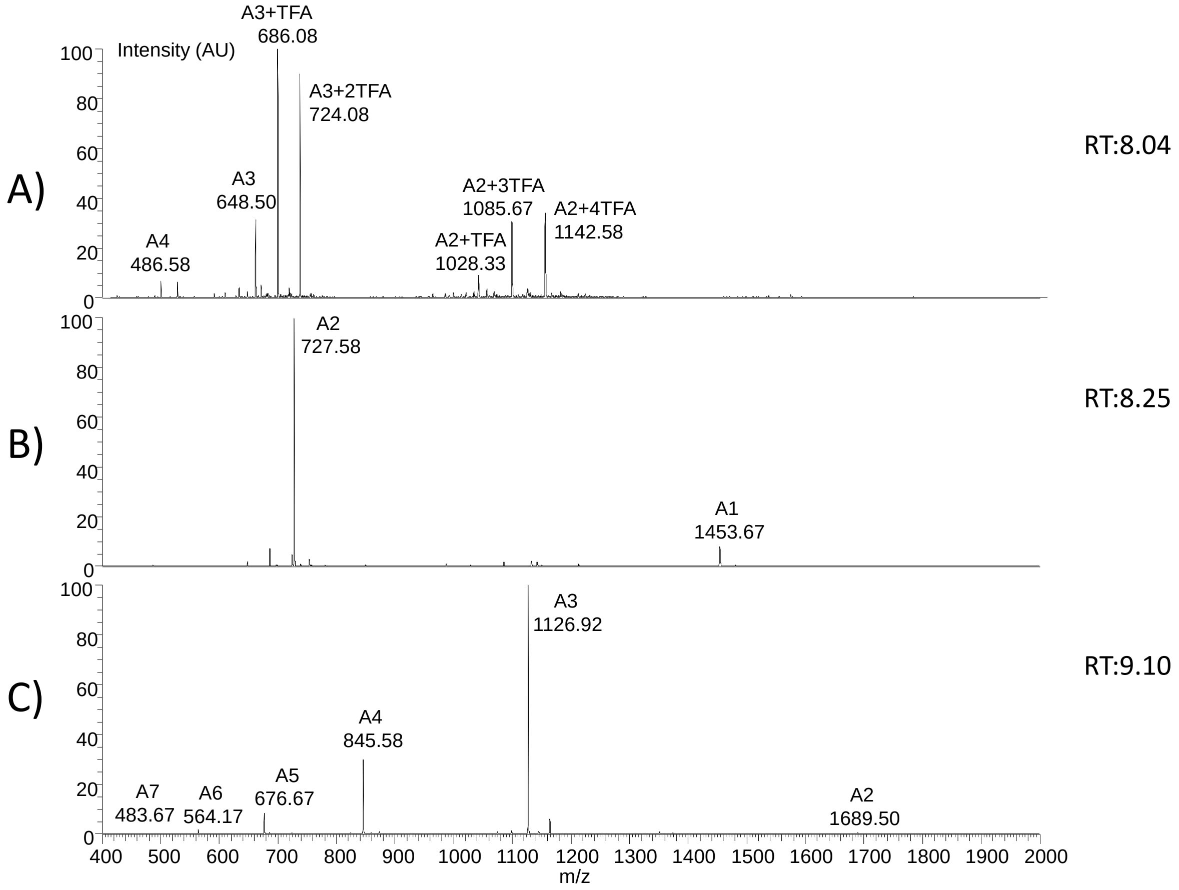

Supplementary Figure 183. MS traces from UPLC-MS analysis of the reaction of peptide thioester **1a** with glycyl peptide **2a** in the presence of 1 mM TCEP after 22 h. A) Rt = 8.04 min, GRRRRRRALKEPVHGV-NH_2 **2a**. $[M+3H]^{3+}$ m/z calcd. (av.) 648.44, obs. 648.50, $[M+4H]^{4+}$ m/z calcd. (av.) 486.58, obs. 486.58. B) Rt = 8.25 min, peptide thioester hydrolysis byproduct Ac-ALKEPVHGVpSGpSA-OH. $[M+H]^{+}$ m/z calcd. (monoisotopic) 1453.60, obs. 1453.67, $[M+2H]^{2+}$ m/z calcd. (av.) 727.70, obs. 727.58. C) Rt = 9.10 min, target ligation product Ac-ALKEPVHGVpSGpSAGRRRRRRALKEPVHGV-NH_2 **3a,a**. $[M+2H]^{2+}$ m/z calcd. (av.) 1689.85, obs. 1689.50, $[M+3H]^{3+}$ m/z calcd. (av.) 1126.90, obs. 1126.92, $[M+4H]^{4+}$ m/z calcd. (av.) 845.43, obs. 845.58, $[M+5H]^{5+}$ m/z calcd. (av.) 676.54, obs. 676.67, $[M+6H]^{6+}$ m/z calcd. (av.) 563.95, obs. 564.17, $[M+7H]^{7+}$ m/z calcd. (av.) 483.53, obs. 483.67.

Effect of *n*-octylglucoside

Effect of adding *n*-octylglucoside: *n*-Octylglucoside (300 µg, 10 mM final concentration) was dissolved in 50 mM sodium bicarbonate/CO_2 buffer (102.6 µL). This solution was used to perform the aminolysis reaction as described in section 6.1.

The data show that *n*-octylglucoside (10 mM) has no significant effect on the aminolysis reaction.

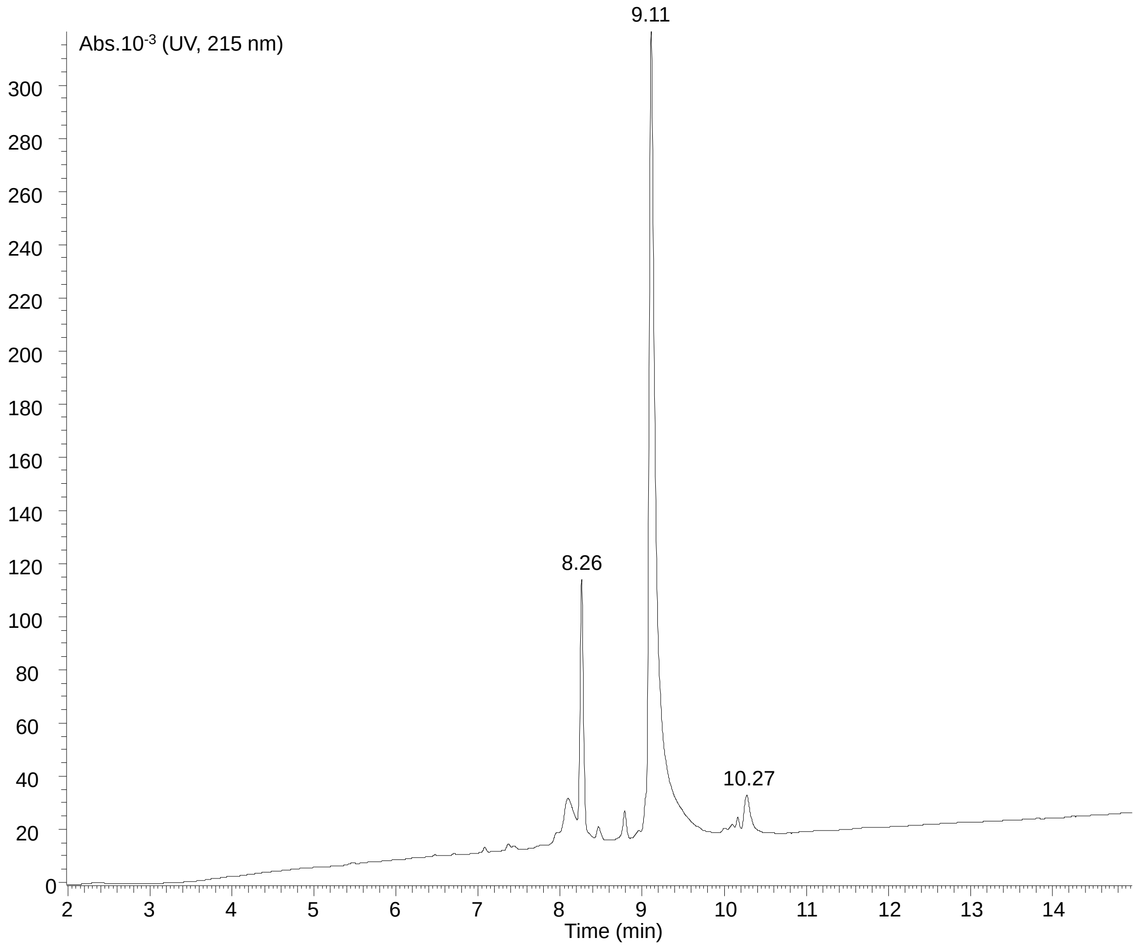

Supplementary Figure 184. UPLC-MS analysis of the reaction of peptide thioester **1a** with glycyl peptide **2a** in the presence of 10 mM *n*-octylglucoside after 18 h. LC trace. Eluent A 0.1% TFA in water, eluent B 0.1% TFA in CH_3CN. C18 BEH 300 Å (1.7 µm, 2.1 × 100 mm) column, gradient 0-40% B in 15 min, 0.4 mL min^{-1}, detection at 215 nm).

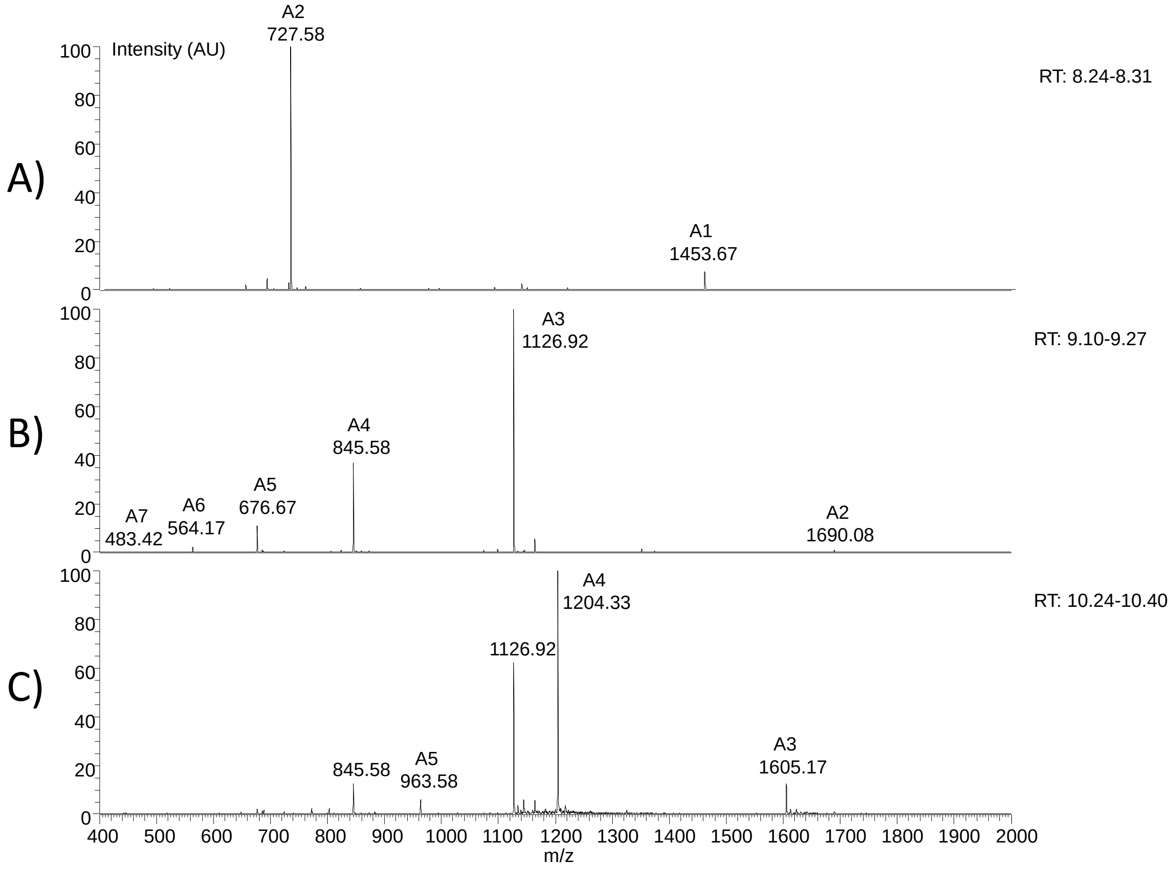

Supplementary Figure 185. MS traces from LC-MS analysis of the reaction of peptide thioester **1a** with glycyl peptide **2a** in the presence of 10 mM *n*-octylglucoside after 18 h. A) Rt = 8.26 min, peptide thioester hydrolysis byproduct Ac-ALKEPVHGVpSGpSA-OH. $[M+H]^{+}$ m/z calcd. (monoisotopic) 1453.60, obs. 1453.67, $[M+2H]^{2+}$ m/z calcd. (av.) 727.70, obs. 727.58. B) Rt = 9.11 min, target ligation product Ac-ALKEPVHGVpSGpSAGRRRRRRALKEPVHGV-NH_2 **3a,a**. $[M+2H]^{2+}$ m/z calcd. (av.) 1689.85, obs. 1690.08, $[M+3H]^{3+}$ m/z calcd. (av.) 1126.90, obs. 1126.92, $[M+4H]^{4+}$ m/z calcd. (av.) 845.43, obs. 845.58, $[M+5H]^{5+}$ m/z calcd. (av.) 676.54, obs. 676.67, $[M+6H]^{6+}$ m/z calcd. (av.) 563.95, obs. 564.17, $[M+7H]^{7+}$ m/z calcd. (av.) 483.53, obs. 483.42. C) Rt = 10.27 min, diacylation byproduct

Ac-ALKEPVHGVpSGpSA
|
Ac-ALKEPVHGVpSGpSA-GRRRRRRALKEPVHGV-NH_2

. $[M+3H]^{3+}$ m/z calcd. (av.) 1605.37, obs. 1605.17, $[M+4H]^{4+}$ m/z calcd. (av.) 1204.27, obs. 1204.33, $[M+5H]^{5+}$ m/z calcd. (av.) 963.62, obs. 963.58.

Synthesis of peptide **3a,a** on preparative scale (Fig. 2a)

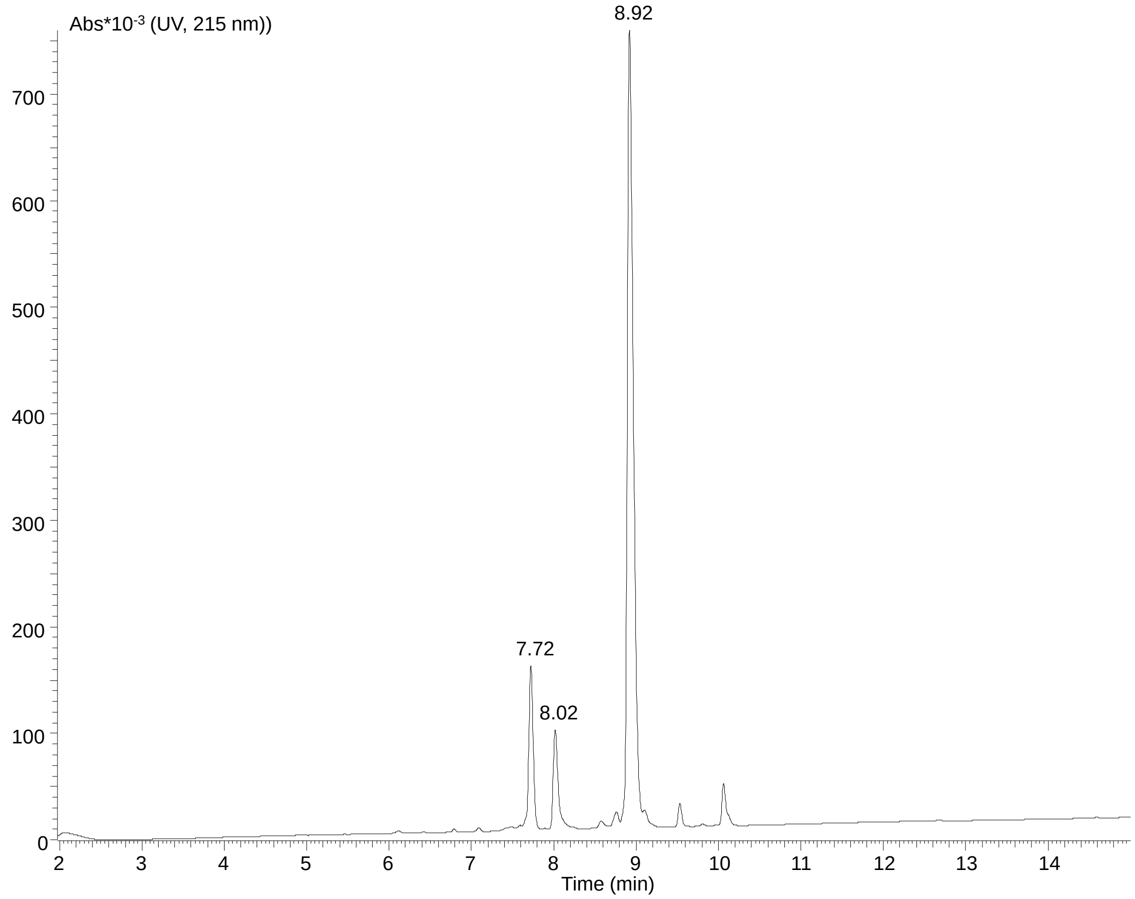

Supplementary Figure 186. UPLC-MS analysis of the crude reaction mixture between peptide thioester Ac-ALKEPVHGVpSGpSA-MPAA **1a** and glycyl peptide GRRRRRRALKEPVHGV-NH_2 **2a** after 24 h. LC trace. Eluent A 0.1% TFA in water, eluent B 0.1% TFA in CH_3CN. C18 BEH 300 Å (1.7 µm, 2.1 × 100 mm) column, gradient 0-40% B in 15 min, 0.4 mL min^{-1}, detection at 215 nm).

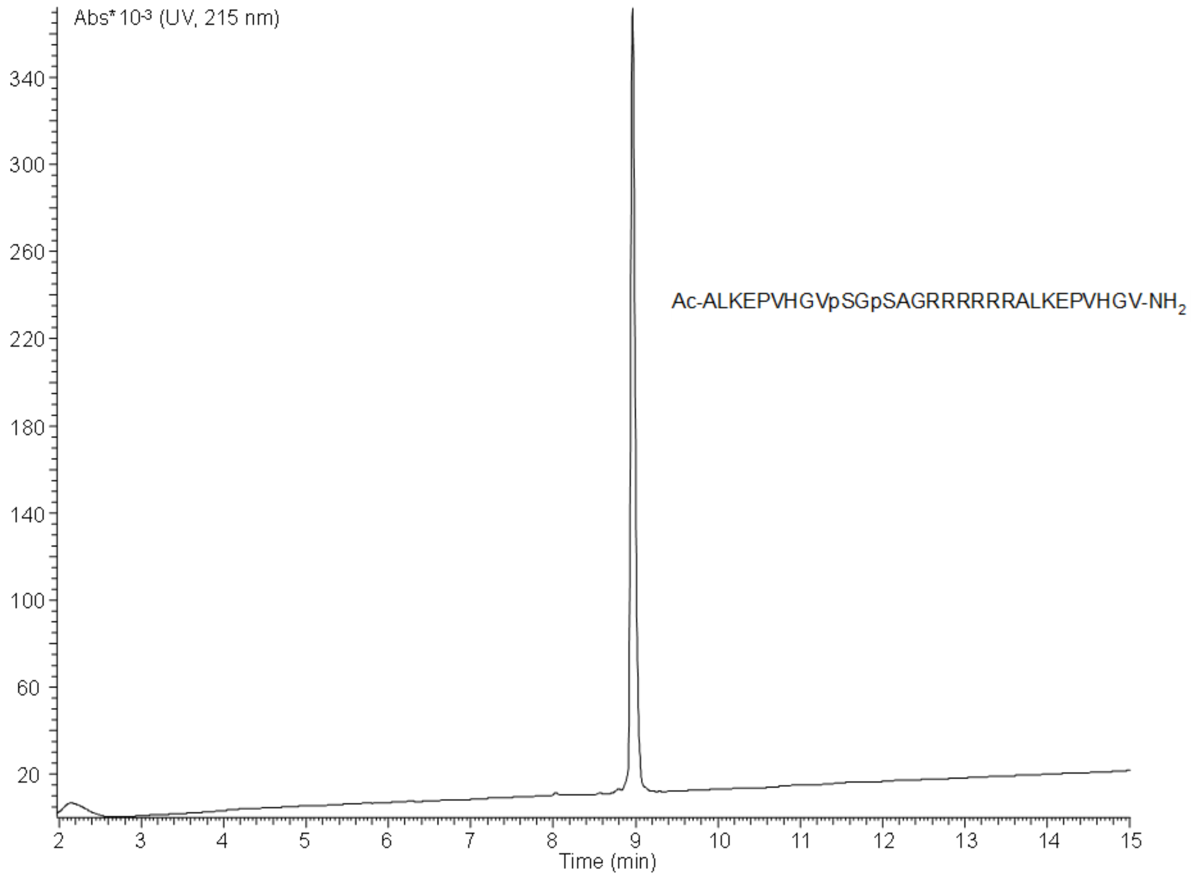

Supplementary Figure 187. UPLC-MS analysis of purified peptide **3a,a**. LC trace. Eluent A 0.1% TFA in water, eluent B 0.1% TFA in CH_3CN. C18 BEH 300 Å (1.7 µm, 2.1 × 100 mm) column, gradient 0-40% B in 15 min, 0.4 mL min^{-1}, detection at 215 nm).

A)

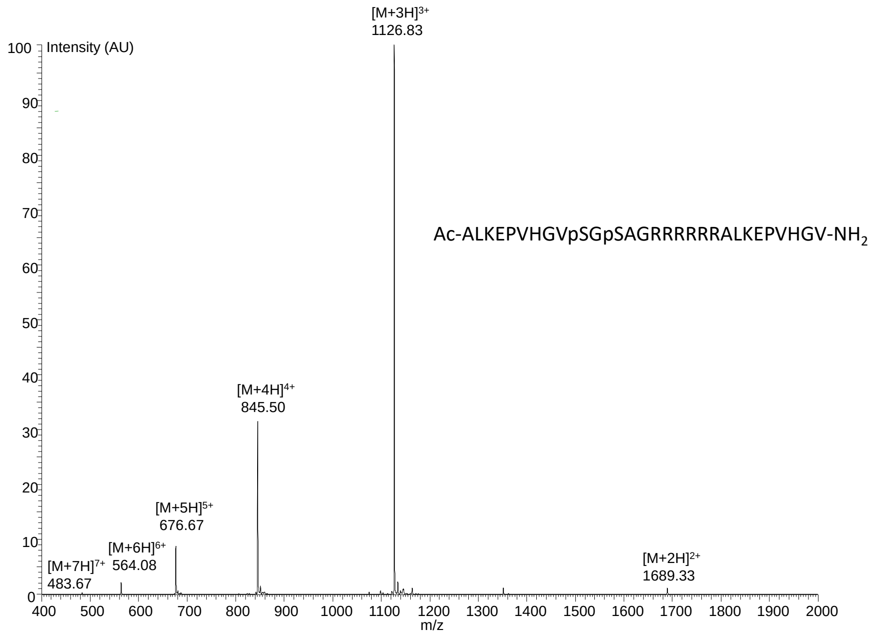

B)

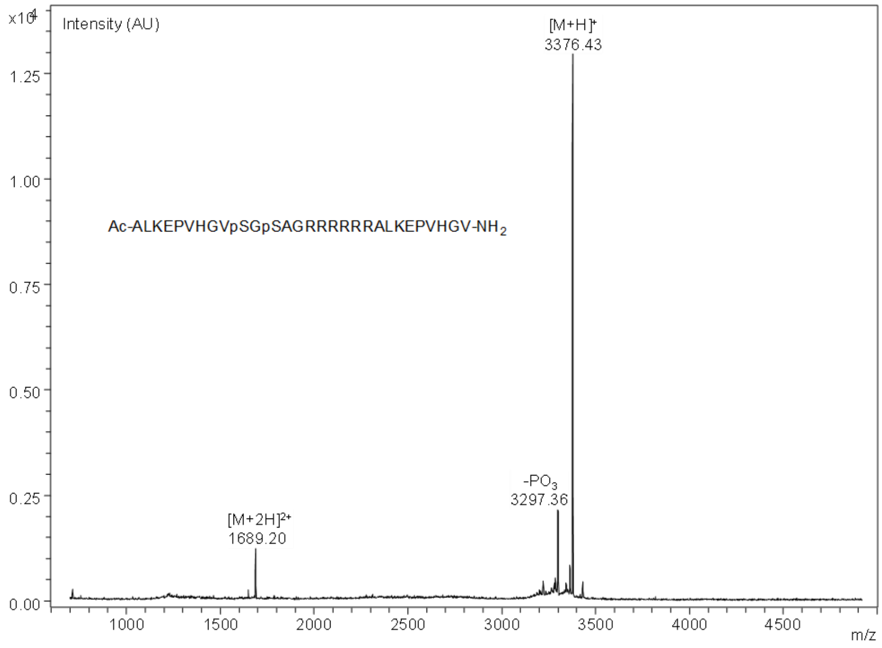

Supplementary Figure 188. Analysis of purified peptide **3a,a** by mass spectrometry. A) MS trace of purified peptide **3a,a** from LC-MS analysis. $[M+2H]^{2+}$ m/z calcd. (av.) 1689.85, obs. 1689.33, $[M+3H]^{3+}$ m/z calcd. (av.) 1126.90, obs. 1126.83, $[M+4H]^{4+}$ m/z calcd. (av.) 845.43, obs. 845.50, $[M+5H]^{5+}$ m/z calcd. (av.) 676.54, obs. 676.67, $[M+6H]^{6+}$ m/z calcd. (av.) 563.95, obs. 564.08, $[M+7H]^{7+}$ m/z calcd. (av.) 483.53, obs. 483.67. B) MALDI-TOF analysis of purified peptide **3a,a**. Matrix alpha-cyano-4-hydroxycinnaminic acid, positive detection mode, $[M+H]^{+}$ m/z calcd. (monoisotopic) 3376.78, found 3376.43, $[M+2H]^{2+}$ m/z calcd. (monoisotopic) 1688.89, found 1689.20.

Comparison with an authentic sample produced by SPPS

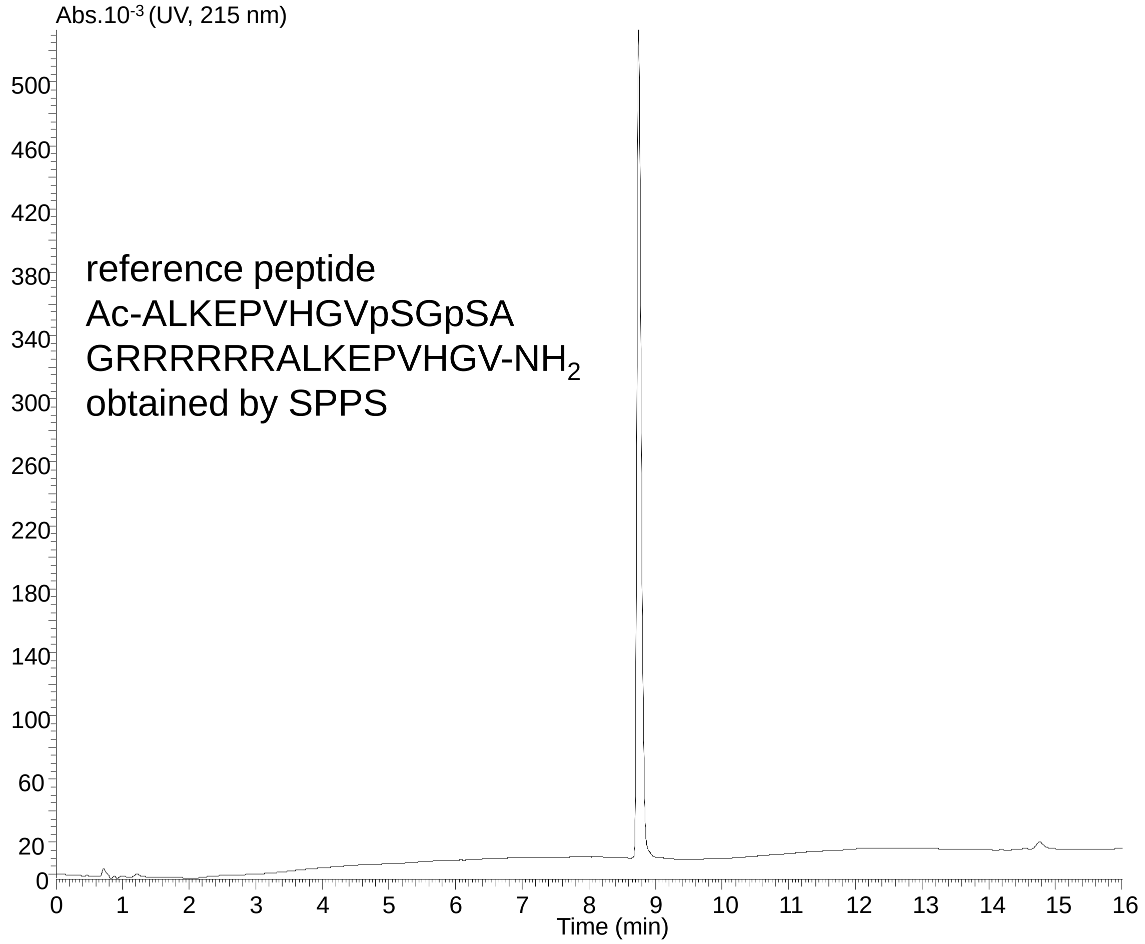

Supplementary Figure 189. UPLC-MS analysis of reference peptide Ac-ALKEPVHGVpSGpSAGRRRRRRALKEPVHGV-NH_2 obtained by SPPS. LC trace. Eluent A 0.1% TFA in water, eluent B 0.1% TFA in CH_3CN. C18 BEH 300 Å (1.7 µm, 2.1 × 100 mm) column, gradient 0-40% B in 15 min, 0.4 mL min^{-1}, detection at 215 nm).

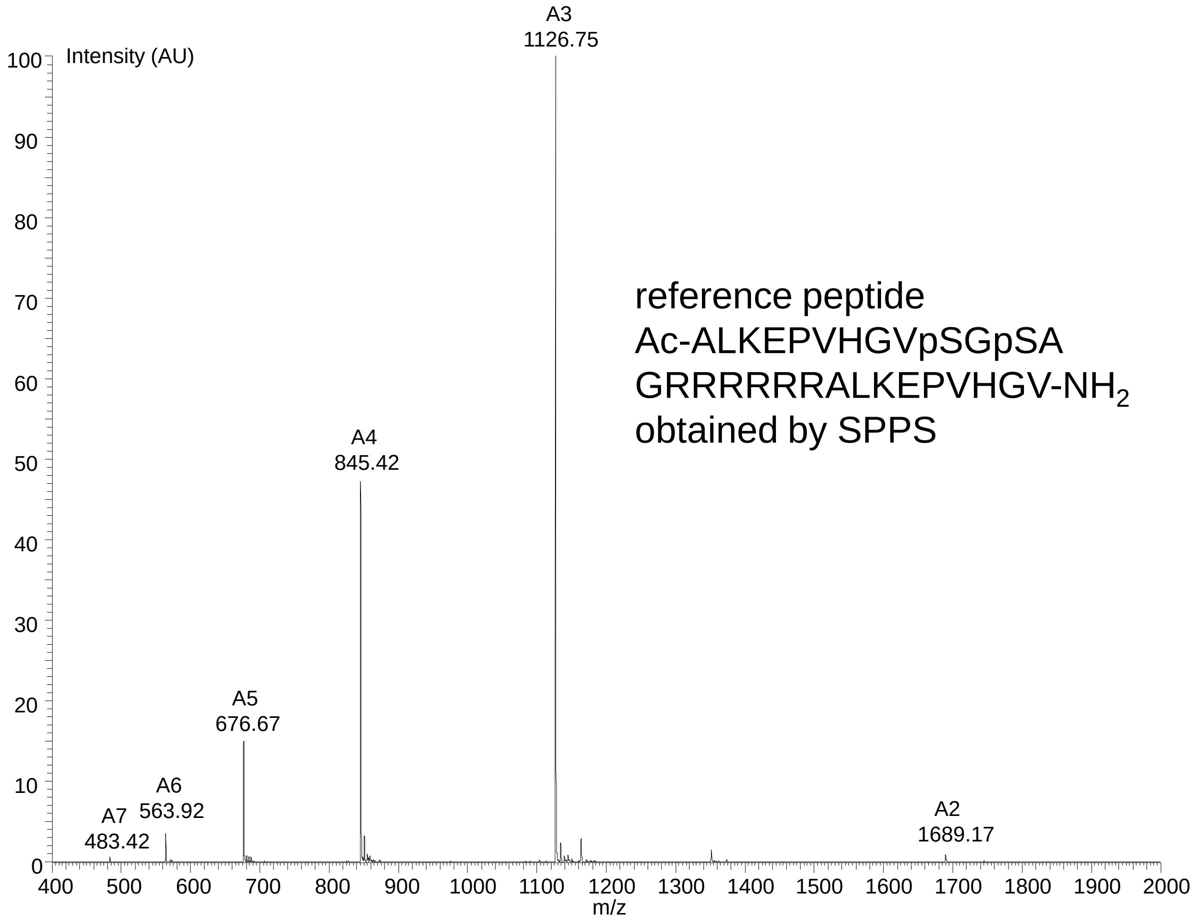

Supplementary Figure 190. MS trace from UPLC-MS analysis of reference peptide Ac-ALKEPVHGVpSGpSAGRRRRRRALKEPVHGV-NH_2 obtained by SPPS. $[M+2H]^{2+}$ m/z calcd. (av.) 1689.85, obs. 1689.17, $[M+3H]^{3+}$ m/z calcd. (av.) 1126.90, obs. 1126.75, $[M+4H]^{4+}$ m/z calcd. (av.) 845.43, obs. 845.42, $[M+5H]^{5+}$ m/z calcd. (av.) 676.54, obs. 676.67, $[M+6H]^{6+}$ m/z calcd. (av.) 563.95, obs. 563.92, $[M+7H]^{7+}$ m/z calcd. (av.) 483.53, obs. 483.42.

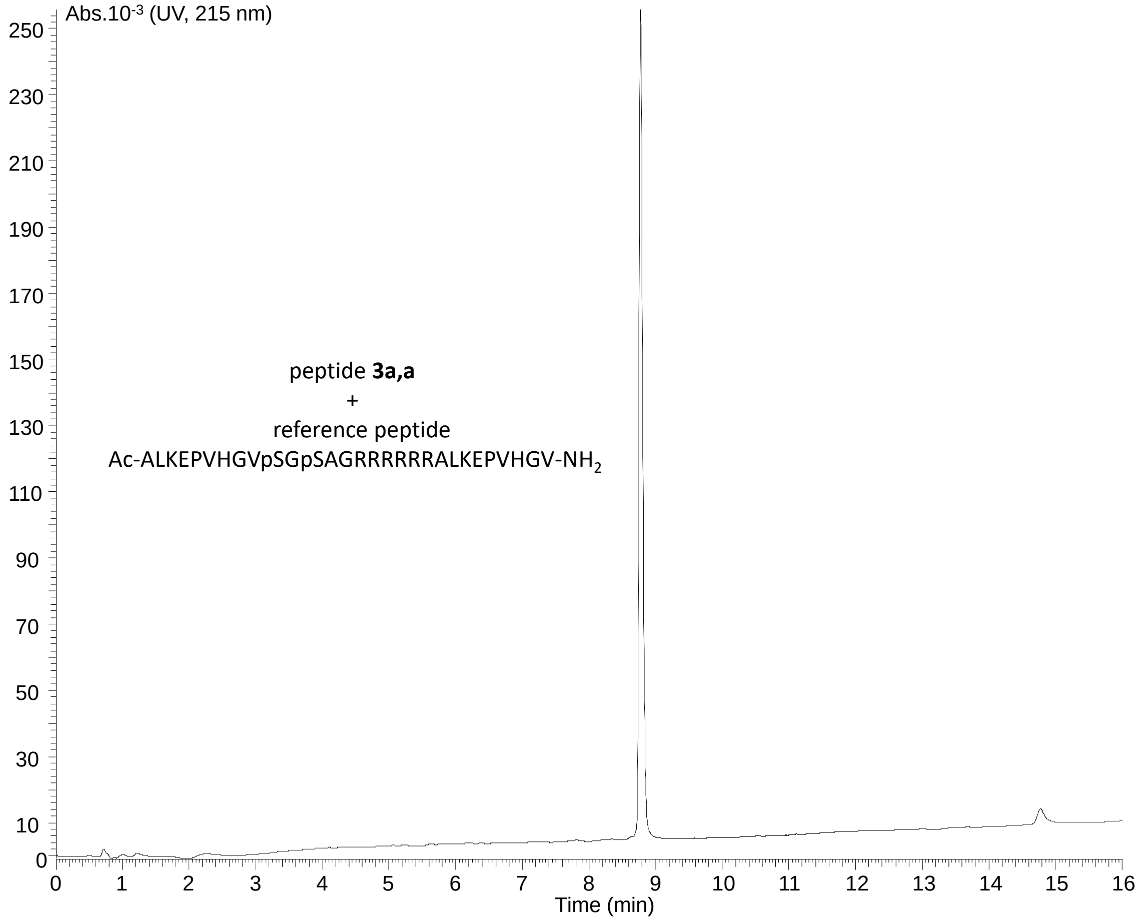

Supplementary Figure 191. UPLC-MS analysis of the mixture of purified peptide **3a,a** with reference peptide Ac-ALKEPVHGVpSGpSAGRRRRRRALKEPVHGV-NH_2 obtained by standard SPPS. LC trace. Eluent A 0.1% TFA in water, eluent B 0.1% TFA in CH_3CN. C18 BEH 300 Å (1.7 µm, 2.1 × 100 mm) column, gradient 0-40% B in 15 min, 0.4 mL min^{-1}, detection at 215 nm).

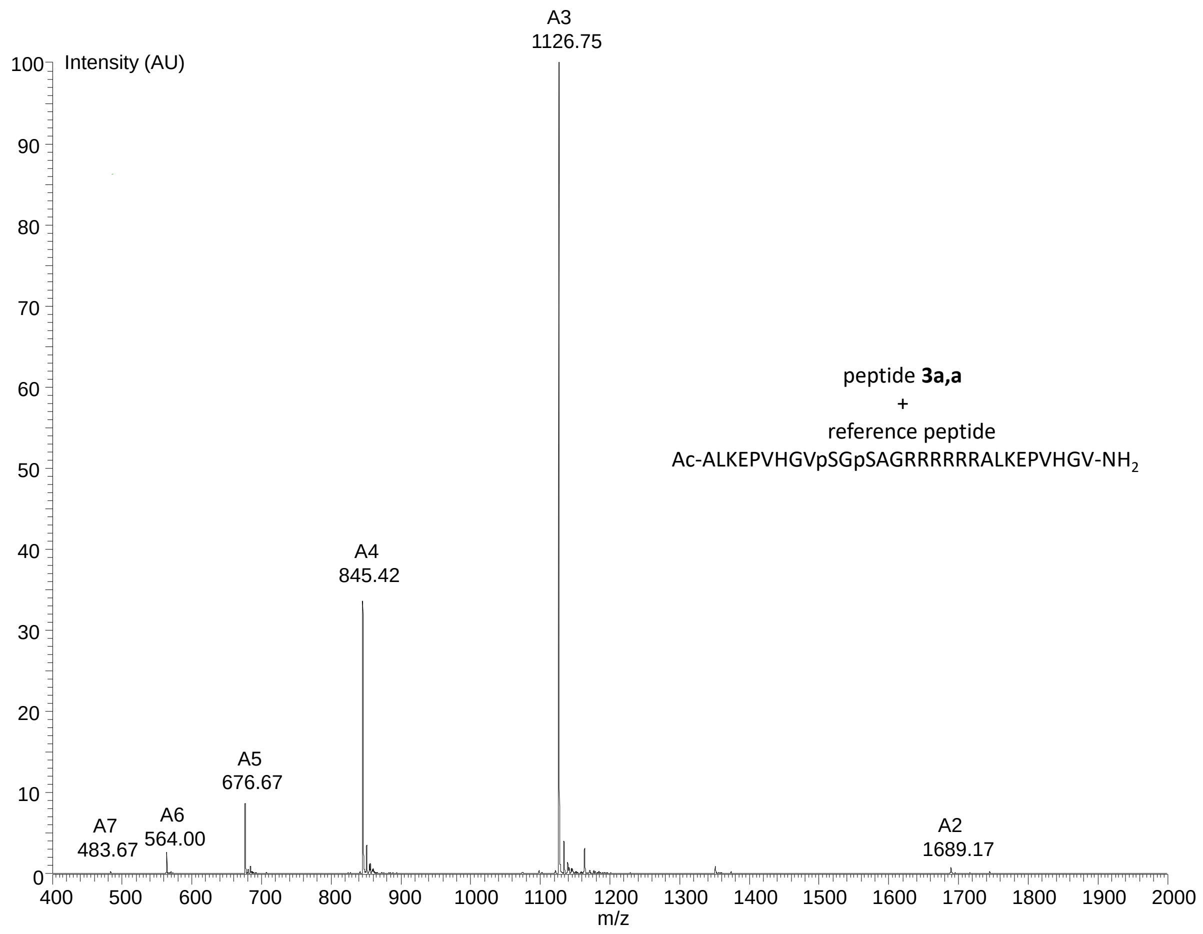

Supplementary Figure 192. MS trace from UPLC-MS analysis of the mixture of purified peptide **3a,a** with reference peptide Ac-ALKEPVHGVpSGpSAGRRRRRRRALKEPVHGV-NH_2 obtained by standard SPPS. $[M+2H]^{2+}$ m/z calcd. (av.) 1689.85, obs. 1689.17, $[M+3H]^{3+}$ m/z calcd. (av.) 1126.90, obs. 1126.75, $[M+4H]^{4+}$ m/z calcd. (av.) 845.43, obs. 845.42, $[M+5H]^{5+}$ m/z calcd. (av.) 676.54, obs. 676.67, $[M+6H]^{6+}$ m/z calcd. (av.) 563.95, obs. 564.00, $[M+7H]^{7+}$ m/z calcd. (av.) 483.53, obs. 483.67.

Peptide thioester aminolysis. Competition experiments

Competition experiment 1: **1a** + **1d** + **2a** →

Ac-ALKEPVHGVSGSA-MPAA **1d** (50 µg, 2.99.10^{-8} mol, 0.3 mM final concentration) was dissolved in 10 mM sodium bicarbonate/CO_2 buffer (96.4 µL). This solution was quickly added to Ac-ALKEPVHGVpSGpSA-MPAA **1a** (53 µg, 2.89.10^{-8} mol, 0.3 mM final concentration). The peptide thioesters were then added to GRRRRRRALKEPVHGV-NH_2 **2a** (86 µg, 2.90.10^{-8} mol, 0.3 mM final concentration). The reaction mixture was placed in a CO_2 incubator for cell biology (5% CO_2 partial pressure, 37 °C, water saturated) with a needle inserted through the cap to allow CO_2 to diffuse into the tube. The final pH of the reaction mixture was 6.96. After 48 h, a sample of the reaction mixture was analyzed by UPLC-MS by diluting 4.5 µL of the reaction mixture with aqueous AcOH (10% by vol, 30 µL).

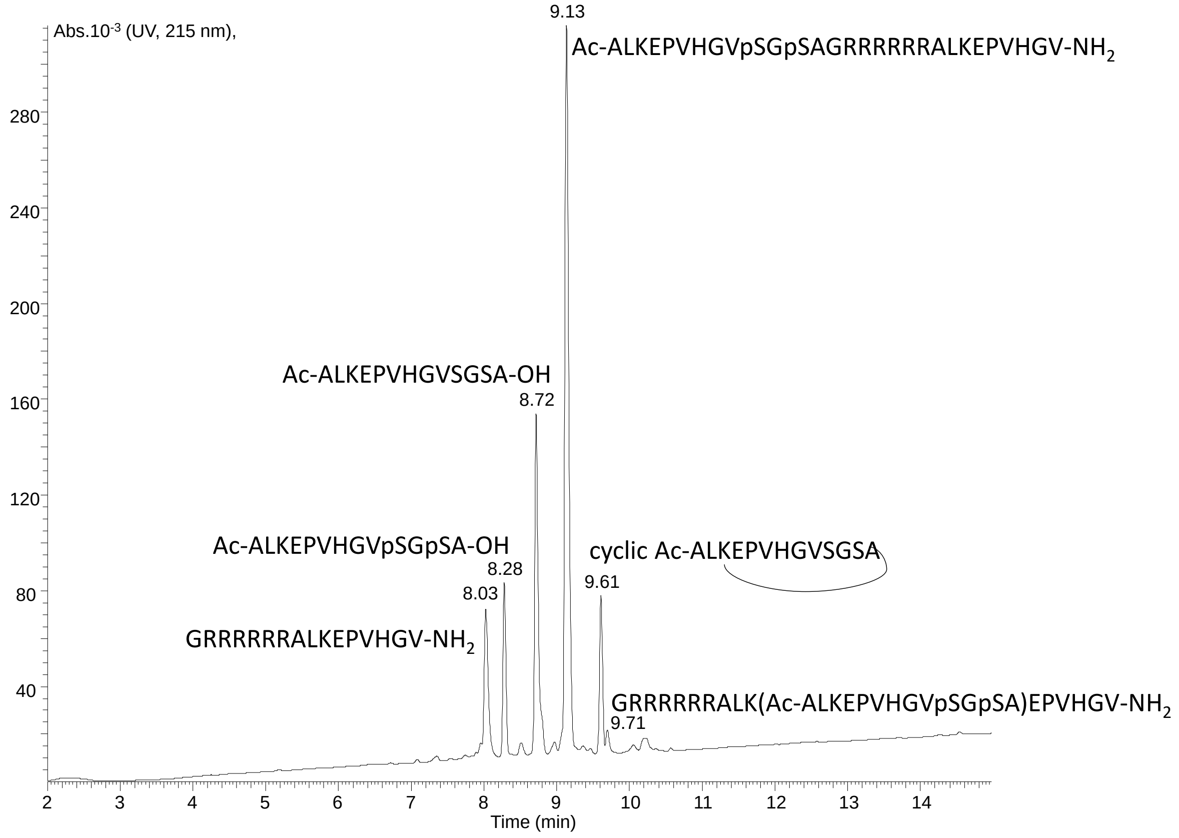

Supplementary Figure 193. UPLC-MS analysis of competition experiment using Ac-ALKEPVHGVpSGpSA-MPAA **1a**, Ac-ALKEPVHGVSGSA-MPAA **1d** and GRRRRRRALKEPVHGV-NH_2 **2a** after 48 h in 10 mM sodium bicarbonate/CO_2 buffer. LC trace. Eluent A 0.1% TFA in water, eluent B 0.1% TFA in CH_3CN. C18 BEH 300 Å (1.7 µm, 2.1 × 100 mm) column, gradient 0-40% B in 15 min, 0.4 mL min^{-1}, detection at 215 nm).

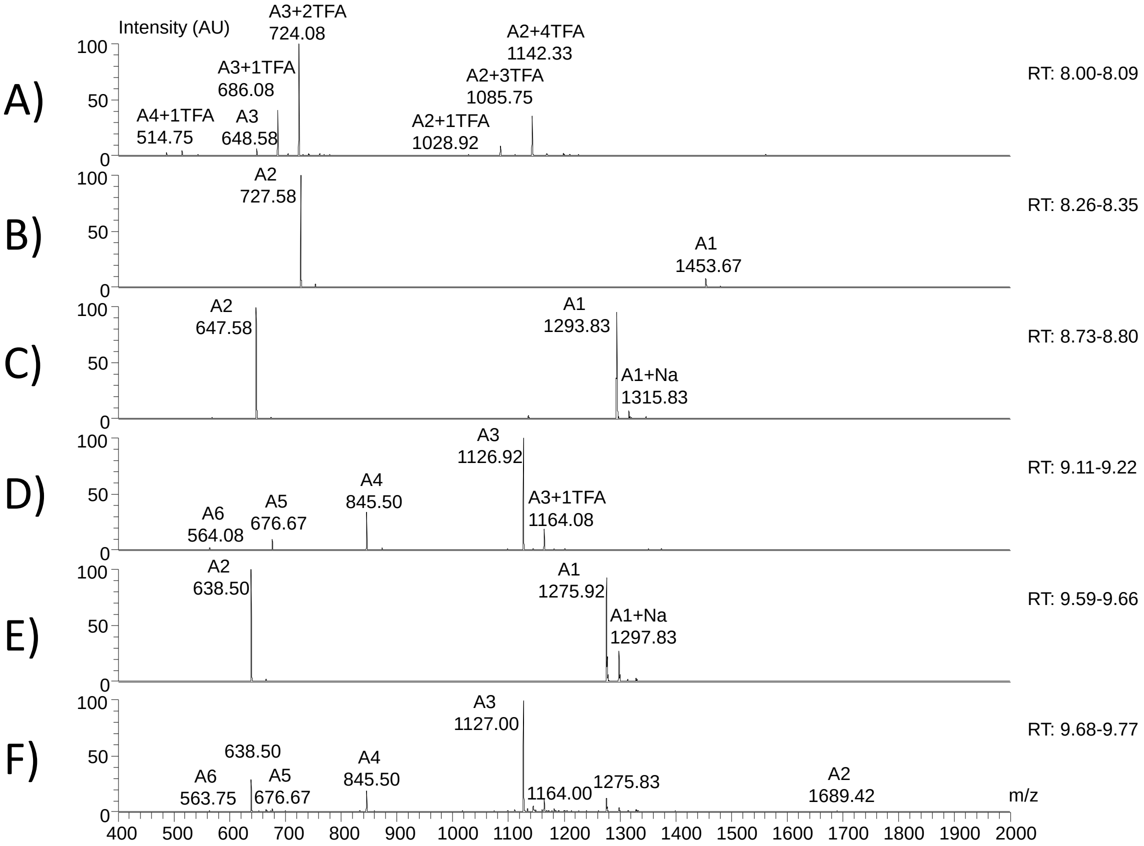

Supplementary Figure 194. MS traces from UPLC-MS analysis of competition experiment using Ac-ALKEPVHGVpSGpSA-MPAA **1a**, Ac-ALKEPVHGVSGSA-MPAA **1d** and GRRRRRRALKEPVHGV-NH_2 **2a** after 48 h in 10 mM sodium bicarbonate/CO_2 buffer. A) Rt = 8.03 min, GRRRRRRALKEPVHGV-NH_2 **2a**. $[M+TFA+2H]^{2+}$ m/z calcd. (av.) 1029.16, obs. 1028.92, $[M+TFA+3H]^{3+}$ m/z calcd. (av.) 686.44, obs. 686.08, $[M+TFA+4H]^{4+}$ m/z calcd. (av.) 515.08, obs. 514.75. B) Rt = 8.28 min, Ac-ALKEPVHGVpSGpSA-OH. $[M+H]^{+}$ m/z calcd. (monoisotopic) 1453.61, obs. 1453.67, $[M+2H]^{2+}$ m/z calcd. (av.) 727.70, obs. 727.58. C) Rt = 8.72 min, Ac-ALKEPVHGVSGSA-OH. $[M+H]^{+}$ m/z calcd. (monoisotopic) 1293.67, obs. 1293.83, $[M+2H]^{2+}$ m/z calcd. (av.) 647.72, obs. 647.58. D) Rt = 9.13 min, Ac-ALKEPVHGVpSGpSAGRRRRRRALKEPVHGV-NH_2. $[M+3H]^{3+}$ m/z calcd. (av.) 1126.90, obs. 1126.92, $[M+4H]^{4+}$ m/z calcd. (av.) 845.43, obs. 845.5, $[M+5H]^{5+}$ m/z calcd. (av.) 676.54, obs. 676.67, $[M+6H]^{6+}$ m/z calcd. (av.) 563.95, obs. 564.08. E) Rt = 9.61 min, cyclized peptide thioester **1d** byproduct Ac-ALKEPVHGVSGSA. $[M+H]^{+}$ m/z calcd. (monoisotopic) 1275.66, obs. 1275.92, $[M+2H]2^{+}$ m/z calcd. (av.) 638.71, obs. 638.50. F) Rt = 9.71 min, branched byproduct GRRRRRRALK(Ac-ALKEPVHGVpSGpSA)EPVHGV-NH_2. $[M+2H]^{2+}$ m/z calcd. (av.) 1689.89, obs. 1689.42, $[M+3H]^{3+}$ m/z calcd. (av.) 1126.90, obs. 1127.00, $[M+4H]^{4+}$ m/z calcd. (av.) 845.43, obs. 845.50, $[M+5H]^{5+}$ m/z calcd. (av.) 676.54, obs. 676.67, $[M+6H]^{6+}$ m/z calcd. (av.) 563.95, obs. 563.75.

Competition experiment 2: 1a + 2a + 2g →

GALKEPVHGV-NH_2 **2g** (75 µg, 5.57.10^{-8} mol, 0.3 mM final concentration) was dissolved in 10 mM sodium bicarbonate/CO_2 buffer (187.4 µL). This solution was quickly added to GRRRRRRALKEPVHGV-NH_2 **2a** (166 µg, 5.59.10^{-8} mol, 0.3 mM final concentration). The glycyl peptides were then added to Ac-ALKEPVHGVpSGpSA-MPAA **1a** (103 µg, 5.62.10^{-8} mol, 0.3 mM final concentration). The reaction mixture was placed in a CO_2 incubator for cell biology (5% CO_2 partial pressure, 37 °C, water saturated) with a needle inserted through the cap to allow CO_2 to diffuse into the tube. The final pH of the reaction mixture was 6.97. After 72 h, a sample of the reaction mixture was analyzed by UPLC-MS by diluting 4.5 µL of the reaction mixture with aqueous AcOH (10% by vol, 30 µL).

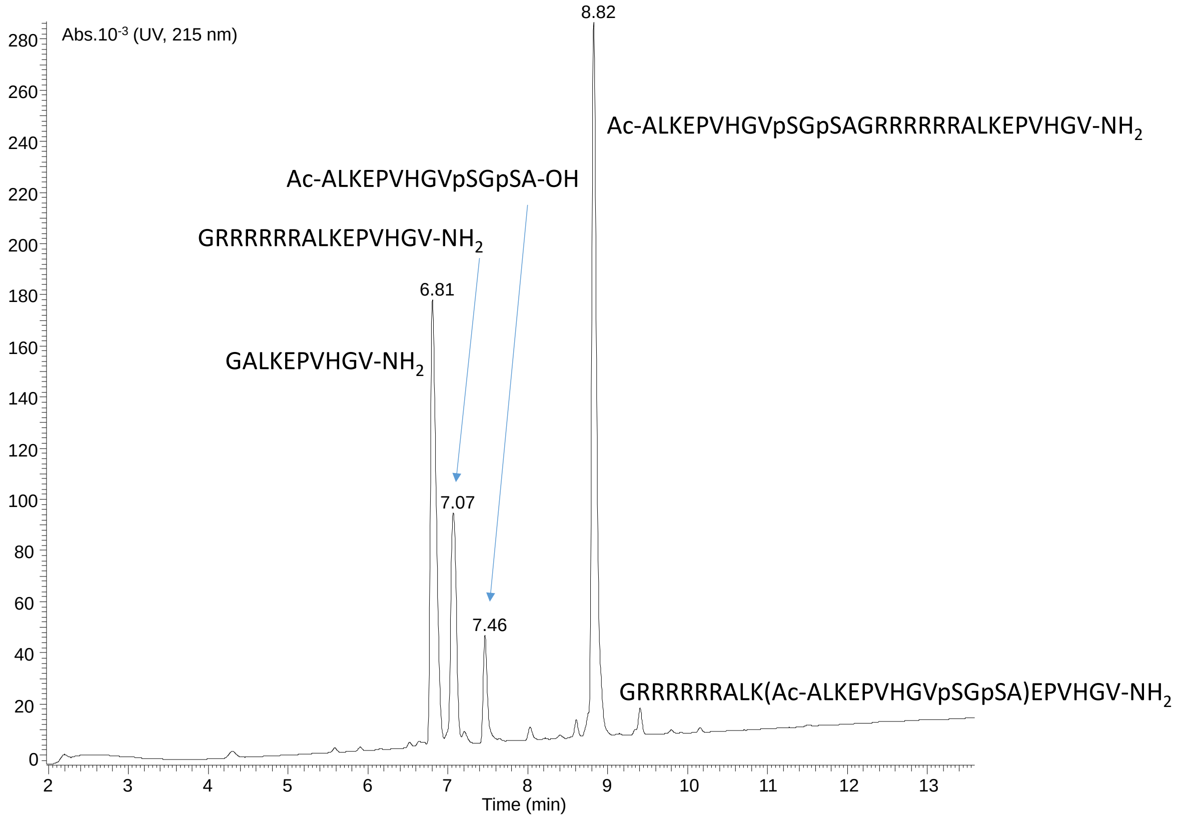

Supplementary Figure 195. UPLC-MS analysis of competition experiment using Ac-ALKEPVHGVpSGpSA-MPAA **1a**, GRRRRRRALKEPVHGV-NH_2 **2a** and GALKEPVHGV-NH_2 **2g** after 72 h in 10 mM sodium bicarbonate/CO_2 buffer. LC trace. Eluent A 0.1% TFA in water, eluent B 0.1% TFA in CH_3CN. SB C3 (1.8 µm, 3.0 × 100 mm) column, gradient 0-40% B in 15 min, 0.4 mL min^{-1}, detection at 215 nm).

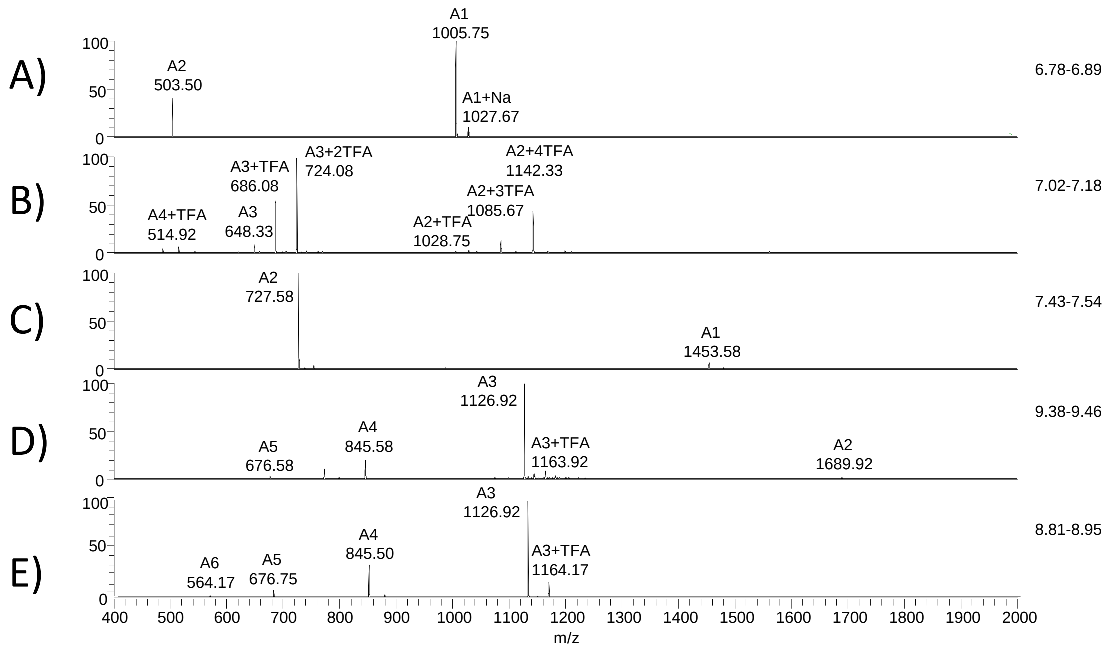

Supplementary Figure 196. MS traces from UPLC-MS analysis of competition experiment using Ac-ALKEPVHGVpSGpSA-MPAA **1a**, GRRRRRRALKEPVHGV-NH_2 **2a** and GALKEPVHGV-NH_2 **2g** after 72 h in 10 mM sodium bicarbonate/CO_2 buffer. A) Rt = 6.81 min, GALKEPVHGV-NH_2 **2g**. $[M+H]^+$ m/z calcd. (monoisotopic) 1005.58, obs. 1005.75, $[M+2H]^{2+}$ m/z calcd. (av.) 503.59, obs. 503.50. B) Rt = 7.07 min, GRRRRRRALKEPVHGV-NH_2 **2a**. $[M+TFA+2H]^{2+}$ m/z calcd. (av.) 1029.16, obs. 1028.75, $[M+TFA+3H]^{3+}$ m/z calcd. (av.) 686.44, obs. 686.08, $[M+TFA+4H]^{4+}$ m/z calcd. (av.) 515.08, obs. 514.92. C) Rt = 7.46 min, Ac-ALKEPVHGVpSGpSA-OH. $[M+H]^+$ m/z calcd. (monoisotopic) 1453.61, obs. 1453.58, $[M+2H]^{2+}$ m/z calcd. (av.) 727.70, obs. 727.58. D) Rt = 9.38 min, branched byproduct GRRRRRRALK(Ac-ALKEPVHGVpSGpSA)EPVHGV-NH_2. $[M+2H]^{2+}$ m/z calcd. (av.) 1689.89, obs. 1689.92, $[M+3H]^{3+}$ m/z calcd. (av.) 1126.90, obs. 1126.92, $[M+4H]^{4+}$ m/z calcd. (av.) 845.43, obs. 845.58, $[M+5H]^{5+}$ m/z calcd. (av.) 676.54, obs. 676.58. E) Rt = 8.82 min, Ac-ALKEPVHGVpSGpSAGRRRRRRALKEPVHGV-NH_2 **3a,a**. $[M+3H]^{3+}$ m/z calcd. (av.) 1126.90, obs. 1126.92, $[M+4H]^{4+}$ m/z calcd. (av.) 845.43, obs. 845.50, $[M+5H]^{5+}$ m/z calcd. (av.) 676.54, obs. 676.75, $[M+6H]^{6+}$ m/z calcd. (av.) 563.95, obs. 564.17.

Aminolysis rate law and dependence to the ionic strength

Aminolysis rate law

The aminolysis reaction was studied using peptide thioester **1a** and glycyl peptide **2a** as reactants.

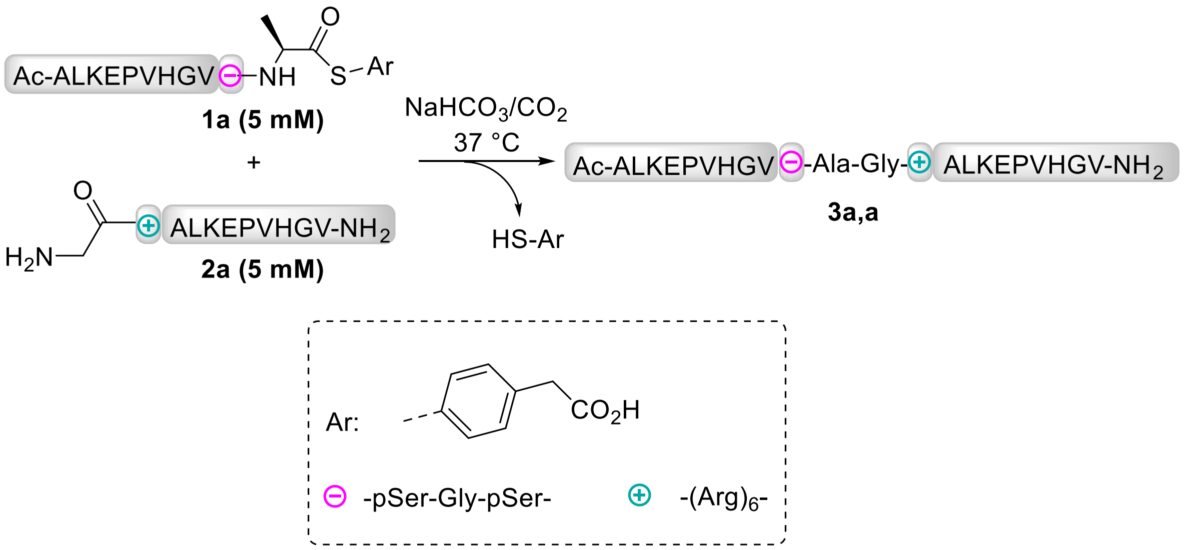

Analysis: The reaction was performed according to the general method described previously.

The experimental data points related to aminolysis product formation **3a,a**, thioester hydrolysis product Ac-ALKEPVHGVpSGpSA-OH, branched byproduct formation

Ac-ALKEPVHGVpSGpSA
|
GRRRRRRALKEPVHGV-NH$_2$

and residual thioester **1a** were simultaneously fitted in order to determine the apparent first order constants. The complete model reaction used for numerical fitting of the aminolysis reaction corresponds to multiple competitive apparent first order chemical processes in the form of:

Thioester **1a** → aminolysis product **3a,a** described by k_1

Thioester **1a** → hydrolysis product Ac-ALKEPVHGVpSGpSA-OH described by k_2

Thioester **1a** → branched byproduct

Ac-ALKEPVHGVpSGpSA
|
GRRRRRRALKEPVHGV-NH$_2$

described by k_3

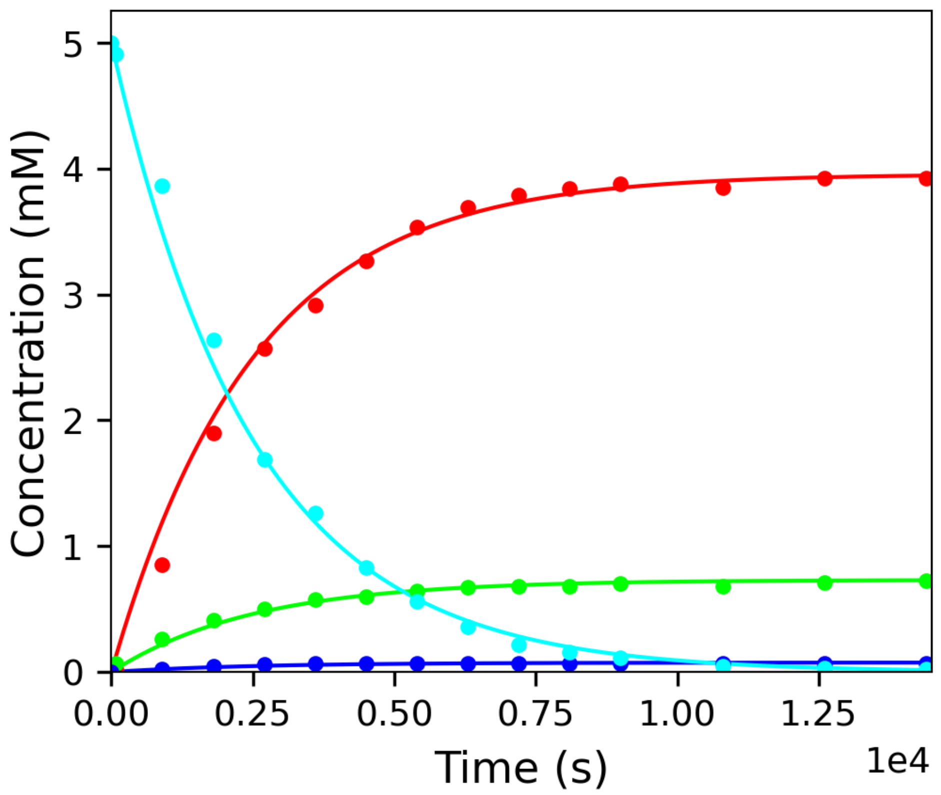

Supplementary Figure 197. Representative fitting curves of aminolysis products formation (sodium hydrogenocarbonate (50 mM)/CO_2 buffer, ~5 mM final concentration for each peptide, pH ~7.2, 37 °C). Experimental data points are represented by colored dots, the fitting curve by colored lines (red: aminolysis product **3a,a**, green: hydrolysis product Ac-ALKEPVHGVpSGpSA-OH, dark blue: branched byproduct

Ac-ALKEPVHGVpSGpSA
|
GRRRRRRALKEPVHGV-NH_2

, cyan: aryl thioester **1a**).

Supplementary Table 1. Apparent first order rate constant of aminolysis **3a,a**, hydrolysis Ac-ALKEPVHGVpSGpSA-OH and branched byproduct Ac-ALKEPVHGVpSGpSA | GRRRRRRALKEPVHGV-NH_2 formation.[a]

Rate constant	k_{app} (× 10^4 s^{-1})	σ (× 10^4)
k_1	3.31	1.66
k_2	0.61	0.05
k_3	0.06	0.002

[a] Sodium hydrogenocarbonate (50 mM)/CO_2 buffer, ~5 mM final concentration for each peptide, pH ~7.2, 37 °C.

Aminolysis rate dependence to the ionic strength (Fig. 3f)

The aminolysis reaction was studied using peptide thioester **1a** and glycyl peptide **2a** as reactants.

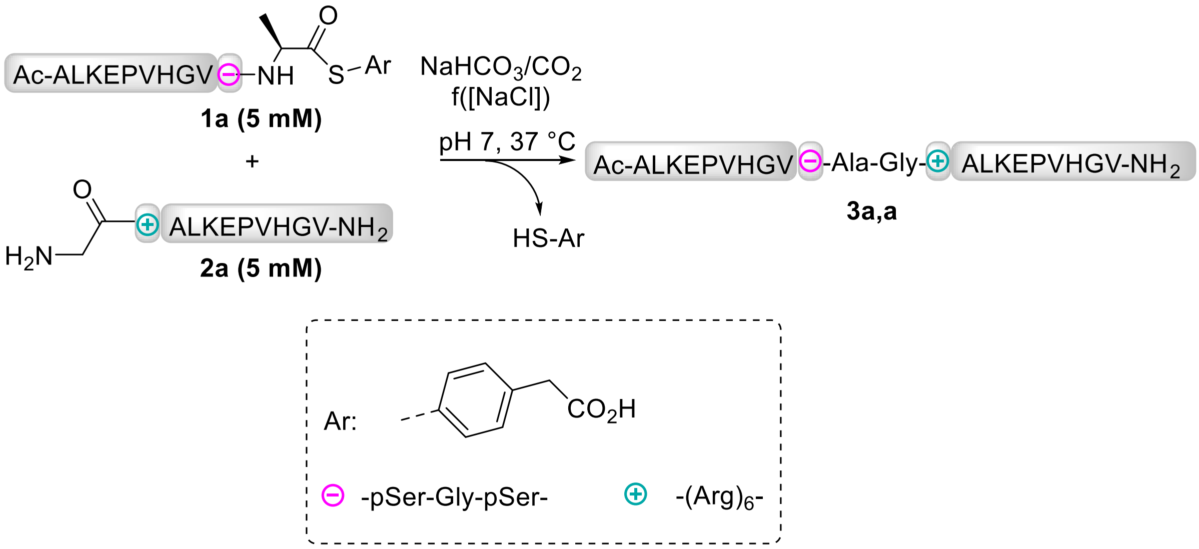

The conditions studied were: 50 mM sodium hydrogenocarbonate/CO_2 buffer, 5 mM Ac-ALKEPVHGVpSGpSA-MPAA **1a**, 5 mM GRRRRRRALKEPVHGV-NH_2 **2a**, 37 °C.

1) w/o NaCl, pH 7.41

2) 20 mM NaCl, pH 7.23

3) 50 mM NaCl, pH 7.11

4) 90 mM NaCl, pH 7.32

5) 150 mM NaCl, pH 7.36

6) 250 mM NaCl, pH 7.09

Only the data points related to product formation were used in order to determine the apparent first order constants given below. The model reaction used for numerical fitting of the aminolysis corresponds to an apparent first order reaction in the form of A $\rightarrow$ P.

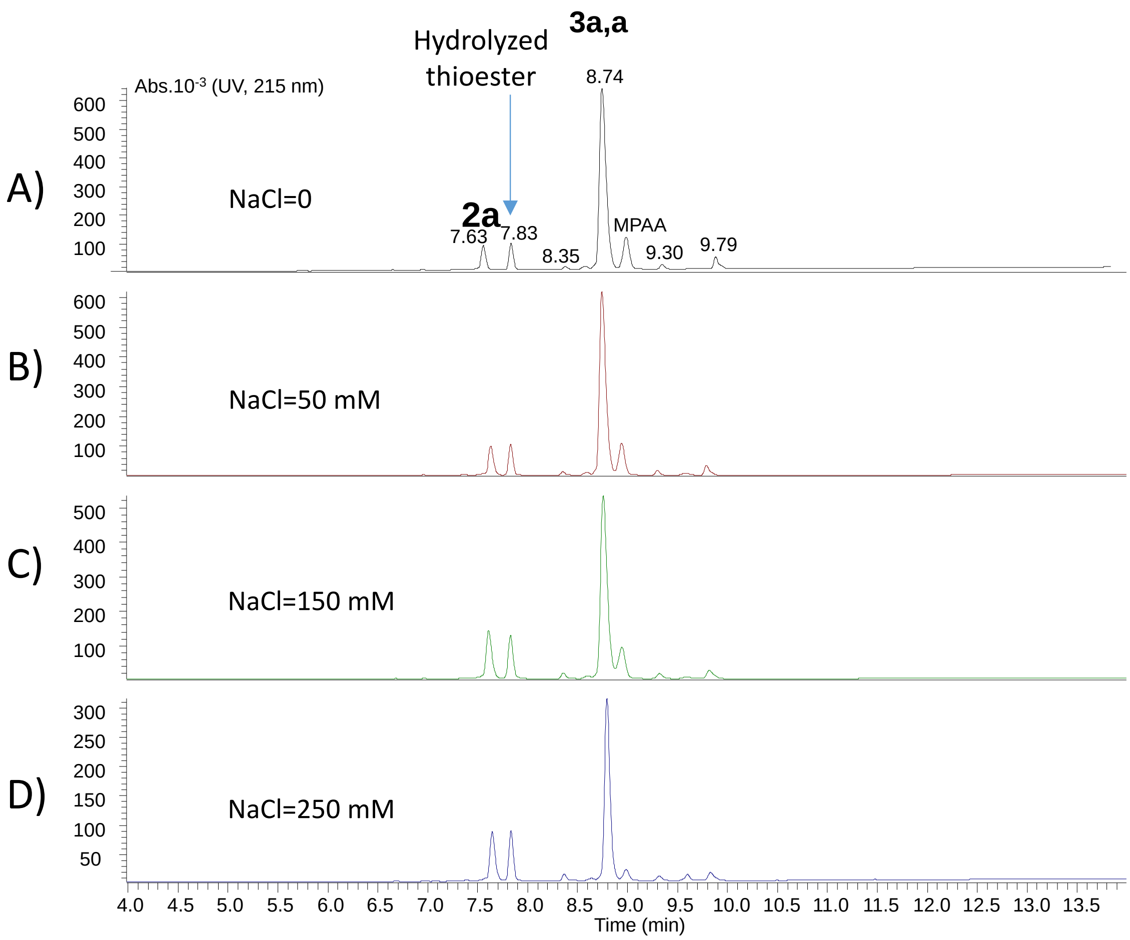

Supplementary Figure 198. UPLC-MS analysis of the reaction of peptide thioester Ac-ALKEPVHGVpSGpSA-MPAA **1a** with glycyl peptide GRRRRRRALKEPVHGV-NH_2 **2a** after 4 h in the presence of increasing concentrations of NaCl: (A) w/o; B) 50 mM; C) 150 mM; D) 250 mM). LC trace. Eluent A 0.1% TFA in water, eluent B 0.1% TFA in CH_3CN. C18 BEH 300 Å (1.7 µm, 2.1 × 100 mm) column, gradient 0-40% B in 15 min, 0.4 mL min^{-1}, detection at 215 nm).

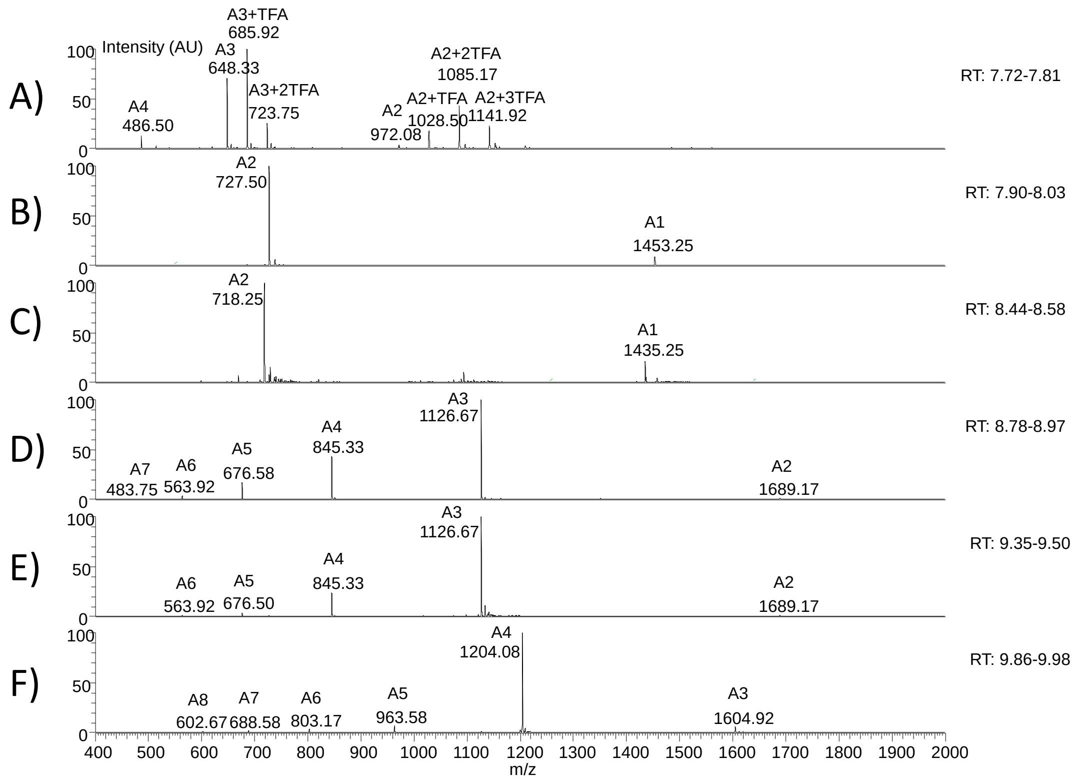

Supplementary Figure 199. MS traces from UPLC-MS analysis of the reaction of peptide thioester Ac-ALKEPVHGVpSGpSA-MPAA **1a** with glycyl peptide GRRRRRRALKEPVHGV-NH2 **2a** after 4 h in the presence of 50 mM NaCl. A) Rt = 7.63 min, GRRRRRRALKEPVHGV-NH_2 **2a**. $[M+2H]^{2+}$ m/z calcd. (av.) 972.16, obs. 972.08, $[M+3H]^{3+}$ m/z calcd. (av.) 648.44, obs. 648.33, $[M+4H]^{4+}$ m/z calcd. (av.) 486.58, obs. 486.50. B) Rt = 7.83 min, peptide thioester hydrolysis byproduct Ac-ALKEPVHGVpSGpSA-OH. $[M+H]^{+}$ m/z calcd. (monoisotopic) 1453.60, obs. 1453.25, $[M+2H]^{2+}$ m/z calcd. (av.) 727.70, obs. 727.50. C) Rt = 8.35 min, peptide thioester cyclized byproduct. $[M+H]^{+}$ m/z calcd. (monoisotopic) 1435.60, obs. 1435.25, $[M+2H]^{2+}$ m/z calcd. (av.) 718.69, obs. 718.25. D) Rt = 8.74 min, target ligation product Ac-ALKEPVHGVpSGpSAGRRRRRRALKEPVHGV-NH_2 **3a,a**. $[M+2H]^{2+}$ m/z calcd. (av.) 1689.85, obs.1689.17, $[M+3H]^{3+}$ m/z calcd. (av.c) 1126.90, obs. 1126.67, $[M+4H]^{4+}$ m/z calcd. (av.) 845.43, obs. 845.33, $[M+5H]^{5+}$ m/z calcd. (av.) 676.54, obs. 676.58, $[M+6H]^{6+}$ m/z calcd. (av.) 563.95, obs. 563.92, $[M+7H]^{7+}$ m/z calcd. (av.) 483.53, obs. 483.75. E) Rt = 9.30 min, branched byproduct GRRRRRRALK(Ac-ALKEPVHGVpSGpSA)EPVHGV-NH_2. MS trace, $[M+2H]^{2+}$ m/z calcd. (av.) 1689.85, obs.1689.17, $[M+3H]^{3+}$ m/z calcd. (av.) 1126.90, obs. 1126.67, $[M+4H]^{4+}$ m/z calcd. (av.) 845.43, obs. 845.33, $[M+5H]^{5+}$ m/z calcd. (av.) 676.54, obs. 676.50, $[M+6H]^{6+}$ m/z calcd. (av.) 563.95, obs. 563.92. F) Rt = 9.79 min, double acylation byproduct

Ac-ALKEPVHGVpSGpSA
|
Ac-ALKEPVHGVpSGpSA-GRRRRRRALKEPVHGV-NH_2

. $[M+3H]^{3+}$ m/z calcd. (av.) 1605.37, obs.1604.92, $[M+4H]^{4+}$ m/z calcd. (av.) 1204.27, obs. 1204.08, $[M+5H]^{5+}$ m/z calcd. (av.) 963.62, obs. 963.58, $[M+6H]^{6+}$ m/z calcd. (av.) 803.18, obs. 803.17, $[M+7H]^{7+}$ m/z calcd. (av.) 688.58, obs. 688.58, $[M+8H]^{8+}$ m/z calcd. (av.) 602.64, obs. 602.67.

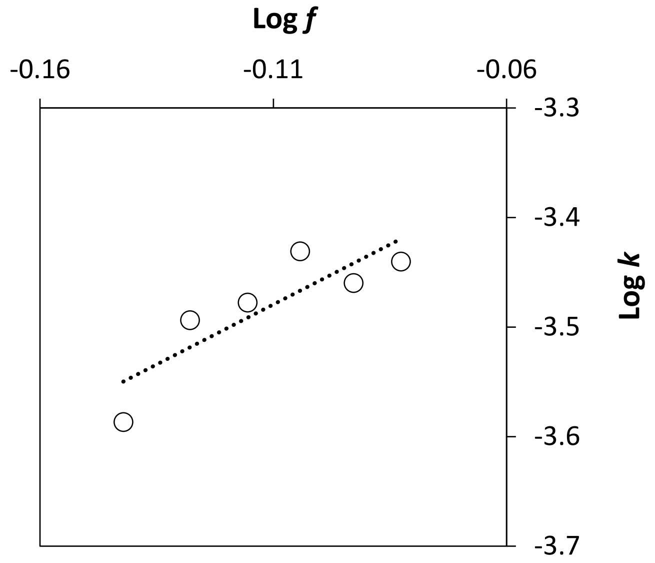

Supplementary Figure 200. Relationship between Log *k* and Log *f*. *k* is the observed pseudo-first order rate constant. *f* is the electrostatic component of the mean rational activity coefficient for NaCl, which is the dominant factor in the activity coefficient up to 0.5 M, for a detailed discussion see ref [7].

Supplementary Table 2. Apparent first order rate constants for aminolysis product **3a,a** formation.[a] The kinetic parameters given below were determined by analytical fitting to the monoexponential function $[P] = [P]_{final} (1-e^{-kt})$ using Kintek explorer software.

Entry	Exp. Conditions	k_{app} (s^{-1}) (× 10^4)	σ (s^{-1}) (× 10^4)	Conversion (%)
1	w/o NaCl, pH 7.41	3.63	0.16	80.3
2	20 mM NaCl, pH 7.23	3.47	0.14	77.7
3	50 mM NaCl, pH 7.11	3.71	0.11	75.7
4	90 mM NaCl, pH 7.32	3.33	0.20	72.3
5	150 mM NaCl, pH 7.36	3.21	0.15	69.2
6	250 mM NaCl, pH 7.09	2.59	0.15	64.6

[a] Sodium hydrogenocarbonate/CO_2 buffer 50 mM, ~5 mM final concentration for each peptide, pH ~7.2, 37 °C.

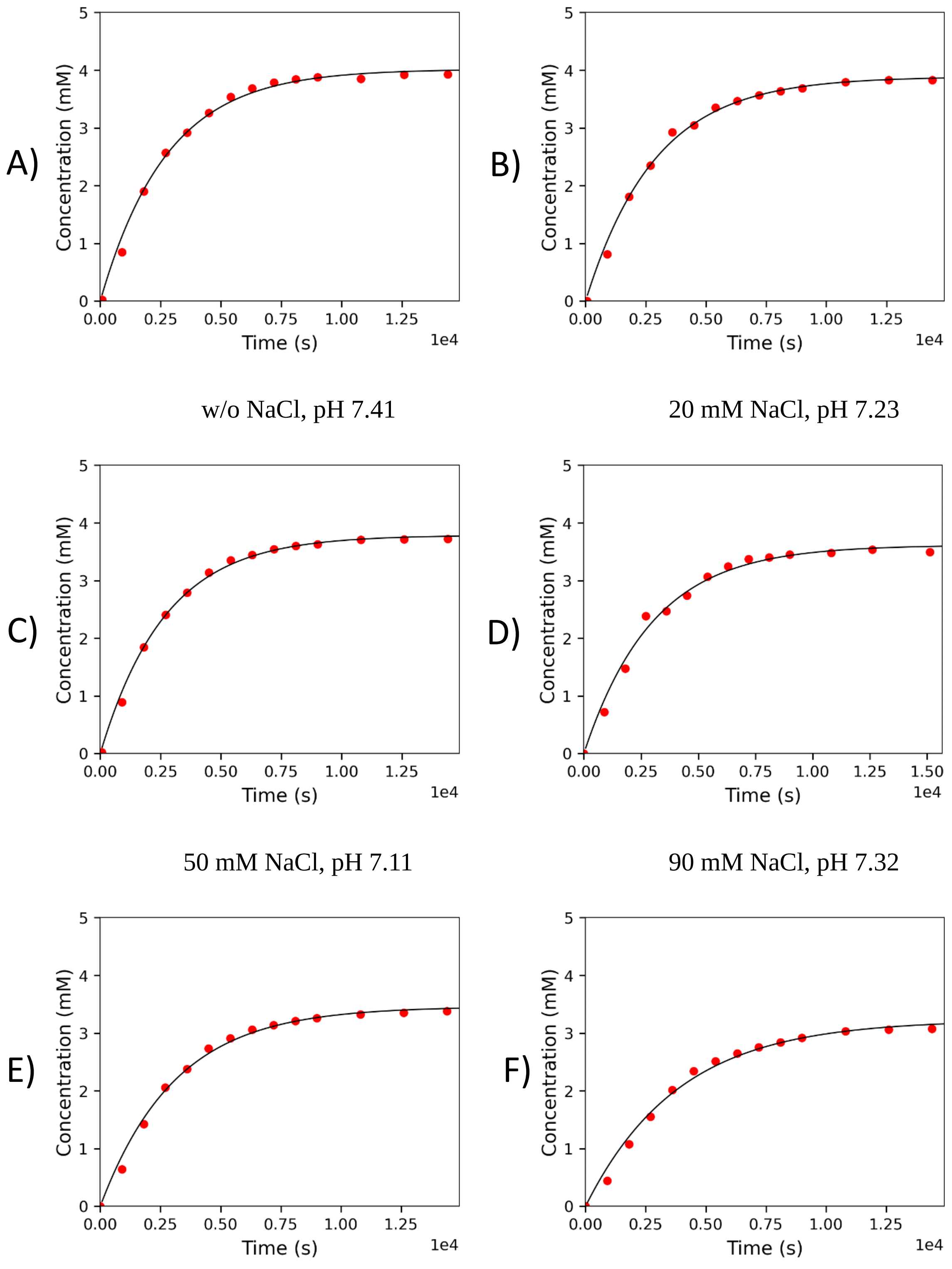

w/o NaCl, pH 7.41

20 mM NaCl, pH 7.23

50 mM NaCl, pH 7.11

90 mM NaCl, pH 7.32

150 mM NaCl, pH 7.36

250 mM NaCl, pH 7.09

Supplementary Figure 201. Representative fitting curves of aminolysis product **3a,a** formation in sodium hydrogenocarbonate/CO_2 buffer (50 mM) in the presence of increasing concentrations of NaCl. A) w/o NaCl. B) 20 mM NaCl. C) 50 mM NaCl. D) 90 mM NaCl. E) 150 mM NaCl. F) 250 mM NaCl. Experimental data points are represented by red dots, the fitting curve by a red line.

Enzymatic dephosphorylation of peptide thioester aminolysis product

Experimental procedure

CALF intestinal alkaline phosphatase (1 µL, 20 u/µL, Sigma Aldrich) was diluted in 50 mM ammonium bicarbonate pH=7.9 (399 µL) to a final concentration of 0.05 u/µL. This solution (20 µL) was added to peptide **3a,a** (2 µg, 2 µL of a 1 mg/mL solution of **3a,a** in water) and the reaction mixture was placed at 37 °C. After 2 h 35, an aliquot of the reaction mixture (17 µL) was acidified with 5% aqueous acetic acid (40 µL) and analysed by UPLC-MS.

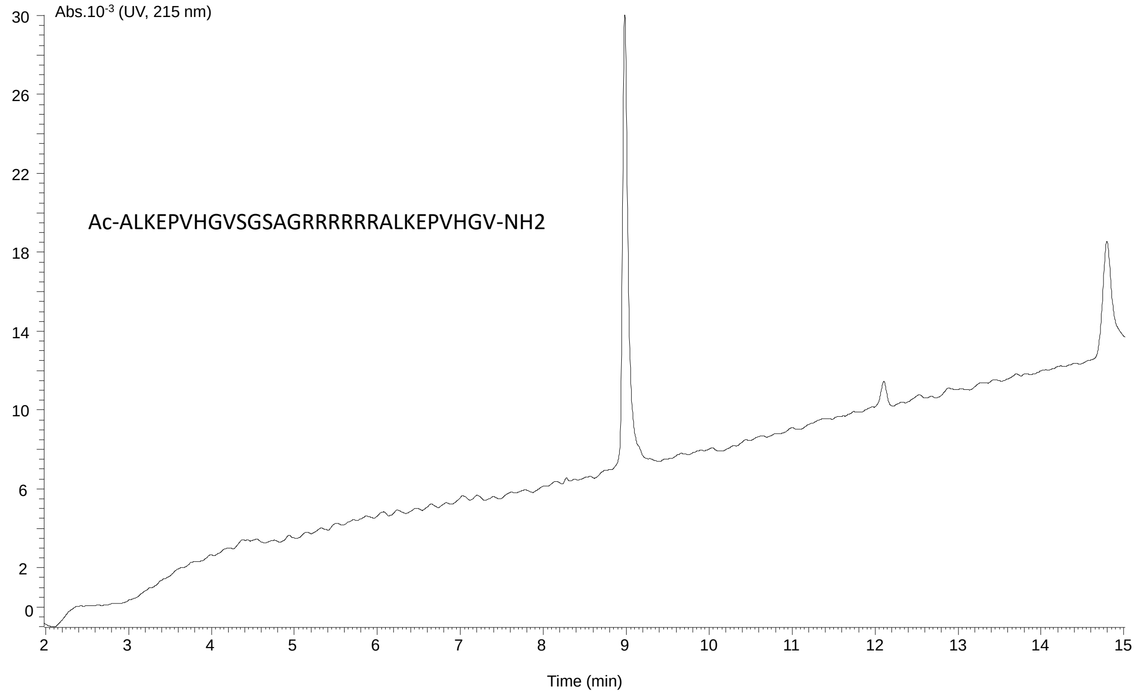

Supplementary Figure 202. Enzymatic dephosphorylation of peptide **3a,a.** UPLC-MS analysis of reaction mixture after 2 h 35 min. LC trace. Eluent A 0.1% TFA in water, eluent B 0.1% TFA in CH_3CN. C18 BEH 300 Å (1.7 µm, 2.1 × 100 mm) column, gradient 0-40% B in 15 min, 0.4 mL min^{-1}, detection at 215 nm).

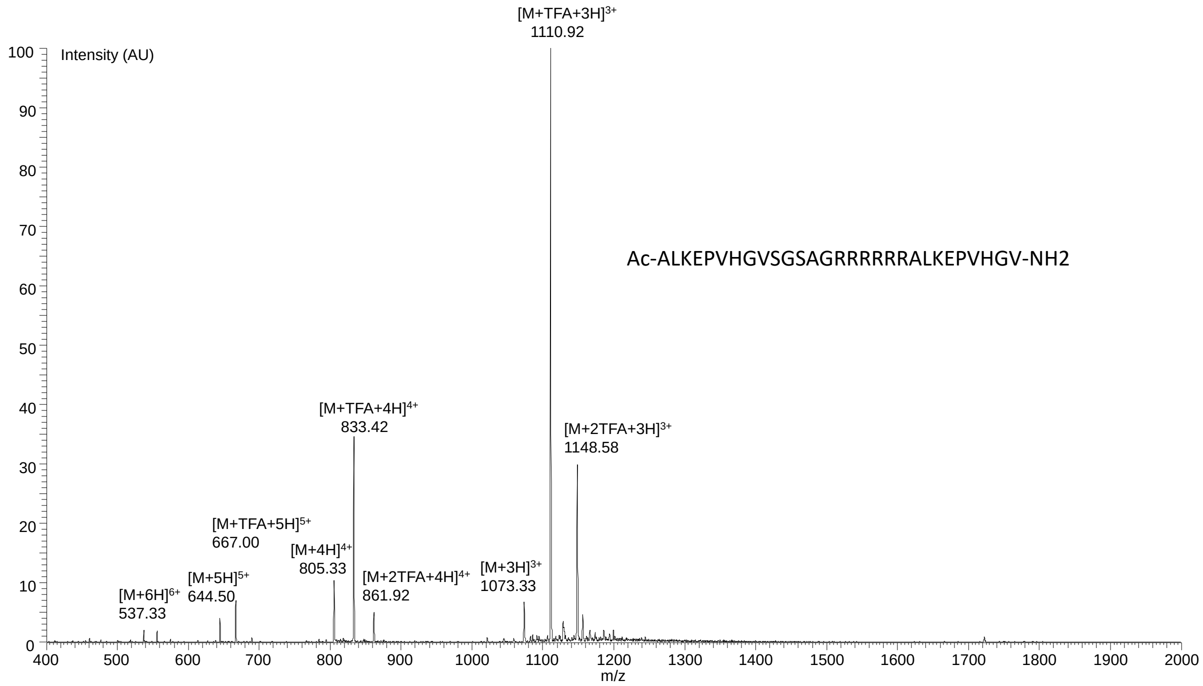

Supplementary Figure 203. Enzymatic dephosphorylation of peptide **3a,a**. MS trace from UPLC-MS analysis of the reaction mixture after 2 h 35 min. $[M+3H]^{3+}$ m/z calcd. (av.) 1073.91, obs. 1073.33, $[M+4H]^{4+}$ m/z calcd. (av.) 805.68, obs. 805.33, $[M+5H]^{5+}$ m/z calcd. (av.) 644.75, obs. 644.50, $[M+6H]^{6+}$ m/z calcd. (av.) 537.46, obs. 537.33.

Synthesis of I27 titin conjugate 6 (Fig. 2c-e)

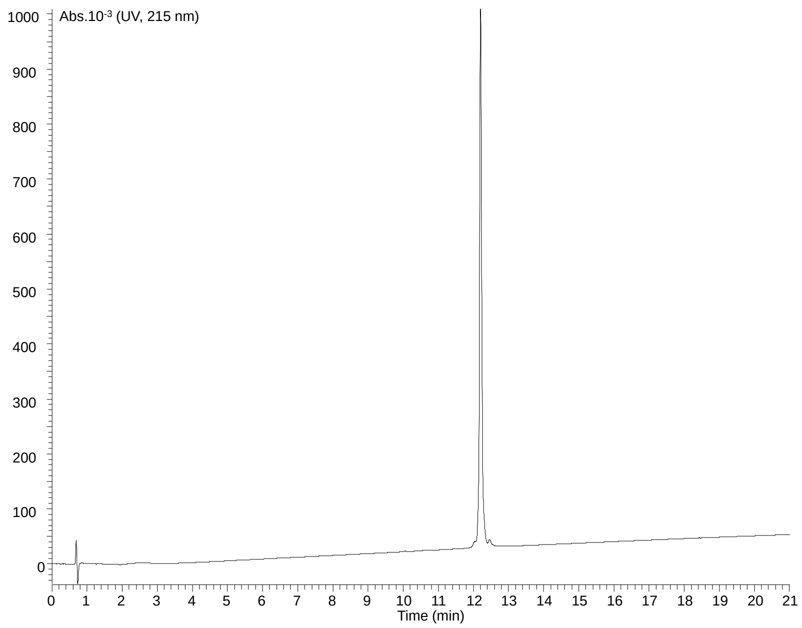

Supplementary Figure 204. UPLC-MS analysis of purified conjugate **6**. A) LC trace. Eluent A 0.1% TFA in water, eluent B 0.1% TFA in CH_3CN. SB C3 (1.8 µm, 3.0 × 100 mm) column, gradient 0-70% B in 20 min, 70 °C, 0.4 mL min^{-1}, detection at 215 nm).

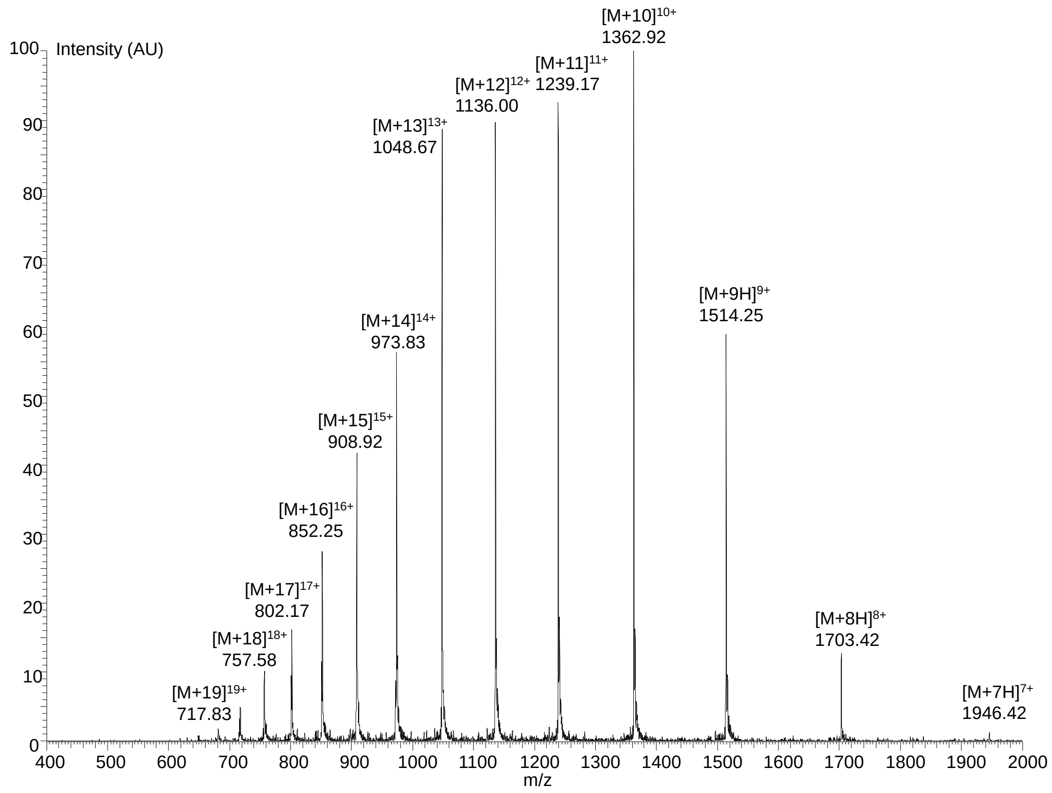

Supplementary Figure 205. MS trace from UPLC-MS analysis of purified conjugate **6**. $[M+7H]^{7+}$ m/z calcd. (av.) 1946.48, obs. 1946.42, $[M+8H]^{8+}$ m/z calcd. (av.) 1703.30, obs. 1703.42, $[M+9H]^{9+}$ m/z calcd. (av.) 1514.15, obs. 1514.25, $[M+10H]^{10+}$ m/z calcd. (av.) 1362.84, obs. 1362.92, $[M+11H]^{11+}$ m/z calcd. (av.) 1239.03, obs. 1239.17, $[M+12H]^{12+}$ m/z calcd. (av.) 1135.86, obs. 1136.00, $[M+13H]^{13+}$ m/z calcd. (av.) 1048.57, obs. 1048.67, $[M+14H]^{14+}$ m/z calcd. (av.) 973.74, obs. 973.83, $[M+15H]^{15+}$ m/z calcd. (av.) 908.89, obs. 908.92, $[M+16H]^{16+}$ m/z calcd. (av.) 852.15, obs. 852.25, $[M+17H]^{17+}$ m/z calcd. (av.) 802.08, obs. 802.17, $[M+18H]^{18+}$ m/z calcd. (av.) 757.58, obs. 757.58, $[M+19H]^{19+}$ m/z calcd. (av.) 717.76, obs. 717.83.

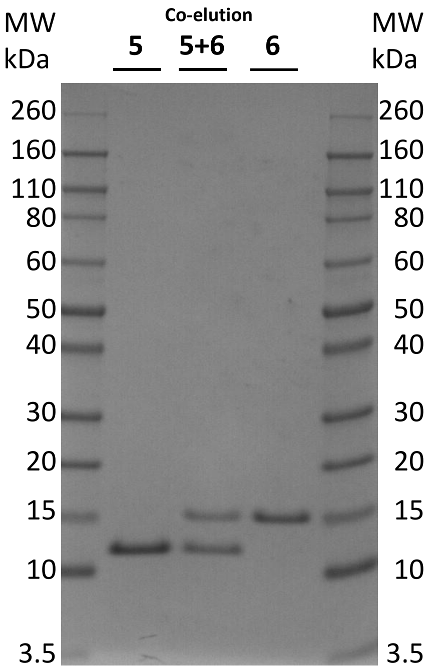

Supplementary Figure 206. SDS-PAGE analysis of purified titin proteins **5** and **6**. Coomassie staining. Coomassie (protein **5**, 2.3 µg deposited, protein **6**, 2.3 µg deposited, co-eluted proteins **5** and **6**, 1.15 µg each).

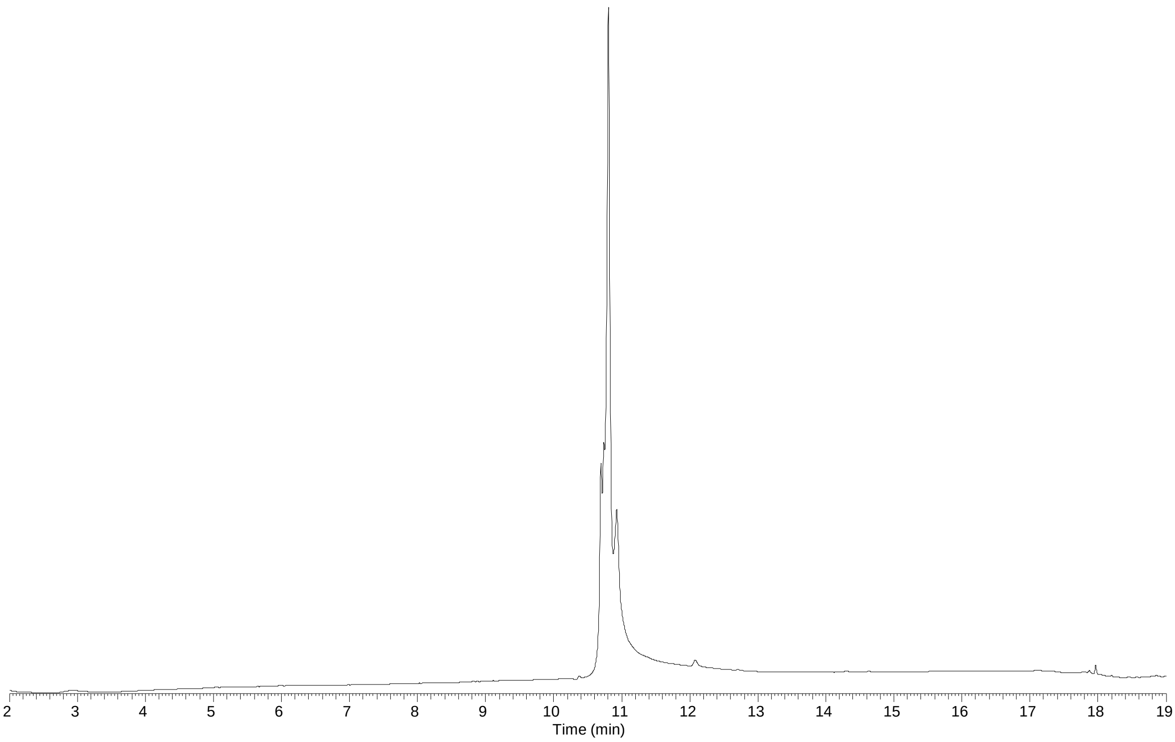

Supplementary Figure 207. Proteomic analysis of conjugate **6**. Alkylation step. UPLC-MS analysis of alkylated protein **6** (crude mixture). LC trace. Eluent A 0.10% TFA in water, eluent B 0.10% TFA in CH_3CN, ACQUITY UPLC Peptide BEH 300Å 1.7 μm (2.1 × 150 mm) column, 50 °C, gradient 0-70% B in 20 min, 0.4 mL/min, UV detection at 215 nm. MS trace: m/z = 1717.75 ($[M+8H]^{8+}$), 1527.00 ($[M+9H]^{9+}$), 1317.42 ($[M+10H]^{10+}$), 1249.42 ($[M+11H]^{11+}$), 1145.67 ($[M+12H]^{12+}$), 1057.67 ($[M+13H]^{13+}$), 982.08 ($[M+14H]^{14+}$), 916.75 ($[M+15H]^{15+}$), 859.25 ($[M+16H]^{16+}$), 807.75 ($[M+17H]^{17+}$), 763.67 ($[M+18H]^{18+}$). Calcd. for M (average) 13734.32, found: 13734.77.

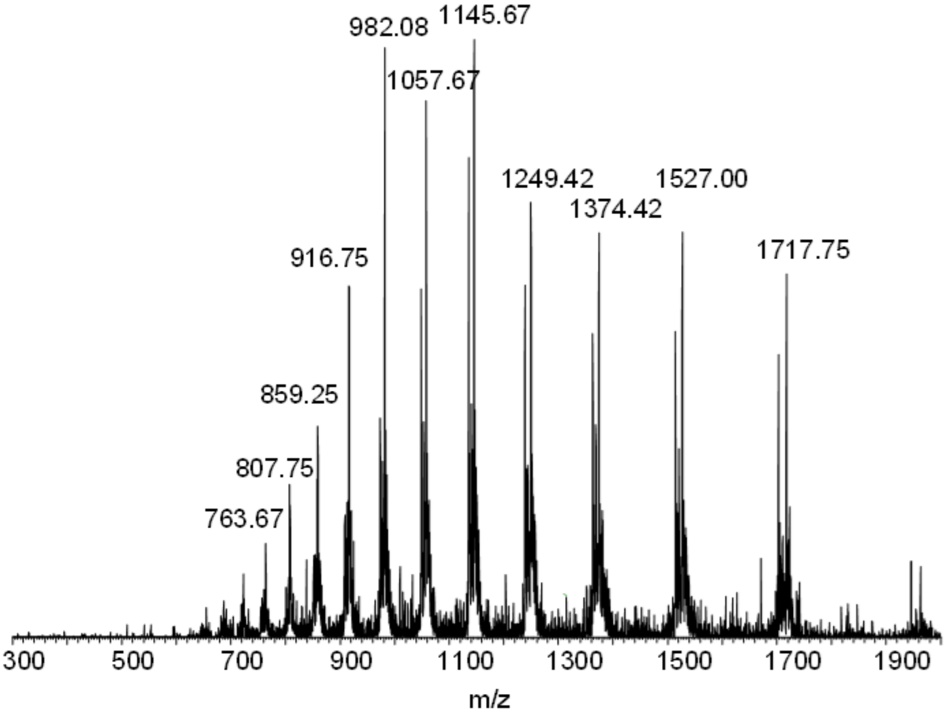

Supplementary Figure 208. Proteomic analysis of conjugate **6**. Alkylation step. MS trace from UPLC-MS analysis of alkylated protein **6** (crude mixture). m/z = 1717.75 ($[M+8H]^{8+}$), 1527.00 ($[M+9H]^{9+}$), 1317.42 ($[M+10H]^{10+}$), 1249.42 ($[M+11H]^{11+}$), 1145.67 ($[M+12H]^{12+}$), 1057.67 ($[M+13H]^{13+}$), 982.08 ($[M+14H]^{14+}$), 916.75 ($[M+15H]^{15+}$), 859.25 ($[M+16H]^{16+}$), 807.75 ($[M+17H]^{17+}$), 763.67 ($[M+18H]^{18+}$). Calcd. for M (average) 13734.32, found: 13734.77.

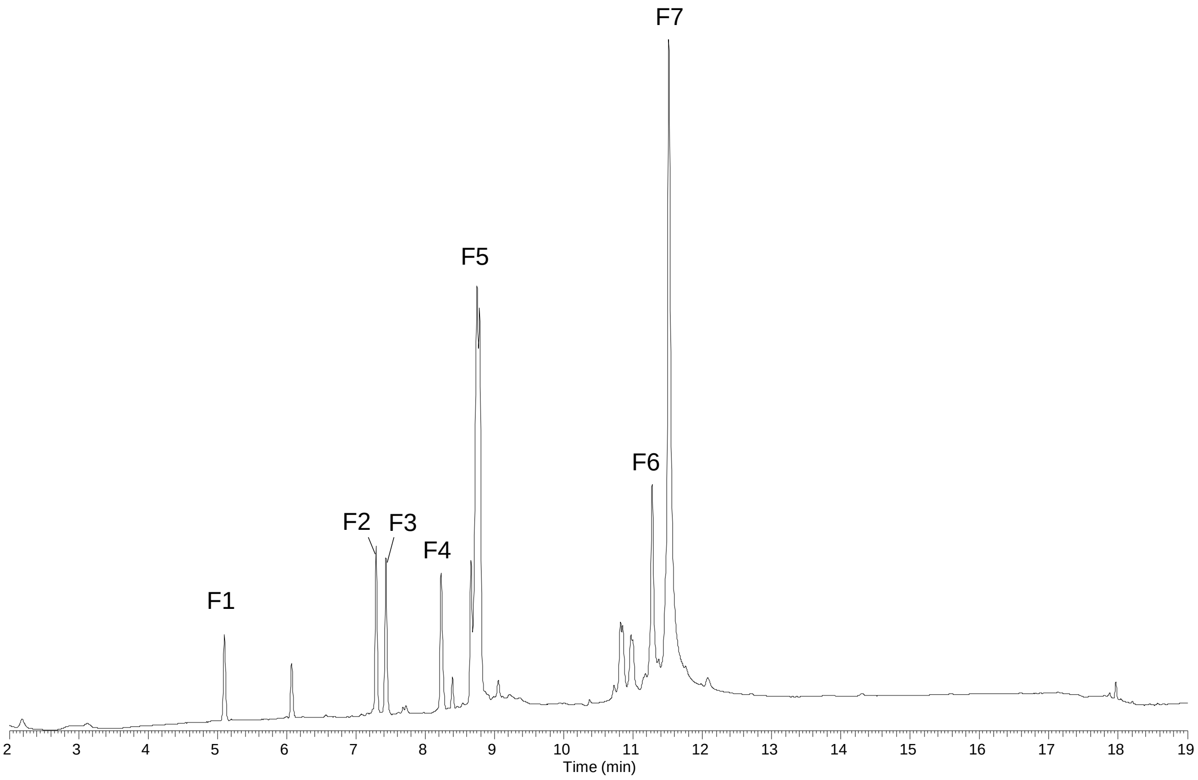

Supplementary Figure 209. UPLC MS analysis after trypsin digestion. Trypsin (0.1 mg/mL, 0.78 µL) was added to the alkylated protein **6**. The fragments obtained confirm the proposed structure for conjugate **6** coupling of the V5 tag to the N-terminus of titin protein **5**. The new peptide junction formed is in fragment F4. Chromatogram of the crude enzymatic lysate (after 90 min). Eluent A 0.10% TFA in water, eluent B 0.10% TFA in CH_3CN, ACQUITY UPLC Peptide BEH 300Å 1.7 µm (2.1 × 150 mm) column, 50 °C, gradient 0-70% B in 20 min, 0.4 mL/min, UV detection at 215 nm.

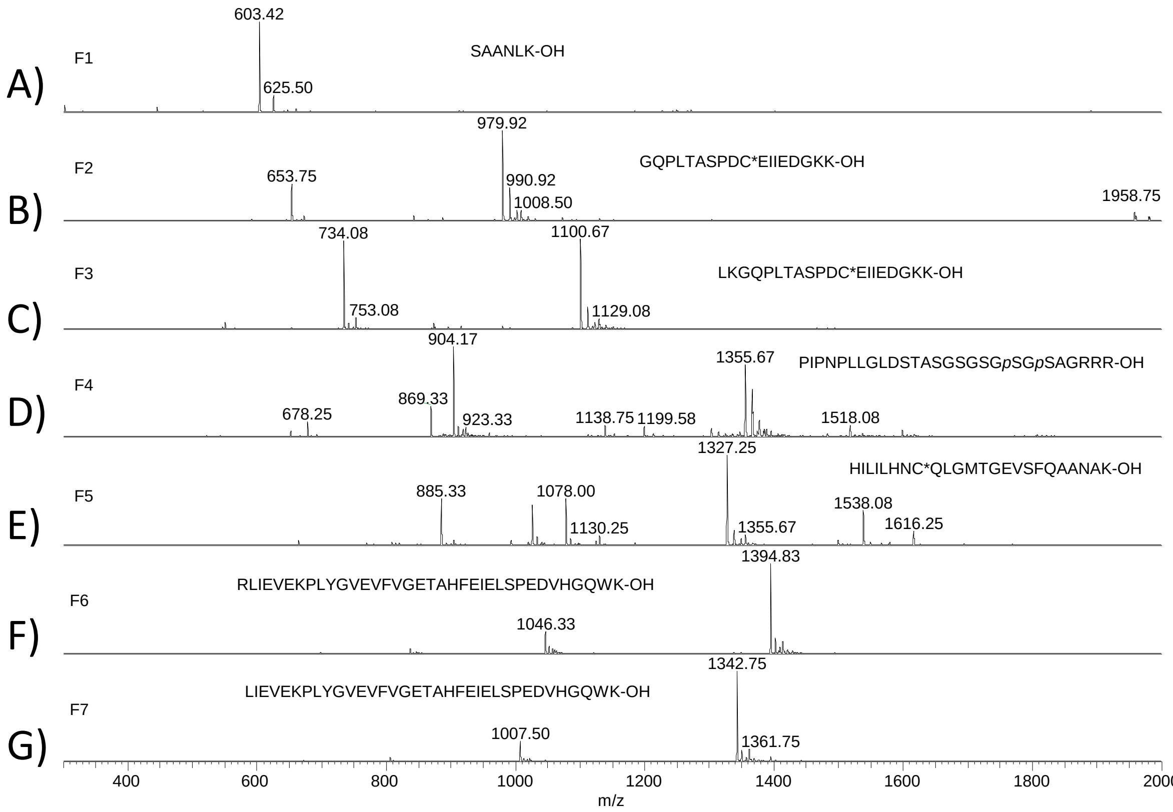

Supplementary Figure 210. MS traces of fractions F1-7 from UPLC-MS analysis after trypsin digestion (after 90 min) are shown in panels A to G, respectively. Trypsin (0.1 mg/mL, 0.78 µL) was added to the alkylated protein **6**. The fragments obtained confirm the proposed structure for conjugate **6** coupling of the V5 tag to the N-terminus of titin protein **5**. The new peptide junction formed is in fragment F4.

Electrostatic assistance of the native chemical ligation (NCL, Fig. 4)

In the experiment depicted in Fig 4b, the reaction of peptide thioester **1a** with Cys peptide **7** furnished ligation product **8a** as a major product, and a branched thioester which was quantified by HPLC.

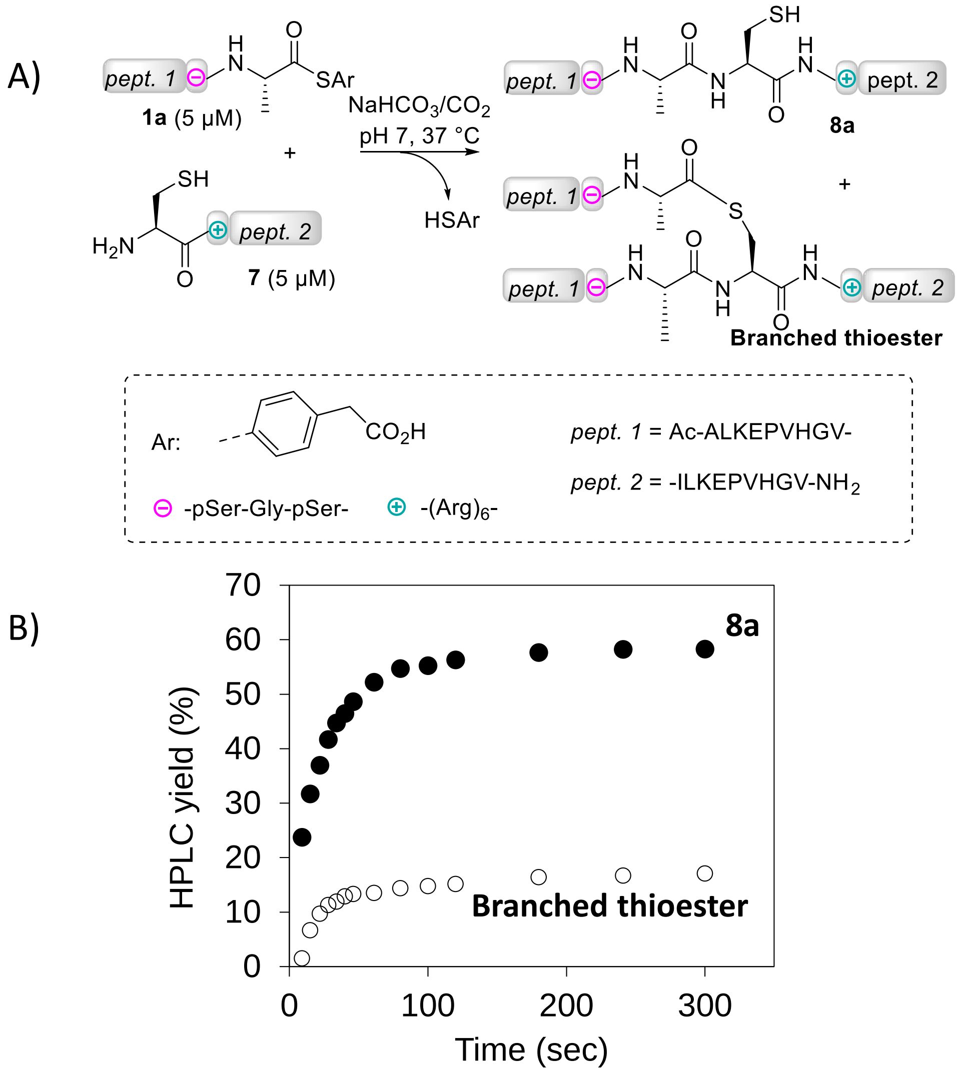

Supplementary Figure 211. Quantification of the branched thioester product formed during the ligation of peptide thioester **1a** with Cys peptide **7**. A) Reaction studied. B) Outcome of target ligation product **8a** (filled circles) and branched byproduct formation (open circles).

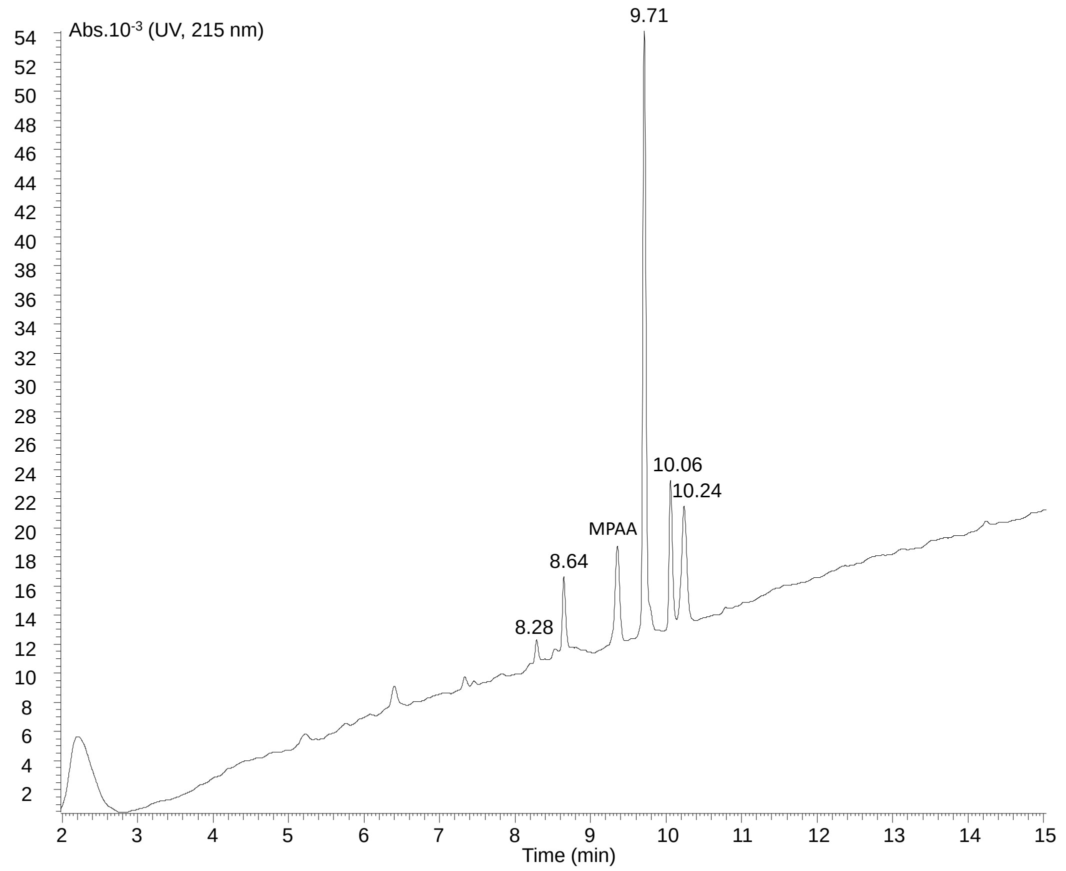

Supplementary Figure 212. UPLC trace of the reaction of peptide thioester Ac-ALKEPVHGVpSGpSA-MPAA **1a** with cysteinyl peptide CRRRRRRILKEPVHGV-NH_2 **7** (peptide concentration 5 µM, 20 mM sodium bicarbonate/CO_2 buffer, rt, pH 7.5) after 5 min. LC trace. Eluent A 0.1% TFA in water, eluent B 0.1% TFA in CH_3CN. C18 BEH 300 Å (1.7 µm, 2.1 × 100 mm) column, gradient 0-40% B in 15 min, 0.4 mL min^{-1}, detection at 215 nm).

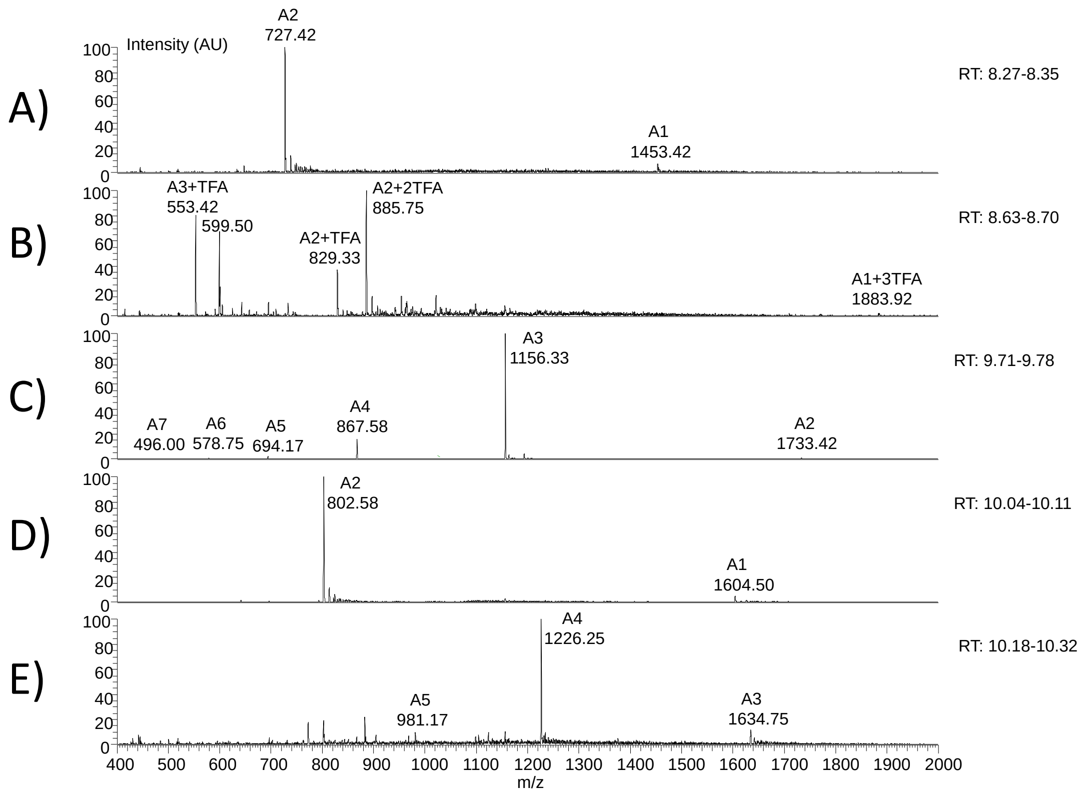

Supplementary Figure 213. MS traces from UPLC-MS analysis of the reaction of peptide thioester Ac-ALKEPVHGVpSGpSA-MPAA **1a** with cysteinyl peptide CRRRRRRILKEPVHGV-NH_2 **7** (peptide concentration 5 µM, 20 mM sodium bicarbonate/CO_2 buffer, rt, pH 7.5) after 5 min. MS traces. A) Rt = 8.28 min, peptide thioester hydrolysis byproduct Ac-ALKEPVHGVpSGpSA-OH. $[M+H]^+$ m/z calcd. (monoisotopic) 1453.60, obs. 1453.42, $[M+2H]^{2+}$ m/z calcd. (av.) 727.70, obs. 727.42. B) Rt = 8.64 min, CRRRRRRILKEPVHGV-NH_2 **7**. The peptide undergoes a fragmentation between Glu and Pro due to mass analysis conditions (CRRRRRRILKE). $[M+3TFA+H]^+$ m/z calcd. (monoisotopic) 1883.93, obs. 1883.92, $[M+TFA+2H]^{2+}$ m/z calcd. (av.) 828.95, obs. 829.33, $[M+2TFA+2H]^{2+}$ m/z calcd. (av.) 885.95, obs. 885.75, $[M+TFA+3H]^{3+}$ m/z calcd. (av.) 552.97, obs. 553.42. C) Rt = 9.71 min, target ligation product Ac-ALKEPVHGVpSGpSACRRRRRRILKEPVHGV-NH_2 **8a**. $[M+2H]^{2+}$ m/z calcd. (av.) 1733.94, obs. 1733.42, $[M+3H]^{3+}$ m/z calcd. (av.) 1156.29, obs. 1156.33, $[M+4H]^{4+}$ m/z calcd. (av.) 867.47, obs. 867.58, $[M+5H]^{5+}$ m/z calcd. (av.) 694.17, obs. 694.17, $[M+6H]^{6+}$ m/z calcd. (av.) 578.65, obs. 578.75, $[M+7H]^{7+}$ m/z calcd. (av.) 496.12, obs. 496.00. D) Rt = 10.06 min, peptide thioester Ac-ALKEPVHGVpSGpSA-MPAA **1a**. $[M+H]^+$ m/z calcd. (av.) 1604.60, obs. 1604.50, $[M+2H]^{2+}$ m/z calcd. (av.) 802.80, obs. 802.58. E) Rt = 10.24 min, diacylation product, branched thioester $[M+3H]^{3+}$ m/z calcd. (av.) 1634.75, obs. 1634.75, $[M+4H]^{4+}$ m/z calcd. (av.) 1226.32, obs. 1226.25, $[M+5H]^{5+}$ m/z calcd. (av.) 981.25, obs. 981.17.

Kinetic model

The reaction depicted in Fig 4b was monitored by UPLC-MS and HPLC to determine the rate of product formation overtime. The data were fitted using Kintek Global Kinetic Explorer Software, Version 10.0.200514, as described in the manuscript.

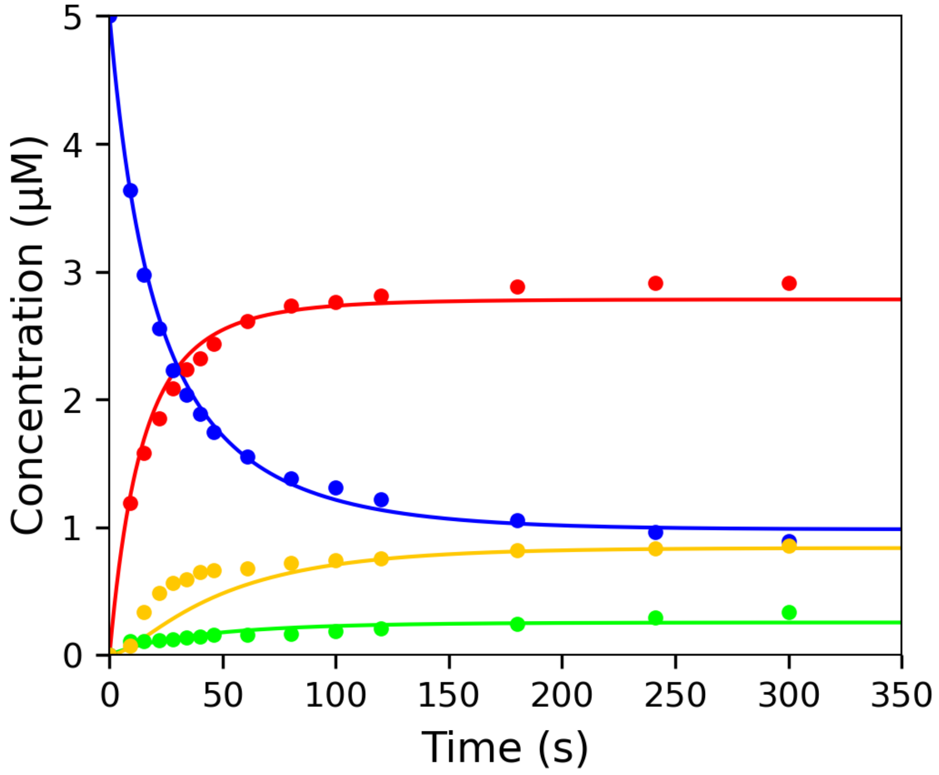

Supplementary Figure 214. Representative fitting curves of promoted NCL (sodium hydrogenocarbonate (20 mM)/CO_2 buffer, ~5 µM final concentration for each peptide, pH ~7.5, room temperature). Data were fitted according to the following model:

1a + 7 -> 8a
1a -> Hydrolysis product
8a + 1a -> Branched thioester

Experimental data points are represented by colored dots, the fitting curve by colored lines (red: NCL product **8a**, green: hydrolysis product Ac-ALKEPVHGVpSGpSA-OH, dark blue: aryl thioester **1a**, yellow: branched thioester byproduct.

Supplementary Table 3. NCL-rate constants corresponding to the formation of ligation product **8a** (k_1), hydrolysis Ac-ALKEPVHGVpSGpSA-OH (k_2) and branched thioester byproduct (k_3).[a]

Rate constant	k_{app}	σ
k_1	7918 M^{-1} s^{-1}	1407
k_2	1.43 10^{-3} s^{-1}	0.25 10^{-3}
k_3	2888 M^{-1} s^{-1}	1455

[a] Sodium hydrogenocarbonate (20 mM)/CO_2 buffer, ~5 µM final concentration for each peptide, pH 7.5, rt.

Reproducibility

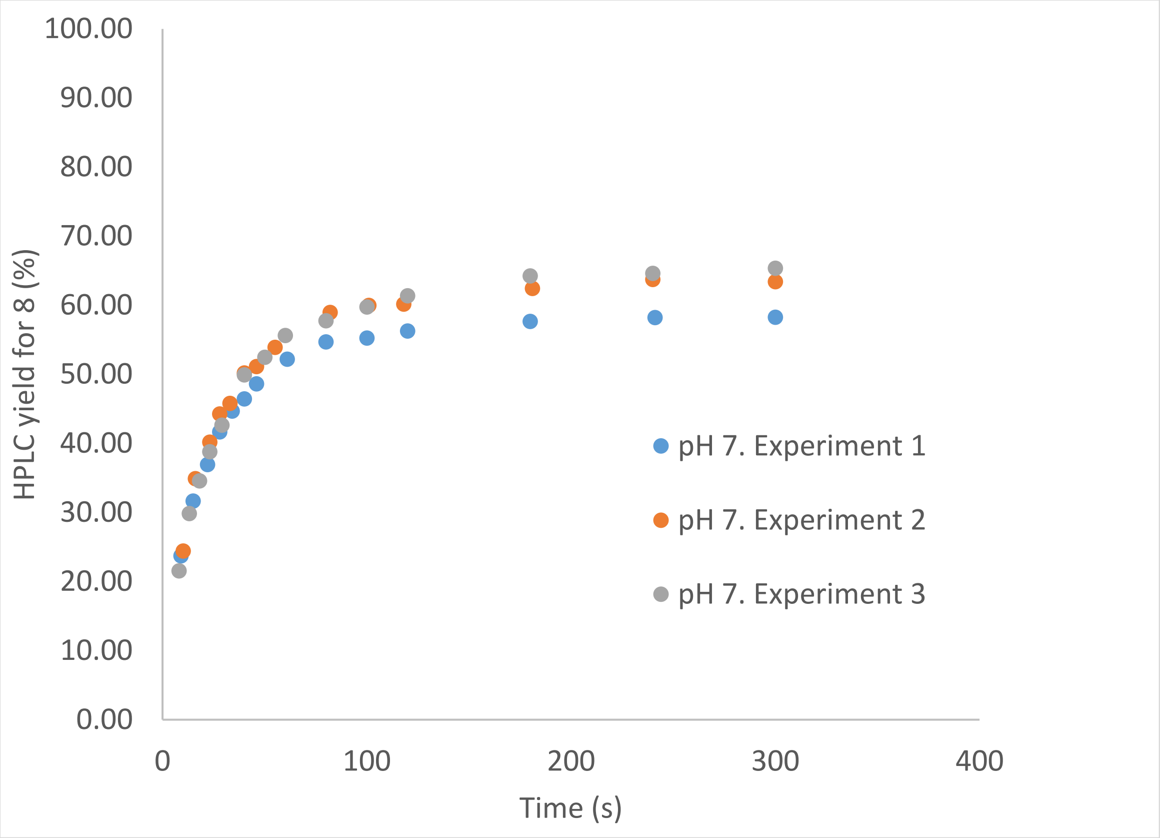

Supplementary Figure 215. Time course of the reaction of peptide thioester **1a** with cysteinyl peptide **7** as followed by HPLC.

Effect of the pH (kinetic studies)

The reaction of peptide thioester **1a** with cysteinyl peptide **7** was performed at different pH values. The pH of the reaction mixture was varied by adjusting the flow of CO_2 into the solution.

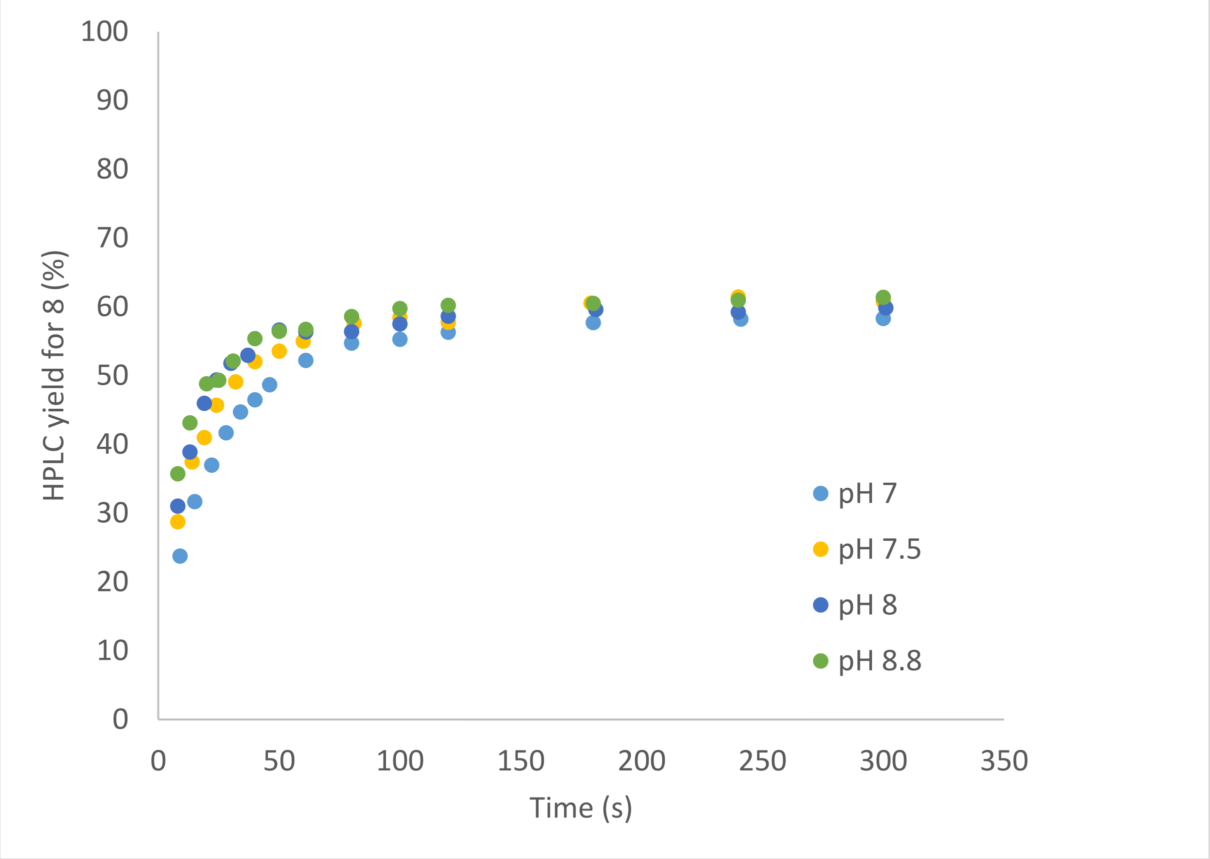

Supplementary Figure 216. Time course of the reaction of peptide thioester **1a** with cysteinyl peptide **7** as followed by HPLC. Effect of the pH.

*Control experiment with glycyl peptide **2a** (importance of side-chain thiol)*

The control experiment showing the importance of the Cys thiol group in the NCL reaction was performed using the general conditions described in the Methods section (5 µM).

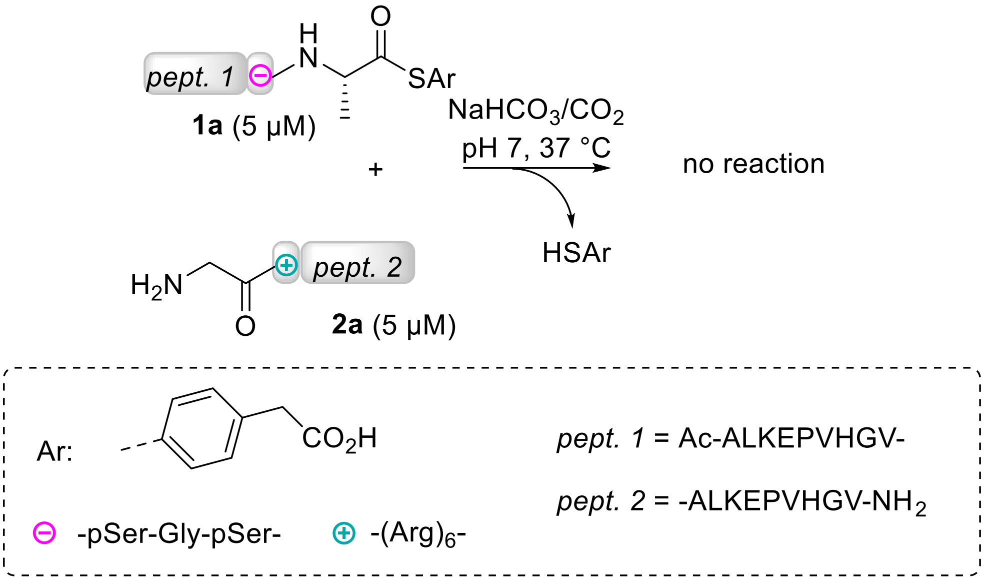

The reaction was followed by RP-HPLC. The data show that no ligation product **3a,a** is formed after 5 min under such experimental conditions due to the low concentration of the reactants.

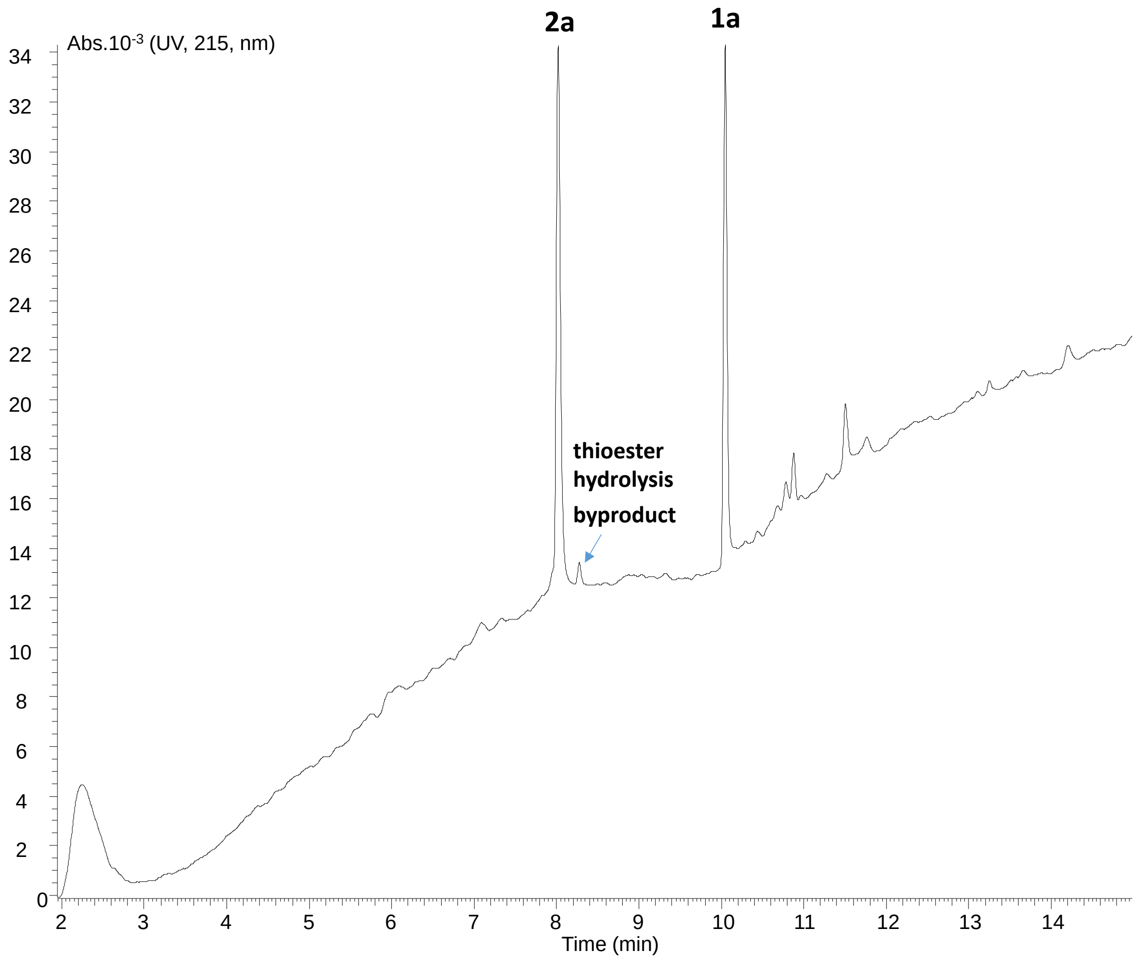

Supplementary Figure 217**.** UPLC analysis of the reaction of peptide thioester **1a** with glycyl peptide **2a** at 5 µM in 20 mM sodium bicarbonate/CO_2 buffer pH=7.12 after 6 min. LC trace. Eluent A 0.1% TFA in water, eluent B 0.1% TFA in CH_3CN. C18 BEH 300 Å (1.7 µm, 2.1 × 100 mm) column, gradient 0-40% B in 15 min, 0.4 mL min^{-1}, detection at 215 nm).

Control experiment with peptide CILKEPVHGV-NH_2 (importance of Arg_6 module)

The control experiment to probe the importance of Arg_6 module in cysteinyl peptide was performed using peptide thioester Ac-ALKEPVHGVpSGpSA-MPAA **1a** and Cys peptide CILKEPVHGV-NH_2. The protocol is identical to the one described in section 8.1 (5 µM peptide concentration, 19 °C, pH 7.1).

The reaction mixture was analysed by UPLC-MS. This experiment shows that no ligation product is formed when the Arg_6 module is absent in the Cys peptide.

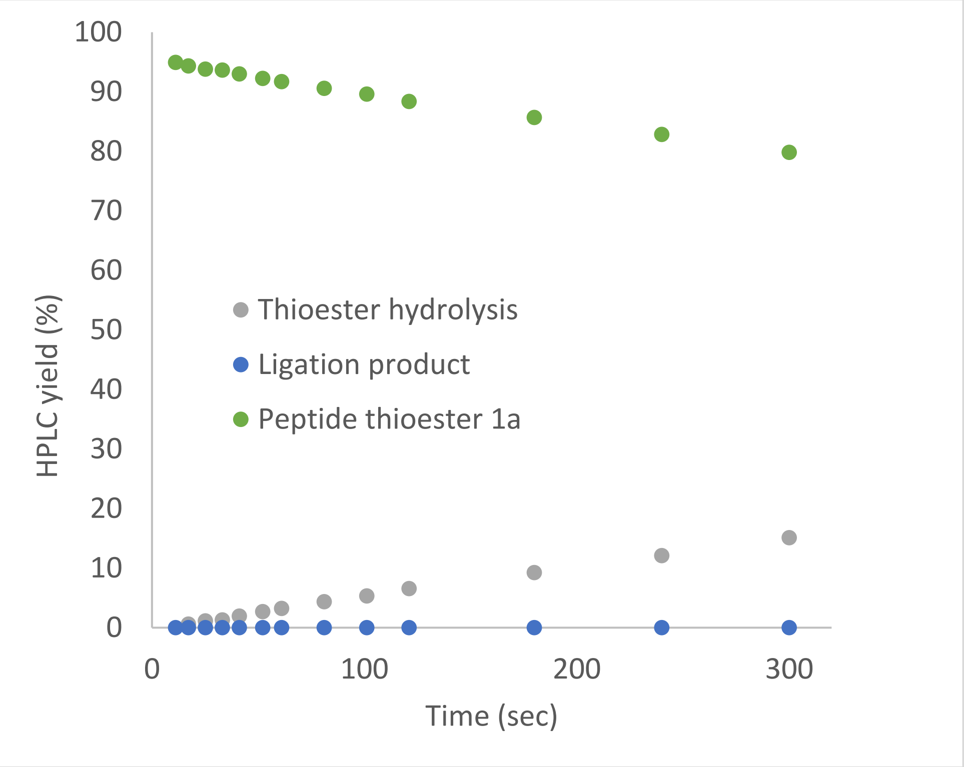

Supplementary Figure 218. The control experiment to probe the importance of Arg_6 module in the cysteinyl peptide was performed using peptide thioester Ac-ALKEPVHGVpSGpSA-MPAA **1a** and Cys peptide CILKEPVHGV-NH_2. HPLC yield for the different species observed in the reaction mixture.

*Replacement of pSer residues by glutamic acid residues (NCL ligation products **8f,g**)*

Replacement of pSer residues by glutamic acid residues is discussed in Fig. 4b,c in the manuscript.

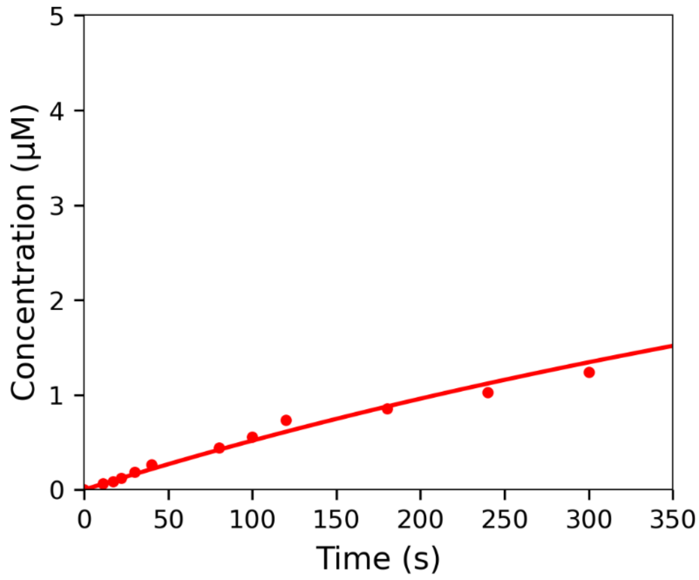

Supplementary Figure 219. Representative fitting curve of promoted NCL by EGE module (sodium hydrogenocarbonate (20 mM)/CO_2 buffer, ~5 µM final concentration for each peptide, pH ~7, room temperature). Data were fitted according to the following model:

1f + 7 -> 8f

Experimental data points are represented by colored dots, the fitting curve by colored lines.

Supplementary Table 4. Apparent second order NCL-rate constant corresponding to the formation of ligation product **8f** (k_1).[a]

Rate constant	k_{app}	σ
k_1	168 M^{-1} s^{-1}	41

[a] Sodium hydrogenocarbonate (20 mM)/CO_2 buffer, ~5 µM final concentration for each peptide, pH 7, rt.

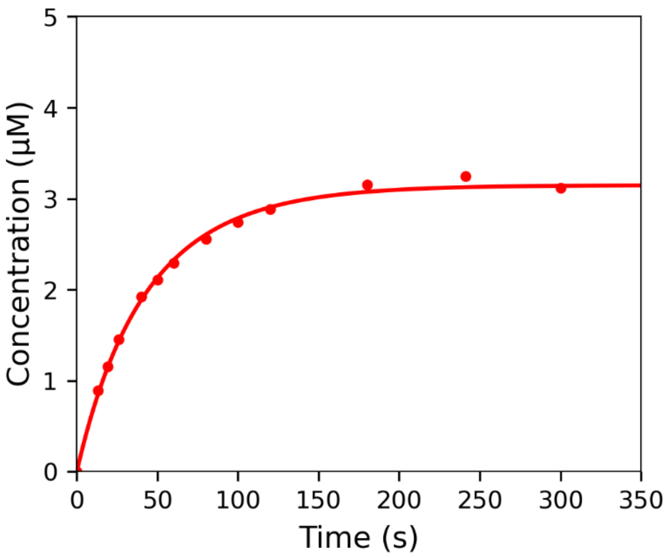

Supplementary Figure 220. Representative fitting curve of promoted NCL by EEGEE module (sodium hydrogenocarbonate (20 mM)/CO_2 buffer, ~5 µM final concentration for each peptide, pH ~7, room temperature). Data were fitted according to the following model:

1g+ 7 -> 8g

Experimental data points are represented by colored dots, the fitting curve by colored lines.

Supplementary Table 5. Apparent second order NCL-rate constant corresponding to the formation of ligation product **8g** (k_1)[a]

Rate constant	k_{app}	σ
k_1	4920 M^{-1} s^{-1}	1300

[a] Sodium hydrogenocarbonate (20 mM)/CO_2 buffer, ~5 µM final concentration for each peptide, pH 7, rt.

Influence of intramolecular electrostatic competition

Cysteinyl peptides featuring an Arg_6 positively charged module at the N-terminus and a negatively charged module at the C-terminus (made of pSer or Glu residues) were reacted with a pSer-Gly-pSer module-containing aryl thioester at 5 µM. For these NCL reactions, we used the typical experimental procedure described in the Methods section of the main manuscript.

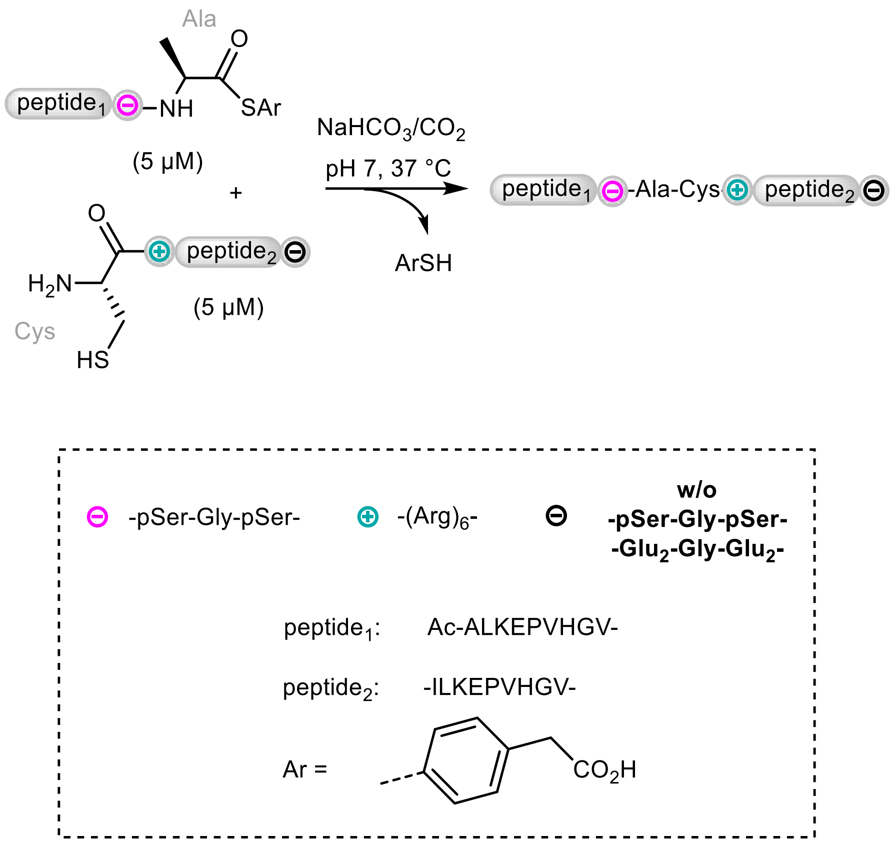

The conditions studied were:

Experiment 1: 5 µM pSer-Gly-pSer-containing peptide thioester **1a** + 5 µM Cys peptide containing an Arg_6 module and a pSer-Gly-pSer module, i.e. CRRRRRRALKEPVHGVpSGpS-NH_2, in 20 mM CO_2 buffered sodium hydrogenocarbonate solution, pH 7.01-7.05, 21.8 °C.

Experiment 2: 5 µM pSer-Gly-pSer-containing peptide thioester **1a**+ 5 µM Cys peptide containing an Arg_6 module and a $(Glu)_2$-Gly-$(Glu)_2$ module, i.e. CRRRRRRALKEPVHGVEEGEE-NH_2, in 20 mM CO_2 buffered sodium hydrogenocarbonate solution, pH 6.97-7.07, 20.3 °C

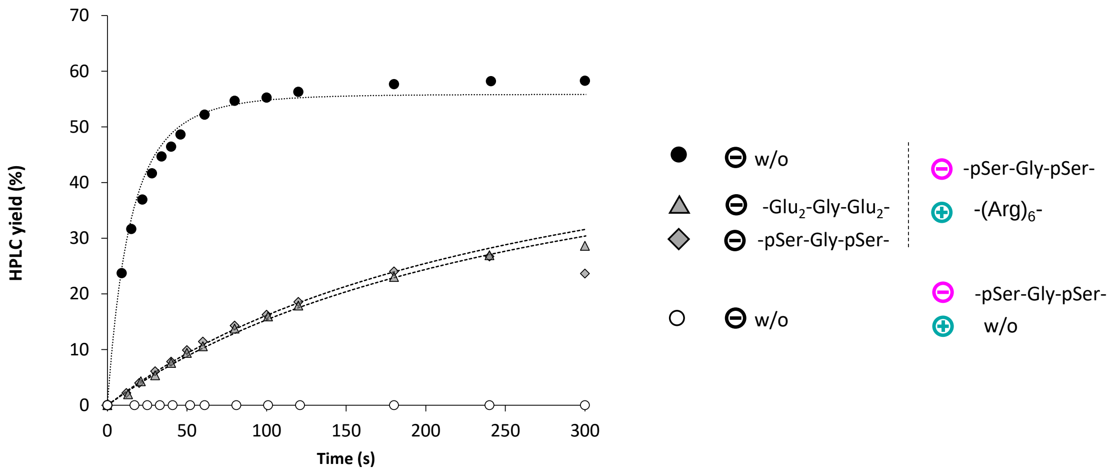

Supplementary Figure 221. Monitoring of the formation of ligation product in the presence or in the absence of a competitive intramolecular charged module in pink.

Supplementary Table 6. Apparent second order rate constants corresponding to the formation of ligation product in the presence of a competing intramolecular electrostatic interaction.

Experiment	Exp. Conditions	k_{app} ($M^{-1}.s^{-1}$)	SEM ($M^{-1}.s^{-1}$)
1	5 µM pSer-Gly-pSer-containing peptide thioester **1a** + 5 µM Cys peptide containing an Arg_6 module and a pSer-Gly-pSer module, i.e. $CRRRRRRALKEPVHGVpSGpS-NH_2$	731	79
2	5 µM pSer-Gly-pSer-containing peptide thioester **1a**+ 5 µM Cys peptide containing an Arg_6 module and a $(Glu)_2$-Gly-$(Glu)_2$ module, i.e. $CRRRRRRALKEPVHGVEEGEE-NH_2$	695	60

Synthesis of conjugate 10 (modified kringle 1 HGF domain, Fig. 4d,e)

The reaction mixture was monitored by RP-HPLC and UPLC-MS.

A)

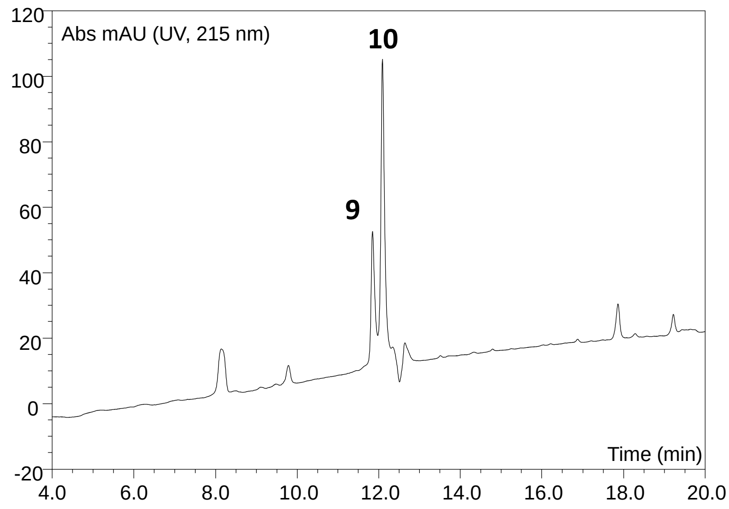

B)

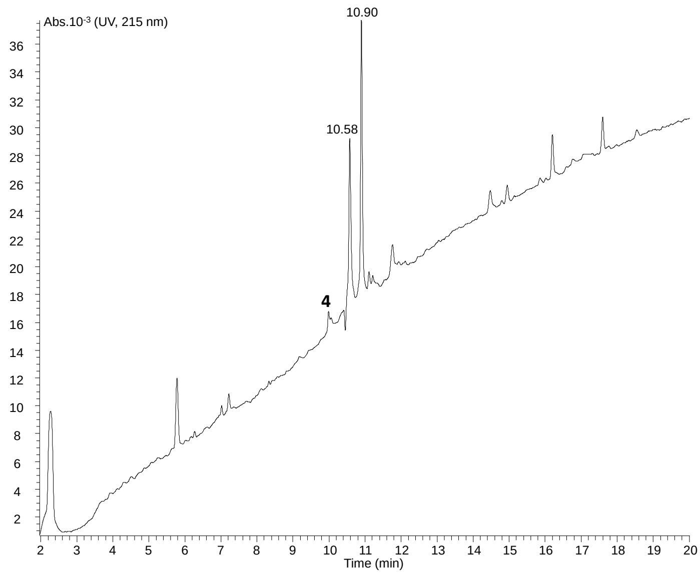

Supplementary Figure 222. Synthesis of conjugate **10**. A) RP-HPLC chromatogram of the reaction of peptide thioester **4** with and CR_6-K1 protein **9** at 1 µM after 5 min at rt (90 µL of the reaction mixture injected). B) UPLC-MS analysis of the reaction of peptide thioester **4** with and CR_6-K1 protein **9** at 1 µM after 5 min at rt (20 µL of the reaction mixture injected). LC trace. Eluent A 0.1% TFA in water, eluent B 0.1% TFA in CH_3CN. C18 BEH 300 Å (1.7 µm, 2.1 × 100 mm) column, gradient 0-70% B in 20 min, 0.4 mL min^{-1}, detection at 215 nm).

A)

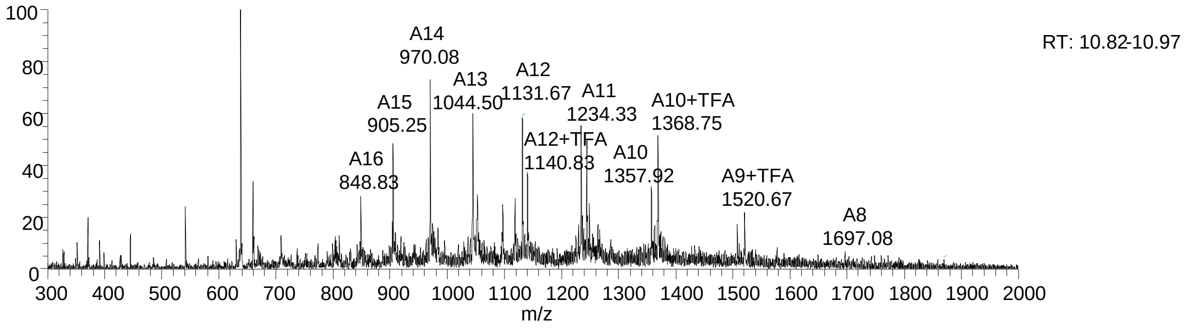

B)

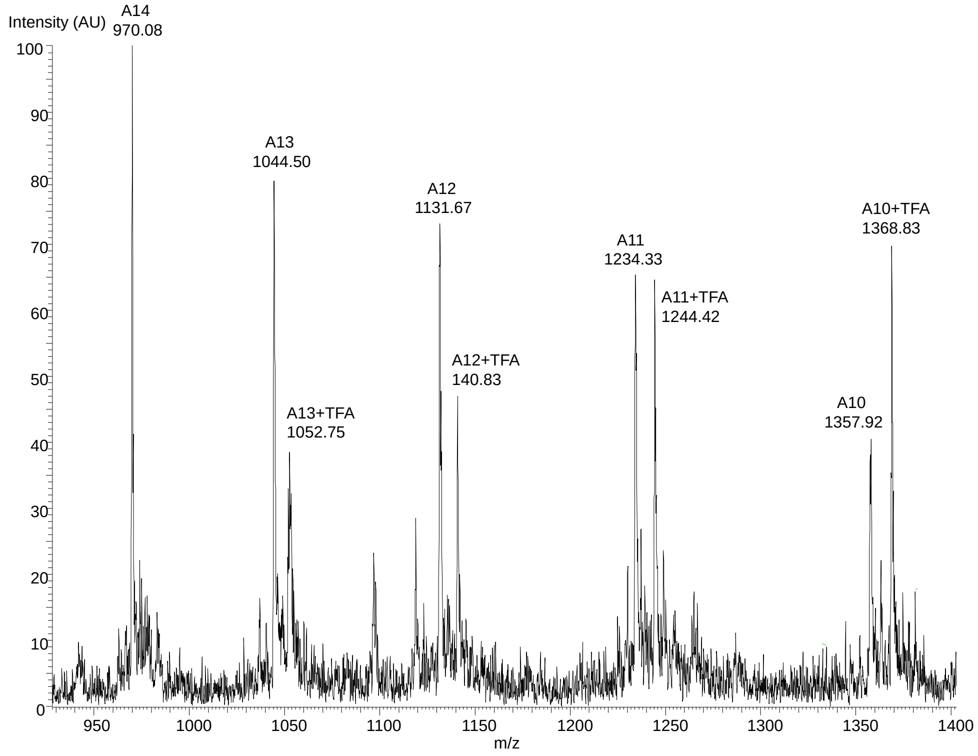

Supplementary Figure 223. Synthesis of conjugate **10**. A) MS trace from UPLC-MS analysis of the reaction of peptide thioester **4** with and CR_6-K1 protein **9** at 1 µM after 5 min at rt. MS trace of the main peak at Rt = 10.90 min corresponds to the conjugate. $[M+8H]^{8+}$ m/z calcd. (av.) 1696.76, obs. 1697.08, $[M+10H]^{10+}$ m/z calcd. (av.) 1357.61, obs. 1357.92, $[M+11H]^{11+}$ m/z calcd. (av.) 1234.28, obs. 1234.33, $[M+12H]^{12+}$ m/z calcd. (av.) 1131.51, obs. 1131.67, $[M+13H]^{13+}$ m/z calcd. (av.) 1044.54, obs. 1044.50, $[M+14H]^{14+}$ m/z calcd. (av.) 970.01, obs. 970.08, $[M+15H]^{15+}$ m/z calcd. (av.) 905.40, obs. 905.25, $[M+16H]^{16+}$ m/z calcd. (av.) 848.88, obs. 848.83. B) Zoom of MS trace.

The reaction mixture was also monitored by SDS-PAGE. Aliquots of the reaction mixture (19 µL) were mixed with an aqueous solution of $CR_6ILKEPVHGV\text{-}NH_2$ peptide **7** (1 mM in 0.1 M sodium phosphate buffer, 1 µL) to quench the reaction by consuming the remaining peptide thioester **4**. The solution was vortexed and 1 µL of this solution was diluted with water (12 µL), RED 10X (2 µL of a mix of 2.45 M DTT and 2.35 M β-mercaptoethanol) and NuPAGE (5 µL, Invitrogen, LDS sample buffer 4X) and immediately frozen in liquid nitrogen. The sample was stored at -80 °C until SDS-PAGE and western blot analysis using streptavidin-horseradish peroxidase. The gels show that the starting CR_6-K1 protein **9** and conjugate **10** are not revealed similarly by Western blot using streptavidin-HRP. Therefore, SDS-PAGE cannot be used to estimate the extent of target product **10** formation. Alkylation of Cys residues with iodoacetamide prior to analysis had almost no effect on the band intensity.

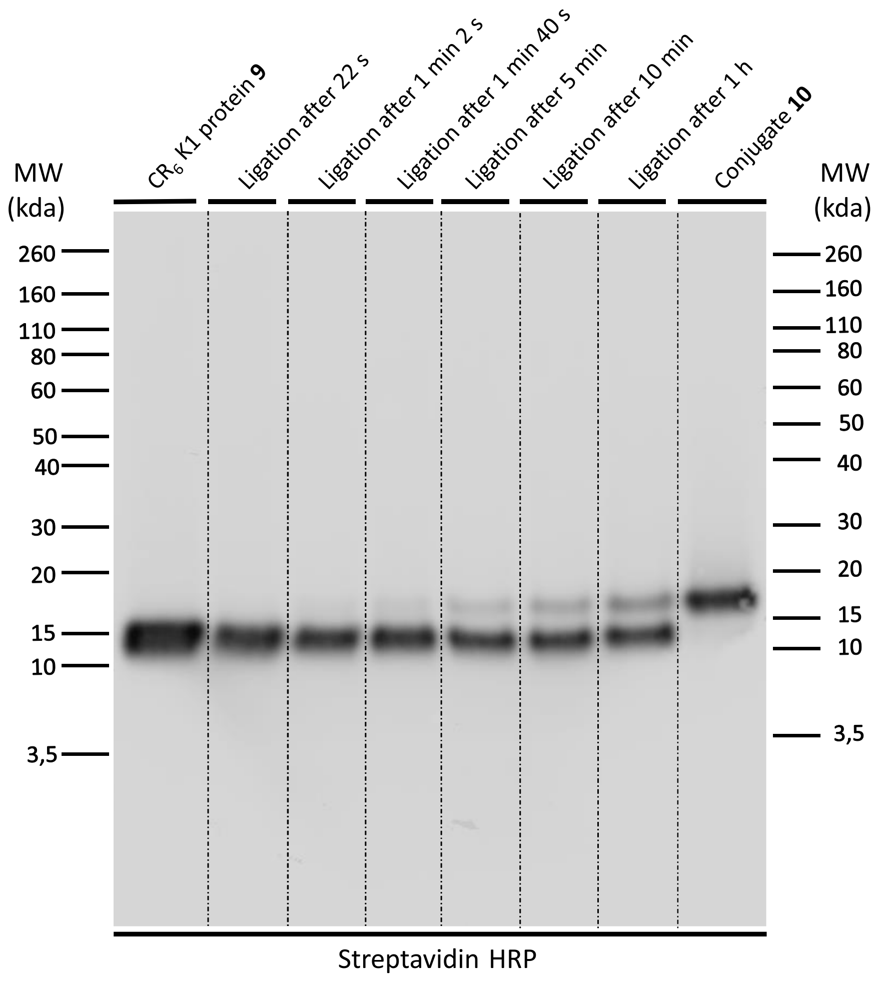

Supplementary Figure 224. Western blot analysis of the reaction of peptide thioester **4** with and CR_6-K1 protein **9** at 1 µM at rt (10.2 ng CR_6-K1 protein **9** loaded and 10.7 ng conjugate **10** loaded).

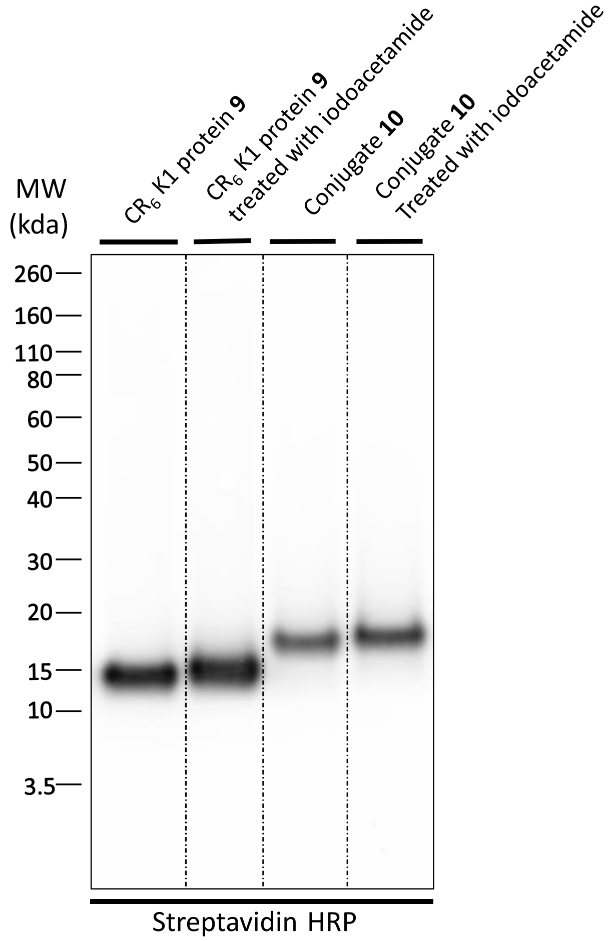

Supplementary Figure 225. SDS-PAGE analysis of purified CR$_6$-K1 protein **9** and conjugate **10** pre-treated or not with iodoacetamide (10.2 ng CR$_6$-K1 protein **9** loaded and 10.7 ng conjugate **10** loaded).

Ligation of CR$_6$-K1 protein 9 with peptide thioester 1g

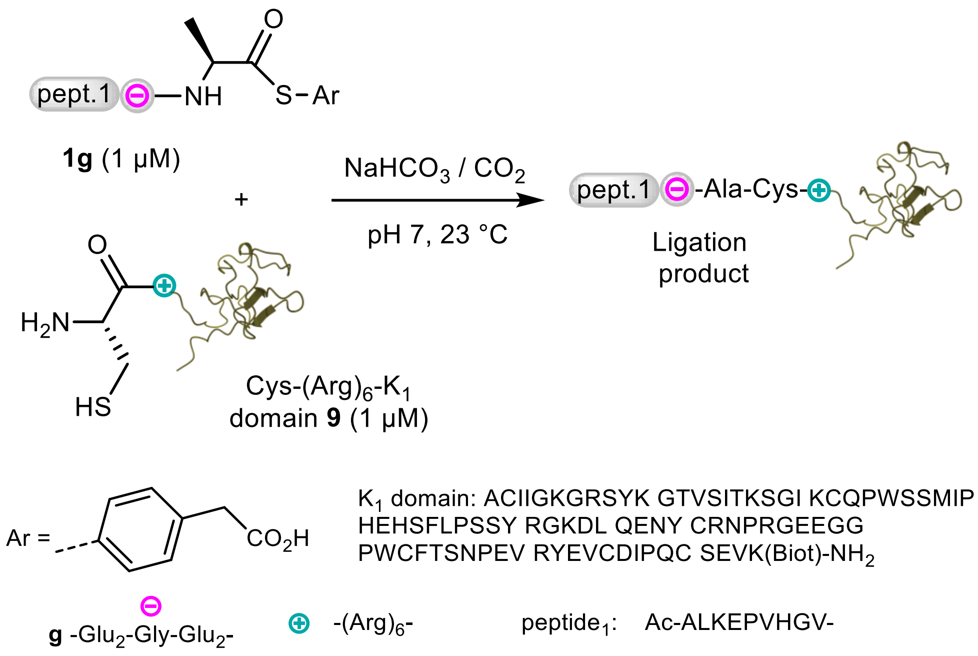

The procedure is similar to the one used for performing NCL at 5 µM with some adaptations. The reaction was performed at room temperature (19-20 °C). Sodium bicarbonate (5.04 mg, 20 mM final concentration) was dissolved in water (3 mL). The ligation was done at 1 µM protein concentration using a 5 mL low bind plastic tube equipped with a magnetic stirrer (600 rpm). The plastic tube was filled with the sodium bicarbonate solution (3 mL), which was supplemented with TCEP·HCl (858 µg, 1 mM final concentration) and *n*-octylglucoside (8.62 mg, 10 mM final concentration) to prevent the oxidation and precipitation of the protein. The flow of CO_2 was adjusted to obtain a pH of 7.14-7.23. CR$_6$-K1 protein **9** (1 µL of a 3 mM stock solution in water, 3 nmol, 1 µM final concentration) was added under stirring (600 rpm) to the buffer in the plastic tube. A few seconds later, the peptide thioester **1g** (1 µL of a 2.99 mM stock solution in water, 2.99 nmol, 1 µM final concentration) was added to the mixture under stirring (600 rpm). The reaction mixture was analysed by UPLC-MS. For this, an aliquot of the reaction mixture (100 µL) was quenched with glacial AcOH (20 µL) and injected directly into the LC-MS system.

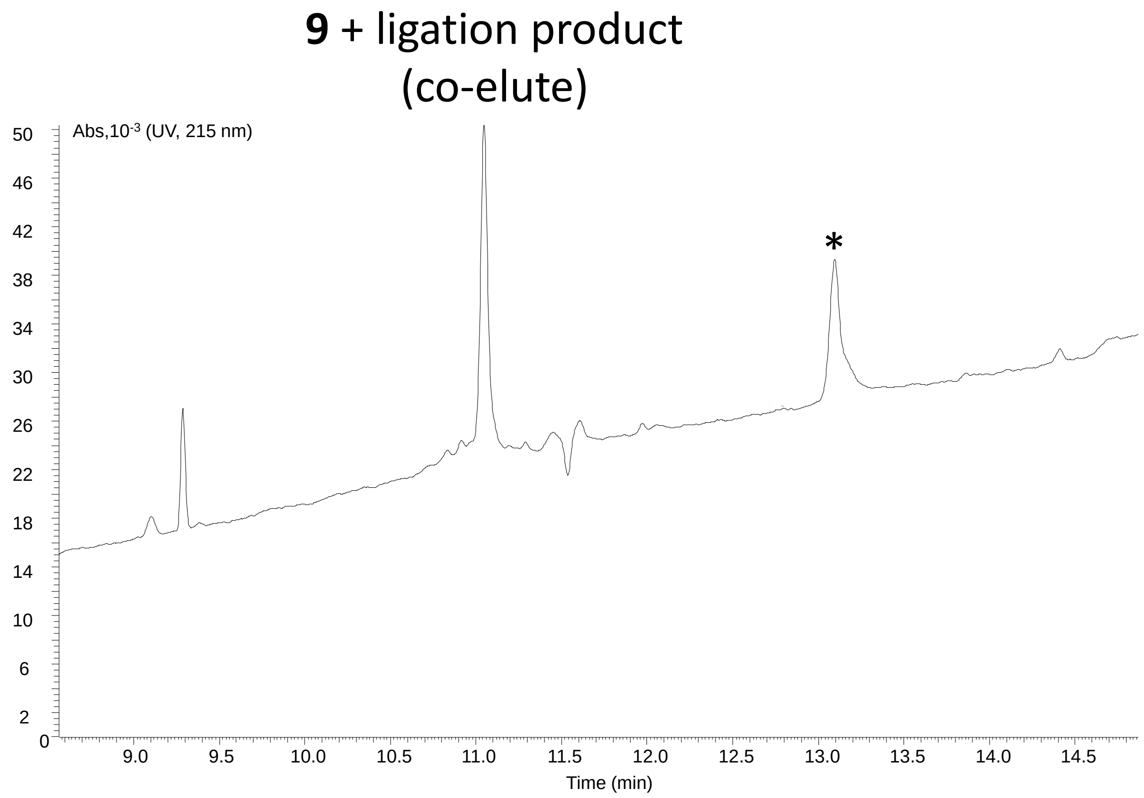

Supplementary Figure 226. UPLC analysis of the ligation mixture between CR_6 K1 polypeptide **9** and peptide **1g**. LC trace.

A)

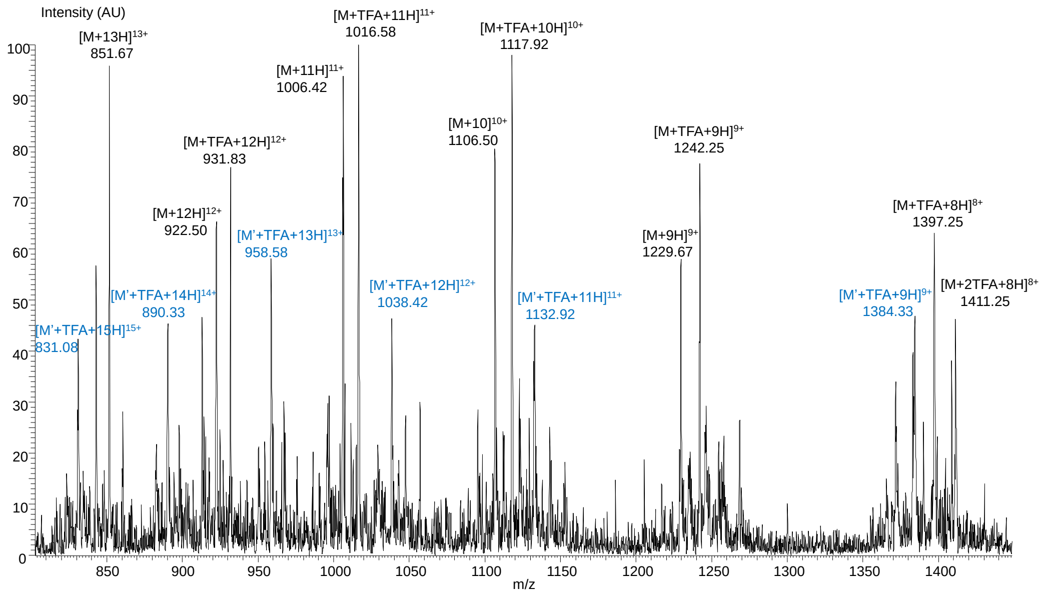

B)

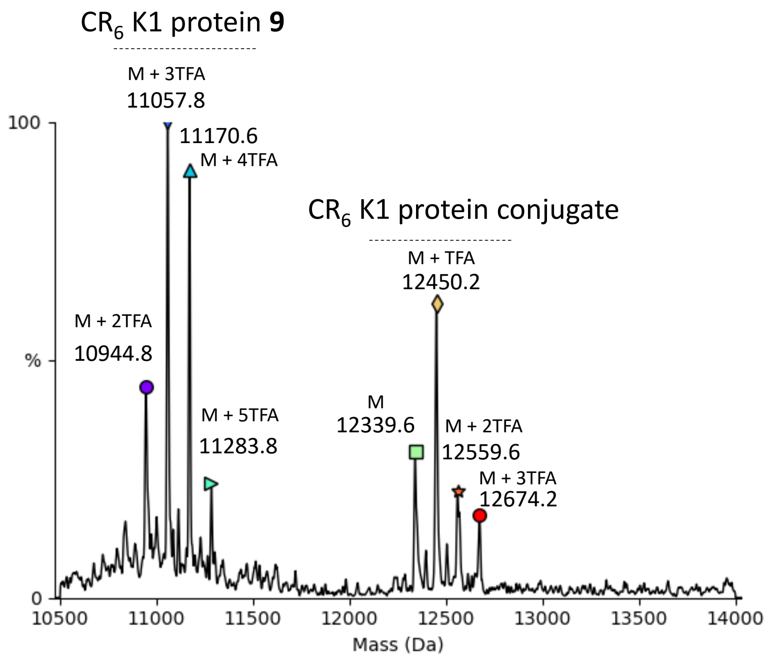

Supplementary Figure 227. UPLC-MS analysis of the ligation mixture between CR_6 K1 polypeptide **9** and peptide **1g**. A) MS trace for the main peak. The peaks corresponding to ligation product are in blue. $[M+TFA+9H]^{9+}$ m/z calcd. (av.) 1384.11, obs. 1384.33, $[M+TFA+11H]^{11+}$ m/z calcd. (av.) 1132.63, obs. 1132.92, $[M+TFA+12H]^{12+}$ m/z calcd. (av.) 1038.33, obs. 1038.42, $[M+TFA+13H]^{13+}$ m/z calcd. (av.) 958.54, obs. 958.58, $[M+TFA+14H]^{14+}$ m/z calcd. (av.) 890.14, obs. 890.33, $[M+TFA+15H]^{15+}$ m/z calcd. (av.) 830.86, obs. 831.08. B) Deconvoluted MS spectrum of the peak corresponding to CR_6-K1 protein **9** (M + 2 TFA calcd. (av.) 10950.3, obs. 10944.8) and ligation product (M calcd. (av.) 12340.02, obs. 12339.6). The multiplicity of the peaks is due to TFA adducts.

Electrostatic assistance of hydrazone ligation

Arg·HCl catalyzed, UV monitoring

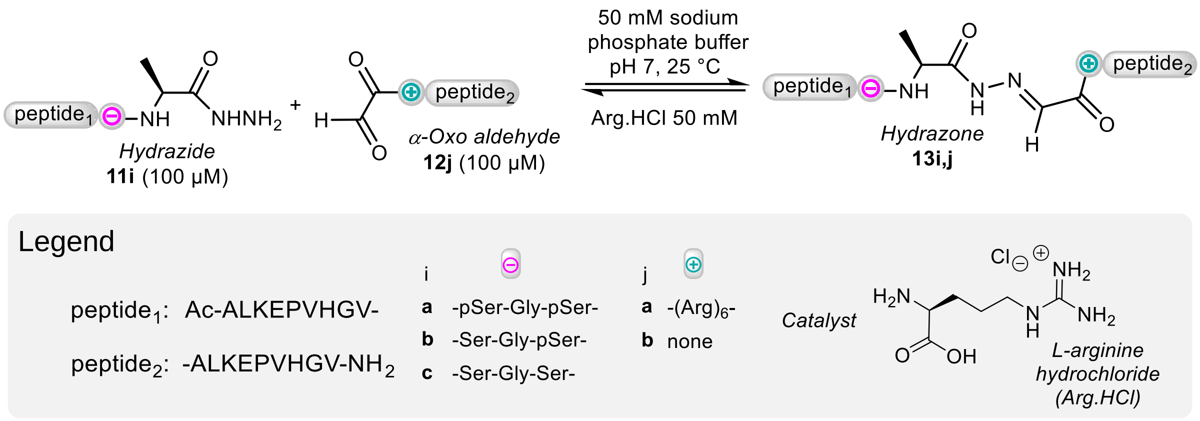

Kinetic data collection

Each kinetic experiment was performed in sextuplicate in the same 96-well plate. The blank was recorded in sextuplicate too. For some experimental conditions that are indicated below, the experiment was repeated in different 96-well plates to judge of the inter-plate variability of the measured k_{app}.

The concentrations of the starting peptide solutions were determined by measuring the UV absorbance at 205 nm using a UV spectrophotometer (Jenway 7315 Spectrophotometer, 1 cm quartz cuvettes).

The reactions were performed in 96-well plates (UV-star microplate 96 well, COC, F-bottom chimney well, µClear, clear, Greiner bio-one) at 25 °C. Hydrazone **13** formation was monitored on real-time by reading the absorbance at 285 nm using an ENVISION multimode microplates reader (Perkin Elmer).

Typical experimental procedure for hydrazone ligation in 96-well plates: Concentrated solutions of peptide hydrazide Ac-ALKEPVHGVpSGpSA-$NHNH_2$ **11a** and CHOCO-RRRRRRALKEPVHGV-NH_2 peptide **12a** were diluted in the appropriate buffer (~0.1 mM final concentration for each peptide, pH 7.00-7.03, 25 °C) and filtered on Nalgene 4 mm syringe filters (0.45 µm, cellulose acetate membrane). The volume of the reaction mixture in each well was 200 µL (185 µL buffer, 10 µL peptide hydrazide solution and 5 µL CHOCO peptide solution). The solutions were delivered to the wells using a multichannel micropipette (pipetman, Eppendorf Research Pro). The peptide hydrazide **11a** was delivered first, followed by the buffer. The CHOCO peptide **12a** was then deposited on the walls of the wells. The 96-well plate was centrifuged quickly (Eppendorf centrifuge 5810R) at 1000 rpm to quickly mix the solutions and trigger hydrazone formation (defined as t = 0). The plate was immediately sealed with a coverslip and placed in the ENVISION multimode microplate reader and read at 285 nm each 20 min. For more details see: [8].

The conditions studied were: 50 mM phosphate buffer, 50 mM arginine, pH 7.00, 25 °C.

Experiment 1: 0.098 mM peptide hydrazide **11c** + 0.099 mM peptide aldehyde **12b**

Experiment 2: 0.098 mM peptide hydrazide **11c** + 0.101 mM peptide aldehyde **12a**

Experiment 3: 0.105 mM peptide hydrazide **11b** + 0.099 mM peptide aldehyde **12b**

Experiment 4: 0.105 mM peptide hydrazide **11b** + 0.101 mM peptide aldehyde **12a**

Experiment 5: 0.098 mM peptide hydrazide **11a** + 0.099 mM peptide aldehyde **12b**

Experiment 6: 0.098 mM peptide hydrazide **11a** + 0.101 mM peptide aldehyde **12a**

Calibration curve

Prior to kinetic data analysis and fitting, absorbances measured for all experiments were converted to concentrations using the calibration curve shown below. Aliquots of ligation mixtures performed in 96-wells microtiter plates were analyzed by HPLC to determine the ligation yield, from which the hydrazone concentration was calculated based on the starting peptide hydrazide **11i** and CHOCO peptide **12j** concentrations used in the experiments (determined by UV).

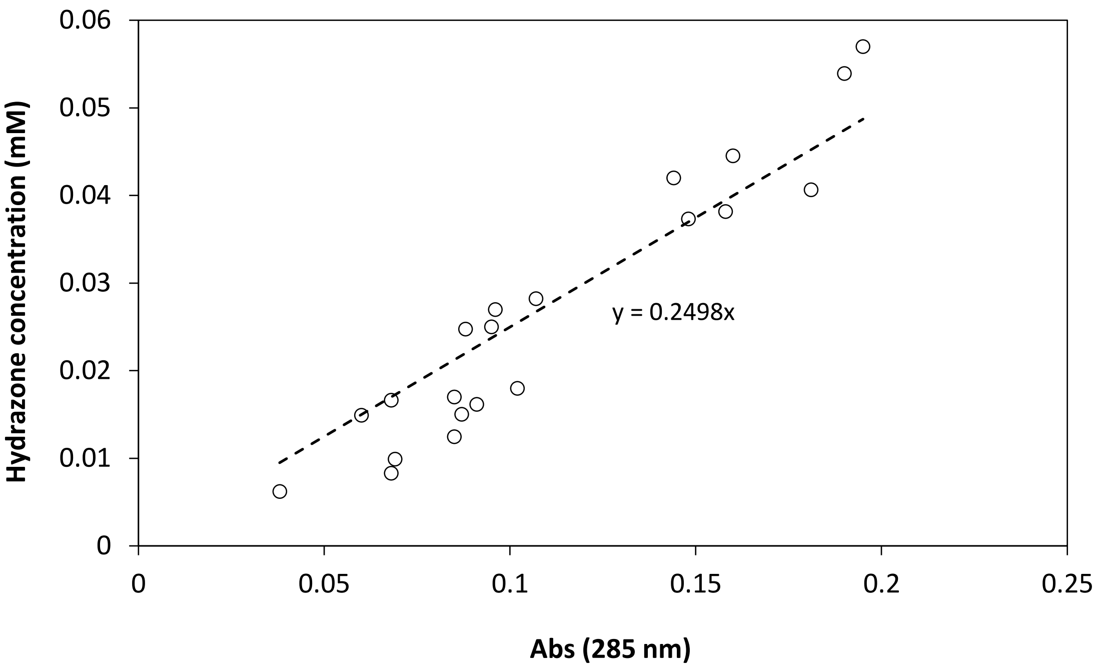

Supplementary Figure 228. Calibration curve used to convert the absorbance at 285 nm into concentrations for the formed hydrazone. The reactions were performed in 96-well plates (UV-star microplate 96 well, COC, F-bottom chimney well, µClear, clear, Greiner bio-one) at 25 °C (200 µL). The absorbance was determined at 285 nm using an ENVISION multimode microplates reader (Perkin Elmer).

Fitting

Kintek Global Kinetic Explorer Software, Version 10.0.200514, was used for kinetic modelization. The model reaction used for numerical fitting of the hydrazone ligation corresponds to a second-order reaction in the form of A + B $\rightarrow$ P.

The standard deviation for each trace was first estimated upon fitting the experimental dataset with an analytical function (3-exponential) so as to determine an average sigma value, further used for numerical data fitting. The subsequent numerical fit allowed determining the apparent second order rate constant k_{app} for each experiment. Fitting to a given model was achieved by nonlinear regression analysis based upon an iterative search to find a set of reaction parameters that gives a minimum χ^2. The process was completed by careful visual examination of the fits.

Fit interval

For each experiment, all the data points would normally be fitted. However, in the case of reactions which tend to reach the plateau long before the end of data acquisition, the process appears to be under the control of two distinct kinetic regimes. The reaction is first dominated by a fast process corresponding to the formation of the hydrazone product until a maximum

absorbance value A_{max} is reached. Then, a very slow decay of the absorbance signal can be observed due to a slow secondary chemical process leading to reversal of hydrazone formation.

Although the extent of this secondary process remains low, the quality of the fits can be significantly altered and results in overestimated apparent rate constants. Therefore, in such cases, the fit interval was reduced to data points of the ascending phase for which $A < 0.95 \times A_{max}$, so as to provide a better estimate of the kinetics of formation of the hydrazone.

Predicted plateau values provided hereafter were estimated from the maximum value attained by the fitting curve after 105 min.

Error

The data are presented mean ± SEM with a 95% confidence limit interval defined by

$$\bar{x} \pm (t\sigma_x / \sqrt{N})$$

where $\bar{x}$ is the sample mean; t the numerical factor obtained from the Student's t-distribution for a 95% confidence limit; σ_x the standard deviation of the sample and N the sample size.

Fitting curves and rate constants

Representative fitting curves of hydrazone **13i,j** formation can be found in the figure below.

A)

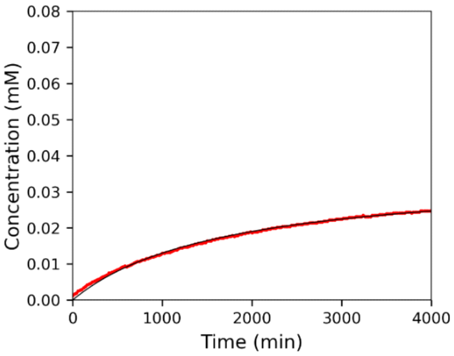

Experiment 1 (peptide hydrazide **11c** + peptide aldehyde **12b)**

B)

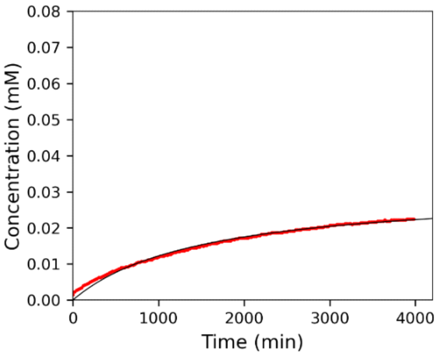

Experiment 2 (peptide hydrazide **11c** + peptide aldehyde **12a)**

C)

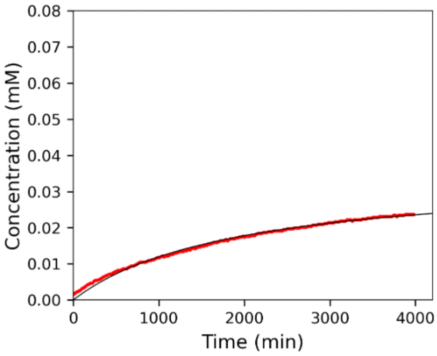

Experiment 3 (peptide hydrazide **11b** + peptide aldehyde **12b)**

D)

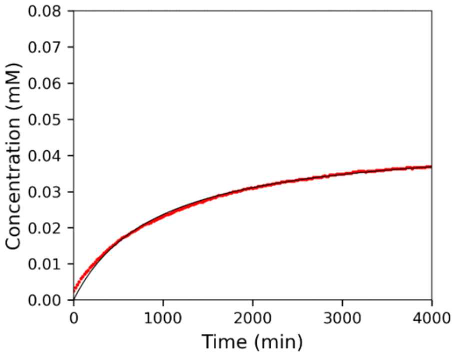

Experiment 4 (peptide hydrazide **11b** + peptide aldehyde **12a)**

E)

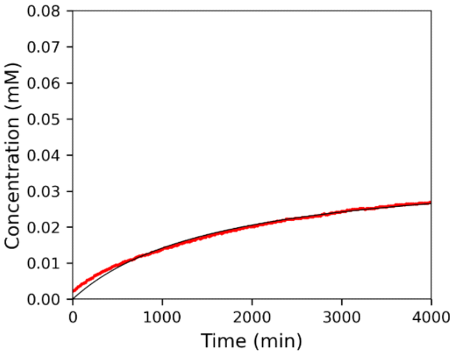

Experiment 5 (peptide hydrazide **11a** + peptide aldehyde **12b)**

F)

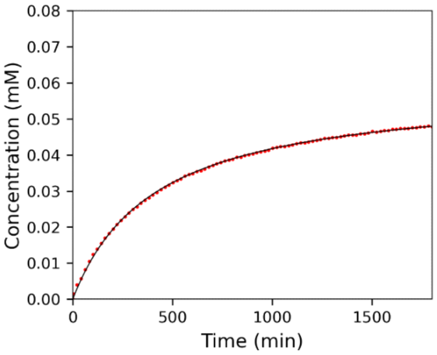

Experiment 6 (peptide hydrazide **11a** + peptide aldehyde **12a)**

Supplementary Figure 229. Representative fitting curves of hydrazone **13i,j** formation. Experimental data points are represented by red dots, the fitting curve by a black line. 0.1 mM final concentration for each peptide, 50 mM phosphate buffer, 50 mM arginine, pH 7.00, 25 °C. The reactions were performed in sextuplicate in 96-well microtiter plates. One representative set of data is shown above for each experiment (Panels A to F correspond to experiments 1 to 6, respectively).

The apparent second-order rate constants can be found in the Supplementary Table below.

Supplementary Table 7. Apparent second order rate constants and extrapolated conversion for hydrazone **13i,j** formation.[a]

Experiment	Ligation product	Exp. conditions	k_{app} ($M^{-1}.min^{-1}$)	SEM ($M^{-1}.min^{-1}$)	Plateau (%)[b]	SEM
1	**13c,b**	0.098 mM peptide hydrazide **11c** + 0.099 mM peptide aldehyde **12b**	6.27	0.50	34.9	0.54
2	**13c,a**	0.098 mM peptide hydrazide **11c** + 0.101 mM peptide aldehyde **12a**	6.45	0.45	30.2	0.42
3	**13b,b**	0.105 mM peptide hydrazide **11b** + 0.099 mM peptide aldehyde **12b**	5.32	0.26	33.0	0.80
4	**13b,a**	0.105 mM peptide hydrazide **11b** + 0.101 mM peptide aldehyde **12a**	9.84	0.77	44.2	0.99
5	**13a,b**	0.098 mM peptide hydrazide **11a** + 0.099 mM peptide aldehyde **12b**	6.06	0.39	37.6	1.04
6	**13a,a**	0.098 mM peptide hydrazide **11a** + 0.101 mM peptide aldehyde **12a**	24.40	1.02	58.3	1.44

[a] 0.1 mM final concentration for each peptide, 50 mM phosphate buffer, 50 mM arginine, pH 7.00, 25 °C. The reactions were performed in sextuplicate in 96-well microtiter plates. The data correspond to the mean ± standard error (95% confidence limit interval).

[b] Extrapolated from the fit at 50.000 min.

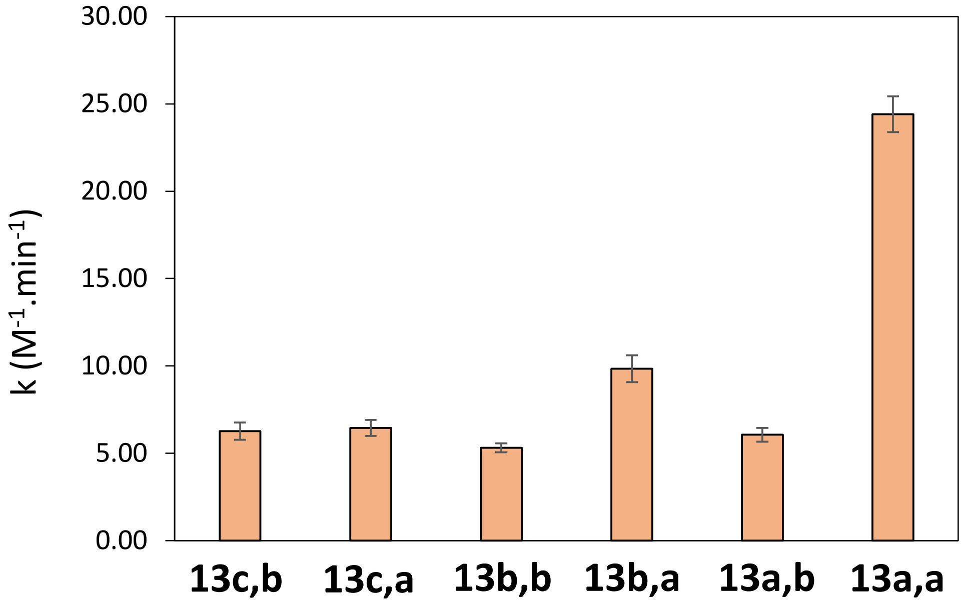

Supplementary Figure 230. Influence of electrostatic assistance on hydrazone formation. The presence of both pSer-Gly-pSer and Arg_6 modules results in significant rate enhancement of the ligation process. Data are extracted from **Supplementary Table 7**. 0.1 mM final concentration for each peptide, 50 mM phosphate buffer, 50 mM arginine, pH 7.00, 25 °C. The reactions were performed in sextuplicate in 96-well microtiter plates. The data correspond to the mean ± standard error (95% confidence limit interval).

2. Supplementary References

1. Zheng, J.-S., Tang, S., Qi, Y.-K., Wang, Z.-P. & Liu, L. Chemical synthesis of proteins using peptide hydrazides as thioester surrogates. *Nat. Protocols* 8, 2483-2495 (2013).
2. Stavropoulos, G., Gatos, D., Magafa, V. & Barlos, K. Preparation of polymer-bound trityl-hydrazines and their application in the solid phase synthesis of partially protected peptide hydrazides. *Lett. Pept. Sci.* 2, 315-318 (1996).
3. Bouchenna, J., Sénéchal, M., Drobecq, H., Vicogne, J. & Melnyk, O. Total Chemical Synthesis of All SUMO-2/3 Dimer Combinations. *Bioconjugate Chem.* 30, 2967-2973 (2019).
4. Ollivier, N. et al. A one-pot three-segment ligation strategy for protein chemical synthesis. *Angew. Chem. Int. Ed.* 51, 209-213 (2012).
5. Simonneau, C. et al. Semi-synthesis of a HGF/SF kringle one (K1) domain scaffold generates a potent in vivo MET receptor agonist. *Chem. Sci.* 6, 2110-2121 (2015).
6. Boll, E. et al. One-pot chemical synthesis of small ubiquitin-like modifier (SUMO) protein-peptide conjugates using *bis*(2-sulfanylethyl)amido peptide latent thioester surrogates *Nat. Protoc.* 10, 269-292 (2015).
7. Schreiber, G. & Fersht, A.R. Rapid, electrostatically assisted association of proteins. *Nat. Struct. Biol.* 3, 427-431 (1996).
8. Ollivier, N. et al. Catalysis of Hydrazone and Oxime Peptide Ligation by Arginine. *Org Lett* 22, 8608-8612 (2020).
